# Pangenome Analysis of *Mycobacterium tuberculosis* Reveals Core-Drug Targets and Screening of Promising Lead Compounds for Drug Discovery

**DOI:** 10.3390/antibiotics9110819

**Published:** 2020-11-17

**Authors:** Hamza Arshad Dar, Tahreem Zaheer, Nimat Ullah, Syeda Marriam Bakhtiar, Tianyu Zhang, Muhammad Yasir, Esam I. Azhar, Amjad Ali

**Affiliations:** 1 Atta-ur-Rahman School of Applied Biosciences, National University of Sciences and Technology, Islamabad 44000, Pakistan; darhamza000@gmail.com (H.A.D.); zaheertahreem@gmail.com (T.Z.); nimatscholar@gmail.com (N.U.); 2 Department of Bioinformatics and Biosciences, Capital University of Science and Technology Islamabad expressway, Kahuta Road, Zone-V, Islamabad 44000, Pakistan; marriam@cust.edu.pk; 3 State Key Laboratory of Respiratory Disease, Guangzhou Institutes of Biomedicine and Health (GIBH), Chinese Academy of Sciences, Guangzhou 510530, China; zhang_tianyu@gibh.ac.cn; 4 Special Infectious Agents Unit, King Fahd Medical Research Center, King Abdulaziz University, Jeddah 21589, Saudi Arabia; yamuhammad@kau.edu.sa; 5 Medical Laboratory Technology Department, Faculty of Applied Medical Sciences, King Abdulaziz University, Jeddah 21589, Saudi Arabia

**Keywords:** *Mycobacterium tuberculosis*, pangenome, drug targets, molecular docking, lead compounds, drug discovery, tuberculosis

## Abstract

Tuberculosis, caused by *Mycobacterium tuberculosis* (*M. tuberculosis*), is one of the leading causes of human deaths globally according to the WHO TB 2019 report. The continuous rise in multi- and extensive-drug resistance in *M. tuberculosis* broadens the challenges to control tuberculosis. The availability of a large number of completely sequenced genomes of *M. tuberculosis* has provided an opportunity to explore the pangenome of the species along with the pan-phylogeny and to identify potential novel drug targets leading to drug discovery. We attempt to calculate the pangenome of *M. tuberculosis* that comprises a total of 150 complete genomes and performed the phylo-genomic classification and analysis. Further, the conserved core genome (1251 proteins) is subjected to various sequential filters (non-human homology, essentiality, virulence, physicochemical parameters, and pathway analysis) resulted in identification of eight putative broad-spectrum drug targets. Upon molecular docking analyses of these targets with ligands available at the DrugBank database shortlisted a total of five promising ligands with projected inhibitory potential; namely, 2′deoxy-thymidine-5′-diphospho-alpha-d-glucose, uridine diphosphate glucose, 2′-deoxy-thymidine-beta-l-rhamnose, thymidine-5′-triphosphate, and citicoline. We are confident that with further lead optimization and experimental validation, these lead compounds may provide a sound basis to develop safe and effective drugs against tuberculosis disease in humans.

## 1. Introduction

According to the 2019 global World Health Organization (WHO) report, tuberculosis (TB) is among the top ten causes of death and the leading cause of a single infectious agent (above HIV/AIDS) [1]. In 2018, this disease was responsible for 1.2 million deaths among HIV-negative people and an additional 251,000 deaths among HIV-positive people. The causative agent of human TB is *Mycobacterium tuberculosis* (*M. tuberculosis*).

TB is majorly an airborne disease where *M. tuberculosis* establishes infection inside the human body by overcoming the immune responses directed by the host against the foreign pathogen. Once inside, the bacteria infiltrate the host macrophages and persist inside them for years thus causing a chronic infection that reflects the failure of host immunity [2]. The increased occurrence of multidrug resistant (MDR) and extensively drug-resistant (XDR) strains of *M. tuberculosis* has been attributed to spontaneous mutations in the bacterial genome, followed by the emergence of these mutant strains at the expense of wild type strains [3]. This, in turn, has led to the loss of effectiveness of standard anti-TB drugs isoniazid and rifampicin.

The availability of the first *M. tuberculosis* genome sequence in 1998 along with further developments in the genomics and associated disciplines has enabled us to understand the importance of many proteins encoded in its genome [4,5]. Especially, the concept of pangenome can now be explored to obtain the core genome, i.e., genes present in all the strains of the dataset [6]. These genes can be exploited to design broad-spectrum therapeutics against the pathogenic species.

Various protocols in bioinformatics have helped scientists to process biological data of pathogens in order to prioritize drug targets, while the parallel developments in the cheminformatics help researchers access ligand databases such as the DrugBank and perform virtual screening for in silico-aided drug discovery [7,8]. Utilizing these useful tools, scientists have endeavored to identify lead compounds for tuberculosis [9,10]. Therefore, in this study, we used the integrative approach of pangenome analyses, subtractive genomics, and bioinformatics to prioritize potential drug targets in the *M. tuberculosis* genome. Further, we explored the structural association of these potential targets with FDA-approved drugs using molecular docking to shortlist promising lead molecules to aid drug development against TB.

Developing a new drug through conventional methods takes at least ten years of comprehensive research and huge funding; Nevertheless, the inclusion of computer-aided analyses at the initial stages can decrease the associated time and costs [11,12]. In silico drug screening serves as a valuable method to shortlist/prioritize only the most relevant compounds that could be checked later through experimental studies, and hence remove biomolecules that do not meet the required specifications to optimize the overall research in drug discovery [13]. Instead of focusing our attention on one drug target, we intended to scrutinize all high-ranked drug targets obtained computationally. Subject to experimental validation, this research work will provide a sound basis for developing novel therapeutics against the most troublesome bacterial pathogen *M. tuberculosis*.

## 2. Results and Discussion

### 2.1. Pangenome and Pan-Phylogeny Analysis of Mycobacterium tuberculosis Genomes

**Figure 1:**
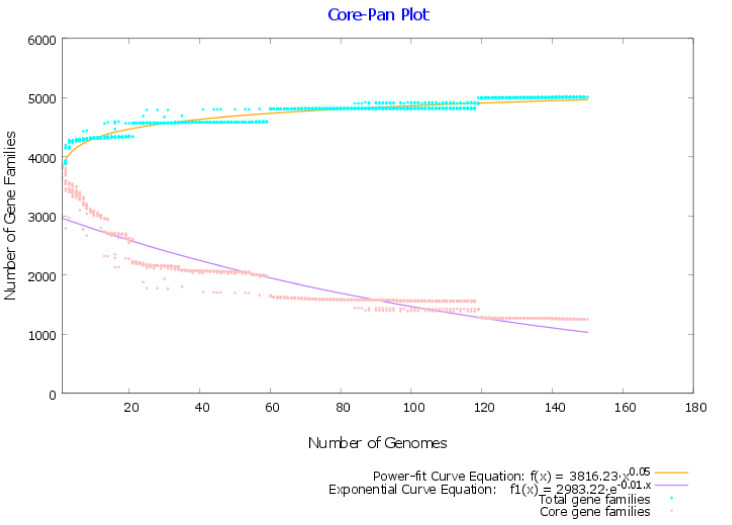
Pan-core plot of 150 *Mycobacterium tuberculosis* genomes. X-axis shows the number of genomes while the y-axis represents the number of gene families. With the addition of every genome, the pangenome size increased while the core genome size declined. The pangenome curve (shown in brown) has almost flattened, i.e., reached plateau. This suggests that the global gene repertoire of this species is unlikely to change significantly in the future and the pangenome is almost closed.

A total of 150 complete genomes and associated proteomes of *M. tuberculosis* were downloaded from the NCBI. Their information such as the accession number, strain name, and genome statistics are provided in Supplementary Table S1. The metadata associated with these bacterial strains such as the isolation source and the country of isolation is provided in Supplementary Table S2. Pangenome analysis of 150 *M. tuberculosis* complete genomes revealed that there were 5009 gene families in total (pangenome), among them, 1251 proteins were common (core genome) in all the studied genomes. The ratio of the core and pangenome size was found to be 0.25, thus the core forms 25% of the pangenome. This signifies the low level of genetic diversity in the *M. tuberculosis* strains. This trend is also visible in the pan-core genome plot, which projects that the global gene repertoire of this species is difficult to alter considerably in the future (Figure 1).

**Figure 2:**
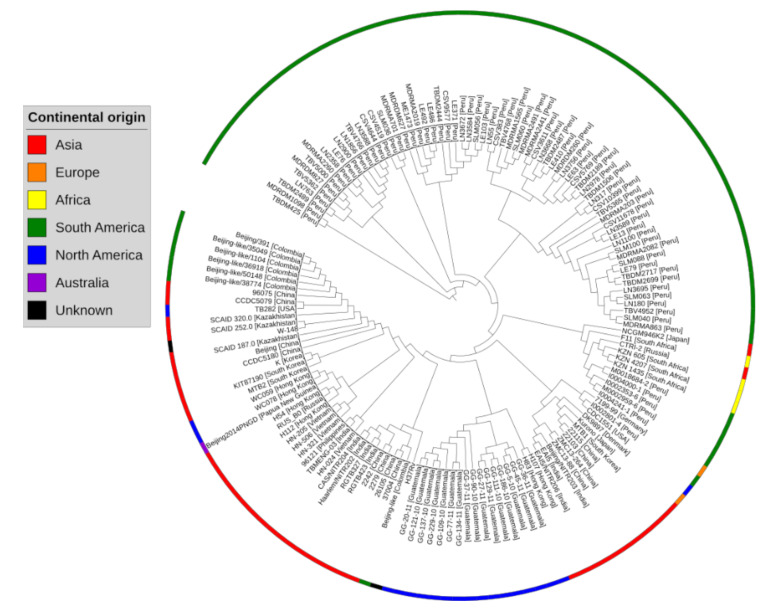
Pan-phylogeny tree of 150 complete genomes of *Mycobacterium tuberculosis*. Colored strips show the continental origin of strains. Overall, the strains originating from different geographical regions of the World clustered into different clades. This pan-phylogeny has been constructed based on accessory gene presence/absence data in different strains.

According to pangenome calculations, the b value of 0.0542 in the power-law regression model is indicative of a nearly close pangenome for *M. tuberculosis*. The pan-phylogeny based phylogenetic tree along with geographical source information is provided in Figure 2.

Each genome on average contained a total of 4102 protein-encoding genes. The core genome size (1251) thus accounted for 30.5% of the average genome size. Similarly, the minimum number of protein-encoding genes (3622) was present in strain RGTB423 while the maximum number of protein-encoding genes (4599) was present in strain 2279.

The level of conservation in the *M. tuberculosis* genomes is believed to be very high, with limited genetic variability between/across its various strains. Our genome-level analysis also indicates that the pangenome of this species is almost closed, with minimum variations. Multiple studies have corroborated this claim [14,15,16]. This is expected, as the lifestyle of this bacterium and its restricted niche makes it difficult to alter its gene pool [17]. Due to these unique conditions and their dependence on the host, it is suggested that there is less flexibility in its genome to accommodate more genes into the global gene repertoire. Hence, considering all this information now is the perfect opportunity to design and develop effective broad-spectrum therapeutics against this deadly human pathogen and to control the further spread of tuberculosis.

### 2.2. Subtractive Proteomics Revealed Putative Mycobacterium tuberculosis Drug Targets

Differential genome analysis was conducted on the core proteins of *M. tuberculosis* for the identification of therapeutic targets. Targets found to be human homologs could adversely affect the host metabolism, therefore, all those proteins were excluded in the first step that were identified as human homologs. The Rv numbers (gene identifiers or virulent strain of *Mycobacterium tuberculosis*) and the gene annotation were also conducted on these core proteins; the core genomes-associated data is provided in Supplementary Table S3. A total of 1185 proteins were identified as non-human homologous (Supplementary File S1) and were further screened based on their essentiality. Among them, 377 proteins were characterized as essential proteins and were considered crucial for pathogen survival (Supplementary File S2). If essential proteins are also functionally characterized as virulent, they are especially of vital significance to unveil novel therapeutic targets as these proteins help bacteria to modulate or degrade host defense mechanisms and may contribute to pathogenesis [18]. Therefore, the essential proteins were further screened to identify genes associated with pathogenicity. Among 377 essential proteins, VFDB and MvirDB identified a total of 93 virulence-related proteins involved in *M. tuberculosis* pathogenicity (Supplementary File S3). Since all the 93 proteins are non-human homologs and essential proteins associated with virulence, they represent an attractive dataset that could be explored for future vaccine production and drug design to tackle tuberculosis disease. However, for the purpose of this study, we further mined this large dataset to shortlist a few potential drug targets in order to facilitate the drug design and development against *M. tuberculosis*.

A total of 55 proteins were obtained after applying physiochemical checks, i.e., low molecular weights, a high value of the aliphatic index and negative GRAVY score (Supplementary File S4). Out of these, only eight proteins were identified by comparative pathway analysis to be involved in the unique bacterial metabolic pathway/s and were positively selected as *M. tuberculosis* drug targets to avoid targeting any human pathway upon drug therapy (Table 1).

Finally, a total of seven prioritized proteins were found to have similarity with targets associated with FDA-approved drugs and thus are likely druggable. These seven proteins, and their corresponding ligands, are tabulated in Table 2.

However, one prioritized target (two-component transcriptional regulatory protein DevR) did not show similarity to any drug target in the DrugBank database thus it is potentially a novel drug target worthy of experimental testing. Virtual screening may be performed with ligands from other ligand databases in order to find potent inhibitors of this protein. Literature studies were conducted on the seven proteins from Table 2 to understand their relevance as a drug target.

Among them, *trcR* has a crucial role in intracellular survival of *M. tuberculosis* and also in the regulation of Rv1057 expression, i.e., the β-propeller gene activated by the envelope stress [19]. In another study by Haydel et al.; they demonstrated that *trcR* and *trcS* two-component system genes are transcribed in broth-grown *M. tuberculosis* validated through reverse transcription PCR analysis. Moreover, through the SCOTS technique the expression of these genes are observed in liquid (broth) medium and after 18 h of *M. tuberculosis* growth in cultured human primary macrophages [20]. Nevertheless, the stimulatory signal for the TrcR-TrcS system is poorly understood and requires further research. Recently, another study, based on the drug target score, also ranked TrcR as the second-best therapeutic target in *M. tuberculosis* [21]. We believe that future studies are also needed to investigate this matter.

The dTDP-4-dehydrorhamnose reductase, another protein classified as a drug target by our study, is already known to be a high-confidence drug target [22]. This protein plays an important role in cell wall synthesis. Targeting the cell wall of *M. tuberculosis* through inhibitors is a good strategy, as has been recognized by earlier studies [23,24].

Similarly, *mtrA* (in Table 1) plays an important role in many crucial processes such as cell wall homeostasis and bacterial growth. It also has a role in cell division, DNA replication, and controls susceptibility of *M. tuberculosis* to the first line antimycobacterial drugs [25]. This signifies the necessity of *mtrA* for the growth of *M. tuberculosis*.

Another prioritized drug target RegX3 (in Table 1) has a role in aerobic respiration, virulence and phosphate absorption [26]. It is also required for optimal growth of *M. tuberculosis* in nutrient rich medium [27]. Moreover, RegX3 directly interacts with exs-5, another essential gene for the growth process. It also causes an increase in the production of membrane vesicles in *M. tuberculosis* [28].

The *kdpE* participates in the two-component system pathway and is involved in transcriptional regulation of Kdp-ATPase [29]. *KdpE* also has a role in the regulation of key factors involved in stress tolerance and energy metabolism [30,31]. Moreover, it is also notorious for its role in virulence caused by *M. tuberculosis*, *Pseudomonas aeruginosa*, *Salmonella enterica*, and *Staphylococcus aureus* [29,32,33,34,35]. Considering the situation, *kdpE* can be considered as a broad-spectrum therapeutic target for multiple pathogens.

The present study also prioritized drug target glucose-1-phosphate thymidylyltransferase. The protein is involved in three metabolic pathways namely, polyketide sugar unit biosynthesis, streptomycin biosynthesis, and the nucleotide sugars metabolism pathway [36,37,38]. All these pathways are crucial for survival of *M. tuberculosis* and elaborate on the relevance of glucose-1-phosphate thymidylyltransferase as a drug target. Additionally, the dTDP-4-dehydrorhamnose 3,5-epimerase protein obtained by our study is known to be a potential drug target in the rhamnose pathway [39].

### 2.3. Docking Analyses of Drug Targets Revealed Potential Lead Compounds for Drug Discovery

**Figure 3:**
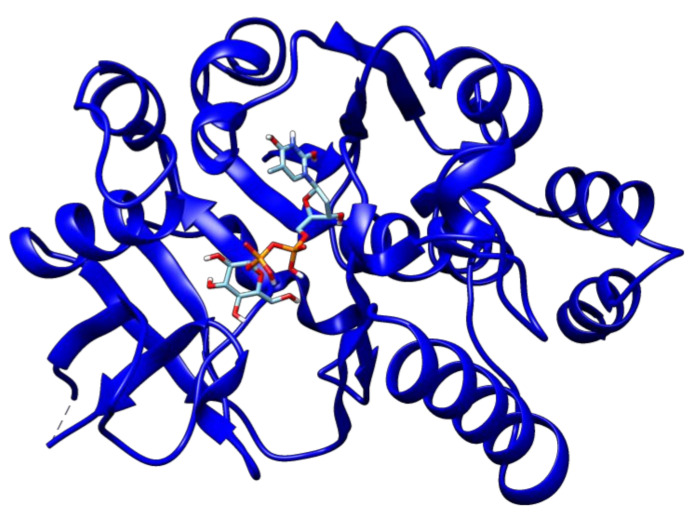
The docked complex of 2′deoxy-thymidine-5′-diphospho-alpha-d-glucose and glucose-1-phosphate thymidylyltransferase.

The Autodock vina docking scores of ligands with drug targets are elaborated in Table 2. Among all the drug–ligand interaction analyses, 2′deoxy-thymidine-5′-diphospho-alpha-d-glucose was found to have the lowest binding energy (−10.1 kcal/mol) with glucose-1-phosphate thymidylyltransferase (Figure 3).

Interestingly, 2′deoxy-thymidine-5′-diphospho-alpha-d-glucose was found to associate with another prioritized drug target protein of this study DNA response regulator, however, the binding energy was somewhat higher (−7.2 kcal/mol) in that case. Nevertheless, our docking analyses proposed the 2′deoxy-thymidine-5′-diphospho-alpha-d-glucose compound as a high-ranked lead compound to develop a drug against tuberculosis.

**Figure 4:**
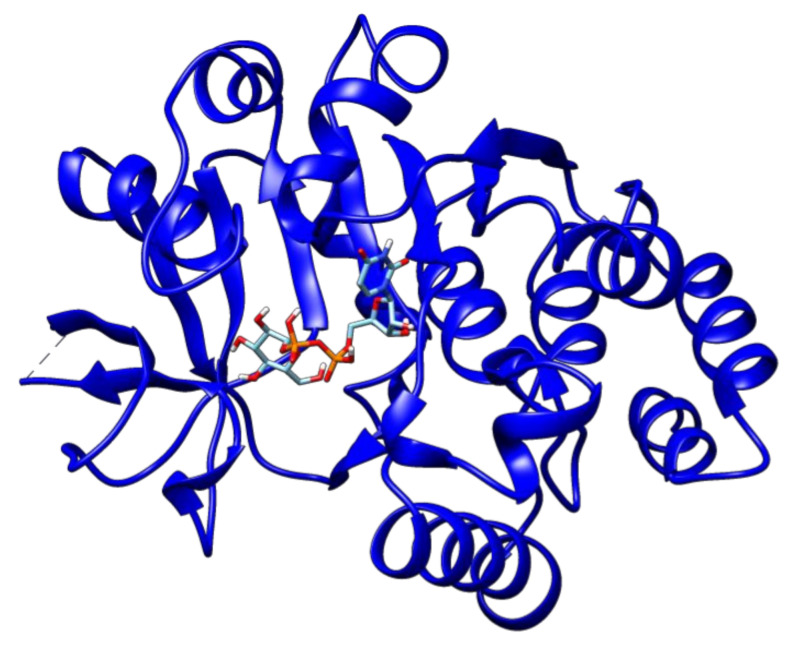
The docked complex of uridine diphosphate glucose and glucose-1-phosphate thymidylyltransferase.

**Figure 5:**
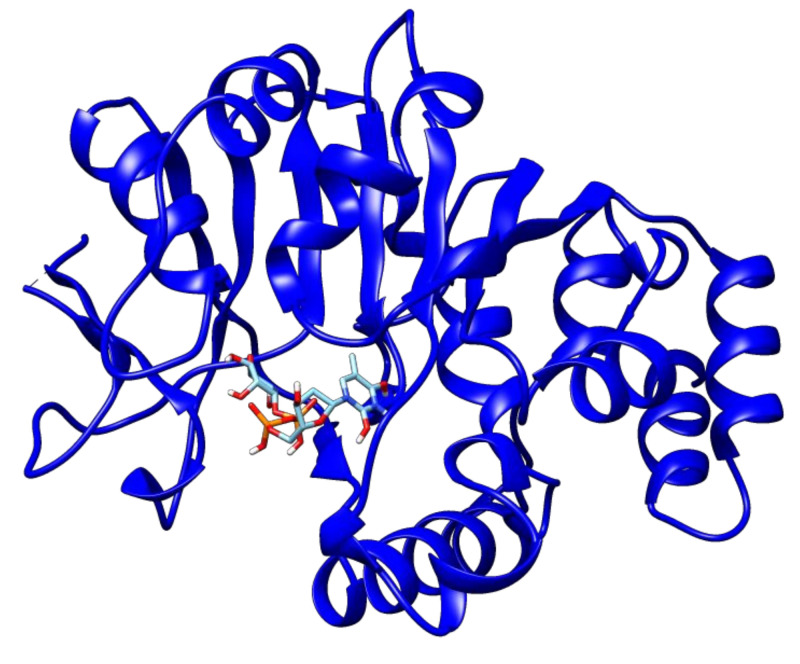
The docked complex of 2′-deoxy-thymidine-beta-l-rhamnose and glucose-1-phosphate thymidylyltransferase.

The second high-ranked ligand in our study is uridine diphosphate glucose. This compound showed a binding energy of −9.8 kcal/mol with glucose-1-phosphate thymidylyltransferase (Figure 4), and thus may also represent a good target for lead optimization. Meanwhile, 2′-deoxy-thymidine-beta-l-rhamnose ligand exhibited docked energy of −9.1 kcal/mol with glucose-1-phosphate thymidylyltransferase (Figure 5).

**Figure 6:**
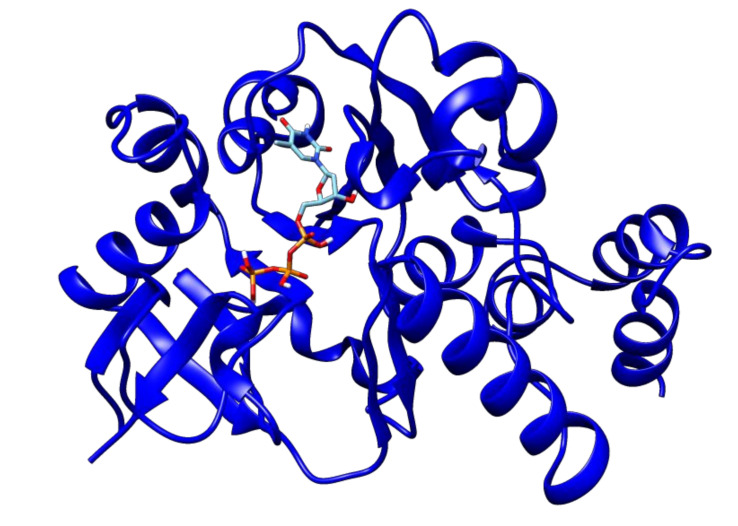
The docked complex of thymidine-5′-triphosphate and glucose-1-phosphate thymidylyltransferase.

**Figure 7:**
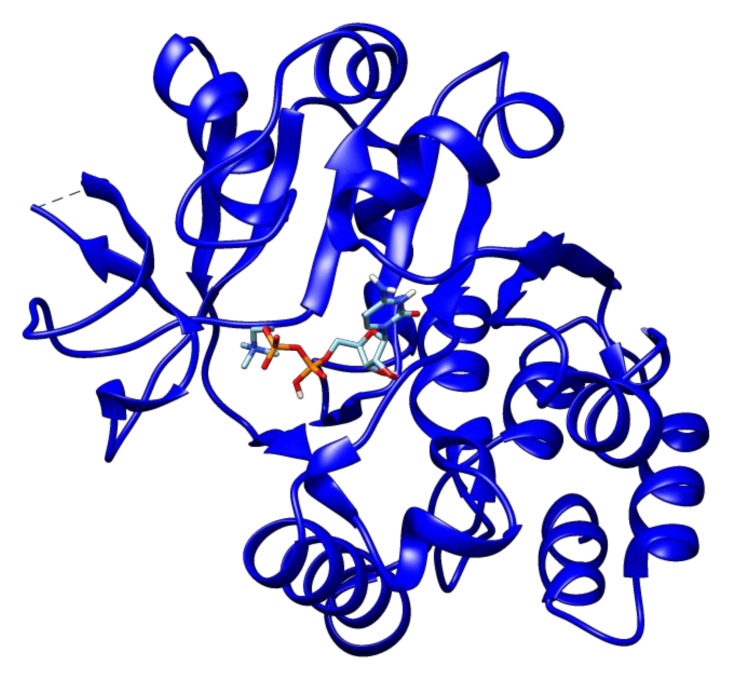
The docked complex of citicoline and glucose-1-phosphate thymidylyltransferase.

This ligand also interacted with another prioritized protein of our study dTDP-4-dehydrorhamnose reductase with the binding energy of −8.3 kcal/mol. Another high-ranked ligand thymidine-5’-triphosphate ligand was found to have −8.9 kcal/mol binding energy with glucose-1-phosphate thymidylyltransferase (Figure 6) while citicoline, on the other hand, showed −8.4 kcal/mol binding energy with this same drug target (Figure 7).

Thus, overall, this study shortlisted five top-ranked ligands with good binding affinity to drug targets and thus may guide drug development studies to counter *M. tuberculosis*. These ligands are 2′deoxy-thymidine-5′-diphospho-alpha-d-glucose, uridine diphosphate glucose, 2′-deoxy-thymidine-beta-l-rhamnose, thymidine-5′-triphosphate, and citicoline, which have good binding affinity with metabolic pathway proteins especially glucose-1-phosphate thymidylyltransferase. The protein is involved in three metabolic pathways namely, polyketide sugar unit biosynthesis, streptomycin biosynthesis, and nucleotide sugars metabolism pathway [36,37,38]. All these pathways are crucial for survival of *M. tuberculosis* and shortlisted repurposed drugs have higher affinity with the enzyme, i.e., glucose-1-phosphate thymidylyltransferase. Moreover, these drugs have a tendency to bind with dTDP-4-dehydrorhamnose 3,5-epimerase that is a potential drug target in rhamnose pathway [39]. These findings may provide a basis for the development of effective therapeutics against tuberculosis and/or replace currently available drugs to treat this deadly pathogen, however, experimental validation is needed to confirm these results.

## 3. Materials and Methods

**Figure 8:**
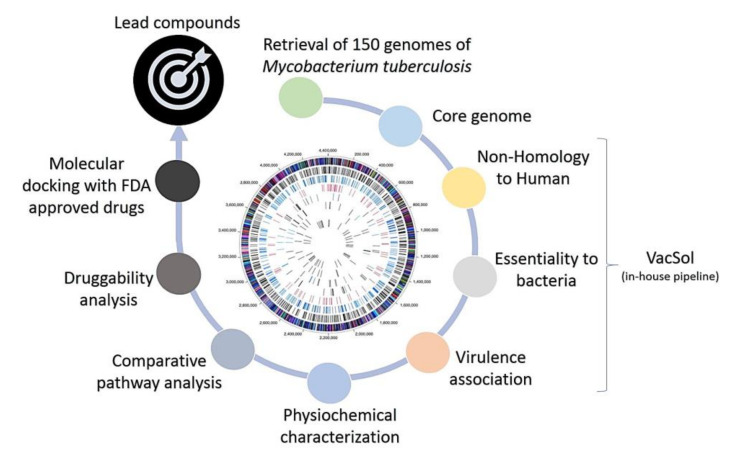
The methodology adopted in the study to find lead compounds (drugs) against *Mycobacterium tuberculosis*.

The methodology adopted in this study was visualized in Figure 8. The different steps were elaborated below.

A total of 150 complete genome sequences of *Mycobacterium tuberculosis* were retrieved from the NCBI GenBank database and pangenome analysis was conducted to identify the genes shared by all the strains of our dataset (i.e., the core genome). The core genome was then subjected to various sequential filters of subtractive genomics (non-homology to humans, bacterial essentiality and virulence, physiochemical checks, comparative pathway analysis, and druggability analysis) to select potential drug targets. Finally, these proteins were subjected to molecular docking with ligands from the DrugBank database to identify potential lead compounds that could be useful to develop a drug against tuberculosis.

### 3.1. Collection of Genomic Data

The complete genome sequences off 150 *M. tuberculosis* strains and their associated proteomes were retrieved from the GenBank database (https://www.ncbi.nlm.nih.gov/genbank/) available at the National Center for Biotechnology Information (NCBI). These strains had been earlier isolated from all the geographical regions of the World.

### 3.2. Pangenome Analysis of Mycobacterium tuberculosis Strains

Pangenome analysis was conducted on the genomes using the Bacterial Pan Genome Analysis (BPGA) tool to obtain highly conserved proteins of *M. tuberculosis* [40]. For this, we used the default threshold of 50% identity to generate orthologous protein clusters by the USEARCH algorithm [41]. BPGA calculates pan and core genome/proteome size by randomly considering 20 permutations and sequentially stating median values after the addition of each genome. Graphically, the core and pan-genome curves are generated by comparing the total number of common and unique gene families against the total number of genomes, respectively. The output also shows the number of new genes with the addition of every genome. The pan phylogeny was generated using the pan-matrix data and the associated pangenome tree was constructed using the neighbor-joining method with a default combination value of 20 iterations. Protein sequences associated with the core genome were retrieved and subjected to further screening steps to identify potential therapeutic targets.

### 3.3. Identification of Non-Host Homologous, Essential, and Virulence-Associated Proteins

To aid the preliminary identification of novel therapeutic targets in the core genome/proteome, we used an in-house pipeline VacSol for non-homology, essentiality, and virulence screening steps [42]. To avoid harmful responses in humans due to drug therapy, the drug targets must be non-homologous to human proteins, so for the identification of non-host homologous targets, the NCBI BLASTp tool (E-value 1 × 10^−3^) was used to compare the core genome with the human genome [43]. From this non-host homologous conserved proteome, essential genes were identified using BLASTp against the Database of Essential Genes (DEG) at an E-value <0.0001 and bit score >100 [44]. The DEG comprises experimentally validated data collected from archaea, bacteria, and eukaryotes, including currently reported essential genomic elements such as genes that are required for cellular life. We subjected the selected proteins to BLASTp search against the Virulence Factor Database (VFDB) and the Microbial Virulence database (MvirDB) to identify potential virulence-associated factors [45,46]. The VFDB is a comprehensive online database that contains information about virulence factors of bacterial pathogens. Virulence factors are known gene products that enable a microorganism to establish itself within a host and thus enhance its disease-inducing potential [47]. MvirDB is another integrated online database comprising publicly available, organized sequences related to known virulence factors, toxins, and antibiotic resistance genes [46].

### 3.4. Identification of Putative Mycobacterium tuberculosis Drug Targets

To identify potential drug targets, the non-human homolog, essential, and virulence-associated core proteins of *M. tuberculosis* were further filtered based on physicochemical parameters such as molecular weight, pI (isoelectric point), grand average of hydropathicity (GRAVY) value, and aliphatic index using the ProtParam tool [48]. Low molecular weight proteins are considered good drug targets, as they are accessible to drugs [49]. A higher value of the aliphatic index of proteins indicates thermostability whereas the negative GRAVY (grand average of hydropathicity) value indicates the hydrophilic nature of putative drug targets [50]. After physicochemical filters, comparative pathway analysis of filtered proteins was performed using the KEGG Automatic Annotation Server (KAAS) version 2.1 to identify the proteins associated with pathogen-specific pathways to enable specific targeting of the pathogen [51].

Finally, druggability assessment of the shortlisted proteins was performed using BLASTp against the DrugBank database at default settings [52]. The druggability of potential targets is also a crucial screening step that evaluates the potential of prioritized targets to be modulated by a drug or drug-like entity [50]. The potential targets should bind to a drug or drug-like compound with high affinity. However, this was not considered an inclusion criterion as novel drug targets may not display homology to targets associated with FDA approved drugs and show specific affinity to other drugs from natural products [53].

### 3.5. Molecular Docking of Putative Drug Targets with Drugs

The sequences of drug targets were checked for the availability of crystal structures, if any, from the RCSB Protein DataBank [54]. Structural file PDB was downloaded, and all the attached ligands were removed from the crystal structure. The drug targets having no associated crystal structure available in the database were modeled using the i-TASSER server [55,56]. Structure with the highest C-Score was selected in each case as these models are of good quality and stability. Using the PyRx-incorporated Autodock vina tool, the structures of drug targets were subjected to molecular docking with the FDA-approved drugs identified earlier in the druggability analysis [57,58]. Blind docking was conducted by adjusting the grid-box in order to cover the whole protein space, and the default value of exhaustiveness (8) was selected. Both ligands and their targets were prepared in the PDBQT format as per requirements and molecular docking analysis was performed. Docked complexes with the lowest binding energies were comparatively analyzed to identify those ligands that associated strongly with drug targets. These ligands were thus shortlisted as lead compounds for developing a potent drug against tuberculosis.

## Figures and Tables

**Table 1:** Metabolic pathway analysis of potential drug targets.

Protein Name	Rv Locus Number	KEGG Orthology	Metabolic Pathway(s)
dTDP-4-dehydrorhamnose reductase	Rv3266c	K00067	ko00521 Streptomycin biosynthesis ko00523 Polyketide sugar unit biosynthesis ko00541 *O*-Antigen nucleotide sugar biosynthesis ko01100 Metabolic pathways ko01110 Biosynthesis of secondary metabolites
glucose-1-phosphate thymidylyltransferase	Rv0334	K00973	ko00521 Streptomycin biosynthesis ko00523 Polyketide sugar unit biosynthesis ko00525 Acarbose and validamycin biosynthesis ko00541 O-Antigen nucleotide sugar biosynthesis ko01100 Metabolic pathways ko01110 Biosynthesis of secondary metabolites
two-component system regulator trcR	Rv1033c	K07672	ko02020 Two-component system
two-component system regulator mtrA	Rv3246c	K07670	ko02020 Two-component system
two-component system regulator regX3	Rv0491	K07776	ko02020 Two-component system
two-component system regulator kdpE	Rv1027c	K07667	ko02020 Two-component system ko02024 Quorum sensing
two-component system regulator devR	Rv3133c	K07695	ko02020 Two-component system
dTDP-4-dehydrorhamnose 3,5-epimerase	Rv3465	K01790	ko00521 Streptomycin biosynthesis ko00523 Polyketide sugar unit biosynthesis ko00541 O-Antigen nucleotide sugar biosynthesis ko01100 Metabolic pathways ko01110 Biosynthesis of secondary metabolites

**Table 2:** The Autodock vina score of all drug targets with their ligands from the DrugBank database. The binding energies are indicated by a bold black color in the case of top interactions.

Target	Rv Locus Number	Ligand	Binding Energy
Glucose-1-phosphate thymidylyltransferase	Rv0334	2′-Deoxy-Thymidine-Beta-l-Rhamnose	−9.1
		2′deoxy-Thymidine-5′-Diphospho-Alpha-d-Glucose	−10.1
		Alpha-d-Glucose-1-Phosphate	−5.8
		Citicoline	−8.4
		Citric acid	−5.7
		Thymidine-5′-Triphosphate	−8.9
		Thymidine	−7.1
		Thymidine monophosphate	−7.7
		Uridine diphosphate glucose	−9.8
DNA-binding response regulator	Rv1027c	Phosphoaspartate	−5.2
		Guanosine-5′-Monophosphate	−7.9
		AlphaBeta-Methyleneadenosine-5′-Triphosphate	−7.3
		Adenosine-5′-Rp-Alpha-Thio-Triphosphate	−6.8
		2-Hydroxyestradiol	−7.9
dTDP-4-dehydrorhamnose 3,5-epimerase	Rv3465	2′deoxy-Thymidine-5′-Diphospho-Alpha-d-Glucose	−7.2
		3′-*O*-Acetylthymidine-5′-Diphosphate	−7.1
		d-tartaric acid	−4.6
		SS-(2-Hydroxyethyl)Thiocysteine	−4.6
		Thymidine_monophosphate	−6.8
		Thymidine-5′-diphospho-beta-d-xylose	−6.7
DNA-binding response regulator TrcR	Rv1033c	S-Methyl Phosphocysteine	−4.6
		Phosphoaspartate	−4.8
		Guanosine-5′-Monophosphate	−7.2
		Glycerine	−4
		AlphaBeta-Methyleneadenosine-5′-Triphosphate	−7.4
		Adenosine-5′-Rp-Alpha-Thio-Triphosphate	−7.6
		3-Aminosuccinimide	−4.5
		2-Hydroxyestradiol	−7.1
DNA-binding response regulator RegX3	Rv0491	2-Hydroxyestradiol	−7.1
		3-Aminosuccinimide	−4.4
		Adenosine-5′-Rp-Alpha-Thio-Triphosphate	−6.7
		AlphaBeta-Methyleneadenosine-5′-Triphosphate	−6.6
		Glycerine	−3.8

## Supplementary Materials

The following are available online at https://www.mdpi.com/2079-6382/9/11/819/s1, File S1: The core proteins of 150 *Mycobacterium tuberculosis* genomes found to be non-homologous to the human genome given in the FASTA format. File S2: The essential and non-human homolog core proteins identified in genomes under study given in the FASTA format. File S3: Projected virulence-associated proteins from the non-homologous essential core proteins given in the FASTA format. File S4: Protein targets found to have appropriate physiochemical parameters provided. Table S1: Information about 150 complete genome sequences of *Mycobacterium tuberculosis* such as strain name, biosample, bioproject, and genome statistics are provided. Table S2: The metadata associated with these bacterial strains such as isolation source and the country of isolation is provided. Table S3: The annotation of core gene targets and their Rv numbers.
