# Supplementary material for: Pangenome Analysis of Mycobacterium tuberculosis Reveals Core-Drug Targets and Screening of Promising Lead Compounds for Drug Discovery
Source: Antibiotics (Basel). 2020 Nov 17;9(11):819. doi: 10.3390/antibiotics9110819 (PMC7698547; doi:10.3390/antibiotics9110819)
Supplement: Supplementary file 1 [file antibiotics-09-00819-s001.zip › antibiotics-984987-supplementary/File S1.docx]

**File S1.** The core proteins of 150 *M. tuberculosis* genomes found to be non-homologous to the human genome given in FASTA format below:

>1|CORE_REP|Org55_Gene4085#

MSLMVAPELVAAAAADLTGIGQAISAANAAAAGPTTQVLAAAGDEVSAAIAALFGTHAQEYQALSARVATFHEQFVRSLTAAGSAYATAEAANASPLQALEQQVLGAINAPTQLWLGRPLIGDGVHGAPGTGQPGGAGGLLWGNGGNGGSGAAGQVGGPGGAAGLFGNGGSGGSGGAGAAGGVGGSGGWLNGNGGAGGAGGTGANGGAGGNAWLFGAGGSGGAGTNGGVGGSGGFVYGNGGAGGIGGIGGIGGNGGDAGLFGNGGAGGAGAAGLPGAAGLNGGDGSDGGNGGTGGNGGRGGLLVGNGGAGGAGGVGGDGGKGGAGDPSFAVNNGAGGNGGHGGNPGVGGAGGAGGLLAGAHGAAGATPTSGGNGGDGGIGATANSPLQAGGAGGNGGHGGLVGNGGTGGAGGAGHAGSTGATGTALQPTGGNGTNGGAGGHGGNGGNGGAQHGDGGVGGKGGAGGSGGAGGNGFDAATLGSPGADGGMGGNGGKGGDGGGGGAGGTAVAGTAGKAGDGGAGGDGGKAGDGGAGAAGDVTLAVNQGAGGDGGNGGEVGVGGKGGAGGVSANPALNGSAGANGTAPTSGGNGGNGGAGATPTVAGENGGAGGNGGHGGSVGNGGAGGAGGNGVAGTGLALNGGNGGNGGNGGIGGNGGSAAGTGGDGGKGGNGGAGANGQDFSASANGANGGQGGNGGNGGIGGKGGDAFATFAKAGNGGAGGNGGAAGNGGGGAAGDVTLAINQGAGGAGGNGGNVGVAGQGGAGGKGAIPAMKGATGADGTAPTSGGDGGNGGNGASPTVAGGNGGDGGKGGSGGNVGNGGNGGAGGNGAAGQAGTPGPTSGDSGTSGTDGGAGGNGGAGGAGGTLAGHGGNGGKGGNGGQGGIGGAGERGADGAGPNANGANGENGGSGGNGGDGGAGGNGGAGGKAQAAGYTDGATGTGGDGGNGGDGGKAGDGGAGENGLNSGAMLPGGGTVGNPGTGGNGGNGGNAGVGGTGGKAGTGSLTGLDGTDGITPNGGNGGNGGNGGKGGTAGNGSGAAGGNGGNGGSGLNGGDAGNGGNGGGALNQAGFFGTGGKGGNGGNGGAGMINGGLGGFGGAGGGGAVDVAATTGGAGGNGGAGGFASTGLGGPGGAGGPGGAGDFASGVGGVGGAGGDGGAGGVGGFGGQGGIGGEGRTGGNGGSGGDGGGGISLGGNGGLGGNGGVSETGFGGAGGNGGYGGPGGPEGNGGLGGNGGAGGNGGVSTTGGDGGAGGKGGNGGDGGNVGLGGDAGSGGAGGNGGIGTDAGGAGGAGGAGGNGGSSKSTTTGNAGSGGAGGNGGTGLNGAGGAGGAGGNAGVAGVSFGNAVGGDGGNGGNGGHGGDGTTGGAGGKGGNGSSGAASGSGVVNVTAGHGGNGGNGGNGGNGSAGAGGQGGAGGSAGNGGHGGGATGGDGGNGGNGGNSGNSTGVAGLAGGAAGAGGNGGGTSSAAGHGGSGGNGGSGGSGGSGGSGTTGGAGAAGGNGGAGAGGGSLSTGQSGGHGGSGGAGGNGGAGSAGNGGAGGAGGNGGAGGNGGGGDAGNAGSGGNGGKGGDGVGPGSTGGAGGKGGAGANGGSSNGNARGGNAGNGGHGGAGGSGDTGGAGGAGGQGGFGGTGGSGSGIGGGAGGNGGNGGAGGTGVVLGGKGGDGGNGDHGGPATNPGSGSRGGAGGSGGNGGAGGNATGSGGKGGAGGNGGDGSFGATSGPASIGVTGAPGGNGGKGGAGGSNPNGSGGDGGNGGNGGAGGNGGSIGANSGIVGGSGGAGGAGGAGGNGSLSSGEGGKGGDGGHGGDGVGGNSSVTQGGSGFFGGKGGFGGDGGQGGPNGGGTVGTVAGGGGNGGVGGRGGDGVFAGAGGQGGLGGQGGNGGGSTGGNGGLGGAGGGGGNAPDGGFGGNGGKGGQGGIGGGTQSATGLGGDGGDGGDGGNGGNSGAKAGGAGGKGQAGQPNSGTEPGFGGDGGLGGAGATP

>2|CORE_REP|Org57_Gene4069#

MRRPNPGVRCGIVVNVTGPPPTIDRRYHDAVIVGLDNVVDKATRVHAAAWTKFLDDYLTRRPQRTGEDHCPLTHDDYRRFLAGKPDGVADFLAARGIRLPPGSPTDLTDDTVYGLQNLERQTFLQLLNTGVPEGKSIASFARRLQVAGVRVAAHTSHRNYGHTLDATGLAEVFAVFVDGAVTAELGLPAEPNPAGLIETAKRLGANPGRCVVIDSCQTGLRAGRNGGFALVIAVDAHGDAENLLSSGADAVVADLAAVTVGSGDAAISTIPDALQVYSQLKRLLTGRRPAVFLDFDGTLSDIVERPEAATLVDGAAEALRALAAQCPVAVISGRDLADVRNRVKVDGLWLAGSHGFELVAPDGSHHQNAAATAAIDGLAEAAAQLADALREIAGAVVEHKRFAVAVHYRNVADDSVDNLIAAVRRLGHAAGLRVTTGRKVVELRPDIAWDKGKALDWIGERLGPAEVGPDLRLPIYIGDDLTDEDAFDAVRFTGVGIVVRHNEHGDRRSAATFRLECPYTVCQFLSQLACDLQEAVQHDDPWTLVFHGYDPGQERLREALCAVGNGYLGSRGCGPESAESEAHYPGTYVAGVYNQLTDHIEGCTVDNESLVNLPNWLSLTFRIDGGAWFNVDTVELLSYRQTFDLRRATLTRSLRFRDAGGRVTTMTQERFASMNRPNLVALQTRIESENWSGTVDFRSLVDGGVHNTLVDRYRQLSSQHLTTAEIEVLADSVLLRTQTSQSGIAIAVAARSTLWRDGQRVDAQYRVARDTNRGGHDIQVTLSAGQSVTLEKVATIFTSRDAATLTAAISAQRCLGEAGRYAELCQQHVRAWARLWERCAIDLTGNTEELRLVRLHLLHLLQTISPHTAELDAGVPARGLNGEAYRGHVFWDALFVAPVLSLRMPKVARSLLDYRYRRLPAARRAAHRAGHLGAMYPWQSGSDGSEVSQQLHLNPRSGRWTPDPSDRAHHVGLAVAYNAWHYYQVTGDRQYLVDCGAELLVEIARFWVGLAKLDDSRGRYLIRGVIGPDEFHSGYPGNEYDGIDNNAYTNVMAVWVILRAMEALDLLPLTDRRHLIEKLGLTTQERDQWDDVSRRMFVPFHDGVISQFEGYSELAELDWDHYRHRYGNIQRLDRILEAEGDSVNNYQASKQADALMLLYLLSSDELIGLLARLGYRFAPTQIPGTVDYYLARTSDGSTLSAVVHAWVLARANRSNAMEYFRQVLRSDIADVQGGTTQEGIHLAAMAGSIDLLQRCYSGLELRDDRLVLSPQWPEALGPLEFPFVYRRHQLSLRISGRSATLTAESGDAEPIEVEWSQLRSNAVATCSGYGAGTPSKSVAAGDQCRTWWVDDLSWKGRPAAVSLIALGVVCSSPATKGTPPG

>3|CORE_REP|Org58_Gene4039#

MNFSVLPPEINSALIFAGAGPEPMAAAATAWDGLAMELASAAASFGSVTSGLVGGAWQGASSSAMAAAAAPYAAWLAAAAVQAEQTAAQAAAMIAEFEAVKTAVVQPMLVAANRADLVSLVMSNLFGQNAPAIAAIEATYEQMWAADVSAMSAYHAGASAIASALSPFSKPLQNLAGLPAWLASGAPAAAMTAAAGIPALAGGPTAINLGIANVGGGNVGNANNGLANIGNANLGNYNFGSGNFGNSNIGSASLGNNNIGFGNLGSNNVGVGNLGNLNTGFANTGLGNFGFGNTGNNNIGIGLTGNNQIGIGGLNSGTGNFGLFNSGSGNVGFFNSGNGNFGIGNSGNFNTGGWNSGHGNTGFFNAGSFNTGMLDVGNANTGSLNTGSYNMGDFNPGSSNTGTFNTGNANTGFLNAGNINTGVFNIGHMNNGLFNTGDMNNGVFYRGVGQGSLQFSITTPDLTLPPLQIPGISVPAFSLPAITLPSLTIPAATTPANITVGAFSLPGLTLPSLTIPAATTPANITVGAFSLPGLTLPSLNIPAATTPANITVGAFSLPGLTLPSLNIPAATTPANITVGAFSLPGLTLPSLNIPAATTPANITVGAFSLPGLTLPSLNIPAATTPANITVGAFSLPGLTLPSLNIPAATTPANITVGAFSLPGLTLPSLNIPAATTPANITVGAFSLPGLTLPSLNIPAATTPANITVGAFSLPGLTLPSLNIPAATTPANITVGAFSLPGLTLPSLNIPAATTPANITVSGFQLPPLSIPSVAIPPVTVPPITVGAFNLPPLQIPEVTIPQLTIPAGITIGGFSLPAIHTQPITVGQIGVGQFGLPSIGWDVFLSTPRITVPAFGIPFTLQFQTNVPALQPPGGGLSTFTNGALIFGEFDLPQLVVHPYTLTGPIVIGSFFLPAFNIPGIDVPAINVDGFTLPQITTPAITTPEFAIPPIGVGGFTLPQITTQEIITPELTINSIGVGGFTLPQITTPPITTPPLTIDPINLTGFTLPQITTPPITTPPLTIDPINLTGFTLPQITTPPITTPPLTIDPINLTGFTLPQITTPPITTPPLTIDPINLTGFTLPQITTPPITTPPLTIDPINLTGFTLPQITTPPITTPPLTIEPIGVGGFTTPPLTVPGIHLPSTTIGAFAIPGGPGYFNSSTAPSSGFFNSGAGGNSGFGNNGSGLSGWFNTNPAGLLGGSGYQNFGGLSSGFSNLGSGVSGFANRGILPFSVASVVSGFANIGTNLAGFFQGTTS

>4|CORE_REP|Org117_Gene2968#

MCDVLMQPVRTPRPSTNLRSKPLRPTGDGGVFPRLGRLIVRRPWVVIAFWVALAGLLAPTVPSLDAISQRHPVAILPSDAPVLVSTRQMTAAFREAGLQSVAVVVLSDAKGLGAADERSYKELVDALRRDTRDVVMLQDFVTTPPLRELMTSKDNQAWILPVGLPGDLGSTQSKQAYARVADIVEHQVAGSTLTANLTGPAATVADLNLTGQRDRSRIEFAITILLLVILLIIYGNPITMVLPLITIGMSVVVAQRLVAIAGLAGLGIANQSIIFMSGMMVGAGTDYAVFLISRYHDYLRQGADSDQAVKKALTSIGKVIAASAATVAITFLGMVFTQLGILKTVGPMLGISVAVVFFAAVTLLPALMVLTGRRGWIAPRRDLTRRFWRSSGVHIVRRPKTHLLASALVLVILAGCAGLARYNYDDRKTLPASVESSIGYAALDKHFPSNLIIPEYLFIQSSTDLRTPKALADLEQMVQRVSQVPGVAMVRGITRPAGRSLEQARTSWQAGEVGSKLDEGSKQIAVHTGDIDKLAGGANLMASKLGDVRAQVNRAISTVGGLIDALAYLQDLLGGNRVLGELEGAEKLIGSMRALGDTIDADASFVANNTEWASPVLGALDSSPMCTADPACASARTELQRLVTARDDGTLAKISELARQLQATRAVQTLAATVSGLRGALATVIRAMGSLGMSSPGGVRSKINLVNKGVNDLADGSRQLAEGVQLLVDQVKKMGFGLGEASAFLLAMKDTATTPAMAGFYIPPELLSYATGESVKAETMPSEYRDLLGGLNVDQLKKVAAAFISPDGHSIRYLIQTDLNPFSTAAMDQIDAITAAARGAQPNTALADAKVSVVGLPVVLKDTRDYSDHDLRLIIAMTVCIVLLILIVLLRAIVAPLYLIGSVIVSYLAALGIGVIVFQFLLGQEMHWSIPGLTFVILVAVGADYNMLLISRLREEAVLGVRSGVIRTVASTGGVITAAGLIMAASMYGLVFASLGSVVQGAFVLGTGLLLDTFLVRTVTVPAIAVLVGQANWWLPSSWRPATWWPLGRRRGRAQRTKRKPLLPKEEEEQSPPDDDDLIGLWLHDGLRL

>5|CORE_REP|Org2_Gene2756#

MGSEHPVDGMTRRQFFAKAAAATTAGAFMSLAGPIIEKAYGAGPCPGHLTDIEHIVLLMQENRSFDHYFGTLSDTRGFDDTTPPVVFAQSGWNPMTQAVDPAGVTLPYRFDTTRGPLVAGECVNDPDHSWIGMHNSWNGGANDNWLPAQVPFSPLQGNVPVTMGFYTRRDLPIHYLLADTFTVCDGYFCSLLGGTTPNRLYWMSAWIDPDGTDGGPVLIEPNIQPLQHYSWRIMPENLEDAGVSWKVYQNKLLGALNNTVVGYNGLVNDFKQAADPRSNLARFGISPTYPLDFAADVRNNRLPKVSWVLPGFLLSEHPAFPVNVGAVAIVDALRILLSNPAVWEKTALIVNYDENGGFFDHVVPPTPPPGTPGEFVTVPDIDSVPGSGGIRGPIGLGFRVPCLVISPYSRGPLMVHDTFDHTSTLKLIRARFGVPVPNLTAWRDATVGDMTSTFNFAAPPNPSKPNLDHPRLNALPKLPQCVPNAVLGTVTKTAIPYRVPFPQSMPTQETAPTRGFPAVSVDPQSAMAATVSRNTRGRADVVSQGAFAGMSRRAFLAKAAGAGAAAVLTDWAAPVIEKAYGAGPCSGHLTDIEHIVLCLQENRSFDHYFGTLSAVDGFDTPTPLFQQKGWNPETQALDPTGITLPYRINTTGGPNGVGECVNDPDHQWIAAHLSWNGGANDGWLPAQARTRSVANTPVVMGYYARPDIPIHYLLADTFTICDQYFSSLLGGTMPNRLYWISATVNPDGDQGGPQIVEPAIQPKLTFTWRIMPQNLSDAGISWKVYNSKLLGGLNDTSLSRNGYVGSFKQAADPRSDLARYGIAPAYPWDFIRDVINNTLPQVSWVVPLTVESEHPSFPVAVGAVTIVNLIRVLLRNPAVWEKTALIIAYDEHGGFFDHVTPLTAPEGTPCEWIPNSVDIDKVDGSGGIRGPIGLGFRVPCFVISPYSRGGLMVHDRFDHTSQLQLIGKRFGVPVPNLTPWRASVTGDMTSAFNFAAPPDPSPPNLDHPVRQLPKVAKCVPNVVLGFLNEGLPYRVPYPQTTPVQESGPARPIPSGIC

>7|CORE_REP|Org26_Gene3910#

MGMRSAARMPKLTRRSRILIMIALGVIVLLLAGPRLIDAYVDWLWFGELGYRSVFTTMLATRIVVCLVAGVVVGGIVFGGLALAYRTRPVFVPDADNDPVARYRAVVLARLRLVGIGIPAAIGLLAGIVAQSYWARIQLFLHGGDFGVRDPQFGRDLGFYAFELPFYRLMLSYMLVSVFLAFVANLVAHYIFGGIRLSGRTGALSRSARVQLVSLVGVLVLLKAVAYWLDRYELLSHTRGGKPFTGAGYTDINAVLPAKLILMAIALICAAAVFSAIALRDLRIPAIGLVLLLLSSLIVGAGWPLIVEQISVKPNAAQKESEYISRSITATRQAYGLTSDVVTYRNYSGDSPATAQQVAADRATTSNIRLLDPTIVSPAFTQFQQGKNFYYFPDQLSIDRYLDRNGNLRDYVVAARELNPDRLIDNQRDWINRHTVYTHGNGFIASPANTVRGIANDPNQNGGYPEFLVNVVGANGTVVSDGPAPLDQPRIYFGPVISNTSADYAIVGRNGDDREYDYETNIDTKRYTYTGSGGVPLGGWLARSVFAAKFAERNFLFSNVIGSNSKILFNRDPAQRVEAVAPWLTTDSAVYPAIVNKRLVWIVDGYTTLDNYPYSELTSLSSATADSNEVAFNRLVPDKKVSYIRNSVKATVDAYDGTVTLYQQDEKDPVLKVWMQVFPGTVKPKSDIAPELAEHLRYPEDLFKVQRMLLAKYHVNDPVTFFSTSDFWDVPLDPNPTASSYQPPYYIVAKNIAKDDNSASYQLISAMNRFKRDYLAAYISASSDPATYGNLTVLTIPGQVNGPKLANNAITTDPAVSQDLGVIGRDNQNRIRWGNLLTLPVARGGLLYVEPVYASPGASDAASSYPRLIRVAMMYNDKVGYGPTVRDALTGLFGPGAGATATGIAPTEAAVPPSPAANPPPPASGPQPPPVTAAPPVPVGAVTLSPAKVAALQEIQAAIGAARDAQKKGDFAAYGSALQRLDEAITKFNDAG

>8|CORE_REP|Org1_Gene2938#

MQRFGTGSSRSWCGRAGTATIAAVLLASGALTGLPPAYAISPPTIDPGALPPDGPPGPLAPMKQNAYCTEVGVLPGTDFQLQPKYMEMLNLNEAWQFGRGDGVKVAVIDTGVTPHPRLPRLIPGGDYVMAGGDGLSDCDAHGTLVASMIAAVPANGAVPLPSVPRRPVTIPTTETPPPPQTVTLSPVPPQTVTVIPAPPPEEGVPPGAPVPGPEPPPAPGPQPPAVDRGGGTVTVPSYSGGRKIAPIDNPRNPHPSAPSPALGPPPDAFSGIAPGVEIISIRQSSQAFGLKDPYTGDEDPQTAQKIDNVETMARAIVHAANMGASVINISDVMCMSARNVIDQRALGAAVHYAAVDKDAVIVAAAGDGSKKDCKQNPIFDPLQPDDPRAWNAVTTVVTPSWFHDYVLTVGAVDANGQPLSKMSIAGPWVSISAPGTDVVGLSPRDDGLINAIDGPDNSLLVPAGTSFSAAIVSGVAALVRAKFPELSAYQIINRLIHTARPPARGVDNQVGYGVVDPVAALTWDVPKGPAEPPKQLSAPLVVPQPPAPAIWCRYGWPPGDWPGTIDRRCGVRYRDLDAAITEAAMKAQRSFGLALSWPRVTAVFLVDVLILAVASHCPDSWQADHHVAWWVGVGVAAVVTLLSVVSYHGITVISGLATWVRDWSADPGTTLGAGCTPAIDHQRRFGRDTVGVREYNGRLVSVIEVTCGESGPSGRHWHRKSPVPMLPVVAVADGLRQFDIHLDGIDIVSVLVRGGVDAAKASASLQEWEPQGWKSEERAGDRTVADRRRTWLVLRMNPQRNVAAVACRDSLASTLVAATERLVQDLDGQSCAARPVTADELTEVDSAVLADLEPTWSRPGWRHLKHFNGYATSFWVTPSDITSETLDELCLPDSPEVGTTVVTVRLTTRVGSPALSAWVRYHSDTRLPKEVAAGLNRLTGRQLAAVRASLPAPTHRPLLVIPSRNLRDHDELVLPVGQELEHATSSFVGQ

>9|CORE_REP|Org10_Gene4001#

MSGTPDDGDIGLIIAVKRLAAAKTRLAPVFSAQTRENVVLAMLVDTLTAAAGVGSLRSITVITPDEAAAAAAAGLGADVLADPTPEDDPDPLNTAITAAERVVAEGASNIVVLQGDLPALQTQELAEAISAARHHRRSFVADRLGTGTAVLCAFGTALHPRFGPDSSARHRRSGAVELTGAWPGLRCDVDTPADLTAARQLGGRARDRASGRTSLTGTGQRRRGIQGGERQTNGERMPAECWQPHPMMSNDRKVTEIENSPVTEVRPEEHAWYPDDSALAAPPAATPAAISDQLPSDRYLNRELSWLDFNARVLALAADKSMPLLERAKFLAIFASNLDEFYMVRVAGLKRRDEMGLSVRSADGLTPREQLGRIGEQTQQLASRHARVFLDSVLPALGEEGIYIVTWADLDQAERDRLSTYFNEQVFPVLTPLAVDPAHPFPFVSGLSLNLAVTVRQPEDGTQHFARVKVPDNVDRFVELAAREASEEAAGTEGRTALRFLPMEELIAAFLPVLFPGMEIVEHHAFRITRNADFEVEEDRDEDLLQALERELARRRFGSPVRLEIADDMTESMLELLLRELDVHPGDVIEVPGLLDLSSLWQIYAVDRPTLKDRTFVPATHPAFAERETPKSIFATLREGDVLVHHPYDSFSTSVQRFIEQAAADPNVLAIKQTLYRTSGDSPIVRALIDAAEAGKQVVALVEIKARFDEQANIAWARALEQAGVHVAYGLVGLKTHCKTALVVRREGPTIRRYCHVGTGNYNSKTARLYEDVGLLTAAPDIGADLTDLFNSLTGYSRKLSYRNLLVAPHGIRAGIIDRVEREVAAHRAEGAHNGKGRIRLKMNALVDEQVIDALYRASRAGVRIEVVVRGICALRPGAQGISENIIVRSILGRFLEHSRILHFRAIDEFWIGSADMMHRNLDRRVEVMAQVKNPRLTAQLDELFESALDPCTRCWELGPDGQWTASPQEGHSVRDHQESLMERHRSP

>10|CORE_REP|Org118_Gene2197#

MVPGEVHMSDTPSGPHPIIPRTIRLAAIPILLCWLGFTVFVSVAVPPLEAIGETRAVAVAPDDAQSMRAMRRAGKVFNEFDSNSIAMVVLESDQPLGEKAHRYYDHLVDTLVLDQSHIQHIQDFWRDPLTAAGAVSADGKAAYVQLYLAGNMGEALANESVEAVRKIVANSTPPEGIRTYVTGPAALFADQIAAGDRSMKLITGLTFAVITVLLLLVYRSIATTLLILPMVFIGLGATRGTIAFLGYHGMVGLSTFVVNILTALAIAAGTDYAIFLVGRYQEARHIGQNREASFYTMYRGTANVILGSGLTSIAGATYCLSFARLTLFHTMGPPLAIGMLVSVAAALTLAPAIIAIAGRFGLLDPKRRLKTRGWRRVGTAVVRWPGPILATSVALALVGLLALPGYRPGYNDRYYLRAGTPVNRGYAAADRHFGPARMNPEMLLVESDQDMRNPAGMLVIDKIAKEVLHVSGVERVQAITRPQGVPLEHASIPFQISMMGATQTMSLPYMRERMADMLTMSDEMLVAINSMEQMLDLVQQLNDVTHEMAATTREIKATTSELRDHLADIDDFVRPLRSYFYWEHHCFDIPLCSATRSLFDTLDGVDTLTDQLRALTDDMNKMEALTPQFLALLPPMITTMKTMRTMMLTMRSTISGVQDQMADMQDHATAMGQAFDTAKSGDSFYLPPEAFDNAEFQQGMKLFLSPNGKAVRFVISHESDPASTEGIDRIEAIRAATKDAIKATPLQGAKIYIGGTAATYQDIRDGTKYDILIVGIAAVCLVFIVMLMITQSLIASLVIVGTVLLSLGTAFGLSVLIWQHFVGLQVHWTIVAMSVIVLLAVGSDYNLLLVSRFKEEGAGAGLKTGIIRAMAGTGASCHVGRSGIRVHHGVHGRQRTPRYRTGRHHHRARSTFRYPGGPIVHDAIHRSAARSLVLVAEHDPLETHRPGGAHTPGRSPNSAASAPGLICTSVP

>11|CORE_REP|Org105_Gene3458#

MTESPTAGPGGVPRADDADSDVPRYRYTAELAARLERTWQENWARLGTFNVPNPVGSLAPPDGAAVPDDKLFVQDMFPYPSGEGLHVGHPLGYIATDVYARYFRMVGRNVLHALGFDAFGLPAEQYAVQTGTHPRTRTEANVVNFRRQLGRLGFGHDSRRSFSTTDVDFYRWTQWIFLQIYNAWFDTTANKARPISELVAEFESGARCLDGGRDWAKLTAGERADVIDEYRLVYRADSLVNWCPGLGTVLANEEVTADGRSDRGNFPVFRKRLRQWMMRITAYADRLLDDLDVLDWPEQVKTMQRNWIGRSTGAVALFSARAASDDGFEVDIEVFTTRPDTLFGATYLVLAPEHDLVDELVAASWPAGVNPLWTYGGGTPGEAIAAYRRAIAAKSDLERQESREKTGVFLGSYAINPANGEPVPIFIADYVLAGYGTGAIMAVPGHDQRDWDFARAFGLPIVEVIAGGNISESAYTGDGILVNSDYLNGMSVPAAKRAIVDRLESAGRGRARIEFKLRDWLFARQRYWGEPFPIVYDSDGRPHALDEAALPVELPDVPDYSPVLFDPDDADSEPSPPLAKATEWVHVDLDLGDGLKPYSRDTNVMPQWAGSSWYELRYTDPHNSERFCAKENEAYWMGPRPAEHGPDDPGGVDLYVGGAEHAVLHLLYSRFWHKVLYDLGHVSSREPYRRLVNQGYIQAYAYTDARGSYVPAEQVIERGDRFVYPGPDGEVEVFQEFGKIGKSLKNSVSPDEICDAYGADTLRVYEMSMGPLEASRPWATKDVVGAYRFLQRVWRLVVDEHTGETRVADGVELDIDTLRALHRTIVGVSEDFAALRNNTATAKLIEYTNHLTKKHRDAVPRAAVEPLVQMLAPLAPHIAEELWLRLGNTTSLAHGPFPKADAAYLVDETVEYPVQVNGKVRGRVVVAADTDEETLKAAVLTDEKVQAFLAGATPRKVIVVAGRLVNLVI

>12|CORE_REP|Org122_Gene4011#

MRSQRLAGHLSAAARTIHALSLPIILFWVALTIVVNVVAPQLQSVARTHSVALGPHDAPSLIAMKRIGKDFQQFDSDTTAMVLLEGQEKLGDEAHRFYDVLVTKLSQDTTHVQHIENFWGDPLTAAGSQSADGKAAYVQLNLTGDQGGSQANESVAAVQRIVDSVPPPPGIKAYVTGPGPLGADRVVYGDRSLHTITGISIAVIAIMLFIAYRSLSAALIMLLTVGLELLAVRGIISTFAVNDLMGLSTFTVNVLVALTIAASTDYIIFLVGRYQEARATGQNREAAYYTMFGGTAHVVLASGLTVAGAMYCLGFTRLPYFNTLASPYFNTLASPCAIGLVTVMLASLTLAPAIIAVASRFGLFDPKRATTKRRWRRIGTVVVRWPGPVLAATLLIALIGLLALPKYQTNYNERYYIPSAAPSNIGYLASDRHFPQARMEPEVLMVEADHDLRNPTDMLILDRIAKTVFHTPGIARVQSITRPLGAPIDHSSIPFQLGMQSTMTIENLQNLKDRVADLSTLTDQLQRMIDITQRTQELTRQLTDATHDMNAHTRQMRDNANELRDRIADFDDFWRPLRSFTYWERHCFDIPICWSMRSLLNSMDNVDKLTEDLANLTDDTERMDTTQRQLLAQLDPTIATMQTVKDLAQTLTSAFSGLVTQMEDMTRNATVMGRTFDAANNDDSFYLPPEAFQNPDFQRGLKLFLSPDGTCARFVITHRGDPASAEGISHIDPIMQAADEAVKGTPLQAASIYLAGTSSTYKDIHEGTLYDVMIAVVASLCLIFIIMLGITRSVVASAVIVGTVALSLGSAFGLSVLIWQHILHMPLHWLVLPMAIIVMLAVGSDYNLLLIARFQEEIGAGLKTGMIRAMAGTGRVVTIAGLVFAFTMGSMVASDLRVVGQIGTTIMIGLLFDTLVVRSYMTPALATLLGRWFWWPRRVDRLARQPQVLGPRRTTALSAERAALLQ

>13|CORE_REP|Org1_Gene3904#

MASLPDGALMRRAAFGLATEIGRELTARTGGVVGRRVCAVVGSGDNGGDALWAATFLRRRGAAADAVLLNPDRTHRKALAAFTKSGGRLVESVSAATDLVIDGVVGISGSGPLRPAAAQVFAAVQAAAIPVVAVDIPSGIDVATGAITGPAVHAALTVTFGGLKPVHALADCGRVVLVDIGLDLAHTDVLGFEATDVAARWPVPGPRDDKYTQGVTGVLAGSSTYPGAAVLCTGAAVAATSGMVRYAGTAHAEVLAHWPEVIASPTPAAAGRVQAWVVGPGLGTDEAGAAALWFALDTDLPVLVDADGLTMLADHPDLVAGRNAPTVLTPHAGEFARLAGAPPGDDRVGACRQLADALGATVLLKGNVTVIADPGGPVYLNPAGQSWAATAGSGDVLSGMIGALLASGLPSGEAAAAAAFVHARAAAAAAADPGPAMRPRRRRASAATFGPLWLPCSPASPQQARKGSAVSRSHPSVPAHSIAPAYTGRMFTAPVPALRMPDESMDPEAAYRFIHDELMLDGSSRLNLATFVTTWMDPEAEKLMAETFDKNMIDKDEYPATAAIEARCVSMVADLFHAEGLRDHDPTSATGVSTIGSSEAVMLGGLALKWRWRQRVGSWKGRMPNLVMGSNVQVVWEKFCRYFDVEPRYLPMERGRYVITPEQVLAAVDENTIGVVAILGTTYTGELEPIAEICAALDKLAAGGGVDVPVHVDAASGGFVVPFLHPDLVWDFRLPRVVSINVSGHKYGLTYPGVGFVVWRGPEHLPEDLVFRVNYLGGDMPTFTLNFSRPGNQVVGQYYNFLRLGRDGYTKVMQALSHTARWLGDQLREVDHCEVISDGSAIPVVSFRLAGDRGYTEFDVSHELRTFGWQVPAYTMPDNATDVAVLRIVVREGLSADLARALHDDAVTALAALDKVKPGGHFDAQHFAH

>14|CORE_REP|Org20_Gene1712#

MIVPAREPEPQPRRVLNGLSDVRAFFHNNTVPLYFISPTPFNLLGIYRWIRNFFYLTYYDSFEGEHSRVFVPRRRDRRDFDGMGDVCNHLLRDPETLEFIKNRGPGGKACFVMLDEETQALARQAGLEVMHPPAELRHRLESKIVMTRLADEAGVPSVPHVIGRVSSYDELSALAHGAGLGDDLVVEAAYGNAGSATFFVRGLRDWDQCAGGIVGQPEIKVMKRIRNVEVCIEATVTRHGTVIGPAMTSLVGYPELTPYRGAWCGNDVWRGALPPAQTRAAREMVAKLGDVLSREGYRGYFEVDLLHDLDADELYLGEVNPRLSGASPMTNLTTEAYADMPLFLFHLLEYMDVDYELDIEAINSRWERGYGEDEVWGQLIMSETSPDLELFTATPRTGMWRLNHDGRVSFARQGNDWATMLDESEAFYMRVAAPGDLRCEGAQLGVLAPAAPADRRLPAHRARPALDRRPQGAVRLDAADARRPDRLAARRTGVTALRPVSHWTTGCRRRIRIGHSSTSKTSCRPRSRGGTEPVVTLPADNAPIADIGLTSTDGIATTVGAVMAATATDGWAVAHRGALVAEQYLDGLGPRTRHLLFSVSKSLVAAVVGALHGAGAIELDAPVTAYVPALADCGYAGATVRHLLDMRSGVAFSENYDDPAAEIHVREQVIGWAPKRGPDLPATAARLPADLRRKSAHGGPFEYRSCETDVLGWICEAAAGQPMPELMSELLWSRIGAQCDATIALDVAGAAGTGIFDGGISACLTDMIRFGSLYLRDGVSLAGQQVVPAAWIADTFDGGPDSRQAFAASPDDNPMPGGMYRNQVWFPYPGSNVALCVGMCGQLIYVNRAAEVVAAKLSTQPHSHEPHMLDTLRAFDAVAHELSGIRSSSTNDPQRPSPPAQEASPG

>15|CORE_REP|Org2_Gene773#

MPQPRTHLPIPSAARTGLITYDAKDPDSTYPPIEQLRPPAGAPNVLLILLDDVGFGASSAFGGPCRTSTAELLAGNGLRYNRFHTTALCSPTRQALLTGRNHHSAGMGGITEIATGAPGYSSVLPNTMSPIARTLKLNGYNTAQFGKCHEVPVWQTSPVGPFDAWPSGGGGFEYFYGFIGGEANQWYPSLYEGTTPVEVNRTPEEGYHFMADMTDKALGWIGQQKALAPDRPFFVYFAPGATHAPHHVPREWADKYRGRFDVGWDALREETFARQKELGVIPADCQLTARHAEIPAWDDMPEDLKPVLCRQMEVYAGFLEYTDHHVGRLVDGLQSLGVLDDTLVFYIIGDNGASAEGTINGTYNEMLNFNGLADIETPRFMTDRLDKFGGPESYNHYSVGWAHAMDTPYQWTKQVASHWGGTRNGTIVHWPNGIAAKGEMRWQFHHVIDVAPTILEAAGLPEPLFVNGVQQHPIEGVSMAYSFDDAQAPDRHETQYFEMFGNRGIYHKGWTAVTKHKTPWILVGEQTVAFDDDVWELYDTTKDWSQAKDLAKEMPEKLHELQRLWLIEATRYNVLPLDDDTASRINPDLAGRPVLIRGNTQVLFSNMGRLSENCVLNLKNKSHTVTAEVEVPETGAEGVIVAQGASIGGWSLYANDGKLKYCYNLGGIKHFYAESADPLPAGAHQVRMEFAYAGGGLGKGGEVTLYVDGQQVGEGHVEATLAIVFSADDGCDVGMDSGSPVSPDYARGVTRSTGGSRACSSRSPRPPLLRAIWSTRSTRSASRWRANRAAQSNGEGTAMEKSRCHAVAHGGGCAGSAKSHKSGGRCGQGRGAGDSHGTRGAGRRYRAASAPHPLAVGAHLRDELAKRSADPRLTDELNDLAGHTLDDL

>16|CORE_REP|Org20_Gene3642#

MVLRVHDVTGASELTLGNTVDWEFAASVGERLARPAPPSTEYTRRQVIDELTVAAEKAEPPVRDVTGLIADGVVPPARVVDRPAWIRSAAESMRAMTHGSAKPRGFLTGRITGAQTGAVLAFVASGILGQYDPFGAAGEGCLLLVYPNVIAVERQLRVEPSDFRLWVCLHEVTHRVQFTANPWLSGYMSQALNLLTFEPVDDIGRVVSRLADFIRSRGHGTDDSEVNPSGILGLVRAVQSEPQRKALDQLLVLGTLLEGHAEHVMDAVGPMVVPSVATIRRRFDDRRHHKQPPLQRLVRALLGFDAKLSQYTRGKAFVDHVVDRAGMKLFNTIWYATTCRCLPRSKTRSDGSTECCSAAACGCGAVRSGPSRRLRPLERGALRRPGLVGAHRIAARLWPTTALIVDHGLQPGSATVAETARIQAISLGCVDARVLCVQVGAAGGREAAARSARYSALEEHRDGPVLLAHTLDDQAETVLLGLGRGSGARSIAGMRPYDPPWCRPLLGVRRSVTHAACRELGLTAWQDPHNTDRRFTRTRLRTEVLPLLEDVLGGGVAEALARTATALREDTDLIDTIAAQALPGAAVAGSRGQELSTSALTALPDAVRRRVIRGWLLAGGATGLTDRQIRGVDRLVTAWRGQGGVAVGSSCAARWSPGGATAYLCCGASPFDARVGRWSRGVARCARDPELLGDHPRADGGALSGGHQVGAAHGRADSGPHRRARRADRQRLPRAVRYHRPGSAADHRAEGRGALRHRPGASDSRADPVRVHGGEFVWVIDILVGRGADPQGPRPRHPRPRRADRRGRRRLRPYAFVVVAEPDEPESAVIAGVHAAAQARCGARQRRNRVRGFRHSQRLRRGLRPGLRRTLP

>18|CORE_REP|Org116_Gene3997#

MSFVLAMPEVLGSAATDLAALGSVLGAADAAAAATTTGIVAAAQDEVSAAIAALFSAHGRAYQVASAQAAAVHAQFVEALSAGAGAYASAEAAGAAVLANPAQSVQQDLLAAVNAQSVALTGRPLIGNGANGAPGTGANGAPGGWLLGNGGAGGSAAAGSGLPGGAGGAAGLFGTGGAGGAGGSSTVGDGGAGGAGGSGGWLLGTGGVGGVGGLGAGAGGAGGVGGAGGLLGAGGHGGAGGLGAVTGGVGGAGGAGGLLAGLVGAGGGNGGAGGIGAGGVGGAGGAGGNAGLLAGPGGAGGTGGRGFLNDGGVGGAGGNAGLLFGAGGTGGSGGAGLGGDGGAGGAGGNAGVLFGNAGSGGTGGFGDTDGGAGGAGGDAGWLGSGGVGGAGGFGETGDGGVGGAGGKAGLLIGNGGAGGAGGQGAVTGGTGGAGGDGVLIGNGGNAGIGGTGPTAGDTGAGGISGLLLGADGFNAPASASPLHTLKQQALAAINAPTQTLTGRPLIGNGTPGAVGSGATGAPGGWLLGDGGAGGSGAAGSGAPGGAGGAAGLWGTGGAGGAGGSSAGGGGAGGAGGAGGWLLGDGGAGGIGGASTVLGGTGGGGGVGGLWGAGGAGGAGGTGLVGGDGGAGGAGGTGGLLAGLIGAGGGHGGTGGLSTNGDGGVGGAGGNAGMLAGPGGAGGAGGDGENLDTGGDGGAGGSAGLLFGSGGAGGAGGFGFLGGDGGAGGNAGLLLSSGGAGGFGGFGTAGGVGGAGGNAGWLGFGGAGGIGGIGGNANGGAGGNGGTGGQLWGSGGAGGEGGAALSVGDTGGAGGVGGSAGLIGTGGNGGNGGTGANAGSPGTGGAGGLLLGQNGLNGLP

>19|CORE_REP|Org118_Gene1392#

MTLTPEASKSVAQPPTQAPLTQEEAIASLGRYGYGWADSDVAGANAQRGLSEAVVRDISAKKNEPDWMLQSRLKALRIFDRKPIPKWGSNLDGIDFDNIKYFVRSTEKQAASWDDLPEDIRNTYDRLGIPEAEKQRLVAGVAAQYESEVVYHQIREDLEAQGVIFLDTDTGLREHPDIFKEYFGTVIPAGDNKFSALNTAVWSGGSFIYVPPGVHVDIPLQAYFRINTENMGQFERTLIIADEGSYVHYVEGCLPPGGELITTADGDLRPIESIRVGDFVTGHDGRPHRVTAVQVRDLDGELFTFTPMSPANAFSVTAEHPLLAIPRDEVRVMRKERNGWKAEVNSTKLRSAEPRWIAAKDVAEGDFLIYPKPKPIPHRTVLPLEFARLAGYYLAEGHACLTNGCESLIFSFHSDEFEYVEDVRQACKSLYEKSGSVLIEEHKHSARVTVYTKAGYAAMRDNVGIGSSNKKLSDLLMRQDETFLRELVDAYVNGDGNVTRRNGAVWKRVHTTSRLWAFQLQSILARLGHYATVELRRPGGPGVIMGRNVVRKDIYQVQWTEGGRGPKQARDCGDYFAVPIKKRAVREAHEPVYNLDVENPDSYLAYGFAVHNCTAPIYKSDSLHSAVVEIIVKPHARVRYTTIQNWSNNVYNLVTKRARAEAGATMEWIDGNIGSKVTMKYPAVWMTGEHAKGEVLSVAFAGEDQHQDTGAKMLHLAPNTSSNIVSKSVARGGGRTSYRGLVQVNKGAHGSRSSVKCDALLVDTVSRSDTYPYVDIREDDVTMGHEATVSKVSENQLFYLMSRGLTEDEAMAMVVRGFVEPIAKELPMEYALELNRLIELQMEGAVG

>20|CORE_REP|Org12_Gene3362#

MTKPAADASAVLTAEDTLVLASTATPVEMELIMGWLGQQRARHPDSKFDILKLPPRNAPPAALTALVEQLEPGFASSPQSGEDRSIVPVRVIWLPPADRSRAGKVAALLPGRDPYHPSQRQQRRILRTDPRRARVVAGESAKVSELRQQWRDTTVAEHKRDFAQFVSRRALLALARAEYRILGPQYKSPRLVKPEMLASARFRAGLDRIPGATVEDAGKMLDELSTGWSQVSVDLVSVLGRLASRGFDPEFDYDEYQVAAMRAALEAHPAVLLFSHRSYIDGVVVPVAMQDNRLPPVHMFGGINLSFGLMGPLMRRSGMIFIRRNIGNDPLYKYVLKEYVGYVVEKRFNLSWSIEGTRSRTGKMLPPKLGLMSYVADAYLDGRSDDILLQGVSICFDQLHEITEYAAYARGAEKTPEGLRWLYNFIKAQGERNFGKIYVRFPEAVSMRQYLGAPHGELTQDPAAKRLALQKMSFEVAWRILQATPVTATGLVSALLLTTRGTALTLDQLHHTLQDSLDYLERKQSPVSTSALRLRSREGVRAAADALSNGHPVTRVDSGREPVWYIAPDDEHAAAFYRNSVIHAFLETSIVELALAHAKHAEGDRVAAFWAQAMRLRDLLKFDFYFADSTAFRANIAQEMAWHQDWEDHLGVGGNEIDAMLYAKRPLMSDAMLRVFFEAYEIVADVLRDAPPDIGPEELTELALGLGRQFVAQGRVRSSEPVSTLLFATARQVAVDQELIAPAADLAERRVAFRRELRNILRDFDYVEQIARNQFVAREFKARQGRDRI

>21|CORE_REP|Org20_Gene1819#

MTDRVSVGNLRIARVLYDFVNNEALPGTDIDPDSFWAGVDKVVADLTPQNQALLNARDELQAQIDKWHRRRVIEPIDMDAYRQFLTEIGYLLPEPDDFTITTSGVDAEITTTAGPQLVVPVLNARFALNAANARWGSLYDALYGTDVIPETDGAEKGPTYNKVRGDKVIAYARKFLDDSVPLSSGSFGDATGFTVQDGQLVVALPDKSTGLANPGQFAGYTGAAESPTSVLLINHGLHIEILIDPESQVGTTDRAGVKDVILESAITTIMDFEDSVAAVDAADKVLGYRNWLGLNKGDLAAAVDKDGTAFLRVLNRDRNYTAPGGGQFTLPGRSLMFVRNVGHLMTNDAIVDTDGSEVFEGIMDALFTGLIAIHGLKASDVNGPLINSRTGSIYIVKPKMHGPAEVAFTCELFSRVEDVLGLPQNTMKIGIMDEERRTTVNLKACIKAAADRVVFINTGFLDRTGDEIHTSMEAGPMVRKGTMKSQPWILAYEDHNVDAGLAAGFSGRAQVGGHVDNAELMADMVETKIAQPRAGASTAWVPSPTAATLHALHYHQVDVAAVQQGLAGKRRATIEQLLTIPLAKELAWAPDEIREEVDNNCQSILGYVVRWVDQGVGCSKVPDIHDVALMEDRATLRISSQLLANWLRHGVITSADVRASLERMAPLVDRQNAGDVAYRPMAPNFDDSIAFLAAQELILSGAQQPNGYTEPSMPDVVGSLRPGPLRSRPHRTGPVTMRPASGPLWGHRPLAGESRPHRAEPVRSSCLTGAGLRRRGRRRRYG

>22|CORE_REP|Org51_Gene3937#

MAIAETDTEVHTPFEQDFEKDVAATQRYFDSSRFAGIIRLYTARQVVEQRGTIPVDHIVAREAAGAFYERLRELFAARKSITTFGPYSPGQAVSMKRMGIEAIYLGGWATSAKGSSTEDPGPDLASYPLSQVPDDAAVLVRALLTADRNQHYLRLQMSERQRAATPAYDFRPFIIADADTGHGGDPHVRNLIRRFVEVGVPGYHIEDQRPGTKKCGHQGGKVLVPSDEQIKRLNAARFQLDIMRVPGIIVARTDAEAANLIDSRADERDQPFLLGATKLDVPSYKSCFLAMVRRFYELGVKELNGHLLYALGDSEYAAAGGWLERQGIFGLVSDAVNAWREDGQQSIDGIFDQVESRFVAAWEDDAGLMTYGEAVADVLEFGQSEGEPIGMAPEEWRAFAARASLHAARAKAKELGADPPWDCELAKTPEGYYQIRGGIPYAIAKSLAAAPFADILWMETKTADLADARQFAEAIHAEFPEQMLAYNLSPSFNWDTTGMTDEEMRRFPEELGKMGFVFNFITYGGHQIDGVAAEEFATALRQDGMLALARLQRKMRLVESPYRTPQTLVGGPRSDAALAASSGRTATTKAMGKGSTQHQHLVQTEVPRKLLEEWLAMWSGHYQLKDKLRVQLRPQRAGSEVLELGIHGESDDKLANVIFQPIQDRRGRTILLVRDQNTFGAELRQKRLMTLIHLWLVHRFKAQAVHYVTPTDDNLYQTSKMKSHGIFTEVNQEVGEIIVAEVNHPRIAELLTPDRVALRKLITKEA

>23|CORE_REP|Org59_Gene1377#

MRWATVALLLFLAGLVAQLNGAPEAMWWTLYLACYLAGGWGSAWAGAQALRBKALDVDLLMIAAAVGAVAIGQIFDGALLIVIFATSGALDDIATRHTAESVKGLLDLAPDQAVVVQGDGSERVVAASELVVGDRVVVRPGDRIXADGAVLSGXXDVDQRSITGESMPVAKARGDEVFAGTVNGSGVLHLVVTRDPSQTVVARIVELVADASATKAKTQLFIEKIEQRYSLGMVAATLALIVIPLMFGADLRPVLLRAMTFMIVASPCAVVLATMPPLLSAIANAGRHGVLVKSAVVVXRLADTSIVALDKTGTLTRGIPRLASVAPLDPNVVDARRLLXXAXAAEQSSEHPLGRAIVAEARRRGIAIPPAKDFRAVPGCGVHALVGNDFVEIASPQSYRGAPLAXLAPLLSAGATAAIVLLDGVAIGVLGLTDQLRPDAVESVAAMAALTAAPPVLLTGDNGRAAWRVARNAGITDVRAALLPEQKVEVVRNLQAGGHQVLLVGDGVNDAPAMAAARAAVAMGAGADLTLQTADGVTIRDELHXIPTIIGLARQARRVVTVNLAIAATFIAVLVLWDLFGQLPLPLGVVGHEGSTVLVALNGMRLLTNRSWRXAXXXXXXGSXVAELTRAALGVPVTTRDLTAAYFQQTISANSNVLVYFWAPLCAPCDLFTPTYEASSRKHFDVVHGKVNIETEKDLASIAGVKLLPTLMAFKKGKLXLXXSRHRQSRDHGQSGATTPGIHLQVPGRRRYRPWNKDFILRR

>24|CORE_REP|Org59_Gene3550#

MEILVTGGAGFQGSHLTESLLANGHWVTVLDKSSRNAVRNMQGFRSHDRAAFISGSVTDGQTIDRAVRDHHVVFHLAAHVNVDQSLGDPESFLETNVMGTYRVLEAVRRYRNRLIYVSTCEVYGDGHNLKEGERLDEHAELKPNSPYGASKAAADRLCYSYFRSYGLDVTIVRPFNIFGVRQKAGRFGALIPRLVRQGINGEGLTIFGAGSATRDYLYVSDIVGAYNLVLRTPTLRGQAINFASGKDTRVRDIVEYVADKFGARIEHRDARPGEVQRFPADISLAKSIGXQPQVEIWDGIDRYINWAKDQPQYPYEQDGFSGSSVSLIXPXPPSSAGKVGRNGAELPAGRITSHSMVTVARRPVCPVTLTPGDPALASVXDLVDAWSAHDALAELVTMFGGAFPQTDHLEARLASLDKFSTAWDYRARARAARALHGEPVRCQDSGGGARWLIPRLDLPAKKRDAIVGLAQQLGXTLESTPQGTTFDHVLVIGTGRHSNLIRARWARELAKGRQVGHIVLAAASRRLLPSEDDAVAVCAPGARTEFELLAAAARDAFGLDVHPAVRYVRQRDDNPHRDSMVWRFAADTNDLGVPITLLEAPSPEPDSSRATSADTFTFTAHTLGMQDSTCLLVTGQPFVPYQNFDALRTLALPFGIQVETVGFGIDRYDGLGELDQQXPAKLLQEVRSTIRAARALLERMXGRRXHGYRSSAVMXCXXWXXGSCPIRLXTXPGGGHGRSPMLARYRTGLTCPSCVRTSKCR

>25|CORE_REP|Org45_Gene3055#

MSAEQPTIIYTLTDEAPLLATYAFLPIVRAFAEPAGIKIEASDISVAARILAEFPDYLTEEQRVPDNLAELGRLTQLPDTNIIKLPNISASVPQLVAAIKELQDKGYAVPDYPADPKTDQEKAIKERYARCLGSAVNPVLRQGNSDRRAPKAVKEYARKHPHSMGEWSMASRTHVAHMRHGDFYAGEKSMTLDRARNVRMELLAKSGKTIVLKPEVPLDDGDVIDSMFMSKKALCDFYEEQMQDAFETGVMFSLHVKATMMKVSHPIVFGHAVRIFYKDAFAKHQELFDDLGVNVNNGLSDLYSKIESLPASQRDEIIEDLHRCHEHRPELAMVDSARGISNFHSPSDVIVDASMPAMIRAGGKMYGADGKLKDTKAVNPESTFSRIYQEIINFCKTNGQFDPTTMGTVPNVGLMAQQAEEYGSHDKTFEIPEDGVANIVDVATGEVLLTENVEAGDIWRMCIVKDAPIRDWVKLAVTRARISGMPVLFWLDPYRPHENELIKKVKTYLKDHDTEGLDIQIMSQVRSMRYTCERLVRGLDTIAATGNILRDYLTDLFPILELGTSAKMLSVVPLMAGGGMYETGAGGSAPKHVKQLVEENHLRWDSLGEFLALGAGFEDIGIKTGNERAKLLGKTLDAAIGKLLDNDKSPSRKTGELDNRGSQFYLAMYWAQELAAQTDDQQLAEHFASLADVLTKNEDVIVRELTEVQGEPVDIGGYYAPDSDMTTAVMRPSKTFNAALEAVQG

>27|CORE_REP|Org67_Gene624#

MPDNTIQWDKDADGIVTLTMDDPSGSTNVMNEAYIESMGKAVDRLVAEKDSITGVVVASAKKTFFAGGDVKTMIQARPEDAGDVFNTVETIKRQLRTLETLGKPVVAAINGAALGGGLEIALACHHRIAADVKGSQLGLPEVTLGLLPGGGGVTRTVRMFGIQNAFVSVLAQGTRFKPAKAKEIGLVDELVATVEELVPAAKAWIKEELKANPDGAGVQPWDKKGYKMPGGTPSSPGLAAILPSFPSNLRKQLKGAPMPAPRAILAAAVEGAQVDFDTASRIESRYFASLVTGQVAKNMMQAFFFDLQAINAGGSRPEGIGKTPIKRIGVLGAGMMGAGIAYVSAKAGYEVVLKDVSLEAAAKGKGYSEKLEAKALERGRTTQERSDALLARITPTADAADFKGVDFVIEAVFENQELKHKVFGEIEDIVEPNAILGSNTSTLPITGLATGVKRQEDFIGIHFFSPVDKMPLVEIIKGEKTSDEALARVFDYTLAIGKTPIVVNDSRGFFTSRVIGTFVNEALAMLGEGVEPASIEQAGSQAGYPAPPLQLSDELNLELMHKIAVATRKGVEDAGGTYQPHPAEAVVEKMIELGRSGRLKGAGFYEYADGKRSGLWPGLRETFKSGSSQPPLQDMIDRMLFAEALETQKCLDEGVLTSTADANIGSIMGIGFPPWTGGSAQFIVGYSGPAGTGKAAFVARARELAAAYGDRFLPPESLLS

>28|CORE_REP|Org2_Gene7#

MGKNEARRSALAPDHGTVVCDPLRRLNRMHATPEESIRIVAAQKKKAQDEYGAASITILEGLEAVRKRPGMYIGSTGERGLHHLIWEVVDNAVDEAMAGYATTVNVVLLEDGGVEVADDGRGIPVATHASGIPTVDVVMTQLHAGGKFDSDAYAISGGLHGVGVSVVNALSTRLEVEIKRDGYEWSQVYEKSEPLGLKQGAPTKKTGSTVRFWADPAVFETTEYDFETVARRLQEMAFLNKGLTINLTDERVTQDEVVDEVVSDVAEAPKSASERAAESTAPHKVKSRTFHYPGGLVDFVKHINRTKNAIHSSIVDFSGKGTGHEVEIAMQWNAGYSESVHTFANTINTHEGGTHEEGFRSALTSVVNKYAKDRKLLKDKDPNLTGDDIREGLAAVISVKVSEPQFEGQTKTKLGNTEVKSFVQKVCNEQLTHWFEANPTDAKVVVNKAVSSAQARIAARKARELVRRKSATDIGGLPGKLADCRSTDPRKSELYVVEGDSAGGSAKSGRDSMFQAILPLRGKIINVEKARIDRVLKNTEVQAIITALGTGIHDEFDIGKLRYHKIVLMADADVDGQHISTLLLTLLFRFMRPLIENGHVFLAQPPLYKLKWQRSDPEFAYSDRERDGLLEAGLKAGKKINKEDGIQRYKGLGEMDAKELWETTMDPSVRVLRQVTLDDAAAADELFSILMGEDVDARRSFITRNAKDVRFLDV

>30|CORE_REP|Org59_Gene3354#

MVATVTDEQSAARELVRGWARTAASGAAATAAVRDMEYGFEEGNADAWRPVFAGLAGLGLFGVAVPEDCGGAGGSIEDLCAMVDEAARALVPGPVATTAVATLVVSDPKLRSALASGERFAGVAIDGGVQVDPKTSTASGTVGRVLGGAPGGVVLLPADGNWLLVDTACDEVVVEPLRATDFSLPLARMVLTSAPVTVLEVSGERVEDLAATVLAAEAAGVARWTLDTAVAYAKVREQFGKPIGSFQAVKHLCAQMLCRAEQADVAAADAARAAADSDGTQLSIAAAVAASIGIDAAKANAKDCIQVLGGIGCTWEHDAHLYLRRAHGIGGFLGGSGRWLRRVTALTQAGVRRRLGVDLAEVAGLRPEIAAAVAEVAALPEEKRQVALADTGLLAPHWPAPYGRGASPAEQLLIDQELAAAKVERPDLVIGWWAAPTILEHGTPEQIERFVPATMRGEFLWCQLFSEPGAGSDLASLRTKAVRAXRRXAXRSGQKVWTSAAHKARWGXCLARXDPDAPKHKGITYFLVDMTTPGIEIRPLREITGDSLFNEVFLDNVFVPDEMVVGAVNDGWRLARTXLANERVAMATGTALGNPMEELLKVLGDMELDVAQQDRLGRLILLAQAGALLDRRIAELAVGGQDPGAQSSVRKLIGVRYRQALAEYLMEVSDGGGLVENRAVYDFLNTRCLTIAGGTEQILLTVAAERLXGLPR

>32|CORE_REP|Org131_Gene298#

MADSSAIYLAAPESQTGKSTIALGLLHRLTAMVAKVGVFRPITRLSAERDYILELLLAHTSAGLPYERCVGVTYQQLHADRDDAIAEIVDSYHAMADECDAVVVVGSDYTDVTSPTELSVNARIAVNLGAPVLLTVRAKDRTPDQVASVVEVCLAELDTQRAHTAAVVANRCELSAIPAVTDALRRFTPPSYVVPEEPLLSAPTVAELTQAVNGAVVSGDVALREREVMGVLAAGMTADHVLERLTDGMAVITPGDRSDVVLAVASAHAAEGFPSLSCIVLNGGFQLHPAIAALVSGLRLRLPVIATALGTYDTASAAASARGLVTATSQRKIDTALELMDRHVDVAGLLAQLTIPIPTVTTPQMFTYRLLQQARSDLMRIVLPEGDDDRILKSAGRLLQRGIVDLTILGDEAKVRLRAAELGVDLDGATVIEPCASELHDQFADQYAQLRKAKGITVEHAREIMNDATYFGTMLVHNCHADGMVSGAAHTTAHTVRPALEIIKTVPGISTVSSIFLMCLPDRVLAYGDCAIIPNPTVEQLADIAICSARTAAQFGIEPRVAMLSYSTGDSGKGADVDKVRAATELVRAREPQLPVEGPIQYDAAVEPSVAATKLRDSPVAGRATVLIFPDLNTGNNTYKAVQRSAGAIAIGPVLQGLRKPVNDLSRGALVDDIVNTVAITAIQAQGVHE

>33|CORE_REP|Org2_Gene4458#

MGPLLPSASLKLNVLPVGVLPRRPVVGRVGRPVFPYEPMVRVSLWLSVTAVAVLFGWGSWQRRWIADDGLIVLRTVRNLLAGNGPVFNQGERVEANTSTAWTYLLYVGGWVGGPMRLEYVALALAMVLSLLGMVLLMLGTGRLYAPSLRGRRAIMLPAGALVYIAVPPARDFATSGLESGLVLAYLGLLWWMMVCWSQPLRARPDSQMFLGALAFVAGCSVLVRPEFALIGGLALIMMLIAARTWRRRVLIVLAGGFLPVAYQIFRMGYYGLLVPSTALAKDAAGDKWSQGMIYVSNFNRPYALWVPLVLSVPLGLLLMTARRRPSFLRPVLAPDYGRVARAVQSPPAVVAFIVGSGVLQALYWIRQGGDFMHGRVLLAPLFCLLAPVGVIPILLPDGKDFSRETGRWLVGALSGLWLGIAGWSLWAANSPGMGGDATRVTYSGIVDERRFYAQATGHAHPLTAADYLDYPRMAAVLTALNNTPEGALLLPSGNYNQWDLVPMIRPSSGTAPGGKPAPKPQHAVFFTNMGMLGMNVGLDVRVIDQIGLVNPLAAHTERLKHARIGHDKNLFPDWVIADGPWVKWYPGIPGYIDQQWVTQAEAALQCPATRAVLNSVRAPITLHRFLSNVLHSYEFTRYRIDRVPRYELVRCGLDVPDGPGPPPRE

>34|CORE_REP|Org20_Gene2239#

MLWILGPHTGPLLFDAVASLDTSPLAAARYHGDQDVAPGVLDFAVNVRHDRPPEWLVRQLAALLPELARYPSTDDVHRAQDAVAERHGRTRDEVLPLVGAAEGFALLHNLSPVRAAIVVPAFTEPAIALSAAGITAHHVVLKPPFVLDTAHVPDDADLVVVGNPTNPTSVLHLREQLLELRRPGRILVVDEAFADWVPGEPQSLADDSLPDVLVLRSLTKTWSLAGLRVGYALGSPDVLARLTVQRAHWPLGTLQLTAIAACCAPRAVAAAAADAVRLTALRAEMVAGLRSVGAEVVDGAAPFVLFNIADADGLRNYLQSKGIAVRRGDTFVGLDARYLRAAVRPEWCWWRRLPMGKAWRTHDAVRLADVIDVLDQAYPPRLAQSWDSVGLVCGDPDDVVDSVTVAVDATPAVVDQVPQAGLLLVHHPLLLRGVDTVAANTPKGVLVHRLIRTGRSLFTAHTNADSASPGVSDALAHAVGLTVDAVLDPVPGAADLDKWVIYVPRENSEAVRAAVFEAGAGHIGDYSHCSWSVAGTGQFLAHDGASPAIGSVGTVERVAEDRVEVVAPARARAEVLAAMRAAHPYEEPAFDIFALVPPPVGSGLGRIGRLPKPEPLRTFVARLEAALPPTATGVRAAGNPDLLVSRVAVCGGAGD

>35|CORE_REP|Org60_Gene3475#

MGRHGSRWSRPPCFRVLRLWTYAHRCDLGHTDPLSRRTEMTTTERPTTMCEAFQRTAVMDPDAVALRTPGGNQTMTWRDYAAQVRRVAAGLAGLGVRRGDTVSLMMANRIEFYPLDVGAQHVGATSFSVYNTLPAEQLTYVFDNAGTKVVICEQQYVDRVRASGVPIEHIVCVDGAPPGTLSLTDLYAAASGDFFDFESTWRAVQPEDIVTLIYTSGTTGNPKGVEMTHANLLFEGYAIDEVLGIRFGDRVTSFLPSAHIADRMTGLYLQAMFGTQVTAVADARTIAAALPDVRPTVWGAVPRVWEKLKAGIEFTVARETDEMKRQALAWAMSVAGKRANALLAGESMSDQLVAEWAKADELVLSKLRERLGFGELRWALSGAAPIPKETLAFFAGIGIPIAEIWGMSELSCVATASHPRDGRLGTVGKLLPGLQGKIAEDGEYLVRGPLVMKGYRKEPAKTAEAIDSDGWLHTGDVFDIDSDGYLRVVDRKKELIINVAGKNMSPANIENTILAACPMVGVMMAIGDGRTYNTALLVFDADSLGPYAAQRGLDASPAALAADPEVIARIAAGVAEGNAKLSRVEQIKRFRILPTLWEPGGDEITLTMKLKRRRIAAKYSAEIEELYASELRPQVYEPAAVPSTQPA

>36|CORE_REP|Org23_Gene3671#

MTAAQQDQAPMATPGCREGETYDVVVLGAGPVGQNVADRARAGGLRVAVVERELVGGECSYWACVPSKALLRPVIAISDARRVDGAREAVDGSINTAGVFGRRNRYVAHWDDTGQADWVSGIGATLIRGDGRLDGPRRVVVTKSSGESVALTARHAVVICTGSRPALPDLPGITEARPWTNRQATDNSTVPDRLAIVGAGGVGVEMATAWQGLGASVTLLARGSGLLPRMEPFVGELIGRGLADAGVDVRVGVSVRALGRPNPTGPVVLELDDGTELRVDEVLFATGRAPRTDDIGLETIGLTPGSWLDVDDTCRVRAVDDGWLYAAGDVNHRALLTHQGKYQARIAGTAIGARAAGRPLDTTSWGMHATTADHHAVPQAFFTDPEAAAVGLTADQAAQAGHRIKAIQQRLPRGGRLPSERELIDRSGLSRVTVRAAVGMLQRQGWLVRRQGLGTFVADPVEQELSCGVRTITEVLLSCGVTPQVDVLSHQTGPAPQRISETLGLVEVLCIRRRIRTGDQPLALVTAYLPPGVGPAVEPLLSGSADTETTYAMWERRLGVRIAQATHEIHAAGASPDVADALGLAVGSPVLVVDRTSYTNDGKPLEVVVFHHRPERYQFSVTLPRTLPGSGAGIIEKRDFA

>37|CORE_REP|Org82_Gene3162#

MVPAGLCAYRDLRRKRARKWGDTVTQPDDPRRVGVIVELIDHTIAIAKLNERGDLVQRLTRARQRITDPQVRVVIAGLLKQGKSQLLNSLLNLPAARVGDDEATVVITVVSYSAQPSARLVLAAGPDGTTAAVDIPVDDISTDVRRAPHAGGREVLRVEVGAPSPLLRGGLAFIDTPGVGGLGQPHLSATLGLLPEADAVLVVSDTSQEFTEPEMWFVRQAHQICPVGAVVATKTDLYPRWREIVNANAAHLQRARVPMPIIAVSSLLRSHAVTLNDKELNEESNFPAIVKFLSEQVLSRATERVRAGVLGEIRSATEQLAVSLGSELSVVNDPNLRDRLASDLERRKREAQQAVQQTALWQQVLGDGFNDLTADVDHDLRTRFRTVTEDAERQIDSCDPTAHWAEIGNDVENAIATAVGDNFVWAYQRSEALADDVARSFADAGLDSVLSAELSPHVMGTDFGRLKALGRMESKPLRRGHKMIIGMRGSYGGVVMIGMLSSVVGLGLFNPLSVGAGLILGRMAYKEDKQNRLLRVRSEAKANVRRFVDDISFVVSKQSRDRLKMIQRLLRDHYREIAEEITRSLTESLQATIAAAQVAETERDNRIRELQRQLGILSQVNDNLAGLEPTLTPRASLGRA

>38|CORE_REP|Org2_Gene1795#

MSPQLCPKVSIVSTTHNQAGYARQAFDSFLDQQTDFPVEIIVADDASTDATPAIIREYAERYPHVFRPIFRTENLGLNGNLTGALSAARGEYVALCEADDYWIDPLKLSKQVAFLDRHPKTTVCFHPVRVIWEDGHAKDSKFPPVRVRGNLSLDALILMNFIQTNSAVYRRLERYDDIPADVMPLDWYLHVRHAVHGDIAMLPDTMAVYRRHAQGMWHNQVVDPPKFWLTQGPGHAATFDAMLDLFPGDPAREELIAVMADWILRQIANVPGPEGAPRCRKPSRAIPGSPCWRCSTAGDTRAAAQDPVAQARRRDAEPQGARGCVALPAPTRLSSLTMSTNPGPAEGANQVMAQEHSAGAVQFTAHNVRLDDGTLTIPESSRTLDESSWFISARGILETVFPGDKSHLRLADVGCLEGGYAVGFARMGFQVLGIEVRELNMAACNYIKSKTNLPNLRFVHDNALNIANHGLFDTVFCCGLFYHLENPKQYLETLSSVTNKLLILQTHFSIINRSDKWLRLPTTARQLTDRLLRRPAPVKFMLSAPTEHEGLPGRWFTEFSDDRSFGQRDTAKWASWDNRRSFWIQREHLLQAIKDVGVDLVMEEYDNLEPSIAESLLGGSYAANLRGTFIGIKTR

>39|CORE_REP|Org2_Gene2657#

MSGALETTEEFGNRFVAAIDSAGLAILVSVGHQTGLLDTMAGLPPATSMEIAEAAGLEERYVREWLGGMTTGQIVEYDAGSSTYSLPAHRAGMLTRAAGPDNLAVIAQFVSLLGEVEQKVIRCFREGGGVPYSEYPRFHKLMAEMSGMVFDAALIDVVLPLVDGLPDRLRSGADVADFGCGSGRAVKLMAQAFGASRFTGIDFSDEAVAAGTEEAARLGLANATFERHDLAELDKVGAYDVITVFDAIHDQAQPARVLQNIYRALRPGGVLLMVDIKASSQLEDNVGVPLSTYLYTTSLMHCMTVSLALDGAGLGTVWGRQLATSMLADAGFTDVTVAEIESDVLNNYYIAGSDAPESMTALEVLGGWPVPAAAAAVIGPAGVLATHGDTARVFALASVTKPLVARAAQVAVEEGVVNLDTPAGPPGSTVRHLLAHTSGLAMHSDQALARPGTRRMYSNYGFTVLAESVQRESGIEFGRYLTEAVCEPLGMVTTRLDGGPAAAGFGATSTVADLAVFAGDLLRPSTVSAQMHADATTVQFPGLDGVLPGYGVQRPNDWGLGFEIRNSKSPHWTGECNSTRTFGHFGQSGGFIWVDPKADLALVVLTARDFGDWALDLWPAISDAVLAEYT

>40|CORE_REP|Org59_Gene3667#

MPEAVQEADLLTAAAVALNRHAALLRELGSVFAAAGHELYLVGGSVRDALLGRLSPDLDFTTDARPERVQEIVRPWADAVWDTGIEFGTVGVGKSDHRMEITTFRADSYDRVSRHPEVRFGDCLEGDLVRRDFTTNAMAVRVTATGPGEFLDPLGGLAALRAKVLDTPAAPSGSFGDDPLRMLRAARFVSQLGFAVAPRVRAAIEEMAPQLARISAERVAAELDKLLVGEDPAAGIDLMVQSGMGAVVLPEIGGMRMAIDEHHQHKDVYQHSLTVLRQAIALEDDGPDLVLRWAALLHDIGKPATRRHEPDGGVSFHHHEVVGAKMVRKRMRALKYSKQMIDDISQLVYLHLRFHGYGDGKWTDSAVRRYVTDAGALLPRLHKLVRADCTTRNKRRAARLQASYDRLEERIAELAAQEXLDRVRPDLDGNQIMAVLDIPAGPQVGEAWRYLKELRLERGPLSTEEATTELLSWWKSRGNXXLGSRVRTVVEYCIAGDDGSAGIWNRPFDVDLDGDGRLDAIGLDLDGDGLRDDALADFDGDDVADHAVFDVDNDGTPESYFIDDGSGTWAVAVDRGGQLRWYGLDGVEHTGGPLVDFDGFGGLDDRLLDTDGDGLADRXLCAGDGSV

>41|CORE_REP|Org82_Gene1896#

MRVTRLVDAESTRCDVGPAPKSVAMLHFTAATSRFRLGRERANSVRSDGGWGVLQPVSATFNPPLRGWQRRALVQYLGTQPRDFLAVATPGSGKTSFALRIAAELLRYHTVEQVTVVVPTEHLKVQWAHAAAAHGLSLDPKFANSNPQTSPEYHGVMVTYAQVASHPTLHRVRTEARKTLVVFDEIHHGGDAKTWGDAIREAFGDATRRLALTGTPFRSDDSPIPFVSYQPDADGVLRSQADHTYGYAEALADGVVRPVVFLAYSGQARWRDSAGEEYEARLGEPLSAEQTARAWRTALDPEGEWMPAVITAADRRLRQLRAHVPDAGGMIIASDRTTARAYARLLTTMTAEEPTVVLSDDPGSSARITEFAQGTSRWLVAVRMVSEGVDVPRLSVGVYATNASTPLFFAQAIGRFVRSRRPGETASIFVPSVPNLLQLASALEVQRNHVLGRPHRESAHDPLDGDPATRTQTERGGAERGFTALGADAELDQVIFDGSSFGTATPTGSDEEADYLGIPGLLDAEQMRALLHRRQDEQLRKRAQLQKGATQPATSGASASVHGQLRDLRRELHTLVSIAHHRTGKPHGWIHDELRRRCGGPPIAAATRAQIKARIDALRQLNSERS

>42|CORE_REP|Org59_Gene3058#

MAWSSVRSFEDGIFHMSAPSLGHGGDDGAIDILLVGLDSRTDAHGNPLSAEELATLHAGDEEATNTDTIILIRVPNNGKSATAISXPRDSYVAAPGLGKTKINGVYGQXRETKRAGLVQAXASPTEAAAAGTEAXREALIKTVADLTGVTVDHYAEIGLLGFALIADALGGVDVCLKEPVYEPLSGADFPAGRQKLNGPQALSFVRQRHDLPRGDLDRVVRQQAVMAALAHRVISGQTLSSPATLXRLEQAVQRSVVLSSGWDIMDFVRQLQKLAGGNVAFATIPVLDGAGWSDDGMQSVVRVDPRQVQDWVVGLLHEQDQGKTDELAYTPAKTTANVVNDTDINGLAAAVSKVLSSKGFTTGSVGNNDGDHVPGSQVRAAKADDLGAQQVAKELGGLPVVADASIAPGSVRVVLANDYSGPGSGLGGSDPNGVVSXARAJXPRVRRRHGXPRRRQSLPPAPTRRSASTDHTDHPERGDPGSDAARRPGRPAPITYYDDATGERIELSAVTLANWAAKTGNLLRDELAAGPASRVAILLPAHWQTAAVLFGVWWXGAQAILDDSPADVALCTADRLAEADAVVNSAAVAGEVAVLSLDPLPVDRXPXCRSASPTMRPRCGYTATR

>43|CORE_REP|Org1_Gene1647#

MASRQTPAELARCDLAKTAEREHTPTATATTPSVAGNVMPMSVRSLPAALRACARLQPHDPAFTFMDYEQDWDGVAITLTWSQLYRRTLNVAQELSRCGSTGDRVVISAPQGLEYVVAFLGALQAGRIAVPLSVPQGGVTDERSDSVLSDSSPVAILTTSSAVDDVVQHVARRPGESPPSIIEVDLLDLDAPNGYTFKEDEYPSTAYLQYTSGSTRTPAGVVMSHQNVRVNFEQLMSGYFADTDGIPPPNSALVSWLPFYHDMGLVIGICAPILGGYPAVLTSPVSFLQRPARWMHLMASDFHAFSAAPNFAFELAARRTTDDDMAGRDLGNILTILSGSERVQAATIKRFADRFARFNLQERVIRPSYGLAEATVYVATSKPGQPPETVDFDTESLSAGHAKPCAGGGATSLISYMLPRSPIVRIVDSDTCIECPDGTVGEIWVHGDNVANGYWQKPDESERTFGGKIVTPSPGAPEGPWLRTGDSGFVTDGKMFIIGRIKDLLIVYGRNHSPDDIEATIQEITRGRCAAISVPGDRSTEKLVAIIELKKRGDSDQDAMARLGAIKREVTSALSSSHGLSVADLVLVAPGSIPITTSGKVRRGACVEQYRQDQFARLDA

>45|CORE_REP|Org148_Gene3863#

MTTTIRNGRGDLITAIGGPCDVQALPESQLPELAVQMRRRLIETVTATGGHLGAGLGMVELTIALHRVFTSPHDIVVFDTGHQTYPHKLLTGRGKDFATLRQADGLSGYPNRHESPHDWVENSHASVSLAWVDGIAKALALQGQCDRRVIAVIGDGALTGGVAWEGLNNLGAATRPVIVVLNDNGRSYDPTAGALAAHLEELRVGTPRGPNLFENMGFTYIGPVDGHNIPDTCAVLRKAAAAARPVVVHAVTSKGRGYPPAEADERDHMHACGVVDIATGLASTPSQRSWTDVFEDEIARIADDRSDVVGLTAAMRLPTGLGALSRRYPHRVFDSGIAEQHLLASAAGLAAAGTHPVVAVYSTFLHRAFDQLLFDIGLHRLPVTLVLDRAGVTGPDGPSHHGLWDLALLACVPGFQIACPRDAPRLRQQLRTAIATAAPTAVRFPKGAPGEPITAEHTIGGLDVLHTPPPHWRPDVLLVAVGAMSRPCMDAARCLSEEQIGVTVVDPQWVWPISPALTELAGRHRITVCVEDAIADVGIGAHLSHHIGRTHPRTRTYTLGLPPAYIPHASRDHILSSHGLTGPAIRIRCKSLLNALHEVPGPEDHPDSGDSY

>46|CORE_REP|Org137_Gene2624#

MTDTDLITAGESTDGKPSDAAATDPPDLNADEPAGSLATMVLPELRALANRAGVKGTSGMRKNELIAAIEEIRRQANGAPAVDRSAQEHDKGDRPPSSEAPATQGEQTPTEQIDSQSQQVRPERRSATREAGPSGSGERAGTAADDTDNRQGGQQDAKTEERGTDAGGDQGGDQQASGGQQARGDEDGEARQGRRGRRFRDRRRRGERSGDGAEAELREDDVVQPVAGILDVLDNYAFVRTSGYLPGPHDVYVSMNMVRKNGMRRGDAVTGAVRVPKEGEQPNQRQKFNPLVRLDSINGGSVEDAKKRPEFGKLTPLYPNQRLRLETSTERLTTRVIDLIMPIGKGQRALIVSPPKAGKTTILQDIANAITRNNPECHLMVVLVDERPEEVTDMQRSVKGEVIASTFDRPPSDHTSVAELAIERAKRLVEQGKDVVVLLDSITRLGRAYNNASPASGRILSGGVDSTALYPPKRFLGAARNIEEGGSLTIIATAMVETGSTGDTVIFEEFKGTGNAELKLDRKIAERRVFPAVDVNPSGTRKDELLLSPDEFAIVHKLRRVLSGLDSHQAIDLLMSQLRKTKNNYEFLVQVSKTTPGSMDSD

>49|CORE_REP|Org8_Gene4056#

MAVHLTRIYTRTGDDGTTGLSDMSRVAKTDARLVAYADCDEANAAIGAALALGHPDTQITDVLRQIQNDLFDAGADLSTPIVENPKHPPLRIAQSYIDRLEGWCDAYNAGLPALKSFVLPGGSPLSALLHVARTVVRRAERSAWADNDVRNVRLFRALLGVDKRTVIEDIEFEEDDAGDGARVIARVRPRSAVLRRCGRCGRKASWYDRGAGLRQWRSLDWGTVEVFLEAEAPRVNCPTHGPTVVAVPWARHHAGHTYAFDDTVAWLAVACSKTAVCELMRIAWRTVGAIVARVWADTEKRIDRFANLRRIGIDEISYKRHHRYLTVVVDHDSGRLVWAAPGHDKATLGLFFDALGAERAAQITHVSADAADWIADVVTERCPDAIQCADPFHVVAWATEALDVERRRAWNDARAIARTEPKWGRGRPGKNAAPRPGRERARRLKGARYALWKNPEDLTERQSAKLAWIAKTDPRLYRAYLLKESLRHVFSVKGEEGKQALDRWISWAQRCRIPVFVELAARIKRHRVAIDAALDHGLSQGLIESTNTKIRLLTRIAFGFRSPQALIALAMLTLAGHRPTRPGRHNHPQISQ

>50|CORE_REP|Org101_Gene3729#

MAARHHTLSWSIASLHGDEQAVGAPLTTTELTALARTRLFGATGTVLMAIGALGAGARPVVQDPTFGVRLLNLPSRIQTVSLTMTTTGAVMMALAWLMLGRFTLGRRRMSRGKLDRTLLLWMLPLLIAPPMYSKDVYSYLAQSEIGRDGLDPYRVGPASGLGLGHVFTLSVPSLWRETPAPYGPLFLWIGRGISSLTGENIVAAVLCHRLVVLIGVTLIVWATPRLAQRCGVAEVSALWLGAANPLLIMHLVAGIHNEALMLGLMLTGVEFALRGLDMANTPRPSPETWRLGPATIRASRRPELGASPRAGASRAVKPRPEWGPLAMLLAGSILITLSSQVKLPSLLAMGFVTTVLAYRWGGNLRALLLAAAVMASLTLAIMAILGWASGLGFGWINTLGTANVVRSWMSPPTLLALGTGHVGILLGLGDHTTAVLSLTRAIGVLIITVMVCWLLLAVLRGRLHPIGGLGVALAVTVLLFPVVQPWYLLWAIIPLAAWATRPGFRVAAILATLIVGIFGPTANGDRFALFQIVDATAASAIIVILLIALTYTRLPWRPLAAEQVVTAAESASKTPATRRPTAAPDAYADST

>51|CORE_REP|Org73_Gene3981#

MSFVIVAPEALMSVASEVAGIGSALNAANAAAAAPTTGVLAAAADEVSAAMAALFGAHAQEYQRLSAQAAGFHAQFVQALNAGVNSYASAEAANASPLQAVEQQVLGLINGPAQTLLGRPLIGNGADGAPGTGQPGGPGGLLWGNGGNGGSGVAGVGGPGGSGGAAGLFGHGGNGGAGGSNAAGAGGVGGAGGAGWLVGNGGAGGFGGVGTTVSGNGGAGGAAGAFGNGGVGGAGGAAVIGGLPGNGGAGGNAGLIGAGGDGGVGGVGAPGTNGMNPPPNQTSQAANGSPGANNGAGSGGAGLPGNPGAVPGRAGGAGGLGGSGSDTSEGPVTGGNGGNGGDGGPGAPGGNGAPGGIGVNTGTGWAYGGNGGNGGDGGAGARGGDGGNGGNGLALNGGNGIGGNGGAGGRGGTGAAGGNGGIGGGATGTLTFFGSGGDGGPGGAGANTAGTGGVGGVGGAGGQGGLLFGDGGNGGAGGAGGIGGTGASGGAGGKGGSGLVGGDGGNGGAGGAGGNGGKGGAGGAGGGAGMFSQPGVHGAGGTGGQGGAGGAGGAGGAAGAGTVVAGNPGDPGGFGAAGADGLPG

>52|CORE_REP|Org59_Gene823#

MRLILNVIWLVFGGLWLALGYLLASLVCFLLIITIPFGFAALRIASYALWPFGRTIVEKPTAGTGALIGNVIWVLLFGIWLALGHLVSAAAMAGHDHRHSASTGQLETDPGVAGAAXQGHRRGQLTGAHMTLXALGMPALRSRTNGIADPRVVPTTGPLVDTFGRVANDLRVSLTDRCNLRCSYCMPERGLRWLPGEQLLRPDELARLIHIAVTRLGVTSVRFTGGEPLLAHHLDEVVAATARLRPRPEISLTTNGVGLARRAGALAEAGLDRVNVSLDSIDRAHFAAITRRDRLAHVLAGLAAAKAAGLTPVKVNAVLDPTTGREDVVDLLRFCLERGYQLRVIEQXPLDAGHSWRRNIALSADDVLAALRPHFRLRPDPAPRGSAPAELWLVDAGPNTPRGRFGVIASVSHAILXBXXXXXXDRRWPDXLAXLFSTEETDLRRLLRGGADDDAIEAAWRAAMWSKPAGHGINAPDFIQPDRPMSAIGXXPVTQVSDESAGIQVTVRYFAAARAAAGAGSEKVTLRSGATVAELIDGLSVRDVRLATVLSRCSYLRDGIVVRDDAVALSAGDTIDVLPPFAGG

>53|CORE_REP|Org75_Gene2858#

MSAADEQGEERATRKSAPDLRLPGSVAEILASPAGPKVGAFFDLDGTLVAGFTAVILTQERLRRRDMGVGELLGMVQAGLNHTLGRIEFEDLIGKAAAALAGRLLTDLEEIGERLFAQRIESRIYPEMRELVRAHVARGHTVVLSSSALTIQVGPVARFLGINNMLTNKFETNEDGILTGGVLKPILWCPGKATAVQRFAAEHDIDLKDSYFYADGDEDVALMYLVGNPRPTNPEGKMAAVAKRRGWPILKFNSRGGVGIRRQLRTLAGLSTIVPVAAGAVGIGVLTGSRRRGVNFFTSTFSQLLLATSGVHLNVIGKENLTAQRPAVFIFNHRNQVDPVIAGALVRDNWVGVGKKELASDPIMGTLGKLLDGVFIDRDDPVAAVETLHTVEERARNGLSIVIAPEGTRLDTTEVGSFKKGPFRIAMAAKIPIVPIVIRNAEIVASRNSTTINPGTVDVAVFPPIPVDDWTLDALPDRIAEVRQLYLDTLADWPVDGLPAVDLYAEQKAARKARAQVAKATAKRVPAKKAPAKSAANKGAAATKAATKKASPKAKPSESKIAGKDGEASASPSSSAKGRS

>54|CORE_REP|Org2_Gene3601#

MTDIEVALPFWLDRPDHEATDVALAAADTGFAALWIGEMATYDAFALATSIGLRTPNMTLKVGPLAVGVRGPVGLALGVSSVASLTGCRVDLALGASSPAIVAGWHGRPWAHHVPVMRETIECLRSIFTGARVEYSGRHVNSRGFRLRGAAPDTRIALGAFGPGMIRLAAQHADEVVLNLASPFRVGRVRAAIDSAAAAAGRAAPRLTVWVPVAVNPGAAAHSQLAAQLAVYLAPPGYGEMFSALGFDGLVRSARSRATRRELAVAVPSELLDRVCALGSPDRVAARLRAYADAGADCVAVVPATAEDPGAGWLCERCDPAGSTAPLATTTADGNLDRDGGVAMSGGLFGLLDHVAVLARLAAASIDDIGAAAGRATAKAAGVVIDDTAVTPQYVHRITAERELPIIKRIAIGSVRNKLLLILPGALLLSQLVPWLLTPLLMLGATYLCYEGAEKVCGVIGGRGHDAAPQVAERELVAGAIRTDFILSAEIMVIALNEVADQPFVPRLIVLVIVALVITAAVYGVVAVIVQMDDVGLRLTQTASRFGQRIGGAWSRGCPNCYRRCQRSGWEPCSGWAAT

>55|CORE_REP|Org24_Gene2374#

MALYRKYRPASFAEVVGQEHVTAPLSVALDAGRINHAYLFSGPRGCGKTSSARILARSLNCAQGPTANPCGVCESCVSLAPNAPGSIDVVELDAASHGGVDDTRELRDRAFYAPVQSRYRVFIVDEAHMVTTAGFNALLKIVEEPPEHLIFIFATTEPEKVLPTIRSRTHHYPFRLLPPRTMRALLARICEQEGVVVDDAVYPLVIRAGGGSPRDTLSVLDQLLAGAADTHVTYTRALGLLGVTDVALIDDAVDALAACDAAALFGAIESVIDGGHDPRRFATDLLERFRDLIVLQSVPDAASRGVVDAPEDALDRMREQAARIGRATLTRYAEVVQAGLGEMRGATAPRLLLEVVCARLLLPSASDAESALLQRVERIETRLDMSIPAPQAVPRPSAAAAEPKHQPAREPRPVLAPTPASSEPTVAAVRSMWPTVRDKVRLRSRTTEVMLAGATVRALEDNTLVLTHESAPLARRLSEQRNADVLAEALKDALGVNWRVRCETGEPAAAASPVGGGANVATAKAVNPAPTANSTQRDEEEHMLAEAGRGDPSPRRDPEEVALELLQNELGARRIDNA

>56|CORE_REP|Org88_Gene3088#

MTTGGLVDENDGAAMRPLRHTLSQLRLHELLVEVQDRVEQIVEGRDRLDGLVEAMLVVTAGLDLEATLRAIVHSATSLVDARYGAMEVHDRQHRVLHFVYEGIDEETVRRIGHLPKGLGVIGLLIEDPKPLRLDDVSAHPASIGFPPYHPPMRTFLGVPVRVRDESFGTLYLTDKTNGQPFSDDDEVLVQALAAAAGIAVANARLYQQAKARQSWIEATRDIATELLSGTEPATVFRLVAAEALKLTAADAALVAVPVDEDMPAADVGELLVIETVGSAVASIVGRTIPVAGAVLREVFVNGIPRRVDRVDLEGLDELADAGPALLLPLRARGTVAGVVVVLSQGGPGAFTDEQLEMMAAFADQAALAWQLATSQRRMRELDVLTDRDRIARDLHDHVIQRLFAIGLALQGAVPHERNPEVQQRLSDVVDDLQDVIQEIRTTIYDLHGASQGITRLRQRIDAAVAQFADSGLRTSVQFVGPLSVVDSALADQAEAVVREAVSNAVRHAKASTLTVRVKVDDDLCIEVTDNGRGLPDEFTGSGLTNLRQRAEQAGGEFTLASVPGASGTVLRWSAPLSQ

>57|CORE_REP|Org59_Gene611#

MKLTDVDFAVEASGMVRAFNQAGVLDVSDVHVAQRLCALAGESDERVALAVAVAVRALRAGSVCVDLLSIARVAGHDDLPWPDPADWLAAVRASPLLADPPVLHLYDDRLLYLDRYWREEEQVCADLLALLTSRRPAGVPDLRRLFPTGFDEQRRAAEIALSQGVTVLTGGPGTGKTTTVARLLALVAEQAELAGEPRPRIALAAPTGKAAARLAEAVRREMAKLDATDRARLGDLHAVTLHRLLGAKPGAXXRLXXRQNRLPHNVIVVDETSMVSLTLMARLAEAVRPGARLILVGDADQLASVEAGAVLADLVDGFSVRDDALVAQLRTSHRFGKVIGTLAEAIRAGDGDAVLGLLRSGEERIEFVDDEDPAPRLRAVLVPHALRLREAALLGASDVALATLDEHRLLCAHRDGPTGVLHWNRRVQAWLAEETGQPPWTPWYAGRPLLVTANDYGLRVYNGDTGVVLAGPTGLRAVISGASGPLDVATGRLGDVETMHAMTIHKSQGSQVDEVTVLMPQEDSRLLTRELLYTAVTRAKRKVRVVGSEASVRAAIARRAVRASGLRMRLQSTGCG

>58|CORE_REP|Org70_Gene4023#

MPAKKTMAQRLGQALETMTRQCGQLPETPAYGSWLLGRVSESPSRRWVRIKRIVTVYIMTANLTGIVVALLVVTFAFPVPSIYTDAPWWVTFGVAPAYATLALAIGTYWITTRIVRASIRWAIEERAPSQADGRNTLLLPFRVAAVHLILWDIGGALLATLYGLANRVFVTIILFSVTICGVLVATNCYLFTEFALRPVAAKALEAGRPPRRFAPGIMGRTMTVWSLGSGVPVTGIATTALYVLLVHNLTETQLASAVLILSITTLIFGFLVMWILAWLTAAPVRVVRAALKRVEQGDLRGDLVVFDGTELGELQRGFNAMVNGLRERERVRDLFGRHVGREVAAAAERERPKLGGEERHVAVVFVDIVGSTQLVTSRPAAEVVMLLNRFFTVIVDEVNHHRGLVNKFQGDASLAVFGAPNRLSHPEDAALATARAIADRLASEMPECQAGIGVAAGQVVAGNVGAHERFEYTVIGEPVNEAARLCELAKSYPSRLLASSQTLRGASENECARWSLGETVTLRGHDQPIRLASPVQQLQMPAQSADIVGGALGDHQTHTIYRGAHPTD

>59|CORE_REP|Org59_Gene2985#

MDDGSVSDIKRGRAARNAKLASIPVGFAGRAALGLGKRLXGKSKDEVTAELMEKAANQLFTVLGELKGGAMKVGQALSVMEAAIPDEFGEPYREALTKLQKDAPPLPASKVHRVLDGQLGTKWRXRXSSFNDTPVASASIGQVHKAIWSDGREVAVKIQYPGADEALRADLKTMQRMVGVLKQLSPGADVQGMVDELVERTEMELDYRLEAANQRAFAKAYHDHPRFQVPHVVASAPKVVIQEWIEGVPMAEIIRHGTTEQRDLIGTLLAELTFDAPRRLGLMHGDAHPGNFMLLPDGRMGIIDFGAVAPMPGGFPIELGMTIRLAREKNYDLLLPTMEKAGLIQRGRQVSVREIDEMLRQYVEPIQVEVFHYTRKWLQKMTVSQIDRSVAQIRTARQMXLPAKLAIPMRVIASVGAILCQLDAHVPIKALSXXADPGFRRARRDRRLSRLAPAGAPSRAMQQHPCADVRADACDSRSSLGRISATTPNAPGFSPLKRRGQALPPDRAAHTQCLGYLZTGRGIGEPQIGITDVARQNRQSLSGGLSGDCQYRHVLFHLLPGLVAVLRK

>60|CORE_REP|Org150_Gene692#

MVLRSRKSTLGVVVCLALVLGGPLNGCSSSASHRGPLNAMGSPAIPSTAQEIPNPLRGQYEDLMEPLFPQGNPAQQRYPPWPASYDASLRVSWRQLQPTDPRTLPPDAPDDRKYDFSVIDNALTRLADRGMRLTLRVYAYSSCCKASYPDGTNIAIPDWERAIASTNTSYPGPATDPSTGVVQVVPNFNDSTYLNDFAQLLAALGRRYDGDERLSVFEFSGYGDFSENHVAYLRDTLGAPGPGPDESVATLGYYSQFRDQNITTASIKQLIAANVSAFPHTQLVTSPANPEIVRELFADEVTNKLAAPVGVRSDCLGVDAPLPAWAESSTSHYVQTKDPVVAALRQRLATAPVITEWCELPTGSSPRAYYEKGLRDVIRYHVSMTSSVNFPDQTATSPMDPALYLVWAQANAAAGYRYSVEAQPGSQALAGKVATISVTWTNYGAAAATEKWVPGYRLVDSTGQVVRTLPAAVDLKTLVSDQRGDRSSDQPTPASVAETVRVDLSGLPAGHYTLRAAIDWQQHKPNGSHVVNYPPMLLSRDGRDDSGFYPVATLDIPRDAQTAVNAS

>61|CORE_REP|Org120_Gene2512#

MALTCTDMSDAVAGSDAEGLTADAIVVGAGLAGLVAACELADRGLRVLILDQENRANVGGQAFWSFGGLFLVNSPEQRRLGIRDSHELALQDWLGTAAFDRPEDYWPEQWAHAYVDFAAGEKRSWLRARGLKIFPLVGWAERGGYDAQGHGNSVPRFHITWGTGPALVDIFVRQLRDRPTVRFAHRHQVDKLIVEGNAVTGVRGTVLEPSDEPRGAPSSRKSVGKFEFRASAVIVASGGIGGNHELVRKNWPRRMGRIPKQLLSGVPAHVDGRMIGIAQKAGAAVINPDRMWHYTEGITNYDPIWPRHGIRIIPGPSSLWLDAAGKRLPVPLFPGFDTLGTLEYITKSGHDYTWFVLNAKIIEKEFALSGQEQNPDLTGRRLGQLLRSRAHAGPPGPVQAFIDRGVDFVHANSLRELVAAMNELPDVVPLDYETVAAAVTARDREVVNKYSKDGQITAIRAARRYRGDRFGRVVAPHRLTDPKAGPLIAVKLHILTRKTLGGIETDLDARVLKADGTPLAGLYAAGEVAGFGGGGVHGYRALEGTFLGGCIFSGRAAGRGAAEDIR

>63|CORE_REP|Org59_Gene1516#

MHASRPGAPPHAGLPSRRTAGDQDHRADPKVTRIMSASTLEQPAAAHVDELVARMRGRLLDPLAIAVLAAVISGAWASRPSLWFDEGATISASASRTLPELWSLLGHIDAVHGLYYLLMHGWFAIFPPTELWSRLPSCLAIGAAAAGVVVFAKQFSGRXTXVCXGAVFAILPRVTWAGIEARSSALSVAAAVWLTVLLVAAVRCNTQRRWLXYALXLMLSILVSINLALLVPAYATMVPLLASGKSRKSPVIWWTVVTAAALGAMTPFILFAHGQVWQXGXIAGLNRNIILDVIXRQYFDHSVPFXILAGLIVAAGIAAHLAGARGPGGDTHRLXPCXSAXAXTXXXPTAVVLIYSATVXPIYYPXYLIXXAPAAAVILAVCVVTIARKPWLXAGVVFLLAAAAFPNYFFTQRGPYAKEGWDYSQVADVISAHAKPGDCLLVDNTAGWRPGPIRALLATRPAAFRSLIDVERGTYGPKVGTLWDGHVAVWLTTAKIDKCPTLWTIANRDKSLPDHQVGEMLSPGTGFGRTPVYRFPSYLGFRIVERWQFHYSQVVKSTR

>64|CORE_REP|Org2_Gene4222#

MPVVKINAIEVPAGAGPELEKRFAHRAHAVENSPGFLGFQLLRPVKGEERYFVVTHWESDEAFQAWANGPAIAAHAGHRANPWRPVLRCWNSRSCLTSVGPARLHNRRAGRRMLALSAAAALIVALASGCSSAPTPSANAANHGHRIDTRTPPGLRAQQTMDMLNSDWPIGEIGVGTLAAPGQVDTVKTTMEALWWDRPFALAGVDIGASVAALHLISSYGAQQDIRIHTDDDGWVDRFDVETQAPSIASWRDVDAVLSKTGARYSFQVAKVDNGRCDPVAGTNTGESLPLASIFKLYVLHALAGAVQHNTVSWDDLLTVTAKSKAVGSSGLELPVGARVSVRTAAEKMIATSDNMATDLLIERLGTRAIEEALASAGHHDPASMTPFPTMYELFSVGWGKPDLRDQWKHATQQVRAQILRQTNSTPYQPDPTRAHTPASNYGAEWYGSAEDICRVHAALRADAVGPASPVRQIMSAVPGIQLDRSVWPYIGAKAGGLPGDLTFSWYAVDKTGQPWVVSFQLNWPRDHGPTVTGWMLQVARQVFALIAPQ

>67|CORE_REP|Org23_Gene3965#

MSFVSVAPEIVVAAATDLAGIGSAISAANAAAAAPTTAVLAAGADEVSAAIAALFSGHAQAYQALSAQAAAFHQQFVQTLAGGAGAYAAAEAQVEQQLLAAINAPTQALLGRPLIGNGADGAPGTGQAGGAGGILYGNGGNGGSGAAGQAGGAGGPAGLIGHGGSGGAGGSGAAGGAGGHGGWLWGNGGVGGSGGAGVGAGVAGGHGGAGGAAGLWGAGGGGGNGGNGADANIVSGGDGGLGGAGGGGGWLYGDGGAGGHGGQGAGGGAGGAGGDGGQGGAGRGLWGTGGAGGHGGQGGGTGGPPLPGQAGMGAAGGAGGLIGNGGAGGDGGVGASGGVAGVXGAGGNAMLIGHGGAGGAGGDSSFANGAAGGAGGAGGHLFGNGGSGGHGGAVTAGNTGIGGAGGVGGDARLIGHGGAGGAGGDRAGALVGRDGGPGGNGGAGGQLYGNGGDGGPGGQGGQAFGANNIGGTGGAGGNGGPAILSGNGGNGGAGGAGGAGGAGGGAGGVGGAGGAPGTGGTLQAAVSGLVTALFGAPGQPGDTGQPG

>68|CORE_REP|Org59_Gene1821#

MHPRAGDRAQPRGGGXAXRENLGGVVVRLGVFLAVCLLTAFLLIAVFGEVRFGDGKTYYAEFANVSNLRTGKLVRIAGVEVGKVTRISINPDATVRVQFTADNSVTLTRGTRAVIRYDNLFGDRYLALEEGAGGLAVLRPGHTIPLARTQPALDLDALIGXXKPLFRALNPEQVNALSEQLLHAFAGQGPTIGSLLAQSAAVTNTLADRDRLIGQXITNLNVVLGSLGAHTDRLDQAVTSLSALIHRLXQRKTDISNAVAYTNAAAGSVADLLSQARAPLXEGGSRDRSGGRHRGRRPRLPRQSAQHAAGQIPGAGPPGYVRXLLRLLPVRRRXQGQRQGRPAGVHQAGRSGQRAVRAEMKSFAERNRLAIGTVGIVVVAAVALAALQYQRLPFXXQGTRVSAYFADAGGLRTGNTXXGLRLSGGKGVQHLARRTGRAGGVQGRHRRPTRKPHRSGNQNQGLVGQQVPRRHPPRGRPTRFSDPDRADHVALPTARRPWRFGRHDQRVAHXAAVRIAGHPGADLCRYAGALPQRHTRGGPARPNPR

>69|CORE_REP|Org2_Gene4492#

MGGLLIAGLVTAIPAVGRAPERLAGYIASNPVPSTGAKINASFNRVASGDCLMWPDGTPESAAIVSCADEHRFEVAESIDMRTFPGMEYGQNAAPPSPARIQQISEEQCEAAVRRYLGTKFDPNSKFTISMLWPGDRAWRQAGERRMLCGLQSPGPNNQQLAFKGKVADIDQSKVWPAGTCLGIDATTNQPIDVPVDCAAPHAMEVSGTVNLAERFPDALPSEPEQDGFIKDACTRMTDAYLAPLKLRTTTLTLIYPTLTLPSWSAGSRVVACSIGATLGNGGWATLVNSAKGALLINGQPPVPPPDIPEERLNLPPIPLQLPTPRPAPRLSSCQVPHQALSTSLPNSQWLRPPGHPNRMRQRRQHRPRPSHRHQTPERRRRPNHQRPHRLAPPSPHRQASRVTVRMDPQRFDELVSDALDLIPPELADAMDNVVVLVANRHPQHENLLGQYEGVALTERGSDYAGSLPDAITIYREALLDACDSEDEVVDQVAITVIHEVAHHFGIDDERLDQLGWRDEPAPGRGNPDLSAPDAMNGP

>70|CORE_REP|Org20_Gene3484#

MDFGALPPEINSARMYAGPGSASLVAAAKMWDSVASDLFSAASAFQSVVWGLTVGSWIGSSAGLMAAAASPYVAWMSVTAGQAQLTAAQVRVAAAAYETAYRLTVPPPVIAENRTELMTLTATNLLGQNTPAIEANQAAYSQMWGQDAEAMYGYAATAATATEALLPFEDAPLITNPGGLLEQAVAVEEAIDTAAANQLMNNVPQALQQLAQPAQGVVPSSKLGGLWTAVSPHLSPLSNVSSIANNHMSMMGTGVSMTNTLHSMLKGLAPAAAQAVETAAENGVWAMSSLGSQLGSSLGSSGLGAGVAANLGRAASVGSLSVPPAWAAANQAVTPAARALPLTSLTSAAQTAPGHMLGGLPLGHPRRQRYPAHWRRHGPTRYPAHRPPDSTTGLRGCVGVVPRRGWRALAIWSKGPDPTGRTPRHRGAVDGIRKAVTGNGIDAGTTIRDDHDGRRQPRDLDGRCYAGDQPARAGLTMAQAGGNLPDQQPTHQRVSRFPAALRSTNRPPRHGRRRRHLVGNKCGRHQRGFACVIPSHRV

>71|CORE_REP|Org23_Gene3889#

MILPAPHVEYFLLAPMLIVFSVAVAGVLAEAFLPRRWRYGAQVTLALGGSAVALIAVIVVARSIHGSGHXAVLGAIAVDRATLFLQGTVLLVTIMAVVFMAERSARVSPQRQNTLAVARLPGLDSFTPQLSAVPGSDAERQAERAGATQTELFPLAMLSVGGMMVFPASNDLLTMFVALEVLSLPLYLMCGLARNRRLLSQEAAMKYFLLGAFSSAFFLYGVALLYGATGTLTLPGIRDALAARTDDSMALAGVALLAVGLLFKVGAVPFHSWIPDVYQGAPTPITGFMAAATKVAAFGALLRVVYVALPPLHDQWRPVLWAIAILTMTVGTVTAVNQTNVKRMLAYSSVAHVGFILTGVIADNPAGLSATLFYLVAYSFSTMGAFAIVGLVRGADGSAGSEDADLSHWAGLGQRSPIVGVMLSMFLLAFAGIPLTSGFVSKFAVFRAAASAGAVPLVIVGVISSGVAAYFYVRVIVSMFFTEESGDTPHVAAPGVLSKAAIAVCTVVTVVLGIAPQPVLDLADQAAQLLR

>72|CORE_REP|Org59_Gene176#

MSTIFDIRNLRLPQLSRASXVIGSLVVVLALAAGIVGVRLYQKLTNNTVVAYFTQANALYVGDKVQIMGLPVGSIDKIXPAGDKMKVTFHYQNKYKVPANASAVILNPTLVASRNIQLEPPYRGGPVLADNAVIPVERTQVPTEWDELRDSVSHIIDELGPTPEQPKGPFGEVIEAFADGLAGKGKQINTTLNSLSQALNALNEGRGDFFAVVRSLALFVNALHQDDQQFVALNKNLAEFTDRLTHSDADLSNAIQQFDSLLAVARPFFAKNREVLTHDVNNLATVTTTLLQPDPLDGLETVLHIFPTLAANINQLYHPTHGGVVSLSAFTNFANPMEFICSSIQAGSRLGYQESAELCAQYLAPVLDAIKFNYFPFGLNVASTASTLPKEIAYSEPRLQPPNGYKDTTVPGIWVPDTPLSHRNTQPGWVVAPGMQGVQVGPITQGLLTPESLAELMGGPDIAPPSSGLQTPPGPPNAYDEXPVLPPIGLQAPQVPIPPPPPGPDVIPGPVPPTPAPVGAPLPADGRXXXX

>73|CORE_REP|Org87_Gene1778#

MAATKASTATDEPVKRTATKSPAASASGAKTGAKRTAAKSASGSPPAKRATKPAARSVKPASAPQDTTTSTIPKRKTRAAAKSAAAKAPSARGHATKPRAPKDAQHEAATDPEDALDSVEELDAEPDLDVEPGEDLDLDAADLNLDDLEDDVAPDADDDLDSGDDEDHEDLEAEAAVAPGQTADDDEEIAEPTEKDKASGDFVWDEDESEALRQARKDAELTASADSVRAYLKQIGKVALLNAEEEVELAKRIEAGLYATQLMTELSERGEKLPAAQRRDMMWICRDGDRAKNHLLEANLRLVVSLAKRYTGRGMAFLDLIQEGNLGLIRAVEKFDYTKGYKFSTYATWWIRQAITRAMADQARTIRIPVHMVEVINKLGRIQRELLQDLGREPTPEELAKEMDITPEKVLEIQQYAREPISLDQTIGDEGDSQLGDFIEDSEAVVAVDAVSFTLLQDQLQSVLDTLSEREAGVVRLRFGLTDGQPRTLDEIGQVYGVTRERIRQIESKTMSKLRHPSRSQVLRDYLD

>74|CORE_REP|Org119_Gene2064#

MGMRLSRRDKIARMLLIWAALAAVALVLVGCIRVVGGRARMAEPKLGQPVEWTPCRSSNPQVKIPGGALCGKLAVPVDYDRPDGDVAALALIRFPATGDKIGSLVINPGGPGESGIEAALGVFQTLPKRVHERFDLVGFDPRGVASSRPAIWCNSDADNDRLRAEPQVDYSREGVAHIENETKQFVGRCVDKMGKNFLAHVGTVNVAKDLDAIRAALGDDKLTYLGYSYGTRIGSAYAEEFPQRVRAMILDGAVDPNADPIEAELRQAKGFQDAFNNYAADCAKNAGCPLGADPAKAVEVYHSLVDPLVDPDNPRISRPARTKDPRGLSYSDAIVGTIMALYSPNLWQHLTDGLSELVDNRGDTLLALADMYMRRDSHGRYNNSGDARVAINCVDQPPVTDRDKVIDEDRRAREIAPFMSYGKFTGDAPLGTCAFWPVPPTSQPHAVSAPGLVPTVVVSTTHDPATPYKAGVDLANQLRGSLLTFDGTQHTVVFQGDSCIDEYVTAYLIGGTTPPSGAKC

>75|CORE_REP|Org9_Gene1083#

MHADLAATTSREDFRLLAAEHRVVPVTRKVLADSETPLSAYRKLAANRPGTFLLESAENGRSWSRWSFIGAGAPTALTVREGQAVWLGAVPKDAPTGGDPLRALQVTLELLATADRQSEPGLPPLSGGMVGFFAYDMVRRLERLPERAVDDLCLPDMLLLLATDVAAVDHHEGTITLIANAVNWNGTDERVDWAYDDAVARLDVMTAALGQPLPSTVATFSRPEPRHRAQRTVEEYGAIVEYLVDQIAAGEAFQVVPSQRFEMDTDVDPIDVYRILRVTNPSPYMYLLQVPNSDGAVDFSIVGSSPEALVTVHEGWATTHPIAGTRWRGRTDDEDVLLEKELLADDKERAEHLMLVDLGRNDLGRVCTPGTVRVEDYSHIERYSHVMHLVSTVTGKLGEGRTALDAVTACFPAGTLSGAPKVRAMELIEEVEKTRRGLYGGVVGYLDFAGNADFAIAIRTALMRNGTAYVQAGGGVVADSNGSYEYNEARNKARAVLNAIAAAETLAAPGANRSGC

>76|CORE_REP|Org90_Gene2789#

MTTPSHAPAVDLATAKDAVVQHLSRLFEFTTGPQGGPARLGFAGAVLITAGGLGAGSVRQHDPLLESIHMSWLRFGHGLVLSSILLWTGVGVMLLAWLGLGRRVLAGEATEFTMRATTVIWLAPLLLSVPVFSRDTYSYLAQGALLRDGLDPYAVGPVGNPNALLDDVSPIWTITTAPYGPAFILVAKFVTVIVGNNVVAGTMLLRLCMLPGLALLVWATPRLASHLGTHGPTALWICVLNPLVLIHLMGGVHNEMLMVGLMTAGIALTVQGRNVAGIILITVAIAVKATAGIALPFLVWVWLRHLRERRGYRPVQAFLAAAAISLLIFVAVFAVLSAVAGVGLGWLTALAGSVKIINWLTVPTGAANVIHALGRGLFTVDFYTLLRITRLIGIVIIAVSLPLLWWRFRRDDRAALTGVAWSMLIVVLFVPAALPWYYSWPLAVAAPLAQARRAIAAIAGLSTWVMVIFKPDGSHGMYSWLHFWIATACALTAWYVLYRSPDRRGVQAATPVVNTP

>77|CORE_REP|Org33_Gene434#

MLTRAIKTQLVLLTVLAVIAVVVLGWYFLRIPSLVGIGRYTLYAELPRSGGLYRTANVTYRGITIGKVTGVEPTERGARATMSIDNGYQIPTDASANVHSVSAVGEQFVDLVSTRTSGPYLRHGQTITTTTVPSQIGPALDAANRGLAVLPKDRVASVLHEASEAVGGLGSSLNRLIEATQAIAHDVRGSLEDIDDIIERSAPIIDSQVNSGNEIARWAANLNTLAAQTAQTDPAVRSILANAAPTADQVNATFSDVRESLPQTLANLEVVIDMLKRYHNGVEQALVFLPQSGAIAQSVTTEFPGQAGLGVGGLALNQPPPCLTGFLPASEWRSPADTSTAPLPKGTYCRIPMDASNVVRGARNNPCVDVPGKRAATPRECRSNEAYVPGGTNPWYGDPNQMLSCPAPAARCDQPVKPGQVIPAPSVNNGINPLPADQLPGTPPPVNDPLQRPGSGTVQCNGQQPNPCVYTPSTFPTTIYDVQSGKVVAPDGVVYSVEASTHAGADGWKVMLAPTG

>78|CORE_REP|Org11_Gene3577#

MRTLEPPNRMRIGLMGIVVALLVVAVGQSFTSVPMLFAKPSYYGQFTDSGGLHKGDRVRIAGLGVGTVEGLKIDGDHIVVKFSIGTNTIGTESRLAIRTDTILGRKVLEIEPRGAQALPPGGVLPVGQSTTPYQIYDAFFDVTKAASGWDIETVKRSLNVLSETVDQTYPHLSAALDGVAKFSDTIGKRDEQITHLLAQANQVASILGDRSEQVDRLLVNAKTLIAAFNERGRAVDALLGNISAFSAQVQNLINDNPNLNHVLEQLRILTDLLVDRKEDLAETLTILGRFSASFGETFASGPYFKVLLANLVPGQILQPFVDAAFKKRGISPEDFWRSAGLPAYRWPDPNGTRFPNGAPPPPPPVLEGTPEHPGPAVPPGSPCSYTPPADGLPRPWDPLPCANLTQGPFGGPDFPAPLDVATSPPNPDGPPPAPGLPIAGRPGEVPPNVPGTPVPIPQEAPPGARTLPLGPAPANTGSAPGGASPPAPPGPGPQLPAPFINPGGTGGSGVTGGSEN

>79|CORE_REP|Org78_Gene1546#

MEQHTLLQREESPRSPAAPSLRRLGGSRHITHWDPEDLGAWEAGNKGIARRNLLWSVVTVHLGYSVWTLWPVLELLMPQDVYGFSTSDKFLLGTIATLFGAFLRMPYALASAIFGGRNWATFSAIVLLIPAIGTTVLLTHPGLPLWPYLVCAALTGLGGGNFASSMSNANAFYPHRLKGSALGIAGGVGNLGVPAIQLVGLLAIATVGERKPYLVCALYVVLVAIAVIGVSLFMNNVEQHRVQVNRLRPIVSAVLSTRDTWLLSLLYLGTFGSFIGFSFVFGQVLQTNFLACGQSPARATLHAVELAFVGPLLAAVARIYGGRLADRVGGSRLTLIVFVAMTLAAGLLISASTLEGRHVGQHRGATMVGYFVCFVALFVLSGLGNGSVYKMIPTIFEACSRSLDLSEAERRDWSRIISGVVIGFVAAFGALGGVGINMALRESYLSTGSGTDAFWIFMMCYAAAAVLTWKVYDRRTVTDMGMLQAALVRQPASTPAELIGPRTQSDRFSGCSISA

>80|CORE_REP|Org2_Gene1185#

MTFPGDTAVLVLAAGPGTRMRSDTPKVLHTLAGRSMLSHVLHAIAKLAPQRLIVVLGHDHQRIAPLVGELADTLGRTIDVALQDRPLGTGHAVLCGLSALPDDYAGNVVVTSGDTPLLDADTLADLIATHRAVSAAVTVLTTTLDDPFGYGRILRTQDHEVMAIVEQTDATPSQREIREVNAGVYAFDIAALRSALSRLSSNNAQQELYLTDVIAILRSDGQTVHASHVDDSALVAGVNNRVQLAELASELNRRVVAAHQLAGVTVVDPATTWIDVDVTIGRDTVIHPGTQLLGRTQIGGRCVVGPDTTLTDVAVGDGASVVRTHGSSSSIGDGAAVGPFTYLRPGTALGADGKLGAFVEVKNSTIGTGTKVPHLTYVGDADIGEYSNIGASSVFVNYDGTSKRRTTVGSHVRTGSDTMFVAPVTIGDGAYTGAGTVVREDVPPGRWQCRRVRNATSRTGCSANAPAAQRLRPQKEPQKWPANSPHNHPTLIRHREVASRLPGDKSGRCATYHG

>81|CORE_REP|Org1_Gene4199#

MHLMRTISPFLRCRHETCCISNVGEEVTRTTYSREHQREYRRKVRLCLDVFETMLAQTRFEADRPLTGMEIECNLVDADYQPAMSNRYVLDAIADPAYQTELGAYNIEFNVPPRPLPGRTCLELEDEVRASLNDAETKASCSGAHIVMIGILPTLMPEHLTDGWMSASARYAALNESIFKARGEDIPINIAGPEPLSCHAGSIAPESACTSVQLHLQLAPADFPANWNAAQVLAGPQLALGANSPYFFGHQLWSETRIELFTQSTDARPEELKSRGVRPRVWFGERWITSVLDLFQENIRYFPTLLPEVSDEDPLAELSAGRIPHLSELRLHNGTVYRWNRPVYDVVDGRPHLRLENRVLPAGPTVVDMLANHAFYYGALRGLSEADPPLWTQMNFAAAQANFLAAARYGMDAQLDWPGLGEVTTRELVLGTLLPMAHEGLRRWGVDAEVRDRFLGVIGGRAQTGRNGARWQVATVAALQDGGLTRPAALAEMLRRYCEHMHSNEPVHTWDT

>82|CORE_REP|Org67_Gene4027#

MRIGPVELSAVKDWDPAPGVLVSWHPTPASCAKAFAAPVSAVPPSYVQARQIRSFSEQAARGLDHSRLLIASVEVFGHCDLRAMTYVINAHVRRHDTYRSWFELRDTDHIVRHSIADPADIEFVPTTHGEMTSADLRQHIVATPDSLHWDCFSFGVIQRADSFTFYASIDHLHADGQFVGVGLMEFQSMYTALIMGEPPIGLSEAGSYVDFCVRQHEYTSALTVDSPEVRAWIDFAEINNGTFPEFPLPLGDPSVRCGGDLLSMMLMDEQQTQRFESACMAANARFIGGMLACIAIAIHELTGADTYFGITPKDIRTPADLMTQGWFTGQIPVTVPVAGLSFNEIARIAQTSFDTGADLAKVPFERVVELSPSLRRPQPLFSLVNFFDAQVGPLSAVTKLFEGLNVGTYSDGRVTYPLSTMVGRFDETAASVLFPDNPVARESVTAYLRAIRSVCMRIANGGTAERVGNVVALSPGRRNNIERMTWRSCRAGDFIDICNLKVANVTVDREA

>83|CORE_REP|Org1_Gene1761#

MADAARARRLAKRIAAIVASAIEYEIKDPGLAGVTITDAKVTADLHDATVYYTVMGRTLHDEPNCAGAAAALERAKGVLRTKVGAGTGVRFTPTLTFTLDTISDSVHRMDELLARARAADADLARVRVGAKPAGEADPYRDNGSVAQSPAPGGLGIRTSDGPEAVEAPLTCGETPVTTIDPRNELVDGRRRAGARVDAVGAAALLSAAARVGVVCHVHPDADTIGAGLALALVLDGCGKRVEVSFAAPATLPESLRSLPGCHLLVRPEVMRRDVDLVVTVDIPSVDRLGALGDLTDSGRELLVIDHHASNDLFGTANFIDPSADSTTTMVAEILDAWGKPIDPRVAHCIYAGLATDTGSFRWASVRGYRLAARLVEIGVDNATVSRTLMDSHPFTWLPLLSRVLGSAQLVSEAVGGRGLVYVVVDNREWVAARSEEVESIVDIVRTTQQAEVAAVFKEVEPHRWSVSMRAKTVNLAAVASGFGGGGHRLAAGYTTTGSIDDAVASLRAALG

>84|CORE_REP|Org78_Gene3169#

MINDLRTVPAALDRLVRQLPDHTALIAEDRRFTSTELRDAVYGAAAALIALGVEPADRVAIWSPNTWHWVVACLAIHHAGAAVVPLNTRYTATEATDILDRAGAPVLFAAGLFLGADRAAGLDRAALPALRHVVRVPVEADDGTWDEFIATGAGALDAVAARAAAVAPQDVSDILFTSGTTGRSKGVLCAHRQSLSASASWAANGKITSDDRYLCINPFFHNFGYKAGILACLQTGATLIPHVTFDPLHALRAIERHRITVLPGPPTIYQSLLDHPARKDFDLSSLRFAVTGAATVPVVLVERMQSELDIDIVLTAYGLTEANGMGTMCRPEDDAVTVATTCGRPFADFELRIADDGEVLLRGPNVMVGYLDDTEATAAAIDADGWLHTGDIGAVDQAGNLRITDRLKDMYICGGFNVYPAEVEQVLARMDGVADAAVIGVPDQRLGEVGRAFVVARPGTGLDEASVIAYTREHLANFKTPRSVRFVDVLPRNAAGKVSKPQLRELG

>85|CORE_REP|Org118_Gene3348#

MPDKRTALDDAVAQLRSGMTIGIAGWGSRRKPMAFVRAILRSDVTDLTVVTYGGPDLGLLCSAGKVKRVYYGFVSLDSPPFYDPWFAHARTSGAIEAREMDEGMLRCGLQAAAQRLPFLPIRAGLGSSVPQFWAGELQTVTSPYPAPGGGYETLIAMPALRLDAAFAHLNLGDSHGNAAYTGIDPYFDDLFLMAAEAALSVGGAHRRHRGTGQIGAAAGAVGQPDDGRRHRGSTRRRPLHHRRTGLRARRAVPAALRRSGVDTGGLAAVRAHLPIRHRSGLPGRGAQLWSITVSTRAEVCAVACAELFRDAGEIMISPMTNMASVGARLARLTFAPDILLTDGEAQLLADTPALGKTGAPNRIEGWMPFGRVFETLAWGRRHVVMGANQVDRYGNQNISAFGPLQRPTRQMFGVRGSPGNTINHATSYWVGNHCKRVFVEAVDVVSGIGYDKVDPDNPAFRFVNVYRVVSNLGVFDFGGPDHSMRAVSLHPGVTPGDVRDATSFRGA

>86|CORE_REP|Org118_Gene1678#

MAEESRGQRGSGYGLGLSTRTQVTGYQFLARRTAMALTRWRVRMEIEPGRRQTLAVVASVSAALVICLGALLWSFISPSGQLNESPIIADRDSGALYVRVGDRLYPALNLASARLITGRPDNPHLVRSSQIATMPRGPLVGIPGAPSSFSPKSPPASSWLVLGDTVATSSSIGSLQGVTVTVIDGTPDLTGHRQILSGSDAVVLRYGGDAWVIREGRRSRIEPTNRAVLLPLGLTPEQVSQARPMSRALFDALPVGPELLVPEVPNAGGPATFPGAPGPIGTVIVTPQISGPQQYSLVLGDGVQTLPPLVAQILQNAGSAGNTKPLTVEPSTLAKMPVVNRLDLSAYPDNPLEVVDIREHPSTCWWWERTAGENRARVRVVSGPTIPVAATEMNKVVSLVKADTSGRQADQVYFGPDHANFVAVTGNNPGAQTSESLWWVTDAGARFGVEDSKEARDALGLTLTPSLAPWVALRLLPQGPTLSRADALVEHDTLPMDMTPAELVVPK

>87|CORE_REP|Org1_Gene1027#

MKPAQGPDFEGSQGAVKRTPRVITRPSAQRLTLRAAAHGARDPTPGRRVIPIATTFSYKHLVGQPYQPKREGVLVADTDDTATLRYPGGEIDLQIVHATEGADGIALGPLLAKTGHTTFDVGFANTAAAKSSITYIDGDAGILRYRGYPIDQLAEKSTFIEVCYLLIYGELPDTDQLAQFTGRIQRHTMLHEDLKRFFDGFPRNAHPMPVLSSVVNALSAYYQDALDPMDNGQVELSTIRLLAKLPTIAAYAYKKSVGQPFLYPDNSLTLVENFLRLTFGFPAEPYQADPEVVRALDMLFILHADHEQNCSTSTVRLVGSSRANLFTSISGGINALWGPLHGGANQAVLEMLEGIRDSGDDVSEFVRKVKNREAGVKLMGFGHRVYKNYDPRARIVKEQADKILAKLGGDDSLLGIAKELEEAALTDDYFIERKLYPNVDFYTGLIYRALGFPTRMFTVLFALGRLPGWIAHWREMHDEGDSKIGRPRQIYTGYTERDYVTIDAR

>88|CORE_REP|Org119_Gene1647#

MRALPAGRHFFRGSDGYEAARRGTVWHRRVPDRYPEVIVQAVSADDIVSAIRYATVNGHKVSVVSGGHSFAASHLRDGAVLLDVSRIDHASIDADKGRAVVGPGKGGSVLMAELEAQGLFFPGGHCRGVCLGGYLLQGGYGWNSRIYGPACESVIGLDVITADGAQIHCDADNHADLYWAARGAGPGFFGVVTSFYLKLYPRPATCGTSVYVYPFDLADEVFTWARAVSAEVDPRVELQALASRGEPSMGIDVPVISLASPAFADSPEEAEQALALFGTCPVVEQALVKVPYMPTDLPAWYDVAMTHYLSDHHYAVDNMWTSASAEDLLPGIRSILDTLPPHPAHFLWLNWGPCPPRQDMAYSIEADIYLALYGSWKDPADEAKYADWARSHMAAMSHLAVGIQLADENLGARPARFASDAAMAKLDRVRAEYETPTVCSTVGWEESDGQRSVARAPPQRRRGHAVRQVLQTRDGPAATACRGGVAAWPPGRDGVARLRRRREHR

>89|CORE_REP|Org29_Gene3386#

MSHLVTAPDMLATAAAHVDEIASTLRAANAAAAGPTCNLLAAAGDEVSAATAALFSAYGREYQAVVKQAAAFHSEFTRTLEAAGNAYAHAEAANAARVSHALDTINAPIRTLLGRAPLSPNGSSGAGGLPAIAQLAAESPITALIMGGTNNPLPDPEYVTDINKAFIQTLFPGAVSQGLFTPEQFWPVTPDLGNLTFNQSVTEGVALLNTAVNNQLALDNKVVAFGYSQSATIINNYINSLMAMGSPNPDDISFVMIGSGNNPVGGLLARFPGFYIPFLDVPFNGATPANSPYPTHIYTAQYDGIAHAPQFPLRILSDINAFMGYFYVHNTYPELMATQVDNAVPLPTSPGYTGNTQYYMFLTQDLPLLQPIRDIPYAGPPIADLFQPQLRVLVDLGYADYGPGGNYADIPTPAGLFSIPNPFAVTYYLIKGSLQAPYGAIVEIGVEAGLIGPEWFPDSYPWVPSINPGLNFYFGQPQVTLLSLMSGGLGNILHLIPPPVFT

>90|CORE_REP|Org144_Gene2091#

MSTLGDLLAEHTVLPGSAVDHLHAVVGEWQLLADLSFADYLMWVRRDDGVLVCVAQCRPNTGPTVVHTDAVGTVVAANSMPLVAATFSGGVPGREGAVGQQNSCQHDGHSVEVSPVRFGDQVVAVLTRHQPELAARRRSGHLETAYRLCATDLLRMLAEGTFPDAGDVAMSRSSPRAGDGFIRLDVDGVVSYASPNALSAYHRMGLTTELEGVNLIDATRPLISDPFEAHEVDEHVQDLLAGDGKGMRMEVDAGGATVLLRTLPLVVAGRNVGAAILIRDVTEVKRRDRALISKDATIREIHHRVKNNLQTVAALLRLQARRTSNAEGREALIESVRRVSSIALVHDALSMSVDEQVNLDEVIDRILPIMNDVASVDRPIRINRVGDLGVLDSDRATALIMVITELVQNAIEHAFDPAAAEGSVTIRAERSARWLDVVVHDDGLGLPQGFSLEKSDSLGLQIVRTLVSAELDGSLGMRDARERGTDVVLRVPVGRRGRLML

>92|CORE_REP|Org1_Gene2915#

MSFVVTIPEALAAVATDLAGIGSTIGTANAAAAVPTTTVLAAAADEVSAAMAALFSGHAQAYQALSAQAALFHEQFVRALTAGAGSYAAAEAASAAPLEGVLDVINAPALALLGRPLIGNGANGAPGTGANGGDGGILIGNGGAGGSGAAGMPGGNGGAAGLFGNGGPAAPGERSVRHRRVRRGRRAGGLLYGAGGAGGAGGRAGGGVGGIGGAGGAGGNGGLLFGAGGPAASADSRPTPVTAGRRRRRVVLRRGRCRRAGGTGTNVTGGAGGAGGNGGLLFGAGGVGGVGGDGVAFLGTAPAGPVVPAGPVGCSASVGPAAPAESDWSGTAVPGVRRVRPALGRRRCRRRGWVGSTTGGAGGAGGNAGLLVGAGGAGGAGALGGGATGVGGAGGNGGTAGLLFGAGGAGGAGGFGFGGAGGAGGLGGKAGLIGDGGDGGAGGNGTGAKGGDGGAGGGAILVGNGGNGGNAGSGTPNGSAGTGGAGGLLGKNGMNGLP

>93|CORE_REP|Org85_Gene4018#

MSQTARRLGPQDMFFLYSESSTTMMHVGALMPFTPPSGAPPDLLRQLVDESKASEVVEPWSLRLSHPELLYHPTQSWVVDDNFDLDYHVRRSALASPGDERELGIPVSRLHSHALDLRRPPWEVHFIEGLEGGRFAIYIKMHHSLIDGYTGQKMLARSLSTDPHDTTHPLFFNIPTPGRSPADTQDSVGGGLIAGAGNVLDGLGDVVRGLGGLVSGVGSVLGSVAGAGRSTFELTKALVNAQLRSDHEYRNLVGSVQAPHCILNTRISRNRRFATQQYPLDRLKAIGAQYDATINDVALAIIGGGLRRFLDELGELPNKSLIVVLPVNVRPKDDEGGGNAVATILATLGTDVADPVQRLAAVTASTRAAKAQLRSMDKDAILAYSAALMAPYGVQLASTLSGVKPPWPYTFNLCVSNVPGPEDVLYXRGSRMEASYPVSLVAHSQALNVTLQSYAGTLNFGFIGCRDTLPHLQRLAVYTGEALDQLAAADGAAGLGS

>94|CORE_REP|Org59_Gene728#

MARHLRGRLPLRVRLVAATLILVATGLVASGIAVTSMLQHRLTSRIDRVLLEEAQIWAQITLPLAPDPYPGHNPDRPPSRFYVRVISPDGQSYTALNDNTAIPAVPANNDVGRHPTTLPSIGGSKTLWRAVSVRASDGYLTTVAIDLADVRSTVRSLVLLQVGIGSAVLVVLGVAGYAVVRRSLRPLAEFEQTAAAIGAGQLDRRVPQWHPRTEVGRLSLALNGMLAQIQRAVASAESSAEKARDSEDRMRQFITDASHELRTPLTTIRGFAELYRQGAARDVGMLLSRIESEASRMGLLVDDLLLLARLDAXRPLELCRVDLLALASDAAHDARAMDPKRRITLEVLDGPGTPEVLGDESRLRQVLRNLVANAIQHTPESADVTVRVGTEGDDAILEVADDGPGMSQEDALRVFERFYRADSSRARASGGTGLGLSIVDSLVAAHGGAVTVTTALGEGCCFRVSLPRVSDRGPAEPHASCARAALILACAIVQRSR

>95|CORE_REP|Org58_Gene3922#

MAREISRQTFLRGAAGALAAGAVFGSVRATADPAASGWEALSSALGGKVAGDLASPQSVGQPDDGPQFATAKQVFNTNYNGYTPAVIVTPTSQLDVQKAMAFAAANNLKVAPRGGGHSYVGASTANGAMVLDLRQLPGDINYDATTGRVTVTPATGLYAMHQVLAAAGRGIPTGTCPTVGVAGHALGGGLGANSRHAGLLCDQLTSASVVLPSGQAVTASATDHPDLFWALRGGGGGNFGVTTSLTFATFPSGDLDVVNLNFPPQSFAQVLVGWQNWLRTADRGSWALADATVDPLGTHCRILATCPAGSGGSVAAAIVSAVGTQPTGTENHTFNYLDLVRYLAVGNLNPSPLGYVGGSDVFTTITPATAQGIASAVDAFPRGAGRMLAIMHALDGALATVSPGATAFPWRRQSALVQWYVETSGSPSEATSWLNTAHQAVRAYSVGGYVNYLEVNQPPARYFGPNLSRLSAVRQKYDPSRVHVLRAELLAAPHEY

>96|CORE_REP|Org59_Gene359#

MKIANEFTVSAPIEQAWSRLCDLEQMIPLMPGAQLIGHEGDEYLGKVKVKVGPVTSEFSGKVHFVEQDRNQHRAVFDAKGKEARGTGNAAATVAAQLHEVGERTRVTVDTDLKIVGKLAQFGSGMLQQVSEKLLGQFVDSLEAELAAQSSESPQGTPPATEAAPIDLLQLADGGQLKKYGSALTGGADRAAADLGAAPAAMNGMATPALLPGVDLAAFAAALAARLRDAGIPVSAXRXSXFXXGVAAVGAAYAGGAVLGRAVDPGQPCRRTGHVRCGIRFAVRGIWQRRTRRCQPPTTAHCRPAHTGGRRRAPRQAAILCRPSPESALGYSLADDGQRRSGRTQPHTARCPAQPHCRPGRRAIRPVRSRRSASARRLAGGHDGALAAAAQHAIRVQPARQAHRPAGDDERVAVDWLGVGAVGTDPAPPTPQAGAPALRCEPLDAALRRHLSASDAGGGAAPGRGPPGGFRVFDVADSTYLGAVSSLGRDGATSGQR

>97|CORE_REP|Org66_Gene1864#

MSDLARTDVVLIGAGIMSATLGVLLRRLEPNWSITLIERLDAVAAESSGPWNNAGTGHSALCEMNYTPEMPDGSIDITKAVRVNEQFQVTRQFWAYAAENGILTDVRSFLNPVPHVSFVHGSRGVEYLRRRQKALAGNPLFAGTEFIESPDEFARRLPFMAAKRAFSEPVALNWAADGTDVDFGALAKQLIGYCVQNGTTALFGHEVRNLSRQSDGSWTVTMCNRRTGEKRKLNTKFVFVGAGGDTLPVLQKSGIKEVKGFAGFPIGGRFLRAGNPALTASHRAKVYGFPAPGAPPLGALHLDLRFVNGKSWLVFGPYAGWSPKFLKHGQISDLPRSIRPDNLLSVLGVGLTERRLLNYLISQLRLSEPERVSALREFAPSAIDSDWELTIAGQRVQVIRRDERNGGVLEFGTTVIGDADGSIAGLLGGSPGASTAVAIMLDVLQKCFANRYQSWLPTLKEMVPSLGVQLSNEPALFDEVWSWSTKALKLGAA

>98|CORE_REP|Org54_Gene2940#

MVTRIVILGGGPAGYEAALVAATSHPETTQVTVIDCDGIGGAAVLDDCVPSKTFIASTGLRTELRRAPHLGFHIDFDDAKISLPQIHARVKTLAAAQSADITAQLLSMGVQVIAGRGELIDSTPGLARHRIKATAADGSTSEHEADVVLVATGASPRILPSAQPDGERILTWRQLYDLDALPDHLIVVGSGVTGAEFVDAYTELGVPVTVVASQDHVLPYEDADAALVLEESFAERGVRLFKNARAASVTRTGAGVLVTMTDGRTVEGSHALMTIGSVPNTSGLGLERVGIQLGRGNYLTVDRVSRTLATGIYAAGDCTGLLPLASVAAMQGRIAMYHALGEGVSPIRLRTVAATVFTRPEIAAVGVPQSVIDAGSVAARTIMLPLRTNARAKMSEMRHGFVKIFCRRSTGVVIGGVVVAPIASELILPIAVAVQNRITVNELAQTLAVYPSLSGSITEAARRLMAHDDLDCTAAQDAAEQLALVPHHLPTSN

>100|CORE_REP|Org9_Gene171#

MIRAASDDPAGVDELVAAIAPGLAGLGLPVINRREVVLVTGPWLAGVSGVRAALAERLPQRRFVETAELGPGDAPVAVVFVVSAATALTESDCVLLDTAAEHTDAVVAVVSKIDVHRGWRDVLTSNRDRLAARASRYARVPWVGAAAAPELGEPYLDDLVAAIQKQLADPAVARRNMLRAWESRLLMVARRFDGDAQSAGRRARVDALRQQRRTVLRQGRQSKSEHTIALRAQIQHARVKLSYFARNRCSLLRVELQEHVAGLSRKDIARFAAYTRGRVQEVVAEVGEGAVAHLADVAQLLGVPVQPPVLENLPAVLPTVVAPPLTSRRLEIRLTTLLGAGFGLGIALTLSRLVAGLTPGLAASGMVAGVAIGLAVTAWVVNARALLHDRVVVDRWTGEVTASLRSVVEQLVATRVVAVETLLSTAISERDDAENARVADQVSIIDGELREHAVAAARAAALRDREMPAVRAALEAVRAELGEPGTPTTGLF

>102|CORE_REP|Org85_Gene2669#

MSAASQRVGAFGEEAGYHKGLKPRQLQMIGIGGAIGTGLFLGAGGRLAKAGPGLFLVYGVCGVFVFLILRALGELVLHRPSSGSFVSYAREFFGEKAAYAVGWMYFLHWAMTSIVDTTAIATYLQRWTIFTVVPQWILALIALTVVLSMNLISVEWFGELEFWAALIKVLALMAFLVVGTVFLAGRYPVDGHSTGLSLWNNHGGLFPTSWLPLLIVTSGVVFAYSAVELVGTAAGETAEPEKIMPRAINSVVARIAIFYVGSVALLALLLPYTAYKAGESPFVTFFSKIGFHGAGDLMNIVVLTAALSSLNAGLYSTGRVMHSIAMSGSAPRFTARMSKSGVPYGGIVLTAVITLFGVALNAFKPGEAFEIVLNMSALGIIAGWATIVLCQLRLHKLANAGIMQRPRFRMPFSPYSGYLTLLFLLVVLVTMASDKPIGTWTVATLIIVIPALTAGWYLVRKRVMAVARERLGHTGPFPAVANPPVRSRD

>103|CORE_REP|Org103_Gene469#

MRAEIGPDFRPHYTFGDAYPASERAHVNWELSAPVWHTAQMGSTTHREVAKLDRVPLPVEAARVAATGWQVTRTAVRFIGRLPRKGPWQQKVIKELPQTFADLGPTYVKFGQIIASSPGAFGESLSREFRGLLDRVPPAKTDEVHKLFVEELGDEPARLFASFEEEPFASASIAQVHYATLRSGEEVVVKIQRPGIRRRVAADLQILKRFAQTVELAKLGRRLSAQDVVADFADNLAEELDFRLEAQSMEAWVSHLHASPLGKNIRVPQVHWDFTTERVLTMERVHGIRIDNAAAIRKAGFDGVELVKALLFSVFEGGLRHGLFHGDLHAGNLYVDEAGRIVFFDFGIMGRIDPRTRWLLRELVYALLVKKDHAAAGKIVVLMGAVGTMKPETQAAKDLERFATPLTMQSLGDMSYADIGRQLSALADAYDVKLPRELVLIGKQFLYVERYMKLLAPRWQMMSDPQLTGYFANFMVEVSREHQSDIEV

>104|CORE_REP|Org59_Gene1579#

MLQRIARELLXGVAXAIVALPLAIAFGITATGTSQGALIGLYGAIFAGFFAAVFGGTPGQVTGPTGPITVVATATIAEHGLEGAFFAFILAGVFQILFGACRLGSLIRYVPHPVISGFMGGIAILIIMTQLDQVRSSSLLVLVTVVLLLASGRFIKAIPPSLLVLVLVSSVLPLAAPWLRDLRAGPVSINRTVDYIGEIPQAMPSFDFPQVANSTMLQVLLSAXAIALLGSLDSLLTSLVMDNIRGTRHRSNKELIGQGIGNIAAGLFGGLSGAGATVRSVVNVRNGGQTALSAATHSVVLFVFVAGLGAVVQYIPLAVLSGILILVAVGMFDWHAMRKAHVSPRGDVIVMFTTMIITVVVDLTIAVMVGIALSLLVHRLRSRQRKAKVTQDDTGTYRIDGPLSFLSVDGVFGSLRDGREDVSLDLQHVTYLDTSGARALLYFIDHSEKDGVAVSIKRIPPRLESQLTALADNEQRDKLRTVLESA

>105|CORE_REP|Org62_Gene3304#

MSETFCLTDHSEPMTARFLSVVLRRIRGMRSDTREEISAALDAYHASLSRVLDLKCDALTTPELLACLQRLEVERRRQGAAEHALINQLAGQACEEELGGTLRTALANRLHITPGEASRRIAEAEDLGERRALTGEPLPAQLTATAAAQREGKIGREHIKEIQAFFKELSAAVDLGIREAAEAQLAELATSRRPDHLHGLATQLMDWLHPDGNFSDQERARKRGITMGKQEFDGMSRISGLLTPELRATIEAVLAKLAAPGACNPDDQTPLVDDTPDADAVRRDTRSQAQRNHDAFLAALRGLLASGELGQHKGLPVTIVVSTTLKELEAATGKGVTGGGSRVPMSDLIRMASHANHYLALFDGAKPLALYHTKRLASPAQRIMLYAKDRGCSRPGCDAPAYHSEVHHVTPWTTTHRTDINDLTLACGPDNRLVEKGWKTRKNAHGDTEWLPPPHLDHGQPRINRYHHPAKILCEQDDDEPH

>106|CORE_REP|Org59_Gene468#

MAGVRHDDGSGLIAQRRPVRGEGATRSRGPSGPSNRNVSAADDPRRVALLAVHTSPLAQPGTGDAGGMNVYMLQSALHLARRGIEVEIFTRATAXXRSTGGAGGTRGAGAZRGGGXXRGXGQVRPAHPALSRXAAGVLRAEAVHEPGYYDIVHSHYWLSGQVGWLARDRWAVPLVHTAHTLAAVKNAALADGDGPEPPLRTVGEQQVVDEADRLIVNTDDEARQVISLHGADPARIDVVHPGVDLDXFRPGDRRAARAALGLPVDERVVAFVGRIQPLKAPDIVLRAAAKLPGVRIIVAGGPSGSGLASPDGLVRLADELGISARVTFLPPQSHTDLATLFRAADLVAVPSYSESFGLVAVEAQACGTPVVAAAVGGLPVAVRDGITGTLVSGHEVGQWADAIDHLLRLCAGPRGRVMSRAAARHAATFSWENTTDALLASYRRAIGEYNAERQRRGGEVISDLVAVGKPRHWTPRRGVGA

>107|CORE_REP|Org13_Gene2210#

MDVTVVGSGPNGLATAVICARAGLNVQVVEAQATFGGGARSAADFEFPEVLHDVCSAVHPLALASPFFAEFDLPARGVTLTVPDIAYANPLPGRPAAIAYHDLAHTCAKLDDGASWRRLLGPLVAHSETVVEFMLSDKRSLPTALGSVLRLGLRMLAQGTPAWRSLAGEDARALFTGVAAHAISPLPSLVSAGAGLMLATLAHSVGWPIPVGGTQAIADALIADLRAHGGRLAAGVEITEPQRSVVVFDTAPTALLRVYRDKLPHRYAKALRRYRFRAGIAKVDFVLSDEIPWSDPRLRRAATLHLGGTRDQMARAEADVAAGRHADWPMVLAACPHVADPGRIDETGRRPFWTYAHVPSGSTLDATETVTSVLERFAPGFRDIVVAARAVPAARMADHNANYVGGDITVGANSTWRAIAGPTPRLNPWRTPIPKVYLCSAATPPGAGVHGMCGWYAARTLLRTEFGITRMPPLGHELRP

>108|CORE_REP|Org83_Gene1572#

MAARRIRAARPLAPHGLPGHLVGFVEALRGSGISVGPSETVDAGRVMATLGLGDREVLREGIACAVLRRPDHRDTYDAMFDLWFPAALGARAVITTEDESAGSGGLPPDDVEAMRQLLLDLLANNQDLAGKDERLVEMIARIVEAYGKYSSSRGPSFSSYQALKAMALDELEGKLLAGLLAPYGDEPTATQEQIAKALAAQKIAQLRRMVDAETKRRTAEQLGREHVQMYGIPQLSENVEFLRASGEQLRQMRRVVAPLARTLATRLAARRRRARAGSIDLRKTLRKSMSTGGVPIDLVLHKPRPARPELVVLCDVSGSVAGFSHFTLLLVHALRQQFSRVRVFAFIDSTDEVTHMFGPESDLAIAIQRITREAGVYARDGHSDYGNAFVSFMQGFPNVLSPRSSLLVLGDGRTNYRNPATDVLADMVTASRHAHWLNPEPKHLWGSGDSAVPRYQEVITMHECRSAKQLATVIDQLLPV

>109|CORE_REP|Org1_Gene3828#

MISAMTDADSAVPPRLDEDAISKLELTEVADLIRTRQLTSAEVTESTLRRIERLDPQLKSYAFVMPETALAAARAADADIACGHYEGVLHGVPIGVKDLCYTVDAPTAAGTTIFRDFRPAYDATVVARLRAAGAVIIGKLAMTEGAYLGYHPSLPTPVNPWDPTAWAGVSSSGCGVATAAGLCFGSIGSDTGGSIRFPTSMCGVTGIKPTWGRVSRHGVVELAASYDHVGPITRSAHDAAVLLSVIAGSDIHDPSCSAEPVPDYAADLALTRIPRVGVDWSQTTSFDEDTTAMLADVVKTLDDIGWPVIDVKLPALAPMVAAFGKMRAVETAIAHADTYPARADEYGPIMRAMIDAGHRLAAVEYQTLTERRLEFTRSLRRVFHDVDILLMPSAGIASPTLETMRGLGQDPELTARLAMPTAPFNVSGNPAICLPAGTTARGTPLGVQFIGREFDEHLLVRAGHAFQQVTGYHRRRPPV

>110|CORE_REP|Org67_Gene510#

MTAAVRHSDVLVVGAGSAGSVVAERLSMDSSCVVTVLEAGPGLADPGLLAQTANGLQLPIGAGSPLVERYRTRLTDRPVRHLPIVRGATVGGSGAINGGYFCRGLPSDFDRASIPGWAWSDVLEHFRAIETDLDFETPVHGRSGPIPVRRTHEMTGITESFMAAAEDAGFAWIADLNDVGPEMPSGVGAVPLNIVNGVRTSSAVGYLMPALGRPNLTLLARTRAVRLRFSATTAVGVDAIGPGGPVSLSADRIVLCAGAIQSAHLLMLSGVGEEEVLRSAGVKVLMALPVGMGCSDHPEWVMPTNWAVAVDRPVLEVLLSTHDGIEIRPYTGGFVAMTGDGTAGHRDWPHIGVALMQPRARGRITLVSSDPQIPVRIEHRYDSEPADVAALRQGSALAHELCGAATRIGPAVWATSQHLCGSAPMGTDDDPRAVVDPRCRVRGIENLWVIDGSVLPSITSRGPHATIVMLGHRAAEFVQ

>112|CORE_REP|Org53_Gene949#

MGNLDLLLRLSGRIVKGCRPLGSVALARCGPAVRWPWWPRPAILEHMFDLVSLAGVDSRDDEASLTARIAELERVKSAAAAGQARAAAALDKLRRCNEADAGVPARRRGRGVASEVALARRDSPARGGRHLGFAKALVYEMPHTLAALEVGRLSEWRATLIVRESACLDVEDRRALDAELCADMSALDGMGDARIAAAARAIAYRLDAQAVVERAARAETERTVTIRPAPDTMTWVTALLPVARGVSVYAALKRAADTTFDDRTRGQVMADTLVERVTGQPAEAAQPVAVNLVLSDETLLAGDRAPAVVDGYGPIPAAVARNLVRDAVADTRSRATLRRLYRHPRSGALVAMESRARRFPKGLAAFIGLRDQRCRMPYCDAPIRHRDHAQPHHRGGPTTATNGLGSCERCNYVKEAPGWRVSTDTDETGRHTAEFTTPTGMYYHCTAPPLPGPLEIDVSQVEARIGVALTHLHAA

>113|CORE_REP|Org6_Gene2737#

MTGRVGNPKDHAVVIGASIAGLCAARVLSDFYSTVTVFERDELPEAPANRATVPQDRHLHMLMARGAQEFDSLFPGLLHDMVAAGVPMLENRPDCIYLGAAGHVLGTGHTLRKEFTAYVPSRPHLEWQLRRRVLQLSNVQIVRRLVTEPQFERRQQRVVGVLLDSPGSGQDREREEFIAADLVVDAAGRGTRLPVWLTQWGYRRPAEDTVDIGISYASHQFRIPDGLIAEKVVVAGASHDQSLGLGMLCYEDGTWVLTTFGVADAKPPPTFDEMRALADKLLPARFTAALAQAQPIGCPAFHAFPASRWRRYDKLERFPRGIVPFGDAVASFNPTFGQGMTMTSLQAGHLRRALKARNSAMKGDLAAELNRATAKTTYPVWMMNAIGDISFHHATAEPLPRWWRPAGSLFDQFLGAAETDPVLAEWFLRRFSLLDSLYMVPSVPIIGRAIAHNLRLWLKEQRERRQPVTTRRSP

>114|CORE_REP|Org28_Gene2952#

MALQTGEPRTLAEKIWDDHIVVSGGGCAPDLIYIDLHLVHEVTSPQAFDGLRLAGRRVRRPELTLATEDHNVPTVDIDQPIADPVSRTQVETLRRNCAEFGIRLHSMGDIEQGIVHVVGPQLGLTQPGMTIVCGDSHTSTHGAFGALAMGIGTSEVEHVLATQTLPLRPFKTMAVNVDGRLPDGVSAKDIILALIAKIGTGGGQGHVIEYRGSAIESLSMEGRMTICNMSIEAGARAGMVAPDETTYAFLRGRPHAPTGAQWDTALVYWQRLRTDVGAVFDTEVYLDAASLSPFVTWGTNPGQGVPLAAAVPDPQLMTDDAERQAAEKALAYMDLRPGTAMRDIAVDAVFVGSCTNGRIEDLRVVAEVLRGRKVADGVRMLIVPGSMRVRAQAEAEGLGEIFTDAGAQWRQAGCSMCLGMNPDQLASGERCAATSNRNFEGRQGAGGRTHLVSPAVAAATAVRGTLSSPADLN

>115|CORE_REP|Org18_Gene1009#

MRRNRRGSPARPAARFVRPAIPSALSVALLVCTPGLATADPQTDTIAALIADVAKANQRLQDLSDEVQAEQESVNKAMVDVETARDNAAAAEDDLEVSQRAVKDANAAIAAAQHRFDTFAAATYMNGPSVSYLSASSPDEIIATVTAAKTLSASSQAVMANLQRARTERVNTESAARLAKQKADKAAADAKASQDAAVAALTETRRKFDEQREEVQRLAAERDAAQARLQAARLVAWSSEGGQGAPPFRMWDPGSGPAGGRAWDGLWDPTLPMIPSANIPGDPIAVVNQVLGISATSAQVTANMGRKFLEQLGILQPTDTGITNAPAGSAQGRIPRVYGRQASEYVIRRGMSQIGVPYSWGGGNAAGPSKGIDSGAGTVGFDCSGLVLYSFAGVGIKLPHYSGSQYNLGRKIPSSQMRRGDVIFYGPNGSQHVTIYLGNGQMLEAPDVGLKVRVAPVRTAGMTPYVVRYIEY

>116|CORE_REP|Org17_Gene504#

MTATRLPDGFAVQVDRRVRVLGDGSALLGGSPTRLLRLAPAARGLLCDGRLKVRDEVSAELARILLDATVAHPRPPSGPSHRDVTVVIPVRNNASGLRRLVTSLRGLRVIVVDDGSACPVESDDFVGAHCDIEVLHHPHSKGPAAARNTGLAACTTDFVAFLDSDVTPRRGWLESLLGHFCDPTVALVAPRIVSLVEGENPVARYEALHSSLDLGQREAPVLPHSTVSYVPSAAIVCRSSAIRDVGGFDETMHSGEDVDLCWRLIEAGARLRYEPIALVAHDHRTQLRDWIARKAFYGGSAAPLAVRHPDKTAPLVISGGALMAWILMSIGTGLGRLASLVIAVLTGRRIARAMRCAETSFLDVLAVATRGLWAAALQLASAICRHYWPLALLAAILSRRCRRVVLIAAVVDGVVDWLRRREGADDDAEPIGPLTYLVLKRVDDLAYGAGLWYGVVRERNIGALKPQIRT

>117|CORE_REP|Org68_Gene2390#

MQGQLSRTRVYAVPVPGSAQSAYACGVERLLASYRSIPATASIRLAKPTSNLFRARVKHDARGLDASGLTGVIGIDPEARTADVAGMCTYEDLIAATLHYGLSPLVVPQLRTITLGGAVTGLGIESASFRNGLPHESVLEMDILTGAGELLTVSPGQHSDLYRAFPNSYGTLGYSTRLRIQLEPVRPFVALRHIRFSSLTAMVAAMERIIDTGGLDGESVDYLDGVVFSADESYLCIGMQTSVPGPVSDYTGQDIYYRSIQHEAGIKEDRLTIHDYFWRWDTDWFWCSRSFGAQNPRLRRWWPRRYRRSSVYWRLMALDQRFGIADRFENSRGRPARERVVQDIEVPIERTCEFLEWFGENVPISPIWLCPLRLRDHAGWPLYPIRPDRSYVNIGFWSSVPVGATEGATNRKIENKVSALDGHKSLYSDSFYTREEFDELYGGETYNTVKKAYDPDSRLLDLYAKAVQRR

>118|CORE_REP|Org128_Gene1845#

MAVGDDEEKVRAERARAIGLFRYQLIWEAADAAHSTKQRGKMVRELASREHTDPFGRRVRISRQTIDRWIRGWRAGGFDALVPNPRQCTPRTPAEVLELAVALRRENPQRTAAAIRRILRTQLGWAPDERTLQRNFHRLGLTGATTGSAPAVFGRFEAEHPNALWTGDVLHGIRIDLRKTYLFAFLDDHSRLVPGYRWGHAEDTVRLAAALRPALASRGVPNAVYVDNGSPYVDAWLLRACAKLGVRLVHSTPGRPQGRGKIERFFRTVREQFLVEITGEPDVVGRHYVADLAELNRLFTAWVETVYHRSVHSETGQTPLARWSAGGPIPLPAPETLTEAFLWEEHRRVTKTATVSLHGNRYEIDPALVGRKVELVFDPFDLTRIEVRLAGAPMGRAIPYHIGRHSHPKAKPETPTAPPKPSGIDYAQLIETAHAAELARGVNYTALTGAADQIPGQLDLLTGQEAQPK

>119|CORE_REP|Org59_Gene321#

MASTLTTGLPPGPRLPRYLQSVLYLRFREWFLPAMHRKYGDVFSLRVPPYADNLVVYTRPEHIKEIFAADPRSLHAGEGNHILGFVMGEHSVLMTDEAEHARMRSLLMPAFTRAALRGYRDMIASVAREHITRWRPHATINSLDHMNALTLDIILRVVFGVTDPKVKAELTSRLQQIINIHPAILAGVPYPSLKRMNPWKRFFHNQTKIDXXXXRXXXXRRIDSDLTARTDVLSRLLQTKDTPTKPLTDAELRDQLITLLLAGHETTAAALSWTLWELAHAPEIQSQVVWAAVGGDDGFLEAVLKEGMRRHTVIASTARKVTAPAEIGGWRLPAGTVVNTSILLAHASEVSHPKPTEFRPSRFLDGSVAPNTWLPFGGGVRRCLGFGFALTEGAVILQEIFXRFTITAAGPSKGETPLVRNITTVPKHGAHLRLIPQRRLGGXWXQXPXJAXGAAHLPRNRVPPRVPNR

>120|CORE_REP|Org59_Gene259#

MSVMDVFMDAALACTVLDYGDHALMLQCDSTADAMAWTDALRAAALPGVVDIVAASRTVLVKLDAPRYQGVTRQRLRRLRVTPEAVAAADHRCDLVIDVVYDGPXLAEVARCTGLTTAAVINAHTATGWRAGFSGSAPGFAYLIDGDPSLRVPRRPERRTSMPPGSVALADGFSAIYPSQAPSDWQIIGHTDAVLWDVDRPQPALLTPGMWVQFRXAXGXEEAAMTTLEILRSGPLALVEDLGRAGLAHLGVGRSGAADRRSHTLANRLVANPDDWATVEVTFGGFSARVRGGDVDIAVTGADTDPTVNGIMVGTNSIHHVRDGQVISLGTPRAGLRTYLAVRGGVCVEPVLGSRSYDVMSAIGPSPLRAGDVLPVGEHTDDYPELDQAPVAAIEEHLVELRVVPGPRDDWLVDPDALVHTIWMASNRSDRVGMRLQGRIRYSTAGRIGNCPAKALPAAQSRCRPTDYR

>121|CORE_REP|Org1_Gene2216#

MRARRLVMLRHGQTDYNVGSRMQGQLDTELSELGRTQAVAAAEVLGKRQPLLIVSSDLRRAYDTAVKLGERTGLVVRVDTRLRETHLGDWQGLTHAQIDADAPGARLAWREDATWAPHGGESRVDVAARSRPLVAELVASEPEWGGADEPDRPVVLVAHGGLIAALSAALLKLPVANWPALGGMGNASWTQLSGHWARAPTSRASGGALMCGMLRRRSPAMSSRRGRRPALLVFADSLAYYGPTGGLPADDPRIWPNIVASQLDWDLELIGRIGWTCRDVWWAATQDPRAWAALPRAGAVIFATGGMDSLPSVLPTALRELIRYVRPSWLRRWVRDGYAWVQPRLSPVARAALPPHLTAEYLEKTRGAIDFNRPGIPIIASLPSVHIAETYGKAHHGRAGTVAAITEWAQHHDIPLVDLKAAVAEQILSGYGNRDGIHWNFEAHQAVAELMLKALAEAGVPNEKSRG

>122|CORE_REP|Org64_Gene2827#

MKSTVEQLSPTRVRINVEVPFAELEPDFQRAYKELAKQVRLPGFRPGKAPAKLLEARIGREAMLDQIVNDALPSRYGQAVAESDVQPLGRPNIEVTKKEYGQDLQFTAEVDIRPKISPPDLSALTVSVDPIEIGEDDVDAELQSLRTRFGTLTAVDRPVAVGDVVSIDLSATVDGEDIPNAAAEGLSHEVGSGRLIAGLDDAVVGLSADESRVFTAKLAAGEHAGQEAQVTVTVRSVKERELPEPDDEFAQLASEFDSIDELRASLSDQVRQAKRAQQAEQIRNATIDALLEQVDVPLPESYVQAQFDSVLHSALSGLNHDEARFNELLVEQGSSRAAFDAEARTASEKDVKRQLLLDALADELQVQVGQDDLTERLVTTSRQYGIEPQQLFGYLQERNQLPTMFADVRRELAIRAAVEAATVTDSDGNTIDTSEFFGKRVSAGEAEEAEPADEGAARAASDEATT

>123|CORE_REP|Org94_Gene1437#

MNWTVDIPIDQLPSLPPLPTDLRTRLDAALAKPAAQQPTWPADQALAMRTVLESVPPVTVPSEIVRLQEQLAQVAKGEAFLLQGGDCAETFMDNTEPHIRGNVRALLQMAVVLTYGASMPVVKVARIAGQYAKPRSADIDALGLRSYRGDMINGFAPDAAAREHDPSRLVRAYANASAAMNLVRALTSSGLASLHLVHDWNREFVRTSPAGARYEALATEIDRGLRFMSACGVADRNLQTAEIYASHEALVLDYERAMLRLSDGEDGEPQLFDLSAHTVWIGERTRQIDGAHIAFAQVIANPVGVKLGPNMTPELAVEYVERLDPHNKPGRLTLVSRMGNHKVRDLLPPIVEKVQATGHQVIWQCDPMHGNTHESSTGFKTRHFDRIVDEVQGFFEVHRALGTHPGGIHVEITGENVTECLGGAQDISETDLAGRYETACDPRLNTQQSLELAFLVAEMLRD

>124|CORE_REP|Org22_Gene1243#

MAQAPHIHRTRYAKCGDMDIAYQVLGDGPTDLLVLPGPFVPIDSIDDEPSLYRFHRRLASFSRVIRLDHRGVGLSSRLAAITTLGPKFWAQDAIAVMDAVGCEQATIFAPSFHAMNGLVLAADYPERVRSLIVVNGSARPLWAPDYPVGAQVRRADPFLTVALEPDAVERGFDVLSIVAPTVAGDDVFRAWWDLAGNRAGPPSMARAVSKVIAEADVRDVLGHIEAPTLILHRVGSTYIPVGHGRYLAEHIAGSRLVELPGTDTLYWVGDTGPMLDEIEEFITGVRGGADAERMLATIMFTDIVGSTQHAAALGDDRWRDLLDNHDTIVCHEIQRFGGREVNTAGDGFVATFTSPSAAIACADDIVDAVAALGIEVRIGIHAGEVEVRDASHGTDVAGVAVHIGARVCALAGPSEVLVSSTVRDIVAGSRHRFAERGEQELKGVPGRWRLCVLMRDDATRTR

>125|CORE_REP|Org87_Gene220#

MIRAAFACLAATVVVAGWWTPPAWAIGPPVVDAAAQPPSGDPGPVAPMEQRGACSVSGVIPGTDPGVPTPSQTMLNLPAAWQFSRGEGQLVAIIDTGVQPGPRLPNVDAGGDFVESTDGLTDCDGHGTLVAGIVAGQPGNDGFSGVAPAARLLSIRAMSTKFSPRTSGGDPQLAQATLDVAVLAGAIVHAADLGAKVINVSTITCLPADRMVDQAALGAAIRYAAVDKDAVIVAAAGNTGASGSVSASCDSNPLTDLSRPDDPRNWAGVTSVSIPSWWQPYVLSVASLTSAGQPSKFSMPGPWVGIAAPGENIASVSNSGDGALANGLPDAHQKLVALSGTSYAAGYVSGVAALVRSRYPGLNATEVVRRLTATAHRGARESSNIVGAGNLDAVAALTWQLPAEPGGGAAPAKPVADPPVPAPKDTTPRNVAFAGAAALSVLVGLTAATVAIARRRREPTE

>126|CORE_REP|Org67_Gene3996#

MSIRPAENSTLDIRHVIGIGTPKAVDLWLDVVTELPDRARELGSLSKAELGKLGPLLDGTNAVELFESIDDKLAAEALHAMDPSLAATFLEALDSDHAANILREFKEPKREALLTLLPLERAMVLRGLLSWPEDCAAAHMVPETLTVRPNMTVSQAVASVRERASGLRSDARTTAYVYVTDADSHLLGVIAFRALVLANPEQRVRELMGDDLIVVSPLTDKELAAQTIMGHNLMAVPVVDADNRLLGIIAEDEAIDIAEEEATEDAERQGGSAPLEVPYLRASPWLLWRKRVVWLLVLFAAEAYTGSVLRAFSDEMQAVIALAFFIPLLIGTGGNTGTQIATTLVRAMATGQVRFRDVPAVLAKELSTGVLVGLTMAAAAVVRAWTLGVGPQVTLTVALTVAAIVVWSSLVAAVLPPLLKKLRIDPAIVSGPMIATIVDGTGLLIYFLVAHLTLTELHGL

>128|CORE_REP|Org46_Gene4085#

MARLKVPEGWCVQAFRFTLNPTQTQAASLARHFGARRKAFNWTVTALKADIKAWRADGTESAKPSLRVLRKRWNTVKDQVCVNAQTGQVWWPECSKEAYADGIAGAVDAYWNWQSCRAGKRAGKTVGVPRFKKKGRDADRVCFTTGAMRVEPDRRHLTLPVIGTIRTYENTRRVERLIAKGRARVLAITVRRNGTRLDASVRVLVQRPQQRRVALPDSRVGVDVGVRRLATVADAEGTVLEQVPNPRPLDAVPLRGLRRVSRARSRCTKGSRRYCERTTELSRLHRRVNDVRTHHLHVLTTRLAKTHGRIVVEGLDAAGMLRQKGLPGARARRRALSDAALATPRRHLSYKTGWYGSSLVVADRWFPSSKTCHACRHVQDIGWDEKWQCDGCSITHQRDDNAAINLARYEEPPSVVGPVGAAVKRGADRKTGPGPAGGREARKATGHPAGEQPRDGVQVK

>130|CORE_REP|Org118_Gene2607#

MPRRSPADPAAALAPRRTTLPGGLRVVTEFLPAVHSASVGVWVGVGSRDEGATVAGAAHFLEHLLFKSTPTRSAVDIAQAMDAVGGELNAFTAKEHTCYYAHVLGSDLPLAVDLVADVVLNGRCAADDVEVERDVVLEEIAMRDDDPEDALADMFLAALFGDHPVGRPVIGSAQSVSVMTRAQLQSFHLRRYTPERMVVAAAGNVDHDGLVALVREHFGSRLVRGRRPVAPRKGTGRVNGSPRLTLVSRDAEQTHVSLGIRTPGRGWEHRWALSVLHTALGGGLSSRLFQEVRETRGLAYSVYSALDLFADSGALSVYAACLPERFADVMRVTADVLESVARDGITEAGMRHRQGIAAGWAGARAGGFQLPDEPARPQRVELRQAPQHRTHLAANRAGHRGGGQRGGPPPAEQALRCCRSWPTRIETITAATTSSDGRVAQMSASSLAPCRRSPSAN

>131|CORE_REP|Org35_Gene3236#

MVAHRAEVSGSPPPRLNLSTQPTVARRVRASFAESFAAADPEADAARRMALRRMKVVAVGFLVGATGVFLACRWAQADGADHAWLGYLGAAAEAGMVGALADWFAVTALFKHPLGIPIPHTAIIKRKKDQLGEGLGTFVRENFLSPPVVETKLRDAQIPSRLGKWLSEATHAQRVAAETATVLRVLVELLRDEDIQQVIDRMIVRRIAEPQWGPPAGRVLATLLAENRQEAFIQLLADRAFQWSLNAGVVIQRVVERDSPSWSPRFIDHLVGDRIHRELMEFTDKVRRNPDHELRRSATRFLFDFADDLQHDPATVARADAIKEELMARDEIATAAAAAWKTLKRLVLEGVDDPSSALRTRITDAVIRIGESLRDDADLRDKVDSWMVRAAQHLVSEYGVEITAIITETIERWDAEEASRRIELHVGRDLQFIRINGTVVGAMAGLAIYAIAQLLF

>132|CORE_REP|Org69_Gene148#

MTTPGKLNKARVPPYKTAGLGLVLVFALVVALVYLQFRGEFTPKTQLTMLSARAGLVMDPGSKVTYNGVEIGRVDTISEVTRDGESAAKFILDVDPRYIHLIPANVNADIKATTVFGGKYVSLTTPKNPTKRRITPKDVIDVRSVTTEINTLFQTLTSIAEKVDPVKLNLTLSAAAEALTGLGDKFGESIVNANTVLDDLNSRMPQSRHDIQQLAALGDVYADAAPDLFDFLDSSVTTARTINAQQAELDSALLAAAGFGNTTADVFDRGGPYLQRGVADLVPTATLLDTYSPELFCTIRNFYDADPLAKAAAGGGNGYSLRTNSEILSGIGISLLSPLALATNGAAIGIGLVAGLIAPPLAVAANLAGALPGIVGGAPNPYTYPENLPRVNARGGPGGAPGCWQPITRDLWPAPYLVMDTGASLAPYNHMEVGSPYAVEYVWGRQVGDNTINP

>133|CORE_REP|Org59_Gene1971#

MADQPDPPTPRPALSPSRATDFKQCPLLYRFRAIDRLPEATSAAQLRGSVVHAALEQLYGLPAGLRSPDTARSLVQRAWDQMVAAEPELAGELDPGQPTPXAGLXXRALVSGYYRLEDPTRFDPQXXEQRVEVELADGTLLRGYIDRIDVAATGELRVVDYKTGKAPPAARALAEFKAMFQMKFYAVALFRSRGVPPTRLRLIYLADGQLLDYSPDRDELLRFEKTLMAIWRAIQSAGETGDXRPNPSRLCDWCPHQQRCPAFGGTPPPYPGWPHRAGGINDRVEVRCHRAAAAATMANPPNTATESSSSAIGXXXXAXGXHQPRTCLIAAHGAEHGADNPSAKPAPPVAPRGADDRAGERAQNDPDSEHRRHGTRRCFGTFVVNEFGDRKNADDHHGHEIAHPGCPGSMQVDPAEKGGPGDHGRGRQGTQPGDDTDKQRQQQNKDMRHSDPS

>134|CORE_REP|Org99_Gene1764#

MTPRSYCVVGGGISGLTSAYRLRQAVGDDATITLFEPADRLGGVLRTEHIGGQPMDLGAEAFVLRRPEMPALLAELGLSDRQLASTGARPLIYSQQRLHPLPPQTVVGIPSSAGSMAGLVDDATLARIDAEAARPFTWQVGSDPAVADLVADRFGDQVVARSVDPLLSGVYAGSAATIGLRAAAPSVAAALDRGATSVTDAVRQALPPGSGGPVFGALDGGYQVLLDGLVRRSRVHWVRARVVQLERGWVLRDETGGRWQADAVILAVPAPRLARLVDGIAPRTHAAARQIVSASSAVVALAVPGGTAFPHCSGVLVAGDESPHAKAITLSSRKWGQRGDVALLRLSFGRFGDEPALTASDDQLLAWAADDLVTVFGVAVDPVDVRVRRWIEAMPQYGPGHADVVAELRAGLPPTLAVAGSYLDGIGVPACVGAAGRAVTSVIEALDAQVAR

>135|CORE_REP|Org4_Gene347#

MVIRVLFRPVSLIPVNNSSTPQSQGPISRRLALTALGFGVLAPNVLVACAGKVTKLAEKRPPPAPRLTFRPADSAADVVPIAPISVEVGDGWFQRVALTNSAGKVVAGAYSRDRTIYTITEPLGYDTTYTWSGSAVGHDGKAVPVAGKFTTVAPVKTINAGFQLADGQTVGIAAPVIIQFDSPISDKAAVERALTVTTDPPVEGGWAWLPDEAQGARVHWRPREYYPAGTTVDVDAKLYGLPFGDGAYGAQDMSLHFQIGRRQVVKAEVSSHRIQVVTDAGVIMDFPCSYGEADLARNVTRNGIHVVTEKYSDFYMSNPAAGYSHIHERWAVRISNNGEFIHANPMSAGAQGNSNVTNGCINLSTENAEQYYRSAVYGDPVEVTGSSIQLSYADGDIWDWAVDWDTWVSMSALPPPAAKPAATQIPVTAPVTPSDAPTPSGTPTTTNGPGG

>136|CORE_REP|Org80_Gene2565#

MSAVALPRVSGGHDEHGHLEEFRTDPIGLMQRVRDECGDVGTFQLAGKQVVLLSGSHANEFFFRAGDDDLDQAKAYPFMTPIFGEGVVFDASPERRKEMLHNAALRGEQMKGHAATIEDQVRRMIADWGEAGEIDLLDFFAELTIYTSSACLIGKKFRDQLDGRFAKLYHELERGTDPLAYVDPYLPIESFRRRDEARNGLVALVADIMNGRIANPPTDKSDRDMLDVLIAVKAETGTPRFSADEITGMFISMMFAGHHTSSGTASWTLIELMRHRDAYAAVIDELDELYGDGRSVSFHALRQIPQLENVLKETLRLHPPLIILMRVAKGEFEVQGHRIHEGDLVAASPAISNRIPEDFPDPHDFVPARYEQPRQEDLLNRWTWIPFGAGRHRCVGAAFAIMQIKAIFSVLLREYEFEMAQPPESYRNDHSKMVVQLAQPACVRYRRRTGV

>137|CORE_REP|Org59_Gene135#

MPVRRRAGERLPTVWDFETDPQYQSKLDWVEKFMAEELEPLDLVALDPYDKKNADTMAILRPLQRQGERXGVVGRAFASRTRRTGXRSGAKLALLNEIIGRSRWAPSAFGCQAPDSGNAEILALXGIXXXESPLSTTAARRRDHLLLFDDRAAGWFRSRAVRDRGDPRCRGKRGLDHQRREVVFHXRQACVVLYRHGRHQAGSPYVREDVAVHRPGRHPGHRDRAQLSGVGAEXXRXXSHGYIRYHDVRVPADGHVLGGEGQAFMIAQTRLGGGRIHHAMRTIALARRAFDMMCERALSRQTRHGRLADLQMTQEKIADSWIQIEQFRLLVLRTAWLIDKHHDYQKVRRDIAAVKVAMPQVLHDVVQRAMHLHGALGVSDEMPFVKMMLAAESLGIADGATELHKMTVARRTLREYQPVTTLFPSQHIPTRRAHAEAWLAQRLEHAIAEF

>138|CORE_REP|Org116_Gene928#

MSTFIGQLFGFAVIVYLVWRFIVPLVGRLMSARQDTVRQQLADAAAAADRLAEASQAHTKALEDAKSEAHRVVEEARTDAERIAEQLEAQADVEAERIKMQGARQVDLIRAQLTRQLRLELGHESVRQARELVRNHVADQAQQSATVDRFLDQLDAMAPATADVDYPLLAKMRSASRRALTSLVDWFGTMAQDLDHQGLTTLAGELVSVARLLDREAVVTRYLTVPAEDATPRIRLIERLVSGKVGAPTLEVLRTAVSKRWSANSDLIDAIEHVSRQALLELAERAGQVDEVEDQLFRFSRILDVQPRLAILLGDCAVPAEGRVRLLRKVLERADSTVNPVVVALLSHTVELLRGQAVEEAVLFLAEVAVARRGEIVAQVGAAAELSDAQRTRLTEVLSRIYGHPVTVQLHIDAALLGGLSIAVGDEVIDGTLSSRLAAAEARLPD

>139|CORE_REP|Org100_Gene3262#

MDVAGLPRLAAGTQAAIIHGMAQPPSLLTTDNGLPFGVQGACDSRFTGVIRAFAGLYPGRKFGGGALSVYIDGRQVVDVWTGWSDRQGKVPWTADTGAMVFSATKGLAATVIHRLVDRGLLSYDAPVAEYWPEFGANGKSEVTVSDVLRHRSGLAHLKGVDKDEVMDHLLMEQKLAAAPLDRQHGKLAYHAVTYGWLLSGLARAVTGKGMRELFREELARPLNTDGIHLGRPPADSPTKAAQTLLPQAKVPTPLLDFIAPKVAGLSFSGLLGAVYFPGILSLLQDDMPFLDGEVPAVNGVVTARALAKTYGALANDGVIDGTRLLSSQAVRGLTGKSELWPDLNLGLPFTYHQGYQSSPVPGLLEGYGHIGLGGTIGWADPETGSAFGYVHNRLLTLLLFDIGSFAGLAALLNSAVVAARRDDPLEVPHFGAPYSEPRHEQAASGA

>140|CORE_REP|Org20_Gene3646#

MAVGAAAVTEVGDTASPVGSSGASGGAIASGIVARVGTATAVTALCGYAVIYLAARNLAPNGFSVFGVFWGAFGLVTGAANGLLQETTREVRSLGYLDVSADGRRTHPLRVSGMVGLGSLVVIAGSSPLWSGRVFAEARWLSVALLSIGLAGFCLHATLLGMLAGTNRWTQYGALMVADAVIRVVVAAATFVIGWQLVGFIWATVAGSVAWLIMLMTSPPTRAAARLMTPGATATFLRGAAHSIIAAGASAILVMGFPVLLKLTSNELGAQGGVVILAVTLTRAPLLVPLTAMQGNLIAHFVDERTERIRALIAPAALIGGVGAVGMLAAGVVGPWIMRVAFGSEYQSSSALLAWLTAAAVAIAMLTLTGAAAVAPLDRAIRWAGLVRRLGRACCCCCRCPWRPAPWSRCYAVRWWESASIWWRWRGRTSKRPISPGPTCNLWA

>141|CORE_REP|Org122_Gene3433#

MSDPARGAEAEDAYGFPAGLWRWLQRHPPPALHRLTRFRSPLRGPWLTSVFGLVLLVALPFVIITGLLSYIAYAPQLGQAIPGDVGWLRLPAFTWPTRPSWLYRLTQGLHVGLGLVIIPVVLAKLWSVIPRLFVWPPARSIAQVLERLSVLMLVGGILFQIVTGVLNIQYDYIFGFSFYTGHYFGAWVFIAGFLLHIVVKIPHMVTGLRSIPMREVLGTNVADTRAQPCDPDGLVSVNPGEATLSRRGALGLVGAGVLLIGVLTVGQTLGGFTRKAALLLPRGRVVSPGDFPVNKTAAAAGITAEAIGPDWRLVLRGGPAEVVLDRATLAGLPQRTARLPLACVEGWSAVRTWSGVPLAELALLAGVPAARSARVTSLQRGGAFGEAKLAANQIADPDALLALRVDGADLSLDHGYPARIIVPALPGVHNTKWVAGIEFHKR

>142|CORE_REP|Org96_Gene291#

MAVGRCAIPRFDQAASGSAINGGQVHLSDGSTSPARQLPAPWPGDAGAAAEGRAGVCCRGNRLPHVSDVGVSHRFDHRPAGVGAGGCRAGAAGAGLAVDDPGQLAAAIDRIVAVADPDAVRQVRERARDREVSIWNSADGMGEVYAQLYATDAQALDARLNALVATVCAGDPRSTDQRRADALGALAAGADRLACRCDNPDCAAEGRPVSAVVIHVVAEQASVKGHGQAPAALLGGDGLIPAELVAELAKTAGLQPIPVPAGTEPGYRPSVKLAAFVRARDLTCRAPGCDRPATQCDLDHTIAFADGGATHAANLKCLCRLHHLLATFCGWRAQQLPDGTVIWTLPGNQTYVTTPGSALLFPALCTPTGDPPAPEPARADRRGQRTAMMPRRASTRTQNRAHCIAAERHRNHQARRIAQAAVIATETHGPPPDPDDDPPPF

>143|CORE_REP|Org1_Gene2272#

MMRRPITLAEQLDAEDAKLVVLARAAMARAEAGAGAAVRDVDGRTYAAAPVALSALELTGLQAAVAAAVSSGATGLQAAVLVAGSVDDPGIAAVRELARPLRSSSPIGQVTRYDAERAFTAEKSGKSLPVFTFGEETGMTEFHSGFVCLVGRPNTGKSTLTNALVGAKVAITSTRPQTTRHAIRGIVHSDDFQIILVDTPGLHRPRTLLGKRLNDLVRETYAAVDVIGLCIPADEAIGPGDRWIVEQLRSTGPANTTLVVIVTKIDKVPKEKVVAQLVAVSELVTNAAEIVPVSAMTGDRVDLLIDVLAAALPAGPAYYPDGELTDEPEEVLMAELIREAALQGVRDELPHSLAVVIDEVSPREGRDDLIDVHAALYVERDSQKGIVIGKGGARLREVGTAARSQIENLLGTKVYLDLRVKVAKNWQRDPKQLGRLGF

>144|CORE_REP|Org40_Gene2846#

MVLPKPTPRGRELIRQAAKVALHPTPEWLDELDRATLAAHPSIAADPALATVVSRANRSHLIHFATANLRKPGQPVPANLGPDPLRMARDLVRRGLDASALDVYRVGQNVAWQRWTEIAFGLTTDPQELHELLTLPFRSASEFIDATLAGLAAQMQLEYDELTRDVHAEHRRIVELILDGAPISRQSAEAKLGYPLDRSHTAAIIWYDDPDDNQNHLDHTARAFGRALGCPQPLIAVASAATRWVWVSDAATLDTDRIHQVLDHAPHARIAVGTTARGIDGFRRSHRDALATQRMLARLRSQQRLAFFADIHMIAVLTENPDSAADFITSTLGDLESASPQLLTTVLTYINEQCNASRAAHVLHTHRNTLLRRLETAQRLLPRPLDHTIIQVAVAISALQWRGSQTSDPVETPVEGITSPPPESLGRRRSRLAQLER

>145|CORE_REP|Org118_Gene2593#

MYAGAGAAPLMAAGATWNGLAVELSTTASSVESVIMQLTTEQWLGPASMSMVVAAQPYLAWLTYTAESAAHAAAQAMASAAAFEAAFAMTVPPAEVAANRALLAALVATNVLGQNTPAIMATEAHYGEMWAQDALAMYGYAASSAAAGRLNPLITPSQTANMAGLAGQAAAVSHAAAASTVQQVGLGSLISNLPNAVMGFASPLTSAADAAGLGGIIQDIEELLGITFVQNAINGAVNTTAWFVMATIPNAVFLGHAFAALNPATVTAAADAVPAAAAAAGLAHTVTPVGVGGASLTASLGEASSVGGLSVPAGWSTAAPAMTFWYHGTGGLGLGGPRGSRASRRNCRVWRGFLGRPKEPVPMPGLGTGSSPSSCPNRSSSDWPVAHRPGRPAMSRTATRQAWPVRQPTVGPQPTPPGPSIKPPEPWRRSRPRRAR

>146|CORE_REP|Org118_Gene2595#

MDFGALPPEVNSARMYGGAGAADLLAAAAAWNGIAVEVSTAASSVGSVITRLSTEHWMGPASLSMAAAVQPYLVWLTCTAESSALAAAQAMASAAAFETAFALTVPPAEVVANRALLAELTATNILGQNVSAIAATEARYGEMWAQDASAMYGYAAASAVAARLNPLTRPSHITNPAGLAHQAAAVGQAGASAFARQVGLSHLISDVADAVLSFASPVMSAADTGLEAVRQFLNLDVPAVRRIRVSRPGWRGRLCHGRHWQYDASCRCYGNRWRSRSRWRRGSRGGTRGCPSGRRRNSADRRFGQCVRGWSPVGAGKLVYCSAGDGSRRGLGWHRLGSSRGGRPDRSDAACPWNGRSRQQCWCRLRTTVRSQADCYAQARPLLIWRHRDKRTRPTSAPPRPHCANPPAARVSFRCCGERSGATLKRIVLRLPVPA

>148|CORE_REP|Org7_Gene3716#

MRKDKALARRLPAAVAAAVIAVELGGCGSADSWVEAAPAQGWPAQYGDAANSSYTTTNGATNLTLRWTRSVKGSLAAGPALSARGYLALNGQTPAGCSLMEWQNDNNGRQRWCVRLVQGGGFAGPLFDGFDNLYVGQPGAIISFPPTQWTRWRQPVIGMPSTPRFLGHGRLLVSTHLGQLLVFDTRRGMVVGSPVDLVDGIDPTDATRGLADCAPARPGCPVAAAPAFSSVNGTVVVSVWQPGEPAAKLVGLKYHAEQLVREWTSDAVSAGVLASPVLSADGSTVYVNGRDHRLWALNAADGKAKWSAPLGFLAQTPPALTPHGLIVSGGGPDTALAAFRDAGDHAEGAWRRDDVTALSTASLAGTGVGYTVISGPNHDGTPGLSLLVFDPANGHTVNSYPLPGATGYPVGVSVGNDRRVVTATSDGQVYSFAP

>149|CORE_REP|Org118_Gene2287#

MAPRVCVVGSVNMDLTFVVDALPRPGETVLAASLTRTPGGKGANQAVAAARAGAQVQFSGAFGDDPAAAQLRAHLRANAVGLDRTVTVPGPSGTAIIVVDASAENTVLVAPGANAHLTPVPSAVANCDVLLTQLEIPVATALAAARAAQSADAVVMVNASPAGQDRSSLQDLAAIADVVIANEHEANDWPSPPTHFVITLGVRGARYVGADGVFEVPAPTVTPVDTAGAGDVFAGVLAANWPRKPRFAGRATARIAAGLRCGCAGNFGVRCRRLRTGRRRDRCGPASQPPQRFMTTATHRRRPADANDHRGRLSAGTLRGFQCGRIRLTQLAAAPTHRIHRLSRRQRPGRFAGVDYRDVLCHRPDRDGGRCGAAADQRCPTAEYSAHGLDSGCRHNPGDGRDRGHGLRPACDGRFVADRGGRAGEHHSGAHRPV

>150|CORE_REP|Org36_Gene786#

MTDTRTYVLDTSVLLSDPWACSRFAEHDVVVPLVVISELEAKRHHHELGWFARQALRLFDDLRLEHGRLDQPIPVGTQGGTLHVELNHTDPAVLPAGFRTDSNDSRILSCAANLAAEGKRVTLVSKDIPLRVKAAAVGLAADEYHAQDVVVSGWSGMHELETASADIDALFADGEIDLVEARDLPCHTGIRLLGGGSHALGRVNAHKRVQLVRGDREAFGLRGRSAEQRVALDLLLDESVGIVSLGGKAGTGKSALALCAGLEAVLERRTHRKVVVFRPLYAVGGQELGYLPGSESEKMGPWAQAVFDTLEGLASPAVLEEVLSRGMLEVLPLTHIRGRSLHDSFVIVDEAQSLERNVLLTVLSRLGTGSRVVLTHDIAQRDNLRVGRHDGVAAVIEKLKGHPLFAHITLLRSERSPIAALVTEMLEEITGPR

>151|CORE_REP|Org12_Gene3891#

MSSWPPRAGSTGSTIAASTSTAATSRRSNSRLPTTLNARDQPPAEVSDQRVSGLTGAVHYAGAGSGPLFMAAAAWEGLAADLRASASSFDAVIAGLAAGPWSGPASVAMAGAAAPYVGWLSAAAGQAELSAGQATAAATAFEAALAATVHPAAVTANRVLLGALVATNILGQNTPAIAATEFDYVEMWAQDVGAMVGYHAGAAAVAETLTPFSVPPLDLAGLASQAGAQLTGMATSVSAALSPIAEGAVEGVPAVVAAAQSVAAGLPVDAALQVGQAAAYPASMLIGPMMQLAQMGTTANTAGLAGAEAAGLAAADVPTFAGDIASGTGLGGAGGLGAGMSAELGKARLVGAMSVPPTWEGSVPARMASSAMAGLGAMPAEVPAAGGPMGMMPMPMGMGGAGAGMPAGMMGRGGANPHVVQARPSVVPRVGIG

>152|CORE_REP|Org2_Gene2223#

MPRPVRHDADVAAPRLTGDQRNAFMASFLGWTMDAFDYFLVVLVYADIATTFHHTKTDVAFLTTATLAMRPVGALLFGLWADRVGRRVPLMVDVSFYSVIGFLCAFAPNFTVLVILRLLYGIGMGGEWGLGAALSMEKVPAERRGVFSGLLQEGYAFGYLLASVAALVVMNWLGLSWRWLFGLSIIPALISLIIRYRVKESEVWEAAQDRMRLTKTRIRDVLGNPAIVRRFVYLVLLMTAFNWMSHGTQDVYPTFLTATTDHGAGLSSLTARWIVVIYNIGAIIGGLAFGTLSQRFSRRYTIVFCAALGLPIVPLFAYSRTAAMLCLGSFLMQVFVQGAWGVIPAHLTEMSPDAIRGVYPGVTYQLGNLLAAFNLPIQERLAESHGYPFALAATIVPVLLVVAVLTAIGKDATGIRFGTTETAFLVRHRNRH

>153|CORE_REP|Org20_Gene2410#

MRTELPAERLQRRLGAVPDIDSHAASAHLDPEPHDPTDDGPDHDEPRDDPNSLLPRWLPDTSRGQGWADRIRADPGRAGAVALAVIAALAVLVTVFTLIRDRTEPVMSAKLPPVEPVSPTNPRSSASPGSPDRSGLPVVVSVVGLVHTPGLVTLAPGARIADALQAAGGAVDGADTVGLNMARQLGDGEQIVVGLAPPSGQPRVLGSSVGAGTPGPAGTSGTATTGPKTAPKTAEVLDLNTATVEQLDALPGIGPVTAAAIVAWRQRNGRFTSVDQLADVDGIGPARLDKRRIWSVSDTGAPYGLRRVPFGRTPGPGGAGQLDCDGGRDRVADRQRVCLVLRRGGPRRRRTWLGSQRGTLRDWVRSAPAWSRSVWWARGTGLRSRCAPRRSIATQSPWHLAPPRWSRSPPARAQCRWGGAVDSPGDGSTAAG

>154|CORE_REP|Org118_Gene3139#

MSLSFGSAVGAYERGRPSYPPEAIDWLLPAAARRVLDLGAGTGKLTTRLVERGLDVVAVDPIPEMLDVLRAALPQTVALLGTAEEIPLDDNSVDAVLVAQAWHWVDPARAIPEVARVLRPGGRLGLVWNTRDERLGWVRELGEIIGRDGDPVRDRVTLPEPFTTVQRHQVEWTNYLTPQALIDLVASRSYCITSPAQVRTKTLDRVRQLLATHPALANSNGLALALRHGLCAGDSGLTPPLGPGAGVNQARQLLADVAEAGDQGRGDQAQRAGVVHTRDVRAAVEDAGELGAEVGEARRRSEQRIRDRVEHARHARAEVVEARQPAATADVEEPRRRDRGGVAESRDGRDDQPGVDGAEQRVDVENHWDSVDGDLQREGEGGGGAGQREAQRELGNGDFRAHEGSDGDGQGQLDMDWIDGYVESRDGDFRC

>155|CORE_REP|Org40_Gene1460#

MSRADDDAVGVPPTCGGRSDEEERRIVPGPNPQDGAKDGAKATAVPREPDEAALAAMSNQELLALGGKLDGVRIAYKEPRWPVEGTKAEKRAERSVAVWLLLGGVFGLALLLIFLFWPWEFKAADGESDFIYSLTTPLYGLTFGLSILSIAIGAVLYQKRFIPEEISIQERHDGASREIDRKTVVANLTDAFEGSTIRRRKLIGLSFGVGMGAFGLGTLVAFAGGLIKNPWKPVVPTAEGKKAVLWTSGWTPRYQGETIYLARATGTEDGPPFIKMRPEDMDAGGMETVFPWRESDGDGTTVESHHKLQEIAMGIRNPVMLIRIKPSDLGRVVKRKGQESFNFGEFFAFTKVCSHLGCPSSLYEQQSYRILCPCHQSQFDALHFAKPIFGPAARALAQLPITIDTDGYLVANGDFVEPVGPAFWERTTT

>157|CORE_REP|Org78_Gene1074#

MSAELSQSPSSSPLFSLSGADIDRAAKRIAPVVTPTPLQPSDRLSAITGATVYLKREDLQTVRSYKLRGAYNLLVQLSDEELAAGVVCSSAGNHAQGFAYACRCLGVHGRVYVPAKTPKQKRDRIRYHGGEFIDLIVGGSTYDLAAAAALEDVERTGATLVPPFDDLRTIAGQGTIAVEVLGQLEDEPDLVVVPVGGGGCIAGITTYLAERTTNTAVLGVEPAGAAAMMAALAAGEPVTLDHVDQFVDGAAVNRAGTLTYAALAAAGDMVSLTTVDEGAVCTAMLDLYQNEGIIAEPAGALSVAGLLEADIEPGSTVVCLISGGNNDVSRYGEVLERSLVHLGLKHYFLVDFPQEPGALRRFLDDVLGPNDDITLFEYVKRNNRETGEALVGIELGSAADLDGLLARMRATDIHVEALEPGSPAYRYLL

>159|CORE_REP|Org59_Gene1576#

MDFGALPPEVNSGRMYCGPGSAPMVAAASAWNGLAAELSVAAVGYERVITTLQTEEWLGPASTLMVEAVAPYVAWMRATAIQAEQAASQARAAAAAYETAFAAIVPPPLIAANRARLTSLVTHNVFGQNTASIAATEAQYAEMWAQDAMAMYGYAGSSATATKVTPFAPPPNTTSPSAAATQLSAVAKAAGTSAGAAQSAIAELIAHLPNTLLGLTSPLSSALTAAATPGWLEWFINWYLPISQLFYNTVGLPYFAIGIGNSLITSWRALGXXGPXAAEAAAAAPAAVGAAVGGTGPVSAGLGNAATIGKLSLPPNWAGASPSLAPTVGSASAPLVSDIVEQPEAGAAGKPVGRHAASRFGHRYGGCGSPLRVPGYGDVPAAVCRITRGLPYPRTRGRHICRTIXRLPXRQTPSRTCVGTXPXAIFS

>161|CORE_REP|Org119_Gene1671#

MDFGLLPPEINSGRMYTGPGPGPMLAAATAWDGLAVELHATAAGYASELSALTGAWSGPSSTSMASAAAPYVAWMSATAVHAELAGAQARLAIAAYEAAFAATVPPPVIAANRAQLMVLIATNIFGQNTPAIMMTEAQYMEMWAQDAAAMYGYAGSSATASRMTAFTEPPQTTNHGQLGAQSSAVAQTAATAAGGNLQSAFPQLLSAVPRALQGLALPTASQSASATPQWVTDLGNLSTFLGGAVTGPYTFPGVLPPSGVPYLLGIRMARVLGTQNGQGVSALLGKIGGKPITGALAPLAEFALHTPILGSEGLGGGSVSAGIGRAGLVGKLSVPQGWTVAAPEIPSPAAALQATRLAAAPIAATDGAGALLGGMALSGLAGRAAAGSTGHPIGSAAAPAVGAAAAAVEDLATEANIFVIPAMDD

>162|CORE_REP|Org2_Gene2983#

MTPPPSPAGEDQLEANRRAVLDAVSDARRDGFAVGEDYTVTDRSTGGSRQQRAARLGQAQGHADFIRHRVGALLATDRDIATRVSAATQGLDELAFEDVPGVDTPAEDGVQAVDFRQAPPPGAPGGMSSGDIDAIDAANRALLQDMLAEYSRLPDGQVKTDRLADIAAIQEALRVPDSHLIYVARPDDPADMIPAVTAVGDPFTADHVSVTVPGVSGTTRQTIATMTQEARGLREEARVIAHSVGESENVATIAWVGYQPPPVLASWNTVDDDLAQAGAPKLEAFLRDLQAGSHNPGHTTALFGHSYGSLLSGIALKDGASSLVDNAVLYGSPGFDATSPAKLGMNDHNFFVMTTPDDPIRYPARLAPLHGWGSDGADTIGTVGRQGTPARVGIRPQRDHRRIPGPLPLHPSADRRGIHSAG

>163|CORE_REP|Org59_Gene2618#

MSTTGMGRSTARRMLTGPGLPEPAEQVDGRRLRARGFSDDARALLEHVWALMGMPCGKYLVVMLELWLPLEAAAGDLXKPFATEAAVAELKAMSAATVDRYLKPARERMRIKGISTTKPSPLLRNSITIHTCSDEAPKVPGVIEADTVAHCGPSLIGEFARTLTMTDLVTGWTENASIRNNAAKWILEGIKECQQRFPFPMTVFDSDCGGEFINHDVAGWLQARDIAQTRSRPYQKNDQAHVESKNNHVVRKHAFYWRYDTGEELELLNRLWPLVSLRCNFFTPTKKPVGYTSTVNGRRKRIYDKPATPWQRLQASGVLDAQQLSTVAARIEGFNPADLTRQINAIQMQLLDLAKTKTEALAXXPPHRPAIIATVNQPIGQGEVMQAPHALTMREAPATLRAHFYVRHLGCCCESCWAAPV

>164|CORE_REP|Org59_Gene1167#

MAEIVLDHVNKSYPDGHTAVRDLNLTIADGEFLILVGPSGCGKTTTLNMIAGLEDISSGELRIAGERVNEKAPKDRDIAMVFQSYALYPHMTVRQNIAFPLTLAKMRKADIAQKVSETAKILDLTNLLDRKPSQLSGGQRQRVAMGRAIVRHPKAFLXXEPLSNLDAKLRVQMRGEIAQLQRRLGTTTVYVTHDQTEAMTLGDRVVVMYGGIAQQIGTPEELYERPANLFVAGFIGSPAMNFFPARLTAIGLXLPFGEVTLAPEVQGVIAAHPKPENVIVGVRPEHIQDAALIDAYQRIRALTFQVKVNLVESLGADKYLYFTTESPAVHSVQLDELAEVEGESALHENQFVARVPRRVQGSHRAVGRVGFRYRQTCRLRRRLRCEPDHSAPRLMAASXHISPXHAXGFGAFCVCSPTRS

>165|CORE_REP|Org59_Gene1373#

MTASVNSLDLAAIRADFPILKRIMRGGNPLAYLDSGATSQRPLQVLDAEREFLTASNGAVHRGAHXLMEEATDAYEQGRADIVGGVIRRQXTRTSWCSPKMPPRRSTWCHMCWGTAVSSVTXGPGDVIVTTELEHHANLIPWQELARRTGATLRWXGVTDDGRIDLDSLYLDDRVKVVAFTHHSNVTGVLTPVSELVSRAHQSGALTVLXXCQSVPHQPVDLHELGVDFAAFSGHKMLGPNGIGVLYGRRELLAQMPPFLTGGSMIETVTXEGATYAPAPQRFEAGTPMTSQVVGLAAAARYLGAIGMAAVEAHERELVAAAIEGLSGIDGVRILGPTSMRDRGSPVAFVVEGVHAHDVGQVLDDGGVAVRVGHHCALPLHRRFGLAATARASFAVYNTADEVDRLVAGVRRSRHFFGRA

>166|CORE_REP|Org137_Gene875#

MRNSNRGPAFLILFATLMAAAGDGVSIVAFPWLVLQREGSAGQASIVASATMLPLLFATLVAGTAVDYFGRRRVSMVADALSGAAVAGVPLVAWGYGGDAVNVLVLAVLAALAAAFGPAGMTARDSMLPEAAARAGWSLDRINGAYEAILNLAFIVGPAIGGLMIATVGGITTMWITATAFGLSILAIAALQLEGAGKPHHTSRPQGLVSGIAEGLRFVWNLRVLRTLGMIDLTVTALYLPMESVLFPKYFTDHQQPVQLGWALMAIAGGGLVGALGYAVLAIRVPRRVTMSTAVLTLGLASMVIAFLPPLPVIMVLCAVVGLVYGPIQPIYNYVIQTRAAQHLRGRVVGVMTSLAYAAGPLGLLLAGPLTDAAGLHATFLALALPIVCTGLVAIRLPALRELDLAPQADIDRPVGSAQ

>167|CORE_REP|Org7_Gene3851#

MASGSGLCKTTSNFIWGQLLLLGEGIPDPGDIFNTGSSLFKQISDKMGLAIPGTNWIGQAAEAYLNQNIAQQLRAQVMGDLDKLTGNMISNQAKYVSDTRDVLRAMKKMIDGVYKVCKGLEKIPLLGHLWSWELAIPMSGIAMAVVGGALLYLTIMTLMNATNLRGILGRLIEMLTTLPKFPGLPGLPSLPDIIDGLWPPKLPDIPIPGLPDIPGLPDFKWPPTPGSPLFPDLPSFPGFPGFPEFPAIPGFPALPGLPSIPNLFPGLPGLGDLLPGVGDLGKLPTWTELAALPDFLGGFAGLPSLGFGNLLSFASLPTVGQVTATMGQLQQLVAAGGGPSQLASMGSQQAQLISSQAQQGGQQHATLVSDKKEDEEGVAEAERAPIDAGTAASQRGQEGTVLRSDTESPAGLCHSESKP

>168|CORE_REP|Org133_Gene1633#

MSGTVVAVPPRVARALDLLNFSLADVRDGLGPYLSIYLLLIHDWDQASIGFVMAVGGIAAIVAQTPIGALVDRTTAKRALVVAGAVLVTAAAVAMPLFAGLYSISVLQAVTGIASSVFAPALAAITLGAVGPQFFARRIGRNEAFNHAGNASAAGATGALAYFFGPVVVFWVLAGMALISVLATLRIPPDAVDHDLARGMDHAPGEPHPQPSRFTVLAHNRELVIFGAAVVAFHFANAAMLPLVGELLALHNRDEGTALMSSCIVAAQVVMVPVAYVVGTRADAWGRKPIFLVGFAVLTARGFLYTLSDNSYWLVGVQLLDGIGAGIFGALFPLVVQDVTHGTGHFNISLGAVTTATGIGAALSNLVAGWIVVVAGYDAAFMSLGALAGAGFLLYLVAMPETVDSDVRVRSRPTLGGK

>169|CORE_REP|Org59_Gene822#

MSHRSAGAIVGFVGMIRDRDGGRGVLRLEYSAHPSAAQVLADLVAEVAEESSGVRAVAASHRIGVLQVGEAALVAAVAADHRRAAXWXPVRTXWRXSSARLPVWKAPVLRGRYRRMGGFGLKSGLSPSADDVRLCERXQRIVAADVLGPNRLPQLLRIGDXRDLGRFXGRLGDVAGRRCVSWLGVGLGEFQIGGKANRAAWSCGGLIARRGLLVHRSGWCQVGGRGWRQFGGRGWRQVGGRGWRQVGGRGCQIGGWRQFGGSCREGFTQRRGQVVYGKFHGWCQVGGRGWRQVGGRGWRQVGGRGWCQVGGWCRVGGSCREGFTQRCGQVVYGKFHGWRDVGGRGWCQVGGWCRVGGSCREGFTQRCGQVVSGKFHGWRRVGGRRGQRCWFAVDRGRVQRSVHRCRSGKHFAGCCVR

>171|CORE_REP|Org98_Gene1547#

MSHVTAAPNVLAASAGELAAIGSTMRAANAAAAAPTAGVLAAGGDDVSAGIAALFGARAQAYQAISAQAALFHDRFVQILQEGAAAYAMAEAANALPLQKAQGVVSELAQDRTGGTGTGQSRGAGGFGGVGQAGGKGWDGGPIGNGQVGEQHGAGQLGSTDGNPGVAGAAHGSGVSASHGSGATGAAGVADPGGSGAGVGSAAGNGTGAGSADAVGGAGTGRDIVGSVRGDGGVGMASGDGGLSTGAAGASAEGGLMPGFGGAPWVGGHWGLGGEGHSGAIGGVGEQVAPAVATAPAVSPATTSAVAAESGSTPATKAQAMHATTNPGNAAHQGNPADPGNSARRADGGRDEQLLLLPLTSLRGLRHTLKKLSGLRARNGLLTASGDNASGSGRPWDRDQLLRALGLRPPGHE

>172|CORE_REP|Org145_Gene3004#

MKIRTLSGSVLEPPSAVRATPGTSMLKLEPGGSTIPKIPFIRPSFPGPAELAEDFVQIAQANWYTNFGPNERRFARALRDYLGPHLHVATLANGTLALLAALHVSFGAGTRDRYLLMPSFTFVGVAQAALWTGYRPWFIDIDANTWQPCVHSARAVIERFRDRIAGILLANVFGVGNPQISVWEELAAEWELPIVLDSAAGFGSTYADGERLGGRGACEIFSFHATKPFAVGEGGALVSRDPRLVEHAYKFQNFGLVQTRESIQLGMNGKLSEISAAIGLRQLVGLDRRLASRRKVLECYRTGMADAGVRFQDNANVASLCFASACCTSADHKAAVLGSLRRHAIEARDYYNPPQHRHPYFVTNAELVESTDLAVTADICSRIVSLPVHDHMAPDDVARVVAAVQEAEVRGE

>173|CORE_REP|Org19_Gene3437#

MSMPAKVSVLITVTGMDQPGVTSALFEVLAQHGVELLNVEQVVIRGRLTLGVLVSCPLDVADGTALRDDVAAAIHGVGLDVAIERSDDLPIIRQPSTHTIFVLGRPITAGAFSAVAREVAALGVNIDFIRGISDYPVTGLELRVSVPPGCVGPLQIALTKVAAEEHVDVAVEDYGLAWRTKRLIVFDVDSTLVQGEVIEMLAARAGAQGQVAAITEAAMRGELDFAESLQRRVATLAGLPATVIDDVAEQLELMPGARTTIRTLRRLGFRCGVVSGGFRRIIEPLARELMLDFVASNELEIVDGILTGRVVGPIVDRPGKAKALRDFASQYGVPMEQTVAVGDGANDIDMLGAAGLGIAFNAKPALREVADASLSHPYLDTVLFLLGVTRGEIEAADAGDCGVRRVEIPAD

>174|CORE_REP|Org1_Gene1211#

MSRAGDDAERSDEEERRMSVMNGREVARESRDAQVFEFGTAPGSAVVKIPVQGGPIGGIAISRDGSLLVVTNNGTDTVSVVGTDTCRVTQTVTSVNEPFAIAMGNAEANRAYVSTVSSAYDAIAVIDVATNTVLGTHPLALSVSDLTLSPDDKYLYVSRNGTRGADVAVLDTTTGALIDVVDVSQAPGTTTQCVRMSPDGSVLYVGANGPSGGLLVVITTRAQSDGGRIGSRSRSRQKSSKPRGNQAAAGLRVVATIDIGSSVRDVALSPDGAIAYVASCGSDFGAVVDVIDTRTHQITSSRAISEIGGLVTRVSVSGDADRAYLVSEDRVTVLCTRTHDVIGTIRTGQPSCVVESPDGKYLYIADYSGTITRTAVASTIVSGTEQLALQRRGSMQWFSPELQQYAPALA

>175|CORE_REP|Org59_Gene662#

MNAHVTSREGVNEFDDGIVIVGGGLAAARTAEQLRRAGYSGRLTIVSDEVHLPYDRPPLSKEVLRSEVDDVALKPREFYDEKDIALRLGSAAVSLDTGEQTVTLADVTVLGYDELVIATGLVPRRIPSLPDLDGIRVLRSFDESMALRKHASAARHAVVVGAGFIGCEVAASLRGLGVDVVLVEPQPAPLASVLGEQIGQLVTRLHRDEGVDVRTGVTVAEVRGKGHVDAVVLTDGTELPADLVVVGIGSTPATEWLEGSGVEVDNGVICDKAGRTSAPNVWALGDVASWRDPMGHQARVEHWSNVADQARVVVPAMLGTDVPTGVVVPYFWSDQYDVKIQCLGXPXVHRRCASGRGRRAQVPCLLRARWRAGWRGRWRDGRQGHEGARXDRRGRAHRRSVRPNSGLELT

>176|CORE_REP|Org69_Gene3571#

MDVSYPPEAEAFRDRIREFVAEHLPPGWPGPGALPPHEREEFARHWRRALAGAGLVAVSWPTEYGGGGLSPMEQVVLAEEFARAGAPERAENDLLGIDLLGNTLIALGSEAQKRHFLPRILSGEHRWCQGFSEPEAGSDLASVRTRGVLDGDEWVINGHKIWTSAGTTANWTFLLARTDPSAAKHRGLSFLLVPMDQPGVVVRPIVNAAGHSSFSEVFLTDARTSAGNVVGRVGDGWSTAMTLLGFERGSHIATAAIDFERDLQRLCELARDRGLHTDPRVRDGLAWCYARVQIMRYRGYRDLTLALTGRPPGAEAAITKVIWSEYFRRYTDLAVEILGLEALGPRGPGNGGARLVPEAGTPNSPACWMDELLYARAATIYAGSSQIQRNVIGKRLLGLPKEPRPEVLC

>177|CORE_REP|Org81_Gene2951#

MALPQSALSELLDAFRTGDGVDLIRDAVRLVLQELSELEATERIGAARYERSDTRVTDRNGARSRVLSTQAGDVELRIPKLRKGSFFPAILEPRRRIDQALYAVVMEAYVHGISTRAVDDLVEAMGVETGISKSEVSRICAGLDEIVGAFRTRTLGHIEFPYVYLDATYLNVRNGTGQVVSMAVIVASGIAADGSREILGLDVGDSEDETFWRGFLTSLKGRGLGGVRLVISDQHAGLVKALKRCFQGAGHQRCRVHFARNLLAHVPKDKADMVASMFRMIFSAPDAEAVHATWEGVRDRLAASFPKIGPLMDDARAEVLAFTAFPKAHWQKIWSTNPLERINKEIKRRSRVVGIFPNPAAVIRLVGAVLADMHDEWQASERRYLSEASMALLYPDSDNAVVAAISGGQ

>178|CORE_REP|Org2_Gene843#

MRPLWRHPDAADRASLSKSRNLSPVVNFTKELSPLFEERPCMTYTGSIRCEGDTWDLASSVGATATMVAAARAMATRAANPLINDQFAEPLVRAVGVDVLTRLASGELTASDIDDPERPNASMVRMAEHHAVRTKFFDEFFMDATRAGIRQVVILASGLDSRAYRLAWPAQTVVYEIDQPQVMEFKTRTLAELGATPTADRRVVTADLRADWPTALGAAGFDPTQPTAWSAEGLLRYLPPEAQDRLLDNVTALSVPDSRFATESIRNFKPHHEERMRERMTILANRWRAYGFDLDMNELVYFGDRNEPASYLSDNGWLLTEIKSQDLLTANGFQPFEDEEVPLPDFFYVSARLQRKHRQYPAHRKPAPSWRHTACPVNELSKSAAYTMTRSDAHQASTTAPPPPGLTG

>179|CORE_REP|Org74_Gene2841#

MIPNPLEELTLEQLRSQRTSMKWRAHPADVLPLWVAEMDVKLPPTVADALRRAIDDGDTGYPYGTEYAEAVREFACQRWQWHDLEVSRTAIVPDVMLGIVEVLRLITDRGDPVIVNSPVYAPFYAFVSHDGRRVIPAPLRGDGRIDLDALQEAFSSARASSGSSGNVAYLLCNPHNPTGSVHTADELRGIAERAQRFGVRVVSDEIHAPLIPSGARFTPYLSVPGAENAFALMSASKAWNLGGLKAALAIAGREAAADLARMPEEVGHGPSHLGVIAHTAAFRTGGNWLDALLRGLDHNRTLLGALVDEHLPGVQYRWPQGTYLAWLDCRELGFDDAASDEMTEGLAVVSDLSGPARWFLDHARVALSSGHVFGIGGAGHVRINFATSRAILIEAVSRMSRSLLERR

>180|CORE_REP|Org145_Gene1996#

MVKPERRTKTDIAAAATIAVVVAVAASLIWWTSDARATISRPAAVAVPTPAPAREVPTSLKQLWTAASPATRVPVVVGGTVATGDGRQVDGRDPATGESLWSYARDTDLCGVTWVYHYAVAVYRYDRGCGQVSTIDGSTGRRGAARSGYADPRVRLFSDGTTVLSAGDTRLELWRSDMVRMLAYGEIDARVKPSNRGLQSGCTLESAAASSAAVSVLEACTNQADLRLVLLRPGKEDDEPIQRIVPEPGVRPGSGARVLVVSQNNTAVYLPARSGAQPRVDVIDETGATVSSTLLAKPPSTSAVASRTGNLVTWWTGDALLVFDAGNLTQRYTIAAGETTAPVGPGVMMAGQLLVPVTGGIGVYDPVSGANNRYIPVTRPPSTSAVIPAVSGSRVIEQRGDTLVALG

>181|CORE_REP|Org117_Gene4060#

MSRAFIIDPTISAIDGLYDLLGIGIPNQGGILYSSLEYFEKALEELAAAFPGDGWLGSAADKYAGKNRNHVNFFQELADLDRQLISLIHDQANAVQTTRDILEGAKKGLEFVRPVAVDLTYIPVVGHALSAAFQAPFCAGAMAVVGGALAYLVVKTLINATQLLKLLAKLAELVAAAIADIISDVADIIKGILGEVWEFITNALNGLKELWDKLTGWVTGLFSRGWSNLESFFAGVPGLTGATSGLSQVTGLFGAAGLSASSGLAHADSLASSASLPALAGIGGGSGFGGLPSLAQVHAASTRQALRPRADGPVGAAAEQVGGQSQLVSAQGSQGMGGPVGMGGMHPSSGASKGTTTKKYSEGAAAGTEDAERAPVEADAGGGQKVLVRNVVQRHGEPNPLLASA

>183|CORE_REP|Org59_Gene3300#

MRANQPVFRDRNGLAAASTYQAVIDAERQPELFSNAGGIRPDQPALPMMIDMDDPAHLLRRKLVNAGFTRKRVKDXXARXXAALCDXLIDAVCERGECDFVRDLAAPLPMAVIGDMLGVRPEQRDMFLRWSDDLVTFLSSHVSQEDFQITMDAFAAYNDFXRATIAARRADPTDDLVSVLVSSEVDGERLSDDELVMETLLILIGGDETTRHTLSGGTEQLLRNRDQWDLLQRDPSLLPGAIEEMLRWTAPVKNMCRVLTADTEFHGTALCAGEKMMLLFESANFDEXVFCEPEKFDVQRNPNSHLAFGFGTHFCLGNQLARLELSLMTERVLRRLPDLRLVADDSVLPLRPXBFVSGLEXHAGGVHAXPAAGLSHAERELRAEYRPNLRREFTFGAARPLTPAR

>184|CORE_REP|Org19_Gene1581#

MMFVTGIVLFALAILISVALHECGHMWVARRTGMKVRRYFVGFGPTLWSTRRGETEYGVKAVPLGGFCDIAGMTPVEELDPDERDRAMYKQATWKRVAVLFAGPGMNLAICLVLIYAIALVWGLPNLHPPTRAVIGETGCVAQEVSQGKLEQCTGPGPAALAGIRSGDVVVKVGDTPVSSFDEMAAAVRKSHGSVPIVVERDGTAIVTYVDIESTQRWIPNGQGGELQPATVGAIGVGAARVGPVRYGVFSAMPATFAFTGDLTVEVGKALAALPTKVGALVRAIGGGQRDPQTPISVVGASIIGGDTVDHGLWVAFWFFLAQLNLILAAINLLPLLPFDGGHIAVAVFERIRNMVRSARGKVAAAPVNYLKLLPATYVVLVLVVGYMLLTVTADLVNPIRLFQ

>185|CORE_REP|Org68_Gene851#

MREVPHVLGIVLAGGEGKRLYPLTADRAKPAVPFGGAYRLIDFVLSNLVNARYLRICVLTQYKSHSLDRHISQNWRLSGLAGEYITPVPAQQRLGPRWYTGSADAIYQSLNLIYDEDPDYIVVFGADHVYRMDPEQMVRFHIDSGAGATVAGIRVPRENATAFGCIDADDSGRIRSFVEKPLEPPGTPDDPDTTFVSMGNYIFTTKVLIDAIRADADDDHSDHDMGGDIVPRLVADGMAAVYDFSDNEVPGATDRDRAYWRDVGTLDAFYDAHMDLVSVHPVFNLYNKRWPIRGESENLAPAKFVNGGSAQESVVGAGSIISAASVRNSVLSSNVVVDDGAIVEGSVIMPGTRVGRGAVVRHAILDKNVVVGPGEMVGVDLEKDRERFAISAGGVVAVGKGVWI

>186|CORE_REP|Org118_Gene1238#

MQYGLEVSSDVAGVAGGLLALSYRGAGVPLRELALVGLTAAIITYFATGPVRMLASRLGAVAYPRERDVHVTPTPRMGGLAMFLGIVGAVFLASQLPALTRGFVYSTGMPAVLVAGAVIMGIGLIDDRWGLDALTKFAGQITAASVLVTMGVAWSVLYIPVGGVGTIVLDQASSILLTLALTVSIVNAMNFVDGLDGLAAGLGLITALAICMFSVGLLRDHGGDVLYYPPAVISVVLAGACLGFLPHNFHRAKIFMGDSGSMLIGLMLAAASTTAAGPISQNAYGARDVFALLSPFLLVVAVMFVPMLDLLLAIVRRTRAGRSAFSPDKMHLHHRLLQIGHSHRRVVLIIYLWVGIVAFGAASSIFFNPRDTAAVMLGAIVVAGVATLIPLLRRGDDYYDPDLD

>187|CORE_REP|Org40_Gene441#

MRCGVSAGSANGKPNRWTLRCGVSAGHRGSVFLLAVLLAPVVLTSCTWRGIANVPLPVGRGMGPDRMTIYVQMPDTLALNTNSRVRVADVWVGTVRDISLRNWIATLTLELEPTVRLPANATAKIGQTSLLGTQHVELAAPPIPSPQPLKSGDTIGLKNSSAYPTVERTLASVALILTGGGIVNLDVIQTEILNILDGHAGQIREFLERLATFTAELNNQRGDLTRAIDSTNQLLTIIANRNDTLDRVLTDVPPLIEHFADTGQLFADATESLGRFSEVANRALAATRPNLHQTLQSLQRPLRQLERASPYVVGALKLGLTAPFNIDEVPNVIRGDYVNVSATFDVTLSALDNALLSGTGISGMLRALEQAWGRDPDTMIPDVRYTPNPNDAPGGPLVERAE

>188|CORE_REP|Org56_Gene2664#

MNLDGNQASIREVCDAGLLSGAVTMVWQREKLLQVNEIGYRDIDAGVPMQRDTLFRIASMTKPVTVAAAMSLVDEGKLALRDPITRWAPELCKVAVLDDAAGPLDRTHPARRAILIEDLLTHTSGLAYGFSVSGPISRAYQRLPFGQGPDVWLAALATLPLVHQPGDRVTYSHAIDVLGVIVSRIEDAPLYQIIDERVLGPAGMTDTGFYVSADAQRRAATMYRLDEQDRLRHDVMGPPHVTPPSFCNAGGGLWSTADDYLRFVRMLLGDGTVDGVRVLSPESVRLMRTDRLTDEQKRHSFLGAPFWVGRGFGLNLSVVTDPAKSRPLFGPGGLGTFSWPGAYGTWWQADPSADLILLYLIQHCPDLSVDAAAAVAGNPSLAKLRTAQPKFVRRTYRALGL

>189|CORE_REP|Org60_Gene2893#

MTSTSIPTFPFDRPVPTEPSPMLSELRNSCPVAPIELPSGHTAWLVTRFDDVKGVLSDKRFSCRAAAHPSSPPFVPFVQLCPSLLSIDGPQHTAARRLLAQGLNPGFIARMRPVVQQIVDNALDDLAAAEPPVDFQEIVSVPIGEQLMAKLLGVEPETVHELAAHVDAAMSVCEIGDEEVSRRWSALCTMVIDILHRKLAEPGDDLLSTIAQANRQQSTMTDEQVVGMLLTVVIGGVDTPIAVITNGLASLLHHRDQYERLVEDPGRVARAVEEIVRFNPATEIEHLRVVTEDVVIAGTALSAGSPAFTSITSANRDSDQFLDPDEFDVERNPNEHIAFGYGPHACPASAYSRMCLTTFFTSLTQRFPQLQLARPFEDLERRGKGLHSVGIKELLVTWPT

>190|CORE_REP|Org69_Gene2725#

MVEAGTRDPLESALLDSRYLVQAKIASGGTSTVYRGLDVRLDRPVALKVMDSRYAGDEQFLTRFRLEARAVARLNNRALVAVYDQGKDGRHPFLVMELIEGGTLRELLIERGPMPPHAVVAVLRPVLGGLAAAHRAGLVHRDVKPENILISDDGDVKLADFGLVRAVAAASITSTGVILGTAAYLSPEQVRDGNADPRSDVYSVGVLVYELLTGHTPFTGDSALSIAYQRLDADVPRASAVIDGVPPQFDELVACATARNPADRYADAIAMGADLEAIAEELALPEFRVPAPRNSAQHRSAALYRSRITQQGQLGAKPVHHPTRQLTRQPGDCSEPASGSEPEHEPITGQFAGIAIEEFIWARQHARRMVLVWVSVVLAITGLVASAAWTIGSNLSGLL

>193|CORE_REP|Org59_Gene1577#

MTLDVPVNQGHVPPGSVACCLVGVTAVADGIAGHSLSNFGALPPEINSGRMYSGPGSGPLMAAAAAWDGLAAELSSAATGYGAAISELTNMRWWSGPASDSMVAAVLPFVGWLSTTATLAEQAAMQARAAAAAFEAAFAMTVPPPAIAANRTLLMTLVDTNWFGQNTPAIATTESQYAEMWAQDAAAMYGYASAAAPATVLTPFAPPPQTTNATGLVGHATAVAALRGQHSWAAAIPWSDIQKYWMMFLGALATAEGFIYDSGGLTLNALQFVGGMLWXXRXXKKPVRPMAXXXAGGAAGWSAWSQLGAGPVAASATLAAKIGPMSVPPGWSAPPATPQAQTVARSIPGIRSAAEAAETSVLLRGAPTPGRSRAAHMGRRYGRRLTVMADRPNVG

>194|CORE_REP|Org149_Gene3340#

MPTSNPAKPLDGFRVLDFTQNVAGPLAGQVLVDLGAEVIKVEAPGGEAARQITSVLPGRPPLATYFLPNNRGKKSVTVDLTTEQAKQQMLRLADTADVVLEAFRPGTMEKLGLGPDDLRSRNPNLIYARLTAYGGNGPHGSRPGIDLVVAAEAGMTTGMPTPEGKPQIIPFQLVDNASGHMLAQAVLAALLHRERNGVADVVQVAMYDVAVGLQANQLMMHLNRAASDQPKPEPAPKAKRRKGVGFATQPSDAFRTADGYIVISAYVPKHWQKLCYLIGRPDLVEDQRFAEQRSRSINYAELTAELELALASKTATEWVQLLQANGLMACLAHTWKQVVDTPLFAENDLTLEVGRGADTITVIRTPARYASFRAVVTDPPPTAGEHNAVFLARP

>195|CORE_REP|Org114_Gene940#

MNVSAESGAPRRAGQRHEVGLAQLPPAPPTTVAVIEGLATGTPRRVVNQSDAADRVAELFLDPGQRERIPRVYQKSRITTRRMAVDPLDAKFDVFRREPATIRDRMHLFYEHAVPLAVDVSKRALAGLPYRAAEIGLLVLATSTGFIAPGVDVAIVKELGLSPSISRVVVNFMGCAAAMNALGTATNYVRAHPAMKALVVCIELCSVNAVFADDINDVVIHSLFGDGCAALVIGASQVQEKLEPGKVVVRSSFSQLLDNTEDGIVLGVNHNGITCELSENLPGYIFSGVAPVVTEMLWDNGLQISDIDLWAIHPGGPKIIEQSVRSLGISAELAAQSWDVLARFGNMLSVSLIFVLETMVQQAESAKAISTGVAFAFGPGVTVEGMLFDIIRR

>196|CORE_REP|Org93_Gene4068#

MPAPDPMRGDPPHPAPPRLRSPLXPTSGDPLHPAPPRLRSPLDPTSGDPLHPAPPRLRSPLDPTSGDPLHPAPPRLRSPLVLLDGASMWFRSFFGVPSSITAPDGRPVNAVRGFIDSMAVVITQQRPNRLAVCLDLDWRPQFRVDLIPSYKAHRVAEPEPNGQPDVEEVPDELTPQVDMIMELLDAFGIAMAGAPGFEADDVLGTLATRERRDPVIVVSGDRDLLQVVADDPVPVRVLYLGRGLAKATLFGPAEVAERYGLPAHRAGAAYAELALLRGDPSDGLPGVPGVGEKTAATLLARHGSLDQIMAAADDRKTTMAKGLRTKLLAASAYIKAADRVVRVATDAPVTLSTPTDRLPLVAADPERTAELATRFGVESSIARLQKALDTLPG

>198|CORE_REP|Org7_Gene1049#

MTTPGEDHAGSFYLPRLEYSTLPMAVDRGVGWKTLRDAGPVVFMNGWYYLTRREDVLAALRNPKVFSSRKALQPPGNPLPVVPLAFDPPEHTRYRRILQPYFSPAALSKALPSLRRHTVAMIDAIAGRGECEAMADLANLFPFQLFLVLYGLPLEDRDRLIGWKDAVIAMSDRPHPTEADVAAARELLEYLTAMVAERRRNPGPDVLSQVQIGEDPLSEIEVLGLSHLLILAGLDTVTAAVGFSLLELARRPQLRAMLRDNPKQIRVFIEEIVRLEPSAPVAPRVTTEPVTVGGMTLPAGSPVRLCMAAVNRDGSDAMSTDELVMDGKVHRHWGFGGGPHRCLGSHLARLELTLLVGEWLNQIPDFELAPDYAPEIRFPSKSFALKNLPLRWS

>199|CORE_REP|Org62_Gene1151#

MRMSALLSRNTSRPGLIGIARVDRNIDRLLRRVCPGDIVVLDVLDLDRITADALVEAEIAAVVNASSSVSGRYPNLGPEVLVTNGVTLIDETGPEIFKKVKDGAKVRLYEGGVYAGDRRLIRGTERTDHDIADLMREAKSGLVAHLEAFAGNTIEFIRSESPLLIDGIGIPDVDVDLRRRHVVIVADEPSGPDDLKSLKPFIKEYQPVLVGVGTGADVLRKAGYRPQLIVGDPDQISTEVLKCGAQVVLPADADGHAPGLERIQDLGVGAMTFPAAGSATDLALLLADHHGAALLVTAGHAANIETFFDRTRVQSNPSTFLTRLRVGEKLVDAKAVATLYRNHISGGAIALLALTMLIAIIVALWVSRTDGVVLHWIIDYWNRFSLWVQHLVS

>200|CORE_REP|Org59_Gene1665#

MDQQSTRTDITVNVDGFWMLQALLDIRHVAPELRCRPYVSTDSNDWLNEHPGMAVMREQGIVVNDAVNEQVAARMKVLAAPDLEVVALLSRGKLLYGVIDDENQPPGSRDIPDNEFRVVLARRGQHWVSAVRVGNDITVDDVTVSDSASIAALVMDGLESIXXAXPAAINAVNVPMEEMLEATKSWQESGFNVFSGGDLRRMGISAATVAALGQALSDPAAEVAVYARQYRDDAKXPSASXLSLKDGSGGRIALYQQARTAGXRRGXAGYLPXYPAVGASRSEDRFGYTALRRVENTQQSMTPGRETRSTTTNLSIRYNPDTYRANCSRIDCNTARQGQPQRFGREARXXKSELXEPQLPVGYRASVPTPTELPAPLKPRCNTFAMAGGTGR

>201|CORE_REP|Org121_Gene3954#

MTGRAATPGVIREFVGLPSRTAGRAAAGGHPCQGLYHHSVGRKPKVALIAAHYQIDFSEHYLAEYMAIRGIGFLGWNTRFRGFESSFLLDHALVDIGVGVRWLREVQGVETVVLLGNSGGGSLMAAYQSQAVDPNVTPLDGMRPAAGVTELPAADAYVAAAAHPGRPDVLTAWMDAAVIDENDPVATDPELDLFDERNGPPYSPEFISRYRSAQVKRNHTITDWAESELKRVRAAGFSDRPFSVMRTWADPRMVDPSIEPTKRRPNQCYAGTPVKANRSAHGIAAACTLRGWLGMWSLRVAQTRAAPHLARITCPALVLNAEADTGIFPSDAQQIYDGLASSDKTQVSIDTDHYFTTPGARSEQADTIAKWIASGGAECSHPFRRPSASQR

>202|CORE_REP|Org20_Gene3603#

MHAVTRPTLREAVARLAPGTGLRDGLERILRGRTGALIVLGHDENVEAICDGGFSLDVRYAATRLRELCKMDGAVVLSTDGSRIVRANVQLVPDPSIPTDESGTRHRSAERAAIQTGYPVISVSHSMNIVTVYVRGERHVLTDSATILSRANQAIATLERYKTRLDEVSRQLSRAEIEDFVTLRDVMTVVQRLELVRRIGLVIDYDVVELGTDGRQLRLQLDELLGGNDTARELIVRDYHANPEPPSTGQINATLDELDALSDGDLLDFTALAKVFGYPTTTEAQDSALSPRGYRAMAGIPRLQFAHADLLVRAFGTLQGLLAASAGDLQSVDGIGAMWCPSCARGVVTAGGIDHQRSIIIRLARETPAEAPALDPERVPARAAAAADSG

>203|CORE_REP|Org59_Gene827#

MELCGXESGXARTPDARQAAVEAAGQARDELAGEAPSLAVLLGSRAHTDRAADVLSAVLQMIDPPALVGCIAQAIVAGRHEIEDEPAVVVWLASGLAAETFQLDFVRTGSGALITGYRFDRTARDLHLLLPXXXTFPSNLLIEHPNTFDLPGTAVVGGVVSGGRRRGDTRLFRDHDVLTSGVVGVRLPGMRGVPVVSQGCRPIGYPYIVTGADGILITELGGRPPLQRLREIVEGLSPDERALVSHGLQIGIVVDEHLAAPGQGDFVIRGLLGADPSTGSIEIDEVVQVGATMQFQVRDAAGADKDLRLTVERAAARLPGRAAGALLFTCNGRGRRMFGVADHDASTIEELLGGIPLAGFFAAGEIGPIAGRNALHGFTASMALFVDDME

>205|CORE_REP|Org20_Gene1241#

MRVAMLTREYPPEVYGGAGVHVTELVAYLRRLCAVDVHCMGAPRPGAFAYRPDPRLGSANAALSTLSADLVMANAASAATVVHSHTWYTALAGHLAAILYDIPHVLTAHSLEPLRPWKKEQLGGGYQVSTWVEQTAVLAANAVIAVSSAMRNDMLRVYPSLDPNLVHVIRNGIDTETWYPAGPARTGSVLAELGVDPNRPMAVFVGRITRQKGVVHLVTAAHRFRSDVQLVLCAGAADTPEVADEVRVAVAELARNRTGVFWIQDRLTIGQLREILSAATVFVCPSVYEPLGIVNLEAMACATAVVASDVGGIPEVVADGITGSLVHYDADDATGYQARLAEAVNALVADPATAERYGHAGRQRCIQEFSWAYIAEQTLDIYRKVCA

>206|CORE_REP|Org20_Gene1915#

MLGLLAATLLLGGCTGQHTTRTAASTTYTPHIKASSQDVLDGAINADEPGCSAAVGVEGKVIWSGVRGIADLASGAKITTDTVFDIASVSKQFTATAILLLVEAGKLTLDDPISQYVPELPDWAQTVTVEQLMHQTSGIPDYVALLAARGYQVSDRTIEAEARQALAAAPELQFKPGTRFDYSNSNYLLLGEIVHRASGQPLPEFLSAEIFQPLGLAMVVDPVGKVPNKAVSYEKGTGGNRSEYRVGNPAWEQIGDGGIQTTPSQLARWADNYRTGSVGGLKLLEAQLAGAVETEPGGGDRYGAGIVSRADGMTTRAPGPDSSRHSTSAVTDGLRWPSAATPTSRTRWPWPMRWGAFGCSGATAVGRRYPGCNHSRYGATTVTPHN

>207|CORE_REP|Org140_Gene3432#

MSRVLLVTNDFPPRRGGIQSYLGQFVGRLVGSRAHAMTVYAPQWKGADAFDDAARAAGYRVVRHPSTVMLPGPTVDVRMRRLIAEHDIETVWFGAAAPLALLAPRARLAGASRVLASTHGHEVGWSMLPVARSVLRRIGDGTDVVTFVSSYTRSRFASAFGPAASLEYLPPGVDTDRFRPDPAARAELRKRYRLGERPTVVCLSRLVPRKGQDTLVTALPSIRRRVDGAALVIVGGGPYLETLRKLAHDCGVADHVTFTGGVATDELPAHHALADVFAMPCRTRGAGMDVEGLGIVFLEASAAGVPVIAGNSGGAPETVQHNKTGLVVDGRSVDRVADAVAELLIDRDRAVAMGAAGREWVTAQWRWDMLAAKLADFLRGDDAAR

>208|CORE_REP|Org71_Gene3018#

MYVRHLGLRDFRSWACVDLELHPGRTVFVGPNGYGKTNLIEALWYSTTLGSHRVSADLPLIRVGTDRAVISTIVVNDGRECAVDLEIATGRVNKARLNRSSVRSTRDVVGVLRAVLFAPEDLGLVRGDPADRRRYLDDLAIVRRPAIAAVRAEYERVLRQRTALLKSVPGARYRGDRGVFDTLEVWDSRLAEHGAELVAARIDLVNQLAPEVKKAYQLLAPESRSASIGYRASMDVTGPSEQSDTDRQLLAARLLAALAARRDAELERGVCLVGPHRDDLILRLGDQPAKGFASHGEAWSLAVALRLAAYQLLRVDGGEPVLLLDDVFAELDVMRRRALATAAESAEQVLVTAAVLEDIPAGWDARRVHIDVRADDTGSMSVVLP

>209|CORE_REP|Org59_Gene2935#

MDFALLPPEVNSARMYTGPGAGSLLAAAGGWDSLAAELATTAEAYGSVLSGLAALHWRGPAAESMAVTAAPYIGWLYTTAEKTQQTAIQARAAALAFEQAYAMXLPPPVVAANRIQLLALIATNFFGQNTAAIAATEAQYAEMWAQDAAAMYGYATASXAXALLTPGXXXPRQTTNPAGLTAQAAAVSQATDPLSLLIETVTQALQALTIPSFIPEDFTFLDAIFAGYATVGVTQDVESFVAGTIGAESNLGLLNVGDENPAEVTPGDFGIGELVSATSPGGGVSASGAGGAASVGNTVLASVGRANSIGQLSVPPSWAAPSTRPVSALSPAGLTTLPGTDVAEHGMPGVPGVPVAXGRASGVLPRYGVRLTVMAHPPAAGXPGA

>210|CORE_REP|Org1_Gene2839#

MRCENLDTVLGLSITPTTLGWVLAEGHGADGAILDRNELELHSGRNAQAIHTAEQLAAEVLLAHEVAAAGDHRLRVIGVTWNAEASAQAALLVESLTGAGFDNVVPVRRLRAIETLAQAIAPVIGYEQIAVCVLEHESATVVMVDTHDGKTQIAVKHVCRGLSGLTSWLTGMFGRDAWRPAGVVVVGSDSEVSEFSWQLERVLPVPVFAQTMAQVTVARGAALAAAQSTEFTDAQLVADSVSQPTVAPRRSRHYAGAAAALAAAAVTFVASLSLAVGIQLAPHNDTGTAKHGAHKPTPRIAKAVAPAVPPPPTVTPPVPARAPRPAAQHEPPARVTSGEALTEPNPPEEQPNASAPQQDRNDSQPITRVLEHIPGAYGDSAPPAE

>211|CORE_REP|Org59_Gene1179#

MTLPKERAAQGGLERIAHVDRVASLTGIRAVAALLVVGTHAAYTTGKYTHGYWGLMSSRMEIGVPIFFVLSGFLLFRPWVKXXXXRRPPAVVEPLCVAPGPADHARLHRHRSVGLPRLSLPHGGGXXPXXTXXGXFRNLTLTQIYTDGYLGAFLHQGLTQMWSLAVEVAFYLALPALAYLLLVLVCRRRWQPRLLLATMAGXTMISPAWLILVHNTHWMPDGARLWLPTYLAWFVGGMMLAVLAAMGVRCYAFVAIPLAVICYFIVSTPIAGAPTTSPTALAEALVKTAFYAVIAVLAVAPLALGDQGWYAQLLASRPMVFLGEISYEIFLIHLVTMEIAMVDVLGYRVYTSSMVNLCLVTLVLTIPLAWLLHRFTRVQGDRPS

>212|CORE_REP|Org59_Gene3118#

MTVLGADAVVIDGRICRPGWVHTADGRILSGGAGAPPMPADAEFPDAIVVPGFVDMHVHGGGGASFADGNAADIARAAEFHLRHGTTTTLASLVTAGPAELLSAVGALAEATRDGVVAGIHLEGPWLSPARCGAHDHTRMRAPDPAEIESVLAAADGAVRMVTLAPELPGSDAAIRRFRDAEVVVAVGHTDATYTQTRHAIDLGADSRXPXVXXAXPPLDHRAPGPVLALLCDPRVTVEIIADGVHVHPAVVHAVIEAVGPDRVAVVTDAIAAAGCGDGAFRLGTMPIEVESSVARVAGASTLAGSTTTMDQLFRTVAGLGSKSDSAGDVALAAAVQVTSATPARALGLTGVGRLAAGYAANLVVLDRDLRVTAVMVNDDWRVG

>213|CORE_REP|Org7_Gene2676#

MRVCSAWVVRCSLVVSFLVPQCVWYARADPPAPAPRPILPPLAPGQVLRIGPTAGTGTPTGDYGIGATDLCEFVEFPSQLLQVCGDSFAGQGVGFGGWYAPVALHVDTESIDDPAGVRYTGVTGVGTPLLADPTPPGDSQLPAGVVQINRRNYLMVTTTKDLQPQNSRLVRAEAARGGWQTVSGSRRNAAYQDGRQTQISGYYDPVPTPDSPTGWVYIVADSFTRGEPAVLYRATPESFTDRSRWQGWAGGPDGGWNKPPTPLWPDQLGEMSIRQIDGQTVLSYFNASTGNMEVRVAHHPTSLGAAPVTTVVRHDEWPEPAESLPPPYDNRLAQPYGGYISPGSTIDELRIFVSQWDTRARQNGPYRVIQFAVNPFKPWSDP

>214|CORE_REP|Org118_Gene1514#

MYADRDLPGAGGLAVRVIPCLDVDDGRVVKGVNFENLRDAGDPVELAAVYDAEGADELTFLDVTASSSGRATMLEVVRRTAEQVFIPLTVGGGVRTVADVDSLLRAGADKVAVNTAAIACPDLLADMARQFGSQCIVLSVDARTVPVGSAPTPSGWEVTTHGGRRGTGMDAVQWAARGADLGVGEILLNSMDADGTKAGFDLALLRAVRAAVTVPVIASGGAGAVEHFAPAVAAGADAVLAASVFHFRELTIGQVKAALAAEGITVPMTLDPKIAARLKRNADGLVTAVVQERGSGDVLMVAWMNDEALARTLQTREATYYSRSRAEQWVKGATSGHTQHVHSVRLDCDGDAVLLTVDQVGGACHIGDHSCFDAAVLLEPDD

>215|CORE_REP|Org78_Gene1937#

MGGLRFGFVDALVHSRLPPTLPARSSMAAATVMGADSYWVGDHLNALVPRSIATSEYLGIAAKFVPKIDANYEPWTMLGNLAFGLPSRLRLGVCVTDAGRRNPAVTAQAAATLHLLTRGRAILGIGVGEREGNEPYGVEWTKPVARFEEALATIRALWNSNGELISRESPYFPLHNALFDLPPYRGKWPEIWVAAHGPRMLRATGRYADAWIPIVVVRPSDYSRALEAVRSAASDAGRDPMSITPAAVRGIITGRNRDDVEEALESVVVKMTALGVPGEAWARHGVEHPMGADFSGVQDIIPQTMDKQTVLSYAAKVPAALMKEVVFSGTPDEVIDQVAEWRDHGLRYVVLINGSLVNPSLRKTVTAVLPHAKVLRGLKKL

>216|CORE_REP|Org60_Gene4003#

MTRSGHPVTLDDLPLRADLRGKAPYGAPQLAVPVRLNTNENPHPPTRALVDDVVRSVREAAIDLHRYPDRDAVALRADLAGYLTAQTGIQLGVENIWAANGSNEILQQLLQAFGGPGRSAIGFVPSYSMHPIISDGTHTEWIEASRANDFGLDVDVAVAAVVDRKPDVVFIASPNNPSGQSVSLPDLCKLLEVAPGIAIVDEAYGEFSSQPSAVSLVEEYPSKLVVTRTMSKAFAFAGGRLGYLIATPAVIDAMLLVRLPYHLSSVTQAAARAALRHSDDTLSSVAALIAERERVTTSLNDMGFRVIPSDANFVLFGEFADAPAAWRRYLEAGILIRDVGIPGYLRATTGLAEENDAFLRASARIATDLVPVTRSPVGAP

>217|CORE_REP|Org28_Gene1462#

MCQQGRPLGWDAVSDVPELIHGPLEDRHRELGASFAEFGGWLMPVSYAGTVSEHNATRTAVGLFDVSHLGKALVRGPGAAQFVNSALTNDLGRIGPGKAQYTLCCTESGGVIDDLIAYYVSDDEIFLVPNAANTAAVVGALQAAAPGGLSITNLHRSYAVLAVQGPCSTDVLTALGLPTEMDYMGYADASYSGVPVRVCRTGYTGEHGYELLPPWESAGVVFDALLAAVSAAGGEPAGLGARDTLRTEMGYPLHGHELSLDISPLQARCGWAVGWRKDAFFGRAALLAEKAAGPRRLLRGLRMVGRGVLRPGLAVLVGDETVGVTTSGTFSPTLQVGIGLALIDSDAGIEDGQQINVDVRGRAVECQVVCPPFVAVKTR

>218|CORE_REP|Org59_Gene1712#

MSALAFTILAVLLAGPTPALLARATWPLRAPRAAMVLWQAIALAAVLSSFSAGIAIASRLLMPGPDGRPTTSFVGAAGRLGWPLWAAYITVFALTVLVGARLAVAVVRVATATRRRRAHHRMVVDLVGVGHNGALAQPCARARDLRVLDVAQPLAYCLPGVRSRVVVSEGTLTALADAEVAAILTHERAHLRARHDLVLEAFTAVHAAFPRLVRSANALGAVQLLVELLADDAAVRAAGRTPLARALVACASGRAPSGALAVGGPSTVLRVRRLSGRGNSAVLSAAAYLAAAAVLVVPTVALAVPWLTQLQRLVHRLAKPGPTERQDMSSSESPAGIAQIGCHWPGRDGFQHRPKLRPARLHRGSAQSVGRQDRRAA

>219|CORE_REP|Org110_Gene2466#

MLLGMHQAGHVGTHERRAAATRRSALTAAGLAVVGAGVLGASACSPQKSPQPSSPRLPDNALITLGVAAGPPPTPSRVGISSVLKIGRDLYVIDCGLGSLNAFTNAGLQFDDLKAMFITHLHTDHIVDYYNFFLSGGFLAPPGRAPVLVYGPGPAGGLPPSEVGNPNPATVNPANPTPGLAAATEALHRAFAYTSNIFIRDYGIDNVADLVKVTEIGLPPGSDYRNRAPKMSPFSVASDDNVSVTATLVSHYDVYPAFGFRFDLKKSGVSVTFSGDTTKSDNLITLAQGTDILVHEAVFSLDTAYFGNAFPPNYLVNSHTSAEQVGEVAAAAKPKQLILSHYAPDDLPDSQWLDKIKKNYSGMTTIARDGQVFAL

>220|CORE_REP|Org118_Gene1360#

MHGLDSFPPGGALVVANHSGGMFPMDVPVFSVDFYDKFGYDRPVYTLSHDILFMGLTGDLFRRTGYIRATRENAAKALRSGGVVVVFPGGDYDAYRPTFAENVIDFNGRKGYVSTAVEAGVPIVPAVSIGGQESQLYLSRGTWLARRLGLKRLLRSDILPISFGFPFGFSAAIPPNLPLPAKIVMQVLDPINLTKQFGEDPDVDAVDEHGTVGDAASPQRPRRQAPLPDSGLSHAYQTSIRLDRHHAPGRVDRSAATRPLPADRRRHAPRRHGVYGGVRRCGPTLPGPPRPDRRTGHADLAPARRTRQRAGRGASGPASRTPQGRRHHVPQSSRLRRCAVGGQPDWRAHPVAQHLLRRSGTGRGGYPRRRRHCRL

>221|CORE_REP|Org1_Gene916#

MAVPAVSPQPILAPLTPAAIFLVATIGADGEATVHDALSKISGLVRAIGFRDPTKHLSVVVSIGSDAWDRLFAGPRPTELHPFVELTGPRHTAPATPGDLLFHIRAETMDVCFELAGRILKSMGDAVTVVDEVHGFRFFDNRDLLGFVDGTENPSGPIAIKATTIGDEDRNFAGSCYVHVQKYVHDMASWESLSVTEQERVIGRTKLDDIELDDNAKPANSHVALNVITDDDGTERKIVRHNMPFGEVGKGEYGTYFIGYSRTPTVTEQMLRNMFLGDPAGNTDRVLDFSTAVTGGLFFSPTIDFLDHPPPLPQAATPTLAAGSLSIGSLKGSPDEQSLPRFGTGHRSRLGGNRIGGGADVQATHRRAPGGRCQ

>223|CORE_REP|Org2_Gene4088#

MSSYYARRPLQSSGCSNSDSCWDGAPIEITESGPSVAGRLAALASRMTIKPLMTVGSYLSPLPLPLGFVDFACRVWRPGQGTVRTTINLPNATAQLVRAPGVRAADGAGRVVLYLHGGAFVMCGPNSHSRIVNALSGFAESPVLIVDYRLIPKHSLGMALDDCHDAYQWLRARGYRPEQIVLAGDSAGGYLALALAQRLQCDDEKPAAIVAISPLLQLAKGPKQDHPNIGTDAMFPARAFDALAAWVRAAAAKNMVDGRPEDLYEPLDHIESSLPPTLIHVSGSEVLLHDAQLGAGKLAAAGVCAEVRVWPGQAHLFQLATPLVPEATCSLRQIGQFIRDATADSSLSPVHRSRYVAGSPRAASRGAFGQSPI

>224|CORE_REP|Org4_Gene3976#

MSRAAGLPRLSWFAGLTWFAGGSTGAGCAAHPALAGLTAGARCPAYAAISASTARPAATALPAVAASTARPAATALPAVAASTARPAATAGTTPATGASGSARPTDAAGMADLARPGVVATHAVRTLGTTGSRAIGLCPCQPLDCPRSPQATLNLGSMGRSLDGPQWRRARVRLCGRWWRRSNTTRGASPRPPSTCRGDNVSMIELEVHQADVTKLELDAITNAANTRLRHAGGVAAAIARAGGPELQRESTEKAPIGLGEAVETTAGDMPARYVIHAATMELGGPTSGEIITAATAATLRKADELGCRSLALVAFGTGVGGFPLDDAARLMVGAVRRHRPGSLQRVVFAVHGDAAERAFSAAIQAGEDTARR

>225|CORE_REP|Org100_Gene2093#

MSAHVATLHPEPPFALCGPRGTLIARGVRTRYCDVRAAQAALRSGTAPILLGALPFDVSRPAALMVPDGVLRARKLPDWPTGPLPKVRVAAALPPPADYLTRIGRARDLLAAFDGPLHKVVLARAVQLTADAPLDARVLLRRLVVADPTAYGYLVDLTSAGNDDTGAALVGASPELLVARSGNRVMCKPFAGSAPRAADPKLDAANAAALASSAKNRHEHQLVVDTMRVALEPLCEDLTIPAQPQLNRTAAVWHLCTAITGRLRNISTTAIDLALALHPTPAVGGVPTKAATELIAELEGDRGFYAGAVGWCDGRGDGHWVVSIRCAQLSADRRAALAHAGGGIVAESDPDDELEETTTKFATILTALGVEQ

>226|CORE_REP|Org59_Gene452#

MQLTPHFGNVQAHYDLSDDFFRLFLDPTQTYSCAYFERDDMTLQEAQIAKIDLALGKLNLEPGMTLLDIGCGWGATMRRAIEKYDVNVVGLTLSENQAGHVQKMFDQMDTPRSRRVLLEGWEKFDEPVDRIVSIGAFEHFGHQRYHHFFEVTHRTLPADGKMLLHTIVRPTFKEGREKGLTLTHELVHFTKFILAEIFPGGWLPSIPTVHEYAEKVGFRVTAVQSLQLHYARTLDMWATALEANKDQAIAIZSQTVYBSLHEVPDRLREAVPPGLHRRRPVHTGKVTGQSALAXXRPVPGRXATPGVSSATPGTXSGGDGLXGQCELSHVADALAEEVLTSGQIVHVFVVNLLGLKSNGAVLVSLQIRRPDV

>227|CORE_REP|Org82_Gene2562#

MKFARSGAAVSLLAAGTLVLTACGGGTNSSSSGAGGTSGSVHCGGKKELHSSGSTAQENAMEQFVYAYVRSCPGYTLDYNANGSGAGVTQFLNNETDFAGSDVPLNPSTGQPDRSAERCGSPAWDLPTVFGPIAITYNIKGVSTLNLDGPTTAKIFNGTITVWNDPQIQALNSGTDLPPTPISVIFRSDKSGTSDNFQKYLDGASNGAWGKGASETFNGGVGVGASGNNGTSALLQTTDGSITYNEWSFAVGKQLNMAQIITSAGPDPVAITTESVGKTIAGAKIMGQGNDLVLDTSSFYRPTQPGSYPIVLATYEIVCSKYPDATTGTAVRAFMQAAIGPGQEGLDQYGSIPLPKSFQAKLAAAVNAIS

>228|CORE_REP|Org67_Gene3069#

MWIRAERVAVLTPTASLRRLTACYAALAVCAALACTTGQPAARAADGREMLAQAIATTRGSYLVYNFGGGHPMPLLNAGGHWYEMNNGGHLMIIKNASQRLSPHLLVDTHTGDQARCEHNPGARTGEGLWQASEIYPPLKAWQRMGRPTIAVNANFFDVRGQKGGSWRSTGCSSPLGAYVDNTRGQGRANQAVTGTVAYAGKQGLSGGNELWSSLTTMILPVGGAPYVLRPKSRQDYDLATPVIEDLLNKNARFVAVAGIGLLSPGNTGQLHDGGPSAARTALAYAKQKDEMYIFQGGNYTPDNIQDLFRGLGSDTAILLDGGGSSAIVLRRDTGGMWAGAGSPKGSCDTRQVLCDSHERALPSWLAFN

>229|CORE_REP|Org40_Gene2908#

MTLIAARRYSATMHGSASEACGSVDHLVDRHPTVSPVRLIAQLRPPPTFAEVSFATYRPDPVEPTQAAAVVACQDFCRQAVERRAGRKKWFGKRDVLPGVGLYLDGGFGVGKTHLLASAYYQLPGTGPDAPTCPKAFATFGELTQLAGVFGFADCIDLLANYTALCIDEFELDDPGNTTLISRLLSALVERGVSVAATSNTLPEQLGEGRFAAQDFLREINTLASIFTTVRIEGPDYRHRDLPPAPAPLSDEEVAARAARVEGATLDDFDALCAHLATMHPSRYLTLIEGVTAVFLTGVHGIDDQNVALRLVALVDRLYDAGIPVVASGAKLDTIFSEEMLAGGYRKKYLRATSRLLALTAGVIQAREP

>230|CORE_REP|Org119_Gene3399#

MSGGACIAVRSLSRSWTDNAIRLIEADARRSADTHLLRYPLPAAWCTDVDVELYLKDETTHITGSLKHRLARSLFLYALCNGWINENTTVVEASSGSTAVSEAYFAALLGLPFIAVMPAATSASKIALIESQGGRCHFVQNSSQVYAEAERVAKETGGHYLDQFTNAERATDWRGNNNIAESIYVQMREEKHPTPEWIVVGAGTGGTSATIGRYIRYRRHATRLCVVDPENSAFFPAYAEGRYDIVMPTSSRIEGIGRPRVEPSFLPGVVDRMVAVPDAASIAAARHVSAVLGRRVGPSTGTNLWGAFGLLAEMVKQGRSGSVVTLLADSGDRYADTYFSDEWVSAQGLDPAGPAAALVEFERSCRWT

>231|CORE_REP|Org5_Gene3476#

MSEHQSLPAPEASTEVRVAIVGVGNCASSLVQGVEYYYNADDTSTVPGLMHVRFGPYHVRDVKFVAAFDVDAKKVGFDLSDAIFASENNTIKIADVAPTNVIVQRGPTLDGIGKYYADTIELSDAEPVDVVQALKEAKVDVLVSYLPVGSEEADKFYAQCAIDAGVAFVNALPVFIASDPVWAKKFTDARVPIVGDDIKSQVGATITHRVLAKLFEDRGVQLDRTMQLNVGGNMDFLNMLERERLESKKISKTQAVTSNLKREFKTKDVHIGPSDHVGWLDDRKWAYVRLEGRAFGDVPLNLEYKLEVWDSPNSAGVIIDAVRAAKIAKDRGIGGPVIPASAYLMKSPPEQLPDDIARAQLEEFIIG

>233|CORE_REP|Org79_Gene870#

MFPGFDALPEVLRPVARPQPPNAHPVAQPPAQALVDCGVYVCGQRLPGKYTYAAALREVREIELTGQEAFVWIGLHEPDENQMQDVADVFGLHPLAVEDAVHAHQRPKLERYDETLFLVLKTVNYVPHESVVLAREIVETGEIMIFVGKDFVVTVRHGEHGGLSEVRKRMDADPEHLRLGPYAVMHAIADYVVDHYLEVTNLMETDIDSIEEVAFAPGRKLDIEPIYLLKREVVELRRCVNPLSTAFQRMQTESKDLISKEVRRYLRDVADHQTEAADQIASYDDMLNSLVQAALARVGMQQNMDMRKISAWAGIIAVPTMIAGIYGMNFHFMPELDSRWGYPTVIGGMVLICLFLYHVFRNRNWL

>234|CORE_REP|Org59_Gene2359#

MTDIGAPVTVQVAVDPPYPVVIGTGLLDELEDLLADRHKVAVVHQPGLAETAEEIRKRLAGKGVDAHRIEIPDAEAGKDLPVVGFIWEVLGRIGIGRKDALVSLGGGAATDVAGFAAATWLRGVSIVHLPTTLLGXVXWRSCRRRQDRHQXRXAGXNLVGXXHQDPLAVLVDLATLQTLPRDEMICGMAEVVKAGFIADPVILDLIEADPQAALDPAGDVLPELIRRAITVKAEVVAADEKESELREILNYGHTLGHAIERRERYRWRHGAAVSVGLVFAAELARLAGRLDDATAQRHRTILSSLGLPVSYDPDALPQLLEIMAGDKKTRAGVLRFVVLDGLAKPGRMVGPDPGLLVTAYAGVCAP

>235|CORE_REP|Org20_Gene868#

MAAFVVDQLEELYRRMWVLRLLDMALEQLRIEGLINGPLQGGFGQEAVSVGAAAALGEGDVIITTHRPHAQHVGTDAPLGPVIADMLGATAGDLEGADEDAHIADPRAGLPAAIRVVKQSPLLAIGHAYALWLRDTGRVTLCVTQDCDVDADAFNEAADLAAVWQLPVVILVENIRGALSVHLDRYTHEPRVYRRAVAYGMPGVSVDGNDVEAVRDCVANAVVRARAGGGPTLVQAITYRTTDFSGSDRGGYRDSGRIRAVSGSADLREKAADCCWHDPRSARRAGAGGIAQGPMPWLRQGQGAAQRRWANQPTNIRLAPTTKDPVLRPRCTLAADNAVGTCRRAAAPASRAASAASPRRPRQLGR

>236|CORE_REP|Org2_Gene1866#

MGSARAEMTKDAGEYLVTQAATRPTNDAGQDGGNNSDILVVARQQVLQRGEGLNQDQVLAVLQLPDDRLEELLALAHEVRMRWCGPEVEVEGIISLKTGGCPEDCHFCSQSGLFASPVRSAWLDIPSLVEAAKQTAKSGATEFCIVAAVRGPDERLMAQVAAGIEAIRNEVEINIACSLGMLTAEQVDQLAARGVHRYNHNLETARSFFANVVTTHTWEERWQTLSMVRDAGMEVCCGGILGMGETLQQRAEFAAELAELGPDEVPLNFLNPRPGTPFADLEVMPVGDALKAVAAFRLALPRTMLRFAGGREITLGDLGAKRGILGGINAVIVGNYLTTLGRPAEADLELLDELQMPLKALNASL

>237|CORE_REP|Org118_Gene2028#

MCAHEADPYRRCRSGDGVHLADPGADPVVPGTQGFGHQIREDGPPSHHTKRGTPSMGGVAILAGIWAGYLGAHLAGLAFDGEGIGASGLLVLGLATALGGVGFIDDLIKIRRSRNLGLNKTAKTVGQITSAVLFGVLVLQFRNAAGLTPGSADLSYVREIATVTLAPVLFVLFCVVIVSAWSNAVNFTDGLDGLAAGTMAMVTAAYVLITFWQYRNACVTAPGLGCYNVRDPLDLALIAAATAGACIGFLWWNAAPAKIFMGDTGSLALGGVIAGLSVTSRTEILAVVLGALFVAEITSVVLQILTFRTTGRRMFRMAPFHHHFELVGWAETTVIIRFWLLTAITCGLGVALFYGEWLAAVGA

>238|CORE_REP|Org3_Gene1855#

MANVQYSAVTQRYPGADAPTVDNLDLDIADGEFLVLVGPSGCGKSTTLRVLAGLEPIESGRISIGDVDVTHLPPRARDVAMVFQNYALYPNMTVAANMGFALRNAGMSRADTRRRVLEVADMLELTDLLDRKPAKLSGGQRQRVAMGRAIVRRPRVFCMDEPLSNLDAKLRVSTRSQISGLQRRLGTTTVYVTHDQVEAMTMGDRVAVLKDGVLQQVDTPRALYDDPVNTFVATFIGAPAMNLIDAAVAHGVVRAPDLAIPVPDPAAERVLVGVRPESWDVASIGTPGSLTVHVELVEELGFESFVYATPVDQRGWSSRAPRIVFRTDRRTAVRVGESLAIVPHSQEVRLFNSRTETRLR

>239|CORE_REP|Org1_Gene945#

MKIQQAKPPVTQDTSATCPLTSTVQDSSPVAGQLGRPIGFRGLAGGCPVSPLGYESPPLPLGPDSLTWRYFGDWRGMLQGPWAGSMQNMHPQLGAAVEDHSTFFRERWPRLLRSLYPIGGVVFDGDRAPVTGVQVRDYHITIKGVDGAGRRYHALNPDVFYWAHATFFVGTLHVAERFCGGLTEAQRRQLFDEHVQWYRMYGMSMRPVPATWEEFQDYWDHMCRNVLENNFAARAVLDLTELPKPPFAQRVPDWLWAAPRKLLARFFVWLTVGLYDPPVRELMGYRWLRRDEWLHRRFGDIVRLVFALVPFRFRKHPRARAGWDRATGRIPADAPLVQTPARNLPPPDERDNPTHYCPKV

>240|CORE_REP|Org1_Gene3523#

MIKPDDHTVNVVDIQVLKNAVLLACRAPSVHNSQPWRWVAESGSEHTTVHLFVNRHRTVPATDHSGRQAIISCGAVLDHLRIAMTAAHWQANITRFPQPNQPDQLATVEFSPIDHVTAGQRNRAQAILQRRTDRLPFDSPMYWHLFEPALRDAVDKDVAMLDVVSDDQRTRLVVASQLSEVLRRDDPYYHAELEWWTSPFVLAHGVPPDTLASDAERLRVDLGRDFPVRSYQNRRAELADDRSKVLVLSTPSDTRADALRCGEVLSTILLECTMAGMATCTLTHLIESSDSRDIVRGLTRQRGEPQALIRVGIAPPLAAVPAPTPRRPLDSVLQIRQTPEKGRNASDRNARETGWFSPP

>241|CORE_REP|Org24_Gene2464#

MGNVAGETRANVIPLHTNRSRVAARRRAGQRAESRQHPSLLSDPNDRASAEQIAAVVREIDEHRRAAGATTSSTEATPNDLAQLVAAVAGFLRQRLTGDYSVDEFGFDPHFNSAIVRPLLRFFFKSWFRVEVSGVENIPRDGAALVVANHAGVLPFDGLMLSVAVHDEHPAHRDLRLLAADMVFDLPVIGEAARKAGHTMACTTDAHRLLASGELTAVFPEGYKGLGKRFEDRYRLQRFGRGGFVSAALRTKAPIVPCSIIGSEEIYPMLTDVKLLARLFGLPYFPITPLFPLAGPVGLVPLPSKWRIAFGEPICTADYASTDADDPMVTFELTDQVRETIQQTLYRLLAGRRNIFFG

>243|CORE_REP|Org29_Gene1580#

MLSLTLSEASCIASASRWRHIIPAGVVCALIAGIGVGCHGGPSDVVGRAGPDRAHTSITLVAYAVPEPGWSAVIPAFNASEQGRGVQVITSYGASADQSRGVADGKPADLVNFSVEPDIARLVKAGKVDKDWDADATKGIPFGSVVTFVVRAGNPKNIRDWDDLLRPGIEVITPSPLSSGSAKWNLLAPYAAKSDGGRNNQAGIDFVNTLVNEHVKLRPGSGREATDVFVQGSGDVLISYENEAIATERAGKPVQHVTPPQTFKIENPLAVVATSTHLGAATAFRNFQYTVQAQKLWAQAGFRPVDPAVAADFADLFPVPAKLWTIADLGGWGSVDPQLFDKATGSITKIYLRATG

>244|CORE_REP|Org119_Gene2378#

MRTVLWPITQTSVVAGLAWYLTHDVFNHPQAFFAPISAVVCMSATNVLRARRAQQMIVGVALGIVLGAGVHALLGSGPIAMGVVVFIALSVAVLCARGLVAQGLMFINQAAVSAVLVLVFASNGSVVFERLFDALVGGGLAIVFSILLFPPDPVVMLCSARADVLAAVRDILAELVNTVSDPTSAPPDWPMAAADRLHQQLNGLIEVRANAAMVARRAPRRWGVRSTVRDLDQQAVYLALLVSSVLHLARTIAGPGGDKLPTPVHAVLTDLAAGTGLADADPTAANEHAAAARATASTLQSAACGSNEVVRADIVQACCHRSTTGNRTPGPVGHVSLNWRETPAPAASAPASARPR

>245|CORE_REP|Org118_Gene2226#

MTSRETRAADAAGARQADAQVRSSIDVPPDLVVGLLGSADENLRALERTLSADLHVRGNAVTLCGEPADVALAERVISELIAIVASGQSLTPEVVRHSVAMLVGTGNESPAEVLTLDILSRRGKTIRPKTLNQKRYVDAIDANTIVFGIGPAGTGKTYLAMAKAVHALQTKQVTRIILTRPAVEAGERLGFLPGTLSEKIDPYLRPLYDALYDMMDPELIPKLMSAGVIEVAPLAYMRGRTLNDAFIVLDEAQNTTAEQMKMFLTRLGFGSKVVVTGDVTQIDLPGGARSGLRAAVDILEDIDDIHIAELTSVDVVRHRLVSEIVGRLCAVRGARVGAESGGSAGVRRPRSPMMGA

>246|CORE_REP|Org59_Gene801#

MSVLDVGCGPGTITVDLAARVVXGSXTGVEPTDDALSLARAEAQLHRLSNISFTTSDVHKLDFPDDAFDVVHAHQVLQHVADPVRALQEMRRVCTPGGIVAARDADYSGFIWFPKLPALDRWLDLYERAARANGGEPDAXLGGCCPGPVRQDSTTSRRRPVSGVSRRPRPANGGXXXGXTGFCNXIWLTSWWIRVWPLPRNSRRSPRRGESGPRPRTVGWRYPTVKSFAGHKLRHTREARAVGCRRRAGRGPARSNIVQPKEFTHHPVESRRLFDERHVGTGLEEFQSXSXXXGPAFLACAQANIRRNGPPPARSAPRCRPTGRRRPSLEHSQRCESRWAQTSSRRSRFPCWRMLP

>247|CORE_REP|Org2_Gene2691#

MTNGLRNYRLGVGYTVERRGRRRAEPLGTVADMKYLDVDGIGQVSRIGLGTWQFGSREWGYGDRYATGAARDIVKRARALGVTLFDTAEIYGLGKSERILGEALGDDRTEVVVASKVFPVAPFPAVIKNRERASARRLQLNRIPLYQIHQPNPVVPDSVIMPGMRDLLDSGDIGAAGVSNYSLARWRKADAALGRPVVSNQVHFSLAHPDALEDLVPFAELENRIVIAYSPLAQGLLGGKYGLENRPGGVRALNPLFGTENLRRIEPLLATLRAIAVDVDAKPAQVALAWLISLPGVVAIPGASSVEQLEFNVAAADIELSAQSRDALTDAARAFRPVSTGRFLTDMVREKVSRR

>248|CORE_REP|Org96_Gene1131#

MSVIAGVFGALPPHRYSQSEITDSFVEFPGLKEHEEIIRRLHAAAKVNGRHLVLPLQQYPSLTDFGDANEIFIEKAVDLGVEALLGALDDANLRPSDIDMIATATVTGVAVPSLDARIAGRLGLRPDVRRMPLFGLGCVAGAAGVARLRDYLRGAPDDVAVLVSVELCSLTYPAVKPTVSSLVGTALFGDGAAAVVAVGDRRAEQVRAGGPDILDSRSSLYPDSLHIMGWDVGSHGLRLRLSPDLTNLIERYLANDVTTFLDAHRLTKDDIGAWVSHPGGPKVIDAVATSLALPPEALELTWRSLGEIGNLSSASILHILRDTIEKRPPSGSAGLMLAMGPGFCTELVLLRWR

>249|CORE_REP|Org2_Gene3633#

MLFVSVAPESVGVAAATLVGPPLIGNGADRPRHRTSRRDLVGQRPFSPNHRSGVLNATTAGAVQFNVLGPLELNLRGTKLPLGTPKQRAVLAMLLLSRNQVVAADALVQAIWEKSPPARARRTVHTYICNLRRTLSDAGVDSRNILVSEPPGYRLLIGDRQQCDLDRFVAAKESGLRASAKGYFSEAIRYLDSALQNWRGPVLGDLRSFMFVQMFSRALTEDELLVHTKLAEAAIACGRADVVIPKLERLVAMHPYRESLWKQLMLGYYVNEYQSAAIDAYHRLKSTLAEELGVEPAPTIRALYHKILRQLPMDDLVGRVTRGRVDLRGGNGAKVEELTESDKDLLPIGLA

>250|CORE_REP|Org59_Gene3530#

MAAARAAETESDNPLINDPFARIFVDAAGDGIWSMYTNRTLLAGATDLDPDLRAPIQQMIDFMAARTAFFDEYFLATADAGVRQVVILASGLDSRAWXLPWPDGTVVYELDQPKVLEFKSATLRQHGAQPASQLVNVPIDLRQDWPKALQKAGFDPSKPCAWLAEGLVRXLPARAQDLLFERIDALSRPGSWLASNVPGAGFLDPERMRRQRADMRRMRXAAAKXVETEISDVDDLWYAEQRTAVEPSGCVNVAGXCRRQRCPSCWLGMAAASLTXXKTQSRQTFSYPRSGPRADGDRGLFREHGRHVVLDHRYRVNRSEVLALFVLSELRPPGAVGPAQLVPYLAGFGQ

>251|CORE_REP|Org59_Gene2324#

MNLRAAGPGWLFCPADRPERFAKAAAAADVVILDLEDGVAEAQKPAARNALRDTPLDPERTVVRINAGGTADQARDLEALAGTAYTTVMLPKAESAAXVIELAPRDVIALVETARGAVCAAEIAAADPTVGMMWGAEDLIATLGGSSSRRADGAYRDVARHVXSTILLAASAFGRLALDAVHLDILDVEGLQEEARDAAAVGFDVTVCIHPSQIPVVRKXYRPSHEKLAWARRRXGRVAKRAWGVRVRRPDGRLSSAHARGNDVAASRGSHLRMRAQIPRDTVAMRSRSVRTLHIWGHNGGSASVAASGSFGAAGDQLRLAAVSQAGCAVSAAPPAPTLRMERTASSDA

>253|CORE_REP|Org33_Gene2262#

MLISQRPTLSEDVLTDNRSQFVIEPLEPGFGYTLGNSLRRTLLSSIPGAAVTSIRIDGVLHEFTTVPGVKEDVTEIILNLKSLVVSSEEDEPVTMYLRKQGPGEVTAGDIVPPAGVTVHNPGMHIATLNDKGKLEVELVVERGRGYVPAVQNRASGAEIGRIPVDSIYSPVLKVTYKVDATRVEQRTDFDKLILDVETKNSISPRDALASAGKTLVELFGLARELNVEAEGIEIGPSPAEADHIASFALPIDDLDLTVRSYNCLKREGVHTVGELVARTESDLLDIRNFGQKSIDEVKIKLHQLGLSLKDSPPSFDPSEVAGYDVATGTWSTEGAYDEQDYAETEQL

>254|CORE_REP|Org61_Gene43#

MSAPWGPVAAGPSALVRSGQASTIEPFQREMTPPTPTPEAAHNPTMNVSRETSTEFDTPIGAAAERAMRVLHTTHEPLQRPGRRRVLTIANQKGGVGKTTTAVNIAAALAVQGLKTLVIDLDPQGNASTALGITDRQSGTPSSYEMLIGEVSLHTALRRSPHSERLFCIPATIDLAGAEIELVSMVARENRLRTALAALDNFDFDYVFVDCPPSLGLLTINALVAAPEVMIPIQCEYYALEGVSQLMRNIEMVKAHLNPQLEVTTVILTMYDGRTKLADQVADEVRQYFGSKVLRTVIPRSVKVSEAPGYSMTIIDYDPGSRGAMSYLDASRELAERDRPPSAKGRP

>255|CORE_REP|Org88_Gene1048#

MTITALTVTLPLLWRRLTTAGVKYADQGHFVGSAGVPAADAGGRDAASEQIARWTQTCTVVLVCGHGPAKWAFRSWCTSRSCDTLPVALRYRLQSNPLVGKLTTKYFLPLGTRQVGDHVVFFNFGYEEDPPMALPLSESDEPNRYCIQLYHQTASQVDLTGKEVLEVSCGAGGGASYIARNLGPASYTGLDLNPASIDLCRAKHRLPGLQFVQGDAQNLPFPDESFDAVVNVEASHQYPDFRGFLAEVARVLRPGGHFLYTDSRRNPVVAEWEAALADAPLRTISQRDIGAQAKRGLDANTARSQEAIGRRAPVLLAGLTRCAVRVLDWDLRRGGGFSYRIYLFAKD

>256|CORE_REP|Org119_Gene3555#

MTYTLRYRWWVVLTGVAIAAFTVHGVAVAIGHFLGSTVPARPAACVSAIAFLIFAVWVWREDTASDSETSPTAAEPRLALFTVVSSFALAELGDKTTLATVTLASDHHWAGVWIGTTLGMILADGLAIGAGLLLHRRLPERLLQVLTGLLFLLFGLWLLFDDALGFRSVAIAVTAAVVLAAATTAVSVRVAQTRRRRPTAAATPEDDSTRPERSSVAPGHPGSILLPLPEVSLRGRRPPSGSPDERCAAPQAAKEALGESPLAAGCPESAASARHGHPDLLAEHVGDGPTRVFSSDILRITCEIRSSCGHITESVGPSSGGSSLTPHNVSTQSAQVCTWLWTQLSLP

>257|CORE_REP|Org59_Gene1012#

MTGESHEVLTNVEGGVGFVTLNRPKAINSLNQTMVDLLATVLMSWEHEDAVHAVVLSGAGERXLCAGGDVDGRLPQCXQGRGRGAAVLAPRVSAQRPDRPXRQALRGVDGRHRNGRRRRRQRTREHPGGYAXXLQGRDARSGHRGVIPDVGGVYLLSRAPGALGLHAALTGAPFSGADAIALGFADHFVPHGDLDAFTQKIVTGGVESALAAHAVEPPPSTLAAQRDWIDECYAGDSVADIVAALRKQGGEPAVNASDLIASRSPIALSVTLQAVRRAAKLDTLEDVLIQDYRVSSASLRSHDLVEGIRAQLIDKDRNPNWSPATLDAITAADIEAYFEPVDDDLSF

>258|CORE_REP|Org108_Gene1099#

MVLQELWFGVIAALFLGFFILEGFDFGVGMLMAPFAHVGMGDPETHRRTALNTIGPVWDGNEVWLITAGAAIFAAFPGWYATVFSALYLPLLAILFGMILRAVAIEWRGKIDDPKWRTGADFGIAAGSWLPALLWGVAFAILVRGLPVDANGHVALSIPDVLNAYTLLGGLATAGLFSLYGAVFIALKTSGPIRDDAYRFAVWLSLPVAGLVAGFGLWTQLAYGKDWTWLVLAVAGCAQAAATVLVWRRVSDGWAFMCTLIVVAAVVVLLFGALYPNLVPSTLNPQWSLTIHNASSTPYTLKIMTWVTAFFAPLTVAYQTWTYWVFRQRISAERIPPPTGLARRAP

>259|CORE_REP|Org139_Gene3112#

MKITGTVVKLGIVSVVLLFFTVMIIVIFGQMRFDRTNGYTAEFSNVSGLRQGQFVRASGVEIGKVKALHLVDGGRRVRVEFNIDRSVPLYQSTTAQIRYSDLIGNRYVELKRGEGKGANDLLPPGGLIPLSRTSPALDLDALIGGFKPVFRALDPAKVNNIANALITVFQGQGGTINDILDQTAQLTSQIAERDQAIGEVVKNLNIVLDTTVKHRKEFDETVNNLENLITGLRNHSDQLAGGLAHISNGAGTVADLLAENRTLVRKAVSYLDAIQQPVIDQRVELDDLLHKTPTALTALGRANGTYGDFQNFYLCDLQIKWNGFQAGGPVRTVKLFSQPTGRCTPQ

>260|CORE_REP|Org119_Gene1356#

MSPSLRVQRVIAAIVILTQGGIAVTGAIVRVTASGLGCPTWPQCFPGSFTPVVVAEVPRVHQAVEFGNRMVTFAVVIAAALAVLVVTRARRRTEVLAYAWLMPVSTVVQAMIGGITVRTGLLWWTVAIHLLASMTMVWLAVLLYVKIGQPDDGVVHELVVSPLRALTALSALNLAAVLVTGTLVTAAGPHAGDRSPSRTVPRLKVEITTLVHMHSSLLVAYLALLIGLGFGLLAVGATRAILVRLAVLLALVATQAARRYHAILHRGTRRPGRHSRGRCRGGYGGHRRAMGVDGRTGPAPAAPTLTGRIARSHAGQDCGCLGPATASPATGPPIAIPATGDLGFHL

>261|CORE_REP|Org20_Gene3700#

MAAVLPTLIRTGAVALGSAIAGIGYAALVERNAFVLREVTMPVLTPGSTPLRVLHISDLHMLPNQHRKQAWLRELASWEPDLVVNTGDNLAHPKAVPAVVQTLSDLLSRPGVFVFGSNDYFGPRLKNPMNYLTSPDHRVRGAALPWQDLRAAFTERGWLDLTHTRREFEVAGLHIAAAGVDDPHIDRDRYDTIAGPASPAANLRLGLTHSPEPRVLDRFAADGYQLVLAGHTHGGQLCLPLYGALVTNCGLDRSRAKGASHWGANMRLHVSAGIGTSPFAPVRFCCRPEATLLTLIATPMGGRDSSSNLGRSQPTVFWRVGLYRGPQPIPELDGQRDPVDRGGRPP

>263|CORE_REP|Org83_Gene269#

MPIATPEVYAEMLGQAKQNSYAFPAINCTSSETVNAAIKGFADAGSDGIIQFSTGGAEFGSGLGVKDMVTGAVALAEFTHVIAAKYPVNVALHTDHCPKDKLDSYVRPLLAISAQRVSKGGNPLFQSHMWDGSAVPIDENLAIAQELLKAAAAAKIILEIEIGVVGGEEDGVANEINEKLYTSPEDFEKTIEALGAGEHGKYLLAATFGNVHGVYKPGNVKLRPDILAQGQQVAAAKLGLPADAKPFDFVFHGGSGSLKSEIEEALRYGVVKMNVDTDTQYAFTRPIAGHMFTNYDGVLKVDGEVGVKKVYDPRSYLKKAEASMSQRVVQACNDLHCAGKSLTH

>264|CORE_REP|Org107_Gene3207#

MTTVLGIETSCDETGVGIARLDPDGTVTLLADEVASSVDEHVRFGGVVPEIASRAHLEALGPAMRRALAAAGLKQPDIVAATIGPGLAGALLVGVAAAKAYSAAWGVPFYAVNHLGGHLAADVYEHGPLPECVALLVSGGHTHLLHVRSLGEPIIELGSTVDDAAGEAYDKVARLLGLGYPGGKALDDLARTGDRDAIVFPRGMSGPADDRYAFSFSGLKTAVARYVESHAADPGFRTADIAAGFQEAVADVLTMKAVRAATALGVSTLLIAGGVAANSRLRELATQRCGEAGRTLRIPSPRLCTDNGAMIAAFAAQLVAAGAPPSPLDVPSDPGLPVMQGQVR

>265|CORE_REP|Org124_Gene42#

MTQPSRRKGGLGRGLAALIPTGPADGESGPPTLGPRMGSATADVVIGGPVPDTSVMGAIYREIPPSAIEANPRQPRQVFDEEALAELVHSIREFGLLQPIVVRSLAGSQTGVRYQIVMGERRWRAAQEAGLATIPAIVRETGDDNLLRDALLENIHRVQLNPLEEAAAYQQLLDEFGVTHDELAARIGRSRPLITNMIRLLKLPIPVQRRVAAGVLSAGHARALLSLEAGPEAQEELASRIVAEGLSVRATEETVTLANHEANRQAHHSDATTPAPPRRKPIQMPGLQDVAERLSTTFDTRVTVSLGKRKGKIVVEFGSVDDLARIVGLMTTDGRDKGLHRDAL

>266|CORE_REP|Org1_Gene568#

MRLGVLDVGSNTVHLLVVDAHRGGHPTPMSSTKATLRLAEATDSSGKITKRGADKLISTIDEFAKIAISSGCAELMAFATSAVRDAENSEDVLSRVRKETGVELQALRGEDESRLTFLAVRRWYGWSAGRILNLDIGGGSLEVSSGVDEEPEIALSLPLGAGRLTREWLPDDPPGRRRVAMLRDWLDAELAEPSVTVLEAGSPDLAVATSKTFRSLARLTGAAPSMAGPRVKRTLTANGLRQLIAFISRMTAVDRAELEGVSADRAPQIVAGALVAEASMRALSIEAVEICPWALREGLILRKLDSEADGTALIESSSVHTSVRAVGGQPADRNAANRSRGSKP

>267|CORE_REP|Org7_Gene2812#

MTSPWPAGSTSRVPVLRDEWREPLRALRDPLAATDRRVRARRDRKRQWRKQTWLGRFVSTYGWRAYALPVLMVLTTVVVYQTVTGTSTPRPAAAQTVRDSPAIGVVGTAILDAPPRGLAVFDANLPAGTLPDGGPFTEAGDKTWRVVPGTTPQVGQGTVKVFRYTVEIENGLDPTMYGGDNAFAQMVDQTLTNPKGWTHNPQFAFVRIDSGKPDFRISLVSPTTVRGGCGYEFRLETSCYNPSFGGMDRQSRVFINEARWVRGAVPFEGDVGSYRQYVINHEVGHAIGYLRHEPCDQQGGLAPVMMQQTFSTSNDDAAKFDPDFVKADGKTCRFNPWPYPIP

>268|CORE_REP|Org103_Gene3292#

MTERKRNLRPVRDVAPPTLQFRTVHGYRRAFRIAGSGPAILLIHGIGDNSTTWNGVHAKLAQRFTVIAPDLLGHGQSDKPRADYSVAAYANGMRDLLSVLDIERVTIVGHSLGGGVAMQFAYQFPQLVDRLILVSAGGVTKDVNIVFRLASLPMGSEAMALLRLPLVLPAVQIAGRIVGKAIGTTSLGHDLPNVLRILDDLPEPTASAAFGRTLRAVVDWRGQMVTMLDRCYLTEAIPVQIIWGTKDVVLPVRHAHMAHAAMPGSQLEIFEGSGHFPFHDDPARFIDIVERFMDTTEPAEYDQAALRALLRRGGGEATVTGSADTRVAVLNAIGSNERSAT

>269|CORE_REP|Org149_Gene1664#

MRDFHCPNCGQRLAFENSACLSCGSALGFSLGRMALLVIADDADVQLCANLHLAQCNWLVPSDQLGGLCSSCVLTIERPSDTNTAGLAEFARAEGAKRRLIAELHELKLPIVGRDQDPDHGLAFRLLSSAHENVTTGHQNGVITLDLAEGDDVHREQLRVEMDEPYRTLLGHFRHEIGHYYFYRLIASSSDYLSRFNELFGDPDADYSQALDRHYRGGPPEGWQDSFVSSYATMHASEDWAETFAHYLHIRDALDTAAWCGLAPASATFDRPALGPSAFNTIIDKWLPLSWSLNMVNRSMGHDDLYPFVLPAAVLEKMRFIHTVVDEVAPDFEPAHSRRTV

>270|CORE_REP|Org59_Gene3446#

MVATTSSGGSSVGWPSRLSGVRLHLVTGKGGTGKSTIAAALALTLAAGGRKVLLVEVEGRQGIAQLFDVPPLPYQELKIATAERGGQVNALAIDIEAAFLEYGLDMFYNLGIAGRAMRRIGAVEFATTIAPGLRDVLLTGKIKETVVRLDKNKLPVYDAIVVDAPPTGRIARFLDVTKAVSDLAKGGPVHAQSEGVVKLLHSNQTAIHLVTLLEALPVQETLEAIEELAQMELPIGSVIVNRNIPAHLEPQDLAKAAEGEVDADSVRAGLLTAGVKLPDADFAGLLTETIQHATRITARAEIAQQLDALQVPRLELPTVSDGVDLGSLYELSESLAQQGVR

>271|CORE_REP|Org14_Gene116#

MTFFEQVRRLRSAATTLPRRLAIAAMGAVLVYGLVGTFGGPATAGAFSRPGLPVEYLQVPSASMGRDIKVQFQGGGPHAVYLLDGLRAQDDYNGWDINTPAFEEYYQSGLSVIMPVGGQSSFYTDWYQPSQSNGQNYTYKWETFLTREMPAWLQANKGVSPTGNAAVGLSMSGGSALILAAYYPQQFPYAASLSGFLNPSEGWWPTLIGLAMNDSGGYNANSMWGPSSDPAWKRNDPMVQIPRLVANNTRIWVYCGNGTPSDLGGDNIPAKFLEGLTLRTNQTFRDTYAADGGRNGVFNFPPNGTHSWPYWNEQLVAMKADIQHVLNGATPPAAPAAPAA

>272|CORE_REP|Org36_Gene2044#

MELGSLIRATNLWGYTDLMRELGADPLPFLRRFDIPPGIEHQEDAFMSLAGFVRMLEASAAELDCPDFGLRLARWQGLGILGPVAVIARNAATLFGGLEAIGRYLYVHSPALTLTVSSTTARSNVRFGYEVTEPGIPYPLQGYELSMANAARMIRLLGGPQARARVFSFRHAQLGTDAAYREALGCTVRFGRTWCGFEVDHRLAGRPIDHADPETKRIATKYLESQYLPSDATLSERVVGLARRLLPTGQCSAEAIADQLDMHPRTLQRRLAAEGLRCHDLIERERRAQAARYLAQPGLYLSQIAVLLGYSEQSALNRSCRRWFGMTPRQYRAYGGVSGR

>273|CORE_REP|Org59_Gene787#

MSAKLTDLQLLHELEPVVEKYLNRHLSMHKPWNPHDYIPWSDGKNYYALGGQDWDPDQSKLSDVAQVAMVQNLVTEDNLPSYHREIAMNMGMDGAWGQWVNRWTAEENRHGIALRDYLVVTRSVDPVELEKLRLEVVNRGFSPGQNHQGHYFAESLTDSVLYVSFQELATRISHRNTGKACNDPVADQLMAKISADENLHMIFYRDVSEAAFDLVPNQAMKSLHLILSHFQMPGFQVPEFRRKAVVIAVGGVYDPRIHLDEVVMPVLKKWRIFEREDFTGEGAKLRDELALVIKDLELACDKFEVSKQRQLDREARTGKKVSAHELHKTAGKLAMSRR

>274|CORE_REP|Org2_Gene3234#

MRDNSTEHKTRRAASSKDVRPAELDEVDRRILSLLHGDARMPNNALADTVGIAPSTCHGRVRRLVDLGVIRGFYTDIDPVAVGLPLQAMISVNLQSSARGKIRSFIQQIRRKRQVMDVYFLAGADDFILHVAARDTEDLRSFVVENLNADADVAGTQTSLIFEHLRGRRPSRAARPAGYDRGMPDPDGPSVTVTVEIDANPDLVYGLITDLPTLASLAEEVVAMQLRKGDDVRKGAVFVGRNENGGRRWTTTCTVTDADPGRVFAFDVRSGIIPISRWQYGIVATEHGCRVTESTWDRRPSWFRAVARMATGVKDRASVNTEHIRRTLQRLKDRAEAG

>275|CORE_REP|Org59_Gene742#

MYFVGVDXAWAGRNPXXXAAVDADGCLVGVGAARDDASVLAALRPYVVGDCLVAFDAPLVVANRTGQRPAEAALNRDFRQFEAGAYPANTEKPEFADVPRAARLARQLALDMDPLSSATRRAIEVYPHPATVALFRLPRALKYKAKPGRSVDLLKSELLRLMDGVEGLAQAGVRMQVAGQPDWVSLRRQXTVAQRKSDLRXSPRIRSTPSYAPTWRCTPXXXPPMSRSMGTSPPGTLSRRRCPPTSERHRTLVDGREHVDEVDHRRQRSGTRVPPFDQRRDHRSVDRTDQRTHQFDLCGAAGALDGLGHGLSALTQAAAHAFAQVRQAGTGDMALVS

>276|CORE_REP|Org142_Gene2431#

MAKNSRRKRHRILAWIAAGAMASVVALVIVAVVIMLRGAESPPSAVPPGVLPPGPTPAHPHKPRPAFQDASCPDVQMISVPGTWESSPQQNPLNPVQFPKALLLKVTGPIAQQFAPARVQTYTVAYTAQFHNPLTTDNQMSYNDSRAEGTRAMVAAMTDMNNRCPLTSYVLIGFSQGAVIAGDVASDIGNGRGPVDEDLVLGVTLIADGRRQQGVGNQVPPSPRGEGAEITLHEVPVLSGLGLTMTGPRPGGFGALDGRTNEICAQGDLICAAPAQAFSPANLPTTLNTLAGGAGQPVHAMYATPEFWNSDGEPATEWTLNWAHQLIENAPHPKHR

>277|CORE_REP|Org119_Gene1717#

MTIRLGLQIPNFSYGTGVEKLFPSVIAQAREAEAAGYDSLFVMDHFYQLPMLGTPDQPMLEAYTALGALATATERLQLGALVTGNTYRSPTLLAKIITTLDVVSAGRAILGIGAGWFELEHRQLGFEFGTFSDRFNRLEEALQILEPMVKGERPTFFGDWYTTESAMAEPRYRDRIPILIGGGGEKKTFAIAARFADHLNIVAAVDELPRKMRALAARCDEAGRDRSTLQTSLLLTVMIDETLSPDAIPAEMSGRVVVGSPAQIADQIQAKVLDAGVDGLIINFGSARLSARGHHHRRSSTGGARCWVCSHRGPVTQARCGVADDTCPQLGLQRSG

>278|CORE_REP|Org38_Gene412#

MRTPATVVAGVDLGDAVFAAAVRAGVARVEQLMDTELRQADEVMSDSLLHLFNAGGKRFRPLFTVLSAQIGPQPDAAAVTVAGAVIEMIHLATLYHDDVMDEAQVRRGAPSANAQWGNNVAILAGDYLLATASRLVARLGPEAVRIIADTFAQLVTGQMRETRGTSENVDSIEQYLKVVQEKTGSLIGAAGRLGGMFSGATDEQVERLSRLGGVVGTAFQIADDIIDIDSESDESGKLPGTDVREGVHTLPMLYALRESGPDCARLRALLNGPVDDDAEVREALTLLRASPGMARAKDVLAQYAAQARHELALLPDVPGRRALAALVDYTVSRHG

>279|CORE_REP|Org112_Gene394#

MTEIATTSGARSVGLLSVGAYRPERVVTNDEICQHIDSSDEWIYTRTGIKTRRFAADDESAASMATEACRRALSNAGLSAADIDGVIVTTNTHFLQTPPAAPMVAASLGAKGILGFDLSAGCAGFGYALGAAADMIRGGGAATMLVVGTEKLSPTIDMYDRGNCFIFADGAAAVVVGETPFQGIGPTVAGSDGEQADAIRQDIDWITFAQNPSGPRPFVRLEGPAVFRWAAFKMGDVGRRAMDAAGVRPDQIDVFVPHQANSRINELLVKNLQLRPDAVVANDIEHTGNTSAASIPLAMAELLTTGAAKPGDLALLIGYGAGLSYAAQVVRMPKG

>280|CORE_REP|Org59_Gene1209#

MAITINMVNPTGFIRYEDVEQEAMTSDVTVGPAPGQYQLSHLRLLEAEAIHVIREVAAEFERPVLLFSGGKDSIVMLHLALKAFRPGRLPFPVMHVDTGHNFDEVIAXRDELVAAAGVRLVVASVQDDIDAGRVVETIPSRNPIQTVTLLRAIRENQFDAAFGGARRDEEKARAKERVFSFRDEFGQWDPKAQRPELWNLYNGRHHKGEHIRVFPLSNWTEFDIWSYIGAEQVRLPSIYFAHRRKVFQRDGMLLAVHRPHAXRGAXEPVFEATVRFRTVGDVTCTGCVESSASTVAEVIAETAVARLTERGATRADDRISEAGMEDRKRQGYF

>281|CORE_REP|Org119_Gene2767#

MAGAKHAGRIVAITTAAAVILAACSSGSKGGAGSGHAGKARSAVTTTDADWKPVADALGRSGKLGDNNTAYRINLPRNDLHITSYGVDIKPGLSLGGYAAFARYDNNETLLMGDLVITEEELPKVTDALQAHGIAQTALHKHLLQQDPPVWWTHIHGMGDAARLAQGLKAALDATTIGPPTPPPARQPPVDIDVAGVDQALGRKGTQDGGLLKYSIPRKDTIIEDGHVLPAVSLNLTTVINFQPVGRGRAAINGDFILIAPEVQEVIRAMRAGNITIVELHNHGLTEEPRLFYMHYWAVDDAVTLARALRPAMECHQPAVVIIPMQPHKGWCG

>282|CORE_REP|Org59_Gene1558#

MVIIELMRRVVGLAQGATAEVAVYGDRDRDLAERWCANTGNTLVRADVDQTGVGTLVVRRGHPPDPASVLGPDRLPGVRLWLYTNFHCNLCCDYCCVSSSPSTPHRELGAERIGRIVGEAARWGXRELFLTGGEPFLLPDIDTIIATCVKQLPTTVLTNGMVFKGRGRRALESLPRGLALQISLDSAXPRAARCXXRRGDVGQGSSWYPVGALTWLPGAGGRDGCQPRTWRADGVSRLPRRAWHRTRGSAGPADRAGGRRVARGGAHPRIAGSRGDRHRRRRVLAPSGRHRRARPGHPYRRTLDPGAGHGKPRLFAEQWTRAAEEXALFPCA

>283|CORE_REP|Org59_Gene2384#

MGGLTISDLVVEYSSGGXAVRPIDGXKPRRGAGVAGDLAWAQRLREDDPLVLPRRXXCARSPAQSSLTMSTSSNLXEGAALAKYRRDKXGIVFQAFNLVSSLTALENVMVPLRAAGVSRAAARKRAEDLLIRVNLGERMKHRPGDMSGGQQQRVAVARAIALDPQLILADEPTAHLDFIQVEEVLRLIRSLAQGDRVVVVATHDSRMLPLADRVLELMPAQVSPNQPPETVHVKAGEVLFEQSTMGDLIYVVSEGEFEIVRELADGGEELVKXAAPGDYFGEIGVLFXLPRSATVRARSDATAVGYTAQAFRERLGXXRXXDLIEHRELASE

>284|CORE_REP|Org18_Gene2243#

MRLLVTGGAGFIGTNFVHSAVREHPDDAVTVLDALTYAGRRESLADVEDAIRLVQGDITDAELVSQLVAESDAVVHFAAESHVDNALDNPEPFLHTNVIGTFTILEAVRRHGVRLHHISTDEVYGDLELDDRARFTESTPYNPSSPYSATKAGADMLVRAWVRSYGVRATISNCSNNYGPYQHVEKFIPRQITNVLTGRRPKLYGAGANVRDWIHVDDHNSAVRRILDRGRIGRTYLISSEGERDNLTVLRTLLRLMDRDPDDFDHVTDRVGHDLRYAIDPSTLYDELCWAPKHTDFEEGLRTTIDWYRDNESWWRPLKDATEARYQERGQ

>285|CORE_REP|Org146_Gene224#

MNPIPSWPGRGRVTLVLLAVVPVALAYPWQSTRDYVLLGVAAAVVIGLFGFWRGLYFTTIARRGLAILRRRRRIAEPATCTRTTVLVWVGPPASDTNVLPLTLIARYLDRYGIRADTIRITSRVTASGDCRTWVGLTVVADDNLAALQARSARIPLQETAQVAARRLADHLREIGWEAGTAAPDEIPALVAADSRETWRGMRHTDSDYVAAYRVSADAELPDTLPAIRSRPAQETWIALEIAYAAGSSTRYTVAAACALRTDWRPGGTAPVAGLLPQHGNHVPALTALDPRSTRRLDGHTDAPADLLTRLHWPTPTAGAHRAPLTNAVSRT

>286|CORE_REP|Org59_Gene3072#

MRAAQRTSSSSFGLGQVASGRLDDVPLPADPSPTLSAYAHPERLXTXDWXSAHMGAPGLAIVESDEDVLLYDVGHIPGAVKIDWHTDLNDPRVRDYINGEQFAELMDRKGIARDDTVVIYGDKSNWWAAYALWVFTLFGHADVRLLNGGRDLWLAERRETTLDVPTKTCTGYPVVQRNDAPIRAFRDDVLAILGAQPLIDVRSPEEYTGKRTHMPDYPEEGALRAGHIPTAVHIPWGKAADESGRFRSREELERLYDFINPDDQXXVYCRIGXXXSHTWFVLTHLLGKADVRNYDGSWTEWGNAVRVPIVAGEEPGVVPVVXPRPRACPRR

>287|CORE_REP|Org59_Gene3520#

MPTLDCDSDCRGATGVRAWAGTHRRFHHAGRRSGGXXGXXWXXXRSXXGXHGARSDGFDAITDLNQTLTRAAATEALIVLAVPMPALPGMLAHIRKSAPGCPLTDVTSVKCAVLDEVTAXGLQARYVGGHPMTGTAHSGWTAGHGGLFNRAPWVVSVDDHVDPTVWSMVMTLALDCGAMVVPAKSDEHDAAAAAVSHLPHLLAEALAVTAAEVPLAFALAAGSFRDATRVAATAPDLVRAMCEANTGQLAPAADRIIDLLSRARDSLQSHGSIADLADAGHAARTRYDSFPRSDIVTVVIGADKWREQLAAAGRAGGVITSALPSLDSPQ

>289|CORE_REP|Org57_Gene3234#

MAGWFAHTLRPAMLAAGRSDRLGRIVERSPLTRGVVRRFVPGDTLDDVVDIVTALRDSGRYLSIDYLGENVTDADDAAAAVRAYLGLLDVLGRRGDIACDGVRPLEVSLKLSALGQALDRDGQKIALDNARAICERAERVGAWVTVDAEDHTTTDSTLSISGDLRVDFPWLGTVVQAYLRRTLADCAELAAVGARVRLCKGAYDEPASVAYRDAAQVTDSYLRCLRVLTAGRGYPMVATHDPVIIAAVPGITRESGRSQGDFEYQMLYGVRDDEQRRLTGAGNHVRVYVPFGTRWYGYFLRRLAERPANLAFFLRALTDRRRARGCAER

>290|CORE_REP|Org30_Gene261#

MPGARELTLRVERGALFRRRWAASAASSARAAIRRDPRRCALGTRPRWVSFLVIVLVIMNVVTAHPKYPNDPLALVLIELRHPRTEPPVPSAISILKEELARWTPILEQEEVRQVNLETGEHTAHSQKKLVARDRRTAITFRPDAMTLEVTDYPGWEEFRSIVHAMVTARQDVAPVDGCIRIGLRYINEIRASLAEPSGWAYWVAESLLGPGTQLADLKLTTTAQRHVIQCEGPEPGDSLTLRYAGARGAVIQSTPFLQRLKEPPAEGDFFLIDIDSAWSDPCKGIPALDAHLVDEVAERLHTPIGPLFESLITSELRTKVLQQPGQE

>292|CORE_REP|Org59_Gene1366#

MLAAQFSLELKLLLRNGEQLLLTMFIPITLLVGLTLLPMGSFGHNRAATFVPVIMALAVISTAFTGQAIAVAFDRRYGALKRLGATPLPVWGIIAGKSLAVVAVVFLQAIILGAIGFALGWRPALTALTLGAGIIALGTXGFAALGLLLGGTLRAEIVLAVANLMWFVFAGFGALTLESNVIPTAFKWVARVTPSGALTEALSHGHDRVGGLVRDRRPSGVGRAGRTGRTALVPVHLNRPPGFATLQPRTRHADASVRGCSFDTGPPVLQRVVFGALRSGGVXQPACPAGHRRNRHPHPGRHRRHRGNRPGYRLRPGVSDLAAVFSG

>293|CORE_REP|Org107_Gene3555#

MPELNTARGPIDTADLGVTLMHEHVFIMTTEIAQNYPEAWGDEDKRVAGAIARLGELKARGVDTIVDLTVIGLGRYIPRIARVAAATELNIVVATGLYTYNDVPFYFHYLGPGAQLDGPEIMTDMFVRDIEHGIADTGIKAGILKCATDEPGLTPGVERVLRAVAQAHKRTGAPISTHTHAGLRRGLDQQRIFAEEGVDLSRVVIGHCGDSTDVGYLEELIAAGSYLGMDRFGVDVISPFQDRVNIVARMCERGHADKMVLSHDACCYFDALPEELVPVAMPNWHYLHIHNDVIPALKQHGVTDEQLHTMLVDNPRRIFERQGGYQ

>294|CORE_REP|Org16_Gene407#

MIPVLPPLEALLDRLYVVALPMRVRFRGITTREVALIEGPAGWGEFGAFVEYQSAQACAWLASAIETAYCAPPPVRRDRVPINATVPAVAAAQVGEVLARFPGARTAKVKVAEPGQSLADDIERVNAVRELVPMVRVDANGGWGVAEAVAAAAALTADGPLEYLEQPCATVAELAELRRRVDVPIAADESIRKAEDPLAVVRAQAADIAVLKVAPLGGISALLDIAARIAVPVVVSSALDSAVGIAAGLTAAAALPELDHACGLGTGGLFEEDVAEPAAPVDGFLAVARTTPDPARLQALGAPPQRRQWWIDRVKACYSLLVPSFG

>296|CORE_REP|Org111_Gene2650#

MNSEHPMTDRVVYRSLMADNLRWDALQLRDGDIIISAPSKSGLTWTQRLVSLLVFDGPDLPGPLSTVSPWLDQTIRPIEEVVATLDAQQHRRFIKTHTPLDGLVLDDRVSYICVGRDPRDAAVSMLYQSANMNEDRMRILHEAVVPFHERIAPPFAELGHARSPTEEFRDWMEGPNQPPPGIGFTHLKGIGTLANILHQLGTVWVRRHLPNVALFHYADYQADLAGELLRPARVLGIAATRDRARDLAQYATLDAMRSRASEIAPNTTDGIWHSDERFFRRGGSGDWQQFFTEAEHLRYYHRINQLAPPDLLAWAHEGRRGYDPAN

>297|CORE_REP|Org1_Gene1170#

MMIVVLVDPRRPTLVPVEAIEFLRGEVQYTEEMPVAVPWSLPAARSAHAGNDAPVLLSSDPNHPAVITRLAAGARLISAPDSQRGERLVDAVAMMDKLRTAGPWESEQTHDSLRRYLLEETYELLDAVRSGSVDQLREELGDLLLQVLFHARIAEDASQSPFTIDDVADTLMRKLGNRAPGVLAGESISLEDQLAQWEAAKASEKARKSVADDVHTGQPALALAQKVIQRAQKAGLPAHLIPDEITSVSVSADVDAENTLRTAVLDFIDRLRCAERAIAVARRGSNVAEQLDVTPLGVITEQEWLAHWPTAVNDSRGGSKKRKGMR

>298|CORE_REP|Org44_Gene896#

MTRYLARRLLNYLVLLALASFLTYCLTSLAFSPLESLMQRSPRPPQAVIDAKAHDLGLDRPILARYANWVSHAVRGDFGTTITGQPVGTELGRRIGVSLRLLVVGSVFGTVAGVVIGAWGAIRQYRLSDRVMTTLALLVLSTPTFVVANLLILGALRVNWAVGIQLFDYTGETSPGVAGGVWDRLGDRLQHLILPSLTLALAAAAGFSRYQRNAMLDVLGQDFIRTARAKGLTRRRALLKHGLRTALIPMATLFAYGVAGLVTGAVFVEKIFGWHGMGEWMVRGISTQDTNIVAAITVFSGAVVLLAGLLSDVIYAALDPRVRVS

>299|CORE_REP|Org28_Gene921#

MTSAPATMRWGNLPLAGESGTMTLRQAIDLAAALLAEAGVDSARCDAEQLAAHLAGTDRGRLPLFEPPGDEFFGRYRDIVTARARRVPLQHLIGTVSFGPVVLHVGPGVFVPRPETEAILAWATAQSLPARPLIVDACTGSGALAVALAQHRANLGLKARIIGIDDSDCALDYARRNAAGTPVELVRADVTTPCLLPELDGQVDLMVSNPPYIPDAAVLEPEVAQHDPHHALFGGPDGMTVISAVVGLAGRWLRPGGLFAVEHDDTTSSSTVDLVSSTKLFVDVQARKDLAGRPRFVTAMRWGHLPLAGENGAIDPRQRRCRAKR

>300|CORE_REP|Org82_Gene638#

MRTEDDSWDVTTSVGSTGLLVAAARALETQKADPLAIDPYAEVFCRAAGGEWADVLDGKLPDHYLTTGDFGEHFVNFQGARTRYFDEYFSRATAAGMKQVVILAAGLDSRAFRLQWPIGTTIFELDRPQVLDFKNAVLADYHIRPRAQRRSVAVDLRDEWQIALCNNGFDANRPSAWIAEGLLVYLSAEAQQRLFIGIDTLASPGSHVAVEEATPLDPCEFAAKLERERAANAQGDPRRFFQMVYNERWARATEWFDERGWRATATPLAEYLRRVGRAVPEADTEAAPMVTAITFVSAVRTGLVADPARTSPSSTSIGFKRFEAD

>301|CORE_REP|Org59_Gene1525#

MSFLTVAPDMVTAAAGNLESVGSALNEAAAAAAPATVGLAAPAADRVSAVVAAMLGAYARDFQGISAQIAGFHNQFVGALRGGAAAYASAEAANVQQTVVNAVNAPAQALLGHPLIGPETVGSSAAAVSFGFGPLLLAGSDPLLAVPFSYPASLPTPFGPVTMTLNGSFDPLTQQVVFDSGSLTAPAPFVYGLGAVGPALTTMTALQNSGTAFSGAVQSGNLLGAAGALLQAPGNAVTGFLFGQTAISQSIPGPSNLGYESVGISVPVGGLLAPLQPVTVTLTPTSGMPTAIQLSGTQFGGLLPALLNXFXPSADSRRKPRDQRV

>302|CORE_REP|Org118_Gene1551#

MIYRVACLLARIRFTVGYVAALASVSTTILMHGPQVHAQVIRHASTNLHNLAHGHLGTLWNSAFVIDEGPLYFWLPCLACLLAVAELQLRSLRLTVAFVVGHIGATLLVAAVLAGAIEIGWLPWSISRVSDVGMSYGALAALGALTAAIPGRWRPAWIGWWVSLGLATATIGGGFTDAGHTVALLLGMLVTACFTRPARWTLGRCALLAVASGFCLVLLAHSWWSLVSGSALGLLGALGAAGFARWTRARANIAATRRAGDSAAGAKSLSPAQRVPSRADRITYDLHLRHAVSGQVGYVHQSSFRSATMRAVRIASSARRVAPRG

>303|CORE_REP|Org88_Gene387#

MNTLHVNVGLARYSDWAFTSAVVALVVALLLLAFEFAQVRGRGLAPLAVPAGSVATDSATPGIVADQRHRPFDERVGRGGLAVAYLGIGLLLACVVLRGLATQRVPWGNMYEFINLTCLSGLIAGAVVLRRARYRPLWVFLLVPVLILLTVSGRWLYANAAPVMPALQSYWLPIHVSVVSLGSGVFLVAGVASILFLVRTSRLGEPTGEGALAGMVRRLPDAQTLDGIAYRTTIFAFPVFGFGVIFGAIWAEEAWGRYWGWDPKETVSFVAWVVYAAYLHARSTAGWRDRKAAWINVAGFVAMVFNLFFVNLVTVGLHSYAGVG

>304|CORE_REP|Org87_Gene3414#

MARDQGADEAREYEPGQPGMYELEFPAPQLSSSDGRGPVLVHALEGFSDAGHAIRLAAAHLKAALDTELVASFAIDELLDYRSRRPLMTFKTDHFTHSDDPELSLYALRDSIGTPFLLLAGLEPDLKWERFITAVRLLAERLGVRQTIGLGTVPMAVPHTRPITMTAHSNNRELISDFQPSISEIQVPGSASNLLEYRMAQHGHEVVGFTVHVPHYLTQTDYPAAAQALLEQVAKTGSLQLPLAVLAEAAAEVQAKIDEQVQASAEVAQVVAALERQYDAFIDAQENRSLLTRDEDLPSGDELGAEFERFLAQQAEKKSDDDPT

>305|CORE_REP|Org89_Gene3641#

MALVSTARVDLVCEGGGVRGIGLVGAVDALADAGYRFPRVAGSSAGAIVASLVAALQTAGEPVTRLAEMMRSIDYPKFLDRNLIGHVPLIGGGLSLLLSDGVYRGAYLEQLLGGLLADLGVHTFGDLRTGEAPEQFAWSLVVTASDLSRRRLVRIPWDLDSYGIHPDDFSVARAVHASSAIPFVFEPVRVRGATWVDGGLLSNFPVALFDRTDAEPRWPTFGIRLSARPGTPPTRPVQGPVSLGIAAIETLVSNQDNAYIDDPCTVRRTIFVPAHDVSPIDFDITAEQREALYQRGFQAGQKFLANWNYADYLADCGGPFTPSL

>306|CORE_REP|Org65_Gene2020#

MTGNAKLIDRVSAINWNRLQDEKDAEVWDRLTGNFWLPEKVPVSNDIPSWGTLTAGEKQLTMRVFTGLTMLDTIQGTVGAVSLIPDALTPHEEAVLTNIAFMESVHAKSYSQIFSTLCSTAEIDDAFRWSEENRNLQRKAEIVLQYYRGDEPLKRKVASTLLESFLFYSGFYLPMYWSSRAKLTNTADMIRLIIRDEAVHGYYIGYKFQRGLALVDDVTRAELKDYTYELLFELYDNEVEYTQDLYDEVGLTEDVKKFLRYNANKALMNLGYEALFPRDETDVNPAILSALSPNADENHDFFSGSGSSYVIGKAVVTEDDDWDF

>307|CORE_REP|Org59_Gene2714#

MLRSNTAHIGRTAPDQPENRPTGALEVKLCAHAXSSXPPTAALCACAVTVSAGAAWADADVQPAGSVPIPDGPAQTWIVADLDSGQVLAGRDQNVAHPPASTIKVLLALVALDELDLNSTVVADVADTQAECNCVGVKPGRSYTARQLLDGLLLVSGNDAANTLAHMLGGQDVTVAKMNAKAATLGATSTHATTPSGLDGPGGSGASTAHDLVVIFRAAMANPVFAQITAEPSAMFPSDNGEQLIVNQDELLQRYPGAIGGKRXGYTNAARKTFVGAAARGGRRLVIAMMYGLVKEGGPTYWDQAATLFDWGFALNPQASVGSL

>308|CORE_REP|Org59_Gene3345#

MALDLTAYFDRINYRGATDPTLDVLQDLVTVHSRTIPFENLDPLLGVPVDDLSPQALADKLVLRRRGGYCFEHNGLMGYVLAELGYRVRRFAARVVWKLAPDAPLPPQTHTLLGVTFPGSGGCYLVDVGFGGPNNPTSPLRLETGAVQPTTHEPYRLXDXXXGFVXQAMVRDTWQTLYEFTTQTRPQIDLKVASWYASTHPASKFVTGLTAAVITDDARWNLSGRDLAVHRAGGTEKIRLADAAAVVDTLSERFGINVADIGERGALETRIDELXAXXPGXDAPXGFFDVAAWPGKRYPNKRTPPCGVEIDSGCDRNTGVGQQL

>309|CORE_REP|Org28_Gene3918#

MLATLSQIRAWSTEHLIDAAGYWTETADRWEDVFLQMRNQAHAIAWNGAGGDGLRQRTRADFSTVSGIADQLRRAATIARNGAGTIDAAQRRVMYAVEDAQDAGFNVGEDLSVTDTKTTQPAAVQAARLAQAQALAGDIRLRVGQLVAAENEVSGQLAATTGDVGNVRFAGAPVVAHSAVPLVDFFKQDGPTPPPPGAPHPSGGADGPYSDPITSMMLPPAGTEAPVSDATKRWVDNMVNELAARPPDDPIAVEARRLAFQALHRPCNSAEWTAAVAGFAGSSAGVVGTALAIPAGPADWALLGAALLGVGGSGAAVVNCATK

>310|CORE_REP|Org95_Gene3181#

MPGNSRRRGAVRKSGTKKGAGVGSGGQRRRGLEGRGPTPPAHLRPHHPAAKRARAQPRRPVKRADETETVLGRNPVLECLRAGVPATALYVALGTEADERLTECVARAADSGIAIVELLRADLDRMTANHLHQGIALQVPPYNYAHPDDLLAAALDQPPALLVALDNLSDPRNLGAIVRSVAAFGGHGVLIPQRRSASVTAVAWRTSAGAAARIPVARATNLTRTLKGWADRGVRVIGLDAGGGTALDDVDGTDSLVVVVGSEGKGLSRLVRQNCDEVVSIPMAAQAESLNASVAAGVVLAEIARQRRRPREPREQTQNRMI

>311|CORE_REP|Org118_Gene1438#

MNAHTSVGPLDRAARVYIAGHRGLVGSALLRTFAGAGFTNLLVRSRAELDLTDRAATFDFVLESRPQVVIDAAARVGGILANDTYPADFLSENLQIQVNLLDAAVAARVPRLLFLGSSCIYPKLAPQPIPESALLTGPLEPTNDAYAIAKIAGILAVQAVRRQHGLPWISAMPTNLYGPGDNFSPSGSHLLPALIRRYDEAKASGAPNVTNWGTGTPRRELLHVDDLASACLYLLEHFDGPTHVNVGTGIDHTIGEIAEMVASAVGYSGETRWDPSKPDGTPRKLLDVSVLREAGWRPSIALRDGIEATVAWYREHAGTVRQ

>312|CORE_REP|Org59_Gene3549#

MAAVRLGXSWXPXATLKACGSGFGCAAWAVGRSLASLXDLHDIHVMLKLASRHSRGHWPASEAIWSTGSAATSCGCTWAGRXSSSGTAARCWGRSGSPSPPERPPSRWAACIPSCFGSELSEHLPYVTLGLIVWNLINAAILDGAEVFVANEGLIKQLPAPLSVHVYRLVWRXXIFFAXNIVIYFVIAIIFPKPWSWADLSFLPALALIFLNCVWVSLCFGILATRYRDIGPLLFSVVQLLFFMTPIIWNDETLRRQGXGRWSSIVELNPLLHYLDIVRAPLLGAHQELRHWLXVLVXXVVGWMLAAFAMRQXRARXPYWV

>313|CORE_REP|Org82_Gene2724#

MAIGEQQVIVIGAGVSGLTSAICLAEAGWPVRVWAAALPQQTTSAVAGAVWGPRPKEPVAKVRGWIEQSLHVFRDLAKDPATGVRMTPALSVGDRIETGAMPPGLELIPDVRPADPADVPGGFRAGFHATLPMIDMPQYLDCLTQRLAATGCEIETRPLRSLAEAAEAAPIVINCAGLGARELAGDATVWPRFGQHVVLTNPGLEQLFIERTGGSEWICYFAHPQRVVCGGISIPGRWDPTPEPEITERILQRCRRIQPRLAEAAVIETITGLRPDRPSVRVEAEPIGRALCIHNYGHGGDGVTLSWGCAREVVNLVGGG

>315|CORE_REP|Org128_Gene2716#

MPAGLPGQASVAVRLSCDVPPDARHHEPRPGMTDHPDTGNGIGLTGRPPRAIPDPAPRSSHGPAKVIAMCNQKGGVGKTTSTINLGAALGEYGRRVLLVDMDPQGALSAGLGVPHYELDKTIHNVLVEPRVSIDDVLIHSRVKNMDLVPSNIDLSAAEIQLVNEVGREQTLARALYPVLDRYDYVLIDCQPSLGLLTVNGLACTDGVIIPTECEFFSLRGLALLTDTVDKVRDRLNPKLDISGILITRYDPRTVNSREVMARVVERFGDLVFDTVITRTVRFPETSVAGEPITTWAPKSAGALAYRALARELIDRFGM

>316|CORE_REP|Org49_Gene727#

MCCTSGCALTIRLLGRTEIRRLAKELDFRPRKSLGQNFVHDANTVRRVVAASGVSRSDLVLEVGPGLGSLTLALLDRGATVTAVEIDPLLASRLQQTVAEHSHSEVHRLTVVNRDVLALRREDLAAAPTAVVANLPYNVAVPALLHLLVEFPSIRVVTVMVQAEVAERLAAEPGSKEYGVPSVKLRFFGRVRRCGMVSPTVFWPIPRVYSGLVRIDRYETSPWPTDDAFRRRVFELVDIAFAQRRKTSRNAFVQWAGSGSESANRLLAASIDPARRGETLSIDDFVRLLRRSGGSDEATSTGRDARAPDISGHASAS

>317|CORE_REP|Org59_Gene91#

MSYLAGAAQIGGVMVGAPLVIGMTRQVRARWEGRAGAGLLQPWRDLLKQLGKQQITPAGTTIVFAAAPVIVAGTTLLIAAIAPLVATGSPLDPSADLFAVVGLLFLGTVALTLAGIDTGTSFGGMGASREITIAALVEPTILLAVFALSIPAGSANLGALVASTIDHPGHVVSLAGVLAFVALVIVIVAETGRLPVDNPATHLELTMVHEAMXXEYAGXRJLALVEWAAGMRLTVLLALLANLFLPWGIAGAAPTALDVLTGVVAVAAKVAILAVLLATFEVFLAKLRLFRVPELLAGSFLLALLAVTAANFFTVGA

>318|CORE_REP|Org7_Gene3486#

MTYSPGNPGYPQAQPAGSYGGVTPSFAHADEGASKLPMYLNIAVAVLGLAAYFASFGPMFTLSTELGGGDGAVSGDTGLPVGVALLAALLAGVALVPKAKSHVTVVAVLGVLGVFLMVSATFNKPSAYSTGWALWVVLAFIVFQAVAAVLALLVETGAITAPAPRPKFDPYGQYGRYGQYGQYGVQPGGYYGQQGAQQAAGLQSPGPQQSPQPPGYGSQYGGYSSSPSQSGSGYTAQPPAQPPAQSGSQQSHQGPSTPPTGFPSFSPPPPVSAGTGSQAGSAPVNYSNPSGGEQSSSPGGRRSNRAFPRPVARVREE

>319|CORE_REP|Org70_Gene916#

MVTQALLPSGLVASAVVAASSANLGPGFDSVGLALSLYDEIIVETTDSGLTVTVDGEGGDQVPLGPEHLVVRAVQHGLQAAGVSAAGLAVRCRNAIPHSRGLGSSAAAVVGGLAAVNGLVVQTDSSPSSDAELIQLASEFEGHPDNAAAAVLGGAVVSWTDHSGDRPNYSAVSLRLHPDIRLFTAIPEQRSSTAETRVLLPAQVSHDDARFNVSRAALLVVALTERPDLLMAATEDLLHQPQRAAAMTASAEYLRLLRRHNVAAALSGAGPSLIALSTDSELPTDAVEFGAAKGFAVTELTVGEAVRWSPTVRVPG

>320|CORE_REP|Org119_Gene1901#

MAPPNRDELLAAVERSPQAAAAHDRAGWVGLFTGDARVEDPVGSQPQVGHEAIGRFYDTFIGPRDITFHRDLDIVSGTVVLRDLELEVAMDSAVTVFIPAFLRYDLRPVTGEWQIAALRAYWELPAMMLQFLRTGSGATRPALQLSRALLGNQGLGGTAGFLTGFRRAGRRHKKLVETFLNAASRADKSAAYHALSRTATMTLGEDELLDIVELFEQLRGASWTKVTGAGSTVAVSLASDHRRGIMFADRAVARQPDQSDSVLPSLTVVVPQDPKSGARWSISRSSGAVCYGVRARLLRPRWLPGPLGVLPMTMTR

>321|CORE_REP|Org59_Gene2995#

MGSTRLTGVNVEPPPEHVLVAFGLAGAQPILLGAGWEGGWRCGEVVLSMVADNARAAWSARVRETLFVDGVRLARPVRSTDGRYVVSGWRADTFVAGAPEPRHDEVVSAAVRLHEATGKLERPRFLTQGPAAPWAEIDVFVAADRAGWEERPLQSVRXRXXPTAPPAADPQRSIDLINQLAGLRKPTKSPNQLVHGDLYGTVLFAGTAPPGITDITPYWRPASWAAGVAVVDALSWGAADDGLIERWNALPEWPQMLLRALMFRLAVYALHPRSTAEALXRPGPHRGPSAAXALNLLSPAETHSGSRSAPPGRQH

>322|CORE_REP|Org114_Gene3134#

MNSRAPRNLAVSSPSAQVTGRMVQNGENLFQFRREGPQVQLSFQDRTYLVTGGGSGIGKGVAAGLVAAGAAVMIVGRNPDKLAAAVKDIEALKTGAIGYEPADITDEEQTLRVVDAATAWHGRLHGVVHCAGGSQTIGPITQIDSQAWRRTVDLNVNGTMYVLKHAARELVRGGGGSFVGISSIAASNTHRWFGAYGVTKSAVDHMMKLAADELGPSWVRVNSIRPGLIRTDLVVPVTESPELSADYRVCTPLPRVGEVEDVANLAMFLLSDAASWITGQVINVDGGHMLRRGPDFSPMLEPVFGADGLRGVVG

>324|CORE_REP|Org2_Gene2504#

MTPSEGNAPLPELHNTVVVAAFEGWNDAGDAASDAVAHLAASWQALPIVEIDDEAYYDYQVNRPVIRQVDGVTRELQWPAMRISHCRPPGSDRDVVLMCGVEPNMRWRTFCDELLAVIDKLNVDTVVILGALLADTPHTRPVPVSGAAYSAASARQFGLQETRYEGPTGIAGVFQSACVGAGIPAVTFWAAVPHYVSHPPNPKATIALLRRVEDVLDVEVPLADLPAQAEAWEREITETIAEDHELAEYVQTLEQHGDAAVDMNEALGNIDGDALAAEFERYLRRRRPGSGARGRLRCGGRRCQPGGPGSPESP

>325|CORE_REP|Org59_Gene286#

MDATPNAVELTVDNAWFIAETIGAGTFPWVLAITMPYSDAAQRGAFVDRQRDELTRMGLLSPQGVINPAVADWIKVVCFPDRWLDLRYVGPASADGACELLRGIVALRTGTGKTSNKTGNGVVALRNAQLVTFTAMDIDDPRALVPILGXGLAHRPPARFDEFSLPTRVGARADERLRSGVPLGEVVDYLGIPASARPVVESVFSGPRSYVEIVAGCNRSXERRXHHHRGRPKHRRHLGGPGVGESVAGIRRRVGLHLQPWDTVCDRRRDPNTDRVLARRAMVPGTAGVAGLLHPILVIRNQKVSTMSQERSR

>326|CORE_REP|Org2_Gene2568#

MVALYGACICSQGGRSFLEVFHWLQHDIVDRGRLPLLCCLVAFVLTFLVTRSFVRFIHRRAADGRPARWWQPRNVHIGSVHIHHVAFGVVLVMISGLTLVTLSVDGREPEFTIAASIFGVGAALVLDEYALILHLSDVYWEEDGRTSVDAVFAAVAVAGLLIMGLHPLIFFLPVRQGANWVVLQTTLIAGLVLTLPLAVVVLLKGKVWTGLLGMFVVVLLVVGAVRLSRPHAPWARWRYTRHPEKMRRALQRERTWRRPVVRIKLWLQYVIAGTPRMPDERAVDAQLDQDVRPAPPPERTAPILISGSVWSD

>329|CORE_REP|Org2_Gene1470#

MDRAQYPQARANCICRGDYRLRHFGVRITRILALLLAVLLAVSGVAGCSADTGDRHPELVVGSTPDSEAMLLAAIYVAALRSYGFAAHAETAADPVAKLDSGAFTVVPAFTGQMLQTLQPDASVRSDAQVYRAIVSALPEGIAAGDYTTAAEDKPALVVTQSTAKAWGGGDLSELPSHCRGLLVGRVAGAHTPAAVGPCRLPAPREFRNDATMFAALRAGQLVAAWTTTADPDIPADLIMLTDGKPALIRAENIVPLYRRNALTERQLLAVNEVAGVLDTTALIGMRRQVAAGADPAAVAAGWLAEHPLGR

>330|CORE_REP|Org12_Gene3383#

MGKGSMTAHATPNEPDYPPPPGGPPPPADIGRLLLRCHDRPGIIAAVSTFLARAGANIISLDQHSTAPEGGTFLQRAIFHLPGLTAAVDELQRDFGSTVADKFGIDYRFAEAAKPKRVAIMASTEDHCLLDLLWRNRRGELELSVVMVIANHPDLAAHVRPFGVPFIHIPATRDTRTEAEQRQLQLLSGNVDLVVLARYMQILSPGFLEAIGCPLINIHHSFLPAFTGAAPYQRARERGVKLIGATAHYVTEVLDEGPIIEQDVVRVDHTHTVDDLVRVGADVERAVLSRAVLWHCQDRVIVHHNQTIVF

>331|CORE_REP|Org93_Gene3373#

MIQIARTWRVFAGGMATGFIGVVLVTAGKASADPLLPPPPIPAPVSAPATVPPVQNLTALPGGSSNRFSPAPAPAPIASPIPVGAPGSTAVPPLPPPVTPAISGTLRDHLREKGVKLEAQRPHGFKALDITLPMPPRWTQVPDPNVPDAFVVIADRLGNSVYTSNAQLVVYRLIGDFDPAEAITHGYIDSQKLLAWQTTNASMANFDGFPSSIIEGTYRENDMTLNTSRRHVIATSGADKYLVSLSVTTALSQAVTDGPATDAIVNGFQVVAHAAPAQAPAPAPGSAPVGLPGQAPGYPPAGTLTPVPPR

>332|CORE_REP|Org119_Gene1433#

MADRSMPVPDGLAGMRVDTGLARLLGLSRTAAAALAEEGAVELNGVPAGKSDRLVSGALLQVRLPEAPAPLQNTPIDIEGMTILYSDDDIVAVDKPAAVAAHASVGWTGPTVLGGLAAAGYRITTSGVHERQGIVHRLDVGTSGVMVVAISGWGAYTVLKRAFKYRTVDKRYHALVQGHPDPSSGTIDAPIGRHRGHEWKFAITKNGRHSLTHYDTLEAFVAASLLDVHLETGRTHQIRVHFAALHHPCCGDLVYGADPKLAKRLGLDRQWLHARSLAFAHPADGRRVEIVSPYPADLQHALKILRGEG

>333|CORE_REP|Org142_Gene1372#

MRAAGLLKRLNPRNRRSRVNPDATMSLVDHLTELRTRLLISLAAILVTTIFGFVWYSHSIFGLDSLGEWLRHPYCALPQSARADISADGECRLLATAPFDQFMLRLKVGMAAGIVLACPVWFYQLWAFITPGLYQRERRFAVAFVIPAAVLFVAGAVLAYLVLSKALGFLLTVGSDVQVTALSGDRYFGFLLNLLVVFGVSFEFPLLIVMLNLAGLLTYERLKSWRRGLIFAMFVFAAIFTPGSDPFSMTALGAALTVLLELAIQIARVHDKRKAKREAAIPDDEASVIDPPSPVPAPSVIGSHDDVT

>334|CORE_REP|Org124_Gene2030#

MTTSGTVLATSIAQHWHNFWRGEIGDWILNRGLRIVMLLIAAVLAARFVTWLANRVTRRLDLGFTESDALVRSEATKHRQAVASVISWVSIVLIYVVVVYEVIDVLPVPVGALVGPAAVLGAALGFGAQRLVQDLLAGFFIIVEKQYGFGDLVELSMVGSPENAAGTVEDVTLRVTKLRSSEGEVFTVPNGNIVKSVNLSKDWARAVVDIPVPTSADLGRVNEVLHQECEHARHDSLLGELLLDEPTVMGVERIEVDTVTLRLVARTLPGKQFEAGRQLRVLVIRALTRAGIVTAADARAAVAESPEQ

>335|CORE_REP|Org40_Gene3344#

MDLSASRSDGGDPLRPASPRLRSPVSDGGDPLRPASPRLRSPVSDGGDPLRPASPRLRSPLGASRPVVGLTAYLEQVRTGVWDIPAGYLPADYFEGITMAGGVAVLLPPQPVDPESVGCVLDSLHALVITGGYDLDPAAYGQEPHPATDHPRPGRDAWEFALLRGALQRGMPVLGICRGTQVLNVALGGTLHQHLPDILGHSGHRAGNGVFTRLPVHTASGTRLAELIGESADVPCYHHQAIDQVGEGLVVSAVDVDGVIEALELPGDTFVLAVQWHPEKSLDDLRLFKALVDAASGYAGRQSQAEPR

>336|CORE_REP|Org113_Gene2614#

MNVRGRVAPRRVTGRAMSTLLAYLALTKPRVIELLLVTAIPAMLLADRGAIHPLLMLNTLVGGMMAAAGANTLNCVADADIDKVMKRTARRPLAREAVPTRNALALGLTLTVISFFWLWCATNLLAGVLALVTVAFYVFVYTLWLKRRTSQNVVWGGAAGCMPVMIGWSAITGTIAWPALAMFAIIFFWTPPHTWALAMRYKQDYQVAGVPMLPAVATERQVTKQILIYTWLTVAATLVLALATSWLYGAVALVAGGWFLTMAHQLYAGVRAGEPVRPLRLFLQSNNYLAVVFCALAVDSVIALPTLH

>337|CORE_REP|Org78_Gene2025#

MTSMYEQVDTNTADPVAGSRIDPVLARSWLLVNGAHGDRFESAAHSRADIVVLDIEDAVAPKDKHAARDNAVRWFGDGNADWVRINGFGTPWWADDLAMLADSPVGGVMLAMVESVDHVTETAKRLPNVPIVALVETARGLERINEIAAAKGTFRLAFGIGDFRRDTGFGEDPATLAYARSRFTIAARAAGLPSAIDGPTIGSNALKLIEATAVSAEFGMTGKICLSPDQCPVVNEGLSPSQDEIVWAKEFFAEFARDGGEIRNGSDLPRIARATKILDLARAYGIEVSDFEDEPVHMPAPTDTYHY

>338|CORE_REP|Org42_Gene1382#

MRNRGFGRRELLVAMAMLVSVTGCARHASGARPASTTLPAGADLADRFAELERRYDARLGVYVPATGTTAAIEYRADERFAFCSTFKAPLVAAVLHQNPLTHLDKLITYTSDDIRSISPVAQQHVQTGMTIGQLCDAAIRYSDGTAANLLLADLGGPGGGTAAFTGYLRSLGDTVSRLDAEEPELNRDPPGDERDTTTPHAIALVLQQLVLGNALPPDKRALLTDWMARNTTGAKRIRAGFPADWKVIDKTGTGDYGRANDIAVVWSPTGVPYVVAVMSDRAGGGYDAEPREALLAEAATCVAGVLA

>339|CORE_REP|Org30_Gene950#

MDRCCQRATAFACALRPTKLIDYEEMFRGAMQARAMVANPDQWADSDRDQVNTRHYLSTSMRVALDRGEFFLVYQPIIRLADNRIIGAEALLRWEHPTLGTLLPGRFIDRAENNGLMVPLTAFVLEQACRHVRSWRDHSTDPQPFVSVNVSASTICDPGFLVLVEGVLGETGLPAHALQLELAEDARLSRDEKAVTRLQELSALGVGIAIDDFGIGFSSLAYLPRLPVDVVKLGGKFIECLDGDIQARLANEQITRAMIDLGDKLGITVTAKLVETPSQAARLRAFGCKAAQGWHFAKALPVDFFRE

>340|CORE_REP|Org118_Gene2558#

MASVEFATILALGAALLAGIGYVTLQRSARQVTAEEYVGHFTLFHLSLRHALWWLGSLAAVASFTLQAIALTMGSVVLVQSLQATALLFALLIDARLTHHRCTPREWMWAVLLAGAVAVIVMSGNPAAGTTRAPFSTWAVVAVVVVPAVVLCVVGARIASGSLSAVLLAVASSATLAVFTVLTKGVVTELGEGFATLIRTPELYAWILVLPIGLMLQQSSLRVGALTASLPTITVARPVIASVLGITVLDEVLHTGRVALVALVAAVVVVVVATVALARDEVRDDDGFRRRIGGRGPACGSLTVSGQ

>341|CORE_REP|Org1_Gene3267#

MAAISGGNFRTGVTLVTFVGVTQHTSTMTDPFLGSEALAAGVLTPYELRSRYVALHKDVYVPQGVELTAQLRAKALWLRSRRRGVLAGYSASAFHGAKWIDADLPAAIIDTNRRRAPGLQVWEERIEPDEICVIEGMRVTTPERTALDLTSRFPLDPAVAAVDALIQATDLKVADVEPLIERYRGRRGMKAARAALDLVDGGAQSPKETWLRLLLIRAGFPRPQTQIAVRNEWGWAEAHLDMGWQDIKVAAEYDGDHHLTSRYHYRKDILRHEKVQHRYGWIVVRVVAEDHPADIIRRVGEARAFRA

>342|CORE_REP|Org48_Gene207#

MAISLVAHQPIPHVERPMADPPRLQLARRRRSAAGPGGNEDSLMGVALLAGPANVIMELAMPGVGYGVLESRVESGRLDRHPIKRARTTFTYVAVAVAGSDDQKAAFRRAVNKVHAQVYSTPESPVSYHAFDPELQLWVAACLYKGGVDVYRTFVGEMDDEEADHHYRAGMAMGTTLQVPPQMWPPDRAAFDRYWRQSLDRVHIDDVVRDYLYPIVALRIRGIALPGPLRRLSEGIALLITTGFLPQRFRDEMRLPWDATKQRRFDALMAVLRTVNRLMPRFVREFPFNLMLWDLDRRMRRGRPLV

>343|CORE_REP|Org46_Gene713#

MSRPEVLTPFTAIVPAAGLGTRFLPATKTVPKELLPVVDTPGIELVAAEAAAAGAERLVIVTSEGKDGVVAHFVEDLVLEGTLEARGKIAMLAKVRRAPALIKVESVVQAEPLGLGHAIGCVEPTLSPDEDAVAVLLPDDLVLPTGVLETMSKVRASRGGTVLCAIEVAREEISAYGVFDVEPVPDGDYTDDPNVLKVRGMVEKPKAETAPSRYAAAGRYVLDRAIFDALRRIDRGAGGEVQLTDAIALLIAEGHPVHVVVHQGSRHDLGNPGGYLKAAVDFALDRDDYGPDLRRWLVARLGLTEQ

>344|CORE_REP|Org1_Gene4070#

MRPKLGRPNIARYSNRFSVPTARSDAPLSVTWMGVATLLVDDGSSALMTDGYFSRPGLARVAAGKVSPSAERVDGCLARANVSRLTAVIPVHTHIDHAMDSALVADRTGAQLVGGESAANVGRGYGLPEESLVVAVPGEPIQLGAFDVTLVESHHCPPDRFPGVISAPLTPPVKASAYRCGEAWSTLVHHRPSGRRLLIQDSAGFVSGALAGYRADAAYLSVGQLGLQPPSYLLEYWTETVRTVGVRRVILIHWDDFFRPLSKPLRALPYAADDLDLSIRILDELAAQDGVALQMPTVWRREDPWM

>345|CORE_REP|Org2_Gene1897#

MTSSRVVPDGKPMSDFDELLAVLDLNAVASDLFTGSHPSKNPLRTFGGQLMAQSFVASSRTLTRHHLPPSAFSVHFINGGDTAKDIEFQVIRLRDERRFANRRVDAVQDGTLLSSAMVSYMAGGRGLEHALDPPQVAEPHTRPPIGELLRGYEETVPHFVNALQPIEWRYANDPAWIMRDKGDRLAYNRVWVKALGEMPDDPVLHTATLLYSSDTTVLDSVITTHGLSWGFDRIFAASANHSVWFHRQVNFDDWVLYSTSSPVAADSRGLGSGHFFDRSGKLIATVVQEGVLKYFPATPDSAAGRS

>346|CORE_REP|Org13_Gene986#

MAATLRELRGRIRSAGSIKKITKAQELIATSRIARAQARLESARPYAFEITRMLTTLAAEAALDHPLLVERPEPKRAGVLVVSSDRGLCGAYNANIFRRSEELFSLLREAGKQPVLYVVGRKAQNYYSFRNWNITESWMGFSEQPTYENAAEIASTLVDAFLLGTDNGEDQRSDSGEGVDELHIVYTEFKSMLSQSAEAHRIAPMVVEYVEEDIGPRTLYSFEPDATMLFESLLPRYLTTRVYAALLESAASELASRQRAMKSATDNADDLIKALTLMANRERQAQITQEISEIVGGANALAEAR

>348|CORE_REP|Org28_Gene47#

MTAPNEPGALSKGDGPNADGLVDRGGAHRAATGPGRIPDAGDPPPWQRAATRQSQAGHRQPPPVSHPEGRPTNPPAAADARLNRFISGASAPVTGPAAAVRTPQPDPDASLGCGDGSPAEAYASELPDLSGPTPRAPQRNPAPARPAEGGAGSRGDSAAGSSGGRSITAESRDARVQLSARRSRGPVRASMQIRRIDPWSTLKVSLLLSVALFFVWMITVAFLYLVLGGMGVWAKLNSNVGDLLNNASGSSAELVSSGTIFGGAFLIGLVNIVLMTALATIGAFVYNLITDLIGGIEVTLADRD

>349|CORE_REP|Org98_Gene3124#

MAGRSERLVITGAGGQLGSHLTAQAAREGRDMLALTSSQWDITDPAAAERIIRHGDVVINCAAYTDVDGAESNEAVAYAVNATGPQHLARACARVGARLIHVSTDYVFDGDFGGAEPRPYEPTDETAPQGVYARSKLAGEQAVLAAFPEAAVVRTAWVYTGGTGKDFVAVMRRLAAGHGRVDVVDDQTGSPTYVADLAEALLALADAGVRGRVLHAANEGVVSRFGQARAVFEECGADPQRVRPVSSAQFPRPAPRSSYSALSSRQWALAGLTPLRHWRSALATALAAPANSTSIDRRLPSTRD

>350|CORE_REP|Org118_Gene3334#

MPSKAKVAIVGSGNISTDLLYKLLRSEWLEPRWMVGIDPESDGLARAAKLGLETTHEGVDWLLAQPDKPDLVFEATSAYVHRDAAPKYAEAGIRAIDLTPAAVGPAVIPPANLREHLGAPPSRNMITCGGQATIPIVYAVSRIVEVPYAEIVASVASVSAGPGTRANIDEFTKTTARGVQTIGGAARGKAIIILNPADPPMIMRDTIFCAIPTDADREAIAASIHDVVKEVQTYVPGYRLLNEPQFDEPSINSGGQALVTTFVEVEGAGDYLPPYAGNLDIMTAAATKVGEEIAKETLVVGGAR

>351|CORE_REP|Org23_Gene3838#

MHYGLVLFTSDRGITPAAAARLAESHGFRTFYVPEHTHIPVKRQAAHPTTGDASLPDDRYMRTLDPWVSLGAASAVTSRIRLATAVALPVEHDPITLAKSIATLDHLSHGRVSVGVGFGWNTDELVDHGVPPGRRRTMLREYLEAMRALWTQEEACYDGEFVKFGPSWAWPKPVQPHIPVLVGAAGTEKNFKWIARSADGWITTPRDVDIDEPVKLLQDIWAAAGRDGLPQIVALDVKPVPDKLARWAELGVTEVLFGMPDRSADDAAAYVERLAAKLACCVXGEQTHKPPTRRACGGLCVCSP

>352|CORE_REP|Org61_Gene557#

MMARMPELSRRAVLGLGAGTVLGATSAYAIDMLLQPRTSHAAPAAAIGTNVPLAPTPALDPAPPAQAAPTMSTGSFVSAARAGKMTNWAIARPPGQTQALRPVIALHGLGGSASAVMDGGVEQGLAQAVNAGLPPFAVVSVDGGSSYWHQRASGEDAGAMVLNELIPLLDTQRLDTSRVAFLGWSMGGYGALLLGSRLGPARTAAICAVSPALWLSAGSVAPGSFDGPDDWSANSVFGLPALGSIPIRVDCGNSDPFYAATKQFVAQLPHPPAGGFSPGGHNGGFWSAQLPAELTWFAPLLTG

>353|CORE_REP|Org119_Gene3514#

MSEDVVTQPPANLVAGVVKAIRPRQWVKNVLVLAAPLAALGGGVRYDYVEVLSKVSMAFVVFSLAASAVYLVNDVRDVEADREHPTKRFRPIAAGVVPEWLAYTVAVVLGVTSLAGAWMLTPNLALVMVVYLAMQLAYCFGLKHQAVVDICVVSSAYFVSRAIAGGVATKIPLSKWFLLIMAFGSLFMVAGKRYAELHLAERTGAAIRKSLESYTSTYLRFVWTLSATAVVLCYGLWAFERDGYSGSWFAVSMIPFTIAILRYAVDVDGGLAGEPEDIALRDRVLQLLALAWIATVGAAVAFG

>354|CORE_REP|Org6_Gene2975#

MSGNEVHPDLRRIAVVTPRQLVGPRTLPVMRALIVVAGLRMSRTPPDIEVLTLESGVGVRLYRPAGSNEPAPALLWIHAGGYVMGTAQQDDRLCLRFSSRLGITVASVDYRLAPENPYPAALGDCYSALTWLASLPAVDPARVAIGGASAGGGLAAALALLARDRGGITPAFQLLVYPMLDDRPSIAPANPHYRLWNGRANRFGWRAYLGDADARVAVPGRRDDLGGLAPAWIGVGTHDLLHDEDLAYAERLTAAGVPCQVEVVEGAFHGFDRVAPNVGVSQRFFTSQCNSLRAALALSNRT

>355|CORE_REP|Org65_Gene284#

MRILVAWATCGAVVLSGLTGCSGSSHSGRTYGAQSARTGESLAVLGWNMSVSNLRWSGDYVLIDVDASPTDPHAPHAKPEDIRFGLYGALAHPMESAALGSCGDAMAHVRDVVSPLSAPAGRLTGTVCLGPLKERSAVRGVYTYSPRDRIPGTAAAYPAAFPVGMLPTNQNDAGLVVKTTSVSAWRADGMQLGKPQLGDPVAFTGNGYMLLGLEVDAVPDRYRDDSAARGGPMMLLAAPTLPGRGLSPACATYGSSVLILPDALLDAVHISASLCTQGEINEALLYATVATVGTHAALWTSR

>356|CORE_REP|Org68_Gene2612#

MMNHARGVENRSEGGGIDVVLVTGLSGAGRGTAAKVLEDLGWYVADNLPPQLITRMVDFGLAAGSRITQLAVVMDVRSRGFTGDLDSVRNELATRAITPRVVFMEASDDTLVRRYEQNRRSHPLQGEQTLAEGIAAERRMLAPVRATADLIIDTSTLSVGGLRDSIERAFGGDGGATTSVTVESFGFKYGLPMDADMVMDVRFLPNPHWVDELRPLTGQHPAVRDYVLHRPGAAEFLESYHRLLSLVVDGYRREGKRYMTIAIGCTGGKHRSVAIAEALMGLLRSDQQLSVRALHRDLGRE

>357|CORE_REP|Org149_Gene3036#

MTRMAEKPISPTKTRTRFEDIQAHYDVSDDFFALFQDPTRTYSCAYFEPPELTLEEAQYAKVDLNLDKLDLKPGMTLLDIGCGWGTTMRRAVERFDVNVIGLTLSKNQHARCEQVLASIDTNRSRQVLLQGWEDFAEPVDRIVSIEAFEHFGHENYDDFFKRCFNIMPADGRMTVQSSVSYHPYEMAARGKKLSFETARFIKFIVTEIFPGGRLPSTEMMVEHGEKAGFTVPEPLSLRPHYIKTLRIWGDTLQSNKDKAIEVTSEEVYNRYMKYLRGCEHYFTDEMLDCSLVTYLKPGAAA

>358|CORE_REP|Org4_Gene3919#

MGWRDAPALSDYQHVASGKVREIYRVDDEHLLLVASDRISAYDYVLDSTIPDKGRVLTAMSAFFFGLVDAPNHLAGPPDDPRIPDEVLGRALVVRRLEMLPVECVARGYLTGSGLLDYQATGKVCGIALPPGLVEASRFATPLFTPATKAALGDHDENISFDRVVEMVGALRANQLRDRTLQTYVQAADHALTRGIIIADTKFEFGIDRHGNLLLADEIFTPDSSRYWPADDYRAGVVQTSFDKQFVRSWLTGSESGWDRGSDRPPPPLPEHIVEATRARYINAYERISELKFDDWIGPGA

>359|CORE_REP|Org25_Gene1706#

MTAFGVEPYGQPKYLEIAGKRMAYIDEGKGDAIVFQHGNPTSSYLWRNIMPHLEGLGRLVACDLIGMGASDKLSPSGPDRYSYGEQRDFLFALWDALDLGDHVVLVLHDWGSALGFDWANQHRDRVQGIAFMEAIVTPMTWADWPPAVRGVFQGFRSPQGEPMALEHNIFVERVLPGAILRQLSDEEMNHYRRPFVNGGEDRRPTLSWPRNLPIDGEPAEVVALVNEYRSWLEETDMPKLFINAEPGAIITGRIRDYVRSWPNQTEITVPGVHFVQEDSPEEIGAAIAQFVRRLRSAAGV

>360|CORE_REP|Org13_Gene2235#

MNVLSLGSSSGVVWGRVPITAPAGAATGVTSRADAHSQMRRYAQTGPTAKLSSAPMTTMWGAPLHRRWRGSRLRDPRQAKFLTLASLKWVLANRAYTPWYLVRYWRLLRFKLANPHIITRGMVFLGKGVEIHATPELAQLEIGRWVHIGDKNTIRAHEGSLRFGDKVVLGRDNVINTYLDIEIGDSVLMADWCYICDFDHRMDDITLPIKDQGIIKSPVRIGPDTWIGVKVSVLRGTTIGRGCVLGSHAVVRGAIPDYSIAVGAPAKVVKNRQLSWEASAAQRAELAAALADIERKKAAR

>361|CORE_REP|Org1_Gene3943#

MVLDPLMDPISMAAESFSVHGPGGVRIVADRLGDPRARAVVFLHGGGQTRRSWGRAAAAVAERGWQAVTIDLRGHGESDWSSEGDYRLVSFAGDIQEVLRNLPGQPALVGASLGGFAAMLLAGELSPGIASAVVLVDIVPNMDLAGASRIHAFMAERVESGFGSLDEVADVIANYNPHRPRPSDPDGLVANLRRRGDRWYWHWDPQFIGGIAAFPPVEVTDVDRMNAAVATILRDEVPVLLVRGQVSDIVRQESADQFLSRFPQVEFTDVRGAGHMVAGDRNDAFAGAVLDFLARHVGVR

>362|CORE_REP|Org59_Gene737#

MAKRLLDWPGGLTVFDVRVEAMAPFVEGGATAAASVSDVAEADIISITVFDDAQVXSVITADNGLATHAKPGTIVAIHSTIADTTAVDLAXKLKPQGIHIVDAPVSGGAAAAAKGELAVMVGADDEAFQRIKEPFSRWASLLIHAGEPGAGTRMKLARNMLTFVSYAAAAEAQRLAEACGLDLVALGKVVRHSDSFTGGAGAIMFRNTTAPMEPADPLRPLLEHTRGLGEKDLSLALALGEVVSVDLPLAQLALQRXRPPASGYRTRTPSQQRRHDGRAAPHRPGQNERGLRLGHARHAR

>363|CORE_REP|Org59_Gene3481#

MLISRMSVRSASMSVMGDVFIGSEAITAGRLTRHELQRWYQPMFRGVYVSRRSVPTLWDRTVGAWLATRRHGVIAGNAASALHGAQWVDVDVAIELISPTTRPQHGLVIRRETLCDDEITRVVXLPVTTLARTAYDLGRHLSRGEAVAXSSJAXXACHPVFXRRRASIGQASRSGARGVRRLRDVLPLVDGGAASPXETWLRLLLIDAGLPVPTTQIPVVHRWRNVGVLDMGWEKYMVAAEYDGDQHRXDRGRYVKDQRRLRKLAELGWIVIRVIAEDNPDDVVNRVRAALLARGWRP

>364|CORE_REP|Org45_Gene1752#

MSSGNSSLGIIVGIDDSPAAQVAVRWAARDAELRKIPLTLVHAVSPEVATWLEVPLPPGVLRWQQDHGRHLIDDALKVVEQASLRAGPPTVHSEIVPAAAVPTLVDMSKDAVLMVVGCLGSGRWPGRLLGSVSSGLLRHAHCPVVIIHDEDSVMPHPQQAPVLVGVDGSSASELATAIAFDEASRRNVDLVALHAWSDVDVSEWPGIDWPATQSMAEQVLAERLAGWQERYPNVAITRVVVRDQPARQLVQRSEEAQLVVVGSRGRGGYAGMLVGSVGETVAQLARTPVIVARESLT

>365|CORE_REP|Org118_Gene851#

MLFNAVHNSLPPNIDIDHAILRGEDHPPTCAKCVARGRISALGSLDLRYHSLRCYAAPPDVGRCEFVPPRRRVLIANQGLDVSRLPPTGTVTLLLADVEESTHLWQMCPEDMATAIAHLDHTVSEAITNHGGVQPVKRYEGDSFVAAFTRASDAAACALDLQRTSLAPIRLRIGLHTGEVQLRDELYVGPTINRTARLRDLAHGGQVVLSAATGDLVTGRLPADAWLVDLGRHPLRGLPRPEWVMAVVPPRHSRKVSPTAHGQIQPDIDSPGAVHHICGPPCANKLRCGHCWRRTGS

>366|CORE_REP|Org89_Gene2902#

MARNPAAQTAFGPMVLAAVEQNEPPGRRLVDDDLADLFLPRPLRWLAGATRSAVLRRLLISASEWSGRGLWANLACRKRFIGDKLDEALGDIDAVVILGAGLDTRAYRLTRRVRMPVFEVDLPVNIARKAKTVRRVLGELPLSVRLVALDFEHDDLLTALAEHGYRTEYRVFFVCEGVTQYLTERAVRRTLEGLRAAAPGSRMVFTYVRRDFIDGTNRYGTRTLYHTVRQRRQLWHFGLDPEEVAGFLADYGWRLTEQAGPEELVQRYVEPTGRNLNASQIEWSAYAEKSEPVTPR

>368|CORE_REP|Org124_Gene2503#

MWRPAQGARWHVPAVLGYGGIPRRASWSNVESVANSRRRPVHPGQEVELDFAREWVEFYDPDNPEHLIAADLTWLLSRWACVFGTPACQGTVAGRPNDGCCSHGAFLSDDDDRTRLADAVHKLTDDDWQFRAKGLRRKGYLELDEHDGQPQHRTRKHKGACIFLNRPGFAGGAGCALHSKALKLGVPPLTMKPDVCWQLPIRRSQEWVTRPDGTEILKTTLTEYDRRGWGSGGADLHWYCTGDPAAHVGTKQVWQSLADELTELLGEKAYGELAAMCKRRSQLGLIAVHPATRAAQ

>369|CORE_REP|Org73_Gene2215#

MNLVSEKEFLDLPLVSVAEIVRCRGPKVSVFPFDGTRRWFHLECNPQYDDYQQAALRQSIRILKMLFEHGIETVISPIFSDDLLDRGDRYIVQALEGMALLANDEEILSFYKEHEVHVLFYGDYKKRLPSTAQGAAVVKSFDDLTISTSSNTEHRLCFGVFGNDAAESVAQFSISWNETHGKPPTRREIIEGYYGEYVDKADMFIGFGRFSTFDFPLLSSGKTSLYFTVAPSYYMTETTLRRILYDHIYLRHFRPKPDYSAMSADQLNVLRNRYRAQPDRVFGVGCVHDGIWFAEG

>370|CORE_REP|Org59_Gene2146#

MSSPSRVSNTAVYAVLTIGAVITLSPFLLGLLTSFTSAHQFATGTPLQLPRPPTLANYADIADAGFRRAAVVTALMTAXILLGQLTFSVLAAYAFARLQFRGRDALFWVYVATLMVPGTVTVVPLYLMMAQLGLRNTFWALVLPFMFGSPYAIFLLREHFRLIPDDLINAARLDGANTLDVIVHVVIPSSRPVLAALAMITVVSQWNNFMWPLVITSGHKWRVLTVATADLQSRFNDQWTLVMAATTVAIVPLIALFVTFQRHIVASIVVXGLKXPXPASPRWXPPPLCWWRSCWV

>371|CORE_REP|Org119_Gene530#

MVESSTASATAVLRARYPRTAASLDRYGGGTARRLERTGTFARFTRISVVQIGWALRRYRRETLRLVAEIGMGTGAMAVVGGTVAIIGFVTLSGGSLIAIQGFASPRATSVSRRLPDSLPHWPNTRVAAPIVSGVALAATVGAGATAQLGAMRISEEIDALEVMGIKSISFLVSTRILGGLVVIMPLYALALDMAFTSGQVVTTVFYGQSNGTYEHYFRTFLRPEDVGWSVVEVVIIAVVVMITDCYYGYTASGGPVGVGQAVGRSMRFSLVSVVVVVLLAELALYGVDPNFNLTV

>372|CORE_REP|Org61_Gene1995#

MTWQIVFVVICVIVAGVAALFWRLPSDDTTRSRAKTVTIAAVAAAAVFFFLGCFTIVGTRQFAIMTTFGRPTGVSLNNGFHGKWPWQMTHPMDGAVQIDKYVKEGNTDQRITVRLGNQSTALADVSIRWQLKQAAAPELFQQYKTFDNVRVNLIERNLSVALNEVFAGFNPLDPRNLDVSPLPSLAKRAADILRQDVGGQVDIFDVNVPTIQYDQSTEDKINQLNQQRAQTSIALEAQRTAEAQAKANEILSRSISDDPNVVVQNCITAAINKGISPLGCWPGSSALPTIAVPGR

>373|CORE_REP|Org58_Gene2846#

MSRILTHVPGRTVNRSYALPALVGSAAGRLSGNHSHGREAYIALPQWACSRQPSTPPLQTPGRINALWSLRPVLPMPGRGCQLLRLGGRWLSVVCCRNGSMNLVVWAEGNGVARVIAYRWLRVGRLPVPARRVGRVILVDEPAGQPGRWGRTAVCARLSSADQKVDLDRQVVGVTAWATAEQIPVGKVVTEVGSALYGRRRTFLTLLGDPTVRRIVMKRRDRLGRFGFECVQAVLAADGRELVVVDSADVDDDVVGDITEILTSICARLYGKRAAGNRAARAVAAAARAGGHEAR

>374|CORE_REP|Org119_Gene587#

MAKLRPYYEESQSAYDISDDFFALFLDPTWVYTCAYFERDDMTLEEAQLAKVDLALDKLNLEPGMTLLDVGCGWGGALVRAVEKYDVNVIGLTLSRNHYERSKDRLAAIGTQRRAEARLQGWEEFEENVDRIVSFEAFDAFKKERYLTFFERSYDILPDDGRMLLHSLFTYDRRWLHEQGIALTMSDLRIPQIPAGVDLPGRRAAIGARHCRQCAGRGLHHRACPAAAAALRTDSRCMGRQPTGCPRTRHRRTVRRGLQQLHALSDRMRGALPQRPNQRRPVHHDQVARPLISVP

>375|CORE_REP|Org40_Gene3937#

MSELRLMAVHAHPDDESSKGAATLARYADEGHRVLVVTLTGGERGEILNPAMDLPDVHGRIAEIRRDEMTKAAEILGVEHTWLGFVDSGLPKGDLPPPLPDDCFARVPLEVSTEALVRVVREFRPHVMTTYDENGGYPHPDHIRCHQVSVAAYEAAGDFCRFPDAGEPWTVSKLYYVHGFLRERMQMLQDEFARHGQRGPFEQWLAYWDPDHDFLTSRVTTRVECSKYFSQRDDALRAHATQIDPNAEFFAAPLAWQERLWPTEEFELARSRIPARPPETELFAGIEPWTRFCSA

>376|CORE_REP|Org149_Gene1860#

MTETTDSPSERQPGPAEPELSSRDPDIAGQVFDAAPFDAAPDADSEGDSKAAKTDEPRPAKRSTLREFAVLAVIAVVLYYVMLTFVARPYLIPSESMEPTLHGCSTCVGDRIMVDKLSYRFGSPQPGDVIVFRGPPSWNVGYKSIRSHNVAVRWVQNALSFIGFVPPDENDLVKRVIAVGGQTVQCRSDTGLTVNGRPLKEPYLDPATMMADPSIYPCLGSEFGPVTVPPGRVWVMGDNRTHSADSRAHCPLLCTDDPLPGTVPVANVIGKARLIVWPPSRWGVVRSVNPQQGR

>378|CORE_REP|Org132_Gene771#

MTVPPAGPYGNYPYGPNTYGQDPYWGGQPQGGSYPPAYPPQQYPPGWPAGPYPPGPPPPGPGSKTPWLILAGLAVLGVILLVVILVIGLRGDNKSTTATSPATSAPTSQPFSQQTATGCTPNVSGGVQPIGDSISAGKLSFPTSAAPGWSAFSDDQNPNLIDAVGVGHEVAGADQWMMQAEVAITNFVTTMDVAAQASKLMQCVADGPGYAGSSPTLGPTKTSSITVDGVRAARVDADITIADSSRNVKGDSVTIIAVDTKPVTVFLGATPIGDATSRATVERVIEALKVNKS

>379|CORE_REP|Org118_Gene1714#

MSENRPEPVAAETSAATTARHSQADAGAHDAVRRGRHELPADHPRSKVGPLRRTRLTEILRGGRSRLVFGTLAILLCLVLGVAIVTQVRQTDSGDSLETARPADLLVLLDSLRQREATLNAEVIDLQNTLNALQASGNTDQAALESAQARLAALSILVGAVGATGPGVMITIDDPGPGVAPEVMIDVINELRAAGAEAIQINDAHRSVRVGVDTWVVGVPGSLTVDTKVLSPPYSILAIGDPPTLAAAMNIPGGAQDGVKRVGGRMVVQQADRVDVTALRQPKQHQYAQPVK

>380|CORE_REP|Org119_Gene384#

MPDGTIDGGHPQRPASPRLRSPLLRLATWNVNSIRTRLDRVLDWLGRADVDVLAMQETKCPDGQFPALPLFELGYDVAHVGFDQWNGVAIASRVGLDDVRVGFDGQPSWSGKPEVAATTEARALGATCGGIRVWSLYVPNGRALDDPHYTYKLDWLAALRDTAEGWLRDDPAAPIALMGDWNIAPTDDDDVWSTEFFAGCTHVSEPERKAFNAIVDAQFTDVVRPFTPGPGVYTYWDYTQLRFPKKQGMRIDFILGSPALAARVMDAQIVREERKGKAPSDHAPVLVDLHAG

>381|CORE_REP|Org83_Gene888#

MTEFASRRTLVVRRFLRNRAAVASLAALLLLFVSAYALPPLLPYSYDDLDFNALLQPPGTKHWLGTNALGQDLLAQTLRGMQKSMLIGVCVAVISTGIAATVGAISGYFGGWRDRTLMWVVDLLLVVPSFILIAIVTPRTKNSANIMFLVLLLAGFGWMISSRMVRGMTMSLREREFIRAARYMGVSSRRIIVGHVVPNVASILIIDAALNVAAAILAETGLSFLGFGIQPPDVSLGTLIADGTASATAFPWVFLFPASILVLILVCANLTGDGLRDALDPASRSLRRGVR

>382|CORE_REP|Org71_Gene4048#

MALVGPGAVGTTVAALLHKAGYSPLLCGHTPRAGIELRRDGADPIVVPGPVHTSPREVAGPVDVLILAVKATQNDAARPWLTRLCDERTVVAVLQNGVEQVEQVQPHCPSSAVVPAIVWCSAETQPQGWVRLRGEAALVVPTGPAAEQFAGLLRGAGATVDCDPDFTTAAWRKLLVNALAGFMVLSGRRSAMFRRDDVAALSRRYVAECLAVARAEGARLDDDVVDEVVRLVRSAPQDMGTSMLADRAAHRPLEWDLRNGVIVRKARAHGLATPISDVLVPLLAAASDGPG

>383|CORE_REP|Org66_Gene2320#

MTATEELTFESTSRFAEVDVDGPLKLHYHEAGVGNDQTVVLLHGGGPGAASWTNFSRNIAVLARHFHVLAVDQPGYGHSDKRAEHGQFNRYAAMALKGLFDQLGLGRVPLVGNSLGGGTAVRFALDYPARAGRLVLMGPGGLSINLFAPDPTEGVKRLSKFSVAPTRENLEAFLRVMVYDKNLITPELVDQRFALASTPESLTATRAMGKSFAGADFEAGMMWREVYRLRQPVLLIWGREDRVNPLDGALVALKTIPRAQLHVFGQCGHWVQVEKFDEFNKLTIEFLGGGR

>385|CORE_REP|Org72_Gene3626#

MRDAPRRRTALAYALLAPSLVGVVAFLLLPILVVVWLSLHRWDLLGPLRYVGLTNWRSVLTDSGFADSLVVTAVFVAIVVPAQTVLGLLAASLLARRLPGTGLFRTLYVLPWICAPLAIAVMWRWIVAPTDGAISTVLGHRIEWLTDPGLALPVVSAVVVWTNVGYVSLFFLAGLMAIPQDIHNAARTDGASAWQRFWRITLPMLRPTMFFVLVTGIISAAQVFDTVYALTGGGPQGSTDLVAHRIYAEAFGAAAIGRASVMAVVLFVILVGATVVQHLYFRRRISYELT

>386|CORE_REP|Org106_Gene1910#

MPELPEVEVVRRGLQAHVTGRTITEVRVHHPRAVRRHDAGPADLTARLRGARINGTDRRGKYLWLTLNTAGVHRPTDTALVVHLGMSGQMLLGAVPCAAHVRISALLDDGTVLSFADQRTFGGWLLADLVTVDGSVVPVPVAHLARDPLDPRFDCDAVVKVLRRKHSELKRQLLDQRVVSGIGNIYADEALWRAKVNGAHVAATLRCRRLGAVLHAAADVMREALAKGGTSFDSLYVNVNGESGYFERSLDAYGREGENCRRCGAVIRRERFMNRSSFYCPRCQPRPRK

>387|CORE_REP|Org28_Gene1920#

MSGPAIDASPALTFNQSSASIQQRRLSTGRQMWVLYRRFAAPSLLNGEVLTTVGAPIIFMVGFYIPFAIPWNQFVGGASSGVASNLGQYITPLVTLQAVSFAAIGSGFRAATDSLLGVNRRFQSMPMAPLTPLLARVWVAVDRCFTGLVISLVCGYVIGFRFHRGALYIVGFCLLVIAIGAVLSFAADLVGTVTRNPDAMLPLLSLPILIFGLLSIGLMPLKLFPHWIHPFVRNQPISQFVAALRALAGDTTKTASQVSWPVMAPTLTWLFAFVVILALSSTIVLARRP

>388|CORE_REP|Org118_Gene328#

MRGIILAGGSGTRLYPITMGISKQLLPVYDKPMIYYPLTTLMMAGIRDIQLITTPHDAPGFHRLLGDGAHLGVNISYATQDQPDGLAQAFVIGANHIGADSVALVLGDNIFYGPGLGTSLKRFQSISGGAIFAYWVANPSAYGVVEFGAEGMALSLEEKPVTPKSNYAVPGLYFYDNDVIEIARGLKKSARGEYEITEVNQVYLNQGRLAVEVLARGTAWLDTGTFDSLLDAADFVRTLERRQGLKVSMPRRSGVAHGLDRRRAAGVQRARALVKSGYGNYLLELLERN

>389|CORE_REP|Org119_Gene805#

MTEGRCAQHPDGLDVQDVCDPDDPRLDDFRDLNSIDRRPDLPTGKALVIAEGVLVVQRMLASRFTPLALFGTDRRLAELKDDLAGVGAPYYRASADVMARVIGFHLNRGVLAAARRVPEPSVAQVVAGARTVAVLEGVNDHENLGSIFRNAAGLSVDAVVFGTGCADPLYRRAVRVSMGHALLVPYCARAADWPTELMTLKESGFRLLAMTPHGNACKLPEAIAAVSHERIALLVGAEGPGLTAAALRISDVRVRIPMSRGTDSLNVATAAALAFYERTRSGHHIGPGT

>390|CORE_REP|Org91_Gene3276#

MFPRWPQQAHNHEVSRADTVSVPRAPTQAEVAAVLRIMTPLRKVIKPKVYGIENVPTERALLVGNHNTLGLVDAPLLAAELWERGRIVRSLGDHAHFKIPGWRDALTRTGVVEGTREITSELMRRGELVMVFPGGAREVNKRKNERYKLVWKNRLGFARLAIQHGYPIVPFASVGAEHGIDIVLDNESPLLAPVQFLAEKLLGTKDGPALVRGVGLTPVPRPERQYYWFGEPIDTTEFMGQQADDNAARRVRERAAAAIEHGIELMLAERAADPNRSLVGRLLRSDA

>391|CORE_REP|Org13_Gene1951#

MSDEDRTDRATEDHTIFDRGVGQRDQLQRLWTPYRMNYLAEAPVKRDPNSSASPAQPFTEIPQLSDEEGLVVARGKLVYAVLNLYPYNPGHLMVVPYRRVSELEDLTDLESAELMAFTQKAIRVIKNVSRPHGFNVGLNLGTSAGGSLAEHLHVHVVPRWGGDANFITIIGGLQGDSAAAARHPSAACHRVGSATMSKLPFLSRAAFARITTPIARGLLRVGLTPDVVTILGTTASVAGALTLFPMGKLFAGACVVWFFVLFDMLDGAMARERGGRHSLRRGAGRHL

>392|CORE_REP|Org59_Gene3031#

MKRYLTIIYGAASYLVFLVAFGYAIGFVGDVVVPRTVDHAIAAPIGQAVVVNLVLLGVFAVQHSVMARQGFKRWWTRFVPPSIERSTYVLLASVALLLLYWQWRTMPAVIWDVRQPAGRXALWALFWLGXXTVLTSTFMINHFELFGLRQXXXALARKAVHRDRFSGSSALPVGTPPDHARIRRRVLGDAHDDGGALAFRDRRDGLHLGRVAVRRARPTRGAGRPIPRLPPRGVDVVAVAAPAYLSQRDCPYRFYLGRISSRIDSYGRQGGAAAGGGPAIRVHQLSR

>394|CORE_REP|Org81_Gene2587#

MQSGPHLVGRVGTSFPLIARHQGATRDDAGDTGQPDPLPHVAHPDRLYPPMVHGVDPSTLALDRALNETRTGDLWLFRGRSRPDRAIQTLTNAPVNHVGMTVAIDDLPPLIWHAELGDKLLDVWTGTNHRGVQLNDARQVVQQWAGRYRQRCWLRQLTPHANRDQEDKLLRVIARMNGTPFPTTARLTGRWLRGRLPTLNDWLRGIPVLDRKVREQTQRRKQQQRTMGLATAYCAETVAITYEEMGLLVTDKDAHWFDPGKFWSGDSLPLAPGYRLGHEIAVDVGG

>395|CORE_REP|Org47_Gene1968#

MCVTWAEMPKIAALIRHIEDLHARHGRSYILRAGISSLFRYIEGVHGERPWGTVLDAGTGVKSLQWIQTLPTERWTAVTAARSLADKTRAALGSAMRPQDRLLVGNWVDDSLLAGETFDTILVDYLVGAIEGFAPYWQDRVFERLRPHLADHGRLYLVGLEPYVQFEPETESGKIIWEIGRVRDACLLLAGERPYREFPLDWMLGRLGLAGFRILEARRFPIRYRARYVNGQLNMCLARIERFSSNGLGMAMRAYVEELRARALQLNERQDGLWHGNDYVIAVEPM

>396|CORE_REP|Org59_Gene884#

MTRQKILITGASSGLGAGMARSFAAQGRDLALCARRTDRLTELKAELSQRYPDIKIAVAELDVNDHERVPKVFAELSDEIGGIDRVIVNAGIGKGARLGSGKLWANKATIETNLVAALVQIETALDMFNQRGSGHLVLISSVLGVKGVPGVKAAYAASKAGVRSLGESLRAEYAQRPIRVTVLEPGYIESEMTAKSASTMLMVDNATGVKALVAAIERLDARTRRGPLVAMGATGAADVGAAAAADQTLRLAGARPPSPRGHVRCGSGAPARRSSCPTAVAVNRRR

>397|CORE_REP|Org105_Gene2694#

MGLSDWELAAARAAIARGLDEDLRYGPDVTTLATVPASATTTASLVTREAGVVAGLDVALLTLNEVLGTNGYRVLDRVEDGARVPPGEALMTLEAQTRGLLTAERTMLNLVGHLSGIATATAAWVDAVRGTKAKIRDTRKTLPGLRALQKYAVRTGGGVNHRLGLGDAALIKDNHVAAAGSVVDALRAVRNAAPDLPCEVEVDSLEQLDAVLPEKPELILLDNFAVWQTQTAVQRRDSRAPTVMLESSGGLSLQTAATYAETGVDYLAVGALTHSVRVLDIGLDM

>398|CORE_REP|Org77_Gene1327#

MVKRSRATRLSPSIWSGWESPQCRSIRARLLLPRGRSRPPNADCCWNQLAVTPDTRMPASSAAGRDAAAYDAWYDSPTGRPILATEVAALRPLIEVFAQPRLEIGVGTGRFADLLGVRFGLDPSRDALMFARRRGVLVANAVGEAVPFVSRHFGAVLMAFTLCFVTDPAAIFRETRRLLADGGGLVIGFLPRGTPWADLYALRAARGQPGYRDARFYTAAELEQLLADSGFRVIARRCTLHQPPGLARYDIEAAHDGIQAGAGFVAISAVDQAHEPKDDHPLESE

>399|CORE_REP|Org145_Gene2164#

MTNCAAGKPSSGPNLGRFGSFGRGVTPQQATEIEALGYGAVWVGGSPPAALSWVEPILQATTTLCVATGIVNIWSAPAQRVAESFHRIEAAYPGRFLLGIGVGHAEMISEYRKPYNALVEYLDRLDDYGVPANRRVVAALGPRVLGLSARRSAGAHPYLTTPEHTARARELIGPSAFLAPEHKVVLTTDSARARTVGRQALDMYFNLANYRNNWKRLGFTDDEVSRPGSDRLVDAVVAYGTPDAIAARLNEHLLAGADHVPIQVLTEDDNLVSALTELAKPLRLT

>400|CORE_REP|Org120_Gene2737#

MQARGQVLITAAELAGMIQAGDPVSILDVRWRLDEPDGHAAYLQGHLPGAVFVSLEDELSDHTIAGRGRHPLPSGASLQATVRRCGIRHDVPVVVYDDWNRAGSARAWWVLTAAGIANVRILDGGLPAWRSAGGSIETGQVSPQLGNVTVLHDDLYAGQRLTLTAQQAGAGGVTLLDARVPERFRGDVEPVDAVAGHIPGAINVPSGSVLADDGTFLGNGALNALLSDHGIDHGGRVGVYCGSGVSAAVIVAALAVIGQDAELFPGSWSEWSSDPTRPVGRGTA

>401|CORE_REP|Org20_Gene2760#

MSFLTTQPEELAAAAGKLETIGSAMVAQNAAAAAPTTTGVIPAAADEISVLQAPLFTAYGTLYQQVSAEAAAVYDLFVKTLGVSAGTYAATEAANSSAAASPLSGIASILGSTPGKVPSWISDIANIFNIGAGNWASAASDLLGLASGGLLPAAEEAALEEGLEGAGLSELGAAEAAVGEAPIAAGLGAAPLAAGLSRASSIGALSVPPSWAGQANLVSSTSTLQGAGWTTAAPHGAAGRALRQAGVGLAAQQRGLRCATLRRQTHRHAQAGGIGGGLDAVART

>402|CORE_REP|Org1_Gene555#

MTTIGTRKRVAVVTGASSGIGEATARTLAAQGFHVVAVARRADRITALANQIGGTAIVADVTDDAAVEALARALSRVDVLVNNAGGAKGLQFVADADLEHWRWMWDTNVLGTLRVTRALLPKLIDSGDGLIVTVTSIAAIEVYDGGAGYTAAKHAQGALHRTLRGELLGKPVRLTEIAPGAVETEFSLVRFDGDQQRADAVYAGMTPLVAADVAEVIGFVATRPSHVNLDQIVIRPATRHQLAVALPTRSVSSRGCAGRGGGIRRRDRGGDRDLRRRRFGRRWR

>403|CORE_REP|Org25_Gene11#

MTGPSAAGRAGTADNVVGVEVTIDGMLVIADRLHLVDFPVTLGIRPNIPQEDLRDIVWEQVQRDLTAQGVLDLHGEPQPTVAEMVETLGRPDRTLEGRWWRRDIGGVMVRFVVCRRGDRHVIAARDGDMLVLQLVAPQVGLAGMVTAVLGPAEPANVEPLTGVATELAECTTASQLTQYGIAPASARVYAEIVGNPTGWVEIVASQRHPGGTTTQTDAAAGVLDSKLGRLVSLPRRVGGDLYGSFLPGTQQNLERALDGLLELLPAGAWLDHTSDHAQASSRG

>404|CORE_REP|Org144_Gene3035#

MLAQATTAGSFNHHASTVLQGCRGVPAAMWSEPAGAIRRHCATIDGMDCEVAREALSARLDGERAPVPSARVDEHLGECSACRAWFTQVASQAGDLRRLAESRPVVPPVGRLGIRRAPRRQHSPMTWRRWALLCVGIAQIALGTVQGFGLDVGLTHQHPTGAGTHLLNESTSWSIALGVIMVGAALWPSAAAGLAGVLTAFVAILTGYVIVDALSGAVSTTRILTHLPVVIGAVLAIMVWRSASGPRPRPDAVAAEPDIVLPDNASRGRRRGHLWPTDGSAA

>405|CORE_REP|Org119_Gene3336#

MSPAPVQVMGVLNVTDDSFSDGGCYLDLDDAVKHGLAMAAAGAGIVDAGGGESSRPGATRVDPAVETSRVIPVVKELAAQGITVSIDTMRADVARAALQNGAQMVNDVSGGRADPAMGPLLAEADVPWVLMHWRAVSADTPHVPVRYGNVVAEVRADLLASVADAVAAGVDPARLVLDPGLGFAKTAQHNWAILHALPELVATGIPVLVGASRKRFLGALLAGPDGVMRPTDGRDTATAVISALAALHGAWGVRVHDVRASVDAIKVVEAWMGAERIERDG

>407|CORE_REP|Org118_Gene3620#

MPPLTSLAPTTAERIRSACARAGGALLVVEREDPVPVPIHHLLYDGSFAVAVPVDRGEVSGSQALLELTDYAPLPVREPVRSLVWIRGCLHQIPPAELVETLDLIATDNPNPALLQVETPRPGPADAAETRYTMQRLEIESVVVTDATGAEPVTVADLLAARPDPFCEIESTLLWHLATAHDDVVARLVSRLPAPLRRGQIRPLGLDRYGVRFRIEARDGDRDIRLPFHKPVDDMTGLSQAIRVLMGCPFPQRAARPQVAGTAAARPRWPAASKGSATYVV

>408|CORE_REP|Org19_Gene1144#

MGWADRIVHRHFIRGLALYAGLIGIAWCALFPIIWALSGSLKADGEVTEPTLFPSHPQWSNYREVFALMPFWRMFFNTVLYAGCVTAGQVFFCSLAGYAFARLQFRGRDTLFVLYLSTLMVPLTVTVIPQFILMRIVGWVDTPWAMIVPGLFGSAFGTYLMRQFFRTLPTDLEEAAILDGCSPWQIYWRILLPHSRPAVLVLGVLTWVNVWNDFLWPLLMIQRNSLATLTLGLVRLRGEYVARWPVLMAASMLMLVPLVILYAVAQRSFVRGIAVTGLGG

>409|CORE_REP|Org14_Gene1333#

MLDRYGTDVLAAGGRRRPRSVEHPVELGMVVEDAETGYVGAVVRVEYGRIDLEDRYGKTRGFPLGPGYLLDGLPVILTAPRCAAAAGPRRTASGSVAVPGARARVARASRIYVEGRHDAELIAAVWGADLRIEGVVVEHLGGVDDLVEIVAKFRPGPRRRLGVLVDHLVAGSKEARIAEVVRRGPGGSDTLVVGHPYVDIWQAVKPQRVGLAAWPRVPRHIEWKHGVCDALGWPHADQADIAAAWRRIRSQVRDWTDLEPALIGRVEELIDFVTQPAGDE

>410|CORE_REP|Org133_Gene2124#

MSPRVPRLRWDDPFRALDMLASLWSSTGMSLVSAGAAQAVAAPYRTLFTTLQQLLIGKEVTVRIGDHDVVLTVTELDSALEPQGLAVGQLGEVRVAARGISWDQHHLHSAVAVLRNVHIRPGVPPLVIAAPVELSSALPTEIFDDVLRQATPQLRGELSESGAARLRWARRPDWGGLEVDVDVAGTTSQTTLWLRPRTVITGQRRWTLPARTPAYRVPLPELPHGLRITDVSLAADCLQLSALLPEWRTELPLRYLESVITQLSQGALSFVWPPLRSGAD

>411|CORE_REP|Org57_Gene348#

MRIALAQIRSGTDPAANLQLVGKYAGEAATAGAQLVVFPEATMCRLGVPLRQVAEPVDGPWANGVRRIATEAGITVIAGMFTPTGDGRVTNTLIAAGPGTPNQPDAHYHKIHLYDAFGFTESRTVAPGREPVVVVVDGVRVGLTVCYDIRFPALYTELARRGAQLIAVCASWGSGPGKLEQWTLLARARALDSMSYVAAAGQADPGDARTGVGASSAAPTGVGGSLVASPLGEVVVSAGTQPQLLVADIDVDNVAAARDRIAVLRNQTDFVQIDKAQSRG

>412|CORE_REP|Org45_Gene3983#

MLEITLLGTGSPIPDPDRAGPSTLVRAGAQAFLVDCGRGVLQRAAAVGVGAAGLSAVLLTHLHSDHIAELSDVLITSWVTNFAADPAPLPIIGPPGTAEVVEATLKAFGHDIGYRIAHHADLTTPPPIEVHEYTAGPAWDRDGVTIRVAPTDHRPVTPTIGFRIESDGASVVLAGDTVPCDSLDQLAAGADALVHTVIRKDIVTQIPQQRVKDICDYHSSVQEAAATANRAGVGTLVMTHYVPAIGPGQEEQWRALAATEFSGRIEVGNDLHRVEVHPRR

>414|CORE_REP|Org20_Gene421#

MHESRLASARLYLCTDARRERGDLAQFAEAALAGGVDIIQLRDKGSPGELRFGPLQARDELAACEILADAAHRYGALFAVNDRADIARAAGADVLHLGQRDLPVNVARQILAPDTLIGRSTHDPDQVAAAAAGDADYFCVGPCWPTLPSGRAAPLGLVRVAAELGGDDKPWFAIGGINAQRLPAVLDAGASASWWCGRSPRLTTHVRRPSSSGRRLQQRTDPPTQRRGRLIRVARRTPEENPGLDLNGARQPLPPFVAPGQWHGRRSGPQSLPTGGESGM

>415|CORE_REP|Org119_Gene237#

MSAPTANRPAIGVFTPTRAQIPERTLRTDLWWLPPLLTNLGLLAFICYATTRAFWGSQYWVEKYHYLTPFYSPCVSASCQPGASHLGVWFGHFPGWIPLGAMVLPFLLGFRLTCYYYRKAYYRSVWQSPTSCAVPEPRAHYTGETRLPLIVQNTHRYFFYIAVVVSLINTYDAIAAFHSPSGFGFGLGNVILTINVVLLWAYTISCHSCRHATGGRLKHFSKHPVRYWIWTQVSKLNTRQHAIRVDHAGDPGAHRFLHHAGGQWQHHRSQIYWLKDRFRS

>416|CORE_REP|Org32_Gene1973#

MTSSNDSHWQRPDDSPGPMPGRPVSASLVDPEDDLTPARYAGDFGSGTTTVIPPYDAASSGVGNSGYSLIEAAEPLPYVQPQPGRQVPAGSAGIDMDDDERVRAAGRRGTQNLGLLILRVGLGAVLIAHGLQKLFGWWDGQGLAGFQNSLSDIGYQHAEILAYVSAGGEIVAGVLLVLGLFTPLAAAGALAFLINGLLAGISAQHSRPVAYFLQDGHEYQITLVVMAVAVILSGPGRYGLDAARGWAHRPFIGSFVALLGGIAAGIAVWVLLNGANPLA

>418|CORE_REP|Org2_Gene1462#

MGVERVGARRATYWAVLDTLVVGYALLPVLWIFSLSLKPTSTVKDGKLIPSTVTFDNYRGIFRGDLFSSALINSIGIGLITTVIAVVLGAMAAYAVARLEFPGKRLLIGAALLITMFPSISLVTPLFNIERAIGLFDTWPGLILPYITFALPLAIYTLSAFFREIPWDLEKAAKMDGATPGQAFRKVIVPLAAPGLVTAAILVFIFAWNDLLLALSLTATKAAITAPVAIANFTGSSQFEEPTGSIAAGAIVITIPIIVFVLIFQRRIVAGLTSGAVKG

>419|CORE_REP|Org139_Gene3998#

MNGLQNSLANGGTAPENGYSAGFRVRLTNFEGPFDLLLQLIFAHQLDVTEVALHQVTDDFIAYTKAIGARLELEETTAFLVIAATLLDLKAARLLPAGQVDDEEDLALLEVRDLLFARLLQYRAFKHVAEMFAELEATALRSYPRAVSLEDGFVGLLPEVMLGVDAHRFAEIAAIALTPRPAPTVATEHLHELMVSVPEQAEHLLAMLKARGSGQSASFSELVADCTAPIEIVGRFLALLELYRTRAVAFEQSEPLGALQVSWTGDDAERSDEKERRL

>420|CORE_REP|Org13_Gene801#

MRETSNPVFRSLPKQRGGYAQFGTGTAQQGFPADPYLAPYREAKATRPLTIDDVVTKTGLTLAMLAGTAVVSYFLVASNVALAMPLTLVGALGGLALVLVATFGRKQDNPAIVLSYAALEGLFLGAISFVLANFTVASANAGVLIGEAILGTMGVFFGMLVVYKTGAIRVTPKFTRMVVAALFGVLVLMLGNLVLAMFNVGGGEGLGLRSPGPLGIIFSLVCIGIAAFSFLIDFDAADQMIRAGAPEKAAWGVALGLTVTLVWLYIEILRLLSYLQNE

>421|CORE_REP|Org20_Gene1553#

MAARSVVLDMVEDVHRHRRATAGVGAKVSRASARRRRAVSDEDKSQRRDEILAAAKIVFAHKGFHATTVADIAKQAGLAYGLIYWYFDSKDDLFHALMAGEEEALRAHVAAELARVGGSTEAPLRALLQAAVQATFEFFETDKATVKLLFRDAYALGGRFEEHLGGIYERFIDDIEAVVVAAQRRGEVVEAPSRMAAYTLAALVGQLAHRRLNTDDNVTAAQVADFVVSLVLDGLQSACTGGRGPRWSGRPNLSKGCQIHGERVRIRDTRPDHVDRDG

>422|CORE_REP|Org56_Gene1256#

MKAIFITGAGSGMGREGATLFHANGWRVGAIDRNEDGLAALRVQLGAERLWARAVDVTDKAALEGALADFCAGNVGGGLDMMWNNAGIGEGGWFEDVPYEAAVRVVDVNFKAVLTGAYAALPYLKKAPGSLMFSTSSSSGTYGMPRIAVYSATKHAVKGLTEALSVEWQRHGVRVADVLPGLIDTAILTSTRQHSDEGPYTISAEQIRAAAPKKGMFRLMPSSSVAEAAWRAYQHPTRLHWYVPRSIRWIDRLKGVSPEFVRRHIAKSLATLEPKRK

>423|CORE_REP|Org2_Gene2985#

MDQSPDTSRRLTDEQKIQLIDSMRNKGSYEAARERLTATARIIADRVSAAIPGQTWKFTEDPAGRKADREGLSCKELTGDIARRPIADAVIFGTAFSAEDFKVVTNIVREEAAKYGATTESSLFNESAKRDYDVQGNGYEFNLGQIKFATLNITGDCFLLQKVLDLPAGQLPPNHPSGRRPRRQPREHHHRCWRDPGSPAGDPAHAQAGSRSRAHDGVFRAQTLQCIYLSIEYLYVCSMSRRTTIDIDDILLARAQAALGTTGLKDRVDAALRAAVR

>424|CORE_REP|Org20_Gene726#

MGQKINPHGFRLGITTDWKSRWYADKQYAEYVKEDVAIRRLLSSGLERAGIADVEIERTRDRVRVDIHTARPGIVIGRRGTEADRIRADLEKLTGKQVQLNILEVKNPESQAQLVAQGVAEQLSNRVAFRRAMRKAIQSAMRQPNVKGIRVQCSGRLGGAEMSRSEFYREGRVPLHTLRADIDYGLYEAKTTFGRIGVKVWIYKGDIVGGKRELAAAAPAGADRPRRERPSGTRPRRAVLRAPRRPVPTRVGPRVAKRPRLTPQRPLKRRARRAESC

>425|CORE_REP|Org20_Gene430#

MNYLPLAPPGMTPPRVLSIAGSDSGGGAGIQADMRTMALLGVHACVAVTAVTVQNTLGVKDIHEVPNDVVAGQIEAVVTDIGVQAAKTGMLASSRIVATVAATWRRLELSVPLVVDPVCASMHGDPLLAPSALDSLRGQLFPLATLLTPNLDEARLLVDIEVVDAESQRAAAKALHALGPQWVLVKGGHLRSSDGSCDLLYDGVSCYQFDAQRLPTGDDHGGGDTLATAIAARWRTASPCPTRWTSGSDGLPNACARPIHWAAATGPFRRCFGCHEP

>426|CORE_REP|Org127_Gene1903#

MITTTSQEIELAPTRLPGSQNAARLFVAQTLLQTNRLLTRWARDYITVIGAIVLPILFMVVLNIVLGNLAYVVTHDSGLYSIVPLIALGAAITGSTFVAIDLMRERSFGLLARLWVLPVHRASGLISRILANAIRTLVTTLVMLGTGVVLGFRFRQGLIPSLMWISVPVILGIAIAAMVTTVALYTAQTVVVEGVELVQAIAIFFSTGLVPLNSYPGWIQPFVAHQPVSYAIAAMRGFAMGGPVLSPMIGMLVWTAGICVVCAVPLAIGYRRASTH

>427|CORE_REP|Org119_Gene1497#

MSPATVLDSILEGVRADVAAREASVSLSEIKAAAAAAPPPLDVMAALREPGIGVIAEVKRASPSAGALATIADPAKLAQAYQDGGARIVSVVTEQRRFQGSLDDLDAVRASVSIPVLRKDFVVQPYQIHEARAHGADMLLLIVAALEQSVLVSMLDRTESLGMTALVEVHTEQEADRALKAGAKVIGVNARDLMTLDVDRDCFARIAPGLPSSVIRIAESGVRGTADLLAYAGAGADAVLVGEGLVTSGDPRAAVADLVTAGTHPSCPKPARASRR

>428|CORE_REP|Org145_Gene1767#

MTPDRLRSSVGYAAMLLVVTLIAGPLLFVFFTSFKDQPDIYAQPTSWWPLRWYPQNYRTATEQIPFWTFLRNSLIITSVLAVVKFTLGVLSAFGLVFVRFPGRTAVFLVIIAALMVPNQITVISNYALISHLGLRNTFAGIILPLAGVAFGTFLMRNHFLSLPAEIIEAARMDGARWWQLLLRVVLPMSRPTMVAVGVITVVNEWNEYLWPFLMSDDESVAPLPIGLTFLQQAEGVTNWGPVMAVTLLAMLPILLVFIALQRQMIKGLTSGAVKG

>429|CORE_REP|Org37_Gene2584#

MDGFPGRGAVITGGASGIGLATGTEFARRGARVVLGDVDKPGLRQAVNHLRAEGFDVHSVMCDVRHREEVTHLADEAFRLLGHVDVVFSNAGIVVGGPIVEMTHDDWRWVIDVDLWGSIHTVEAFLPRLLEQGTGGHVVFTASFAGLVPNAGLGAYGVAKYGVVGLAETLAREVTADGIGVSVLCPMVVETNLVANSERIRGAACAQSSTTGSPGPLPLQDDNLGVDDIAQLTADAILANRLYVLPHAASRASIRRRFERIDRTFDEQAAEGWRH

>430|CORE_REP|Org11_Gene710#

MAQKPVADALTLELEPVVEANMTRHLDTEDIWFAHDYVPFDQGENFAFLGGRDWDPSQSTLPRTITDACEILLILKDNLAGHHRELVEHFILEDWWGRWLGRWTAEEHLHAIALREYLVVTREVDPVANEDVRVQHVMKGYRAEKYTQVETLVYMAFYERCGAVFCRNLAAQIEEPILAGLIDRIARDEVRHEEFFANLVTHCLDYTRDETIAAIAARAADLDVLGADIEAYRDKLQNVADAGIFGKPQLRQLISDRITAWGLAGEPSLKQFVTG

>431|CORE_REP|Org61_Gene1948#

MCAFVPHVPRHSRGDNPPSASTASPAVLTLTGERTIPDLDIENYWFRRHQVVYQRLAPRCTARDVLEAGCGEGYGADLIACVARQVIAVDYDETAVAHVRSRYPRVEVMQANLAELPLPDASVDVVVNFQVIEHLWDQARFVRECARVLRGSGLLMVSTPNRITFSPGRDTPINPFHTRELNADELTSLLIDAGFVDVAMCGLFHGPRLRDMDARHGGSIIDAQIMRAVAGAPWPPELAADVAAVTTADFEMVAAGHDRDIDDSLDLIAIAVRP

>432|CORE_REP|Org20_Gene2745#

MPWTTDADGGPALVEFAGRACYQSWSKPNPKTATNAGYLRHIIDVGHFSVLEHASVSFYITGISRSCTHELIRHRHFSYSQLSQRYVPEKDSRVVVPPGMEDDADLRHILTEAADDRRAIYSELLSRWKPSSPTNPTRSCAASRPAKPSRAVLPNATETRIVVTGNYRAWRHFIAMRASEHADVEIRRLAIECLRQLAAVAPAVFADFEVTTLADGTEVATSPLAPKPEAACRWTNTRARGRDKAPGNLGSRDHRRIRRRSAPRNPADRDGDTV

>433|CORE_REP|Org59_Gene1728#

MSHLSVMHRFRIYVDIAVVVLVLVLTNLIAHFTTPWASIATVPAAAVGLVILVRSRGLGWAELGLSRQHWKSGLVYALAAVALVVAXXSVGVLLPITRPMFMNHHYATISGAVIASMVMIPLQTVIPEELAFRGVLHGALNRAWGFRGVAVAGSVLFGLWHIATSLGLTSSNVGFTRLFGGGIIGLVAGVMLAVLATGVAGFVFSWLLXAQRQPDRTDRTALVAERDGCPGRCPGLAPVDLSSVRSAARRHRRCGRRSGRPAHDRLGQAGTPAW

>434|CORE_REP|Org141_Gene2460#

MKPTVPALVACDVDGTLLDDGETVTKRTRDAVHAAVDAGTHFILATGRPPRWVRPIVDALGFAPMAVCANGAVIYDPGTDRVMSVRTLPVDALATLAEVATRVIPGAGLAVERIGERAHDTATPQFVSSPGYEHAWLNPDNTEVSIDHLLSAPAIKLLIRKAGAASADMAAELAKHVGFEGDITYSTNNGLVEIVPLGISKATGVDEIARPLGISDAEVVAFGDMPNDVPMLLRAGLGVAMGNAHPDALAVADEVTAPNSEDGVARVLERWWS

>435|CORE_REP|Org92_Gene2680#

MIPVKVENNTSLDQVQDALNCVGYAVVEDVLDEASLAATRDRMYRVQERILTEIGKERLARAGELGVLRLMMKYDPHFFTFLEIPEVLSIVDRVLSETAILHLQNGFILPSFPPFSTPDVFQNAFHQDFPRVLSGYIASVNIMFAIDPFTRDTGATLVVPGSHQRIEKPDHTYLARNAVPVQCAAGSLFVFDSTLWHAAGRNTSGKDRLAINHQFTRSFFKQQIDYVRALGDAVVLEQPARTQQLLGWYSRVVTNLDEYYQPPDKRLYRKGQG

>436|CORE_REP|Org34_Gene841#

MRRCIPHRCIGHGTVVSVRITVLGCSGSVVGPDSPASGYLLRAPHTPPLVIDFGGGVLGALQRHADPASVHVLLSHLHADHCLDLPGLFVWRRYHPSRPSGKALLYGPSDTWSRLGAASSPYGGEIDDCSDIFDVHHWADSEPVTLGALTIVPRLVAHPTESFGLRITDPSGASLAYSGDTGICDQLVELARGVDVFLCEASWTHSPKHPPDLHLSGTEAGMVAAQAGVRELLLTHIPPWTSREDVISEAKAEFDGPVHAVVCDETFEVRRAG

>437|CORE_REP|Org32_Gene1810#

MIDRPLEGKVAFITGAARGLGRAHAVRLAADGANIIAVDICEQIASVPYPLSTADDLAATVELVEDAGGGIVARQGDVRDRASLSVALQAGLDEFGRLDIVVANAGIAMMQAGDDGWRDVIDVNLTGVFHTVQVAIPTLIEQGTGGSIVLISSAAGLVGIGSSDPGSLGYAAAKHGVVGLMRAYANHLAPQNIRVNSVHPCGVDTPMINNEFFQQWLTTADMDAPHNLGNALPVELVQPTDIANAVAWLASEEARYVTGVTLPVDAGFVNKR

>438|CORE_REP|Org28_Gene1579#

MTSLPAARYLVRSVALGYVFVLLIVPVALILWRTFEPGFGQFYAWISTPAAISALNLSLLVVAIVVPLNVIFGVTTALVLARNRFRGKGVLQAIIDLPFAVSPVIVGVSLILLWGSAGALGFVEQDLGFKIIFGLPGIVLASMFVTCPFVVREVEPVLHELGTDQEQAAATLGSGWWQTFWRITLPSIRWGLTYGIVLTVARTLGEYGAVIIVSSNLPGTSQTLTLLVSDRYHRGAEYGAYALSTLLMAVSVVVLIVQMVLDARRARAVSEG

>439|CORE_REP|Org75_Gene2351#

MLLAIDVRNTHTVVGLLSGMKEHAKVVQQWRIRTESEVTADELALTIDGLIGEDSERLTGTAALSTVPSVLHEVRIMLDQYWPSVPHVLIEPGVRTGIPLLVDNPKEVGADRIVNCLAAYDRFRKAAIVVDFGSSICVDVVSAKGEFLGGAIAPGVQVSSDAAAARSAALRRVELARPRSVVGKNTVECMQAGAVFGFAGLVDGLVGRIREDVSGFSVDHDVAIVATGHTAPLLLPELHTVDHYDQHLTLQGLRLVFERNLEVQRGRLKTAR

>440|CORE_REP|Org55_Gene992#

MRAVFGCAIAVVGIAGSVVAGPADIHLVAAKQSYGFAVASVLPTRGQVVGVAHPVVVTFSAPITNPANRHAAERAVEVKSTPAMTGKFEWLDNDVVQWVPDRFWPAHSTVELSVGSLSSDFKTGPAVVGVASISQHTFTVSIDGVEEGPPPPLPAPHHRVHFGEDGVMPASMGRPEYPTPVGSYTVLSKERSVIMDSSSVGIPVDDPDGYRLSVDYAVRITSRGLYVHSAPWALPALGLENVSHGCISLSREDAEWYYNAVDIGDPVIVQE

>441|CORE_REP|Org59_Gene1492#

MHLDSLVAPLVEQASXIXXXLQRLJFLVGHRADSAVRKKGNDFATEVDLAIERQVVAALVAATGIEVXGEEFXGPAVDSXWVWXXDPIDGTINYAAGSPLAAILLGLLHDGVPVAGLTWMPFTDQRYTAVAGGPLIKNGXPXPPLADAELANVLVGVGTFSADSRGQFPGRYRLAVLEKLSRVSSRLRMHGSTGIDLVFVADGILGGAISFGGHVWDHAAGVALVRAAGGVVTDLAGQPWTPASRSALAGPPRVHAQILEILGSIGEPEDY

>442|CORE_REP|Org59_Gene2732#

MSMLARHGPRYGGSXNGHSDXSXGXAKXAAPTLYIFPHAGGTAKDYVAFSREFSADVKRIAVQYPGQHDRSGLPPLESIPTLADEIFAMMKPSARIDDPVAFFGHSMGGMLAFEVALRYQSAGHRVLAFFVSXXSAPGHIRYKQLQDLSDREMLDLFTRMTGMNPDFFTDXEFFVGALPTLRAVRAIAGYSCPPETKLSCPIYAFIGDKDWIATQDDMDPWRDRTTEEFXIRVFPGDHFYLNDNLPELVSDIEDKTLQWLXSXPXLCSGCS

>443|CORE_REP|Org5_Gene583#

MPMRKVLVGVTGAAIVVAVLIVGAVGADFGASIYAEYRLSTTVRKAANLRSDPFVAILRFPFIPQAMREHYAELEIKAFAVEHAGSGTATLEATMHSIDLSYASWLIRPDAKLPVGELESRIIIDSMHLGRYLGISDLMVAAPRQESNDATGGTTESGISGSRGLVFSGTPISANFAHRVSVLVDLSVASDDRATLVITPTAVVTGPDTADQPVPDDKRDAVLHAFASKLPNQKLPFGVVPNTVGARGSDVIIEGITRGVTISLDEFKQS

>444|CORE_REP|Org136_Gene1795#

MANPFVKAWKYLMALFSSKIDEHADPKVQIQQAIEEAQRTHQALTQQAAQVIGNQRQLEMRLNRQLADIEKLQVNVRQALTLADQATAAGDAAKATEYNNAAEAFAAQLVTAEQSVEDLKTLHDQALSAAAQAKKAVERNAMVLQQKIAERTKLLSQLEQAKMQEQVSASLRSMSELAAPGNTPSLDEVRDKIERRYANAIGSAELAESSVQGRMLEVEQAGIQMAGHSRLEQIRASMRGEALPAGGTTATPRPATETSGGAIAEQPYGQ

>445|CORE_REP|Org91_Gene3520#

MTSTTLPHRASLVDRSTEFCHTDVVKIPAVSTTVPAAVSDGHTRRAIVRLLLESGSITAGEIGDRLGLSAAGVRRHLDALIEAGDAEASAAAPWQQVGRGRPAKRYRLTAAGRAKLDHSYDDLASAAMRQLREIGGEEAVRTFARRRIDAILADVAPADGPDDAALEAAAERIATALSKAGYVATTTRVGGPIHGVQICQHHCPVSHVAEEFPELCETEQQAMAEVLGTHVQRLATIVNGDCACTTHVPLSPAPSPRPPATSTEGASR

>446|CORE_REP|Org114_Gene798#

MSNYRIDTRTIVPGLAVTLADGVLSVTIDRPESLNSLTKPVLAGMADAIEGAATDPRVKVVRLGGAGRGFSSGGAISVDDVWASGPPTDTVAEANRTVRAIVALPQPVVAVVQGPTVGCGVSLALACDLVLASDNAFFMLAHTNVGLMPDGGASALVQAAIGRIRAMHMALLPDRVPAAEALSWGLVSAVYPAADFDAEVDKLISRLLAGPALAIAKTKNAINAATLTELAPTLLRELDGQALLLRTDDFAEGATAFQQRRTPMFTGR

>447|CORE_REP|Org119_Gene1362#

MTILEIKDLHVSVENPAEADHEIPILRGVDLTVKSGETHALMGPNGSGKSTLSYAIAGHPKYHVTSGTITLDGADVLAMSIDERARAGLFLAMQYPVEVPGVSMSNFLRSAATAIRGEPPKLRHWVKEVKAAMAALDIDPAFAERSVNEGFSGGEKKRHEILQLELLKPKIAILDETDSGPGRRRAARGQRGGEPLRRIPARRHPADHALHPHPALHPPGIRARVRRRPHRRVRWFGARRRTRPERLRAFLPRKRAVPPPTRANRSLT

>448|CORE_REP|Org47_Gene225#

MSRAVRPYLVLATQRSGSTLLVESLRATGCAGEPQEFFQYLPSTGMAPQPREWFAGVDDDTILQLLDPLDPGTPDTATPVAWREHVRTSGRTPNGVWGGKLMWNQTALLQQRAAQLPDRSGDGLRAAIRDVIGNEPVFVHVHRPDVVSQAVSFWRAVQTQVWRGHPDPKRDSQAVYHAGAIAHIIRNLRDQENGWRAWFAEEGIDPIDIAYPVLWRNLTAIVASVLDAIGQDPKLAPAPMLERQANQRSDEWVDRYRAEAPRLGLPT

>449|CORE_REP|Org59_Gene2743#

MPTTKATQRRDVSTEIAYLTRALKAPTLRESVSRLADRXRAENXSHEEXLAACLQREVSARESHGGEGRIRAARFPARKSLEEXXFEHARGLKRDTIAHLGTLDXRSPPAITSCFWAPPGTGKTHLAVGLAIRACQAGHRVLFATAAEWVARLXXAHHAGRIYAELTRLCRYPLLVVDEVGYIPFEPEAANLFFQLVSSRYERASLIVTSNKAFGRWGEVXGGDDVVAXAMIDRLVHHAEVVALKGXXYRLKXRDLGRVPPAXXTEE

>450|CORE_REP|Org69_Gene3393#

MTDTRVLAVANQKGGVAKTTTVASLGAAMVEKGRRVLLVDLDPQGCLTFSLGQDPDKLPVSVHEVLLGEVEPNAVLVTTMEGMTLLPANIDLAGAEAMLLMRAGREYALKRALAKFSDRFDVVIIDCPPSLGVLTLNGLTAADEAIVPLQCEMLAHRGVGQFLRTVADVQQITNPNLRLLGALPTLYDSRTTHTRDVLLDVADRYDLQVLAPPIPRTVRFAEASASGSSVMAGRKNKGAVAYRELAQALLKHWKTGRPLPTFTVDL

>451|CORE_REP|Org142_Gene3020#

MTDRDRLRPPLDERSLRDQLIGAGSGWRQLDVVAQTGSTNADLLARAASGADIDGVVLIAEHQTAGRGRHGRGWAATARAQIILSVGVRVVDVPVQAWGWLSLAAGLAVLDSVAPLIAVPPAETGLKWPNDVLARGGKLAGILAEVAQPFVVLGVGLNVTQAPEEVDPDATSLLDLGVAAPDRNRIASRLLRELEARIIQWRNANPQLAADYRARSLTIGSRVRVELPGGQDVVGIARDIDDQGRLCLDVGGRTVVVSAGDVVHLR

>452|CORE_REP|Org103_Gene3499#

MGQIVAGEIGGQRTTPVGGGLPLACCLDGRPPIVPHRRRRRIAALRSVLRMRDTPRPARSRCDQVTSHAVLIGWRAVPRRHGGELPRRGALALGCIALLLMGIVGCTTVTDGTAMPDTNVAPAYRSSVSASVSASAATSSIRESQRQQSLTTKAIRTSCDALAATSKDAIDKVNAYVAAFNQGRNTGPTEGPAIDALNNSASTVSGSLSAALSAQLGDALNAYVDAARAVANAIGAHASTAEFNRRVDRLNDTKTKALTMCVAAF

>453|CORE_REP|Org51_Gene430#

MTTHAVIITYLRDQTQPAVDAIGGFYRTCVLTGKALVRRPFHWREAIEQGWFITSVSLLPTLAVSIPLTVLIIFTLNILLAEFGAADISGAGAALGAVTQLGPLTTVLVIAGAGATAICADLGARTIREEIDAMEVLGIDPIHRLVVPRVVAATIVAALLNGAVITIGLVGGFVFSVFIQHVSAGAYVGTLTLVTGLPEVIISVVKSATFGLIAGLVGCYRGLTTKGGPKGVGTAVNETLVLCVIALFATNVVLTTIGVRFGTGH

>454|CORE_REP|Org69_Gene1556#

MRLYRDRAVVLRQHKLGEADRIVTLLTRDHGLVRAVAKGVRRTRSKFGARLEPFAHIEVQLHPGRNLDIVTQVVSVDAFATDIVADYGRYTCGCAILETAERLAGEERAPAPALHRLTVGALRAVADGQRPRDLLLDAYLLRAMGIAGWAPALTECARCATPGPHRAFHIATGGSVCAHCRPAGSTTPPLGVVDLMSALYDGDWEAAEAAPQSARSHVSGLVAAHLQWHLERQLKTLPLVERFYQADRSVAERRAALIGQDIAGG

>455|CORE_REP|Org75_Gene506#

MLARYIKMQLLVLLCGGLVGPIFLVVYFTLGLGSLMSWMFYVGLIITVADVLVALALTNYGAKTAAKTAALERSGVLALAQITGLSETGTRINDQPLVKVHLHISGPGITPFDTEDRVIASVTRLGNLTARKLVVLVNPATQQYLIDWERSALVNGLVPAQFTVAEDNKTYDLSGQTGPLMEILQILKANNVPLNRMVDIRSNPALRQQVQAVVRRAAERQAPAAEPASQGSIAERLAELESLRASGAVNAAEYESKRAQIISEI

>456|CORE_REP|Org118_Gene1241#

MTETILAAQIEVGEHHTATWLGMTVNTDTVLSTAIAGLIVIALAFYLRAKVTSTDVPGGVQLFFEAITIQMRNQVESAIGMRIAPFVLPLAVTIFVFILISNWLAVLPVQYTDKHGHTTELLKSAAADINYVLALALFVFVCYHTAGIWRRGIVGHPIKLLKGHVTLLAPINLVEEVAKPISLSLRLFGNIFAGGILVALIALFPPYIMWAPNAIWKAFDLFVGAIQAFIFALLTILYFSQAMELEGGTPLVPDAGNGYQSHQGG

>457|CORE_REP|Org1_Gene4344#

MTLKPAGVISSDDAPQTTIVSGGVRLLTMWSTVLVLALSVICEPVRIGLVVLMLNRRRPLLHLLTFLCGGYTMAGGVAMVTLVVLGATPLAGHFSVAEVQIGTGLIALLIAFALTTNVIGKHVRRATHARVGDDGGRVLRESVPPSGAHKLAVRARCFLQGDSLYVAGVSGLGAALPSANYMGAMAAILASGATPATQALAVVTFNVVAFTVAEVPLVSYLAAPRKTRAFMAALQSWLRSRSRRDAALLVAAGGCLMLTLGLSNL

>458|CORE_REP|Org104_Gene1889#

MTKTWPPRTVIRKSGGLRGMRTLESALHRGGLGPVAGVDEVGRGACAGPLVVAACVLGPGRIASLAALDDSKKLSEQAREKLFPLICRYAVAYHVVFIPSAEVDRRGVHVANIEGMRRAVAGLAVRPGYVLSDGFRVPGLPMPSLPVIGGDAAAACIAAASVLAKVSRDRVMVALDADHPGYGFAEHKGYSTPAHSRALARLGPCPQHRYSFINVRRVASGSNTAEVADGQPDPRDGTAQTGEGRWSKSSHPATMRATGRAQGT

>459|CORE_REP|Org9_Gene2357#

MSKTAESLTHPAYGQLRAVTDTASVLLADNPGLLTLDGTNTWVLRGPLSDELVVVDPGPDDDEHLARVAALGRIALVLISHRHGDHTSGIDKLVALTGAPVRAADPQFLRRDGETLTDGEVIDVAGLTITVLATPGHTADSLSFVLDDAVLTADTVLGCGTTVIDKEDGSLADYLESLHRLRGLGRRTVLPGHGPDLLDLEAIASGYLLHRHERLEQIRAALRDLGDDATVREVVEHVYLDVDEKLWNAAEWSVQAQLDYLRTR

>460|CORE_REP|Org125_Gene338#

MSLDKKLMPVPDGHPDVFDREWPLRVGDIDRAGRLRLDAACRHIQDIGQDQLREMGFEETHPLWIVRRTMVDLIRPIEFGDMLRCRRWCSGTSNRWCEMRVRVDGRKGGLIESEAFWIHVNRETEMPARIADDFLAGLHRTTSVDRLRWKGYLKPGSRDDASEIHEFPVRVTDIDLFDHMNNAVYWSVIEDYLASHAELLRGPLRVTIEHEAPVALGDKLEIISHVHPAGSTEIFGPGLVDRAVTTLTYVVGDEPKAVASLFNL

>461|CORE_REP|Org59_Gene2621#

MLREGRLCALGGAVVTVEADVDQVERRLAAGELSCPSCGGVLAGWGRARSRQLRGPAGPVELCPRRSRCTGCGVTHVLLPVSALLRRADTAAVIVSALAAKATSRVGFRRIATDVARPAETVRGWLRRFAERVEAVRSVFTVWLCAVDADPVMPDAGGGGFVDAVVAIGALAAAIGRRFSLPTVSLAETAVAVSGGRLLAPGXPGEWVQHGVXXAVAVDRAVNLCAVVCFDRQQMERSGRWRSAMTRRRCARSARGRXGCFATS

>462|CORE_REP|Org7_Gene3616#

MSDGGAPTVEFLRHGGRIAMAHRGFTSFRLPMNSMGAFQEAAKLGFRYIETDVRATRDGVAVILHDRRLAPGVGLSGAVDRLDWRDVRKAQLGAGQSIPTLEDLLTALPDMRVNIDIKAASAIEPTVNVIERCNAHNRVLIGSFSERRRRRALRLLTKRVASSAGTGALLAWLTARPLGSRAYAWRMMRDIDCVQLPSRLGGVPVITPARVRGFHAAGRQVHAWTVDEPDVMHTLLDMDVDGIITDRADLLRDVLIARGEWDGA

>463|CORE_REP|Org104_Gene167#

MVHHGQMHAQPGVGLRPDTPVASGQLPSTSIRSRRSGISKAQRETWERLWPELGLLALPQSPRGTPVDTRAWFGRDAPVVLEIGSGSGTSTLAMAKAEPHVDVIAVDVYRRGLAQLLCAIDKVGSDGINIRLILGNAVDVLQHLIAPDSLCGVRVFFPDPWPKARHHKRRLLQPATMALIADRLVPSGVLHAATDHPGYAEHIAAAGDAEPRLVRVDPDTELLPISVVRPATKYERKAQLGGGAVIELLWKKHGCSERDLKIR

>464|CORE_REP|Org36_Gene3699#

MNNRPIRLLTSGRAGLGAGALITAVVLLIALGAVWTPVAFADGCPDAEVTFARGTGEPPGIGRVGQAFVDSLRQQTGMEIGVYPVNYAASRLQLHGGDGANDAISHIKSMASSCPNTKLVLGGYSQGATVIDIVAGVPLGSISFGSPLPAAYADNVAAVAVFGNPSNRAGGSLSSLSPLFGSKAIDLCNPTDPICHVGPGNEFSGHIDGYIPTYTTQAASFVVQRLRAGSVPHLPGSVPQLPGSVLQMPGTAAPAPESRHGR

>465|CORE_REP|Org142_Gene639#

MTGPTEESAVATVADWPEGLAAVLRGAADQARAAVVEFSGPEAVGDYLGVSYEDGNAATHRFIAHLPGYQGWQWAVVVASYSGADHATISEVVLVPGPTALLAPDWVPWEQRVRPGDLSPGDLLAPAKDDPRLVPGYTASGDAQVDETAAEIGLGRRWVMSAWGRAQSAQRWHDGDYGPGSAMARSTKRVCRDCGFFLPLAGSLGAMFGVCGNELSADGHVVDRQYGCGAHSDTTAPAGGSTPIYEPYDDGVLDIIEKPAES

>466|CORE_REP|Org142_Gene1047#

MTSAPTVSVITISFNDLDGLQRTVKSVRAQRYRGRIEHIVIDGGSGDDVVAYLSGCEPGFAYWQSEPDGGRYDAMNQGIAHASGDLLWFLHSADRFSGPDVVAQAVEALSGKGPVSELWGFGMDRLVGLDRVRGPIPFSLRKFLAGKQVVPHQASFFGSSLVAKIGGYDLDFGIAADQEFILRAALVCEPVTIRCVLCEFDTTGVGSHREPSAVFGDLRRMGDLHRRYPFGGRRISHAYLRGREFYAYNSRFWENVFTRMSK

>467|CORE_REP|Org25_Gene255#

MRKPASSLAKVDYSSAYLEQTHAFGELIRNVDQSTPVPTCPGWSLGQLFRHVGRGDRWAAQIVRDRLDHFLDPRSVEGGKPPPDPDDAISWLYGGARLLVDAVEQTGVETPVWTFLGPRPAGWWVRRRLHEVAVHRADVAITVGGEFTLEPNVAADGISEFLERIAVQAGSGGTPLPLEDDDTLHLHATDPGLLEAGEWTVRRDERGVTWSHRHGKGAVALRGGATELLLAMVRRLSVADTGIELLGDAGVWQKWLDRTPL

>468|CORE_REP|Org99_Gene2472#

MAEPFFRMMEILVPSIVAANGNKITFEGLENIPERGGALIALNHTSYVDWVPASIAAHHRRRRLRFMIKAEMQDVRAVNYVIKHAQLIPVDRSVGADAYAVAVQRLRAGELVGLHPEATISRSLELREFKTGAARMALEAQVPIIPMIVWGAHRIWPKDHPKNLFRNKIPIVAAIGSPVRPEGNAEQLNAVLRQAMNAILYRVQEEYPHPKGEHWVPRRLGGGAPTVEESRQLRIAELAKRRQKRGYDGVTSSRRSQVGPH

>469|CORE_REP|Org54_Gene1219#

MNGLISQACGSHRPRRPSSLGAVAILIAATLFATVVAGCGKKPTTASSPSPGSPSPEAQQILQDSSKATKGLHSVHVVVTVNNLSTLPFESVDADVTNQPQGNGQAVGNAKVRMKPNTPVVATEFLVTNKTMYTKRGGDYVSVGPAEKIYDPGIILDKDRGLGAVVGQVQNPTIQGRDAIDGLATVKVSGTIDAAVIDPIVPQLGKGGGRLPITLWIVDTNASTPAPAANLVRMVIDKDQGNVDITLSNWGAPVTIPNPAG

>471|CORE_REP|Org59_Gene2937#

MSHDDLMLALALADRADELTRVRFGALDLRIDTKPDLTPVTDADRAVESDVRQTLGRDRPGDGVLGEEFGGSTTFTGRQWIVDPIDGTKNFVRGVPVWASLIALLEDGVPSVGVVSAPALQRRWWAARGRGAFASVDGARPHRLSVSSVAELHSASLSFSSLSGWARXGLRERFIGLTDTVWRVRAYGDFLSYCLVAEGAVDIAAEPQVSVWDLAALDIVVREAGGRLTSLRRTXAGPHGGSAVATNGLLHDEVLTRLNAG

>473|CORE_REP|Org20_Gene2219#

MSTGPHPRQSHRDAFAGNSFRIRNGDTHTGLSDHPDGPWPDDRAAGGARLRWVHCWGRTWAGAQVFGPSSPLSGMLTVAVLLVVTRGLHIDGVADTADGLGCYGPPQRALAVMRDGYAGPFGVAAVVLVIALQGLAFATLTTVGIAGITLAVLSGRVTAVLVCRRLVPAAHGSTLGSRVAGTQPAPVVAAWLAVLLAVSVPAGPRPWQGPIAVLVAVTAGAALAAHCVHRFGGVTGDVLGSAIELSTTVSAVTLAGLARL

>474|CORE_REP|Org135_Gene2254#

MEHDVATSPPAGWYTDPDGSAGQRYWDGDRWTRHRRPNPSAPRSPLALRVDGLRSRWLGMPAGLRLTVPVAAVLTMVGVAVYAWIRPLPDDWSQLPKRLSCQLRPGPTPPATITVASVDVSHPRGAVLRLVVRFAEPLPPSPSGSFASGFAGYLLTYTIANNGKEFAELGPQQDTDELAIRKPGESRGTEPNMRPDRNTNARRTAPDTVEINLETKRLGLDQAPVDPQLTFAAQFRTPSTVTVDFGSQFCQGERLAGQRR

>475|CORE_REP|Org118_Gene317#

MLLSDRDLRAEISSGRLGIDPFDDTLVQPSSIDVRLDCLFRVFNNTRYTHIDPAKQQDELTSLVQPVDGEPFVLHPGEFVLGSTLELFTLPDNLAGRLEGKSSLGRLGLLTHSTAGFIDPGFSGHITLELIQRRQPADHFVARHENRSAVHVAPDQPVRASLRQFPGGVEIPGSARAHAVALLPELHQVYLASGAARPVAGSCHLPFAWCSAPRCGSLIAATYTQWCAMQRLRHWVSGCHPRRRYGATGTRGRRGRYRSR

>476|CORE_REP|Org44_Gene2475#

MEPVYGTVIRLARLSWRIQGLKITVTGVDNLPTSGGAVVAINHTSYLDFTFAGLPAYQQGLGRKVRFMAKQEVFDHKITGPIMRSLRHIPVDRQDGSASYDAAVRMLKAGELVGVYPEATISRSFEIKEFKTGAARMAIEAGVPIVPHIVWGAQRIWTKDRPKKLFRPKVPVTIVVGERIEPTLPTAELNGLLHSRMQHLLERAQELYGPHPAGEFWVPHRLGGGAPSLAEAARLDAQEAAVRAARRAQRAHPAGAPEQ

>477|CORE_REP|Org62_Gene2295#

MTLAEAADAINFGLAGRVVLVTGGVRGVGAGISSVFAEQGATVITCARRAVDGQPYEFHRCDIRDEDSVKRLVGEIGERHGRLDMLVNNAGGSPYALAAEATHNFHRKIVELNVLAPLLVSQHANVLMQAQPNGGSIVNICSVSGRRPTPGTAAYGAAKAGLENLTTTLAVEWAPKVRVNAVVVGMVETERSELFYGDAESIARVAATVPLGRLARPADIGWAAAFLASDAASYISGATLEVHGGGEPPPYLGASSANK

>478|CORE_REP|Org148_Gene939#

MSKREDGRLDHELRPVIITRGFTENPAGSVLIEFGHTKVLCTASVTEGVPRWRKATGLGWLTAEYAMLPSATHSRSDRESVRGRLSGRTQEISRLIGRSLRACIDLAALGENTIAIDCDVLQADGGTRTAAITGAYVALADAVTYLSAAGKLSDPRPLSCAIAAVSVGVVDGRIRVDLPYEEDSRAEVDMNVVATDTGTLVEIQGTGEGATFARSTLDKLLDMALGACDTLFAAQRDALALPYPGVLPQGPPPPKAFGT

>479|CORE_REP|Org127_Gene2951#

MQREIYDGEARLSWVLAALAGILGATAFTHSAGYFVTFMTGNSQRAVLGLFGDDAWMSVTASLLILFFVAGVVIASVCRRHFWAAHPHGPTVLTTFSLIFAAGVDIMLGGWHESMLDFVPILFVVFGIGALNTSFVKDGEVSVPLSYVTGTLVKMGQGIERHLAGGKVEDWLGYFLLHASFVLGAAAGGAISMVVTGPQMLAVAAVVCAATTGYTYLHADRRGLVNQKRPQPGKRLFRALRRGELDSGTSTPATNYGSS

>480|CORE_REP|Org77_Gene1755#

MPDSGQLGAADTPLRLLSSVHYLTDGELPQLYDYPDDGTWLRANFISSLDGGATVDGTSGAMAGPGDRFVFNLLRELADVIVVGVGTVRIEGYSGVRMGVVQRQHRQARGQSEVPQLAIVTRSGRLDRDMAVFTRTEMAPLVLTTTAVADDTRQRLAGLAEVIACSGDDPGTVDEAVLVSQLAARGLRRILTEGGPTLLGTFVERDVLDELCLTIAPYVVGGLARRIVTGPGQVLTRMRCAHVLTDDSGYLYTRYVKT

>481|CORE_REP|Org119_Gene856#

MVAVSTAAKSPTALAIAVRTQDSVVILTADGALDSSSSALLRDSLTRATLEQPSAVIVNVTELQVAEESAWSVFISARWQADFRADVPVLLVCGHRAGRAAVTRTGVARFMPVYPTEKAASKAIGRLARRNFKRSDAQLPANLNSLRESRQLVREWLTQWSRPGLIPVALVVVNVFVENVLKHTGSDPVMRIESDGPTATIAVSDGSSAPAVRLASPPKGIDVSGLAIVAALSRAWGPRCPTSSGKTVWAIIGPENQL

>482|CORE_REP|Org31_Gene855#

MELLGGPRVGNTESQLCVADGDDLPTYCSANSEDLNITTITTLSPTSMSHPQQVRDDQWVEPSDQLQGTAVFDATGDKATMPSWDELVRQHADRVYRLAYRLSGNQHDAEDLTQETFIRVFRSVQNYQPGTFEGWLHRITTNLFLDMVRRRARIRMEALPEDYDRVPADEPNPEQIYHDARLGPDLQAALASLPPEFRAAVVLCDIEGLSYEEIGATLGVKLGTVRSRIHRGRQALRDYLAAHPEHGECAVHVNPVR

>483|CORE_REP|Org22_Gene721#

MTTMSGYTRSQRPRQAILGQLPRIHRADGSPIRVLLVDDEPALTNLVKMALHYEGWDVEVAHDGQEAIAKFDKVGPDVLVLDIMLPDVDGLEILRRVRESDVYTPTLFLTARDSVMDRVTGLTSGADDYMTKPFSLEELVARLRGLLRRSSHLERPADEALRVGDLTLDGASREVTRDGTPISLSSTEFELLRFLMRNPRRALSRTEILDRVWNYDFAGRTSIVDLYISYLRKKIDSDREPMIHTVRGIGYMLRPPE

>484|CORE_REP|Org38_Gene3787#

MTRSKRGSADGGSAEALPPKSLRQFVGGAYKEVGAEFVGYLVDLCGLQPDEAVLDVGCGSGRMALPLTGYLNSEGRYAGFDISQKAIAWCQEHITSAHPNFQFEVSDIYNSLYNPKGKYQSLDFRFPYPDASFDVVFLTSVFTHMFPPDVEHYLDEISRVLKPGGRCLCTYFLLNDESLAHIAEGKSAHNFQHEGPGYRTIHKKRPEEAIGLPETFVRDVYGKFGLAVHEPLHYGSWSGREPHLSFQDIVIATKTAS

>485|CORE_REP|Org102_Gene3785#

MADKSKRPPRFDLKSADGSFGRLVQIGGTTTIVVVFAVVLVFYIVTSRDDKKDGVAGPGDAVRVTSSKLVTQPGTSNPKAVVSFYEDFLCPACGIFERGFGPTVSKLVDIGAVAADYTMVAILDSASNQHYSSRAAAAAYCVADESIEAFRRFHAALFSKDIQPAELGKDFPDNARLIELAREAGVVGKVPDCINSGKYIEKVDGLAAAVNVHATPTVRVNGTEYEWSTPAALVAKIKEIVGDVPGIDSAAATATS

>486|CORE_REP|Org59_Gene956#

MAKSASNQLRVTVRTETGKGASRRARRAGKIPAVLYGHGAEPQHLELPGHDYAAVLRHSGTNAVLTLDIAGKEQLALTKALHXHPIRRTIQHADLLVVRRGEKVVVEVSVVVEGQAGPDTLVTQETNSIEIEAEALSIPEQLTVSIEGAEPGTQLTAGQIALPAGVSLISDPDLLVVNVVKAPXAEEXXGXXAXEXXKPRKPRXKPAKPRPLASPSRRLATWPSRCSWSASATLEPITPVPGTTSGSWSPICSPRD

>488|CORE_REP|Org80_Gene3146#

MPEGDTVWHTAATLRRHLAGRTLTRCDIRVPRFAAVDLTGEVVDEVISRGKHLFIRTGTASIHSHLQMDGSWRVGNRPVRVDHRARIILEANQQEQAIRVVGVDLGLLEVIDRHNDGAVVAHLGPDLLADDWDPQRAAANLIVAPDRPIAEALLDQRVLAGIGNVYCNELCFVSGVLPTAPVSAVADPRRLVTRARDMLWVNRFRWNRCTTGDTRAGRRLWVYGRAGQGCRRCGTLIAYDTTDERVRYWCPACQR

>489|CORE_REP|Org35_Gene2559#

MMISSSDELLRDGADPAVIIDQLRVIRGKRLALQDVSVRVACGTITGLLGPSGSGKTTLIRCIVGSQIIASGSVSVLGQPAGSAELRHRVGYMPQDPTIYNDLRVIDNIRYFAELCGVDRQAADEVIEAVDLRDHRTARCANLSGGQRARVSLACALVGRPDLLVLDEPTIGLDPVLRVELWDRFTALARRGTTLLVSSHVMDEADRCGDLLLLRQGQLLAHTTPHRLRKETGCTSLEEAFLSIVRRTTTVPAAG

>490|CORE_REP|Org6_Gene2931#

MSTATSLLGEKRLARMLARPVSAPVLSGDTANEGTQLIKMADIDGVTGSAGLQPGPSEETDEELTARFERDAIPLLDQLYGGALRMTRNPADAEDLLQETMVKAYAGFRSFRHGTNLKAWLYRILTNTYINSYRKKQRQPAEYPTEQITDWQLASNAEHSSTGLRSAEVEALEALPDTEIKEALQALPEEFRMAVYYADVEGFPYKEIAEIMDTPIGTVMSRLHRGRRQLRGLLADVARDRGFARGEQAHEGVSS

>491|CORE_REP|Org40_Gene3637#

MWTMVLLLGLGMAIDPARLGLAVVMLSRRRPMLNLFAFWVGGMVAGVGIALAVLVFMRDVALAAIQGVVSAANEFREAVGILAGGRLHIVIGVIMLLLAARMVARARAQVGVPVGPVGVADGGMSALALAQRPPGLVARLEVRTQQMLQGDVVWPAFVVGVASSAPPFESVVALTVIMASGAEIGTQLGAFVVFTLLVLAVIEIPLVAYLAIPQQTQQVMLRFQDWVRSNRRQISLTILIGVGFLFLYQGVTSL

>492|CORE_REP|Org107_Gene2468#

MVLDAVGNPQTVLLLGGTSEIGLAICERYLHNSAARIVLACLPDDPRREDAAAAMKQAGARSVELIDFDALDTDSHPKMIEAAFSGGDVDVAIVAFGLLGDAEELWQNQRKAVQIAEINYTAAVSVGVLLAEKMRAQGFGQIIAMSSAAGERVRRANFVYGSTKAGLDGFYLGLSEALREYGVRVLVIRPGQVRTRMSAHLKEAPLTVDKEYVANLAVTASAKGKELVWAPAAFRYVMMVLRHIPRSIFRKLPI

>493|CORE_REP|Org59_Gene1729#

MTVAPRRLAWTNARQSYPVRVAHVLSVNLARVRANPDPRSXDSSKLIRNRQSXLXSEAVMVRAPGSMHAGVGSGLVGDTVGNPKLHGGDDQAVYAYAREDLDAWETQLHRTLHNGMFGENLTTSGVDVTYARIGERWRIGSDGLVLEVSAPRIPCRTFAAFLDLRYWIKTFXRAAKPGAYLRVIAPGTVRAGDTITVDYRPEHNVTVGLVFRARTSESELLPQLLAADALAAELKAYARERTPSPPPVDSADDV

>494|CORE_REP|Org66_Gene975#

MTVVPGAPSRPASAVSRPSYRQCVQASAQTSARRYSFPSYRRPPAEKLVFPVLLGILTLLLSACQTASASGYNEPRGYDRATLKLVFSMDLGMCLNRFTYDSKLAPSRPQVVACDSREARIRNDGFHANAPSCMRIDYELITQNHRAYYCLKYLVRVGYCYPAVTTPGKPPSVLLYAPSACDESLPSPRVATALVPGTRSANREFSRFVVTEIKSLGAGGRCDSASVSLQPPEEIEGPAIPPASSQLVCVAPK

>495|CORE_REP|Org121_Gene1741#

MRAISSLAGPRALAAFGRNDIRGTYRDPLLVMLVIAPVIWTTGVALLTPLFTEMLARRYGFDLVGYYPLILTAFLLLTSIIVAGALAAFLVLDDVDAGTMTALRVTPVPLSVFFGYRAATVMVVTTIYVVATMSCSGILEPGLVSSLIPIGLVAGLSAVVTLLLILAVANNKIQGLAMVRALGMLIAGLPCLPWFISSNWNLAFGVLPPYWAAKAFWVASDHGTWWPYLVGGAVYNLAIVWVLFRRFRAKHA

>496|CORE_REP|Org33_Gene304#

MAESKLVIGDRSFASRLIMGTGGATNLAVLEQALIASGTELTTVAIRRVDADGGTGLLDLLNRLGITPLPNTAGCRSAAEAVLTAQLAREALNTNWVKLEVIADERTLWPDAVELVRAAEQLVDDGFVVLPYTTDDPVLARRLEDTGCAAVMPLGSPIGTGLGIANPHNIEMIVAGARVPVVLDAGIGTASDAALAMELGCDAVLLASAVTRAADPPAMAAAMAAAVTAGYLARCAGRIPKRFWAQASSPAR

>497|CORE_REP|Org112_Gene1036#

MTAPAICNTTETVHGIATSLGAVARQASLPRIVGTVVGITVLVVVALLVPVPTAVELRDWAKSLGAWFPLAFLLVHTVVTVPPFPRTAFTLAAGLLFGSVVGVFIAVVGSTASAVIAMLLVRATGWQLNSLVRRRAINRLDERLRERGWLAILSLRLIPVVPFAAINYAAGASGVRILSFAWATLAGLLPGTAAVVILGDAFAGSGSPLLILVSVCTGALGLTGLVYEIRNYRRQHRRMPGYDDPVREPALI

>498|CORE_REP|Org146_Gene2635#

MARTFEDLVAEAASASVGGWDFSWLDGRATEERPSWGYQRQLSQRLANATAALDLETGGGEVLAGAGNFPPTMVATEAWPPNAAMATRRLHPLGAVVVITGDKPPLPFADAAFDLVTSRHPSTRWWTEIARVLRAGGSYFAQHVGPATLWDLREHFLGPREHNGADQYAQVVRTCITDAGLEIVDLQMERLRVEFFDVGAVIYFLRKVIWFLPDFTVEGYHDRLRALHERIQAEGPFVTYSTRALIEARKPS

>499|CORE_REP|Org92_Gene714#

MRPPLAPQFAADLLVKTVSTLRSSGAALGRLTTMRKAVLAVGSVCWLVGCSSGASSTTASTGDIAKVAEVKSGFGPEYTVTDVTPRAIDPGFFSARKLPDGLSFDPANCAQVAAGPQLPTGLQGNMAAVSAEGNGNRFVVIAVETSQPLPAPSPGKDCSKVTFSGTQLRGGIEVVDVPHIDGTQTLGVHRVLQAVVGGSARTGELYDYSARFGDYQVIVIANPLVIPGRPVARVDTQRARDLLVQAVAAVRG

>500|CORE_REP|Org125_Gene1892#

MPTGPTTGKWHPHEVWRYLLEVLLLTDEADLESALPELESFAQSVQRAPLDDPGAAKGADADVAIIDARADLAAARRVCRRLTTSAPALAVVAVVAPANFVAVDGDWIFDDVLLNAAGGAELQARLRLAITRRRSTLAGTLQFGDLVLHPASYTASLGDRDLGLTLTEFKLMNFLVQHAGRAFTRTRLMREVWGYECHGRIRTVDVHVRRLRAKLGAEHESMIDTVRGVGYMAVTPPQPRWIISESILNRCK

>501|CORE_REP|Org20_Gene2115#

MAVRSEFSVFHSPEQAMRERSELARKGIARAKSVVALAYAGGVLFVAENPSRSLQKISELYDRVGFAAAGKFNEFDNLRRGGIQFADTRGYAYDRRDVTGRQLANVYAQTLGTIFTEQAKPYEVELCVAEVAHYGETKPPELYRITYDGSIAEEPHFVVMGGTTEPIANALKESYAENASLTDALGIAVAALRAGSADTSGGDQPTLGVASLEVAVLDANRPRRAFRRITGSALQALLVDQESPQSDGESSG

>502|CORE_REP|Org111_Gene1716#

MSGHSKWATTKHKKAVVDARRGKMFARLIKNIEVAARVGGGDPAGNPTLYDAIQKAKKSSVPNENIERARKRGAGEEAGGADWQTIMYEGYAPNGVAVLIECLTDNRNRAASEVRVAMTRNGGTMADPGSVSYLFSRKGVVTLEKNGLTEDDVLAAVLEAGAEDVNDLGDSFEVISEPAELVAVRSALQDAGIDYESAEASFQPSVSVPVDLDGARKVFKLVDALEDSDDVQNVWTNVDVSDEVLAALDDE

>503|CORE_REP|Org135_Gene3644#

MTVYFIGAGPGAADLITVRGQRLLQRCPVCLYAGSIMPDDLLAQCPPGATIVDTGPLTLEQIVRKLADADADGRDVARLHSGDPSLYSALAEQCRELDALGIGYEIVPGVPAFAAAAAALKRELTVPGVAQTVTLTRVATLSTPIPPGEDLAALARSRATLVLHLAAAQIDAIVPRLLDGGYRPETPVAVVAFASWPQQRTLRGTLADIAARMHDAKITRTAVIVVGDVLTAEGFTDSYLYSVARHGRYAQ

>504|CORE_REP|Org148_Gene3144#

MHHNRDVDLALVERPSSGYVYTTGWRLATTDIDEHQQLRLDGVARYIQEVGAEHLADAQLAEVHPHWIVLRTVIDVINPIELPSDITFHRWCAALSTRWCSMRVQLQGSAGGRIETEGFWICVNKDTLTPSRLTDDCIARFGSTTENHRLKWRPWLTGPNIDGTETPFPLRRTDIDPFEHVNNTIYWHGVHEILCQIPTLTAPYRAVLEYRSPIKSGEPLTIRYEQHDDVVRMHFVVGDDVRAAALLRRL

>505|CORE_REP|Org101_Gene955#

MFMALRAPMLERMNGLHTDDAPVNWLERRGGRLTSRRRVTLLHAGVEHPMRLWGVQSEAITAAMVLSRKVSAIIAGHCGVRLVDQGVGDGFVAAFAHASDAVACALELHQAPLSPIVLRIGIHTGEAQLVDERIYAGATMNLAAELRDLAHGGQTVMSGATEDAVLGRLPMRAWLIGLRPMEGSPEGHNFPQSQRIAQLCHPNLRNTFPPLRMRIADASGIPYVGRILVNVQVVPHWEGGCAAAGMVLAG

>506|CORE_REP|Org59_Gene319#

MAGQSDRKAALLDQVARVGKALANGRRLQILDLLAQGERAVEAIATATGMNLTTASANLQALKSGGLVEARREGTRQYYRIAGEDVARLFALVQVVADEHLADVAVAAADVLGSPEDAITRAELLRRREAGEVTLVDVRPHEEYQAGHIPGAINIPIAELXDRLXELTGDXXIVAYXPWCLLRHGPRCRPHRARRGAGGETPRRRNARMAIGRTAGRRGCTGRAWGLIARGAEGKSTFGEAAARTARCPA

>507|CORE_REP|Org20_Gene2401#

MQRFAENLVFTEAPKLVRHLQNTQETLRTIRQAVKITANIMTTAVPSPPAEIAAGRPVTSTSCPTAARARRLVYAPDLDGRADPGEIVWTWVAYEQDPTRGKDRPVLVVGRDRSVLLGLLVSSQERHAADRDWVGIGSGAWDYEGRESWVRLDRVLDVPEESIRREGAILEREVFDVVAARLRADYAWRKPGRAASAIGWATSPDQAPISPAWRRRGPPSGPLRSRAVSPARRRCGPPSGPLRSRAISPE

>508|CORE_REP|Org13_Gene3073#

MPPTEGKSTTNRDEGIQVLRRAVAALDEIAAEPGHLRLVDLCERLGLAKSTTRRLLVGLVEVGLVSVDSHGRFALGERLLGFGSVTGAHIAAAFRPTVERVARATDGETVDLSVLRGQRMWFVDQIESSYRLRAVSAVGLRFPLNGTANGKAALAALDDADAEAALCRLDPMVAEGLRREIVEIRRTGIAFDRNEHTPGISAAAIARRALGDNVIAISVPAPTARFLEKEQRIIAALRAAADSPDWTR

>509|CORE_REP|Org119_Gene2031#

MWYYLFKYIFMGPLFTLLGRPKVEGLEYIPSSGPAILASNHLAVADSFYLPLVVRRRIWFLAKSEYFTGTGLKGWINRWFYSVSGQVPIDRTNADSAQGALQTAVVLLGQGKLLGMYPEGTRSPDGRLYKGKTGLARLALHTGVPVIPVAMIGTNVVNPPGRKMLRFGRVTVRFGKPMDFSRFEGLAGNHFIERAVTDEVIYELMGLSGQEYVDIYAASVVKDGRNAGGAGANPNSTDAARIPETAAG

>510|CORE_REP|Org122_Gene995#

MSSSIEIFPDSDILVAAAGKRLVGAIGAAVAARGQALIVLTGGGNGIALLRYLSAQAQQIEWSKVHLFWGDERYVPEDDDERNLKQARRALLNHVDIPSNQVHPMAASDGDFGGDLDAAALAYEQVLAASAAPGDPAPNFDVHLLGMGPEGHINSLFPHSPAVLESTRMVVAVDDSPKPPPRRITLTLPAIQRSREVWLLVSGPGKADAVAAAIGGADPVSVPAAGAVGRQNTLWLLDRDAAAKLPS

>511|CORE_REP|Org131_Gene199#

MNLILTAHGTRRPSGVAMIADIAAQVSALVDRTVQVAFVDVLGPSPSEVLSALSCRPAIVVPAFLSRGYHVRTDLPAHVAASAHPHVTVTPALGPCREIAQIVTQQLVESGWRPGDSVILAAAGASDRRARADLHTTRTLVSELTGSWVDMGFAGTGGPDVRTAVQRARDRAEANRGARRVVVASFLLAEGLFQERLRASGADVVTRPLGTHPGLAQLVANRFRSAVARQQRLHRWHGTPTPVTLDL

>512|CORE_REP|Org1_Gene1397#

MLGRRSNQARLPDGVANWTYAAGMDGTPGHDDMPGQPAPSRGESLWAHAEGSISEDVILAGARERATDIGAGAVTPAVGALLCLLAKLSGGKAVAEVGTGAGVSGLWLLSGMRDDGVLTTIDIEPEHLRLARQAFAEAGIGPSRTRLISGRAQEVLTRLADASYDLVFIDADPIDQPDYVAEGVRLLRSGGVIVVHRAALGGRAGDPGARDAEVIAVREAARLIAEDERLTPALVPLGDGVLAAVRD

>513|CORE_REP|Org59_Gene543#

MSTVLTYIRAVDIYEHMTESLDLEFESAYRGESVAFGEGXRPPWSIGEPQPELAALIVQGKFRGDVLDVGCGEAAISLALAERGHTTVGLDLSPAAVELARHEAAKRGLANASFEVADASSFTGYDGRXRHHRRQHAVPLHAGRVPGGLSAIDRACGGTGRXRHTSTXCVXSXGRRXRRGAGXXCGHRGRCCARRLSKYWIIDEIKPARLYARFPAGFAGMPXLLDIREEPNGLQSIGGWLXSAHLG

>514|CORE_REP|Org119_Gene2715#

MNDGKRAVTSAVLVVLGACLALWLSGCSSPKPDAEEQGVPVSPTASDPALLAEIRQSLDATKGLTSVHVAVRTTGKVDSLLGITSADVDVRANPLAAKGVCTYNDEQGVPFRVQGDNISVKLFDDWSNLGSISELSTSRVLDPAAGVTQLLSGVTNLQAQGTEVIDGISTTKITGTIPASSVKMLDPGAKSARPATVWIAQDGSHHLVRASIDLGSRVDSAHAVEMERTRQRRLGRSCVDALLETPL

>515|CORE_REP|Org39_Gene251#

MARSIPADRFSAIVAASARVFIAHGYQRTQVQDVADALALAKGTLYGYAQGKAALFAAAVRYGDAQEALPLASELPVAAPVAGEIAAVVSARLAGEVTDMRLTHALRATLPPGATTGDARAELAGIVTDLYSRLARHRIALKLVDRCAPELPDLAEVWFGTGRNAQVDAVQAYLVHRERAGLLILPGPAPMVARTIVELCALWAVHLHFDPSPEPWSIVQPGVIDDDAIAATLAEFVVRATTASSD

>516|CORE_REP|Org51_Gene823#

MAVLDLVEIFWDAAPYVVVAIAVVGTWWRYRYDKFGWTTRSSQLYESRLLSIGSPMFHFGSLLVIMGHVMGLFIPDSWTRAFGMSDHLYHLQALLLGAPAGFATLLGIGLLIYRRRIQTPVWLATTRNDKLMYLVLVCAIVAGLACTLMGATHEGDMHDYRRSVSVWFRSIWMLAPRGDLMAQATLYYQVHVLIALALFALWPFTRLVHAFSAPIAYLFRPYIVYRSREVAAKHELIGSAPRRRGW

>517|CORE_REP|Org136_Gene1811#

MPKTTDTAATPDGTCAVRLFTPDGPGRWPGVVMFPDAGGVRDTFDRMAAKLAGFGYVVLLPDVYYREGDWAPFDMKTAFGDPQERARIMFMIGTLTPDRVTRDADALLNYLASRPEVIGDRFGVCGYCMGGRMSVVVAGRLPDRVAAAAAFHPGGLVANSPDSPHLLADRISATVYIGGAENDPSFTADHAEKLDKAFSAAGVPHRIECYPAAHGFAVPDNPSYDAAADERHWAAMTETFGAALN

>518|CORE_REP|Org91_Gene1477#

MKAGVAQQRSLLELAKLDAELTRIAHRATHLPQRAAYQQVQAEHNAANDRMAALRIAAEDLDGQVSRFESEIDAVRKRGDRDRSLLTSGATDAKQLADLQHELDSLQRRQASLEDALLEVLERREELQAQQTAESRALQALRADLAAAQQALDEALAEIDQARHQHSSQRDMLTATLDPELAGLYERQRAGGGPGAGRLQGHRCGACRIEIGRGELAQISAAAEDEVVRCPECGAILLRLEGFEE

>519|CORE_REP|Org92_Gene1926#

MYRVFEALDELSAIVEEARGVPMTAGCVVPRGDVLELIDDIKDAIPGELDDAQDVLDARDSMLQDAKTHADSMVSSATTEAESILNHARTEADRILSDAKAQADRMVSEARQHSERMVADAREEAIRIATAAKREYEASVSRAQAECDRLIENGNISYEKAVQEGIKEQQRLVSQNEVVAAANAESTRLVDTAHAEADRLRGECDIYVDNKLAEFEEFLNGTLRSVGRGRHQLRTAAGTHDYAVR

>520|CORE_REP|Org75_Gene2388#

MPGRWSAETRLALVRRARRMNRALAQAFPHVYCELDFTTPLELAVATILSAQSTDKRVNLTTPALFARYRTARDYAQADRTELESLIRPTGFYRNKAASLIGLGQALVERFGGEVPATMDKLVTLPGVGRKTANVILGNAFGIPGITVDTHFGRLVRRWRWTTAEDPVKVEQAVGELIERKEWTLLSHRVIFHGRRVCHARRPACGVCVLAKDCPSFGLGPTEPLLAAPLVQGPETDHLLALAGL

>521|CORE_REP|Org22_Gene653#

MTTTSDQNAAAPPRFDGLRALFINATLKRSPELSHTDGLIERSSGIMREHGVQVDTLRAVDHDIATGVWPDMTEHGWATDEWPALYRRVLDAHILVLCGPIWLGDNSSVMKRVIERLYACSSLLNEDGQYAYYGRAGGCLITGNEDGVKHCAMNVLYSLQHLGYTIPPQADAGWIGEAGPGPSYLDPGSGGPENDFTNRNTTFMTFNLMHIAQMLRVAGGIPAYGNQRTKWDAGCRPDFANPDYR

>522|CORE_REP|Org19_Gene3763#

MSSRCRLDQLEDSGLMTTVDFHFDPLCPFAYQTSVWIRDVRAQLGITINWRFFSLEEINLVAGKKHPWERDWSYGWSLMRIGALLRRTNMSLLDRWYAAIGHELHTLGGKPHDPAVARRLLCDVGVNAAILDAALDDPTTHDDVRADHQRVVAAGGYGVPTLFLDGQCLFGPVLVDPPAGPAALNLWSVVTGMAGLPHVYELQRPKSPADVELIAQQLRPYLDGRDWVSINRGEIVDIDRLAGRS

>523|CORE_REP|Org112_Gene3043#

MPKKYGVKEKDQVVAHILNLLLTGKLRSGDRVDRNEIAHGLGVSRVPIQEALVQLEHDGIVSTRYHRGAFIERFDVATILEHHELDGLLNGIASARAAANPTPRILGQLDAVMRSLRNSKESRAFAECVWEYRRTVNDEYAGPRLHATIRASQNLIPRVFWMTYQNSRDDVLPFYEEENAAIHRREPEAARAACIGRSELMAQTMLAELFRRRVLVPPEGACPGPFGAPIPGFARSYQPSSPVP

>524|CORE_REP|Org59_Gene138#

MTAVAAGALVVETDSFRLRLLDGLVASIGERGYRATTVSDIVRHARTSKRTFYDRFTSKEQCFLELLLADNETLGNSIRAAVDPNADWHDQIRQAVEAYVTHIESRPAVTLSWIREFPSLGAAAYPVQRRGMEQLTSLLIELXASXGFRRAXLPPLNVPLAVILLGGLRELXALTVEDXQPIRNIVRAGGGCVNRAARSPQLGTHAPGRAPARVAARRSNGPRRRPAPPRNRWPARHSRPAGRA

>525|CORE_REP|Org46_Gene3286#

MSPRRWLRAVAVIGATAMLLASSCTWQLSLFITDGVPPPPGDPVPPVDTHAGGRPADQLREWAEKRAAALGIPVIALEAYAYAARVAEVENPKCHLAWTTLAGIGRVESHHGTYRGATIAPNGDVSPPIRGVRLDGTGGTLRIVDRDGGGLDGDAAVERAMGPMQFISETWRLYGVAARNDGIANVDNIDDAALSAAGYLCWRGKDLATPRGWITALRAYNNSVIYARAVRDWATAYAAGHPL

>526|CORE_REP|Org47_Gene1050#

MRLARRARNILRRNGIEVSRYFAELDWERNFLRQLQSHRVSAVLDVGANSGQYARGLRGAGFAGRIVSFEPLPGPFAVLQRSASTDPLWECRRCALGDVDGTISINVAGNEGASSSVLPMLKRHQDAFPPANYVGAQRVPIHRLDSVAADVLRPNDIAFLKIDVQGFEKQVIAGGDSTVHDRCVGMQLELSFQPLYEGGMLIREALDLVDSLGFTLSGLQPGFTDPRNGRMLQADGIFFRGSD

>527|CORE_REP|Org83_Gene3136#

MPNFWALPPEINSTRIYLGPGSGPILAAAQGWNALASELEKTKVGLQSALDTLLESYRGQSSQALIQQTLPYVQWLTTTAEHAHKTAIQLTAAANAYEQARAAMVPPAMVRANRVQTTVLKAINWFGQFSTRIADKEADYEQMWFQDALVMENYWEAVQEAIQSTSHFEDPPEMADDYDEAWMLNTVFDYHNENAKEEVIHLVPDVNKERGPIELVTKVDKEGTIRLVYDGEPTFSYKEHPKF

>528|CORE_REP|Org147_Gene1183#

MPGGVCSGRPWGRPWWHPGLVGLLIRLAELLVVMLPLIGVLYVGIKALSSFTRRLGEASGDLASDSPAMPRPTTVENDAARWRAITRAVEAHERTDARWLEYELDAAKLLDFPVMTDMRDPLTTAFHKAKLQADFHKPLRAEDLLDDPDAAGHYLDAVRDYVTAFDTAEAEAMRRRRTGFSREEQQRLARAQSLLRVASDAGATAQERERAYRLARTELDGLIVLPDRTRAGIERGIAGELDD

>529|CORE_REP|Org6_Gene2988#

MLTQTLTPAIYRTTISHCRQVPVHHSFAYRSYSWYVDVDNLPQLPWWLRPFARFHADDHFADPFSCPPHSSLRDRLDAFFAARGLAVPDGRITALLQARVLGYVFNPLSIFWCHDRDGQLRHVIAEVHNTYGGRHAYLLPPADLPVVTAKNFYVSPFHQLAGYYLIRAPRPDRELDVTVTLHRDRRQVCPEFTATLRGQRRPATTRQIAMMQIISPLAPMVVAARIRIQGIRLWLRRVPVVPR

>530|CORE_REP|Org128_Gene555#

MNLGQTLVGIATWPARAGLAAADTGLNMAGAAVDMAKQALGDAGGASGSTSMANMLGIDDTIARANRLARLLDDDMPLGRAIAPNGPMDRMLRPGGVVDLLTQPGGLLDRLTAEGGAMQRALQPGGLADQLLAEDGLIERVLSEDGLADRLLAEGGLIDKITAKDGPLEQLADVADTLARLTPGMEALEPAIATLQDAVIALTMVVNPLSSIAERIPLPGRRPARRSSSRSVRSQRVVDSE

>531|CORE_REP|Org22_Gene2685#

MNSHCSHTFITDNRSPRARRGHAMSTLHKVKAYFGMAPMEDYDDEYYDDRAPSRGYARPRFDDDYGRYDGRDYDDARSDSRGDLRGEPADYPPPGYRGGYADEPRFRPREFDRAEMTRPRFGSWLRNSTRGALAMDPRRMAMMFEDGHPLSKITTLRPKDYSEARTIGERFRDGSPVIMDLVSMDNADAKRLVDFAAGLAFALRGSFDKVATKVFLLSPADVDVSPEERRRIAETGFYAYQ

>532|CORE_REP|Org118_Gene1274#

MGMTPRRKRRGGAVQITRPTGRPRTPTTQTTKRPRWVVGGTTILTFVALLYLVELIDQLSGSRLDVNGIRPLKTDGLWGVIFAPLLHANWHHLMANTIPLLVLGFLMTLAGLSRFVWATAIIWILGGLGTWLIGNVGSSCGPTDHIGASGLIFGWLAFLLVFGLFVRKGWDIVSWVGRVLFVYGGILLGAMPVLGQCGGVSWQGHLSGAVAGVVAAYLLSAPERKARALKRAGARSGHPKL

>533|CORE_REP|Org132_Gene1903#

MIRSRQPLLDALGVDLPDELLSLALTHRSYAYENGGLPTNERLEFLGDAVLGLTITDALFHRHPDRSEGDLAKLRASVVNTQALADVARRLCAEGLGVHVLLGRGEANTGGADKSSILADGMESLLGAIYLQHGMEKAREVILRLFGPLLDAAPTLGAGLDWKTSLQELTAARGLGAPSYLVTSTGPDHDKEFTAVVVVMDSEYGSGVGRSKKEAEQKAAAAAWKALEVLDNAMPGKTSA

>534|CORE_REP|Org20_Gene445#

MPKPADHRNHAAVSTSVLSALFLGAGAALLSACSSPQHASTVPGTTPSIWTGSPAPSGLSGHDEESPGAQSLTSTLTAPDGTKVATAKFEFANGYATVTIATTGVGKLTPGFHGLHIHQVGKCEPNSVAPTGGAPGNFLSAGGHYHVPGHTGTPASGDLASLQVRGDGSAMLVTTTDAFTMDDLLSGAKTAIIIHAGADNFANIPPERYVQVNGTPGPDETTLTTGDAGKRVACGVIGSG

>535|CORE_REP|Org59_Gene2298#

MDVAFGVRLAANHGGLFGGGPMAHGKKRRGHRSSGVAAGVTGPASCLHSVHSHRLASGVETHPPNRHESASIWNRRRXLLLNSTYEPLTALSMRRXXVMVICGKADVVHEDPSGPVIHSATRSILVPSVIQLRSYVRVPYRARVPMTRAALMHRDRFCCAYCGGKADTVDHVVPXSRGGAHSWENCVACCSPCNHRKGDRLLTELGWALRRAPLPPTGPHWRLLSSGQGAGPVLGAIPR

>536|CORE_REP|Org129_Gene1526#

MRADMSVTSMLDREVYVYAEVDKLIGLPAGTAKRWINGYERGGKDHPPILRVTPGATPWVTWGEFVETRMLAEYRDRRKVPIVRQRAAIEELRARFNLRYPLAHLRPFLSTHERDLTMGGEEIGLPDAEVTIRTGQALLGDARWLASIATPGRDEVGEAVIVELPVDKAFPEIVINPSRYSGQPTFVGRRVSPVTIAQMVDGGEEREDLAADYGLSLKQIQDAIDYTKKYRLARLVAA

>537|CORE_REP|Org21_Gene1617#

MAPTSSSVASELLMPWPSAAASGVVGWRTTATASQRYHRPMSDTPFAEPYPEQRPPWGVPPPGWDGSSRPAPSTTPRSPGRWSLVAALALAVVSLGVGIVGWFHRQPHDKPSPAPSAPTFTSQQISDAKENVCAAHRIVRQAAVLNTNQANPVPGDPTGDLAVAANARLALYSGGDYLLRRLTAEPATPAELRDAVRSLANALQELAVNYLAGAPDSVVTPLRLALERDTRAVDPLCV

>538|CORE_REP|Org1_Gene3733#

MDGVDRSRGWTHPYQPPFRGPSHDCYIGFNAVQVHVVDHPLAAARLTTLRDERTDNAGFRAALRELTLLLIYEATRDAPCEPVPIRTPLAETVGSRLTKPPLLVPVLRAGLGMVDEAHAALPEAHVGFVGVARDEQTHQPVPYLDSLPDDLTDVPVMVLDPMVATGGSMTHTLGLLISRGAADITVLCVVAAPEGIAALQKAAPNVRLFTAAIDEGLNEVAYIVPGLGDAGDRQFGPR

>539|CORE_REP|Org145_Gene3027#

MGKQLAALAALVGACMLAAGCTNVVDGTAVAADKSGPLHQDPIPVSALEGLLLDLSQINAALGATSMKVWFNAKAMWDWSKSVADKNCLAIDGPAQEKVYAGTGWTAMRGQRLDDSIDDSKKRDHYAIQAVVGFPTAHDAEEFYSSSVQSWSSCSNRRFVEVTPGQDDAAWTVADVVNDNGMLSSSQVQEGGDGWTCQRALTARNNVTIDIVTCAYSQPDLVAIGIANQIAAKVAKQ

>540|CORE_REP|Org117_Gene1922#

MNRRTLLWLSAIAALALVVAYQTLGSSAGRHADEFAARAGVPTVQPGADVLAGIAVLPKRIHRYDYRRSAFGHPWDDRNDAPGGHNGCDTRDDILDRDLVDKTYVSIKRCPNAVATGTLRDPYTNTTVAFQRGASVGQSVQIDHIVPLSYAWDMGAYRWPNSERMRFANDPANLLAVQGQANQDKGDSPPAQWMPPNKAFACQYAMQFIAVLRGYSLPVDQPSSDVLRQAAATCPTG

>541|CORE_REP|Org19_Gene382#

MSKDRLYFRQLLSGRDFAVGDMFATQMRNFAYLIGDRTTGDCVVVDPAYAAGDLLDALESDDMQLSGVLVTHHHPDHVGGSMMGFQLPGLAELLERASVPVHVNTHEALWVSRVTGIPVGDLITHEHGDKVSVGDIDIELLHTPGHTPGSQCFLLDGRLVAGDTLFLEGCGRTDFPGGDSDEMYRSLRQLAELPGDPTVFPGHWYSAEPSASLSEVKRSNYVYRPASLDQWRMLMGG

>542|CORE_REP|Org16_Gene1743#

MTRLVPALRLELTLQVRQKFLHAAVFSGLIWLAVLLPMPVSLRPVAEPYVLVGDIAIIGFFFVGGTVFFEKQERTIGAIVSTPLRFWEYLAAKLTVLLAISLFVAVVVATIVHGLGYHLLPLVAGIVLGTLLMLLVGFSSSLPFASVTDWFLAAVIPLAIMLAPPVVHYSGLWPNPVLYLIPTQGPLLLLGAAFDQVSLAPWQVGYAVVYPIVCAAGLCRAAKALFGRYVVQRSGVL

>543|CORE_REP|Org119_Gene2820#

MFARQEPVPERGDAARNRALLLEAARRLIARSGADAITMDDVAAAAGVGKGTLFRRFGSRAGLMMVLLDEDERASQQAFLFGPPPLGPDAPPLDRLIAFGRERMRFVHAHHQLLSEANRDPQTRHSAALSVLRTHLRVLLASAPNHRRPGCPDRCPASAARRRLCRAPTQRRRPYPANPGRRMGEPGAKTVRTMIDHYADSSTAMDPARRPRPVFGVGRTAPPPRTGRFAGHRRRQR

>544|CORE_REP|Org20_Gene529#

MTPTGDTKPKLLFYEPGASWYWVLTGPLAAVSVLLLEISSGAGVGLITPAIFLVMVSAFVALQVKAARIHTSVELTHDALRQGTETIRLAEIVKIYPEADGRETSGEEPAKWQSARTLGELVGVPRGRVGIGLKLTGGRTAQAWARRHQQLRAALTPLVQGGSGPWILMSPTSTVTTPGQRGDRPLPGRGRTVPGLCRACRICGELVADPSLPVAVAPVIDGQPVTLSVVYHPHLRR

>545|CORE_REP|Org119_Gene3384#

MQTAHRRFAAAFAAVLLAVVCLPANTAAADDKLPLGGGAGIVVNGDTMCTLTTIGHDKNGDLIGFTSAHCGGPGAQIAAEGAENAGPVGIMVAGNDGLDYAVIKFDPAKVTPVAVFNGFAINGIGPDPSFGQIACKQGRTTGNSCGVTWGPGESPGTLVMQVCGGPGDSGAPVTVDNLLVGMIHGAFSDNLPSCITKYIPLHTPAVVMSINADLADINAKNRPGRGIRPGTGLSWLG

>546|CORE_REP|Org146_Gene2041#

MSPPNQDAQEGRPDSPTAEVVDVRRGMFGVSGTGDTSGYGRLVRQVVLPGSSPRPYGGYFDDIVDRLAEALRHERVEFEDAVEKVVVYRDELTLHVRRDLLPRVAQRLRDEPELRFELCLGVSGVHYPHETGRELHAVYPLQSITHNRRLRLEVSAPDSDPHIPSLFAIYPTNDWHERETYDFFGIIFDGHPALTRIEMPDDWQGHPQRKDYPLGGIPVEYKGAQIPPPDERRGYN

>547|CORE_REP|Org28_Gene2905#

MNDSNDTSVAGGAAGADSRVLSADSALTERQRTILDVIRASVTSRGYPPSIREIGDAVGLTSTSSVAHQLRTLERKGYLRRDPNRPRAVNVRGADDAALPPVTEVAGSDALPEPTFVPVLGRIAAGGPILAEEAVEDVFPLPRELVGEGTLFLLKVIGDSMVEAAICDGDWVVVRQQNVADNGDIVAAMIDGEATVKTFKRAGGQVWLMPHNPAFDPIPGNDATVLGKVVTVIRKV

>548|CORE_REP|Org137_Gene2206#

MAKRTPVRKACTVLAVLAATLLLGACGGPTQPRSITLTFIRNAQSQANADGIIDTDMPGSGLSADGKAEAQQVAHQVSRRDVDSIYSSPMAADQQTAGPLAGELGKQVEILPGLQAINAGWFNGKPESMANSTYMLAPADWLAGDVHNTIPGSISGTEFNSQFSAAVRKIYDSGHNTPVVFSQGVAIMIWTLMNARNSRDSLLTTHPLPNIGRVVITGNPVTGWRLVEWDGIRNFT

>549|CORE_REP|Org128_Gene1468#

MKLLGHRKSHGHQRADASPDAGSKDGCRPDSGRTSGSDTSRGSQTTGPKGRPTPKRNQSRRHTKKGPVAPAPMTAAQARARRKSLAGPKLSREERRAEKAANRARMTERRERMMAGEEAYLLPRDRGPVRRYVRDVVDSRRNLLGLFMPSALTLLFVMFAVPQVQFYLSPAMLILLALMTIDAIILGRKVGRLVDTKFPSNTESRWRLGLYAAGRASQIRRLRAPRPQVERGGDVG

>550|CORE_REP|Org107_Gene1699#

MPGSAGWRKVFGGTGGATGALPRHGRGSIVYARSTTIEAQPLSVDIGIAHVRDVVMPALQEIDGCVGVSLLVDRQSGRCIATSAWETLEAMRASVERVAPIRDRAALMFAGSARVEEWDIALLHRDHPSHEGACVRATWLKVVPDQLGRSLEFYRTSVLPELESLDGFCSASLMVDHPACRRAVSCSTFDSMDAMARNRDRASELRSRRVRELGAEVLDVAEFELAIAHLRVPELV

>551|CORE_REP|Org2_Gene911#

MSVSGIGESTLADVDAFCAEMDARSVPVSLLVAPRMRDDYRLDRDPRTVDWLTGRRAAGDALVLHGYDEAATKRRRGEFAMLRAHEANLRLMAADRVLEHLGLRTRLFAAPGWLVSPGVRTALPANGFRLLADLHGITDLVRLTTVRARVLGIGEGFLAEPWWCRMVVMSAERIARRGASSGLRWPPVICASPVRCRRCSMPSTWRCCRGAHRWCTGGEPMRRYSTRPDRAPDRWR

>552|CORE_REP|Org1_Gene3852#

MTSLLFENMFEVIRHPSLESVKVMRGCLVQSRKTTSVLAAALLFCGLLGPGTAPPATGGGPACRPAELFATDNTTDGFELPAVATIALTGTVVTGSTLVDGVFWSNERQQIGYERSREFHLCVVDAPTLHNAAEALHRQFNQEAVLTFDYLPQNAPEADAILITVPDIGIARFRDAFASDLAAHHRLRGGSVTTADHTLILVAGNGDLDVARRLVEEAGGDWNATTIAHGRREFVN

>553|CORE_REP|Org1_Gene4082#

MPNTNPVAAWKALKEGNERFVAGRPQHPSQSVDHRAGLAAGQKPTAVIFGCADSRVAAEIIFDQGLGDMFVVRTAGHVIDSAVLGSIEYAVTVLNVPLIVVLGHDSCGAVNAALAAINDGTLPGGYVRDVVERVAPSVLLGRRDGLSRVDEFEQRHVHETVAILMARSSAISERIAGAAWRSWASPINSTMGGLYCATTSATSARRSELPPDNRPPLTRRRPAKPATRRGGSSAFA

>554|CORE_REP|Org40_Gene475#

MSKTSKAYRAAAAKVDRTNLYTPLQAAKLAKETSSTKQDATVEVAIRLGVDPRKADQMVRGTVNLPHGTGKTARVAVFAVGEKADAAVAAGADVVGSDDLIERIQGGWLEFDAAIATPDQMAKVGRIARVLGPRGLMPNPKTGTVTADVAKAVADIKGGKINFRVDKQANLHFVIGKASFDEKLLAENYGAAIDEVLRLKPSSSKGRYLKKITVSTTTGPGIPVDPSITRNFAGE

>555|CORE_REP|Org59_Gene769#

MFDLRITTPRLQLQLPTEELCDQLIDTILEGVHDPDRMPFSVPWTRASREXLPFNTLSHLWQQLAGXKRDDWSLPLAVLVDGRAVGVQALSSKDFPITRQVDSGSWLGLRYQGHGYGTEMRAAVLYFAFAELEAQVATSRSFVDNPASIAXXRRNGYRDNGLDRVAREGAMAEALLFRLTRDDWQRHRTVEVRVDGFDRCRPLFGPLEPPRYXPATQKALPSGSASTTQRKLSPT

>556|CORE_REP|Org19_Gene2602#

MEGDAGAGQLNPADANKSSSTEVKAADSAESDAGADQTGPQVKAADSAESDAGELGEDACPEQALVERRPSRLRRGWLVGIAATLLALAGGLGAAGYFALRSHQESQSIAREDLAAIEAAKDCVAATQAPDAGAMSASMQKIIECGTGDFGAQASLYTSMLVEAYQAASVHVQVTDMRAAVERNNNDGSVDVLVALRVKVSNTDSDAHEVGYRLRVRMALDEGRYKIAKLDQVTK

>557|CORE_REP|Org20_Gene2537#

MRGAGGRGRGGDHALVLARGVRWKNSAGIRWPKPSATSPPTSAASPTAKHRPRGSPVNRWHQLFDRPAGLGKSTILRWPRRSGSACSTPTTRSSSATGRSIADIFATDGEQEFRRIEEDVVRAALADHDGVLSLGGGAVTSPGVRAALAGHTALMKISAAEGVRRTGGNTVRPLLAGPDRAEKYRALMAKRARCTGAWTCGGHQSPQPRGGGPPYPVAAAGSQPQRGGQAESAHP

>558|CORE_REP|Org24_Gene2187#

MTILILTDNVHAHALAVDLQARHGDMDVYQSPIGQLPGVPRCDVAERVAEIVERYDLVLSFHCKQRFPAALIDGVRCVNVHPGFNPYNRGWFPQVFSIIDGQKVGVTIHEIDDQLDHGPIIAQRECAIESWDSSGSVYARLMDIERELVLEHFDAIRDGSYTAKSPATEGNLNLKKDFEQLRRLDLNERGTFGHFLNRLRALTHDDFRNAWFVDASGRKVFVRVVLEPEKPAEA

>559|CORE_REP|Org44_Gene1136#

MACPEWEISRSKRTRKPVLRPRHSVSTLTNRFLAEFCHRYGIGVPTRLARGATVPTRRLQDINDQPVDVPAATGRTHLQFRRFAACPICHLHLRSFANRHQEVADSGITEVVFFHSAADALRGYQSLLPFAVIADPDRVQYREFGVEKSLGAITHPRALWAAVRGSAAMLHRNDPERAGVGFGDGTTHLGLPADFLLDADGTVAAVHYGRHADDQWSVDQLIDINRSLGGKGTQ

>560|CORE_REP|Org118_Gene1703#

MQTLTVADFALRLAVGVGCGAIIGLERQWRARMAGLRTNALVATGATLFVLYAVATEDSSPTRVASYVVSGIGFLGGGVILREGFNVRGLNTAATLWCSAAVGVLAASGHLVFTLIGTGTIVAVHLLGRPLGRLVDRDNAVEDEGLQPYQVRVICRPKAETYVRAHIVQRTSSNDITLRGIRTGPAGDDNITLTAHLLMVGHTPAKLERLVAELSLQPGVYAVHWYAGEHAQAE

>561|CORE_REP|Org59_Gene1734#

MEKVIAVLMRPEPDDDWCARQRAQVADALLGLGVAGLSINVRDSTVRDSLMXLTTLYPPVAAVVSLWTQQCYGEQVAAALRLLAQECDELGAYLVTESVPLTFPSLVESGSRTPGLANIALLRRPDGLDQATWLTRWQRDHTQVAIEAQATFGYTQNWVVRALTPEAPGIAGIVEELFPVAATTDLKAFFGAADDNDLRNRISRMVASTSAFGANQNIDXVPXSRYVFRTPFKD

>562|CORE_REP|Org3_Gene1957#

MRQHSGIGVLDKAVGVLHAVAESPCGLAELCDRTDLPRATAYRLAAALEVHRLLGRGQDGHWRLGPAITELATHVDDPLLVACAAVLPQLRDATGESVQVYRREGTSRVCVAALEPAAGLRDTVPVGARLPMTAGSGAKVLLAHTDAATQAAVLPKAVFSARALAEVCRRGWAQSVAEREPGVASVSAPVRDGRGVVIAAISVSGPIDRMGRRPGVRWAADLLSAADALTRRL

>563|CORE_REP|Org14_Gene2362#

MCRHLGWLGAQVAVSSLVLDPPQGLRVQSYAPRRQKHGLMNADGWGVGFFDGAIPRRWRSPAPLWGDTSFHSVAPALRSHCILAAVRSATVGMPIEVSATPPFTDGHWLLAHNGVVDRAVLPAGPAAESVCDSAILAATIFAHGLDALGDTIVKVGAADPNARLNILAANGSRLIATTWGDTLSILRRADGVVLASEPYDDDSGWGDVPDRHLVEVTQKGVTLTALDRAKGPR

>564|CORE_REP|Org20_Gene678#

MARMTSQTGVRDELLHAGVRLLDDHGPDALQTRKVAAAAGTSTMAVYTHFGGMRGLIAAIAEEGLRQFDVALTVPQTADPVADLLAIGTAPALRHRAPAHVPANVRQHQRTRHQRASARRVDPQGCRDRTPAPQFRACGASGAPVPAGRPVRDRAWSRRRHGNSCHRGAVLVTDPRLRDARAGRLHGDRGAAVEPVLAAMTVNLLVALGDSPERAQCSLRAEQTQKNTLGRAT

>565|CORE_REP|Org133_Gene3777#

MHLMIPAEYISNVIYEGPRADSLYAADQRLRQLADSVRTTAESLNTTLDELHENWKGSSSEWMADAALRYLDWLSKHSRQILRTARVIESLVMAYEETLLRVVPPATIANNREEVRRLIASNVAGGKHSSNRRPRGTIRAVPGRKYPSNGPLSKLDPICAIEAAPMAGAAADPQERVGPRGRRGLAGQQQCRGRPGPSLRCSHDTPRFQMNQAFHTMVNMLLTCFACQEKPR

>567|CORE_REP|Org59_Gene161#

MPSDTSPNGLSRREELLAVATKLFAARGYHGTRMDDVADVIGLNKATVYHYYASKSLILFDIYRQAAEGTLAAVHDDPSWTAREALYQYTVRLLTAIASNPERAAVYFQEQPYITEWFTSEQVAEVREKEQQVYEHVHGLIDRGIASGEFYECDSHVVALGYIGMTLGSYRWLRPSGRRTAKEIAAZLQHGTAARADPRRIDPQPVSAWNSEGNVNLTRSVESISLRTRGRH

>568|CORE_REP|Org54_Gene2857#

MPSPSSADQVADSPRPRLPADHPGVNELFALLAYGEVAAFYRLTDEARMAPDLRGRISMASMAAAEMGHYELLRNALERRGVDVVSAMSKYTSALENYHRLTTPSTWLEALVKTYVADALAADLYLEIADGLPDEVADVVRAALSETGHSQFVVAEVRAAVTASGKQRSRLALWSRRLLGEAITQAQLVLADHDELVDLVVSGSGGLSQLGAFFDRLQQTHDQRMRELGLS

>569|CORE_REP|Org30_Gene1769#

MARLDYDALNATLRYLMFSVFSVSPGALGDQRDAIIDDASTFFKQQEERGVVVRGLYDVAGLRADADFMVWTHAERVEALQATYADFRRTTTLGRACTPVWSGVGLHRPAEFNKSHIPAFLAGEEPGAYICVYPFVRSYEWYLLPDEERRRMLAEHGMAARGYKDVRANTVPAFALGDYEWILAFEAPELDRIVDLMRELRATDARRHTRAETPFFTGPRVPVEQLVHSLP

>570|CORE_REP|Org59_Gene613#

MSDPVSYTRKDSIAVISMDDGKVNALGPAMQQALNAAIDNADRDDVGALVITGNGRVFSGGFDLKILTSGEVQPAIDMLRGGFELAYRLLSYPKPVVMACTGHAIAMGAFLLSCGDHRVAAHAYNIQANEVAIGMTIPYAALEIMKLRLTRSAYQQATGLAKTFFGETALAAGFIDEIAXXEVVVSRAEEAAREFAGLNQHAHAATKLRSRADALTAIRAGIDGIAAEFGL

>571|CORE_REP|Org96_Gene319#

MARRPRPDGPQHLLALVRSAVPPVHPAGRPFIAAGLAIAAVGHRYRWLRGTGLLAAAACAGFFRHPQRVPPTRPAAIVAPADGVICAIDSAAPPAELSMGDTPLPRVSIFLSILDAHVQRAPVSGEVIAVQHRPGRFGSADLPEASDDNERTSVRIRMPNGAEVVAVQIAGLVARRIVCDAHVGDKLAIGDTYGLIRFGSRLDTYLPAGAEPIVNVGQRAVAGETVLAECR

>572|CORE_REP|Org19_Gene1227#

MPGPHSPNPGVGTNGPAPYPEPSSHEPQALDYPHDLGAAEPAFAPGPADDAALPPAAYPGVPPQVSYPKRRHKRLLIGIVVALALVSAMTAAIIYGVRTNGANTAGTFSEGPAKTAIQGYLNALENRDVDTIVRNALCGIHDGVRDKRSDQALAKLSSDAFRKQFSQVEVTSIDKIVYWSQYQAQVLFTMQVTPAAGGPPRGQVQGIAQLLFQRGQVLVCSYVLRTAGSY

>574|CORE_REP|Org31_Gene75#

MITRYKPESGFVARSGGPDRKRPHDWIVWHFTHADNLPGIITAGRLLADSAVTPTTEVAYNPVKELRRHKVVAPDSRYPASMASDHVPFYIAARSPMLYVVCKGHSGYSGGAGPLVHLGVALGDIIDADLTWCASDGNAAASYTKFSRQVDTLGTFVDFDLLCQRQWHNTDDDPNRQSRRAAEILVYGHVPFELVSYVCCYNTETMTRVRTLLDPVGGVRKYVIKPGMYY

>575|CORE_REP|Org125_Gene1079#

MKRSMKSGSFAIGLAMMLAPMVAAPGLAAADPATRPVDYQQITDVVIARGLSQRGVPFSWAGGGISGPTRGTGTGINTVGFDASGLIQYAYAGAGLKLPRSSGQMYKVGQKVLPQQARKGDLIFYGPEGTQSVALYLGKGQMLEVGDVVQVSPVRTNGMTPYLVRVLGTQPTPVQQAPVQPAPVQQAPVQQAPVQQAPVQQAPVQQAPVQQAPVQQAPVQPPPFGTARSR

>576|CORE_REP|Org56_Gene158#

MRNAWRLVVFDVLAPLATIAALAAIGVLLGWPLWWVSTCSVLVLLVVEGVAINFWLLRRDSVTVGTDDDAPGLRLAVVFLCAAAISAAVVTGYLRWTTPDRDFNRDSREVVHLATGMAETVASFSPSAPAAAVDRAAAMMVPEHAGGFKEQYAKSSADLARRGVTAQAATLAAGVEAIGPSAASVAVILRVSQSIPGQPTSQAARALRVTLTKRGSGWLVLDVTPINAR

>577|CORE_REP|Org18_Gene1295#

MRIKIFMLVTAVVLLCCSGVATAAPKTYCEELKGTDTGQACQIQMSDPAYNINISLPSYYPDQKSLENYIAQTRDKFLSAATSSTPREAPYELNITSATYQSAIPPRGTQAVVLKVYQNAGGTHPTTTYKAFDWDQAYRKPITYDTLWQADTDPLPVVFPIVQGELSKQTGQQVSIAPNAGLDPVNYQNFAVTNDGVIFFFNPGELLPEAAGPTQVLVPRSAIDSMLA

>578|CORE_REP|Org15_Gene972#

MACLGRPGCRGWAGASLVLVVVLALAACTESVAGRAMRATDRSSGLPTSAKPARARDLLLQDGDRAPFGQVTQSRVGDSYFTSAVPPECSAALLFKGSPLRPDGSSDHAEAAYNVTGPLPYAESVDVYTNVLNVHDVVWNGFRDVSHCRGDAVGVSRAGRSTPMRLRYFATLSDGVLVWTMSNPRWTCDYGLAVVPHAVLVLSACGFKPGFPMAEWASKRRAQLDSQV

>579|CORE_REP|Org147_Gene3012#

MDTMRQRILVVDDDASLAEMLTIVLRGEGFDTAVIGDGTQALTAVRELRPDLVLLDLMLPGMNGIDVCRVLRADSGVPIVMLTAKTDTVDVVLGLESGADDYIMKPFKPKELVARVRARLRRNDDEPAEMLSIADVEIDVPAHKVTRNGEQISLTPLEFDLLVALARKPRQVFTRDVLLEQVWGYRHPADTRLVNVHVQRLRAKVEKDPENPTVVLTVRGVGYKAGPP

>580|CORE_REP|Org7_Gene631#

MPEAKRPESKRRSPASRPGKAGDSVRGGRATKPSAKPSTPAPHASRKTTRTPHEHIVEPIKRAITESVEKRSEQRLGFTARRAAILAAVVCVLTLTIARPVRTYFAQRAEMEQLAATEAMLRRQIADLEEQQVKLADPAYIAAQARERLGFVMPGDIPFQVQLPSTPLAPPQPGSDAATATNNEPWYTALWHTIADDPHLPPAAPPAPEPGRPGPLPPASPNPEQPGG

>581|CORE_REP|Org108_Gene432#

MKHFTAAVATVALSLALAGCSFNIKTDSAPTTSPTTTSPTTSTTTTSATTSAQAAGPNYTIADYIRDNHIQETPVHHGDPGSPTIDLPVPDDWRLLPESSRAPYGGIVYTQPADPNDPPTIVAILSKLTGDIDPAKVLQFAPGELKNLPGFQGSGDGSAATLGGFSAWQLGGSYSKNGKLRTVAQKTVVIPSQGAVFVLQLNADALDDETMTLMDAANVIDEQTTITP

>582|CORE_REP|Org119_Gene1217#

MRLVIAQCTVDYIGRLTAHLPSARRLLLFKADGSVSVHADDRAYKPLNWMSPPCWLTEESGGQAPVWVVENKAGEQLRITIEGIEHDSSHELGVDPGLVKDGVEAHLQALLAEHIQLLGEGYTLVRREYMTAIGPVDLLCRDERGGSVAVEIKRRGEIDGVEQLTRYLELLNRDSVLAPVKGVFAAQQIKPQARILATDRGIRCLTLDYDTMRGTGIAASTGCSELRD

>583|CORE_REP|Org59_Gene3442#

MDEILARAGIFQGVEPSAIAALTKQLQPVDFPRGHTVFAEGEPGDRLYIIISGKVKIGRRAPDGRENLLTIMGPSDMFGELSIFDPGPRTSSATTITEVRAVSMDRDALRSWIADRPEISEQLLRALDIAXXXLRRTNNNLADLIFTDVPGRVAKQLLQLAQRFGTQEGGALRVTHDLTQEEIAQLVGASRDEPVNKALADFAHRGWIRLEGKSVLISDSERLARRAR

>584|CORE_REP|Org59_Gene2969#

MPLVYFDASAFVKLLTTETGSSLASALWDGCDAALSSRLAYPEVRAALAAAARNHDLTESELADAERDWEDFWAATRPVELTATVEQHAGHLARAHALRGADAVHLASALAVGDPGLVVAVWDRRLHTGAHAAGCRXRPRPTRPVIRPLDVSHIGFGDRRPRASAADGSASVVQLPKRGDVKSAEEIMEILEAYDLTGSLRDARSWWGACITRSRVCGRAGARCELVE

>585|CORE_REP|Org16_Gene1961#

MRYLIATAVLVAVVLVGWPAAGAPPSCAGLGGTVQAGQICHVHASGPKYMLDMTFPVDYPDQQALTDYITQNRDGFVNVAQGSPLRDQPYQMDATSEQHSSGQPPQATRSVVLKFFQDLGGAHPSTWYKAFNYNLATSQPITFDTLFVPGTTPLDSIYPIVQRELARQTGFGAAILPSTGLDPAHYQNFAITDDSLIFYFAQGELLPSFVGACQAQVPRSAIPPLAI

>586|CORE_REP|Org33_Gene1438#

MARTRRRGMLAIAMLLMLVPLATGCLRVRASITISPDDLVSGEIIAAAKPKNSKDTGPALDGDVPFSQKVAVSNYDSDGYVGSQAVFSDLTFAELPQLANMNSDAAGVNLSLRRNGNIVILEGRADLTSVSDPDADVELTVAFPAAVTSTNGDRIEPEVVQWKLKPGVVSTMSAQARYTDPNTRSFTGAGIWLGIAAFAAAGVVAVLAWIDRDRSPRLTASGDPPTS

>587|CORE_REP|Org133_Gene366#

MTSVLIVEDEESLADPLAFLLRKEGFEATVVTDGPAALAEFDRAGADIVLLDLMLPGMSGTDVCKQLRARSSVPVIMVTARDSEIDKVVGLELGADDYVTKPYSARELIARIRAVLRRGGDDDSEMSDGVLESGPVRMDVERHVVSVNGDTITLPLKEFDLLEYLMRNSGRVLTRGQLIDRVWGADYVGDTKTLDVHVKRLRSKIEADPANPVHLVTVRGLGYKLEG

>588|CORE_REP|Org59_Gene1159#

MRLAHFEHGPTETEHSDRGSWLSFELALNKMRANSPCPGEARSXRQWHWLAATLLLITTAACSRPGTEEPDCPTKITLPPGATPTTTLDPRCIVRATTTGTADGDAASRWTGTVRIAGFYASICNAVWDGNVSLAGKDELTGKATLILVETSCPGKVVAGELVLKGNVGSDSLXITXAHPELPQRAFDLGAGQGTIRRSGDRAEGTFNSDMGGGTEFFLTWSLTMRN

>589|CORE_REP|Org2_Gene805#

MPHESRVGRRRSTTPHHISDVAIELFAAHGFTDVSVDDIARAAGIARRTLFRYYASKNAIPWGDFSTHLAQLQGLLDNIDSRIQLRDALRAALLAFNTFDESETIRHRKRMRVILQTPELQAYSMTMYAGWREVIAKFVARRSGGKTTDFMPQTVAWTMLGVALSAYEHWLRDESVSLTEALGPRLTSSAPVWTDSTSDGHRTPGPHPAVTGAGVHLLRLPDVPRAT

>590|CORE_REP|Org102_Gene2503#

MAGRRCPQDSVRPLAVAVAVATLAMSAVACGPKSPDFQSILSTSPTTSAVSTTTEVPVPLWKYLESVGVTGEPVAPSSLTDLTVSIPTPPGWAPMKNPNITPNTEMIAKGESYPTAMLMVFKLHRDFDIAEALKHGTADARLSTNFTELDSSTADFNGFPSSMIQGSYDLHGRRLHTWNRIVFPTGAPPAKQRYLVQLTITSLANEAVKHASDIEAIIAGFVVAAK

>591|CORE_REP|Org40_Gene740#

MTLVLVIDDEPQILRALRINLTVRGYQVITASTGAGALRAAAEHPPDVVILDLGLPDMSGIDVLGGLRGWLTAPVIVLSARTDSSDKVQALDAGADDYVTKPFGMDEFLARLRAAVRRNTAAAELEQPVIETDSFTVDLAGKKVIKDGAEVHLTPTEWGMLEMLARNRGKLVGRGELLKEVWGPAYATETHYLRVYLAQLRRKLEDDPSHPKHLLTESGMGYRFEA

>592|CORE_REP|Org79_Gene2554#

MSSGAGSDATGAGGVHAAGSGDRAVAAAVERAKATAARNIPAFDDLPVPADTANLREGADLNNALLALLPLVGVWRGEGEGRGPDGDYRFGQQIVVSHDGGDYLNWESRSWRLTATGDYQEPGLREAGFWRFVADPYDPSESQAIELLLAHSAGYVELFYGRPRTQSSWELVTDALARSRSGVLVGGAKRLYGIVEGGDLAYVEERVDADGGLVPHLSARLSRFVG

>593|CORE_REP|Org2_Gene1632#

MSPSPTYTPPKLASMPGIDFDALYRGESPGEGLPPITTPPWDTKAPKDNVIGWHTGGWVHGDVLDIGCGLGDNAIYLARNGYQVTGLDISPTALTTAKRRASDAGVDVKFAVGDATKLTGYTGAFDTVIDCGMFHCLDDDGKRSYAASVHRATRPGATLLLSCFSNAMPPDEEWPRSTVSEQTLRDVLGGAGWDIESLEPATVRRELDGTEVEMAFWNVRAQRRGS

>594|CORE_REP|Org59_Gene84#

MNAVESTLRRVAKDLTGLRQRWALVGGFAVSARSEPRFTRDVDIVVAVANDDAAESLVRQLLTQQYHLLASVEQDAARRLAAVRLGATADTAANVVVDLLFASCGIEPEIAEAAEEIEILPDLVAPVATTAHLIAMKLLARDDDRRPQDRSDLRALVDAASPQDIQDARKAIELITLRGFHRDRDLAAEWXRXAAKWXPSQSCAXXXXSXXRXXSXFAEAAPSASR

>595|CORE_REP|Org20_Gene2633#

MINPTRARRMRYRLAAMAGMPEGKLILLNGGSSAGKTSLALAFQDLAAECWMHIGIDLFWFALPPEQLDLARVRPEYYTWDSAVEADGLEWFTVHPGPILDLAMHSRYRAIRAYLDNGMNVIADDVIWTREWLVDALRVFEGCRVWMVGVHVSDEEGARRELERGDRHPGWNRGSARAAHADAEYDFELDTTATPVHELARELHESYQACPYPMAFNRKLRKRFLS

>596|CORE_REP|Org111_Gene1242#

MAVEVLVTGGDTDLGRTMAEGFRNDGHKVTLVGARRGDLEVAAKELDVDAVVCDTTDPTSLTEARGLFPRHLDTIVNVPAPSWDAGDPRAYSVSDTANAWRNALDATVLSVVLTVQSVGDHLRSGGSIVSVVAENPPAGGAESAIKAALSNWIAGQAAVFGTRGITINTVACGRSVQTGYEGLSRTPAPVAAEIARLALFLTTPAARHITGQTLHVSHGALAHFG

>597|CORE_REP|Org59_Gene832#

MSAXEAITLVIDGGRTRSRIANAPGVIAPSLASVVNAESWDSETGDDGFRNRSWRESRMXANDKSLASRASLSSTAEVTTEVQTRASETANDDFGSTHCRPTTHQQVVVPPAGAEATHXGPFADASSPRYVXXGXSVENSQIREPPPLPPVLLEVWPVIAVGALAWLVAAVAAFVVPGLASWRPVTVAGLATGLLGTTIFVWQLAAARRGARGAQAGLETYLDPK

>598|CORE_REP|Org59_Gene45#

MSVVRSIGKKMQRISGPNALAVKGRPTQVYGHTHVRLDCRFMADSEFTAPEVTQLAEGLHRALSKLISMLRRGDPNGAAAGDLTLAQLSILVTLLDQGPIRMTDLAAHERVRTPTTTVAIRRLEKIGLVKRSRDPSDLRAVLVDITPQGRAVHGESLANRRDGSGRAAQPTSPLRLGNPQEGPGATGASRLGRTGQWPCKQLPXSQTSVKVPRYQPIWSPPALAA

>599|CORE_REP|Org19_Gene1426#

MLITGFPAGLLACNCYVLAERPGTDAVIVDPGQGAMGTLRRILDKNRLTPAAVLLTHGHIDHIWSAQKVSDTFGCPTYVHPADRFMLTDPIYGLGPRIAQLVAGAFFREPKQVVELDRDGDKIDLGGISVNIDHTPGHTRGSVVFRVLQATNNDKDIVFTGDTLFERAIGRTDLAGGSGRDLLRSIVDKLLVLDDSTVVLPGHGNSTTIGAERRFNPFLEGLSR

>600|CORE_REP|Org138_Gene92#

MSVYKHAPSRVRLRQTRSTVVKGRSGSLSWRRVRTGDLGLAVWGGREEYRAVKPGTPGIQPKGDMMTVTVVDAGPGRVSRSVEVAAPAAELFAIVADPRRHRELDGSGTVRGNIKVPAKLVVGSKFSTKMKLFGLPYRITSRVTALKPNELVEWSHPLGHRWRWEFESLSPTLTRVTETFDYHAAGAIKNGLKFYEMTGFAKSNAAGIEATLAKLSDQYARGRA

>601|CORE_REP|Org36_Gene866#

MHIGLKIFIWGVLGLVVFGALLFGPAGTFDYWQAWVFLAAFVSTTIGPTIYLARNDPAALQRRMRSGPLAEGRTIQKFIVIGAFLGFFAMMVLSACDHRYGWSSVPAAVCVIGDVLVMTGLGIAMLVVIQNRYAASTVRVEAGQILASDGLYKIVRHPMYAGNVVMMTGIPLALGSYWAMFILVPGTLVLVFRILDEEKLLTQELSGYREYRQLVRYRLVPYVW

>602|CORE_REP|Org121_Gene3217#

MIGRDRAYAVTRRKDIAKQRLVWRLCQRYPRAARRLIRHLNAKQLAAGYPADEHFKPVYNPWDQRLCAVPDADMFKAIRDGRASVVTEAIDTFTENGIRLQSGRELAADISITATGLNLLAFGGINLSVDGVAVDVAEKVAFKGFLLSDVSNFAGPHGRTRAHHLLSAAARSHADPAAAGRRSPLADLKVLREGPVDDDHLRFTTSASASRLTVKRITRSTPWN

>603|CORE_REP|Org59_Gene92#

MSNANFSILVDFAAGGLVLASVLIVWRRDLSPXXVRLLAWQXELRTAXPLXPLLRGIRDNDRALIAVGIAVLALRALVLPWLLARAVGAEAAAQREATPLVNTASSLLITAGLTLTAFAITQPVVNLEPGVTINAVPAAFAVVLIALFVMTTRLHAVSQAAGFLMLDNGIAATAFLLTAGVPLIVELGASLDVLFAVIVIGVLTGRLRRIFGDADLDKLRELRD

>604|CORE_REP|Org78_Gene575#

MTARIGVVTFPGTLDDVDAARAARQVGAEVVSLWHADADLKGVDAVVVPGGFSYGDYLRAGAIARFAPVMDEVVAAADRGMPVLGICNGFQVLCEAGLLPGALTRNVGLHFICRDVWLRVASTSTAWTSRFEPDADLLVPLKSGEGRYVAPEKVLDELEGEGRVVFRYHDNVNGSLRDIAGICSANGRVVGLMPHPEHAIEALTGPSDDGLGLFYSALDAVLTG

>605|CORE_REP|Org2_Gene2896#

MVSGIGGVVERGLWLPDPAHRADLATFVDHALRLDDAAVIRIRARSTGLLSAWVATGFDVLASRVVAGKVRPDDLSVAARSLAHGLATTDASGYVDPGYSMDSAWRGGLPPESGFTYLDDVPARVMLDLAHRGARLAKEHGSSAGPPVSLLDQEVIQVSSADVVVGLPMRCVFALTAMGFLPQSAETISADELIRVRISPAWLRLDARFGSVYRHRGHAALVLR

>607|CORE_REP|Org40_Gene252#

MNSCNRLPCAHEVLAVFAHPDDESFGLGAVLGDFTAQGTRLRGLCFTHGEASTLGRTDRNLGEVRREELAAAAQVLGVDHVQLLAYPDNGLAQIPLNELTQRVVDALAGADLLLVFDDNGVTGHPDHRRATEAALAAASTPGIPVLAWALPQPIADRLNAEFSASFGGRGHGHLDIMIEVDRSRQLAAIGCHFTQSADNPVLWRRLELLGDREYLRWLRRSVP

>608|CORE_REP|Org26_Gene512#

MAAQEQKTLKIDVKTPAGKVDGAIELPAELFDVPANIALMHQVVTAQRAAARQGTHSTKTRGEVSGGGRKPYRQKGTGRARQGSTRAPQFTGGGVVHGPKPRDYSQRTPKKMIAAALRGALSDRARNGRIHAITELVEGQNPSTKSARAFLASLTERKQVLVVIGRSDEAGAKSVRNLPGVHILAPDQLNTYDVLRADDVVFSVEALNAYIAANTTTSEEVSA

>609|CORE_REP|Org2_Gene1155#

MLIARLFVGAVPALARDGRGCVIVEDMAMASKSALRDQLLAARRRVADDVRAAEARMLRGHLERMVTSDSTVCAYVPVGGEPGSIEMLDVLLRRAGRVLLPVARTAGGDLPLPLRWGEYRAGGLARARWGLLEPPEPWLPEAALAQASLVLVPALAVDRQGVRLGRGRGFYDRSLRCRDPHARLVAVVRTVELVDVLPSEPHDVPMTHALTPERGLIALPCGE

>610|CORE_REP|Org140_Gene3844#

MPPRIAGMRLLVIKPEPLARRLLKLAGTTYAAEAGIRIRDKPMPLFQLLVLCMLASKPIGAATAARAARELFCSGLRTPKAVLSAERQTMISAFGRAHYVRYDESSATRLTAIAHRVRDEYSGDLRELAQRTRPDVSAAKRMLKTFNGIGDTGADIFLREVQDVWIWVRPYFDDRATAAAKQLGLPTDPKKLASVAPSSNALLAAALVRVALDDELRLQVTG

>611|CORE_REP|Org82_Gene1074#

MTEPPGFGGPSEPSGAPRTSRTRAVLFVMLGLSATGVLVGGLWAWIAPPIHAVVAITRAGERVHEYLGSESQNFFIAPFMLLGLLSVLAVVASALMWQWREHRGPQMVAGLSIGLTTAAAIAAGVGALVVRLRYGALDFDTVPLSRGDHALTYVTQAPPVFFARRPLQIALTLMWPAGIASLVYALLAAGTARDDLGGYPAVDPSSNARTEALETPQAPVS

>612|CORE_REP|Org33_Gene2766#

MSRRASATCALSATTAVAIMAAPAARADDKRLNDGVVANVYTVQRQAGCTNDVTINPQLQLAAQWHTLDLLNNRHLNDDTGSDGSTPQDRAHAAGFRGKVAETVAINPAVAISGIELINQWYYNPAFFAIMSDCANTQIGVWSENSPDRTVVVAVYGQPDRPSAMPPRGAVTGPPSPVAAQENVPIDPSPDYDASDEIEYGINWLPWILRGVYPPPAMPPQ

>613|CORE_REP|Org33_Gene2178#

MITVNVLYFGAVREACKVAHEKISLESGTTVDGLVDQLQIDYPPLADFRKRVRMAVNESIAPASTILDDGDTVAFIPQVAGGSDVYCRLTDEPLSVDEVLNAISGPSQGGAVIFVGTVRNNNNGHEVTKLYYEAYPAMVHRTLMDIIEECERQADGVRVAVAHRTGELRIGDAAVVIGASAPHRAAAFDAARMCIERLKQDVPIWKKEFALDGVEWVANRP

>614|CORE_REP|Org22_Gene2120#

MRTVYHQRLTELAGRLGEMCSLAGIAMKRATQALLEADIGAAEQVIRDHERIVAMRAQVEKEAFALLALQHPVAGELREIFSAVQIIADTERMGALAVHIAKITRREYPNQVLPEEVRNCFADMAKVAIALGDSARQVLVNRDPQEAAQLHDRDDAMDDLHRHLLSVLIDREWRHGVRVGVETALLGRFFERFADHAVEVGRRVIFMVTGVLPTEDEISTY

>615|CORE_REP|Org118_Gene2822#

MLAVRSYLLRIELADRPGSLGSLAVALGSVGADILSLDVVERGNGYAIDDLVVELPPGAMPDTLITAAEALNGVRVDSVRPHTGLLEAHRELELLDHVAAAEGATARLQVLVNEAPRVLRVSWCTVLRSSGGELHRLAGSPGAPETRANSAPWLPIERAAALDGGADWVPQAWRDMDTTMVAAPLGDTHTAVVLGRPGPEFRPSEVARLGYLAGIVATMLR

>616|CORE_REP|Org19_Gene3573#

MKLLSRVRERSSATTMRDRLVWQSASLLLAYPDDGLAERLHMVDALRAHQTGPAAALLGRTVAELRALAPMAAAAQYVETFDMRRRSTMYLTYWTAGDTRNRGREMLAFATAYRDAGVKPPRTEAPDYLPVVLEFAATVDPEAGRRLLTEHRVPIDVLRGALADAKSPYEYTVAAICETLPAATNQEVRRAQRLAQSGRPAEAVGLQPFTLTVPPKRAEGA

>617|CORE_REP|Org59_Gene1254#

MAPDRADDDAERSDEEEWRLMTKLXVASRNRKKLAELRRVLDGAGLSGXTXLSXGDVSPLPETPETGVTFEDNALAKARDAFSATGLASVADDSGLEVAALGGMPGVLSARWSGRYGDDAANTALLLAQLCDVPDERRGAAFVSACALVSGSGEVVVRGEWPGTIAREPRGDGGFGYDPVFVPYGDDRTAAQLSPAEKDAVSHRGRALALLLPALRSLATG

>618|CORE_REP|Org20_Gene995#

MVGRALVVVVDDRTAHGDEDHSGPLVTELLTEAGFVVDGVVAVSADEVEIRNALNTAVIGGVDLVVSVGGTGVTPRDVTPEATRDILDREIIGIAEVRDVRAVRGNRRRRVVERPAGVSGSTLVVNLAGSRYAVRDGMWTASCGTDHRAVVELGDLNPDRVSGYCDSVLARGPSVGDGVPRPPCLPGESPFVCASRSRISVSSTTWVSPGCSTSPFLRSVL

>619|CORE_REP|Org146_Gene531#

MAEQPAGQAGTTDNRDARGDREGRRRDSGRGSRERDGEKSNYLERVVAINRVSKVVKGGRRFSFTALVIVGDGNGMVGVGYGKAKEVPAAIAKGVEEARKSFFRVPLIGGTITHPVQGEAAAGVVLLRPASPGTGVIAGGAARAVLECAGVHDILAKSLGSDNAINVVHATVAALKLLQRPEEVAARRGLPIEDVAPAGMLKARRKSEALAASVLPDRTI

>620|CORE_REP|Org1_Gene372#

MGRHELARDRRKSSAVLAAVLAPAAVFFATGGDVSTLAARADANPVLGDDAPCCVQIVPVAPLAFSSQISGGEIGTGLAASQFASASRWRIVSRYLPVGVAPEQGLQVKTVLTARSISAAFPEIREIGGVRPDALRWHPNGLALDVMVPNPGTAEGIALGNEIVAFVLKNATRFGMQDVIWRGAYYTPNGARTTGAGHYDHIHITTVGGGYPTGEELYIR

>621|CORE_REP|Org81_Gene1867#

MINVQAKPAAAASLAAIAIAFLAGCSSTKPVSQDTSPKPATSPAAPVTTAAMADPAADLIGRGCAQYAAQNPTGPGSVAGMAQDPVATAASNNPMLSTLTSALSGKLNPDVNLVDTLNGGEYTVFAPTNAAFDKLPAATIDQLKTDAKLLSSILTYHVIAGQASPSRIDGTHQTLQGADLTVIGARDDLMVNNAGLVCGGVHTANATVYMIDTVLMPPAQ

>622|CORE_REP|Org66_Gene938#

MTDDVRDVNTETTDATEVAEIDSAAGEAGDSATEAFDTDSATESTAQKGQRHRDLWRMQVTLKPVPVILILLMLISGGATGWLYLEQYRPDQQTDSGAARAAVAAASDGTIALLSYSPDTLDQDFATARSHLAGDFLSYYDQFTQQIVAPAAKQKSLKTTAKVVRAAVSELHPDSAVVLVFVDQSTTSKDSPNPSMAASSVMVTLAKVDGNWLITKFTPV

>623|CORE_REP|Org20_Gene1001#

MRCGGILPESEPGQPDIGVFAPGLDPDRAGPALRRGRLVMLAGVAALRSNPEDDRAEVVVAAHDLRPGTALTPGDVRLEKRSATTLPDGSQADLDAVVGSTLASPTRRGEVLTDVRLLGSRLAESTAGPDARIVPLHLADSALVDLVRVGDVVDVLAAPVTDSPAALRLLATDAIVVLVSAQQKAQAADSDRVVLVALPARLANTVAGAALGQTVTLTLH

>624|CORE_REP|Org1_Gene4067#

MSSANTNTSSAPDAPPRAVMKVAVLAESELGSEAQRERRKRILDATMAIASKGGYEAVQMRAVADRADVAVGTLYRYFPSKVHLLVSALGREFSRIDAKTDRSAVAGATPFQRLNFMVGKLNRAMQRNPLLTEAMTRAYVFADASAASEVDQVEKLIDSMFARAMANGEPTEDQYHIARVISDVWLSNLLAWLTRRASATDVSKRLDLAVRLLIGDQDSA

>625|CORE_REP|Org66_Gene3172#

MPELETPDDPESIYLARLEDVGEHRPTFTGDIYRLGDGRMVMILQHPCALRHGVDLHPRLLVAPVRPDSLRSNWARAPFGTMPLPKLIDGQDHSADFINLELIDSPTLPTCERIAVLSQSGVNLVMQRWVYHSTRLAVPTHTYSDSTVGPFDEADLIEEWVTDRVDDGADPQAAEHECASWLDERISGRTRRALLSDRQHASSIRREARSHRKSVKLAD

>626|CORE_REP|Org142_Gene165#

MPDGEQSQPPAQEDAEDDSRPDAAEAAAAEPKSSAGPMFSTYGIASTLLGVLSVAAVVLGAMIWSAHRDDSGERTYLTRVMLTAAEWTAVLINMNADNIDASLQRLHDGTVGQLNTDFDAVVQPYRQVVEKLRTHSSGRIEAVAIDTVHRELDTQSGAARPVVTTKLPPFATRTDSVLLVATSVSENAGAKPQTVHWNLRLDVSDVDGKLMISRLESIR

>627|CORE_REP|Org6_Gene3590#

MEFRRAVQTGQNRGRWSGVPLESRHALRRDNLVAAGVQLLGGAGGPALTVRAVCRHAGLTERYFYESFADREHFVRAVYDDVCTRAMATLTSAQTPREAVEQFVELMVDDPVRGRVLLLAPAVEPALTRSGAEWMPNFIELLQRKLSRIVDPVLQKLVATSLIGALTGLFTAYLNGRLGATRKQFIDYCVNMLLSTAATYAPHRERGESEHSIPAGPHN

>628|CORE_REP|Org6_Gene3064#

MSRLAVDSGQVLAEPKSNAEIVFKGRNVEIPDHFRIYVSQKLARLERFDRTIYLFDVELDHERNRRQRKSCQRVEITARGRGPVVRGEACADSFYAALESAVVKLESRLRRGKDRRKVHYGDKTPVSLAEATAVVPAPENGFNTRPAEAHDHDGAVVEREPGRIVRTKEHPAKPMSVDDALYQMELVGHDFFLFYDKDTERPSVVYRRHAYDYGLIRLA

>629|CORE_REP|Org84_Gene537#

MNFVDDPESAVLAAAKDMLRRGLVEGTAGNISARRSDGNVVITPSSVDYAEMLLHDLVLVDAGGAVLHAKDGRSPSTELNLHLACYRAFDDIGSVIHSHPVWATMFAVAHEPIPACIDEFAIYCGGDVRCTEYAASGTPEVGRNAVRALEGRAAALIANHGLVAVGPRPDQVLRVTALVERTAQIVWGARALGGPVPIPEDVCRNFTGVYGYLRANPL

>630|CORE_REP|Org4_Gene2637#

MPPVCGRRCSRTGEIRGYSGSIVRRWKRVETRDGPRFRSSLAPHEAALLKNLAGAMIGLLDDRDSSSPSDELEEITGIKTGHAQRPGDPTLRRLLPDFYRPDDLDDDDPTAVDGSESFNAALRSLHEPEIIDAKRVAAQQLLDTVPDNGGRLELTESDANAWIAAVNDLRLALGVMLEIGPRGPERLPGNHPLAAHFNVYQWLTVLQEYLVLVLMGSR

>631|CORE_REP|Org21_Gene234#

MSRLLALLCAAVCTGCVAVVLAPVSLAVVNPWFANSVGNATQVVSVVGTGGSTAKMDVYQRTAAGWQPLKTGITTHIGSAGMAPEAKSGYPATPMGVYSLDSAFGTAPNPGGGLPYTQVGPNHWWSGDDNSPTFNSMQVCQKSQCPFSTADSENLQIPQYKHSVVMGVNKAKVPGKGSAFFFHTTDGGPTAGCVAIDDATLVQIIRWLRPGAVIAIAK

>632|CORE_REP|Org25_Gene1147#

MSGAKKLIFEQFALVGQALSSGHRLELLDLLVQGERSVDALARASGLTFANASQHLLQLRRAGLVTSRRDGKRVIYALSDPQVWDVVRAVRAVAERNLASVGSLVRQYYTDRDSLEPISRDELQARVAAGSVLVLDVRPAMEYAAGHLPGAVSIPLDELAERLDELPSGIDIVACCRGPYCVYAYDALELLRPNGFSARRLDGGFSEWLAADLPVVRT

>634|CORE_REP|Org118_Gene2777#

MTRIIGGVAGGRRIAVPPRGTRPTTDRVRESLFNIVTARRDLTGLAVLDLYAGSGALGLEALSRGAASVLFVESDQRSAAVIARNIEALGLSGATLRRGAVAAVVAAGTTSPVDLVLADPPYNVDSADVDAILAALGTNGWTREGTVAVVERATTCAPLTWPEGWRRWPQRVYGDTPFGTGRTAFCQRVASSVMTAGDDAVGAVPACGGRSDEEERRR

>635|CORE_REP|Org109_Gene2034#

MVKVFLVDDHEVVRRGLVDLLGADPELDVVGEAGSVAEAMARVPAARPDVAVLDVRLPDGNGIELCRDLLSRMPDLRCLILTSYTSDEAMLDAILAGASGYVVKDIKGMELARAVKDVGAGRSLLDNRAAAALMAKLRGAAEKQDPLSGLTDQERTLLGLLSEGLTNKQIADRMFLAEKTVKNYVSRLLAKLGMERRTQAAVFATELKRSRPPGDGP

>636|CORE_REP|Org43_Gene263#

MTISFSSSNLRDDATSGNGDYRLDKLPETTPSTSVFDRADVTYRQFTELHGQARDTRREAHVVELESKTGERARCAPMHALEQLADYGFAWRDIARVVGVSVPAITKWRKGAGVTGENRLKIARLLALIDMLSDRFIGEPASWLEMPIQAGVGITRMDLLERGRYDLVLALASTHTGDGTVEYVLNETDKDWRETVVDNAFESYTAEDGVISIRPKR

>637|CORE_REP|Org129_Gene2003#

MTSHAADEKQAAPPMRRRGDRHRQAILRAARELLEETPFAELSVRAISLRAGVARSGFYFYFDSKYSVLAQILAEATEELEEASQHFSARQPGESPEQFVNRMIGSVAAVYANNDPVLRACNAARQSDMEIRDILERQFQVLLRETIGVFEAEVKAGTAHPISEDLPTLVRTLAATTALMLTGDALLVGPDSDAARRVRVLEQMWLNALWGGGKAP

>638|CORE_REP|Org111_Gene8#

MTTSAASQASLPRGRRTARPSGDDRELAILATAENLLEDRPLADISVDDLAKGAGISRPTFYFYFPSKEAVLLTLLDRVVNQADMALQTLAENPADTDRENMWRTGINVFFETFGSHKAVTRAGQAARATSVEVAELWSTFMQKWIAYTAAVIDAERDRGAAPRTLPAHELATALNLMNERTLFASFAGEQPSVPEARVLDTLVHIWVTSIYGENR

>640|CORE_REP|Org53_Gene2730#

MIGPARRSTTTRRSTPRADRLAGCWCLPGAICQTPRAWWSQARRDGDDETGMRRKGAEMCWMCDHPEATAEEYLDEVYGIMLMHGWAVQHVECERRPFAYTVGLTRRGLPELVVTGLSPRRGQRLLNIAARRALVGDLLTPGMQTTLPAGPLVETVQVTHPDAHLYCAIAIFGDKVTALQLVWADRRGRWPWAADFDEGRGTQPVLGMRATRRSA

>641|CORE_REP|Org112_Gene1653#

MTASAPDGRPGQPEATNRRSQLKSDRRFQLLAAAERLFAERGFLAVRLEDIGAAAGVSGPAIYRHFPNKESLLVELLVGVSARLLAGARDVTTRSANLAAALDGLIEFHLDFALGEADLIRIQDRDLAHLPAVAERQVRKAQRQYVEVWVGVLRELNPGLAEADARLMAHAVFGLLNSTPHSMKAADSKPARTVRARAVLRAMTVAALSAADRCL

>642|CORE_REP|Org145_Gene4069#

MLNXRSADLTAHARIREAAIEQFGRHGFGVGLRAIAEAAGVSAALVIHHFGSKEGLRKACDDFVAEEIRSSKAAALKSNDPTTWLAQMAEIESYAPLMAYLVRSMQSGGELAKMLWQKMIDNAEEYLDEGVRAGTVKPSRDPRARARFLAITGGGGFLLYLQMHENPTDLRAALRDYAHDMVLPSLEVYTEGLLADRAMYEAFLAEAQQGEAHVG

>643|CORE_REP|Org59_Gene1889#

MIAAVXRFDTEKSMMDMARAERAELAAFLTTLTLQQWETPSLCAGWSVKEVVAHMISYEDLGVFGLLKRFAKGRIVRANEVGVDEFAGLSPQELADYVGRHLQPRGLTAGFGGMIALVDGMIHHQDIRRPLGQPRTIPAQRLDRVLRLMPKNPRLRARPRIKGLRLRATDLDWTIGTGPEVTGPGEALLMAMAGRPAAVSDLSGPGKPTLAGRLG

>644|CORE_REP|Org118_Gene1629#

MIATTRDREGATMITFRLRLPCRTILRVFSRNPLVRGTDRLEAVVMLLAVTVSLLTIPFAAAAGTAVHDSRSHVYAHQAQTRHPATATVIDHEGVIDSNTTATSAPPRTKITVPARWVVNGIERSGEVNAKPGTKSGDRVGIWVDSAGQLVDEPAPPARAIADAALAALGLWLSVAAGCGRPAGAHSGDSDPRSQRQLATRHRQPVLHAAVTTVR

>645|CORE_REP|Org118_Gene2992#

MKLADAIATAPRRTLKGTYWHQGPTRHPVTSCADPARGPGRYHRTGEPGVWYASNKEQGAWAELFRHFVDDGVDPFEVRRRVGRVAVTLQVLDLTDERTRSHLGVDETDLLSDDYTTTQAIAAARDANFDAVLAPAAALPGCQTLAVFVHALPNIEPERSEVRQPPPRLANLLPLIRPHEHNARLRAQIACNADTCRSRSNPAPTTLKASRPDGL

>646|CORE_REP|Org67_Gene2416#

MRIAAAVVSIGLAVIAGFAVPVADAHPSEPGVVSYAVLGKGSVGNIVGAPMGWEAVFTRPFQAFWVELPACNNWVDIGLPEVYDDPDLASFNGATTQTSATDQTHLVKQAVGVFASNDAADRAFHRVVDRTVGCSGQTTAIHLDDGTTQVWSFAGGPSTGTDEAWTKQEAGTDRRCFVQTRLRENVLLQAKVCQSGNAGPAVNVLAGAMQNTLG

>647|CORE_REP|Org68_Gene3974#

MAHQWAFPPRKKQQQGERSRESILDATERLMATKGYAATSISDIRDACGLAPSSIYWHFGSKEGVLAAMMERGAQRFFAAIPTWDEAHGPVEQRSERQLTELVSLQSQHPDFLRLFYLLSMERSQDPAVAAVVRRVRNTAIARFRDSITHLLPSDIPPGKADLVVAELTAFAVALSDGVYFAGHLEPDTTDVERMYRRLRQALEALIPVLLEET

>648|CORE_REP|Org96_Gene814#

MEFPLITANSLSSKTWRAMPRAYVAVASFSGGLVQSGMAKFAAFLRGVNVGGVNLKMAEVATALTDAGFCNVRTILASGNVLLESTCGAAEVREKTEATLRERFGYDAWALIYDVDTVRTIVAAYPFECELEGYQSYVTFVADAAILDELSALADTAGPDENISRGPDPLGVLYWQVPKGSTLDSTIGQTMGKKRYKSSTTTRNLRTLAKVLR

>649|CORE_REP|Org20_Gene1009#

MALESPASGMADGRRAAGSRQAFPPWPVRMRDGVHWSRIRLADRAHLEPWEPSADGEWTVRHTVAAWPAVCSGLRSEARNGRMLPYVIELYGQFCGQLTIGNVTHGALRSAWIGYWVPSAATGGGVATGALALGLDHCFGPVMLHRVEATVRPENAASRAVLAKVGFREEGLLRRYLEVDRAWRDHLLMAITVEEVYGSVASTLVRAGHASWP

>650|CORE_REP|Org59_Gene3050#

MRGPLLPPTVPGWRSRAERFDMAVLEAYEPIERRWQERVSQLDIAVDEIPRIAAKDPESVQWPPEVIADGPIALARLIPAGVDVRGNATRARIVLFRKPIERRAKDTEELGELLHEILVAQVAIYLDVDPSVIDPXIXDXXAPPTPAAGSDDPAFEAAALAFGKTTPDAEPLVMSQGVLQTLVPHLAAHANLLGLTRGAALLREERFGIRLRT

>651|CORE_REP|Org67_Gene2241#

MVDPGVSPGCVRFVTLEISPSMTMQGERLDAVVAEAVAGDRNALREVLETIRPIVVRYCRARVGTVERSGLSADDVAQEVCLATITALPRYRDRGRPFLAFLYGIAAHKVADAHRAAGRDRAYPAETLPERWSADAGPEQMAIEADSVTRMNELLEILPAKQREILILRVVVGLSAEETAAAVGSTTGAVRVAQHRALQRLKDEIVAAGDYA

>652|CORE_REP|Org59_Gene3201#

MSRVQISTVLAIDTATPAVTAGIVRRHDLVVLGERVTVDARAHXERLTPNVLAALADAALTMADLDAVVVGCGPGPFTGLRAGMASAAAYGHALGIPVYGVCSLDAIGGXXIGXTLXVTDARRRASVYWARYCDGIRTVGPAVNAAADVDPGPALAVAGAPEHAALFALPCVEPSRPSPAGLVAAVNWADKPAPLVPLYLRRPDAKPLXVXT

>653|CORE_REP|Org59_Gene2458#

MSRVQLALNVDDLEAAITFYSRLFNAEPAKRKPGYANFAIADPPLKLVLLENPGTGGTLNHLGVEVGSSNTVHAEIARLTEAGLVTEKEIGTTCCFATQDKVWVTGPGGEQLGGLYRAGRLRDLRQRSSAQRXQRRRSKHVLRRPSRRWRKRLTVGLTPGCXLXAAEPTPGHSMPPNPRSAVHRVAACGPDSIFVNIDVCRICIRYQRWRAA

>654|CORE_REP|Org34_Gene3162#

MPLFSFEGRSPRIDPTAFVAPTATLIGDVTIEAGASVWFNAVLRGDYAPVVVREGANVQDGAVLHAPPGIPVDIGPGATVAHLCVIHGVHVGSEALIANHATVLDGAVIGARCMIAAGALVVAGTQIPAGMLVTGAPAKVKGPIEGTGAEMWVNVNPQAYRDLAARHLAGLEPMQASLRVKPSAVLTSGCKSRSRRRRRRCPPPHQLGRRVR

>656|CORE_REP|Org4_Gene2108#

MSTPSATVAPVKRIPYAEASRALLRDSVLDAMRDLLLTRDWSAITLSDVARAAGISRQTIYNEFGSRQGLAQGYALRLADRLVDNVHASLDANVGNFYEAFLQGFRSFFAESAADPLVISLLTGVAKPDLLQLITTDSAPIITRASARLAPAFTDTWVATTDNDANVLSRAIVRLCLSYVSMPPEADHDVAADLARLITPFAERHGVINVP

>657|CORE_REP|Org69_Gene2884#

MPHPWDTGDHERNWQGYFIPAMSVLRNRVGARTHAELRDAENDLVEARVIELREDPNLLGDRTDLAYLRAIHRQLFQDIYVWAGDLRTVGIEKEDESFCAPGGISRPMEHVAAEIYQLDRLRAVGEGDLAGQVAYRYDYVNYAHPFREGNGRSTREFFDLLLSERGSGLDWGKTDLEELHGACHVARANSDLTGLVAMFKGILDAEPTYDF

>658|CORE_REP|Org53_Gene1592#

MGGTFDPIHYGHLVAASEVADLFDLDEVVFVPSGQPWQKGRQVSAAEHRYLMTVIATASNPRFSVSRVDIDRGGPTYTKDTLADLHALHPDSELYFTTGADALASIMSWQGWEELFELARFVGVSRPGYELRNEHITSLLGQLAKDALTLVEIPALAISSTDCRQRAEQSRPLWYLMPDGVVQYVSKCRLYCGACDAGARSTTSLAAGNGL

>659|CORE_REP|Org119_Gene1487#

MTTTQTAKASRRARIERRTRESDIVIELDLDGTGQVAVDTGVPFYDHMLTALGSHASFDLTVRATGDVEIEAHHTIEDTAIALGTALGQALGDKRGIRRFGDAFIPDGRNTGPRRRRLIRPPLLRAYRRAGSPCSTPLLPAVQCPTTPSSTRHVFESLAANARIALHVRVLYGRDPHHITEAQYKAVARALRQAVEPDPRVSGVPSTKGAL

>660|CORE_REP|Org27_Gene1940#

MVAARPAERSGDPAAVRVPVPSAWWVLIGGVIGLFASMTLTVEKVRILLDPIYVPSCNVNPIVSCGSVMTTPQASLLGFPNPLLGIAGFTVVVVTGVLAVAKVPLPRWYWIGLAVGILVGVAFVHWLIFQSLYRIGALCPYCMVVWAVIATLLVVVASIVFGPMRENRGSQERVGARLLYQWRWSLATLWFTTVFLLIMVRFWDYWSTLI

>661|CORE_REP|Org9_Gene938#

MTKPTSAGQADDALVRLARERFDLPDQVRRLARPPVPSLEPPYGLRVAQLTDAEMLAEWMNRPHLAAAWEYDWPASRWRQHLNAQLEGTYSLPLIGSWHGTDGGYLELYWAAKDLISHYYDADPYDLGLHAAIADLSKVNRGFGPLLLPRIVASVFANEPRCRRIMFDPDHRNTATRRLCEWAGCKFLGEHDTTNRRMALYALEAPTTAA

>662|CORE_REP|Org59_Gene2553#

MSRSTRYSVAVSAQPETGQIAGRARIANLANILTLLRLXXVPVFLLALFYGGGHHSAARVVAWAIFATACITDRFDGLLARNYGMATEFGAFVDPIADKTLIGSALIGLSMLGDLPWWVTVLILTRELGVTVLRLAVIRRGVIPASWGGKLKTFVQAVAIGLFVLPLSGPLHVAAXVVMAAAILLTVITGVDYVARALRDIGGIRQTAS

>663|CORE_REP|Org7_Gene1062#

MEPVLTQNRVLTVPNMLSVIRLALIPAFVYVVLSAHANGWGVAILVFSGVSDWADGKIARLLNQSSRLGALLDPAVDRLYMVTVPIVFGLSGIVPWWFVLTLLTRDALLAGTLPLLWSRGLSALPVTYVGKAATFGFMVGFPTILLGQCDPLWSHVLLACGWAFLIWGMYAYLWAFVLYAVQMTMVVRQMPKLKGRAHRPAAQNAGERG

>664|CORE_REP|Org59_Gene533#

MARLNVYVPDELAERARARGLNVSALTQAAISAELENSATDAWLEGLEPRSTGARHDDVLGAIDAARDEFEXXEHRPLRRRSRWXSTRVPWWIYWLALAIGSSAVRARLARTAMHAPAHFDAEVLSALGRMQRAGALTVAYVDAALEELRQVPVTRHGLSSLLAGAWSRRDTLRLTDALYVELAETAGLXLLTTXERLARAWPSAHAIG

>665|CORE_REP|Org109_Gene241#

MRLTHPARRYLSSQAARPTGAFGRLLGRIWRAETADVNRIAVELLAPGPGERVCEIGFGPGRTLGLLAAAGAQVSGVEVSTTMIAIAAHHNAKAIAAGLISLYHGDGVTLPVADHSLDKVLGVHNFYFWPDPRASLCDIARALRPGGRLVLTSISDDQPLAARFDPAIYRVPPTLDTAAWLGAAGFIDVGIKRSADHPATVWFTATAT

>667|CORE_REP|Org28_Gene1370#

MLDYLRDAAEIYRRSFAVIRAEADLARFPADVARVVVRLIHTCGQVDVAEHVAYTDDVVARAGAALAAGAPVLCDSSMVAAGITTSRLPADNQIVSLVADPRATELAARRQTTRSAAGVELCAERLPGAVLAIGNAPTALFRLLELVDEGAPPPAAVLGGPVGFVGSAQAKEELIERPRGMSYLVVRGRRGGSAMAAAAVNAIASDRE

>668|CORE_REP|Org1_Gene3126#

MSVMTGPTTDADAAVPRRVLIAEDEALIRMDLAEMLREEGYEIVGEAGDGQEAVELAELHKPDLVIMDVKMPRRDGIDAASEIASKRIAPIVVLTAFSQRDLVERARDAGAMAYLVKPFSISDLIPAIELAVSRFREITALEGEVATLSERLETRKLVERAKGLLQTKHGMTEPDAFKWIQRAAMDRRTTMKRVAEVVLETLGTPKDT

>669|CORE_REP|Org2_Gene567#

MFVGFVAMMTLKVAIGPQNAFVLRQGIRREYVLVIVALCGIADGALIAAGVGGFAALIHAHPNMTLVARFGGAAFLIGYALLAARNAWRPSGLVPSESGPAALIGVVQMCLVVTFLNPHVYLDTVVLIGALANEESDLRWFFGAGAWAASVVWFAVLGFSAGRLQPFFATPAAWRILDALVAVTMIGVAVVVLVTSPSVPTANVALII

>670|CORE_REP|Org86_Gene1143#

MAAPDNSRRRPGRPAGSSDTRERILSSARELFAHNGIDRTSIRAVAAKAGVDAALVHHYFGTKQQLFAAAIHIPIDPMVIIGPIREAPVEELGYKLPSLLLPIWDSELGAGLIATLRSLISGSDVGLARSFLEEVVTVELGSRVDNPPGTGKIRTQFVASQLMGVVMARYIVRIEPFASLPAEQIVQTIAPNLQRYLTGELPDDLAP

>672|CORE_REP|Org102_Gene1803#

MPQGNPLAVPNDGLTTRARRNMPILAVHTGEGKGKSTAAFGMALRAWNAGLDIAVFQFVKSAKWKVGEEAAFRQLGRLHDQHGIGGAVEWHKMGAGWSWTRTSRKAGTDVDRAAAAADGWAEIALRLATQRHDFYLLDEFTYPLKWGWLDVDEVVDVLRARPGHQHVVITGRDAPQRLVAAADLVTEMTKVKHPMDAGRKGQKGIEW

>673|CORE_REP|Org149_Gene2825#

MLTVVAVIGILECGLVLHMPDNDLWYCGPWTLWVMAGRGVASGAGVWRGDRVATPLAVAITAAGLVSGARIGPGAAAKRDPQLAQWNEIRSHYQEIAEWIDHDTATAHPAVAATQISAAGSFGRANMVDYLGLLDSRADETVRRDEFSRWLSAKPDYLVTTEQSVDAATIALPEFRHAYDRAATIGTLNVYRRNSPDGDEPLPADGN

>674|CORE_REP|Org62_Gene208#

MPDFPTQRGRRTQAAIDAAARTVVVRNGILATTVADITAEAGRSAASFYNYYDSKEAMVRQWALRFRDDANQRALSVIRHGLSDRERAYEAAAAHWYTYRNRLAEAISVSQLAMVSDDFAQYWSEICQIPISFITETVKRAQAHGYCVGDDPQLMAEAIVAMFNQFCYLQLSGKRSRRGQPDDQACIQTLANIYYRAIYSKEDSSN

>675|CORE_REP|Org118_Gene3123#

MSVQTDPALREHPNRVDWNARYQLAGATAHAPFAPVPWLADVLRAGVPDGPVLELASGRSGTALALAAHGRQVTAIDVSDVALLQLDSEAVRRGVADRLNLVQADLGCWEPGETRFALVLSRLFWDAAIFHRACEAVMPGGVLAWESLALSGAEAGTASAKRRVKPGEPACLLPADFTVVHEGQGNCDSAPSRIMIARRSPLPGA

>676|CORE_REP|Org61_Gene841#

MSGDGLVRCPWAEVRPGPDAQLYRDYHDNEWGRPLYGRVALFERMSLEAFQSGLSWLIILRKRENFRRAFSGFDIDKIARYTDTDVRRLLADDGIVRNRAKIEATIANARAAADLGSSEDLSELLWSFAPPPRPRPVDGSEIPSVSTESKAMSRELKRRGFRFVGPTTAYALMQATGMVDDHIQACWVPTERPFDQPGCPMAAR

>677|CORE_REP|Org32_Gene182#

MAGGTKRLPRAVREQQMLDAAVQMFSVNGYHETSMDAIAAEAQISKPMLYLYYGSKEDLFGACLNREMSRFIDALRSSINFDQSPKDLLRNTIVSFLRYIDANRASWIVMYTQATSSQAFAHTVREGREQIVQLVAELVRAGTRGPLTDAEIEMMAVALVGAGEAVATRLGIGDTDVDEAAEMMINLFWLGLKGAPVDRLETGH

>678|CORE_REP|Org139_Gene2426#

MFEGPVQDLIDELGKLPGIGPKSAQRIAFHLLSVEPSDIDRLTGVLAKVRDGVRFCAVCGNVSDNERCRICSDIRRDASVVCIVEEPKDIQAVERTREFRGRYHVLGGALDPLSGIGPDQLRIRELLSRIGERVDDVDVTEVIIATDPNTEGEATATYLVRMLRDIPGLTVTRIASGLPMGGDLEFADELTLGRALAGRRVLA

>679|CORE_REP|Org100_Gene2704#

MTRSYRPAPPIERVVLLNDRGDATGVADKATVHTGDTPLHLAFSSYVFDLHDQLLITRRAATKRTWPAVWTNSCCGHPLPGESLPGAIRRRLAAELGLTPDRVDLILPGFRYRAAMADGTVENEICPVYRVQVDQQPRPNSDEVDAIRWLSWEQFVRDVTAGVIAPVSPWCRSQLGYLTKLGPCPAQWPVADDCRLPKAAHGN

>680|CORE_REP|Org25_Gene1456#

MTSAVGTSGTAITSRVHSLNRPNMVSVGTIVWLSSELMFFAGLFAFYFSARAQAGGNWPPPPTELNLYQAVPVTLVLIASSFTCQMGVFAAERGDIFGLRRWYVITFLMGLFFVLGQAYEYRNLMSHGTSIPSSAYGSVFYLATGFHGLHVTGGLIAFIFLLVRTGMSKFTPAQATASIVVSYYWHFVDIVWIALFTVIYFIR

>681|CORE_REP|Org74_Gene2097#

MGVRNHRLLLLRHGETAWSTLGRHTGGTEVELTDTGRTQAELAGQLLGELELDDPIVICSPRRRTLDTAKLAGLTVNEVTGLLAEWDYGSYEGLTTPQIRESEPDWLVWTHGCPAGESVAQVNDRADSAVALALEHMSSRDVLFVSHGHFSRAVITRWVQLPLAEGSRFAMPTASIGICGFEHGVRQLAVLGLTGHPQPIAAG

>682|CORE_REP|Org59_Gene2871#

MTAGSDRRPRDPAGRRQAIVEAAERVIARQGLGGLSHRRVAAEANVPVGSTTYYFNDLDALREAALAHAANASADLLAQWRSDLDKDRDLAATLARHSPPSTWPTRTAIARSTSCXWRQLIDRNCSAXPGCGQMVYSRCSNRASGRRAANAVTVFFDGATLHALITGTPLSTDELTDAIARLVADGPEQREVGQSAHAGRTPD

>683|CORE_REP|Org59_Gene49#

MLELAILGLLIESPMHGYELRKRLTGLLGAFRAFSYGSLYPALRRMQADGLIAENAAPAGTPVRRARRVYQLTDKGRRRFGELVADTGPHNYTDDGFGVHLAFFNRTPAEARMRILEGRRRQVEERREGLREAXARGQQFLRPLHPPIASTRARVQRARGQVAQRAHRRGTGSTQPRRTDVNPARTNTQDTPEVMRLGERLNE

>684|CORE_REP|Org21_Gene1049#

MPDEPTGSADPLTSTEEAGGAGEPNAPAPPRRLRMLLSVAVVVLTLDIVTKVVAVQLLPPGQPVSIIGDTVTWTLVRNSGAAFSMATGYTWVLTLIATGVVVGIFWMGRRLVSPWWALGLGMILGGAMGNLVDRFFRAPGPLRGHVVDFLSVGWWPVFNVADPSVVGGAILLVILSIFGFDFDTVGRRHADGDTVGRRKADG

>685|CORE_REP|Org141_Gene873#

MAGTDWLSARRTELAADRILDAAERLFTQRDPASIGMNEIAKAAGCSRATLYRYFDSREALRTAYVHRETRRLGREIMVKIADVVEPAERLLVSITTTLRMVRDNPALAAWFTTTRPPIGGEMAGRSEVIAALAAAFLNSLGPDDPTTVERRARWVVRMLTSLLMFPGRDEADERAMIAEFVVPIVTPASAAARKAGHPGPE

>686|CORE_REP|Org120_Gene3695#

MKARELDVPGAWEITPTIHVDSRGLFFEWLTDHGFRAFAGHSLDVRQVNCSVSSAGVLRGLHFAQLPPSQAKYVTCVSGSVFDVVVDIREGSPTFGRWDSVLLDDQDRRTIYVSDGLAHGFLALQDNSTVMYLCSAEYNPQREHTICATDPTLAVDWPLVDGAAPSLSDRDAAAPSFEDVRASGLLPRWEQTQRFIGEMRGT

>688|CORE_REP|Org6_Gene3166#

MTGPPRSYTGRRDLIAEKLEPYFQISAMLPKNTRPTSETAEEFWDNSLWCSWGDRETGYTRTVTVSICQVADGEREAEGVRDMMRLECPAGLDLRTPNPEAYEITGQRPGEFVFVLGYLGHVRAIVGNCYIEIMPMGTRVELSKLADVALDIGRSVGCSAYENDFTLPDIPTQWRNQPLGWYTQGLAPYLPGLSDPKDAAEG

>689|CORE_REP|Org77_Gene2535#

MLGPIRQPRLTVRPGRLPGMIAGVAAKRMNREQFFRAASGLDEDRLRKALWNLYWRGTANMRERIEAELASAGRARPARKIKPPADPDIVGWEVDEFVSLARSGAYLGGDRRVSPRERSRWRFTFKRLAAEAQDALRAEDAEPAASALEQLIDLAREADGYDYFRSDDPVAAAGFVVSDVAAAGHPHFREFAAEIGAAIPP

>690|CORE_REP|Org39_Gene973#

MFTGIVEERGEVTGREALVDAARLTIRGPMVTADAGHGDSIAVNGVCLTVVDVLPDGQFTADVMAETLNRSNLGELRPGSRVNLERAAALGSRLGGHIVQGHVDATGEIVARCPSEHWEVVRIEMPASVARYVVEKGSITVDGISLTVSGLGAEQRDWFEVSLIPTTRELTTLGSAAVGTRVNLEVDVVAKYVERLMRSAG

>691|CORE_REP|Org32_Gene822#

MTVSAPAKANPYRRRGEVLERALYDATLAELESAGYGGLTMEGIAARAQTGKAALYRRWAGKRELVLAAVQYALPPVPEPRADRSARENLLAVFTANCEILAGKTALPSMEIVSQLLHEPELRAIFINSVWAPRLRIVESILQAGVRSGEIDPATLTPMTARIGPALIHQHVLFTGSPPDREQLTRIIDAMILTTGERRES

>692|CORE_REP|Org137_Gene3434#

MSTETRVNERIRVPEVRLIGPGGEQVGIVRIEDALRVAADADLDLVEVAPNARPPVCKIMDYGKYKYEAAQKARESRRNQQQTVVKEQKLRPKIDDHDYETKKGHVVRFLEAGSKVKVTIMFRGREQSRPELGYRLLQRLGADVADYGFIETSAKQDGRNMTMVLAPHRGAKTRARARHPGEPAGGPPPKPTAGDSKAAPN

>693|CORE_REP|Org61_Gene241#

MQQQRTNRDKLLDGALACLRERGYGNTSSRDIARAAGVNIASINYHFGSKDALLDDALGRCFSTWNQRVQEAFDHSRAAGPAGQILAVLEATVDSFEQIRPAVYACVESYAPALRSEALRERLAAGYADVRQHSVDLAGAALAGTDIAPPENLSTIVSVLMAVIDGLMIQWIADPSATPRSTEVIRALASIGAVVTSQLR

>695|CORE_REP|Org6_Gene2123#

MNAVPSDLTPRVWPAMLTWRAQDISRMESVRVQLSGKRIRANGRIVAAATANNPAFGAHYDLQTDETGATKRFGLTVTLAERERQLAIARDEENMWLVTDHQGERRAAYNGALDIDLVFSPFFNALPIRRLGLHERAESIALPVVYVNVPEMSVDAATVSYTSEGRLDGIKLRSPVADTTVTVDSDGFIVDYPGLAERM

>696|CORE_REP|Org10_Gene3899#

MTECFLSDQEIRKLNRDLRILIAANGTLTRVLNIVADDEVIVQIVKQRIHDVSPKLSEFEQLGQVGVGRVLQRYIILKGRNSEHLFVAAESLIAIDRLPAAIITRLTQTNDPLGEVMAASHIETFKEEAKVWVGDLPGWLALHGYQNSRKRAVARRYRVISGGQPIMVVTEHFLRSVFRDAPHEEPDRLQFSNAITLAR

>697|CORE_REP|Org94_Gene867#

MTAPSGSSGESAHDAAGGPPPVGERPPEQPIADAPWAPPASSPMANHPPPAYPPSGYPPAYQPGYPTGYPPPMPPGGYAPPGYPPPGTSSAGYGDIPYPPMPPPYGGSPGGYYPEPGYLDGYGPSQPGMNTMALVSLISALVGVLCCIGSIVGIVFGAIAINQIKQTREEGYGLAVAGIVIGIATLLVYMIAGIFAIP

>698|CORE_REP|Org41_Gene1966#

MEAFHTHSGIGVPLRRSNVDTDQIIPAVFLKRVTRTGFEDGLFAGWRSDPAFVLNLSPFDRGSVLVAGPDFGTGSSREHAVWALMDYGFRVVISSRFGDIFRGNAGKAGLLAAEVAQDDVELLWKLIEQSPGLEITANLQDRIITAATVVLPFKIDDHSAWRLLEGLDDIALTLRKLDEIEAFEGACAYWKPRTLPAP

>699|CORE_REP|Org104_Gene1057#

MRWIGVLVTALVLSACAANPPANTTSPTAGQSLDCTKPATIVQQLVCHDRQLTSLDHRLSTAYQQALAHRRSAALEAAQSSWTMLRDACAQDTDPRTCVQEAYQTRLVQLAIADPATATPPVLTYRCPTQDGPLTAQFYNQFDPKTAVLNWKGDQVIVFVELSGSGARYGRQGIEYWEHQGEVRLDFHGATFVCRTS

>700|CORE_REP|Org136_Gene734#

MTGTERRHQLIGIARSLFAERGYDGTSIEEIAQRANVSKPVVYEHFGGKEGLYAVVVDREMSALLDGITSSLTNNRSRVRVERVALALLTYVEERTDGFRIMIRDSPASISSGTYSSLLNDAVSQVSSILAGDFARRGLDPDLAPLYAQALVGSVSMTAQWWLDAREPKKEVVAAHLVNLVWNGLTHLEADPRLQDE

>701|CORE_REP|Org142_Gene281#

MTATQITGVVLAAGRSNRLGTPKQLLPYRDTTVLGATLDVARQAGFDQLILTLGGAASAVRAAMALDGTDVVVVEDVERGCAASLRVALARVHPRATGIVLMLGDQPQVAPATLRRIIDVGPATEIMVCRYADGVGHPFWFSRTVFGELARLHGDKGVWKLVHSGRHPVRELAVDGCVPLDVDTWDDYRRLLESVPS

>702|CORE_REP|Org19_Gene2300#

MFLYVAVGSLVVARLLLYPLRPADLTPPYWVAMGATAITVLAGAHIVEMADAPMAIVTSGLVAGASVVFWAFGPWLIPPLVAASIWKHVVHRVPLRYEATLWSVVFPLGMYGVGAYRLGLAAHLPIVESIGEFEGWVALAVWTITFVAMLHHLAATIGRSGRSSHAIGAADDTHAIICRPPRSFDHQVRAFRRNQPM

>703|CORE_REP|Org1_Gene1468#

MTVTDDYLANNVDYASGFKGPLPMPPSKHIAIVACMDARLDVYRMLGIKEGEAHVIRNAGCVVTDDVIRSLAISQRLLGTREIILLHHTDCGMLTFTDDDFKRAIQDETGIRPTWSPESYPDAVEDVRQSLRRIEVNPFVTKHTSLRGFVFDVATGKLNEVTPSSPSRQPRAHWRTGSPPRWGCVDSDREAWLHRWQ

>704|CORE_REP|Org60_Gene1290#

MISDTEDFAHGDKAAPPRLRASYAACGGDAAGCWTMSDNGASRVPPVDETPAAESAEPITAVSLAWLPAGDYERALDLWPDFAGSDLVTGPDGPVAHPLYCRRMQQKLVEFAEAGFPGLAVAAIRVAPFAAWCAEQGQEPDSPEARAEYAAYLTAHGDHDVMAWPPGRNQQCWCGSGHKYKKCCAAASFIDTEPAP

>705|CORE_REP|Org55_Gene819#

MPNLQLVQEPAADALLNANPFALLVGMLLDQQVPMETAFAGPKKIADRMGSFDAGDIADYDPDKFVALCSERPAIHRFPGSMAKRIQALAQIIVDRYDGDAAALWTAGEPDGNELLRRLKGLPGFGEQKARIFLALLGKQYGVTPKGWQVAAGEFGQPGTYLSVADIVDAGSLGQVRSHKRQRKAAAKAEGKAPT

>706|CORE_REP|Org18_Gene2634#

MCHTAPMEPSPVVSPLPRLLPHLWKSTLASGILSLILGVLVLAWPGISILVAAMAFGVYLLITGVAQVAFAFSLHVSAGGRILLFISGAASLILAVLAFRHFGDAVLLLAIWIGIGFIFRGVATTVSAISDPMLPGRGWSIFVGVISLIAGIVVMASPFESIWILALVVGIWLVVIGTCEIASSFAIRKASQTLG

>707|CORE_REP|Org109_Gene839#

MLLAYVLITKGEFGAAASMLEPAAATLERTGYSWGPLSLMLLATAIAQQGHIAESAKTLQRAEARHGTKSALFAPELGLARAWTRAAAQDMTGAIAAAREAARTAERAGQAAVALCAWHNAVRLGDIRAVDPVTRLAAEIDCTVGNILVKHARGLADGDAAELTAVAEELAGIGMAAAAADATKAAARLGPQQR

>708|CORE_REP|Org66_Gene945#

MGAAGDAAIGRESRELMSAADVGRTISRIAHQIIEKTALDDPVGPDAPRVVLLGIPTRGVTLANRLAGNITEYSGIHVGHGALDITLYRDDLMIKPPRPLASTSIPAGGIDDALVILVDDVLYSGRSVRSALDALRDVGRPRAVQLAVLVDRGHRELPLRADYVGKNVPTSRSESVHVRLREHDGRDGVVISR

>709|CORE_REP|Org16_Gene2174#

MALKHSEASGTASTKIVIAGGFGSGKTTFVGAVSEIMPLRTEAMVTDASAGVDMLEATPDKRSTTVAMDFGRITLGEDLVLYLFGTPGQRRFWFMWDDLVRGAIGAIVLVDCRRLQDSFAAVDFFEHRNLPFLIAINEFDSAPRYPVSAVRDALTLPAHIPVINVDARNRRSATDALIAVSEYALATLSPAGG

>710|CORE_REP|Org1_Gene3698#

MQWGYHPRAGDEAMRRSGAYDSCRRAAPGRVIMGSDSDWPVMADAAAALAEFDIPAEVRVVSAHRTPEAMFSYARGAAERGLEVIIAGAGGAAHLPGMVAAATPLPVIGVPVPLGRLDGLDSLLSIVQMPAGVPVATVSIGGAGNAGLLAVRMLGAANPQLRARIVAFQDRLADVVAAKDAELQRLAGKLTRD

>711|CORE_REP|Org132_Gene2350#

MSQLSFFAAESVPPAVADLSGVLAGPGQIVLVGCGARLSVVVAESWRASALAEMIQEAGLVPEVARTDENTPLVRTAVDPLLCGIAAEWTRGAVKTVPPRWLPGPRELRAWTLAAGSPEADRYLLGLDPHAPDTHSPLASALMRVGIAPTLIGTRGTRPALRISGRRRLSRLVENVGEPPDGAEAWVQWPRT

>712|CORE_REP|Org118_Gene876#

MAPRVGPRQARSANPQIGPKLHRAGAAVTANVWCRAGGIRMAPRPVIPVATQQRLRRQADRQSLGSSGLPALNCTPIRHTIDVMATKPERKTERLAARLTPEQDALIRRAAEAEGTDLTNFTVTAALAHARDVLADRRLFVLTDAAWTEFLAALDRPVSHKAPGWRSCSPRGPFSTPRGERLQRAATYQRRR

>713|CORE_REP|Org111_Gene2755#

MSVAVDSDAEDDAVSEIAEAAGVSPAPAKPSMSAPRRMLLFGLVVVVALAVLLCCWGFRVQRARHAQDQRGHFLQAARQCALNLTTIDWRNAEADVRRILDGATGEFYNDFAQRSQPFVEVLRHAKASTVGTITEAGLQTQTADTAQALVAVSVQTSNAGEADPVPRAWRMRITVQRVGDRVKVSDVGFVP

>715|CORE_REP|Org73_Gene4093#

MHYPVWRQSWTGILDPYLLDMIGSPKLWVEESYPQSLKRGGWSMWIAESGGQPIGMTMFGPDIAHPDRIQIDALYVAENSQRHGIGGRLLNRALHSHPSAEPPRHVRRLQFLERMGSCQVVHRGGTRRSCVSGRCGWSQRSAVSTIRSGQRSVRSPVYLVLAARRRCVSGCARRRSMPAHGPGPRPKNPLS

>716|CORE_REP|Org43_Gene337#

MTGQNGQVARISPGKFRQLGPVNWLVAKLAARAVGAPQMHLFTTLGYRQYLFWTFAIYTGRLLHGRLPGVDTELVILRVAHLRSCEYELQHHRRMARRRGLDANTQATIFAWPDVPDGDGPRKVLSARQQALLQATDELIKDRTITAGTWERLATHLDPRLLIEFCLLATQYDAIAATITALAIPPDNPQ

>717|CORE_REP|Org2_Gene4243#

MNSEGTVDLPGNDFDSNDFDAVDLWGADGAEGWTADPIIGVGSAATPDTGPDLDNAHGQAETDTEQEIALFTVTNPPRTVSVSTLMDGRIDHVELSARVAWMSESQLASEILVIADLARQKAQSAQYAFILDRMSQQVDADEHRVALLRKTVGETWGLPSPEEAAAAEAEVFATRYSDDCPAPDDESDPW

>718|CORE_REP|Org1_Gene2599#

MFSMPHSTADRRLRLTRQALLAAAVVPLLAGCALVMHKPHSAGSSNPWDDSAHPLTDDQAMAQVVEPAKQIVAAADLQAVRAGFSFTSCNDQGDPPYQGTVRMAFLLQGDHDAYFQHVRAAMLSHGWIDGPPPGQYFHGITLHKNGVTANMSLALDHSYGEMILDGECRNTTDHHHDDETTNITNQLVQP

>719|CORE_REP|Org20_Gene1262#

MSKPFAPRRLYTPRTSRTLAPRLDPEAVGRTTESIARFFGTGRYLLVQTLLVLTWIVLNLFAVGLRWDPYPFILLNLAFSTQASYAAPLILLAQNRQEKRDRAVFEEDRRRAAQTKADTEYNARELAALRLAIGESPRALPAPRTGQPARSVGGAAADGSGCGAAAGGRRGGAARQEIGVITESAIAPLP

>720|CORE_REP|Org2_Gene539#

MADNDYRSAPGTEPFVPDFDTGAHSQRFLSLAGQQDRAGKSWPGSTPKPQEDPVGVAPSASVEVLGSEPAATLAHSVTVPGRYTYLKWWKFVLVVLGVWIGAGEVGLSLFYWWYHTLDKTAAVFVVLVYVVACTVGGLILALVPGRPLITALSLGVMSGPFASVAAAAPLYGYYYCERMSHCLVGVIPY

>721|CORE_REP|Org119_Gene3151#

MTTRPATDRRKMPTGREEVAAAILQAATDLFAERGPAATSIRDIAARSKVNHGLVFRHFGTKDQLVGAVLDHLGTKLTRLLHSEAPADIIERALDRHGRVLARALLDGYPVGQLQQRFPNVAELLDAVRPRYDSDLGARLAVAHALALQFGWRLFAPMLRSATGIDELTGDEERQLSVNDAVARILEPH

>722|CORE_REP|Org102_Gene2269#

MTRVVLSVGSNLGDRLARLRSVADGLGDALIAASPIYEADPWGGVEQGQFLNAVLIADDPTCEPREWLRRAQEFERAAGRVRGQRWGPRNLDVDLIACYQTSATEALVEVTARENHLTLPHPLAHLRAFVLIPWIAVDPTAQLTVAGCPRPVTRLLAELEPADRDSVRLFRPSFDLNSRHPVSRAPES

>723|CORE_REP|Org59_Gene3676#

MADADTTDFDVDAEAPGGGVREDTATDADEADDQEERLVAEGEIAGDYLEELLDVLDFDGDIDLDVEGNRAVVSIDGSDDLNKLVGRGGEVLDALQELTRLAVHQKTGVRSRLMLDIARWRRRRREELSXAVXDEVARRVAETGDREELVPMTPFERKIVHDAVAAVPGVHSESEGVEPERRVVVLRD

>724|CORE_REP|Org118_Gene2617#

MRWPTAWLLALVCVMATGCGPSGHGTRAGEEGPLSPEKVAELENPLRAKPPLEDAKDQYRAAVTQLANAITALVPGLTWRTDMDTWTGCGGEYEWTRAKAAYFMIVFSGPIPDDKWLQAVQIVKDGVEQFGATGFGVMKNKPADHDVYFAGHGGVEFKCSTQKAAVLTAQSTARRISRTDTPKPSPTP

>725|CORE_REP|Org62_Gene2308#

MSAQIDPRTFRSVLGQFCTGITVITTVHDDVPVGFACQSFAALSLEPPLVLFCPTKVSRSWQAIEASGRFCVNVLTEKQKDVSARFGSKEPDKFAGIDWRPSELGSPIIEGSLAYIDCTVASVHDGGDHFVVFGAVESLSEVPAVKPRPLLFYRGDYTGIEPEKTTPAHWRDDLEAFLTTTTQDTWL

>726|CORE_REP|Org9_Gene2544#

MSAKIDITGDWTVAVYCAASPTHAELLELAAEVGAAIAGRGWTLVWGGGHVSAMGAVASAARACGGWTVGVIPKMLVYRELADHDADELIVTDTMWERKQIMEDRSDAFIVLPGGVGTLDELFDAWTDGYLGTHDKPIVMVDPWGHFDGLRAWLNGLLDTGYVSPTAMERLVVVDNVKDALRACAPS

>727|CORE_REP|Org111_Gene1018#

MPKVSEDHLAARRRQILDGARRCFAEYGYDKATVRRLEQAIGMSRGAIFHHFRDKDALFFALAREDTERMAAVASREGLIGVMRDMLAAPDQFDWLATRLEIARKLRNDPDFSRGWAERSAELAAATTDRLRRQKQANRVRDDVPSDVLRCYLDLVLDGLLARLASGEDPQRLAAVLDLVENSVRRS

>728|CORE_REP|Org86_Gene524#

MTTAQKVQPRLKERYRSEIRDALRKQFGYGNVMQIPTVTKVVVNMGVGEAARDAKLINGAVNDLALITGQKPEVRRARKSIAQFKLREGMPVGVRVTLRGDRMWEFLDRLTSIALPRIRDFRGLSPKQFDGVGNYTFGLAEQAVFHEVDVDKIDRVRGMDINVVTSAATDDEGRALLRALGFPFKEN

>729|CORE_REP|Org55_Gene1191#

MQNHDYVTYEEFGRRFFEVAVTPDRVAAAFADIAGSEFAMEPISQGPGGIAKVSANVKIREPRVTRKLGDLITFVIHIPLSIDLLLDLRLDKQRFMVAGDIALRATARAAEPLLLIVDVAKPRPSDITVNVSSKSIRGEVLRILAGVDGEIRRFIAQYVSAEIDSPKSQAAQVINVAEQLDSTWSGP

>730|CORE_REP|Org1_Gene2610#

MPMTATASDDEAVTALALSAAKGNGRALEAFIKATQQDVWRFVAYLSDVGSADDLTQETFLRAIGAIPRFSARSSARTWLLAIARHVVADHIRHVRSRPRTTRGARPEHLIDGDRHARGFEDLVEVTTMIADLTTDQREALLLTQLLGLSYADAAAVCGCPVGTIRSRVARARDALLADAEPDDLTG

>731|CORE_REP|Org1_Gene766#

MRDLRNRTSQVVDAVKAGVPVTLTVHGEPVADIVPHRRRIRWLSGRICAMSSPSARPTRASPMNSTTWPVIPSTTCDRGRGRVGLLDTSVFIARESGGAIADLPERVALSVMTIGELQLGLLNAGDSATRSRRADTLALARTADQIPVSEAVMISLARLVADCRAAGVRRSVKLTDALIAATAEIKV

>732|CORE_REP|Org118_Gene1524#

MLKGVTDPLQHGAFEPGWQSAPPGYPPPYPQYPGPGSYFDPFAPYGRHPVTGQPFSDKSKTVAGLLQLLGLFGIAGIGRIYLGHTGLGIAQLLVGWVTCGLGAVIWGVIDALLILTDKVGDPWGRPLRDGSLAGVNVATPRPVRSCYWPARLATSDLSTRTTRIRYIHRAYSSCLRAGTAPRAGVCG

>733|CORE_REP|Org59_Gene2999#

MALGAVATAVIINSGDSTSTKAIVGAPAPRTVISTSPRPTAPTXTTSPHPSPSTLRPQLPPETVTTVAPPGTGPTTVPTRTPTAAPPQTAVPPPAPLNXRTVVYRVTGTKQLFDLVNVVYTDARGFPVTDFNVSLPWTKMVVLNPGVQTESVVATSLYSRLNCSIVNTGAQTVVASTNNAIIATCTR

>734|CORE_REP|Org84_Gene262#

MRLSLIARGMAALLAATALVAGCNTTIDGRPVASPGSGPTEPTFPTPRPTTAPPGTTAPTLPTTPVSPTAPAGAIPLPPDSNGYVFIETKSGMTRCQINRDSVGCEAPFTNSPLRDGEHANGIHITAGGSVQWVLGNLGAIPTVSIDYRTYEAQGWTIDATTDGTRFTNNRTGHGMFVSIEKVDTF

>735|CORE_REP|Org113_Gene1014#

MTGPYFPQTIPFLPSYIPQDVDMTAVKAEVAALGVSAPPAATPGLLEVVQHARDEGIDLKIVLLDHNPPNDTPLRDIATVVGADYSDATVLVLSPNYVGSYSTQYPRVTLEAGEDHSKTGNPVQSAQNFVHELSTPEFPWSALTIVLLIGVLAAAVGARLMQLRGRRSATSTDAAPGAGDDLNQGV

>736|CORE_REP|Org7_Gene1140#

MSDALDEGLVQRIDARGTIEWSETCYRYTGAHRDALSGEGARRFGGRWNPPLLFPAIYLADSAQACMVEVERAAQAASTTAEKMLEAAYRLHTIDVTDLAVLDLTTPQAREAVGLENDDIYGDDWSGCQAVGHAAWFLHMQGVLVPAAGGVGLVVTAYEQRTRPGQLQLRQSVDLTPALYQELRAT

>737|CORE_REP|Org125_Gene2739#

MPGNDWIVGGNRRTIAAERIYAAATDLITRYGLNALDIDKLAREVHCSRATIYRRAGGKAQIRDVVLTRAAARIADGVRSDVETLRGRERVVAAILLSLQRIRSDPLGKLMFGSIHGGAGELAWLTESPLLADFATELTGIAGGDPQGAKWVVRVVLSLMYWPAENDEAERRLVEKYVAPAFAEQS

>738|CORE_REP|Org30_Gene1197#

MLRAVNEIRQHDGTLKLGKGVGMFTIVGVIVALIGAFVQSRRHRHRPAADIHMLWWMVLIVGVVSIIGAGYHVFDGERTAELIGYTRGDGGFQWENAMGDLAIGVVGLMAYRFRGHFWLATIVVLTIQYVGDAAGHIYYWVVENNTNPYNIGVPLWTDILLPIVMWALYAWSWHSNGDAVPKGQP

>739|CORE_REP|Org110_Gene545#

MTRQQLAHLLRRACAVVGDVDVLVLGSQSILGSFDENELPPQATASQEADIAFVNDPARDKADHVDVAIGEMSDFHRSNGVYAEGVHIDTAILPNGWRDRLVSWTVESSRPAKPRFLEPHDLAVAKLAAGREKDKAFVAALIRSGLLDVGVIQARVLLLPEETDPRIGQRIAAWLNYYGAGNHSS

>740|CORE_REP|Org29_Gene1887#

MIDEALFDAEEKMEKAVAVARDDLSTIRTGRANPGMFSRITIDYYGAATPITQLASINVPEARLVVIKPYEANQLRAIETAIRNSDLGVNPTNDGALIRVAVPQLTEERRRELVKQAKHKGEEAKVSVRNIRRKAMEELHRIRKEGEAGEDEVGRAEKDLDKTTHQYVTQIDELVKHKEGELLEV

>741|CORE_REP|Org119_Gene900#

MLKGFKEFLARGNIVDLAVAVVIGTAFTALVTKFTDSIITPLINRIGVNAQSDVGILRIGIGGGQTIDLNVLLSAAINFFLIAFAVYFLVVLPYNTLRKKGEVEQPGDTQVVLLTEIRDLLAQTNGDSPGRHGGRGTPSPTDRASREHRIAIARHSIRIQISKLDNCPMICAASGFSVAIPSRTA

>742|CORE_REP|Org59_Gene3152#

MFRLLFVSPRIAPNTGNAIRTCAATGCELHLVEPLGFDLSEPKLRRAGLDYHDLASVTVHASLAHAWEALSPARVFAFTAQATTLFTNVGYRAGDVLMFGPEPTGLDEATLADTHITGQVRIPIYAGGXAXVEPVQRRSRRGLRGLASARLCRGGLVATKVTPNPAGMRTTKLIGVGRRTGAPNR

>745|CORE_REP|Org118_Gene3031#

MSSPVSSRRLANLVKESLQGSVLGGVVSDAVLPAVSDDVKPGAGEDAYRVPVVVAAGSGAVVQVGGLEVGSAAVAGEVADTVAELFVCRPTEPDVGDFVGLAGGAGDAGQAGQQFGLGVGVRGESFGARRRSLALSTVGASGATAGLRKTHDGHHGCQARGALTQRRLYIGNPSEITDTRMVHQ

>746|CORE_REP|Org150_Gene2746#

MTTGLPSQRQVIELLGADFACAGYEIEDVVIDARARPPRIAVIADGDAPLDLDTIAALSRRASALLDGLDGANKIRGRYLLEVSSPGVERPLTSEKHFRRARGRKVELVLSDGSRLTGRVGEMRAGTVALVIREDRGWAVREIPLAEIVKAVVQVEFSPPAPAELELAQSSEMGLARGTEAGA

>747|CORE_REP|Org63_Gene2945#

MSHTDLTPCTRVLASSGTVPIAEELLARVLEPYSCKGCRYLIDAQYSATEDSVLAYGNFTIGESAYIRSTGHFNAVELILCFNQLAYSAFAPAVLNEEIRVLRGWSIDDYCQHQLSSMLIRKASSRFRKPLNPQKFSARLLCRDLQVIERTWRYLKVPCVIEFWDENGGAASGEIELAALNIP

>748|CORE_REP|Org3_Gene3993#

MRLPGRHVLYALSAVTMLAACSSNGARGGIASTNMNPTNPPATAETATVSPTPAPQSARTETWINLQVGDCLADLPPADLSRITVTIVDCATAHSAEVYLRAPVAVDAAVVSMANRDCAAGFAPYTGQSVDTSPYSVAYLIDSHQDRTGADPTPSTVICLLQPANGRQPMPPSRPPPKRLPNC

>749|CORE_REP|Org119_Gene3267#

MPDDQPAVPDVDRLARSMLLLHGDHHDHNDSPEQHRTCGSWSKSRDFADDPQRAAAVREASRAERDRYLTSGLQPVDCRFCHVTVTVKRLGPGHTAVQWNTEAVAALRVLHRAAGTRRGFRTHQVLSPADRQHRTRSGRGLLGAPRPKPITSHTRLPRDTVPHPARCHRGVRFVTAVPGLSFL

>750|CORE_REP|Org59_Gene2835#

MAHSIXRTLLASGAATALIAIPTACSFSIGTSHSHSVSKAEVARQITAKMTDASGNKPESVTCPSDLPAEVGAELNCEMKIKDRTFNVNVTVTSVDGSDVKFDMVETVDKNQVANIISDKLFQRVGARPDSVXCPDNLKGVEGAKLRCRLTDGSKTYGISVIVTSVDAGDVNFDFKVDDHPE

>752|CORE_REP|Org149_Gene170#

MFDIATRFKNSYGSGPLHLLAMVSGFALLGYIVATARPSALWNQATWWQSIAVWFVAAVVAHDLLLYPLYALADRILARLVGRRDVSAPRRRPELPVRNYIRIPALAAGLTLLVFLPGIIRQGAPTYLDATGQTQEPFLGRWLLLTAVAFGISAAAYAIRLVVAHVRRRRAGCSRVDAIDEE

>753|CORE_REP|Org59_Gene189#

MLALRASFDRAAESTIAHFTFGLALLAGLYVAASPWIVGFSAXRGLPTCDLIVGIAVAYLAYGFASALDRTHGMTWTLPVLGVWVIFSPWVLPGVAVTAGMMWSHIIAGAVVAVLGFYFGMRTRAAANXGXXEVXGARGQLGNVLAGXVPARQRLXAAXXXEXAPXAAELTSLTYLDSTPVV

>754|CORE_REP|Org43_Gene1308#

MTDRTDADDLDLQRVGARLAARAQIRDIRLLRTQAAVHRAPKPAQGLTYDLEFEPAVDADPATISAFVVRISCHLRIQNQAADDDVKEGDTKDETQDVATADFEFAALFDYHLQEGEDDPTEEELTAYAATTGRFALYPYIREYVYDLTGRLALPPLTLEILSRPMPVSPGAQWPATRGTP

>755|CORE_REP|Org98_Gene565#

MAGYPRDELEDVVHRWLQANRTAERRGDWTLLADFYTDDATYGWNVGPNEDVMCVGIDEIRDIALGQEMDGLQGWRYPYQRVVIDEKQGEVVGFWKQVATDANGAEQEVYGIGGSWFRYAGGGKWNWQRDFFDFGHVSALYLELIKAGKLSPGMQKRIERAVSGNKVPGYYPLGKTPVPLW

>757|CORE_REP|Org20_Gene2639#

MAARFKALADPVRIATAELGCQSAGGEACVCDISAGVEVSQPTISHHLKVLRDAGLLTSRRRASWVYYAVVPEALTVLSNLLSVHADAAPPWGHRHDGDGHPHRRPGGGGQTLDAGPLLAGVDRVGNGRRATTGPVDSRPAHRPRRGSARRDFAADRARPADHDVSGAGQGALRPPRHRHR

>758|CORE_REP|Org1_Gene761#

MTMLSFRADDHDVDLADAWARRLHIGRSELLRDALRRHLAALAADQDVQAYTERPLTDDENALAEIADWGRRRTGPTGPMRRGELWFAATPGGDRPVLVLTRDPVADRIGAVVVVALTRTRRGLVSELELTAVENRVPSDCVVNFDNIHTLPRTAFRRRITRLSPARLHEACQTLRASTGC

>759|CORE_REP|Org2_Gene3570#

MRDEPPTDTAAAPTTGAAPEIDTAREYEVTAEYQSWRVVWGSAAALLTVGVGIGAAILLGWFTLAHRHPDQPGAAATPPPAGLTTRSAPTAAPPSTLQSPDLDSVFLGNLHDRGISFTNPDAAVYNGKMVCTNLGGGMTVQQVVEALQSSSPALGDRTTAYVAVSIRTYCPKYDAVLPPGS

>760|CORE_REP|Org138_Gene3316#

MRRVLVGAAALITALLVLTGCTKSISGTAVKAGGAGVPRNNNSQERYPNLLKECEVLTTDILAKTVGADPLDIQSTFVGAICRWQAANPAGLIDITRFWFEQGSLSNERKVAEGLKYQVETRAIQGVDSIVMRTGDPNGACGVASDAAGVVGWWVNPQAPGIDACGQAIKLMELTLATNA

>761|CORE_REP|Org1_Gene1792#

MTCPSLVGLRTEAAELSYSDQPDALGVAMRERREQQNLVRPPRRNASRRINTDQTSTKYVYITYMPETLTGRLNFRLSPEQEQALRHAAALTGQSLSGFVLSAAVDHAHDLLARANRIELSEAAFRRFVAALDEPDEAAPELVRLARRKSRIPPIEHPRARPRRAVGPGPARHGALLQRC

>762|CORE_REP|Org119_Gene660#

MSRIGKQPIPVPAGVDVTIEGQSISVKGPKGTLGLTVAEPIKVARNDDGAIVVTRPDDERRNRSLHGLSRTLVSNLVTGVTQGYTTKMEIFGVGYRVQLKGSNLEFALGYSHPVVIEAPEGITFAVQAPTKFTVSGIDKQKVGQIAANIRRLRRPDPSKRGKGVRYEGEQIRRKVGKTGK

>763|CORE_REP|Org118_Gene333#

MANILLLDFVISLVRDPEAAARYAANPERSIAEAHLTDVTRADVNSLIPVVSDSLSMSEPIGAAGGAHAGDRGNVWASGAATAALDAFAPHADAGVVQQHGAVGSVLNQPTPPGPGVTPTDPRPFRAGPHETSALLTSAEIPDTTSEDGGLPTDHPAVWNHPVVDPHTVEPDHHGYDIHG

>764|CORE_REP|Org98_Gene289#

MAGPDRAELAELVRRLSVVHGRVTLSSGREADYYVDLRRATLHHRASALIGRLMRELTADWDYSVVGGLTLGADPVATAIMHAPGRPIDAFVVRKSAKAHGMQRLIEGSEVTGQRVLVVEDTSTTGNSALTAVHAVQDVGGEVVGVATVVDRATGAAEAIEAEGLRYRSVLGLADLGLD

>765|CORE_REP|Org84_Gene1748#

MLHRDDHINPPRPRGLDVPCARLRATNPLRALARCVQAGKPGTSSGHRSVPHTADLRIEAWAPTRDGCIRQAVLGTVESFLDLESAHAVHTRLRRLTADRDDDLLVAVLEEVIYLLDTVGETPVDLRLRDVDGGVDVTFATTDASTLVQVGAVPKAVSLNELRFSQGRHGWRCAVTLDV

>766|CORE_REP|Org2_Gene4416#

MGSTPPRTPQEVFAHHGQALAAGDLDEIVADYADDSFVITPAGIARGKEGIRQLFVKLLDDIPNALWDLKTQIFEGDILFLEWTANSAVSRVDDGVDTFVFRDGTIWAHTVRYTRTPRPDVSSRWRMWTSRRSPITDQTEALLSDAGGYPQGARSSPRTKSVGCSRTLRRNRLLICRIG

>767|CORE_REP|Org15_Gene2894#

MRKRMVIGLSTGSDDDDVEVIGGVDPRLIAVQENDSDESSLTDLVEQPAKVMRIGTMIKQLLEEVRAAPLDEASRNRLRDIHATSIRELEDGLAPELREELDRLTLPFNEDAVPSDAELRIAQAQLVGWLEGLFHGIQTALFAQQMAARAQLQQMRQGALPPGVGKSGQHGHGTGQYL

>768|CORE_REP|Org68_Gene3534#

MRRQRSAVPILALLALLALLALLALIVGLGASGCAWKPPTTRPSPPNTCKDSDGPTADTVRQAIAAVPIVVPGSKWVEITRGHTRNCRLHWVQIIPTIASQSTPQQLLFFDRNIPLGSPTRNPKPYITVLPAGDDTVTVQYQWQIGSDQECCPTGIGTVRFHIGSDGKLEALGSIPHQ

>769|CORE_REP|Org118_Gene2459#

MTTARDIMNAGVTCVGEHETLTAAAQYMREHDIGALPICGDDDRLHGMLTDRDIVIKGLAAGLDPNTATAGELARDSIYYVDANASIQEMLNVMEEHQVRRVPVISEHRLVGNRHRSRHRPTPARARHCAVRQGNLLAHGPRQLATSDHPPARKALSASIGGTQCVMRSRLGGSPGLW

>770|CORE_REP|Org40_Gene551#

MARVSGAAAAEAALMRALYDEHAAVLWRYALRLTGDAAQAEDVVQETLLRAWQHPEVIGDTARPARAWLFTVARNMIIDERRSARFRNVVGSTDQSGTPEQSTPDEVNAALDRLLIADALAQLSAEHRAVIQRSYYRGWSTAQIATDLGIAEGTVKSRLHYAVRALRLTLQELGVTR

>771|CORE_REP|Org82_Gene3889#

MGTCPCESSERNEPVSRVSGTNEVSDGNETNNPAEVSDGNETNNPAEVSDGNETNNPAPVSRVSGTNEVSDGNETNNPAPVSRVSGTNEVSDGNETNNPAPVTEKPLHPHEPHIEILRGQPTDQELAALIAVLGSISGSTPPAQPEPTRWGLPVDQLRYPVFSWQRITLQEMTHMRR

>772|CORE_REP|Org129_Gene2330#

MTRLIPGCTLVGLMLTLLPAPTSAAGSNTATTLFPVDEVTQLETHTFLDCHPNGSCDFVAGANLRTPDGPTGFPPGLWARQTTEIRSTNRLAYLDAHATSQFERVMKAGGSDVITTVYFGEGPPDKYQTTGVIDSTNWSTGQPMTDVNVIVCTHMQVVYPGVNLTSPSTCAQANFS

>773|CORE_REP|Org100_Gene3633#

MHPLPADHGRSRCNRRPISPLSLIGNASATSGDMSSMTRIAKPLIKSAMAAGLVTASMSLSTAVAHAGPSPNWDAVAQCESGGNWAANTGNGKYGGLQFKPATWAAFGGVGNPAAASREQQIAVANRVLAEQGLDAWPTCGAASGLPIALWSKPAQGIKQIINEIIWAGIQASIPR

>774|CORE_REP|Org96_Gene1910#

MELVVGRVVKSHGVTGEVVVEIRTDDPADRFAPGTRLRAKGPFDGGAEGSAVSYVIESVRQHGGRLLVRLAGVADRDAADALRGSLFVIDADDLPPIDEPDTYYDHQLVGLMVQTATGEGVGVVTEVVHTAAGELLAVKRDSDEVLVPFVRAIVTSVSLDDGIVEIDPPHGLLNLE

>775|CORE_REP|Org20_Gene624#

MRSPAEWPIRWRLTLVFSAAMALVLAAAGAVTVVPVPRRRYEADPDGALRGLTDDITADLVRELVTILPIVLVIAAVAAYLLSRAALRPVDRIRAAAQTLTTTPHPDTDAPLPVPPTDDEIAWLATTLNTMLTRLQRALAHEQQFVADASHELRTPLALLTTELELRCAGPDPPTS

>776|CORE_REP|Org20_Gene1264#

MTSPFQPRQVPGSTPAAAGAGRRGVPALPTPPKGWPVGSYPTYAEAQRAVDYLSEQQFPVQQVTIVGVDLMQVERVTGRLTWPKVLGGGVLSGAWLGLFIGLVLGFFSPNPWSALVTGLVAGVFFGLITSAVPYAMARGTRDFSSTMQLVAGRYDVLCDPQNAEKARDLLARLAI

>777|CORE_REP|Org59_Gene1518#

MSAYKTVVVGTDGSDSSMRAVDRAAQIAGADAKLIIASAYLPQHEDARAADILKDESYKVTGTAPIYEILHDAKERAHNAGAKNVEERPIVGAPVDALVNLADEEKADLLVVGNVGLSTIAGRLLGSVPANVSRRAKVDVLIVAAPPSGRYQPRARHSLRLGRSAPSSVSSPWPG

>778|CORE_REP|Org59_Gene1324#

MATIGEVEVFVDHGADDVFXTXPLWIGTRQADRLRQLADRARIAVGAGTAEGASNTGARLADAAGAIDVLIEIDSGHHRSGVRAEQVLEVAHAVGEAGLHLVGVFTFPGHSYAPGKPGEAGEQERQRSQRRGERXXRGGXPDQLPQRXVXSXNRDXITAADGXCRXFXPXVXRAR

>779|CORE_REP|Org1_Gene3361#

MYDEVDAGDGPGKGVMMPTEYPATAEESVDVITDALLTASRLLVAISAHSIAQVDENITIPQFRTLVILSNHGPINLATLATLLGVQPSATGRMVDRLVGAELIDRLPHPTSRRELLAALTKRGRDVVRQVTEHRRTEIARIVEQMAPAERHGLVRALTAFTEAGGEPDARYEIE

>780|CORE_REP|Org36_Gene1822#

MTERPRDCRPVVRRARTSDVPAIKQLVDTYAGKILLEKNLVTLYEAVQEFWVAEHPDLYGKVVGCGALHVLWSDLGEIRTVAVDPAMTGHGIGHAIVDRLLQVARDLQLQRVFVLTFETEFFARHGFTEIEGTPVTAEVFDEMCRSYDIGVAEFLDLSYVKPNILGNSRMLLVL

>781|CORE_REP|Org64_Gene341#

MPRSFDMSADYEGSVEEVHRAFYEADYWKARLAETPVDVATLESIRVGGDSGDDGTIEVVTLQMVRSHNLPGLVTQLHRGDLSVRREETWGPVKEGIATASIAGSIVDAPVNLWGTAVLSPIPESGGSRMTLQVTIQVRIPFIGGKLERLIGTQLSQLVTIEQRFTTLWITNNV

>782|CORE_REP|Org82_Gene2976#

MGAQRASMQRPAADTPDGFGVAVVREEGRWRCSPMGPKALTSLRAAETELRELRSAGAVFGLLDVDDEFFVIVRPAPSGTRLLLSDATAALDYDIAAEVLDNLDAEIDPEDLEDADPFEEGDLGLLSDIGLPEAVLGVILDETDLYADEQLGRIAREMGFADQLSAVIDRLGR

>783|CORE_REP|Org57_Gene1892#

MSLRLVSPIKAFADGIVAVAIAVVLMFGLANTPRAVAADERLQFTATTLSGAPFDGASLQGKPAVLWFWTPWCPFCNAEAPSLSQVAAANPAVTFVGIATRADVGAMQSFVSKYNLNFTNLNDADGVIWARYNVPWQPAFVFYRADGTSTFVNNPTAAMSQDELSGRVAALTS

>784|CORE_REP|Org63_Gene2809#

MRRWLIVLATLLVAAAGVAAANDVPRAWAGDAPIGHIGDTLRVDTGTYVADVTVSSVVPVDPPPGFGYTRSGVPVKSFPDSSVTRADVTVRAVRVPNSFILATNFSFTGVTPFADAYKPRPCDASDWLDAALGNAPQGSIVRGGVYWDAYRDPVSVVVLLDEKTGQHLAQWNL

>785|CORE_REP|Org6_Gene2339#

MRFDQLVRIVNAADPFSINDLGCGYGALLDYLDARGFKTDYTGIDVSPEMVRAAALRFEGRANADFICAARIDREADYSVASGIFNVRLKSLDTEWCAHIEATLDMLNAASRRGFSFNCLTSYSDASKMRDDLYYADPCALFDLCKRRYSKSVALLHDYGLYEFTILVRKAS

>786|CORE_REP|Org118_Gene3534#

MIVGAFLAEAASVVDNKLNVSGGVLYRFAVDPDRSAQFLLVVLTQAETDDPDRRVDVEVWPPTGDDAHHIEFELPEAAVAAEVGFAIFRIEVNLPVDGRWVLVVTGGAGTISLPADRDGVRRRPLPTELPALLIEWEQDAEAEWRSGREQTPCLSGELWGPARPAHLAGCAA

>787|CORE_REP|Org13_Gene407#

MISPKPLLHILIHGRSDELPDTRGRIVLRWLRIAVLIVTGLVTLQSVLLVAGAWRNDIAIQRNMGVAQAEVLSAGPRRSTIEFVTPDRITYRPQLGVLYPSELSTGMRIYVEYNKRDPNLVRVQHRNAGLAIIPAGSIAVVAWLIAAAALVVLAVLDKRLERRENSASATG

>788|CORE_REP|Org40_Gene2928#

MRAWLAAATTALFVVATGCSSATNVAELKVGDCVKLAGTPDRPQATKAECGSPASNFKVVAVVQEDHAECPADVDSTYSMRNAFNGSTNTICLDIDWVIGGCMSVDPTHNTDPFRVDCDDASVPHRQRATQILKDLDSPVSVDQCASGVGYVYTQRRFAVCVEDVTGGPRS

>789|CORE_REP|Org128_Gene1296#

MSCTFDMVPETVDHLDEVGLRRVFGCFPCGVIAVCAMVDDQPVGMAASSFTSVSVDPPLVSICVQNCSTTWPKLRDRPRLGVSVLAEGHDAACMSLSRKEGNRFAGVFWSELSSGGVVIAGAGAWLDCRPYAEIPAGDHLIALLEICAVRADPETPPLVFHGSRFRRLESR

>790|CORE_REP|Org56_Gene2653#

MGEVSAIVLAASQAAEEGGESSNFLIPNGTFFVVLAIFLVVLAVIGTFVVPPILKVLRERDAMVAKTLADNKKSDEQFAAAQADYDEAMTEARVQASSLRDNARADGRKVIEDARVRAEQQVASTLQTAHEQLKRERDAVELDLRAHVGTMSATLASRILGVDLTASAATR

>791|CORE_REP|Org119_Gene2391#

MESFVLFLPFLLIMGGFMYFASRRQRRAMQATIDLHDSLQPGERVHTTSGLEATIVAIADDTIDLEIAPGVVTTWMKLAIRDRILPDDDIDEELNEDLDKDVDDVAGEPPGDQRFLTGNRPTGTDEPLARRAPARHVGSVGGRKLILEEIQGTWHRLRRRCTLPVTCRCSW

>792|CORE_REP|Org59_Gene201#

MTXAAEPHPAPPQQPTVAWSEPDVDRRVEFWPTVAIRSALESGDIATWQRIAAALXXDPYGRTARQVEEVLEGIPATGIANAFWEVLDRARTHLDANERAEVARQVGLLLDRSGLQRQEFASRIGVTAQDLTAYLDGIVSPSASLMIRMRQXCRTGSSEPNPYVPPTXSER

>793|CORE_REP|Org64_Gene1278#

MPSGWVSHRLGGSPKCISALSLPSGTVGAPSKPDNDATRGRTRPTVPPPDPAAMGTWKFFRASVDGRPVFKKEFDKLPDQARAALIVLMQRYLVGDLAAGSIKPIRGDILELRWHEANNHFRVLFFRWGQHPVALTAFYKNQQKTPKTKIETALDRQKIWKRAFGDTPPI

>794|CORE_REP|Org31_Gene202#

MGTRSKSRTRQLKQSNGCTATTSGASDRRRRARRRTAPAWLREDEWLRHHLPHPPRQLSRCLHRRRRSACHHRYSRRTPKGGLPMTSSLVPISEARAHLSRLVRESADDDVVLMNHGRPAAILISAERYESLMEELEDLRDRLSVHEREHVTMPLDKLGAELGVDIGRV

>795|CORE_REP|Org95_Gene2117#

MTQVYIPATLAMLQRLVADGALWPVNGTAFAVTPTLRESYAEGDDEELAEVALREAALASLRLLAADIGATADALPPRRAVLAAEVDDATYRPDLDDAVVRLAGPITIDQVVAAYVDNAGAEPAVMAAIAVIDAADLGDEDAELVVGDAQDHDLAWYANQELPFLLDLL

>796|CORE_REP|Org139_Gene3036#

MPCCGSLTRAPIGLCGRRTSWPRLGEPWSTASTSAPNGLTTAFAFGYNDLIAAMNNHYKDRHVLAAAVRERAEVIVTTNLKHFPDDALKPYQIKALHPDDFLLDQLDLYEEATKAVILGMVDAYIDPPFTPHSLLDALGEQVPQFAAKARRLFPSGSPFGLGVLLPFDQ

>797|CORE_REP|Org20_Gene3245#

MHDSLSVVNVPRRCCRPGCPHYAVATLTFVYSDSTAVIGPLATAREPHSWDLCVGHAGRITAPRGWELVRHAGPLPSHPDEDDLVALADAVREGGPSAGRRHHPGGNGAPLHGFHIRDGDRSAHRGWRACAARAWGRAPARTSTGVARPRRLVFPCCSRPIRPASFAGR

>798|CORE_REP|Org83_Gene2228#

MRPVDEQWIEILRIQALCARYCLTIDTQDGEGWAGCFTEDGAFEFDGWVIRGRPALREYADAHARVVRGRHLTTDLLYEVDGDVATGRSASVVTLATAAGYKILGSGEYQDRLIKQDGQWRIAYRRLRNDRLVSDPSVAVNVADADVAAVVGHLLAAARRLGTQMSDT

>799|CORE_REP|Org143_Gene2241#

MSREGIRRRPKARAGLTGGGTATLPRVEDTLTLGSRLGEQLCAGDVVVLSGPLGAGKTVLAKGIAMAMDVEGPITSPTFVLARMHRPRRPGTPAMVHVDVYRLLDHNSADLLSELDSLDLDTDLEDAVVVVEWGEGLAERLSQRHLDVRLERVSHSDTRIATWSWGRS

>800|CORE_REP|Org12_Gene548#

METLLKTSEAAQILGVSRQHVVNMCDRGEMVCVHVGSHRRVPSSEVERVTSRRLTREEERSLWLHRALLSPLLTEPDTVVSAARENLRRWSGMHRRDGMAGWYFTKWQRVLNDGLDAVMHVLTSPSEDAREMRQNSPFAGILPEATRVAVLRSFKDHWDREHERAMTE

>801|CORE_REP|Org6_Gene2892#

MFETPLTVVGHIVNDLQRRKVGDQEVVKFRVASNSRRRTSDGGWEPGNSLFITVNCWGRLVTGVGAALGKGAPVIVVGHVYTSEYEDRDGIRRSSLEMRATSVGPDLSRVIVRIEKPAYTGPSAGDLPAATGTGAAGAADAPASAADSVSDVVVDDAITGHNPLPISA

>802|CORE_REP|Org131_Gene1485#

MSGHRKKAMLALAAASLAATLAPNAVAAAEPSWNGQYLVTLSANAKTGTSMAANRPEYPHKANYTFSSRCASDVCIATVVDAPPPKNEFIPRPIEYTWNGTQWVREISWQWDCLLPDGTIEYAPAKSITAYTPGQYGILTGVFHTDIASGTCKGNVDMPVSAKPIVG

>803|CORE_REP|Org59_Gene883#

MLVAGTPQPRALGPDALDVSTDDLAGLLAGNTGRIKTVITDQKVIAGIGNAYSDEILHVAKISPFATAGKLSGAQLXCLHEAMASVLSDAVRRSVGQGAAMLKGEKRSGLRVHARTGLPCPVCGDTVREVSFADKSFQYCPTCQTGGKALADRRMSRLLKXSICSPE

>804|CORE_REP|Org2_Gene4061#

MARLVGVDLPRDKRMEVALTYIFGIGRTRSNEILAATGIDRDLRTRDLTEEQLIHLRDYIEANLKVEGDLRREVQADIRRKIEIGCYQGLRHRRGMPVRGQRTKPTRGPAKAPSAPSQARRRLGNRCHQQKKGRQRRLGRARRPAGGRRRTSRTAPPTSRARSTTRS

>805|CORE_REP|Org118_Gene2596#

MRRLLIVHHTPSPHMQEMFEAVVSGATDPEIEGVEVVRRPALTVSPIEMLEADGYLLGTPANLGYISGALKHAFDVCYYPCLDTTRGRSFGAYIHGNEGTEGAERAVDAITTGLGWVQAAETVVVMGKPSKADIEACWNLGGNRRCAVNGMIWAPSTPWRAWCLRWR

>806|CORE_REP|Org17_Gene657#

MSGYSAPRRISDADDVTSFSSGEPSLDDYLRKRALANHVQGGSRCFVTCRDGRVVGFYALASGSVAHADAPGRVRRNMPDPVPVILLSRLAVDRKEQGRGLGSHLLRDAIGRCVQAADSIGLRAILVHALHDEARAFYVHFDFEISPTDPLHLMLLMKDARALIGD

>807|CORE_REP|Org119_Gene1824#

MIYLETSALVKLIRIEVESDALADWLDDRTELRWITSALTEVELSRAIRAVSPEGLPAVPSVLARLDRFEIDAVIRSTAAAYPNPALRSLDAIHLATAQTAGSVAPLTALVNRPKTALRKPPRPCRWRSSHPDKRDEAGTRSLLSRWLSYGRPWSPPALDGLVGYP

>808|CORE_REP|Org117_Gene1268#

MAQITLRGNAINTVGELPAVGSPAPAFTLTGGDLGVISSDQFRGKSVLLNIFPSVDTPVCATSVRTFDERAAASGATVLCVSKDLPFAQKRFCGAEGTENVMPASAFRDSFGEDYGVTIADGPMAGLLARAIVVIGADGNVAYTELVPEIAQEPNYEAALAALGA

>809|CORE_REP|Org92_Gene2909#

MTPVRPPHTPDPLNLRGPLDGPRWRRAEPAQSRRPGRSRPGGAPLRYHRTGVGMSRTGHGSRPVPPATTVGLALLAAAITLWLGLVAQFGQMITGGSADGSADSTGRVPDRLAVVRVETGESLYDVAVRVAPNAPTRQVADRIRELNGLQTPALAVGQTLIAPVG

>810|CORE_REP|Org117_Gene484#

MVEKPLRADRATHSRLATFALALAAAALPLAGCSSTANPPAATTTPATATTTTATSGPTAAPTVTTGESTTASIQIGDMLTYGSIGTTATLDCADGKSLNVAGSDNTLTVNGTCETVTVGGANNKIAFDRIDERLVVVGLDNTVTYKNGDPTIDNLGAGNRINKE

>811|CORE_REP|Org108_Gene2623#

MTQLPQPTWRWWQQRETEQVQSSHIDGEIVGALIPDLAVLHSEDASRAAVGREKHRCSLDPLGGGFRSRRASMPAGALLLSAVIAIQLDRMNARVFGDGWIGAQACMWVNKFHEESTVTALSPSSPIAQGSIARHPETMQSAYVRIAEGGSRDVAPAAQLQRRRP

>812|CORE_REP|Org135_Gene2632#

MNSGTLAGSLIFAAVLVMLIAVLARLMMRGWRRRSERQAELLGDLPDVPEHVSSATVTTRGLYVGATLSPAWNERVTVGDLGYRSKAVLTRYPSGIMVERARAQPIWIPTESIAAIRMERGVAGKVVAGIGILAIRWRLPSGTEIDVGFRADNRDEYQEWLEEPV

>813|CORE_REP|Org2_Gene1386#

MVVLALLTVDGVVSALAGALLMPWYIGSAPFPISALISGLVNAALVWAAARWTTSSRVAALPLWAWLLTVAAMSFGGPGDDVILGGQGLLVYGALVFVVAGPCHRRGCCGGAGSKLTDLASPKLGQRRESRRADWRQRRQEAAYPVDLGVDELVDRCRGGRFDIG

>814|CORE_REP|Org59_Gene56#

MAGXTTITJVGNLTADPELRFTPSGAAVANFTVASTPRIYDRQTGEWKDGEALFLRCNIWREAAENVAESLTRGARVIVSGRLKQRSFETREGEKRTVIEVEVDEIGPSLRXXXAKVNKXXTAAAGLAADPVRRRRRPAXASGDDPWGSAPASGSFGGGDDEPPF

>815|CORE_REP|Org25_Gene2758#

MFLPTNAQYQLLVVGVSPWDTPSPSGRISWGSAWPHQARRAQTCQRVRRHWMIDTTEAAYRLTYQPDGTSITVRENLVDILARELLGPIRGPQEVLPFSPRSQYLVGHLAPVKLTGAALIDDNAVQARANAEALAEGGGVPAYAADETTPTPTTTPKTAHPSRA

>816|CORE_REP|Org140_Gene763#

MTDTQVTWLTQESHDRLKAELDQLIANRPVIAAEINDRREEGDLRENGGYHAAREEQGQQEARIRQLQDLLSNAKVGEAPKQSGVALPGSVVKVYYNGDKSDSETFLIATRQEGVSDGKLEVYSPNSPLGGALIDAKVGETRSYTVPNGSTVSVTLVSAEPYHS

>817|CORE_REP|Org104_Gene1322#

MLSKSKRSCRRRETLRIGEKMSAPITNLQAAQRDAIMNRPAVNGFPHLAETLRRAGVRTNTWWLPAMQSLYETDYGPVLDQGVPLIDGVAEVPAFDRTALVTALRADQAGQTSFREFAAAAWRAGVLRYVVDLENRTCTYFGLHDQTYMEHYAAVEPSGGAPTS

>818|CORE_REP|Org118_Gene2183#

MSPSPAAANRSEVGGPLPGLGADLLAVVARLNRLATQRIQMPLPAAQARLLATIEAQGEARIGDLAAVDHCSQPTMTTQVRRLEDAGLVTRTADPGDARAVRIRITPEGIRTLTAVRADRAAAIEPQLALLPPADRRVLADAVDVLRRLLDHAATTPGRATRQ

>819|CORE_REP|Org60_Gene1112#

MPEVTREEPAIDGWFTTDKAGNPHLLGGKCPQCGTYVFPPRADNCPNPACGSDTLESVGLSTRGKLWSYTENRYAPPPPYPAPDPFEPFAVAAVELADEGLIVLGKVVDGTLAADLKVGMEMELTTMPLFADDDGVQRIVYAWRIPSRAGDDAERSDAEERRR

>820|CORE_REP|Org88_Gene236#

MCCNGVVTPGDPADIAAIKQLKYRYLRALDTKHWDDFTDTLAEDVTGDYGSSVGTELHFTNRADLVDYLRQALGPGVITEHRVTHPEITVTGDTATGIWYLQDRVIVAEFNFMLIGAAFYHDQYRRTTDGWRISATGYDRTYEATMSLAGLNFNIRPGRALAD

>821|CORE_REP|Org25_Gene421#

MADSSFDIVSKVDRQEVDNALNQAAKELATRFDFRGTDTKIAWKGDEAVELTSSTEERVKAAVDVFKEKLIRRDISLKAFEAGEPQASGKTYKVTGALKQGISSENAKKITKLIRDAGPKNVKTQIQGDEVRVTSKKRDDLQAVIAMLKKADLDVALQFVNYR

>822|CORE_REP|Org118_Gene2018#

MLIIALVLALIGLLALVFAVVTSNQLVAWVCIGASVLGVALLIVDALRERQQGGADEADGAGGKGVSRRKPTSTTRRKPPRRAKPSTPVSSAVRSHRRRPAKRPRSRRYRRTEATTAPSRVVSDTDLGRGVVGGYRVVCRPAPDLDPRPRILTIPVTSRESSR

>823|CORE_REP|Org119_Gene3352#

MQFDVTIEIPKGQRNKYEVDHETGRVRLDRYLYTPMAYPTDYGFIEDTLGDDGDPLDALVLLPQPVFPGVLVAARPVGMFRMVDEHGGDDKVLCVPAGDPRWDHVQDNPGTFRLSSWMRSSISLWHYKDLEPGKFVKAADWVDRAEAEAEVQRSVERFKAGTH

>824|CORE_REP|Org118_Gene457#

MSSEEKLAAKVSTKASDVASDIGSFIRSQRETAHVSMRQLAERSGVSNPYLSQVERGLRKPSADVLSQIAKALRVSAEVLYVRAGILEPSETSQVRDAIHHRYGDHRASEADSARYLTRHLPTRTKPPGRSVRAIRHRPMTSRWPAVLRTGWRVIKPEGQSSG

>825|CORE_REP|Org1_Gene3954#

MAITGSAAPSWPRLLHAEGPPSVICIRLLVGLVFLSEGIQKFMYPDQLGPGRFERIGIPAATFFADLDGVVEIVCGTLVLLGLLTRVAAVPLLIDMVGAIVLTKLRALQPGGFLGVEGFWGMAHAARTDLSMLLGLIFLLWSGPGRWSLDRRLSKRATACGAR

>826|CORE_REP|Org2_Gene3656#

MLKVTVTARSPGSSRQPGHRPAAALAAPYDGSWSELNVYIPILVLAALAAAFAVVSVVIASLVGPSRFNRSKQAAYECGIEPASTGARTSIGPGAASGQRFPIKYYLTAMLFIVFDIEIVFLYPWAVSYDSLGTFALVEMAIFMLTVFVAYAYVWRRGGLTWD

>827|CORE_REP|Org142_Gene3842#

MNSTNNLTPSSLREAFGHFPTGVVAIAAEVDGVRQGLAASTFVPVSLEPPLVSFCVQNTSTTWPKLTGVPMLGISVLGEAHDAAVRTLAAKTGDRFAGLETVFNDAGAVFIKGTSVWLESAIEQLVPAGDHTIVVLRVNQVKVDPNVAPIVFHRSVLRRLGV

>828|CORE_REP|Org68_Gene1008#

MTLRLEQIYQDVILDHYKHPQHRGLREPFGAQVYHVNPICGDEVTLRVALSEDGTRVTDVSYDGQGCSISQAATSVLTEQVIGQRVPRALNIVDAFTEMVSSRGTVPGDEDVLGDGVAFAGVAKYPARVKCALLGWMAFKDALAQASEAFEEVTDERNQRTG

>829|CORE_REP|Org1_Gene4076#

MIFKVGDTVVYPHHGAALVEAIETRTIKGEQKEYLVLKVAQGDLTVRVPAENAEYVGVRDVVGQEGLDKVFQVLRAPHTEEPTNWSRRYKANLEKLASGDVNKVAEVVRDLWRRDQERGLSAGEKRMLAKARQILVGELALAESTDDAKAETILDEVLAAAS

>830|CORE_REP|Org90_Gene2978#

MSLAWDVVSVDKPDDVNVVIGQAHFIKAVEDLHEAMVGVSPSLRFGLAFCEASGPRLVRHTGNDGDLVELATRTALAIAAGHSFVIFLREGFPINILNPVQAVPEVCTIYCATANPVDVVVAVTPHGRGIVGVVDGQTPLGVETDRDIAQRRDLLRAIGYKL

>831|CORE_REP|Org59_Gene157#

MYNELLENLAILVLSGFVGFAVISKVPNTLHTPLMSGTNAIHGIVVLGALVVFGEIEHPSLVLQVILFVAVVFGTLNVIGGFIVTDRMLGMXKAKXXPPCQPSPTATRRSDEPALPGRDSLHHLLFTLHLRVDGAHRPQDRGAREPDRRGRHDXPPWRPRWS

>832|CORE_REP|Org89_Gene146#

MTAISCSPRPRYASRMPVLSKTVEVTADAASIMAIVADIERYPEWNEGVKGAWVLARYDDGRPSQVRLDTAVQGIEGTYIHAVYYPGENQIQTVMQQGELFAKQEQLFSVVATGAASLLTVDMDVQVTMPVPEPMVKMLLNNVLEHLAENLKQRAEQLAAS

>833|CORE_REP|Org36_Gene1960#

MTGAVCPGSFDPVTLGHVDIFERAAAQFDEVVVAILVNPAKTGMFDLDERIAMVKESTTHLPNLRVQVGHGLVVDFVRSCGMTAIVKGLRTGTDFEYELQMAQMNKHIAGVDTFFVATAPRYSFVSSSLAKEVAMLGGDVSELLPEPVNRRLRDRLNTERT

>834|CORE_REP|Org28_Gene1772#

MSGTRLAPHSVRYRERLWVPWWWWPLAFALAALIAFEVNLGVAALPDWVPFATLFTVAAGTLLWLGRVEIRVTAGSADGAGVKLWAGPAHLPVAVIARSAEIPATAKSAALGRQLDPAAYVLHRAWVGPMVLVVLDDPNDPTPYWLVSCRHPERVLSALRS

>835|CORE_REP|Org96_Gene466#

MSDEGDVADEAVADGAENADSRGSGGRTALVTKPVVRPQRPTGKRSRSRAAGADADVDVEEPSTAASEATGVAKDDSTTKAVSKAARAKKASKPKARSVNPIAFVYNYLKQVVAEMRKVIWPNRKQMLTYTSVVLAFLAFMVALVAGADLGLTKLVMLVFG

>836|CORE_REP|Org1_Gene2827#

MSLPSPSDCIAGLAASRLVRVLVQRVSSAAVRVDGRVVGAIRPDGQGLVAFVGVTHGDDLDKARRLAEKLWNLRVLADEKSASDMHAPILVISQFTLYADTAKGRRPSWNAAAPGAVAQPLIAAFAAALRQLGAHVEAGVFGAHMQVELVNDGPVTVMLEG

>837|CORE_REP|Org44_Gene2040#

MSKSSRGGRQIVASNRKARHNYSIIEVFEAGVALQGTEVKSLREGQASLADSFATIDDGEVWLRNAHIPEYRHGSWTNHEPRRNRKLLLHRRQIDTLVGKIREGNFALVPLSLYFAEGKVKVELALARGKQARDKRQDMARRDAQREVLRELGRRAKGMT

>838|CORE_REP|Org46_Gene2487#

MVSLLVHAALGVVVIGWIVSSNPKVFTRPAGGSWFSLPECVYYVVGIASIALGWYFNIRFVQQYAHGAANPLWGPGSWAEYVRLMFTNPAASSAGQDYTIANVILLPLFSTTDGYRRGLRRPWLYFVSSLFTSFAFAFAFYFATIERQHRHERSRATVGA

>839|CORE_REP|Org88_Gene2659#

MKGGAGVPDLPSLDASGVRLAIVASSWHGKICDALLDGARKVAAGCGLDDPTVVRVLGAIEIPVVAQELARNHDAVVALGVVIRGQTPHFDYVCDAVTQGLTRVSLDSSTPIANGVLTTNTEEQALDRAGLPTSAEDKGAQATVAALATALTLRELRAHS

>840|CORE_REP|Org104_Gene2051#

MKRLIALGIFLIVGIELLALILHDRRLVLAGSGLALALVLLNVRRMLGNRDELTAAPDSDDLGEGLRRWLSNTETTIRWSESTRADWDRHLRPMLARRFEIATGHRQAKDPVAFAATGRMLFGDELWEWVNPNNVTHTGDRQPGPGRAALEEILQKLEQV

>841|CORE_REP|Org86_Gene2427#

MKRGLTVAVAGAAILVAGLSGCSSNKSTTGSGETTTAAGTTASPGAASGPKVVIDGKDQNVTGSVVCTTAAGNVNIAIGGAATGIAAVLTDGNPPEVKSVGLGNVNGVTLGYTSGTGQGNASATKDGSHYKITGTATGVDMANPMSPVNKSFEIEVTCS

>842|CORE_REP|Org37_Gene1293#

MKLTTMIKTAVAVVAMAAIATFAAPVALAAYPITGKLGSELTMTDTVGQVVLGWKVSDLKSSTAVIPGYPVAGQVWEATATVNAIRGSVTPAVSQFNARTADGINYRVLWQAAGPDTISGATIPQGEQSTGKIYFDVTGPSPTIVAMNNGMEDLLIWEP

>843|CORE_REP|Org37_Gene1268#

MQGDPDVLRLLNEQLTSELTAINQYFLHSKMQDNWGFTELAAHTRAESFDEMRHAEEITDRILLLDGLPNYQRIGSLRIGQTLREQFEADLAIEYDVLNRLKPGIVMCREKQDTTSAVLLEKIVADEEEHIDYLETQLELMDKLGEELYSAQCVSRPPT

>844|CORE_REP|Org59_Gene501#

MTTTIPTSKSACSVTTRPGNAAVDYGGAQIRAYLHHLATVVTIRGEIDAANVEQISEHVRRFSLGTNPMVLDLSELSHFSGAGISLLCILXEDCRAAGVQWALVASPAVVEQLGGRCDQGEHESMFPMARSVHKALHDLADXIXRRRQLVLPLISRSA

>845|CORE_REP|Org4_Gene3127#

MVGRAVPSPNRRYRRVWPPRTKGQHLSNPYAQHQLKLIRHTGALILWQQRTYVVSGTREQCEAAYKSAQTYNLLVGWWSLVSLLAMNWIALISNFNAIRRVRAAADGASVPHGPHAIAHPAVPRGPIPAGWYPDPSGAGLRYWDGATWTHWTHPPRHR

>846|CORE_REP|Org97_Gene2359#

MGPTRKRDLTAAVVGAAAVGYLLVAVLYRWFPPITVWTGLSLLAVAVAEALWARYVRVKISDGEIGDGPGWLHPLVVARSLMVAKASAWVGALVTGWWIGVLAYFLPRRSWLRAAAEDTTGTVVAAGSALALVVAALWLQHCCKSPQDPTEHADGAES

>847|CORE_REP|Org58_Gene464#

MALSADIVGMHYRYPDHYEVEREKIREYAVAVQNDDAWYFEEDGAAELGYKGLLAPLTFICVFGYKAQAAFFKHANIATAEAQIVQVDQVLKFEKPIVAGDKLYCDVYVDSVREAHGTQIIVTKNIVTNEEGDLVQETYTTLAGRAGEDGEGFSDGAA

>848|CORE_REP|Org31_Gene2240#

MTADTEPVTIGALTRADAQRCAELEAQLFVGDDPWPPAAFNRELASPHNHYVGARSGGTLVGYAGISRLGRTPPFEYEVHTIGVDPAYQGRGIGRRLLRELLDFARGGVVYLEVRTDNDAALALYRSVGFQRVGLRRRYYRVSGADAYTMRRDSGDPS

>849|CORE_REP|Org111_Gene1482#

MPIATVCTWPAETEGGSTVVAADHASNYARKLGIQRDQLIQEWGWDEDTDDDIRAAIEEACGGELLDEDTDEVIDVVLLWWRDGDGDLVDTLMDAIGPLAEDGVIWVVTPKTGQPGHVLPAEIAEAAPTAGLMPTSSVNLGNWSASRLVQPKSRAGKR

>850|CORE_REP|Org7_Gene3019#

MRGPRWQKVRSSSVKLSNQKRHWPGYLFGRIRTSTLVLIAAFLAVWWIYETYRPQAPGPGDSPPTQVVPPGFVPDPDYTWVPRTRVQPPTVKATPTTTSSTPPVSPPETTTDSAVPPPFELPPPFGPGTTTPTPPAPLPQPGPGPTAGTYPKSEPPTR

>851|CORE_REP|Org119_Gene3560#

MAISFRPTADLVDDIGPDVRSCDLQFRQFGGRSQFAGPISTVRCFQDNALLKSVLSQPSAGGVLVIDGAGSLHTALVGDVIAELARSTGWTGLIVHGAVRDAAALRGIDIGIKALGTNPRKSTKTGAGERDVEITLGGVTFVPGDIAYSDDDGIIVV

>852|CORE_REP|Org101_Gene1661#

MTKTTRLTPGDKAPAFTLPDADGNNVSLADYRGRRVIVYFYPAASTPGCTKQACDFRDNLGDFTTAGLNVVGISPDKPEKLATFRDAQGLTFPLLSDPDREVLTAWGAYGEKQMYGKTVQGVIRSTFVVDEDGKIVVAQYNVKATGHVAKLRRDLSV

>853|CORE_REP|Org1_Gene1044#

MRSISVTTMTRRLRPGWLVALSAAVIAASTWMPWLTTTVGGGGWVNAIGGTHGSLELPHGFGPGQLIVLLSSTLLVVGAMAGRGLSVKLSSIAALVVSLLIVALTVWYYKLNVNPPVSAEYGLYFGAAGGVCAVGCSLWAAVSAASPGRRRHREVVR

>854|CORE_REP|Org1_Gene1444#

MPLRKTDPMDISRWLERHVGVQLLRLHDAIYRGTNGRIGHRIPGAPPSLLLHTTGAKTSQPRTTSLTYARDGDAYLIVASKGGDPRSPGWYHNLKANPDVEINVGPKRFGVTAKPVQPHDPDYARLWQIVNENNANRYTNYQSRTSRPIPVVVLTRR

>855|CORE_REP|Org43_Gene3277#

MRLKPAPSPAAAFAVAGLILAGWAGSVGLAGADPEPAPTPKTAIDSDGTYAVGIDIAPGTYSSAGPVGDGTCYWKRMGNPDGALIDNALSKKPQVVTIEPTDKAFKTHGCQPWQNTGSEGAAPAGVPGPEAGAQLQNQLGILNGLLGPTGGRVPQP

>856|CORE_REP|Org118_Gene1911#

MATTLPVQRHPRSLFPEFSELFAAFPSFAGLRPTFDTRLMRLEDEMKEGRYEVRAELPGVDPDKDVDIMVRDGQLTIKAERTEQKDFDGRSEFAYGSFVRTVSLPVGADEDDIKATYDKGILTLCRWRFRKGSQPKSTFRSGPPTDHWVRADDRSG

>857|CORE_REP|Org119_Gene3404#

MKTQDKLRTATIRMLLAAIQTEEVSGKQARELSDDEVIKVLARESRKRGEAAEIYTQNGRGELAATEHAEARIIDEYLPTPLTEGELADVADTAIAEVAEELGHRPSMKQMGLVMKAATVIAAGKADGARLSAARSRNAYRPISTEAICVSALEGC

>858|CORE_REP|Org118_Gene615#

MAPKKKVAGLIKLQIVAGQANPAPPVGPALGQHGVNIMEFCKAYNAATENQRGNVIPVEITVYEDRSFTFTLKTPPAAKLLLKAAGVAKGSAEPHKTKVAKVTWDQVREIAETKKTDLNANDVGPERPRSSPVPLGRWASPSNRALPVGGPASAR

>859|CORE_REP|Org134_Gene1575#

MTPGLLTTAGAGRPRDRCARIVCTVFIETAVVATMFVALLGLSTISSKADDIDWDAIAQCESGGNWAANTGNGLYGGLQISQATWDSNGGVGSPAAASPQQQIEVADNIMKTQGPGAWPKCSSCSQGDAPLGSLTHILTFLAAETGGCSGSRDD

>860|CORE_REP|Org57_Gene2749#

MSGRKFSFEVTKTSSAPAATLFRLVTDGGNWATWAKPIVAQSSWARRGDPAPGGIGAIRKLGMWPVFVQEETVEYEQDRRHVYKLVGARTPVQDYFGEVVLTPNASGGTDLRWSGSFTEKVRGTGPVMRAALGGAVRFFAGQLVKAAEREAVRR

>861|CORE_REP|Org1_Gene2487#

MQNGSLTRMPGRAPGSTLARVGSIPAGDDVLDPDEPTYDLPRVAELLGVPVSKVAQQLREGHLVAVRRAGGVVIPQVFFTNSGQVVKSLPGLLTILHDGGYRDTEIMRWLFTPDPSLTITRDGSRDAVSNARPVDALHAHQAREVVRRAQAMAY

>862|CORE_REP|Org79_Gene1778#

MSTTLAIVRLDPGLPLPSRAHDGDAGVDLYSAEDVELAPGRRALVRTGVAVAVPFGMVGLVHPRSGLATRVGLSIVNSPGTIDAGYRGEIKVALINLDPAAPIVVHRGDRIAQLLVQRVELVELVEVSSFDEAGLASTSRGDGGHGSSGGHASL

>863|CORE_REP|Org89_Gene1093#

MKTGDTVADFELPDQTGTPRRLSVLLSDGPVVLFFYPAAMTPGCTKEACHFRDLAKEFAEVRASRVGISTDPVRKQAKFAEVRRFDYPLLSDAQGTVAAQFGVKRGLLGKLMPVKRTTFVIDTDRKVLDVISSEFSMDAHADKALATLRAIRSG

>864|CORE_REP|Org113_Gene2785#

MLYSFDTSAILNGRRDLFRPAVFRSLWGRVEDAISAGQIRSVDEVQRELARRDDDAKRWADGQTGLFCPLDEQIQQAARHILRLHPNMVRQGGRRSAADPFVIALAMVNNATVVTQETASGNIEKPRIPDVCDALGVPWLTLMGYIEAQGWTF

>865|CORE_REP|Org147_Gene642#

MAINVEPALSPHLVVDDAASAIDFYVKAFDAVELGRVPGPDGKLIHAALRINGFTVMLNDDVPQMCGGKSMTPTSLGGTPVTIHLTVTDVDAKFQRALNAGATVVTALEDQLWGDRYGVVADPFGHHWSLGQPVREVNMDEIQAAMSSQGDG

>866|CORE_REP|Org118_Gene2390#

MLAAAVLAWMGVLCVCDVRQRRLPNWLTLPGAGVILLFAGLAGRGVPALAGAAALAGVYLLVHLALPAAMGAGDVKLAIGLGGLTGCFGVEVWFLAALAAPLLTGGVRRDGDAMGCPHPAARAVDVCGQPGGGGVGAAGLRLARYQCRPEVV

>867|CORE_REP|Org119_Gene1386#

MSVGEVEVLKVENSRVRAEQLAKLYELRSSRDRVRVDAALAELSRAAAARGCAGTSGLGNNLMAPGPPHSLLGRDRCSLNLLPTTKAGRAAGVHTGLASDPWSRTRCHRHSASVSLFFDLGARRVVMVCHSALLSKALAVPTKRHWAVQKSS

>868|CORE_REP|Org133_Gene119#

MRTFESVADLAAAAGEKVGQSDWVTITQEEVNLFADATGDHQWIHVDPERAAAGPFGTTIAHGFMTLALLPRLQHQMYTVKGVKLAINYGLNKVRFPAPVPVGSRVRATSSLVGVEDLGNGTVQATVSTTVEVEGSAKPACVAESIVRYVA

>869|CORE_REP|Org114_Gene142#

MAALPAPEKLLRSDFPVLWPVGTRWADNDMFGHLNNAVYYQLFDTAINAWINTSTGVDPLAMPVLGIVAESGCRYFSELRFPESLMVGLAVTRLGRSSVTYRLGVFKEPDDAGVITALGHWVHVYVDRTSRRPVPIPEAIRSLLSTACVSG

>870|CORE_REP|Org129_Gene2389#

MSAKARLGQLGVTLPQVAAPLAAYVPAVRTGNLVYTAGQLPLEAGKLVRTGKLGADVNPEEGKTLARICALNALAAVDSLVDLDAVTRVVKVVGFVASAPGFHGQPSVINGASDLLAEVFGDSGAHARSAVGVSELPLDAPVEVELIVEVG

>871|CORE_REP|Org89_Gene198#

MARSQEPSRGLLDPVAKMLRLPFGTPDFIEKIVTGSVNQVGRRTLYVLITTWDAAGGGPFAASAIATTGLAKTAEIVQSMFIGPVFNPLLKMLGADKIAIRASLCAAQLVGLGIMRYGVRSEPLHSMSVEMLVDAIGPTMQRYLVGDIGRG

>872|CORE_REP|Org57_Gene2255#

MTETTPAPQTPAAPAGPAQSFVLERPIQTVGRRKEAVVRVRLVPGTGKFDLNGRSLEDYFPNKVHQQLIKAPLVTVDRVESFDIFAHLGGGGPSGQAGALRLGIARALILVSPEDRPALKKAGFLTRDPRATERKKYGLKKARKAPQYSKR

>873|CORE_REP|Org20_Gene640#

MTVLLDANVLIALVVAEHVHHDAAADWLMASDTGFATCPMTQGSLVRFLVRSGQSAAAARDVVSAVQCTSRHEFWPDALSFAGVEVAGVVGHRQVTDATCPAARPGAGDARQRLSTPARRRRGTHSNDHLMCIVSRRRGEPPQNQRLGHDA

>874|CORE_REP|Org117_Gene3964#

MIVLDASAAVELMLTTPAGAAVARRLRGETVHAPAHFDVEVIGAIRQAVVRQLISDHEGLVVVVNFLSLPVRRWPLKPFTQRAYQLRSTHTVADGAYVALAEGLGVPLITCDGRLAQSHGLNRPGMSGDSSSWKGWGHVRWFIEEVPAGAA

>875|CORE_REP|Org118_Gene2056#

MNSIQIADETYVAADAARVSAAVADRCSWRRWWPDLRLQVTEDRADKGIRWTVTGALTGTMEIWLEPSMDGVLLHYFLHAEPTGVAAWQLARMNLARMTAPPTGRGQKNGLRGQDSARTVTPHRGFSGNLTGSRLARGSTVSPREIGSSRR

>876|CORE_REP|Org111_Gene1999#

MDIAGRSLVYFSSVSENTHRFVQKLGIPATRIPLHGRIEVDEPYVLILPTYGGGRANPGLDAGGYVPKQVIAFLNNDHNRAQLRGVIAAGNTNFGAEFCYAGDVVSRKCSVPYLYRFELMGTEDDVAAVRTGLAEFWKEQTCHQPSLQSL

>877|CORE_REP|Org107_Gene2164#

MNEALDDIDRILVRELAADGRATLSELATRAGLSVSAVQSRVRRLESRGVVQGYSARINPEAVGHLLSAFVAITPLDPSQPDDAPARLEHIEEVESCYSVAGEESYVLLVRVASARALEDLLQRIRTTANVRTRSTIILNTFYSDRQHIP

>878|CORE_REP|Org20_Gene1088#

MVLALAWLSAVAGCSRGGSSKAGRSSSVAGTLPAGVVGVSPAGVTTRVDAPAESTEEEYYQAHAARLWMDAQPGSGESLIEPYLAVVQASPSGVAGSWHIRWAALTPARQAPVVAARAAATRNADNARQLRSGVQETSGRIPIRAPRRAE

>879|CORE_REP|Org20_Gene1947#

MSIDFPLGDDLAGYIAEAIAADPSFKGTLEDAEEARRLVDALIALRKHCQLSQVEVAKRMGVRQPTVSGFEKEPSDPKLSTLQRYARALDARLRLVLEVPTLREVPTWHRLSSYRGSARDHQVRVGADKEILMQTNWARHISVRQVEVA

>880|CORE_REP|Org19_Gene3358#

MAPPNQPEDTQPDAGTESRSALDEPDERTPPRVLTLRIAPWDVVCTVAILAVFLVMVTMTSWPSRLFAFTDNVCPPDACPLVPFGVNYYIYPVMWGGIGAAIATAVIGPFVSMLKGWYMSFWPIISIAVITVTSIAGYAIAGFSERYWH

>881|CORE_REP|Org20_Gene1579#

MPLSGEYAPSPLDWSREQADTYMKSGGTEGTQLQGKPVILLTTVGAKTGKLRKTPLMRVEHDGQYAIVASLGGAPKNPVWYHNVVKNPRVELQDGTVTGDYDAREVFGDEKAIWWQRAVAVWPDYASYQTKTDRQIPVFVLTPVRAGG

>882|CORE_REP|Org126_Gene343#

MKALVAVSAVAVVALLGVSSAQADPEADPGAGEANYGGPPSSPRLVDHTEWAQWGSLPSLRVYPSQVGRTASRRLGMAAADAAWAEVLALSPEADTAGMRAQFICHWQYAEIRQPGKPSWNLEPWRPVVDDSEMLASGCNPGSPEESF

>883|CORE_REP|Org106_Gene334#

MSRLSSILRAGAAFLVLGIAAATFPQSAAADSTEDFPIPRRMIATTCDAEQYLAAVRDTSPVYYQRYMIDFNNHANLQQATINKAHWFFSLSPAERRDYSEHFYNGDPLTFAWVNHMKIFFNNKGVVAKGTEVCNGYPAGDMSVWNWA

>884|CORE_REP|Org4_Gene1523#

MDRLDDTDERILAELAEHARATFAEIGHKVSLSAPAVKRRVDRMLESGVIKGFTTVVDRNALGWNTEAYVQIFCHGRIAPDQLRAAWVNIPEVVSAATVTGTSDAILHVLAHDMRHLEAALERIRSSADVERSESTVVLSNLIDRMPP

>885|CORE_REP|Org74_Gene925#

MSAPMIGMVVLVVVLGLAVLALSYRLWKLRQGGTAGIMRDIPAVGGHGWRHGVIRYRGGEAAFYRLSSLRLWPDRRLSRRGVEIISRRAPRGDEFDIMTDEIVVVELCDSTQDRRVGYEIALDRGALTAFLSWLESRPSPRARRRSM

>886|CORE_REP|Org7_Gene2435#

MTTLNEAAALAAAERGLAVVSTVRADGTVQASLVNVGLLPHPVSGEPSLGFTTYGKVKLGNLRARPQLAVTFRNGWQWATVEGRAQLVGPDDPRPWLVDGERLRLLLREVFTAAGGTHDDWDEYDRVMAQEQRAVVLITPTRIYSNG

>887|CORE_REP|Org80_Gene2991#

MGQVSAASTILINAEPTATLDALADYETVRPKILSPHYSEYQVLEGGKGRGTVAKWRLQATQSRVRDVQVNVDVAGHTVIEKDMNSSMVTNWTVAPAGPGSSVTVKTTWTGAGGVKGFFEKTFAPLGLKKIQAEVLSNLKTELEGDA

>888|CORE_REP|Org23_Gene599#

MAIKESRDIVIEASPEEILDVIADFEAMTEWSPAHQSVEILETGDDGRPSKVKMKVKTAGITDEQVVAYSWTDRSVRWTLVSSTQQRSQDGKYELTPKGDNTLVQFEITVDPQVPLPGFVLKRAIKGTIDTATEALRSQVLKVKKGQ

>889|CORE_REP|Org20_Gene2312#

MSGGAAARWPPATRWWAPRADRVELPREVHQALRQVVAALHAGKAVTIAPQSMTLTTQQAADLLGVSRPTVVRLIKSGELAAERIGNRHRLVLDDVLAYREARRQRQSTPAGARGHRRRQDPEVICEQLREARRVVAARRRTERRRA

>890|CORE_REP|Org114_Gene3112#

MKISEVAALTNTSTKTLRFYENSGLLPPPARTASGYRNYGPEIVDRLRFIHRGQAAGLALQEVRQILAIHDRGEAPCAHVRQLLSTRIDEVRAQIAELIALEGHLQTLLDHASYGPPTEHDHSTVCWILESDLDEPTAIEVSDIHA

>891|CORE_REP|Org2_Gene1390#

MRLSLTALSAGVGAVAMSLTVGAGVASADPVDAVINTTCNYGQVVAALNATDPGAAAQFNASPVAQSYLRNFLAAPPPQRAAMAAQLQAVPGRHSTSALSSRLPAPATTIKPMRAPSRDPASSPGLGQIAPLLNGPHPATRHRRRG

>892|CORE_REP|Org103_Gene2143#

MADSDLPTKGRQRGVRAVELNVAARLENLALLRTLVGAIGTFEDLDFDAVADLRLAVDEVCTRLIRSALPDATLRLVVDPRKDEVVVEASAACDTHDVVAPGSFSWHVLTALADDVQTFHDGRQPDVAGSVFGITLTARRAASSR

>893|CORE_REP|Org119_Gene3411#

MSETFDVDVLVHATHRASPFHDKAKTLVERFLAGPGLVYLLWPVALGYLRVVTHPTLLGAPLAPEVAVENIEQFTSRPHVRQVGEANGFWPVYRRVADPVKPRGNLVPDAHLVALMRHHGIATIWSHDRDFRKFEGIRIRDPFSG

>894|CORE_REP|Org87_Gene2501#

MTKRTITPMTSMGDLLGPEPILLPGDSDAEAELLANESPSIVAAAHPSASVAWAVLAEGALADDKTVTAYAYARTGYHRGLDQLRRHGWKGFGPVPYSHQPNRGFLRCVAALARAAAAIGETDEYGRCLDLLDDCDPAARPALGL

>895|CORE_REP|Org10_Gene3196#

MLSIDTNILLYAQNRDCPEHDAAAAFLVECAGRADVAVCELVLMELYQLLRNPTVVTRPLEGPEAAEVCQTFRRNRRWALLENAPVMNEVWVLAATPRIARRRLFDARLALTLRHHGVDEFATRNINGFTDFGFSRVWDPITSDG

>896|CORE_REP|Org40_Gene1264#

MKSASDPFDLKRFVYAQAPVYRSVVEELRAGRKRGHWMWFVFPQLRGLGSSPLAVRYGISSLEEAQAYLQHDLLGPRLHECTGLVNQVQGRSIEEIFGPPDDLKLCSSMTLFARATDANQDFVALLAKYYGGGEDRRTVALLAVT

>897|CORE_REP|Org59_Gene68#

MDECVVDAAAVVDALAGKGASAIVLRGLLKESISNAPHLLDAEVGHALRRAVLSDEISEEQARAALDALPYLIDNRYPHSPRLIEYTWQLRHNVTFYDALYVALATALDVPLLTGDSRLXXAPGPSVRNQTRSVTSLCGTPMAPS

>898|CORE_REP|Org58_Gene4045#

MVLLRGLPAVNTTATTVSRGRRPPRTLYRGDPGMWSWVCHRISGATIFFFLFVHVLDAAMLRVSPQTYNAVLATYKTPIVGLMEYGLVAAVLFHALNGIRVILIDFWSEGPRYQRLMLWIIGSVFLLLMVPAGVVVGIHMWEHFR

>899|CORE_REP|Org66_Gene1435#

MADKTTQTIYIDADPGEVMKAIADIEAYPQWISEYKEVEILEADDEGYPKRARMLMDAAIFKDTLIMSYEWPEDRQSLSWTLESSSLLKSLEGTYRLAPKGSGTEVTYELAVDLAVPMIGMLKRKAERRLIDGALKDLKKRVEG

>900|CORE_REP|Org48_Gene564#

MMDELRRTGLDKMNEVYAWDMPDMPGEFFALTVDHLFGRIWTRPGLSMRDRRMAVIAVLTAQGQSDLLEVQVNAVLHNDELTIDELRELAVFITHYVGFPLGSRLNSAIERVAAKRKQAAENGSLPDTKANVAEVLAKESGKSS

>901|CORE_REP|Org115_Gene2187#

MSAPVRQRSHDRPASLDNPRSPRRRAGMPNFEKFAWLFMRFSGVVLVFLAIGHLFIMLMWDNGVYRLDFNFVAQRWASPFWQTWDLLLLWLAQLHGGNGLRTIIDDYSRKDTTRFWLNSLLVLSMLFTLMLGTYVIVTFDPNIS

>902|CORE_REP|Org16_Gene2577#

MPCVFCAIIAGEAPAIRIYEDGGYLAILDIRPFTRGHTLVLPKRHTVDLTDTPPEALADMVAIGQRIARAARATKLADATHIAINDGRAAFQTVFHVHLHVLPPRNGDKLSVAKGMMLRRDPDREATGRILREALAQQDAAAQD

>903|CORE_REP|Org1_Gene2819#

MMRTVAIGPGAGPSSTRPSSQPSDLHSGLRAVTECTGSAVVVHVGGDIDASNEVAWQRLVSKSAAIAIAPGPFVIDIRDLDFMGSCAYAVWAQESVRCRRRGVNMRLVSNQPIVARTIAACGLRRLIPLYATVETALAPPPSAH

>904|CORE_REP|Org77_Gene774#

MTHTPIPRPDARYGRPRLSRRARRRVAIALGVLVAAAGIVIAVIGYQRISTSAVTGSLVGYRLVDDETASVTISVTRSDPSRPVACIVRVRATNGSETGRRELLVPPSEATTVQVTTTVKSSQPPVMADVYGCGTEVPSYLRLP

>905|CORE_REP|Org2_Gene388#

MAKMAGSHAAVAAAHGNFQTMTTSEIATVLAWHDALNAADIETLVALSTDDIDIGDAHGAVQGHDALRGWASSLTTTAELGRMYVHHGVVVVEQKITSGEDPGIARTGAAAFRVVQDHVASVFRHEDLASALAATELTEDDLVD

>906|CORE_REP|Org1_Gene2297#

MAAGTMPVGGRQHVFEKLASILGLVAAPLMLLGLSACGRSAGKTSEPTCPTEPIDAADSSTTPDPSCVVRATEINGNGSRIQTWTGSYDAAATQSGGVCGGTCNFHATVRFTVDEGQISGSVDQVYQAAMVAIATRPTSPSLAP

>907|CORE_REP|Org4_Gene864#

MIIPDINLLLYAVITGFPQHRRAHAWWQDTVNGHTRIGLTYPALFGFLRIATSARVLAAPLPTADAIAYVREWLSQPNVDLLTAGPRHLDIALGLLDKLGTASHLTTDVQLAAYGIEYDAEIHSSDTDFARFADLKWTDPLRE

>908|CORE_REP|Org31_Gene637#

MLDSDARLASDLSLAVMRLSRQLRFRNPSSPVSLSQLSALTTLANEGAMTPGALAIRERVRPPSMTRVIASLADMGFVDRAPHPIDGRQVLVSVSESGAELVKAARRARQEWLAERLATLNRSERDILRSAADLMLALVDESP

>909|CORE_REP|Org82_Gene1205#

MITNLRRRTAMAAAGLGAALGLGILLVPTVDAHLANGSMSEVMMSEIAGLPIPPIIHYGAIAYAPSGASGKAWHQRTPARAEQVALEKCGDKTCKVVSRFTRCGAVAYNGSKYQGGTGLTRRAAEDDAVNRLEGGRIVNWACN

>910|CORE_REP|Org2_Gene4045#

MKTSSGRCVRKPYGPSTPLVEPGRIGGNQTRLAAVLLDVSTPNTLNADFDLMRSVAGITDARNEEIRAMLQAFIGRMSGVPPSVWGGLAAARFQDVVDRWNAESTRLYHVLHAIADTIRHNEAALREAGQIHARHIAAAGGDL

>911|CORE_REP|Org36_Gene4051#

MRGCQKVDLKRLAAALPDYPFAYLITVDDGHRVHTVAVEPVLRELPDGPDGPRAVVDVGLIGGRTRQNLAHRSEVTLLWPPSDPSGYSLIVDGRAQASDAGPDDDTARCGVVPIRALLHRDAAPDSPTAAKGCLHDCVVFSVP

>912|CORE_REP|Org69_Gene1554#

MPSLPDRLASILRDVLPAEEEPDGALTVRHDGTFASLRVVSIAEDLELVSLTQILAWDLPLTKRLTEQVAKQARDINFGSVSLREKVSEKAARRSSGRPASNTADVMLRYNFPGTGLTDDALRTLILLVLETGATIRSALVG

>913|CORE_REP|Org98_Gene220#

MFLIDVNVLLAAHRGDHPNHRTVRPWFDRLLAADDPFTVPNLVWASFLRLTTNRRIFEIPSPRADAFAFVEAVNAQPHHLPTSPGPRHLVLLRKLCDEADASGDLIPDAVLGAIAVEHHCAVVSLDRDFARFASVRHIRPPI

>914|CORE_REP|Org97_Gene1597#

MRIADVLRNKGAAVVTINPDATVGELLAGLAEQNIGAMVVVGAEGVVGIVSERDVVRQLHTYGASVLSRPVAKIMSTTVATCTKSDTVDKISVLMTENRVRHVPVLDGKKLIGIVSIGDVVKSRMGELEAEQQQLQSYITQG

>915|CORE_REP|Org62_Gene2826#

MSASRTMVSSGSEFESAVGYSRAVRIGPLVVVAGTTGSGDDIAAQTRDALRRIEIALGQAGATLADVVRTRIYVTDISRWREVGEVHAQAFGKIRPVTSMVEVTALIAPGLLVEIEADAYVGSAVADRNSGAGPKDPSPAGG

>916|CORE_REP|Org1_Gene4305#

MIRSESGAAPPRQHLHLSAQVMRFVVTGGLAGIVDFGLYVVLYKVAGLQVDLSKAISFIVGTITAYLINRRWTFQAEPSTARFVAVMLLYGITFAVQVGLNHLCLALLHYRAWAIPVAFVIAQGTATVINFIVQRAVIFRIR

>917|CORE_REP|Org119_Gene2826#

MVKDLDRRLAGCLPAVLSLFRLVYGLLFAGYGSMILFGWPVTSAQPAIEFGSWPGWYAGVIELVAGLLIATGLFTRAVAFVASGEMAVAYFWMHQPYALWPIGGPPDGNGGTPAILFCFGFFLLVLTGGGIYSIDARRTVTA

>918|CORE_REP|Org132_Gene987#

MQMSASNAFVEGFADFWKAPSPDRLTDHLHPDVVLVRPLSPPRHGLGAAQREFTRILGLLPDLHGEVDRWSQAGDVVFIEFRLIARLGSEVVEWPVVDRFLLRGDKAVERVSYFDSLPLLIKVVKHPSAWRGWLTTMRSRA

>919|CORE_REP|Org89_Gene1635#

MALLDVNALVALAWDSHIHHARIREWFTANATLGWATCPLTEAGFVRVSTNPKVLPSAIGIADARRVLVALRAVGGHRFLADDVSLVDDDVPLIVGYRQVTDAHLLTLARRRGVRLVTFDAGVFTLAQQRPKTPVELLTIL

>920|CORE_REP|Org14_Gene2914#

MQQTAWAPRTSGIAGCGAGGVVMAIASVTLVTDTPGRVLTGVAALGLILFASATWRARPRLAITPDGLAIRGWFRTQLLRHSNIKIIRIDEFRRYGRLVRLLEIETVSGGLLILSRWDLGTDPVEVLDALTAAGYAGRGQR

>921|CORE_REP|Org110_Gene1737#

MSAGPAIEVAVAFVWLGMVVAISFLEAPLKFRAAGVTLQIGLGIGRLVFRALNTVEVGFALVILAIVVVGSTPARIAAAFSVALAALAVQLIAVRPRLTRRSNQVLAGLQAPRSRGHHIYVGLEIVKVVALLVAGILLLNG

>922|CORE_REP|Org59_Gene2669#

MIFVDTNVFMYAVGRDHPLRMPAREFLEHSLEHQDRLVTSAEAMQELLNAYVPVGRNSTLDSALTLVRALTEIWPVEAADVEXMREPCTTATPVWARAICYTWHAASVAVSRGSRRSTTHWPAHSDHDASVWARASAVVRP

>923|CORE_REP|Org59_Gene380#

MSYAGDITPLQAWEMLSDNPRAVLVDVRCEAEWRFVGVPDLSSLGREVVYVEWATSDGTHNDNFLAELRDRIPADADQHEXXXXFILCRSGNRSIGAAEVATEAGITPAYNVLDGFEGHLDAEGHRGATGWRAVGLPWRQG

>924|CORE_REP|Org63_Gene1457#

MTAKSPPDYPGKTLGLPDTGPGSLAPMGRRLAALLIDWLIAYGLALLGVEFGVWSTPMLSTVVLVIWLLLGVAAVRLFGFTPGQLMLGLVVVAVGGRRPVGIGRLVVRGLLIGLVVPPLFTDSDGRGLHDRLTATAVVRR

>925|CORE_REP|Org18_Gene3499#

MQPSPDSPAPLNVTVPFDSELGLQFTELGPDGARAQLDVRPKLLQLTGVVHGGVYCAMIESIASMAAFAWLNSHGEGGSVVGVNNNTDFVRSISSGMVYGTAEPLHRGRRQQLWLVTITDDTDRVVARGQVWLQNLEARP

>926|CORE_REP|Org4_Gene2547#

MTTMIVASVATGALATIARWLLTRRSVILREVGPETTPAAPARTAELGLSGAGPTVVHFRAPGCAPCDRVRRGVGDVCADLGDVAHIEVDLDSNPQAARRFSVLSLPTTLIFDVDGRQRYRTSGVPKAADLRSALKPLLA

>927|CORE_REP|Org59_Gene3076#

MGQIPPQPVRRVLPLMVVPGNGQKWRNRTETEEAMGDTYRDPVDHLRTTRPLAGESLIDVVHWPGYLLIVAGVVGGXGALAAFGTGHHAEGMTFGVVAIVVTVVGLAWLAFEHRRIRKIADRWYTEHPEVRRQRLAGXTS

>928|CORE_REP|Org59_Gene1196#

MLSPLSPRIIAAFTTAVGAAAIGXAVATAGTAGANTKDEAFIAQMESIGVTFSSPQVATQQAQLVCKKLASGETGTEIAEEVLSQTNLTTKQAAYFVVDATKAYXPQXXXPAXXSRCPPDAGIPSTGIPAFALPPDNESV

>929|CORE_REP|Org119_Gene699#

MTQTTQSPALIASQSSWRCVQAHDREGWLALMADDVVIEDPIGKSVTNPDGSGIKGKEAVGAFFDTHIAANRLTVTCEETFPSSSPDEIAHILVLHSEFDGGFTSEVRGVFTYRVNKAGLITNMRGYWNLDMMTFGNQE

>930|CORE_REP|Org3_Gene2787#

MIVDTSAVVALVQGERPHATLVAAALAGAHSPVMSAPTVAECLIVLTARHGPVARTIFERLRSEIGLSVSSFTAEHAAATQRAFLRYGKGRHRAALNFGDCMTYATAQLGHQPLLAVGNDFPQTDHEFRGVVGYWPGVA

>931|CORE_REP|Org67_Gene2938#

MGLADDAPLGYLLYRVGAVLRPEVSAALSPLGLTLPEFVCLRMLSQSPGLSSAELARHASVTPQAMNTVLRKLEDAGAVARPASVSSGRSLPATLTARGRALAKRAEAVVRAADARVLARLTAPQQREFKRMLEKLGSD

>932|CORE_REP|Org135_Gene2330#

MLRTMLKSKIHRATVTCADLHYVGSVTIDADLMDAADLLEGEQVTIVDIDNGARLVTYAITGERGSGVIGINGAAAHLVHPGDLVILIAYATMDDARARTYQPRIVFVDAYNKPIDMGHDPAFVPENAGELLDPRLGVG

>933|CORE_REP|Org36_Gene1687#

MTALLDVNVLIALGWPNHVHHAAAQRWFTQFSSNGWATTPITEAGYVRISSNRSVMQVSTTPAIAIAQLAAMTSLAGHTFWPDDVPLIVGSAGDRDAVSNHRRVTDCHLIALAARYGGRLVTFDAALADSASAGLVEVL

>934|CORE_REP|Org142_Gene1445#

MHIEARLFEFVAAFFVVTAVLYGVLTSMFATGGVEWAGTTALALTGGMALIVATFFRFVARRLDSRPEDYEGAEISDGAGELGFFSPHSWWPIMVALSGSVAAVGIALWLPWLIAAGVAFILASAAGLVFEYYVGPEKH

>935|CORE_REP|Org76_Gene697#

MRVNRPQCARVPYSAESLVRVEASWYGRTLRAIPEVLSQVGYQQADHGESLLTSHHCCLGAAEGARPGWVGSSAGALSGLLDSWAEASTAHAARIGDHSYGMHLAAVGFAEMEEHNAAALAAVYPTGGGSARCDGVDVS

>936|CORE_REP|Org120_Gene1785#

MTTRYLLDKSAAYRAHLPAVRHRLEPLMERGLLARCGITDLEFGVSARSREDHRTLGTYRRDALEYVNTPDTVWVRAWEIQEALTDKGFHRSVKIPDLIIAAVAEHHGIPVMHYDQDFERIAAITRQPVEWVVAPGTA

>937|CORE_REP|Org120_Gene1619#

MSVGFVTPVGVRWSDIDMYQHVNHATMVTILEEARVPFLKDAFGADITSTGLLIADVRVTYKGQLRLSDSPLQVTIWTKRLRAVDFTLGYEVRSVNAEPDSRPAVIAESQLAAFHIEEQRLVRLSPHHREYLQRWFRG

>938|CORE_REP|Org101_Gene527#

MLIPRKVKHRKQHHPRQRGIASGGTTVNFGDYGIQALEHAYVTNRQIESARIAINRHIKRGGKVWINIFPDRPLTKKPAETRMGSGKGSPEWWVANVKPGRVLFELSYPNEGVARAALTRAIHKLPIKARIITREEQF

>939|CORE_REP|Org1_Gene455#

MPEHELGPVRALGWLREDRKPLLNAKLLVLGHLALNVYDPDNGYGEEVLDFEPRTVWWGSANWTVRAGSHLEVGFACDDPTLVEEATAFVADVIAFSEPIDTTCAGPEPNLVQVEFDDAAMAEAMEEMAEPDDDGEDW

>940|CORE_REP|Org59_Gene633#

MIYFLVDSSAVWRLATTXGTHRGLELRAAQRAVGSCEPQRAEFCRSARNADEFDQMSRMFGDVYPDVPVPKSVWRWIDSAQHRLARAGAVGALSVVDLLICDTAAARGLVVLHDDADYELAERHLPDIRVRRVVSADD

>941|CORE_REP|Org2_Gene982#

MGHVESGHVVWMRSAIVAVALGVTVAAVAAACWLPQLHRHVAHPNHPLTTSVGSEFVINTDHGHLVDNSMPPCPERLATAVLPRSATPVLLPDVVAAAPGMTAALTDPVAPAARGPPAAQGSVRTGQDLLTRFCLARR

>942|CORE_REP|Org150_Gene430#

MKPPLAVDTSVAIPLLVRTHTAHAAVVAWWAHREAALCGHALAETYSVLTRLPRDLRLAPMDAARLLTERFAAPLLLSSRTTEHLPRVLAQFEITGGAVYDALVALAAAEHRAELATRDARAKDTYEKIGVHVVVAA

>943|CORE_REP|Org26_Gene1911#

MTQLWVERTGTRRYIGRSTRGAQVLVGSEDVDGVFTPGELLKIALAACSGMASDQPLARRLGDDYQAVVKVSGAADRDQERYPLIEETMELDLSGLTEDEKERLLVVINRAVELACTVGRTLKSGTTVNLEVVDVGA

>944|CORE_REP|Org45_Gene2794#

MAMVNTTTRLSDDALAFLSERHLAMLTTLRADNSPHVVAVGFTFDPKTHIARVITTGGSQKAVNADRSGLAVLSQVDGARWLSLEGRAAVNSDIDAVRDAELRYAQRYRTPRPNPRRVVIEVQIERVLGSADLLDRA

>945|CORE_REP|Org57_Gene1415#

MRNMKSTSHESESGKLLSISSCRPREMVLQRYSLGMTVTADRHLADKREEFAVEDISTGIFASGYGQVGDGRSFSFHIEHRSLVVEIYRPRVAGPVPQAEDVVAMAVRGLVDIDLTDERSLAAAVRDSVASAAPVSR

>946|CORE_REP|Org47_Gene1692#

MVFCVDTSAWHHAARPEVARRWLAALSADQIGICDHVRLEILYSANSATDYDALADELDGLARIPVGAETFTRACQVQRELAHVAGLHHRSVKIADLVIAAAAELSGTIVWHYDENYDRVAAITGQPTEWIVPRGTL

>947|CORE_REP|Org99_Gene70#

MDYTLRRRSLLAEVYSGRTGVSEVCDANPYLLRAAKFHGKPSRVICPICRKEQLTLVSWVFGEHLGAVSGSARTAEELILLATRFSEFAVHVVEVCRTCSWNHLVKSYVLGAARPARPPRGSGGTRTARNGARTASE

>948|CORE_REP|Org20_Gene1613#

MSAKDHPNNAPGVPMVFPLWLERLQVKYINRALKPIARYLPGTATIEHRGRKSGKPYQTIVTAYRKDGVLAIALAHGKTDWVKNVLAAGEADVHFARGVVHVINPRIVPAGSDGQGLPRMARLQLRRIGVFVGDIA

>949|CORE_REP|Org127_Gene127#

MTPFDDPQAELAWMFLQSLCEGGDLDEGFALLSNDFTYWSIVTRTELDKKTFRRAVERRKQVFEVNIELIRCVNEGETVVVEGHCDGVSADRTRYDSPFVCIFETRDGMIISLREYSDTQSLAEVYPVACATPGRC

>950|CORE_REP|Org148_Gene1056#

MGLLSRLRKREPISIYDKIGGHEAIEVVVEDFYVRVLADDQLSAFFSGTNMSRLKGKQVEFFAAALGGPEPYTGAPMKQVHQGRGITMHHFSLVAGHLADALTAAGVPSETITEILGVIAPLAVDVTSGESTTAPV

>952|CORE_REP|Org64_Gene2212#

MIYMDTSALTKLLISEPETTELRTWLTAQSGQGEDAATSTLGRVELMRVVARYGQPGQTERARYLLDGLDILPLTEPVIGLAETIGPATLRSLDAIHLAAAAQIKRELTAFVTYDHRLLSGCREVGFVTASPGAVR

>953|CORE_REP|Org150_Gene1315#

MAEPRRGDLWLVSLGAARAGEPGKHRPAVVVSVDELLTGIDDELVVVVPVSSSRSRTPLRPPVAPSEGVAADSVAVCRGVRAVARARLVERLGALKPATMRAIENALTLILGLPTGPERGEAATHSPVRWTGGRDP

>954|CORE_REP|Org20_Gene2415#

MRDHLGLVPGEVEVVADGAGLGVATLAGDSLGERHGLPVIPAGSAARCRPALAPCSWTRASRSHQVQRGHDHHEDTFQALRGRTLGLAGHAAFELHAGDRVELLAHTFPATRFLGAGAAMSLLPELAPAEIAGGAV

>955|CORE_REP|Org59_Gene2500#

MGAQGYLRRLTRRLTEDLEQRDVEELSDEVLNAGAQRAIDCQRGQEVTVVGTLRSVETNGKGCSGGVRAELFDGSDTVTLVWLGQRRIPGIDTGRTLRVRGRLGXLGKWDEGYLQPALRNSAVTIRQSELPVSRER

>956|CORE_REP|Org137_Gene2747#

MRWIVDGMNVIGSRPDGWWRDRHRAMVMLVERLEGWAITKARGDDVTVVFERPPSTAIPSSVVEVAHAPKAAANSADDEIVRLVRSGAQPQEIRVVTSDKALTDRVRDLGAAVYPAERFRDLIDPRGSNAARRTQ

>957|CORE_REP|Org90_Gene1555#

MVTSPSTPTAAHEDVGADEVGGHQHPADRFAECPTFPAPPPREILDAAGELLRALAAPVRIAIVLQLRESQRCVHELVDALHVPQPLVSQHLKILKAAGVVTGERSGREVLYRLADHHLAHIVLDAVAHAGEDAI

>958|CORE_REP|Org86_Gene1258#

MIRELVTTAAITGAAIGGAPVAGADPQRYDGDVPGMNYDASLGAPCSSWERFIFGRGPSGQAEACHFPPPNQFPPAETGYWVISYPLYGVQQVGAPCPKPQAAAQSPDGLPMLCLGARGWQPGWFTGAGFFPPEP

>959|CORE_REP|Org2_Gene2399#

MGDGMPGSRVTPPAHPCGRISVSLNLRFVSTYRSPDRAWQALADGTRRAIVERLAHGPLAVGELARDLPVSRPAVSQHLKVLKTARLVCDRPAGTRRVYQLDPTGLAALRTDLDRFWTRALTGYAQLIDSEGDDT

>960|CORE_REP|Org10_Gene1048#

MILIDTSAWVEYFRATGSIAAVEVRRLLSEEAARIAMCEPIAMEILSGALDDNTHTTLERLVNGLPSLNVDDAIDFRAAAGIYRAARRAGETVRSINDCLIAALAIRHGARIVHRDADFDVIARITNLQAASFR

>961|CORE_REP|Org66_Gene1263#

MVPFLMRAAVTGFALWVVTLFVPGMRFAGGDTTLQRVAIIFVVAVIFGLVNAFIKPIVQILSIPLYILTLGLFHVVVNASMLWLTAWITEHTTHWGLQIDHFWWTAIWAAILLSIVSWILSLLARDFRRVTRAH

>962|CORE_REP|Org83_Gene3312#

MSDIPSDLHYTAEHEWIRRSGDDTVRVGITDYAQSALGDVVFVQLPVIGTAVTAGETFGEVESTKSVSDLYAPISGKVSEVNSDLDGTPQLVNSDPYGAGWLLDIQVDSSDVAALESALTKLLDAEAYRGTLTE

>963|CORE_REP|Org61_Gene1684#

MIAPDTSVLVAGFATWHEGHEAAVRALNRGVHLIAHAAVETYSVLTRLPPPHRIAPVAVHAYLADITSSNYLALDARSYRGLTDHLAEHDVTGGATYDALVGFTAKAAGAKLLTRDLRAVETYERLRVEVELVT

>964|CORE_REP|Org119_Gene3335#

MADRIELRGLTVHGRHGVYDHERVAGQRFVIDVTVWIDLAEAANSDDLADTYDSPRAGISGAAEIVAGPPRKLIETVGAEIADHVMDDQRVHAVEVAVHKPQAPIPQTFDDVAVVIRRSRRGGRGWVVPAGGAV

>965|CORE_REP|Org117_Gene960#

MLGDAQQLELGRCAPADIALTVAATVVSRQDCRSGLRRIVLDCGSKILGSDRPAWATGFGRLIDHADARIAALSEHHATVVWPDDAPLPPVGTRLRVIPNHVCLTTNLVDDVAVVRDATLIDRWKVAARGKNH

>966|CORE_REP|Org142_Gene2607#

MILVDSDVLIAHLRGVVAARDWLVSARKDGPLAISVVSTAELIGGMRTAERREVWRLLASFRVQPATEVIARRAGDMMRRYRRSHNRIGLGDYLIAATADVQDLQLATLNVWHFPMFEQLKPPFAVPGHRPRA

>967|CORE_REP|Org13_Gene439#

MIVDTSAIIAILRDEDDAAAYADALANADVRRLSAASYLECGIVLDSQRDPVISRALDELIEEAEFVVEPVTERQARLARAAYADFGRGSGHPAGLNFGDCLSYALAIDRREPLLWKGNDFGHTGVQRALDRR

>968|CORE_REP|Org149_Gene2009#

MPDSSTALRILVYSDNVQTRERVMRALGKRLHPDLPDLTYVEVATGPMVIRQMDRGGIDLAILDGEATPTGGMGIAKQLKDELASCPPILVLTGRPDDTWLASWSRAEAAVPHPVDPIVLGRTVLSLLRAPAH

>969|CORE_REP|Org120_Gene2388#

MQPGGDMSALLAQAQQMQQKLLEAQQQLANSEVHGQAGGGLVKVVVKGSGEVIGVTIDPKVVDPDDIETLQDLIVGAMRDASQQVTKMAQERLGALAGAMRPPAPPAAPPGAPGMPGMPGMPGAPGAPPVPGI

>970|CORE_REP|Org71_Gene1660#

MTTWILDKSAHVRLVAGATPPAGIDLTDLAICDIGELEWLYSARSATDYDSQQTSLRAYQILRAPSDIFDRVRHLQRDLAHHRGMWHRTPLPDLFIAETALHHRAGVLHHDRDYKRIAVVRPGFQACELSRGR

>971|CORE_REP|Org28_Gene2011#

MPNHDYRELAAVFAGGALGALARAALSALAIPDPARWPWPTFTVNVVGAFLVGYFTTRLLERLPLSSYRRPLLGTGLCGGLTTFSTMQVETISMIEHGHWGLAAAYSVVSITLGLLAVHLATVLVRRVRIRR

>972|CORE_REP|Org121_Gene3#

MSTTFAARLNRLFDTVYPPGRGPHTSAEVIAALKAEGITMSAPYLSQLRSGNRTNPSGATMAALANFFRIKAAYFTDDEYYEKLDKELQWLCTMRDDGVRRIAQRAHGLPSAAQQKVLDRIDELRRAEGIDA

>973|CORE_REP|Org27_Gene529#

MTMTDPIADFLTRLRNANSAYHDEVSLPHSKLKANIAQILKNEGYISDFRTEDARVGKSLVIQLKYGPSRERSIAGLRRVSKPGLRVYAKSTNLPRVLGGLGVAIISTSSGLLTDRQAARQGVGGEVLAYVW

>974|CORE_REP|Org86_Gene1099#

MEASGRQRRYAAAGSVVLLAGALGYIGLVDPHNSNSLYPPCLFKLLTGWNCPACGGLRMIHDLLHGELAASINDNVFLLVGVPVLASWVLLRRRHGDLALPIPVMIAVAVAVIAWTVLRNLPGFPLVPTISG

>975|CORE_REP|Org60_Gene1670#

MIFVDTSFWAALGNAGDARHGTAKRLWASKPPVVMTSNHVLGETWTLLNRRCGHRAAVAAAAIRLSTVVRVEHVTADLEEQAWEWLVRHDEREYSFVDATSFAVMRKKGIQNAYAFDGDFSAAGFVEVRPE

>976|CORE_REP|Org126_Gene1232#

MILVDSNIPMYLVGASHPHKLDAQRLLESALSGGERLVTDAEVLQEICHRYVAIKRREAIQPAFDAIIGVVDEVLPIERTDVEHARDALLRYQTLSARDALHIAVMAHHDITRLMSFDRGFDSYPGIKRLA

>977|CORE_REP|Org148_Gene1790#

MIVDTSAIVAIVSGESGAQVLKEALERSPNSRMSAPNYVELCAIMQRRDRPEISRLVDRLLDDYGIQVEAVDADQARVAAQAYRDYGRGSGHPARLNLGDTYSYALAQVTGEPLLFRGDDFTHTDIRPACT

>978|CORE_REP|Org12_Gene3799#

MDQMPKSFYDAVGGAKTFDAIVSRFYAQVAEDEVLRRVYPEDDLAGAEERLRMFLEQYWGGPRTYSEQRGHPRLRMRHAPFRISLIERDAWLRCMHTAVASIDSETLDDEHRRELLDYLEMAAHSLVNSPF

>979|CORE_REP|Org120_Gene460#

MVIDTSALVAMLSDEPDAERFEAAVEADHIRLMSTASYLETALVIEARFGEPGGRELDLWLHRAAVDLVAVHADQADAARAAYRTYGKGRHRAGLNYGDCFSYGLAKISGQPLLFKGEDFQHTDIATVALP

>980|CORE_REP|Org92_Gene3553#

MHSEQSASIEHVDVLIVGAGISGTGAAYYLKTMQPAKTFAIVEARYPAIRSDSDLHTFSYEFKPWQHEKATASADAIMVHRGRSLAGGDRTLRHRRTRHHELRMVIIGSGATAVTLVPAMAQTAGAVTMPK

>981|CORE_REP|Org130_Gene2040#

MTVSTPEQHEQRASHDASEGKHNVCQGRLAALADAAVSEKLGALPGWQLLDMRLSRAFQCTNFDQSIDFMNRVASIANDINHHPDIAVLDKRSVRVTAWTRKLGYLTDIDFDLAASVEAMYATEFADRPAR

>982|CORE_REP|Org4_Gene3939#

MYHRTGGRVGCKLRLGAGFRKPVPTLLLEHRSRKSGKNFVAPLLYITDRNNVIVVASALGQAENPQWYRNLPPNPDTHIQIGSDRRPVRAVVASSDERARLWPRPVDAYADFDSCQSWTERGIPVIILRPR

>983|CORE_REP|Org16_Gene851#

MFANIGWGEMLVLVMVGLVVLGPERLPGAIRWAASALRQARDYLSGVTSQLREDIGPEFDDLRGHLGELQKLRGMTPRAALTKHLLDGDDSLFTGDFDRPTPKKPDAAGSAGPDATEQIGAGPIPFDSDAT

>984|CORE_REP|Org12_Gene760#

MSRIDRVLEAARRRYRRLAADQVPEAARRGAVLVDIRPQAQRAREGEVPGALVIERNVLEWRCDPTSDARLPQAVDDDVEWVILCSEGYTSSLAAASLLDLGLHRATDVVGGYRALAAGGVLAELGGAVGG

>985|CORE_REP|Org1_Gene691#

MVNVRRALADTSVFIGIEATRFDPDRFAGYEWGVSVVTLGELRLGVLQASGPEAAARRLSTYQLAQRFEPLGIDEAVSEAWALLVSKLRAAKLRVPINDSWIAATAVAHGIAILTQDNDYAAMPDVEVITI

>986|CORE_REP|Org140_Gene1830#

MTTVLLDSHVAYWWSAEPQRLSMAASQAIEHADELAVAAISWFELAWLAEQERIQLAIPVLSWLQQLAEHVRTVGITPSVAATAVALPSSFPGDPADRLIYATAIEHGWRLVTKDRRLRSHRHPRPVTVW

>987|CORE_REP|Org13_Gene603#

MSARDRVDPAKTRQVVLALADWLRDETLPAPDTDVLAAAVRLTARTLAALAPPGASVEVRIPPFAAVQCISGPRHTRGTPPNVVQTDPRTWLLVATGLSGVAQARGSGALQLSGSRAGEIEAWLPLVDLG

>988|CORE_REP|Org63_Gene598#

MYADSGPDPLPDDQVCLVVEVFRMLADATRVQVLWSLADREMSVNELAEQVGKPAPSVSQHLAKLRMARLVRTRRDGTTIFYRLENEHVRQLVIDAVFNAEHAGPGIPRHHRAAGGLQSVAKASATKDVG

>989|CORE_REP|Org117_Gene2185#

MAAIYLDSSAIVKLAVREPESDALRRYLRTRHPRVSSALARAEVMRALLDKGESARKAGRRALAHLDLLRVDKRVLDLAGGLLPFELRTLDAIHLATAQRLGVDLGRLCTYDDRMRDAAKTLGMAVIAPS

>990|CORE_REP|Org130_Gene1667#

MGIVGVGIDLVSIPDFAEQVDQPGTVFAETFTPGERRDASDKSSSAARHLAARWAAKEAVIKAWSGSRFAQRPVLPEDIHRDIEVVTDMWGRPRVRLTGAIAEYLADVTIHVSLTHEGDTAAAVAILEAP

>991|CORE_REP|Org126_Gene2206#

MKARLPDSPLDWLVSKFAREVPGVAHALLVSVDGLPVAASEHLPRERADQLAAVTSGLASLAGGAAQLFDGGQVLQSVVEMQNGYLLLMQVGDGSALAALAATGCDIGQIGYEMAILVERVGGVVQSCRR

>992|CORE_REP|Org108_Gene2761#

MSAAGVRSTRQRAAISTLLETLDDFRSAQELHDELRRRGENIGLTTVYRTLQSMASSGLVDTLHTDTGESVYRRCSEHHHHHLVCRSCGSTIEVGDHEVEAWAAEVATKHGFSDVSHTIEIFGTCSDCRS

>993|CORE_REP|Org47_Gene2428#

MTSLLEVLGAPEVSVCGNAGQPMTLPEPVRDALYNVVLALSQGKGISLVPRHLKLTTQEAADLLNISRPTLVRLLEDGRIPFEKPGRHRRVSLDALLEYQQETRSNRRAALGELSRDALGELQAALAEKK

>994|CORE_REP|Org41_Gene2319#

MTVVGAVLPELKLYGDPTFIVSTALATRDFQDVHHDRDKAVAQGSKDIFVNILTDTGLVQRYVTDWAGPSALIKSIGLRLGVPWYAYDTVTFSGEVTAVNDGLITVKVVGRNTLGDHVTATVELSMRDS

>995|CORE_REP|Org102_Gene3999#

MATWDDVARIVGGLPLTAEQAPHDWRVGRKLLAWERPLRKSDREALTRAGSEPPSGDIVGVRVSDEGVKFALIADEPGVYFTTPHFDGYPAVLVRLAEIEVRDLEELITEAWLMXAPKQLVQAFLANSG

>996|CORE_REP|Org127_Gene1757#

MRMTPDPAMLVHLCGVQEWSHARERGGIYPESDKTGYIHLSTLEQVHLPANRLYRGRADLVLLYIDPAALDSPVRWEPGVPTDPRSMLFPHLYGPLPVRAVIGAAAYPPAGDGSFGPAPEFRSATADPT

>997|CORE_REP|Org54_Gene69#

MPAVTTPSNHWGDERRKLSHQPPVRGQILGRRQARRLSQHFARVGVEAPPKRLQEMLLGAPAADEEWTDVKFALIVTQLNHEKRVAKFHRLQRRATHSLICLGLVLVALNFLICLAYIFFSLTQHAAAL

>998|CORE_REP|Org55_Gene1956#

MKPQDQGLHFPYRYDLRLAPMWLPFRWPGSQGVTVTEDGRFVARYGPFRVEAPLSSVRDAHITGPYRWWTAVGPRLSMVDDGLTFGTNAAAGVCIHFEPRIHRVIGLRDHSALTVTVADPEGLVAALSS

>999|CORE_REP|Org102_Gene1073#

MARVKRAVNAHKKRRSILKASRGYRGQRSRLYRKAKEQQLHSLNYAYRDRRARKGEFRKLWIARINAAARLNDITYNRLIQGLKAAGVEVDRKNLADIAISDPAAFTALVDVARAALPEDVNAPSGEAA

>1000|CORE_REP|Org40_Gene2670#

MNPTQAGSFTTPVSNALKATIQHHDSAVIIHARGEIDAANEHTWQDLVTKAAAATTAPEPLVVNLNGLDFMGCCAVAVLAHEAERCRRRGVDVRLVSRDRAVARIIHACGYGDVLPVHPTTESALSAT

>1001|CORE_REP|Org108_Gene1528#

MPPVFLPQIGRLTPDAVGEAIGIAADDIPMAARWIGSRPCSLIGQPNTMGDEMGYLGPGLAGQRCVDRLVMGASRSTCSRLPVIASVDERLSVLKPVRPRLHSISFIFKGRPGEVYLTVTGYNFRGVP

>1002|CORE_REP|Org49_Gene703#

MIVVDASAALAALLNDGQARQLIAAERLHVPHLVDSEIASGLRRLAQRDRLGAADGRRALQTWRRLAVTRYPVVGLFERIWEIRANLSAYDASYVALAEALNCALVTADLRLSDTGQAQCPITVVPR

>1003|CORE_REP|Org44_Gene1146#

MTAHTHDGTRTWRTGRQATTLLALLAGVFGGAASCAAPIQADMMGNAFLTALTNAGIAYDQPATTVALGRSVCPMVVAPGGTFESITSRMAEINGMSRDMASTFTIVAIGTYCPAVIAPLMPNRLQA

>1004|CORE_REP|Org59_Gene2705#

MSAEDLEKYETEMELSLYREYKDIVGQFSYVVETERRFYLANSVEMVPRNTDGEVYFELRLADAWXWDMYRPARFVKQVRVVTFKDVNIEXVEXPRAAPAGIAAPSAVTRQSAVCGSRSVVDDXLRR

>1005|CORE_REP|Org26_Gene264#

MAKNPKDGESRTFLISVAAELAGMHAQTLRTYDRLGLVSPRRTSGGGRRYSLHDVELLRQVQHLSQDEGVNLAGIKRIIELTSQVEALQSRLQEMAEELAVLRANQRREVAVVPKSTALVVWKPRR

>1006|CORE_REP|Org56_Gene119#

MSNRIVLEPSADHPITIEPTNRRVQVRVNGEVVADTAAALCLQEASYPAVQYIPLADVVQDRLIRTETSTYCPFKGEASYYSVTTDAGDIVDDVMWTYENPYPAVAAIAGHVACYPDKAEISIFPG

>1007|CORE_REP|Org148_Gene1992#

MTASTALTVAIWIGVMLIGGIGSVLRFLVDRSVARRLARTFPYGTLTVNITGAALLGFLAGLALPKDAALLAGTGFVGAYTTFSTWMLETQRLGEDRQMVSALANIVVSVVLGLAAALLGQWIAQI

>1008|CORE_REP|Org26_Gene1592#

MTANREAIDMARVAAGAAAAKLADDVVVIDVSGQLVITDCFVIASGSNERQVNAIVDEVEEKMRQAGYRPARREGAREGRWTLLDYRDIVVHIQHQDDRNFYALDRLWGDCPVVPVDLSANSAGAQ

>1009|CORE_REP|Org99_Gene4078#

MARTLALRASAGLVAGMAMAAITLAPGARAETGEQFPGDGVFLVGTDIAPGTYRTEGPSNPLILVFGRVSELSTCSWSTHSAPEVSNENIVDTNTLNRPGMSGDSSSWKGWGHVRWFIEEVPAGAA

>1010|CORE_REP|Org73_Gene1288#

MKTARLQVTLRCAVDLINSSSDQCFARIEHVASDQADPRPGVWHSSGMNRIRLSTTVDAALLTSARDMRAGITDAALIDEALAALLARHRSAEVDASYAAYDKHPVDEPDEWGDLASWRRAAGDS

>1011|CORE_REP|Org124_Gene1676#

MKLIDTTIAVDHLRGEPAAAVLLAELINNGEEIAASELVRFELLAGVRESELAALEAFFSAVVWTLVTEDIARIGGRLARRYRSSHRGIDDVDYLIAATAIVVDADLLTTNVRHFPMFPDLQPPY

>1012|CORE_REP|Org135_Gene1060#

MTPSTSDARSRRRSAEPFLWLLFSAGGMVTALVAPVLLLLFGLAFPLGWLDAPDHGHLLAMVRNPITKLVVLVLVVLALFHAAHRFRFVLDHGLQLGRFDRVIALWCYGMAVLGSATAGWMLLTM

>1013|CORE_REP|Org2_Gene3496#

MATTSPVVIKVSPMAHFAVGFLTLGLLVPVLTWPVSAPLLVIPVALSASIIRLRTLADERGVTVRTLVGSRAVRWDDIDGLRFHRGSWARATLKDGTELRLPAVTFATLPHLTEASSGRVPNPYR

>1014|CORE_REP|Org96_Gene498#

MKWNTVAASLAAGVITIAVALAAPPPAAHAKNGDTHVTGQGIERTLDCNESTLLVNGTQNIVTALGTCWAVTVMGSSNTVVADTIINDITVYGWDETVFFRNGDPFIWDRGRELGMVNRLQRVG

>1015|CORE_REP|Org118_Gene2642#

MSVIQDDYVKQAEVIRGLPKKKNGFELTTTQLRVLLSLTAQLFDEAQQSANPTLPRQLKEKVQYLRVRFVYQSGREDAVKTFVRNAKLLEALEGIGDSRDGLLRFCRYMEALAAYKKYLDPKDK

>1016|CORE_REP|Org5_Gene1648#

MLPENLEQRVTALESQVRELADRVRASEQDAAAARVLAGAADRDVTEFVGEFRDFRRATIGSFNALREDFTALREEMTERFSHVEERFSRVDDGFTEMRGKLDGAAAGQQRIVELIEQLIADQG

>1017|CORE_REP|Org115_Gene1631#

MTDRSREPADPWKGFSAVMAATLILEAIVVLLAIPVVDAVGGGLRPASLGYLVGLAVLLILLTGLQRRPWAIWVNLGAQPVLVAGFAVYPGVGFIGVLFAALWVLIAYLRAEVRRRRDYRVSQ

>1018|CORE_REP|Org45_Gene1020#

MTTRDLTAAQFNETIQSSDMVLVDYWASWCGPCRAFAPTFAESSEKHPDVVHAKVDTEAERELAAAAQIRSIPTIMAFKNGKLLFNQAGALPPAALESLVQQLKAYEVEAGEATTQNGRAQQA

>1019|CORE_REP|Org134_Gene118#

MTKKPRNPADYVIGDDVEVSDVDLKQEEVYVDGERLTDERVEQMASESLRLAREREANLIPGGKSLSGGSAHSPAVQVVVSKATHAKLKELARSRKMSVSKLLRPVLDEFVQRETGRILPRR

>1020|CORE_REP|Org44_Gene485#

MFLPNTRAYRRYNRSVWAVRGSTRPQWQPPPKFQHAKCMSMRLAHRLQILLDDECHRRITAVARERGVPVATVVREAIDRGLVSPAGRRKSAGRRLLDAADMSVPEPRELKQELEALRARRG

>1021|CORE_REP|Org48_Gene2384#

MSAPDSITVTVADHNGVAVLSIGGEIDLITAAALEEAIGEVVADNPTALVIDLSAVEFLGSVGLKILAATSEKIGQSVKFGVVARGSVTRRPIHLMGLDKTFRLFSTLHDALTGVRGGRIDR

>1022|CORE_REP|Org115_Gene528#

MAQSVSATRRISRLRRHTRLRKKLSGTAERPRLVVHRSARHIHVQLVNDLNGTTVAAASSIEADVRGVPGDKKARSVRVGQLIAERAKAAGIDTVVFDRGGYTYGGRIAALADAARENGLSF

>1023|CORE_REP|Org88_Gene848#

MALVLVYLVVLVLVAIVLFAAASLLFGRGEQLPPLPRATTATTLPAFGVTRADVDAVKFTQVLRGYKTSEVDWVLERLGRELEALRSQLGAIHASSEDAEAESDASNPSRGETVVHYRSDPA

>1024|CORE_REP|Org53_Gene521#

MIQQESRLKVADNTGAKEILCIRVLGGSSRRYAGIGDVIVATVKDAIPGGNVKRGDVVKAVVVRTVKERRRPDGSYIKFDENAAVIIKPDNDPRGTRIFGPVGRELREKRFMKIISLAPEVL

>1025|CORE_REP|Org90_Gene117#

MAGSVSAAAGIGWVGLNVTETNRDQCYRVERTTVDALTHPEYRVHTRGVQRVRVTRNARKHRVSKHRIVAAMRHCGVPVIQEDGSLYYQGRDTSGRLTEVVAVEADDGDLIITHAMPKEWKR

>1026|CORE_REP|Org127_Gene917#

MAELNVEIVAVDRNIWSGTAKFLFTRTTVGEIGILPRHIPLVAQLVDDAMVRVEREGEKDLRIAVDGGFLSVTEEGVSILAESAEFESEIDEAAAKQDSESDDPRIAARGRARLRAVGAID

>1027|CORE_REP|Org20_Gene300#

MTLRVVPEGLAAASAAVEALTARLAAAHASAAPVITAVVPPAADPVSLQTAAGFSAQGVEHAVVTAEGVEELGAPALVWANPAPATWPVMRPPPLRTGSWAAEHGRAHLDGFAAGGTFGVA

>1028|CORE_REP|Org54_Gene1613#

MGSDTAWSPARMIGIAALAVGIVLGLVFHPGVPEVIQPYLPIAVVAALDAVFGGLRAYLERIFDPKVFVVSFVFNVLVAALIVYVGDQLGVGTQLSTAIIVVLGIRIFGNTAALRRRLFGA

>1029|CORE_REP|Org19_Gene3400#

MVSQSMYSYPAMTANVGDMAGYTGTTQSLGADIASERTAPSRACQGDLGMSHQDWQAQWNQAMEALARAYRRCRRALRQIGVLERPVGDSSDCGTIRVGSFRGRWLDPRHAGPATAADAGD

>1030|CORE_REP|Org131_Gene2802#

MSTQRPRHSGIRAVGPYAWAGRCGRIGRWGVHQEAMMNLAIWHPRKVQSATIYQVTDRSHDGRTARVPGDEITSTVSGWLSELGTQSPLADELARAVRIGDWPAAYAIGEHLSVEIAVAV

>1031|CORE_REP|Org95_Gene1054#

MKRALITGITGPDGSYLAKLPLKGYVAAGSPAEVYFCWATRNYRELYGLLAVNSIWFNHESPRHGETFMTRNPAPYRGRQRGADRCADADAPAHPDRYQYWGVPASVRGVIDRAMGVCVE

>1032|CORE_REP|Org63_Gene939#

MTAPETPAAQHAEPAIAVERIRTALLGYRIMAWTTGLWLIALCYEIVVRYVVKVDNPPTWIGVVHGWVYFTYLLLTLNLAVKVRWPLGKTAGVLLAGTIPLLGIVVEHFQTKEIKARFGL

>1033|CORE_REP|Org65_Gene3576#

MGHGVEGRNRPSAPLDSQAAAQVASTLQALATPSRLMILTQLRNGPLPVTDLAEAIGMEQSAVSHQLRVLRNLGLVVGDRAGRSIVYSLYDTHVAQLLDEAIYHSEHLHLGLSDRHPSAG

>1034|CORE_REP|Org2_Gene3708#

MLIPDAATEAFRRRAAMQCSQRSHRAVADVASWVATGFSGVPESFDELVGVMITVLDMNGFKDARPDRLPLSASVWDIAQRYNKGGPTVTEALYEALKELEAQVIALQRSEGKGLLSRLS

>1035|CORE_REP|Org2_Gene1154#

MPTYSYECTQCANRFDVVQAFTDDALTTCERCSGRLRKLFNAVGVVFKGTGFYRTDSRESGKKSKSQTNGSSTSESTKSSGVRGVLARVKARLRGQPRSQPAPQPPPRPSELRGYPQGDF

>1036|CORE_REP|Org59_Gene372#

MYKVIDIIGTSPTSWEQAAAEAVQRARDSVDDIRVARVIEQDMAVDSAGKITYRIKLEVSFKXRPAQPRXHGPASRRKIAXFAVDSCDFVSARRGLPGAAQXPRAAVSRRASRFRRPRRR

>1037|CORE_REP|Org148_Gene3226#

MDRVRRVVTDRDSGAGALARHPLAGRRTDPQLAAFYHRLMTTQRHCHTQATIAVARKLAERTRVTITTGRPYQLRDTNGDPVTARGAKELIDAHYHVDTRTHPHNRAHTDTMQNSKPAR

>1038|CORE_REP|Org9_Gene690#

MSKELTAKKRAALNRLKTVRGHLDGIVRMLESDAYCVDVMKQISAVQSSLERANRVMLHNHLETCFSTAVLDGHGQAAIEELIDAVKFTPALTGPHARLGGAAVGESATEEPMPDASNM

>1039|CORE_REP|Org86_Gene1744#

MPKSLPVIDISAPVCCAPVAAGPMSDGDALAVALRLKALADPARVKIMSYLFSSPAGEQVSGQLAAALSLSDGTVSHHLAQLRKAGLVISDRRGMHVFHRVHPEALQALCTVLNPNCCA

>1040|CORE_REP|Org12_Gene1447#

MTVQNEPSAKTHGVILTEAAAAKAKSLLDQEGRDDLALRIAVQPGGCAGLRYNLFFDDRTLDGDQTAEFGGVRLIVDRMSAPYVEGASIDFVDTIEKQGFTIDNPNATGSCACGDSFN

>1041|CORE_REP|Org137_Gene3228#

MSGLTSPKTYAVLAALQAGDAVACAIPLPPIARLLDDLDVPVSVRPVLPVVKAASAVGLLSVTRFPALARLTTAMLTLYFILAVGAHVRVRDRVVNAIPAASFLTLFALMTAKGPERT

>1042|CORE_REP|Org8_Gene1824#

MMRRGEIWQVDLDPARGSEANNQRPAVVVSNDRANATATRLGRGVITVVPVTSNIAKVYPFQVLLSATTTGLQVDCKAQAEQIRSIATERLLRPIGRVSAAELAQLDEALKLHLDLWS

>1043|CORE_REP|Org139_Gene2403#

MSGTRPAARRTNLTAAQNVVRSVDAEERIAWVSKALCRTTDPDELFVRGAAQRKAAVICRHCPVMQECAADALDNKVEFGVWGGMTERQRRALLKQHPEVVSWSDYLEKRKRRTGTAG

>1044|CORE_REP|Org138_Gene198#

MTLLNDIQVWTTACAYDHLIPGRGVGVLLDDGSQVALFRLDDGSVHAVGNVDPFSGAAVMSRGIVGDRGGRAMVQSPILKQAFALDDGSCLDDPRVSVPVYPARVTPEGRIQVARVAV

>1045|CORE_REP|Org59_Gene3512#

MSFDPAVADIGSQVVNNAFQGLQAGAVAWVSLSSLLPAGAEEVSAWAVTAXTTXGXXXAGAESSGAGRAEKGGRGVYRDRPDVFGRRRQGGRLLARSNSATRPDPRAGIVRRDARRIS

>1046|CORE_REP|Org4_Gene1721#

MESISLTSLAAEKLAEAQQTHSGRAAHTIHGGHTHELRQTVLALLAGHDLSEHDSPGEATLQVLQGHVCLTAGEDAWNGRAGDYVAIPPTRHALHAVEDSVIMLTVLKSLPDAHSGS

>1047|CORE_REP|Org1_Gene4270#

MTSNPSSSADQPLSGTTVPGSVPGKAPEEPPVKFTRAAAVWSALIVGFLILILLLIFIAQNTASAQFAFFGWRWSLPLGVAILLAAVGGGLITVFAGTARILQLRRAAKKTHAAALR

>1048|CORE_REP|Org20_Gene2124#

MWIGWLEFDVLLGDVRSLKQKRSVTRPLVAELQRKFSVSAAETGSHDLYRRAGIGVAVVSGDRSHAVDVLDKATIVVPHIRSSSCCPCAGACTALTTKWTGSQLCLPLPVASLALTT

>1049|CORE_REP|Org1_Gene3322#

MAGVTAAVSARLKADEARRPGFYAAGSGPLPQVRGSTLPVMELALQITLIVTSVLVVLLVLLHRAKGGGLSTLFGGGVQSSLSGSTVVEKNLDRLTLFVTGIWLVSIIGVALLIKYR

>1050|CORE_REP|Org24_Gene2896#

MRYAFAAEATTCNAFWRNVDMTVTALYEVPLGVCTQDPDRWTTTPDDEAKTLCRACPRRWLCARDAVESAGAEGLWAGVVIPESGRARAFALGQLRSLAERNGYPVRDHRVSAQSA

>1051|CORE_REP|Org99_Gene2756#

MTVFGIKPDNYFGDVVLAAADRDGLRIFQYAVRSAHESGQATFDIDGVQQRIVRESGTADMELGSQTVVWRFDDTKLVEILDKLSPLIDGEGPGHQYIDDLNSPAPTLMISVDEYA

>1052|CORE_REP|Org135_Gene38#

MTDSEKSATIKVTDASFATDVLSSNKPVLVDFWATWCGPCKMVAPVLEEIATERATDLTVAKLDVDTNPETARNFQVVSIPTLILFKDGQPVKRIVGAKGKAALLRELSDVVPNLN

>1053|CORE_REP|Org35_Gene64#

MFLAGVLCMCAAAASALFGSWSLFHTPTADPTALALRAMAPTQLAAAVMLAAGGVVAVAAPGHTALMVVIVCIAGAVGTLAAGSWQSAQYALRRETASPTANCVGSCAVCTQACH

>1054|CORE_REP|Org8_Gene1126#

MIRAVWNGTVLAEAPRTVRVEGNHYFPPESLHREHLIESPTTSICPWKGLAHYYNVVVDGPYGPVNPDAAWYYRRPSPLARRIKNHVAFWHGVTVEGESESRHGLARRVVAWLGK

>1055|CORE_REP|Org96_Gene1012#

MSETSAPAEELLADVEEAMRDVVDPELGINVVDLGLVYGLDVQDGDEGTVALIDMTLTSAACPLTDVIEDQSRSALVGSGLVDDIRINWVWNPPWGPDKITEDGREQLRALGFTV

>1056|CORE_REP|Org96_Gene1491#

MPVTQEEIIAGIAEIIEEVTGIEPSEITPEKSFVDDLDIDSLSMVEIAVQTEDKYGVKIPDEDLAGLRTVGDVVAYIQKLEEENPEAAQALRAKIESENPDAVANVQARLEAESK

>1057|CORE_REP|Org104_Gene1306#

MVISRAEIYWADLGPPSGSQPAKRRPVLVIQSDPYNASRLATVIAAVITSNTALAAMPGNVFLPATTTRLPRDSVVNVTAIVTLNKTDLTDRVGEVPASLMHEVDRGLRRVLDL

>1058|CORE_REP|Org44_Gene1330#

MTYVIGSECVDVMDKSCVQECPVDCIYEGARMLYINPDECVDCGACKPACRVEAIYWEGDLPDDQHQHLGDNAAFFHQVLPGRVAPLGSPGGAAAVGPIGVDTPLVAAIPVECP

>1059|CORE_REP|Org87_Gene4095#

MGADDTLRVEPAVMQGFAASLDGAAEHLAVQLAELDAQVGQMLGGWRGASGSAYGSAWELWHRGAGEVQLGLSMLAAAIAHAGAGYQHNETASAQVLREVGGGWAVSGGSDRVG

>1060|CORE_REP|Org76_Gene1314#

MGVNVLASTVSGAIERLGLTYEEVGDIVDASPRSVARWTAGQVVPQRLNKQRLIELAYVADALAEVLPRDQANVWMFSPNRLLEHRKPADLVRDGEYQRVLALIDAMAEGVFV

>1061|CORE_REP|Org147_Gene1881#

MNRLDFVDKPSLRDDIPAFNPGDTINVHVKVIEGAKERLQVFKGVVIRRQGGGIRETFTVRKESYGVGVERTFPVHSPNIDHIEVVTRGDVRRAKLYYLRELRGKKAKIKEKR

>1062|CORE_REP|Org2_Gene3285#

MCRNITELRGLQPPATPVEIAAAARQYVRKVSGITHPSAATAEAFEAAVAEVTATTTRLLDALPPGGSRRRPSRRCADPTWLPGSRDRGDASAEGVERGGARAAGRPADGAAA

>1063|CORE_REP|Org135_Gene1795#

MAALVREVVGDVLRGARMSQGRTLREVSDSARVSLGYLSEIERGRKEPSSELLSAICTALQLPLSVVLIDAGERMARQERLARATPAGRATGATIDASTKVVIAPVVSLAVA

>1064|CORE_REP|Org46_Gene2352#

MAKKVTVTLVDDFDGSGAADETVEFGLDGVTYEIDLSTKNATKLRGDLKQWVAAGRRVGGRRRGRSGSGRGRGAIDREQSAAIREWARRNGHNVSTRGRIPADVIDAYHAAT

>1065|CORE_REP|Org114_Gene1530#

MKGHLATFGHPALPTYRGSWLSREPGSPYRLPAGAGRDRGDACRRIPRRTGSGTLLRPGQRCTFAANADPMAKGVDRALCEIVAERRQLDLDLAKAQVRSALANQRYHRDVH

>1066|CORE_REP|Org27_Gene2474#

MMQFYDDGVVQLDRAALTLRRYHFPSGTAKVIPLDQIRGYQAESLGFLMARFNIWGRPDLRRWLPLDVYRPLKSTLVTLDVPGMRPKPACTPTRPKEFIALLDELLALHRT

>1067|CORE_REP|Org42_Gene976#

MSISQSDASLAAVPAVDQFDPSSGASGGYDTPLGITNPPIDELLDRVSSKYALVIYAAKRARQINDYYNQLGEGILEYVGPLVEPGLQEKPLSIALREIHADLLEHTEGE

>1068|CORE_REP|Org112_Gene2050#

MGWEFGVLLILIAVLAVFLAPRLIPRGPRGDLASGTLLVTGVSPRPDAGGQQYVTIAGIITGPTVNEYAVYQRMAVDVDQWPTVGQILPVVYSPKNPDNWTFTPNGPPVG

>1069|CORE_REP|Org24_Gene1714#

MVVRSILLFVLAAVAEIGGAWLVWQGVREQRGWLWAGLGVIALGVYGFFATLQPDAHFGRVLAAYGGVFVAGSLAWGMALDGFRPDRWDVIGALGCMAGVAVIMYAPRGH

>1070|CORE_REP|Org2_Gene2044#

MTSELSLVATGKGSNIMCGDQSDHVLQHWTVDISIDEHEGLTRAKARLRWREKELVGVGLARLNPADRNVPEIGDELSVARALSDLGKRMLKVSTHDIEAVTHQPARLLY

>1071|CORE_REP|Org89_Gene355#

MSFLLDPPLLFVCGVLIERRLPVDRRDAAEAAALGVFFGASFGLYHNVPGLGMLWRPFRAQNGRDFMWNSGVFSVDVARAEWPLHAMAAAIFATYPFFIKLGRRLGRRI

>1072|CORE_REP|Org20_Gene715#

MWGLLTVPAPAQARRADSSEFDPDRGWRLHPQVAVRPEPFGALLYHFGTRKLSFLKNRTILAVVQTLADYPDIRSACRGAGVDDCDQDPYLHALSVLAGSNMLVPRQTT

>1073|CORE_REP|Org63_Gene1816#

MFQISPEQWMHSAAQVTTQGEGLAVGHLSSDYRMQAAQFGWQGASAMALNAKMDDWLDASRALLTRIGDHAFGLQEAAIQHAAAEAERAQALAQVGVSADVVAGPRGV

>1074|CORE_REP|Org50_Gene1571#

MLLPLGPPLPPDAVVAKRAESGMLGGLSVPLSWGVAVPPDDYDHWAPAPEDGADVDVQAAEGADAEAAAMDEWDEWQAWNEWVAENAEPRFEVPRSSSSVIPHSPAAG

>1075|CORE_REP|Org57_Gene2282#

MREFQRAAVRLHILHHAADNEVHGAWLTQELSRHGYRVSPGTLYPTLHRLEADGLLVSEQRVVDGRARRVYRATPAGRAALTEDRRALEELAREVLGGQSHTAGNGT

>1076|CORE_REP|Org28_Gene3022#

MADTIQVTPQMLRSTANDIQANMEQAMGIAKGYLANQENVMNPATWSGTGVVASHMTATEITNELNKVLTGGTRLAEGLVQAAALMEGHEADSQTAFQALFGASHGS

>1077|CORE_REP|Org137_Gene2002#

MIYLYLLCAIFAEVVATSLLKSTEGFTRLWPTVGCLVGYGIAFALLALSISHGMQTDVAYALWSAIGTAAIVLVAVLFLGSPISVMKVVGVGLIVVGVVTLNLAGAH

>1078|CORE_REP|Org118_Gene1118#

MEGDGHEVFFVTTRPDSIGETAANLHEIGVTMSAHDDGVTPLITNVESPAHDLVSIVTSMLFSMHGELYKAIARQAHVIHESFVQTLQTSKTSYWLTELANRAGTST

>1079|CORE_REP|Org66_Gene928#

MWRYPLSTRLALPNTPGVASFAMTSSPSTVSTTLLSILRDDLNIDLTRVTPDARLVDDVGLDSVAFAVGMVAIEERLGVALSEEELLTCDTVGELEAAIAAKYRDE

>1080|CORE_REP|Org94_Gene2027#

MVIRGAVYRVDFGDAKRGHEQRGRRYAVVISPGSMPWSVVTVVPTSTSAQPAVFRPELEVMGTKTRFLVDQIRTIGIVYVHGDPVDYLDRDQMAKVEHAVARYLGL

>1081|CORE_REP|Org7_Gene3839#

MYRFACRTLMLAACILATGVAGLGVGAQSAAQTAPVPDYYWCPGQPFDPAWGPNWDPYTCHDDFHRDSDGPDHSRDYPGPILEGPVLDDPGAAPPPRLPVAAHSAR

>1082|CORE_REP|Org4_Gene1551#

MIFKGVREGKPYPEHGLSYRDWSQIPPQQIRLDELVTTTTVLALDRLLSEDSTFYGDLFPHAVKWRGTTYLEDGLHRAVRAALRNRTVLHARVFDMDASPGGRRS

>1083|CORE_REP|Org104_Gene516#

MKVHKGDTVLVISGKDKGAKGKVLQAYPDRNRVLVEGVNRIKKHTAISTTQRGARSGGIVTQEAPIHVSNVMVVDSDGKPTRIGYRVDEETGKRVRISKRNGKDI

>1084|CORE_REP|Org68_Gene683#

MRPEPPHHENAELAAMNLEMLESQPVPEIDTLREEIDRLDAEILALVKRRAEVSKAIGKARMASGGTRLVHSREMKVIERYSELGPDGKDLAILLLRLGRGRLGH

>1085|CORE_REP|Org3_Gene387#

MSEAPNDKTTRGVVDILVYATARLLLVVAVSAAIFGVARLIGLTEFPVVVATLFGLIIAMPLGIWVFSPLRRRATAALAVAGERRRAERERLRARLRGESLPEEQ

>1086|CORE_REP|Org120_Gene59#

MTDRIHVQPAHLRQAAAHHQQTADYLRTVPSSHDAIRESLDSLGPIFSELRDTGRELLELRKQCYQQQADNHADIAQNLRTSAAMWEQHERAASRSLGNIIDGSR

>1087|CORE_REP|Org132_Gene1028#

MNAPLRGQVYRCDLGYGAKPWLIVSNNARNRHTADVVAVRLTTTRRTIPTWVAMGPSDPLTGYVNADNIETLGKDELGDYLGEVTPATMNKINTALATALGLPWP

>1088|CORE_REP|Org89_Gene3095#

MVIRFDQIGSLVLSMKSLASLSFQRCLRENSSLVAALDRLDAAVDELSALSFDALTTPERDRARRDRDHHPWSRSRSQLSPRMAHGAVHQCQWPKAVWAVIDNP

>1089|CORE_REP|Org116_Gene1240#

MIPGEIFYGSGDIEMNAAALSRLQMRIINAGDRPVQVGSHVHLPQANRALSFDRATAHGYRLDIPAATAVRFEPGIPQIVGLVPLGGRREVPGLTLNPPGRLDR

>1090|CORE_REP|Org55_Gene1364#

MFVDVGLLHSGANESHYAGEHAHGGADQLSRGPLLSGMFGTFPVAQTFHDAVGAAHAQQMRNLHAHRQALITVGEKARHAATGFTDMDDGNAAELKAVVCSCAT

>1091|CORE_REP|Org87_Gene2963#

MFGQWEFDVSPTGGIAVASTEVEHFAGSQHEVDTAEVPSAAWGWSRIDHRTWHIVGLCIFGFLLAMLRGNHVGHVEDWFLITFAAVVLFVLARDLWGRRRGWIR

>1092|CORE_REP|Org121_Gene4019#

MRPDSVNSAGIDIAAVYAVADRFSAAAELIDDAIGNHLTRLAFGGACAGRGHASRGDALRCRLDRLAGELSVWSRAAVQIAFALRAGANRYAEADLCAAAARIG

>1093|CORE_REP|Org27_Gene1300#

MTYVLDTNVVSALRVPGRHPAVAAWADSVQVAEQFVVAITLAEIERGVIAKERTDPTQSEHLRRWFDDKVLRIFVFARRGTNLIMQPLAGHIGYSLYSGISWF

>1094|CORE_REP|Org63_Gene1189#

MGSTGGSQPMTANRGPAAISSGSNSGRVLDTARGILIALRRCPAETAFDELHNAAQRHRLPVFEIAWALVHLAVEGSTPCRSFVDAQSAARREWGQLFAHAAA

>1095|CORE_REP|Org12_Gene2877#

MTENLTVQPERLGVLASHHDNAAVDASSGVEAAAGLGESVAITHGPYCSQFNDTLNVYLTAHNALGSSLHTAGVDLAKSLRIAAKIYSEADEAWRKAIDGLFT

>1096|CORE_REP|Org108_Gene783#

MRPIHIAQLDKARPVLILTREVVRPHLTNVTVAPITTTVRGLATEVPVDAVNGLNQPSVVSCDNIQTIPVCDLGRQIGYLLASQEPALAEAIGNAFDLDWVVA

>1097|CORE_REP|Org47_Gene1279#

MSVLVAFSVTPLGVGEGVGEIVTEAIRVVRDSGLPNQTDAMFTVIEGDTWAEVMAVVQRAVEAVAARAPRVSAVIKVDWRPGVTDAMTQKVATVERYLLRPE

>1098|CORE_REP|Org148_Gene321#

MDSAMARAIRSGDDAEVADGLTRREHDILAFERQWWKFAGVKEEAIKELFSMSATRYYQVLNALVDRPEALAADPMLVKRLRRLRASRQKARAARRLGFEVT

>1099|CORE_REP|Org77_Gene2216#

MTPTACATVSTMTSVGVRALRQRASELLRRVEAGETIEITDRGRPVALLSPLPQGGPYEQLLASGEIERATLDVVDLPEPLDLDAGVELPSVTLARLREHER

>1100|CORE_REP|Org34_Gene1988#

MPQPEQLPGPNADIWNWQLQGLCRGMDSSMFFHPDGERGRARTQREQRAKEMCRRCPVIEACRSHALEVGEPYGVWGGLSESERDLLLKGTMGRTRGIRRTA

>1101|CORE_REP|Org59_Gene1951#

MGSLSPWHWAILAVVVIVLFGAKKLPDAARSLGKSLRIFKSEVRELQNENKAEASIETPTPVQSQRVDPSAASGQDSTEARPAXLGRRFSARRRSSQTAQPT

>1102|CORE_REP|Org48_Gene57#

MTDANPAFDTVHPSGHILVRSCRGGYMHSVSLSEAAMETDAETLAEAILLTADVSCLKALLEVRNEIVAAGHTPSAQVPTTDDLNVAIEKLLAHQLRRRNR

>1103|CORE_REP|Org109_Gene1236#

MDITATTEFSAMNLDGKTGIGWLGYIVIGGIAGWLASKIVKGGGSGILMNVVIGVVGAFGAGLVLNALGVDVNHGGYWFTFFVALGGAVVLLWIVGMVRKT

>1104|CORE_REP|Org50_Gene939#

MAVVSAPAKPGTTWQRESAPVDVTDRAWVTIVWDDPVNLMSYVTYVFQKLFGYSEPHATKLMLQVHNEGKAVVSAGSRESMEVDVSKLHAAGLWATMQQDR

>1105|CORE_REP|Org123_Gene512#

MAGQKIRIRLKAYDHEAIDASARKIVETVVRTGASVVGPVPLPTEKNVYCVIRSPHKYKDSREHFEMRTHKRLIDIIDPTPKTVDALMRIDLPASVDVNIQ

>1106|CORE_REP|Org133_Gene2091#

MAPVTDEQVELVRSLVAAIPLGRVSTYGDIAALAGLSSPRIVGWIMRTDSSDLPWHRVIRASGRPAQHLATRQLELLRAEGVLSVDGRVALSEIRYEFPPG

>1107|CORE_REP|Org55_Gene2079#

MSENCGPTDAHADHDDSHGGMGCAEVIAEVWTLLDGECTPETRERLRRHLEACPGCLRHYGLEERIKALIGTKCRGDRAPEGLRERLRLEIRRTTIIRGGP

>1108|CORE_REP|Org70_Gene3163#

MNADPVLSYNFDAIEYSVRQEIHTTAARFNAALQELRSQIAPLQQLWTREAAAAYHAEQLKWHQAASALNEILIDLGNAVRHGADDVAHADRRAAGAWAR

>1109|CORE_REP|Org58_Gene1232#

MRLTPHEQERLLLSYAAELARRRRARGLRLNHPEAIAVIADHILEGARDGRTVAELMASGREVLGRDDVMEGVPEMLAEVQVEATFPDGTKLVTVHQPIA

>1110|CORE_REP|Org34_Gene207#

MIAPGDIAPRRDSEHELYVAVLSNALHRAADTGRVITCPFIPGRVPEDLLAMVVAVEQPNGTLLPELVQWLHVAALGAPLGNAGVAALREAASVVTALLC

>1111|CORE_REP|Org128_Gene2920#

MPTDYDAPRRTETDDVSEDSLEELKARRNEAASAVVDVDESESAESFELPGADLSGEELSVRVVPKQADEFTCSSCFLVQHRSRLASEKNGVMICTDCAA

>1112|CORE_REP|Org58_Gene19#

MAEMKTDAATLAQEAGNFERISGDLKTQIDQVESTAGSLQGQWRGAAGTAAQAAVVRFQEAANKQKQELDEISTNIRQAGVQYSRADEEQQQALSSQMGF

>1113|CORE_REP|Org108_Gene3127#

MPFLVALSGIISGVRDHSMTVRLDQQTRQRLQDIVKGGYRSANAAIVDAINKRWEALHDEQLDAAYAAAIHDNPAYPYESEAERSAARARRNARQQRSAQ

>1114|CORE_REP|Org81_Gene392#

MNRFLTSIVAWLRAGYPEGIPPTDSFAVLALLCRRLSHDEVKAVANELMRLGDFDQIDIGVVITHFTDELPSPEDVERVRARLAAQGWPLDDVRDREEHA

>1115|CORE_REP|Org26_Gene2228#

MAKVNIKPLEDKILVQANEAETTTASGLVIPDTAKEKPQEGTVVAVGPGRWDEDGEKRIPLDVAEGDTVIYSKYGGTEIKYNGEEYLILSARDVLAVVSK

>1116|CORE_REP|Org19_Gene1665#

MSQISRDEVAHLARLARLALTETELDSFAGQLDAILTHVSQIQAVDVTGVQATDNPLKDVNVTRPDETVPCLTQRQVLDQAPDAVDGRFAVPQILGDEQ

>1117|CORE_REP|Org74_Gene3347#

MAFVLVCPDALAIAAGQLRHVGSVIAARNAVAAPATAELAPAAADEVSALTATQFNFHAAMYQAVGAQAIAMNEAFVAMLGASADSYAATEAANIIAVS

>1118|CORE_REP|Org45_Gene2078#

MNPANYLYLSVLLFTIGASGVLLRRNAIVMFMCVELMLNAVNLAFVTFARMHGHLDAQMIAFFTMVVAACEVVVGLAIIMTIFRTRKSASVDDANLLKG

>1119|CORE_REP|Org70_Gene3818#

MEKMSHDPIAADIGTQVSDNALHGVTAGSTALTSVTGLVPAGADEVSAQAATAFTSEGIQLLASNASAQDQLHRAGEAVQDVARTYSQIDDGAAGVFAE

>1120|CORE_REP|Org2_Gene2448#

MPPSSATPVVVFSVAVVVVCLIGGVAGSLWPRPAGRLRGGCYFAFMGVAWVLLAISAIANAVKGSLWWDIWSLGLLVLIPAVVYGKMRRSRRISSDQDR

>1121|CORE_REP|Org110_Gene2496#

MASRFMTDPHAMRDMAGRFEVHAQTVEDEARRMWASAQNISGAGWSGMAEATSLDTMAQMNQAFRNIVNMLHGVRDGLVRDANNYEQQEQASQQILSS

>1122|CORE_REP|Org67_Gene1303#

MSSRYLLSPAAQAHLEEIWDCTYDRWGVDQAEQYLRELQHAIDRAAANPRIGRACDEIRPGYRKLSAGSHTLFYRVTGEGTIDVVRVLHQRMDVDRNL

>1123|CORE_REP|Org118_Gene489#

MSRPQVELLTRAGCACVWVRVAEQLAELSSELGFDMMTIDVDVAASTGNPGLRAEFGDRLPVVLLDGREHSYWEVDEHRLRADIARSTFGSPPDKRLP

>1124|CORE_REP|Org53_Gene194#

MSTTAELAELHDLVGGLRRCVTALKARFGDNPATRRIVIDADRILTDIELLDTDVSELDLERAAVPQPSEKIAIPDTEYDREFWRDVDDEGVGGHRY

>1125|CORE_REP|Org117_Gene2166#

MTVRAVFRRTVGAQWPILLVGSIFAVGFVLAGANFWRRGALLIGIGVGVAAVLRLVLSEERAGLLVVRSKGIDFVTTVTVAAAMVYIASTIDPLGTG

>1126|CORE_REP|Org86_Gene215#

MSLLDAHIPQLVASQSAFAAKAGLMRHTIGQAEQAAMSAQAFHQGESSAAFQAAHARFVAAAAKVNTLLDVAQANLGEAAGTYVAADAAAASTYTGF

>1127|CORE_REP|Org107_Gene337#

MTRRASTDTPQIIMGAIGGVVTGYILWLAAISVGDGLTTVSQWSRVVLLLSVLVAVCGAAGGLRLRSRGKLAWSAFAFSLPIPPVVLTVAVLADIYL

>1128|CORE_REP|Org131_Gene2582#

MSDDHPYHVAITATAARDLQRLPEKIAAACVEFVFGPLLNNPHRLGKPLRNDLEGLHSARRGDYRVVYAIDDGHHRVEIIHIARRSASYRMNPCRPR

>1129|CORE_REP|Org2_Gene2748#

MDATAPLVGGTALIGYVAVLGLGYVLGAKAGRRRYEQIASTYRALTGSPVARSMIEGGRRKIANRISPDAGFVTLAEIDNQTAVVQRGVERQPKTAR

>1130|CORE_REP|Org107_Gene221#

MSQIMYNYPAMLGHAGDMAGYAGTLQSLGAEIAVEQAALQSAWQGDTGITYQAWQAQWNQAMEDLVRAYHAMSSTHEANTMAMMARDTAEAAKWGG

>1131|CORE_REP|Org35_Gene153#

MTAAHGYTQQKDNYAKRLRRVEGQVRGIARMIEEDKYCIDVLTQISAVTSALRSVALNLLDEHLSHCVTRAVAEGGPGADGKLAEASAAIARLVRS

>1132|CORE_REP|Org10_Gene3109#

MVVFFQILGFALFIFWLLLIARVVVEFIRSFSRDWRPTGVTVVILEIIMSITDPPVKVLRRLIPQLTIGAVRFDLSIMVLLLVAFIGMQLAFGAAA

>1133|CORE_REP|Org7_Gene3070#

MAEPFRVDPTVLADAVARMAEFGRHVEELVAEIESLVTRLHVTWTGEGAAAHAEAQRHWAAGEAMMRQALAQLTAAGQSAHANYTGAMATNLGMWS

>1134|CORE_REP|Org118_Gene2434#

MKTTLDLPDELMRAIKVRAAQQGRKMKDVVTELLRSGLSQTHSGAPIPTPRRVQVSPGALRWRGYPRTRNDAGACCRGLARPGGPVVVRILLDAAL

>1135|CORE_REP|Org119_Gene3594#

MSDQITYNPGAVSDFASDVGSRAGQLHMIYEDTASKTNALQEFFAGHGAQGFFDAQAQMLSGLQGLIETVGQHGTTTGHVLDNAIGNPHPPIAGLF

>1136|CORE_REP|Org41_Gene19#

MTEQQWNFAGIEAAASAIQGNVTSIHSLLDEGKQSLTKLAAAWGGSGSEAYQGVQQKWDATATELNNALQNLARTISEAGQAMASTEGNVTGMFA

>1137|CORE_REP|Org134_Gene3901#

MTINYQFGDVDAHGAMIRAQAGLLEAEHQAIIRDVLTAGDFWGGAGSAACQGFITQLGRNFQVIYEQANAHGQKVQAAGNNMAQTDSAVGSSWA

>1138|CORE_REP|Org77_Gene98#

MSARCQITGRTVGFGKAVSHSHRRTRRRWPPNIQLKAYYLPSEDRRIKVRVSAQGIKVIDRDGHRGRRRAARAGSAPAHFARQAGSSLRTAAIL

>1139|CORE_REP|Org118_Gene459#

MGTVMLVLLVAVLVTAVYAFVHAALQRPDAYTAADKLTKPVWLVILGAAVALASILYPVLGVLGMAMSRLCVRRVSGRRAAQASRDSGQVALTE

>1140|CORE_REP|Org119_Gene2505#

MTSLPVHQASQPMPCLARQPVDLPPWAGPRCGPYCPRARITLLQRTTIAKSNRKYYENGYPADVKLMPGHAAVVSNRAAARAGFALPCRKRQPD

>1141|CORE_REP|Org59_Gene1973#

MQQSLAVKTFEDLFAELXCDRARXRXADSTTVAALDGGVHALGKKLLEEAGEVWLXXEXESNDALAEEISQLLYWTQVLMISRGLSJDDVYRKL

>1142|CORE_REP|Org1_Gene809#

MPRSLKKGPFVDEHLLKKVDVQNEKNTKQVIKTWSRRSTIIPDFIGHTFAVHDGRKHVPVFVTESMVGHKLGEFAPTRTFKGHIKDDRKSKRR

>1143|CORE_REP|Org54_Gene11#

MPKSKVRKKNDFTVSAVSRTPMKVKVGPSSVWFVSLFIGLMLIGLIWLMVFQLAAIGSQAPTALNWMAQLGPWNYAIAFAFMITGLLLTMRWH

>1144|CORE_REP|Org122_Gene1851#

MRILPISTIKGKLNEFVDAVSSTQDQITITKNGAPAAVLVGADEWESLQETLYWLAQPGIRESIAEADADIASGRTYGEDEIRAEFGVPRRPH

>1145|CORE_REP|Org55_Gene2113#

MAIQVFLAKATTTVITGLAGVTAYEILKKAAAKAPLRQTAVSAAALGLRGTRKAEEAAESARLKVADVMAEARERIGEESPTPAISDLHDHDH

>1146|CORE_REP|Org59_Gene821#

MCSVIADQRRPDQPCGVGGCKTCQNGFVADIAEGKARKTRYVDHGWPTTDPDDHAVSELVTDRTGALSPFGELTFPVPSDDLPYIHPVTVINR

>1147|CORE_REP|Org2_Gene538#

MIPLPRSWQLTSAMLVGNAIGLLAGVACSVLVHARIRPDIVIAMVVGIPSAIGLLVILFSGRRWVTMLGAFILALAPGWFGVLVAIQVASSG

>1148|CORE_REP|Org7_Gene2198#

MMAEKNTRRATSQREAVAKIREAETIVMNLPICGQVKIPRPEHLAYYGGLAALAALELIDWPVALVIATGHILANNHHNRVLEELGEAMEEA

>1149|CORE_REP|Org78_Gene2202#

MSISASEARQRLFPLIEQVNTDHQPVRITSRAGDAVLMSADDYDAWQETVYLLRSPENARRLMEAVARDKAGHSAFTKSVDELREMAGGEE

>1150|CORE_REP|Org113_Gene2411#

MTTLKELGARVAALEANQADYRAVLAAVNPPGANQREIATTVREHTGRLDRVTTKVGQLAAKSDDTNARVRSLEEGQAEIKDLLLRALDK

>1151|CORE_REP|Org3_Gene2772#

MNEVSIRTLNQETSKVLARVKRGEEINLTERGKVIARIIPASAGPLDSLISTGSVQPARVHGPAPRPTIPMRGGLDSGTLLERMRAEERY

>1152|CORE_REP|Org2_Gene2987#

MYGDVMRTQVTLGKEELELLDRAAKASGASRSELIRRAIHRAYGTGSKQERLAALDHSRGSWRGRDFTGTEYVDAIRGDLNERLARLGLA

>1153|CORE_REP|Org11_Gene1632#

MSLNIKSQRTVALVRELAARTGTNQTAAVEDAVARRLSELDREDRARAEARRAAAEQTLRDLDKLLSDDDKRLIRRHEVDLYDDSGLPR

>1154|CORE_REP|Org57_Gene1830#

MALTAEQKKEILRSYGLHETDTGSPEAQIALLTKRIADLTEHLKVHKHDHHSRRGLLLLVGRRRRLIKYISQIDVERYRSLIERLGLRR

>1155|CORE_REP|Org2_Gene1466#

MTCMRTTLTLDDDVVQLVEDAVHRERRPMKQVINDALRRALAPPVKRQEQYRLEPHESAVRSGLDLAGFNKLADELEDEALLDATRRAR

>1156|CORE_REP|Org2_Gene3214#

MHRGYALVVCSPGVTRTMIDIDDDLLARAAKELGTTTKKDTVHAALRAALRASAARSLMNRMAENATGTQDEALVNAMWRDGHPENTA

>1157|CORE_REP|Org95_Gene1372#

MAAKSARKGPTKAKKNLLDSLGVESVDYKDTATLRVFISDRGKIRSRGVTGLTVQQQRQVAQAIKNAREMALLPYPGQDRQRRAALCP

>1158|CORE_REP|Org7_Gene357#

MKAKVGDWLVIKGATIDQPDHRGLIIEVRSSDGSPPYVVRWLETDHVATVIPGPDAVVVTAEEQNAADERAQHRFGAVQSAILHARGT

>1159|CORE_REP|Org65_Gene60#

MKEAINATIQRILRTDRGITANQVLVDDLGFDSLKLFQLITELEDEFDIAISFRDAQNIKTVGDVYTSVAVWFPETAKPAPLGKGTA

>1160|CORE_REP|Org10_Gene1206#

MSKRLQVLLDPDEWEELREIARRHRTTVSEWVRRTLREAREREPRGDLDMKLRSVRAAARHEFPTADVEQMLEEIERGRGAEREGSR

>1161|CORE_REP|Org117_Gene635#

MGKGRKPTDSETLAHIRDLVAEEKALRAQLRHGGISESEEQQQLRRIEIELDQCWDLLRQRRALRQTGGDPREAVVRPADQVEGYTG

>1162|CORE_REP|Org59_Gene608#

MSEVASRELRNDTAGVLRRPXRAGEDVTITVSGRPVAVLTPVRPRRRRWLSKTEFLSRLRGAQADPGLRNDLAVLAGDTTEDLGPIR

>1163|CORE_REP|Org9_Gene1586#

MAHKKGASSSRNGRDSAAQRLGVKRYGGQVVKAGEILVRQRGTKFHPGVNVGRGGDDTLFAKTAGAVEFGIKRGRKTVSIVGSTTA

>1164|CORE_REP|Org80_Gene2849#

MANIKSQQKRNRTNERARLRNKAVKSSLRTAVRAFREAAHAGDKAKAAELLASTNRKLDKAASKGVIHKNQAANKKSALAQALNKL

>1165|CORE_REP|Org148_Gene3747#

MRPSRQGEVGEVAGYVVEYNRRTHVRRITEFATPQEAMEHRLKLEAERTDSNIEIVALVSKSLGTLKQTHSRYFTGEELNVGNGAR

>1166|CORE_REP|Org43_Gene974#

MKRTNIYLDEEQTASLDKLAAQEGVSRAELIRLLLNRALTTAGDDLASDLQAINDSFGTLRHLDPPVRRSGGREQHLAQVWRATS

>1167|CORE_REP|Org12_Gene787#

MVCDPNGDDTGRTHATVPVSQLGYEACRDELMEVVRLLEQGGLDLDASLRLWERGEQLAKRCEEHLAGARQRVSDVLAGDEAQNG

>1168|CORE_REP|Org131_Gene2182#

MRSVNFDPDAWEDFLFWLAADRKTARRITRLIGEIQRDPFSGIGKPEPLQGELSGYWSRRIDDEHRLVYRAGDDEVTMLKARYHY

>1169|CORE_REP|Org104_Gene1374#

MTFTTVVLNPPLATLEVAVLDADRLRRAFRRIAGAALGKRLRELDRKDAKGHKGVPRAPKTPSPTANRRISDGPARRLTRCCRQS

>1170|CORE_REP|Org121_Gene439#

MSATIPARDLRNHTAEVLRRVAAGEEIEVLKDNRPVARIVPLKRRRQWLPAAEVIGELVRLGPDTTNLGEELRETLTQTTDDVRW

>1171|CORE_REP|Org59_Gene1955#

MRTTVTLDDDVEQLVRRRMAERQVSFKKALNDAIRDGASGRPAPSHCAAPARQTWCVPAVNXDRALQLAADLEDEELVRRQRRGS

>1172|CORE_REP|Org3_Gene449#

MALSIKHPEADRLARALAARTGETLTEAVVTALRERLARETGRARVVPLRDELAAIRHRCAALPVVDNRSAEAILGYDERGLPA

>1173|CORE_REP|Org147_Gene2080#

MDWRHKAVCRDEDPELFFPVGNSGPALAQIADAKLVCNRCPVTTECLSWALNTGQDSGVWGGMSEDERRALKRRNARTKARTGV

>1174|CORE_REP|Org4_Gene73#

MAKSSKRRPAPEKPVKTRKCVFCAKKDQAIDYKDTALLRTYISERGKIRARRVTGNCVQHQRDIALAVKNAREVALLPFTSSVR

>1175|CORE_REP|Org1_Gene3611#

MITAALTIYTTSWCGYCLRLKTALTANRIAYDEVDIEHNRAAAEFVGSVNGGNRTVPTVKFADGSTLTNPSADEVKAKLVKIAG

>1176|CORE_REP|Org14_Gene463#

MGSDCGCGGYLWSMLKRVEIEVDDDLIQKVIRRYRVKGAREAVNLALRTLLGEADTAEHGHDDEYDEFSDPNAWVPRRSRDTG

>1177|CORE_REP|Org26_Gene709#

MSTKYYLQKVPVEAVQPGFSLAIPHDGDYRLFQVDCTQMCQRSGQPVMIRLMSESVDGGQPWVLEYEAGTAVIRLLGVCQAAS

>1178|CORE_REP|Org150_Gene1256#

MGKNTSFVLDEHYSAFIDGEIAAGRYRSASEVIRSALRLLEDRETQLRALREALEAGERSGSSTPFDFDGFLGRKRADASRGR

>1179|CORE_REP|Org19_Gene1116#

MKTAISLPDETFDRVSRRASELGMSRSEFFTKAAQRYLHELDAQLLTGQIDRALESIHGTDEAEALAVANAYRVLETMDDEW

>1180|CORE_REP|Org116_Gene2833#

MSGMQTQTIERTDADERVDDGTGSDTPKYFHYVKKDKIAESAVMGSHVVALCGEVFPVTRAPKPGSPVCPDCKRIYDTLKKG

>1181|CORE_REP|Org6_Gene2674#

MAEPVRDRILAAVCDVLYIDEADLIDGDETDLRDLGLDSVRFVLLMKQLGVNRQSELPSRLAANPSIAGWLRELEAVCTEFG

>1182|CORE_REP|Org119_Gene1116#

MAVQCRVWLEIQWRGMLGADQARAGGPARIWREHSMAAMKPRTGDGPLEATKEGRGIVMRVPLEGWRSPGRRADTRRSRRTG

>1183|CORE_REP|Org48_Gene919#

MDPTIAAGALIGGGLIMAGGAIGAGIGDGVAGNALISGVARQPEAQGRLFTPFFITVGLVEAAYFINLAFMALFVFATPVK

>1184|CORE_REP|Org48_Gene447#

MALNIKDPSVHQAVKQIAKITGESQARAVATAVNERLARLRSDDLAARLLAIGHKTASRMSPEAKRLDHDALLYDERGLPA

>1185|CORE_REP|Org75_Gene1720#

MRTTIDVAGRLVIPKRIRERLGLRGNDQVEITERDGRIEIEPAPTGVELVREGSVLVARPERPLPPLTDEIVRETLDRTRR

>1186|CORE_REP|Org2_Gene2513#

MHNSGMRTTVSLADDVAAAVQRLRKERSIGLSEAVNELIRAGLTKRQVANRFQQQTYDMGEGIDYSNIGDAIETLDGPASG

>1187|CORE_REP|Org119_Gene3459#

MSDCNVLGGALEQGGTDPLTGFYRDGCCATGPEDLGWHTICAVMTTEFLAHQRSVGNDLSIAAPATVAAPLRWCPLRRWGL

>1188|CORE_REP|Org132_Gene902#

MKSDIHPAYEETTVVCGCGNTFQTRSTKPGGRIVVEVCSQCHPFYTGKQKILDSGGRVARFEKRYGKRKVGADKAVSTGK

>1189|CORE_REP|Org90_Gene2874#

MVAADHRALGSNKSYPASQTAEAIWPPARTLRYDRQSPWLATGFDRRMSQTVTGVGVQNCAVSKRRCSAVDHSSRTPYRR

>1190|CORE_REP|Org10_Gene2664#

MYSGVVSRTNIEIDDELVAAAQRMYRLDSKRSAVDLALRRLVGEPLGRDEALALQGSGFDFSNDEIESFSDTDRKLADES

>1191|CORE_REP|Org134_Gene1180#

MRDTTFGPVVTRLCGWTYALSVVLLWVTCTAYAVLIAVSTTRIVIFRKEFADDLADPRRGFGMFTFVAASDVLGTRLVGQ

>1192|CORE_REP|Org38_Gene1186#

MTNVGDQGVDAVFGVIYPPQVALVSFGKPAQRVCAVDGAIHVMTTVLATLPADHGCSDDHRGALFFLSINELTRCAAVTG

>1193|CORE_REP|Org6_Gene3203#

MFRVESNGNPDLVPVETLHSGDPITDVNGGGQRYIVLESKTVGDSCVVLELESRVNHQLQVIEKSFPAGYHVGRAHHRIL

>1194|CORE_REP|Org105_Gene1998#

MTVTVYTKPACVQCSATSKALDKQGIAYQKVDISLDSEARDYVMALGYLQAPVVVAGNDHWSGFRPDRIKALAGAALTA

>1195|CORE_REP|Org1_Gene3640#

MAEKSGVMMAEDVRAEIVASVLEVVVNEGDQIDKGDVVVLLESMKMEIPVLAEAAGTVSKVAVSVGDVIQAGDLIAVIS

>1196|CORE_REP|Org56_Gene360#

MTSTNGPSARDTGFVEGQQAKTQLLTVAEVAALMRVSKMTVYRLVHNGELPAVRVGRSFRVHAKAVHDMLETSYFDAG

>1197|CORE_REP|Org150_Gene431#

MKAVVDAAGRIVVPKPLREALGLQPGSTVEISRYGAGLHLIPTGRTARLEEENGVLVATGETTIDDEVVFGLIDSGRK

>1198|CORE_REP|Org136_Gene1061#

MTAALHNDVVTVASAPKLRVVRDVPPAPASKKVARRLDAQPFGTGGDPLVDGAARLLSIPLRHLYAALWRVGLLEVQA

>1199|CORE_REP|Org114_Gene791#

MAAYQKFGQEHAAAIRGGAVLHPTATATTVRVTGARGGDVVTGDGPYEAADLDEQGPFPMETVYLWEDGPNGTTRMTL

>1200|CORE_REP|Org91_Gene2810#

MLRCRRGAGYGSVVVVGERPGFQSDSAARQTAPPVRPMTSDQLPATKADLYAAVDAMRADMRELLEQISTLIREATQK

>1201|CORE_REP|Org31_Gene3952#

MYDRTQVQLPDELYRDAKRVAHEHEMTLAEVVRRGLEHMVRIYPRRDAASDTWQPPTPRRLGPFRASEETWRELANEA

>1202|CORE_REP|Org48_Gene1372#

MSTSTTIRVSTQTRDRLAAQARERGISMSALLTELAAQAERQAIFRAEREASHAETTTQAVRDEDREWEGTVGDGLG

>1203|CORE_REP|Org131_Gene1619#

MEIHLFFVGIPLLLVVVLSVLIWSRKGPHPATYKLSEPWTHPPILWAATDEVVGSAHGGHGHDASEFTVGGGASGTW

>1204|CORE_REP|Org61_Gene1718#

MEVRASARKHGINDDAMLHAYRNALRYVELEYHGEVQLLVIGPDQTGRLLELVIPADEPPRIIHANVLRPKFYDYLR

>1205|CORE_REP|Org50_Gene3490#

MKLSVSLSDDDVAILDAYVKRAGLPSRSAGLQHAIRVLRYPTLEDDYANAWQEWSAAGDTDAWEQTVGDGVGDAPR

>1206|CORE_REP|Org53_Gene448#

MRTTIDLPQDLHKQALAIARDTHRTLSETVADLMRRGLAANRPTALSSDPRTGLPLVSVGTVVTSEDVRSLEDEQ

>1207|CORE_REP|Org126_Gene1169#

MSAMVQIRNVPDELLHELKARAAAQRMSLSDFLLARLAEIAEEPALDDVLDRLAALPRRDLGASAAELVDEARSE

>1208|CORE_REP|Org67_Gene1666#

MVDRDPNTIKQEIDQTRDQLAATIDSLAERANPRRLADDAKTRVIAFLRKPIVTVSLVGIGSVVVVVVIHKIRNR

>1209|CORE_REP|Org7_Gene3226#

MEQIVIRNLPEGTKAALRVRAARHHHSVEAEARAILTAGLLGEEVPMPVLLAADSGHDIDFEPERLGLIARTPQL

>1210|CORE_REP|Org2_Gene3287#

MTRKMTATEVKAKILSLLDEVAQGEEIEITKHGRTVARLVAATGPHALKGRFSGVAMAAVDDDELFTTGVSWNVS

>1211|CORE_REP|Org88_Gene1671#

MRTTLQIDDDVLEDARSIARSEGKSVGAVISELARRSLRPVGIVEVDGFPVFDVPPDAPTVTSEDVVRALEDDV

>1212|CORE_REP|Org19_Gene2037#

MRTTIDLDDDILRALKRRQREERKTLGQLASELLAQALAAEPPPNVDIRWSTADLRPRVDLDDKDAVWAILDRG

>1213|CORE_REP|Org57_Gene697#

MKTLYLRNVPDDVVERLERLAELAKTSVSAVAVRELTEASRRADNPALLGDLPDIGIDTTELIGGIDAERAGR

>1214|CORE_REP|Org69_Gene2244#

MAKKDGAIEVEGRVVEPLPNAMFRIELENGHKVLAHISGKMRQHYIRILPEDRVVVELSPYDLSRGRIVYRYK

>1215|CORE_REP|Org129_Gene1269#

MSFNPKDAVDAVRDIAANAVEKASDIVENAGHIIRGDIAGGASGIVKDSIDIATHAVDRTKEVFTGKTDDEG

>1216|CORE_REP|Org59_Gene2991#

MATMAAVVGGGPQDEIPEADAVEQGRAVDFDDEAGLDTAYLSGGAGDRDASEADVVDQAFVVPVADDEEIDR

>1217|CORE_REP|Org118_Gene2017#

MVVNRALLASVDALSRDEQIELVEHINGNLAEGMHISEANQALIEARAKHTSEGRPWTTIDDFDKRIRARLG

>1218|CORE_REP|Org12_Gene1554#

MSTNPFDDDNGAFFVLVNDEDQHSLWPVFADIPAGWRVVHGEASRAACLDYVEKNWTDLRPKSLRDAMAED

>1219|CORE_REP|Org9_Gene2583#

MSNHTYRVIEIVGTSPDGVDAAIQGGLARAAQTMRALDWFEVQSIRGHLVDGAVAHFQVTMKVGFRLEDS

>1220|CORE_REP|Org55_Gene1503#

MSHDIATEEADDGALDRCVLCDLTGKRVDVKEATCTGRPATTFEQAFAVERDAGFDDFLHGPVGPRSTP

>1221|CORE_REP|Org26_Gene2827#

MFVIRLADGEEVHGECDELTINPATGVLTVCRVDGFEETTTHYSPSAWRSVTHRKRGVGVRPSLVSTAQ

>1222|CORE_REP|Org37_Gene2873#

MPAGSMLAGMREIDPGADVAPLDCSKVSKDDVGNPVAAGSVALLLADRVGSTHLGGRRGKSEQGLSRR

>1223|CORE_REP|Org150_Gene2270#

MLVITMFRVLVARMTALAVDESGMSTVEYAIGTIAAAAFGAILYTVVTGDSIVSALNRIIGRALSTKV

>1224|CORE_REP|Org66_Gene300#

MIVVVNEQQVEVDEQTTIAALLDSLGFGDRGIAVALNFSVLPRSDWATKICELRKPVRLEVVTAVQGG

>1225|CORE_REP|Org82_Gene1386#

MLRRGESIIRNRYASKPPLYGMAMVFLAMAVVAVTAYFRMGWWSIIGYAAAAIIGVIGFALAFRDLS

>1226|CORE_REP|Org88_Gene1582#

MGPMNGFLSWWDGVELWLSGLPFALQALAVMPVVLALAYFTAALLDALLGRVIQLIRRARRPDQAPR

>1227|CORE_REP|Org2_Gene2087#

MKVRLDPSRCVGHAQCYAVDPDLFPIDDSGNSILAEHEVRPEDMQLTRDGVAACPEMALILEEDDAD

>1228|CORE_REP|Org19_Gene440#

MSQLKITQVRSTIGARWKQRESLRTLGLRRIRHSVIREDNAATRGLIAVVRHLVEVEPAQTGGKT

>1229|CORE_REP|Org150_Gene10#

MNCALGFDTKPILLASYVTHGARRATANQFERPAKGAGVLMALLILGEMAGFAVVVTGVVFGQLV

>1230|CORE_REP|Org55_Gene790#

MRTTVTVDDALLAKAAELTGVKEKSTLLREGLQTLVRVESARRLAALGGTDPQATAAPRRRTSPR

>1231|CORE_REP|Org15_Gene2333#

MFVQATELQKVKRRFRNVRATRRNTELEGTRSTAATRADQNDYARGKITAAELGERVRRRYNIQ

>1232|CORE_REP|Org147_Gene1378#

MAQEQTKRGGGGGDDDDIAGSTAAGQERREKLTEETDDLLDEIDDVLEENAEDFVRAYVQKGGQ

>1233|CORE_REP|Org85_Gene1874#

MAAVCDICGKGPGFGKSVSHSHRRTSRRWDPNIQTVHAVTRPGGNKKRLNVCTSCIKAGKITRG

>1234|CORE_REP|Org67_Gene2730#

MSVKSKNGRLAARVLVALAALFAMIALTGSACLAEGPPLGRNPQGAPAPVGGTVIVAPMHSGV

>1235|CORE_REP|Org67_Gene1844#

MKTNPRYGPAFYSVMTVLFLALFVLNVCTHGSTLGLISTGGLAVLMGYIGYRGWSGKRHINRQ

>1236|CORE_REP|Org106_Gene110#

MTTMIMTFVVPQRVTRATKGRARSLLRVSRRLTDTFRAPLAWTPQERADRYVARMPIAVIAD

>1237|CORE_REP|Org119_Gene632#

MRHHIRPSISALDAILCPDRRIAVETCWRKAIQMDYETDTDTELVTETLVEEVSIDGMCGVY

>1238|CORE_REP|Org94_Gene527#

MAKKALVNKAAGKPRFAVRAYTRCSKCGRPRAVYRKFGLCRICLREMAHAGELPGVQKSSW

>1239|CORE_REP|Org23_Gene2067#

MNDYKLFRCIQCGFEYDEALGWPEDGIAAGTRWDDIPDDWSCPDCGAAKSDFEMVEVARS

>1240|CORE_REP|Org86_Gene587#

MGRGRAKAKQTKVARELKYSSPQTDFQRLQRELSGTGTDRLDGDGPSDDDSWNDEDDWRR

>1241|CORE_REP|Org111_Gene2569#

MGILDKVKNLLSQNADKVETVINKAGEFVDEQTQGNYSDAIHKLHDAASNVVGMSDQQS

>1242|CORE_REP|Org104_Gene1142#

MTIDPDQIRAEIDALLASLPDPADAENGPSLAELEGIARRLSEAHEVLLAALESAEKG

>1243|CORE_REP|Org136_Gene191#

MNRIVAPAAASVVVGLLLGAAAIFGVTLMVQQDKKPPLPGGDPSSSVLNRVEYGNRS

>1244|CORE_REP|Org2_Gene3785#

MMAAYRCPVCDYVYDEANGDAREGFPAGTGWDQIPDDWCCPDCAVREKVDFEKIGG

>1245|CORE_REP|Org106_Gene1160#

MGSLAAFKLGWLLSAMAPNVVLLTAFRVPQGLTMLTVFATGQAGQHRCRTFHVTP

>1246|CORE_REP|Org27_Gene2534#

MASSTDVRPKITLACEVCKHRNYITKKNRRNDPDRLELKKFCPNCGKHQAHRETR

>1247|CORE_REP|Org79_Gene2277#

MSTKSDHGEIGDVEPLADSTASQARRVVAAYANDADECRIFLSMLGIGPAKLES

>1248|CORE_REP|Org139_Gene1368#

MARTDIRPIVKLRSTAGTGYTYTTRKNRRNDPDRLILRKYDPILRRHVDFREER

>1249|CORE_REP|Org121_Gene2357#

MTQPTAWEYATVPLLTHATKQILDQWGADGWELVAVLPGPTGEQHVAYLKRPK

>1250|CORE_REP|Org12_Gene483#

MSVTQIDLDDEALADVMRIAAVHTKKEAVNLAMRDYVERFRRIEALARSRE

>1251|CORE_REP|Org2_Gene1201#

MAMTTVDNIVGLVIAVALMAFLFAALLFPEKF
