# Supplementary material for: Pangenome Analysis of Mycobacterium tuberculosis Reveals Core-Drug Targets and Screening of Promising Lead Compounds for Drug Discovery
Source: Antibiotics (Basel). 2020 Nov 17;9(11):819. doi: 10.3390/antibiotics9110819 (PMC7698547; doi:10.3390/antibiotics9110819)
Supplement: Supplementary file 1 [file antibiotics-09-00819-s001.zip › antibiotics-984987-supplementary/File S2.docx]

**File S2.** The essential and non-human homolog core proteins identified in genomes under study given in FASTA format below:

>2|CORE_REP|Org57_Gene4069#

MRRPNPGVRCGIVVNVTGPPPTIDRRYHDAVIVGLDNVVDKATRVHAAAWTKFLDDYLTRRPQRTGEDHCPLTHDDYRRFLAGKPDGVADFLAARGIRLPPGSPTDLTDDTVYGLQNLERQTFLQLLNTGVPEGKSIASFARRLQVAGVRVAAHTSHRNYGHTLDATGLAEVFAVFVDGAVTAELGLPAEPNPAGLIETAKRLGANPGRCVVIDSCQTGLRAGRNGGFALVIAVDAHGDAENLLSSGADAVVADLAAVTVGSGDAAISTIPDALQVYSQLKRLLTGRRPAVFLDFDGTLSDIVERPEAATLVDGAAEALRALAAQCPVAVISGRDLADVRNRVKVDGLWLAGSHGFELVAPDGSHHQNAAATAAIDGLAEAAAQLADALREIAGAVVEHKRFAVAVHYRNVADDSVDNLIAAVRRLGHAAGLRVTTGRKVVELRPDIAWDKGKALDWIGERLGPAEVGPDLRLPIYIGDDLTDEDAFDAVRFTGVGIVVRHNEHGDRRSAATFRLECPYTVCQFLSQLACDLQEAVQHDDPWTLVFHGYDPGQERLREALCAVGNGYLGSRGCGPESAESEAHYPGTYVAGVYNQLTDHIEGCTVDNESLVNLPNWLSLTFRIDGGAWFNVDTVELLSYRQTFDLRRATLTRSLRFRDAGGRVTTMTQERFASMNRPNLVALQTRIESENWSGTVDFRSLVDGGVHNTLVDRYRQLSSQHLTTAEIEVLADSVLLRTQTSQSGIAIAVAARSTLWRDGQRVDAQYRVARDTNRGGHDIQVTLSAGQSVTLEKVATIFTSRDAATLTAAISAQRCLGEAGRYAELCQQHVRAWARLWERCAIDLTGNTEELRLVRLHLLHLLQTISPHTAELDAGVPARGLNGEAYRGHVFWDALFVAPVLSLRMPKVARSLLDYRYRRLPAARRAAHRAGHLGAMYPWQSGSDGSEVSQQLHLNPRSGRWTPDPSDRAHHVGLAVAYNAWHYYQVTGDRQYLVDCGAELLVEIARFWVGLAKLDDSRGRYLIRGVIGPDEFHSGYPGNEYDGIDNNAYTNVMAVWVILRAMEALDLLPLTDRRHLIEKLGLTTQERDQWDDVSRRMFVPFHDGVISQFEGYSELAELDWDHYRHRYGNIQRLDRILEAEGDSVNNYQASKQADALMLLYLLSSDELIGLLARLGYRFAPTQIPGTVDYYLARTSDGSTLSAVVHAWVLARANRSNAMEYFRQVLRSDIADVQGGTTQEGIHLAAMAGSIDLLQRCYSGLELRDDRLVLSPQWPEALGPLEFPFVYRRHQLSLRISGRSATLTAESGDAEPIEVEWSQLRSNAVATCSGYGAGTPSKSVAAGDQCRTWWVDDLSWKGRPAAVSLIALGVVCSSPATKGTPPG

>3|CORE_REP|Org58_Gene4039#

MNFSVLPPEINSALIFAGAGPEPMAAAATAWDGLAMELASAAASFGSVTSGLVGGAWQGASSSAMAAAAAPYAAWLAAAAVQAEQTAAQAAAMIAEFEAVKTAVVQPMLVAANRADLVSLVMSNLFGQNAPAIAAIEATYEQMWAADVSAMSAYHAGASAIASALSPFSKPLQNLAGLPAWLASGAPAAAMTAAAGIPALAGGPTAINLGIANVGGGNVGNANNGLANIGNANLGNYNFGSGNFGNSNIGSASLGNNNIGFGNLGSNNVGVGNLGNLNTGFANTGLGNFGFGNTGNNNIGIGLTGNNQIGIGGLNSGTGNFGLFNSGSGNVGFFNSGNGNFGIGNSGNFNTGGWNSGHGNTGFFNAGSFNTGMLDVGNANTGSLNTGSYNMGDFNPGSSNTGTFNTGNANTGFLNAGNINTGVFNIGHMNNGLFNTGDMNNGVFYRGVGQGSLQFSITTPDLTLPPLQIPGISVPAFSLPAITLPSLTIPAATTPANITVGAFSLPGLTLPSLTIPAATTPANITVGAFSLPGLTLPSLNIPAATTPANITVGAFSLPGLTLPSLNIPAATTPANITVGAFSLPGLTLPSLNIPAATTPANITVGAFSLPGLTLPSLNIPAATTPANITVGAFSLPGLTLPSLNIPAATTPANITVGAFSLPGLTLPSLNIPAATTPANITVGAFSLPGLTLPSLNIPAATTPANITVGAFSLPGLTLPSLNIPAATTPANITVGAFSLPGLTLPSLNIPAATTPANITVSGFQLPPLSIPSVAIPPVTVPPITVGAFNLPPLQIPEVTIPQLTIPAGITIGGFSLPAIHTQPITVGQIGVGQFGLPSIGWDVFLSTPRITVPAFGIPFTLQFQTNVPALQPPGGGLSTFTNGALIFGEFDLPQLVVHPYTLTGPIVIGSFFLPAFNIPGIDVPAINVDGFTLPQITTPAITTPEFAIPPIGVGGFTLPQITTQEIITPELTINSIGVGGFTLPQITTPPITTPPLTIDPINLTGFTLPQITTPPITTPPLTIDPINLTGFTLPQITTPPITTPPLTIDPINLTGFTLPQITTPPITTPPLTIDPINLTGFTLPQITTPPITTPPLTIDPINLTGFTLPQITTPPITTPPLTIEPIGVGGFTTPPLTVPGIHLPSTTIGAFAIPGGPGYFNSSTAPSSGFFNSGAGGNSGFGNNGSGLSGWFNTNPAGLLGGSGYQNFGGLSSGFSNLGSGVSGFANRGILPFSVASVVSGFANIGTNLAGFFQGTTS

>4|CORE_REP|Org117_Gene2968#

MCDVLMQPVRTPRPSTNLRSKPLRPTGDGGVFPRLGRLIVRRPWVVIAFWVALAGLLAPTVPSLDAISQRHPVAILPSDAPVLVSTRQMTAAFREAGLQSVAVVVLSDAKGLGAADERSYKELVDALRRDTRDVVMLQDFVTTPPLRELMTSKDNQAWILPVGLPGDLGSTQSKQAYARVADIVEHQVAGSTLTANLTGPAATVADLNLTGQRDRSRIEFAITILLLVILLIIYGNPITMVLPLITIGMSVVVAQRLVAIAGLAGLGIANQSIIFMSGMMVGAGTDYAVFLISRYHDYLRQGADSDQAVKKALTSIGKVIAASAATVAITFLGMVFTQLGILKTVGPMLGISVAVVFFAAVTLLPALMVLTGRRGWIAPRRDLTRRFWRSSGVHIVRRPKTHLLASALVLVILAGCAGLARYNYDDRKTLPASVESSIGYAALDKHFPSNLIIPEYLFIQSSTDLRTPKALADLEQMVQRVSQVPGVAMVRGITRPAGRSLEQARTSWQAGEVGSKLDEGSKQIAVHTGDIDKLAGGANLMASKLGDVRAQVNRAISTVGGLIDALAYLQDLLGGNRVLGELEGAEKLIGSMRALGDTIDADASFVANNTEWASPVLGALDSSPMCTADPACASARTELQRLVTARDDGTLAKISELARQLQATRAVQTLAATVSGLRGALATVIRAMGSLGMSSPGGVRSKINLVNKGVNDLADGSRQLAEGVQLLVDQVKKMGFGLGEASAFLLAMKDTATTPAMAGFYIPPELLSYATGESVKAETMPSEYRDLLGGLNVDQLKKVAAAFISPDGHSIRYLIQTDLNPFSTAAMDQIDAITAAARGAQPNTALADAKVSVVGLPVVLKDTRDYSDHDLRLIIAMTVCIVLLILIVLLRAIVAPLYLIGSVIVSYLAALGIGVIVFQFLLGQEMHWSIPGLTFVILVAVGADYNMLLISRLREEAVLGVRSGVIRTVASTGGVITAAGLIMAASMYGLVFASLGSVVQGAFVLGTGLLLDTFLVRTVTVPAIAVLVGQANWWLPSSWRPATWWPLGRRRGRAQRTKRKPLLPKEEEEQSPPDDDDLIGLWLHDGLRL

>7|CORE_REP|Org26_Gene3910#

MGMRSAARMPKLTRRSRILIMIALGVIVLLLAGPRLIDAYVDWLWFGELGYRSVFTTMLATRIVVCLVAGVVVGGIVFGGLALAYRTRPVFVPDADNDPVARYRAVVLARLRLVGIGIPAAIGLLAGIVAQSYWARIQLFLHGGDFGVRDPQFGRDLGFYAFELPFYRLMLSYMLVSVFLAFVANLVAHYIFGGIRLSGRTGALSRSARVQLVSLVGVLVLLKAVAYWLDRYELLSHTRGGKPFTGAGYTDINAVLPAKLILMAIALICAAAVFSAIALRDLRIPAIGLVLLLLSSLIVGAGWPLIVEQISVKPNAAQKESEYISRSITATRQAYGLTSDVVTYRNYSGDSPATAQQVAADRATTSNIRLLDPTIVSPAFTQFQQGKNFYYFPDQLSIDRYLDRNGNLRDYVVAARELNPDRLIDNQRDWINRHTVYTHGNGFIASPANTVRGIANDPNQNGGYPEFLVNVVGANGTVVSDGPAPLDQPRIYFGPVISNTSADYAIVGRNGDDREYDYETNIDTKRYTYTGSGGVPLGGWLARSVFAAKFAERNFLFSNVIGSNSKILFNRDPAQRVEAVAPWLTTDSAVYPAIVNKRLVWIVDGYTTLDNYPYSELTSLSSATADSNEVAFNRLVPDKKVSYIRNSVKATVDAYDGTVTLYQQDEKDPVLKVWMQVFPGTVKPKSDIAPELAEHLRYPEDLFKVQRMLLAKYHVNDPVTFFSTSDFWDVPLDPNPTASSYQPPYYIVAKNIAKDDNSASYQLISAMNRFKRDYLAAYISASSDPATYGNLTVLTIPGQVNGPKLANNAITTDPAVSQDLGVIGRDNQNRIRWGNLLTLPVARGGLLYVEPVYASPGASDAASSYPRLIRVAMMYNDKVGYGPTVRDALTGLFGPGAGATATGIAPTEAAVPPSPAANPPPPASGPQPPPVTAAPPVPVGAVTLSPAKVAALQEIQAAIGAARDAQKKGDFAAYGSALQRLDEAITKFNDAG

>8|CORE_REP|Org1_Gene2938#

MQRFGTGSSRSWCGRAGTATIAAVLLASGALTGLPPAYAISPPTIDPGALPPDGPPGPLAPMKQNAYCTEVGVLPGTDFQLQPKYMEMLNLNEAWQFGRGDGVKVAVIDTGVTPHPRLPRLIPGGDYVMAGGDGLSDCDAHGTLVASMIAAVPANGAVPLPSVPRRPVTIPTTETPPPPQTVTLSPVPPQTVTVIPAPPPEEGVPPGAPVPGPEPPPAPGPQPPAVDRGGGTVTVPSYSGGRKIAPIDNPRNPHPSAPSPALGPPPDAFSGIAPGVEIISIRQSSQAFGLKDPYTGDEDPQTAQKIDNVETMARAIVHAANMGASVINISDVMCMSARNVIDQRALGAAVHYAAVDKDAVIVAAAGDGSKKDCKQNPIFDPLQPDDPRAWNAVTTVVTPSWFHDYVLTVGAVDANGQPLSKMSIAGPWVSISAPGTDVVGLSPRDDGLINAIDGPDNSLLVPAGTSFSAAIVSGVAALVRAKFPELSAYQIINRLIHTARPPARGVDNQVGYGVVDPVAALTWDVPKGPAEPPKQLSAPLVVPQPPAPAIWCRYGWPPGDWPGTIDRRCGVRYRDLDAAITEAAMKAQRSFGLALSWPRVTAVFLVDVLILAVASHCPDSWQADHHVAWWVGVGVAAVVTLLSVVSYHGITVISGLATWVRDWSADPGTTLGAGCTPAIDHQRRFGRDTVGVREYNGRLVSVIEVTCGESGPSGRHWHRKSPVPMLPVVAVADGLRQFDIHLDGIDIVSVLVRGGVDAAKASASLQEWEPQGWKSEERAGDRTVADRRRTWLVLRMNPQRNVAAVACRDSLASTLVAATERLVQDLDGQSCAARPVTADELTEVDSAVLADLEPTWSRPGWRHLKHFNGYATSFWVTPSDITSETLDELCLPDSPEVGTTVVTVRLTTRVGSPALSAWVRYHSDTRLPKEVAAGLNRLTGRQLAAVRASLPAPTHRPLLVIPSRNLRDHDELVLPVGQELEHATSSFVGQ

>9|CORE_REP|Org10_Gene4001#

MSGTPDDGDIGLIIAVKRLAAAKTRLAPVFSAQTRENVVLAMLVDTLTAAAGVGSLRSITVITPDEAAAAAAAGLGADVLADPTPEDDPDPLNTAITAAERVVAEGASNIVVLQGDLPALQTQELAEAISAARHHRRSFVADRLGTGTAVLCAFGTALHPRFGPDSSARHRRSGAVELTGAWPGLRCDVDTPADLTAARQLGGRARDRASGRTSLTGTGQRRRGIQGGERQTNGERMPAECWQPHPMMSNDRKVTEIENSPVTEVRPEEHAWYPDDSALAAPPAATPAAISDQLPSDRYLNRELSWLDFNARVLALAADKSMPLLERAKFLAIFASNLDEFYMVRVAGLKRRDEMGLSVRSADGLTPREQLGRIGEQTQQLASRHARVFLDSVLPALGEEGIYIVTWADLDQAERDRLSTYFNEQVFPVLTPLAVDPAHPFPFVSGLSLNLAVTVRQPEDGTQHFARVKVPDNVDRFVELAAREASEEAAGTEGRTALRFLPMEELIAAFLPVLFPGMEIVEHHAFRITRNADFEVEEDRDEDLLQALERELARRRFGSPVRLEIADDMTESMLELLLRELDVHPGDVIEVPGLLDLSSLWQIYAVDRPTLKDRTFVPATHPAFAERETPKSIFATLREGDVLVHHPYDSFSTSVQRFIEQAAADPNVLAIKQTLYRTSGDSPIVRALIDAAEAGKQVVALVEIKARFDEQANIAWARALEQAGVHVAYGLVGLKTHCKTALVVRREGPTIRRYCHVGTGNYNSKTARLYEDVGLLTAAPDIGADLTDLFNSLTGYSRKLSYRNLLVAPHGIRAGIIDRVEREVAAHRAEGAHNGKGRIRLKMNALVDEQVIDALYRASRAGVRIEVVVRGICALRPGAQGISENIIVRSILGRFLEHSRILHFRAIDEFWIGSADMMHRNLDRRVEVMAQVKNPRLTAQLDELFESALDPCTRCWELGPDGQWTASPQEGHSVRDHQESLMERHRSP

>10|CORE_REP|Org118_Gene2197#

MVPGEVHMSDTPSGPHPIIPRTIRLAAIPILLCWLGFTVFVSVAVPPLEAIGETRAVAVAPDDAQSMRAMRRAGKVFNEFDSNSIAMVVLESDQPLGEKAHRYYDHLVDTLVLDQSHIQHIQDFWRDPLTAAGAVSADGKAAYVQLYLAGNMGEALANESVEAVRKIVANSTPPEGIRTYVTGPAALFADQIAAGDRSMKLITGLTFAVITVLLLLVYRSIATTLLILPMVFIGLGATRGTIAFLGYHGMVGLSTFVVNILTALAIAAGTDYAIFLVGRYQEARHIGQNREASFYTMYRGTANVILGSGLTSIAGATYCLSFARLTLFHTMGPPLAIGMLVSVAAALTLAPAIIAIAGRFGLLDPKRRLKTRGWRRVGTAVVRWPGPILATSVALALVGLLALPGYRPGYNDRYYLRAGTPVNRGYAAADRHFGPARMNPEMLLVESDQDMRNPAGMLVIDKIAKEVLHVSGVERVQAITRPQGVPLEHASIPFQISMMGATQTMSLPYMRERMADMLTMSDEMLVAINSMEQMLDLVQQLNDVTHEMAATTREIKATTSELRDHLADIDDFVRPLRSYFYWEHHCFDIPLCSATRSLFDTLDGVDTLTDQLRALTDDMNKMEALTPQFLALLPPMITTMKTMRTMMLTMRSTISGVQDQMADMQDHATAMGQAFDTAKSGDSFYLPPEAFDNAEFQQGMKLFLSPNGKAVRFVISHESDPASTEGIDRIEAIRAATKDAIKATPLQGAKIYIGGTAATYQDIRDGTKYDILIVGIAAVCLVFIVMLMITQSLIASLVIVGTVLLSLGTAFGLSVLIWQHFVGLQVHWTIVAMSVIVLLAVGSDYNLLLVSRFKEEGAGAGLKTGIIRAMAGTGASCHVGRSGIRVHHGVHGRQRTPRYRTGRHHHRARSTFRYPGGPIVHDAIHRSAARSLVLVAEHDPLETHRPGGAHTPGRSPNSAASAPGLICTSVP

>11|CORE_REP|Org105_Gene3458#

MTESPTAGPGGVPRADDADSDVPRYRYTAELAARLERTWQENWARLGTFNVPNPVGSLAPPDGAAVPDDKLFVQDMFPYPSGEGLHVGHPLGYIATDVYARYFRMVGRNVLHALGFDAFGLPAEQYAVQTGTHPRTRTEANVVNFRRQLGRLGFGHDSRRSFSTTDVDFYRWTQWIFLQIYNAWFDTTANKARPISELVAEFESGARCLDGGRDWAKLTAGERADVIDEYRLVYRADSLVNWCPGLGTVLANEEVTADGRSDRGNFPVFRKRLRQWMMRITAYADRLLDDLDVLDWPEQVKTMQRNWIGRSTGAVALFSARAASDDGFEVDIEVFTTRPDTLFGATYLVLAPEHDLVDELVAASWPAGVNPLWTYGGGTPGEAIAAYRRAIAAKSDLERQESREKTGVFLGSYAINPANGEPVPIFIADYVLAGYGTGAIMAVPGHDQRDWDFARAFGLPIVEVIAGGNISESAYTGDGILVNSDYLNGMSVPAAKRAIVDRLESAGRGRARIEFKLRDWLFARQRYWGEPFPIVYDSDGRPHALDEAALPVELPDVPDYSPVLFDPDDADSEPSPPLAKATEWVHVDLDLGDGLKPYSRDTNVMPQWAGSSWYELRYTDPHNSERFCAKENEAYWMGPRPAEHGPDDPGGVDLYVGGAEHAVLHLLYSRFWHKVLYDLGHVSSREPYRRLVNQGYIQAYAYTDARGSYVPAEQVIERGDRFVYPGPDGEVEVFQEFGKIGKSLKNSVSPDEICDAYGADTLRVYEMSMGPLEASRPWATKDVVGAYRFLQRVWRLVVDEHTGETRVADGVELDIDTLRALHRTIVGVSEDFAALRNNTATAKLIEYTNHLTKKHRDAVPRAAVEPLVQMLAPLAPHIAEELWLRLGNTTSLAHGPFPKADAAYLVDETVEYPVQVNGKVRGRVVVAADTDEETLKAAVLTDEKVQAFLAGATPRKVIVVAGRLVNLVI

>12|CORE_REP|Org122_Gene4011#

MRSQRLAGHLSAAARTIHALSLPIILFWVALTIVVNVVAPQLQSVARTHSVALGPHDAPSLIAMKRIGKDFQQFDSDTTAMVLLEGQEKLGDEAHRFYDVLVTKLSQDTTHVQHIENFWGDPLTAAGSQSADGKAAYVQLNLTGDQGGSQANESVAAVQRIVDSVPPPPGIKAYVTGPGPLGADRVVYGDRSLHTITGISIAVIAIMLFIAYRSLSAALIMLLTVGLELLAVRGIISTFAVNDLMGLSTFTVNVLVALTIAASTDYIIFLVGRYQEARATGQNREAAYYTMFGGTAHVVLASGLTVAGAMYCLGFTRLPYFNTLASPYFNTLASPCAIGLVTVMLASLTLAPAIIAVASRFGLFDPKRATTKRRWRRIGTVVVRWPGPVLAATLLIALIGLLALPKYQTNYNERYYIPSAAPSNIGYLASDRHFPQARMEPEVLMVEADHDLRNPTDMLILDRIAKTVFHTPGIARVQSITRPLGAPIDHSSIPFQLGMQSTMTIENLQNLKDRVADLSTLTDQLQRMIDITQRTQELTRQLTDATHDMNAHTRQMRDNANELRDRIADFDDFWRPLRSFTYWERHCFDIPICWSMRSLLNSMDNVDKLTEDLANLTDDTERMDTTQRQLLAQLDPTIATMQTVKDLAQTLTSAFSGLVTQMEDMTRNATVMGRTFDAANNDDSFYLPPEAFQNPDFQRGLKLFLSPDGTCARFVITHRGDPASAEGISHIDPIMQAADEAVKGTPLQAASIYLAGTSSTYKDIHEGTLYDVMIAVVASLCLIFIIMLGITRSVVASAVIVGTVALSLGSAFGLSVLIWQHILHMPLHWLVLPMAIIVMLAVGSDYNLLLIARFQEEIGAGLKTGMIRAMAGTGRVVTIAGLVFAFTMGSMVASDLRVVGQIGTTIMIGLLFDTLVVRSYMTPALATLLGRWFWWPRRVDRLARQPQVLGPRRTTALSAERAALLQ

>15|CORE_REP|Org2_Gene773#

MPQPRTHLPIPSAARTGLITYDAKDPDSTYPPIEQLRPPAGAPNVLLILLDDVGFGASSAFGGPCRTSTAELLAGNGLRYNRFHTTALCSPTRQALLTGRNHHSAGMGGITEIATGAPGYSSVLPNTMSPIARTLKLNGYNTAQFGKCHEVPVWQTSPVGPFDAWPSGGGGFEYFYGFIGGEANQWYPSLYEGTTPVEVNRTPEEGYHFMADMTDKALGWIGQQKALAPDRPFFVYFAPGATHAPHHVPREWADKYRGRFDVGWDALREETFARQKELGVIPADCQLTARHAEIPAWDDMPEDLKPVLCRQMEVYAGFLEYTDHHVGRLVDGLQSLGVLDDTLVFYIIGDNGASAEGTINGTYNEMLNFNGLADIETPRFMTDRLDKFGGPESYNHYSVGWAHAMDTPYQWTKQVASHWGGTRNGTIVHWPNGIAAKGEMRWQFHHVIDVAPTILEAAGLPEPLFVNGVQQHPIEGVSMAYSFDDAQAPDRHETQYFEMFGNRGIYHKGWTAVTKHKTPWILVGEQTVAFDDDVWELYDTTKDWSQAKDLAKEMPEKLHELQRLWLIEATRYNVLPLDDDTASRINPDLAGRPVLIRGNTQVLFSNMGRLSENCVLNLKNKSHTVTAEVEVPETGAEGVIVAQGASIGGWSLYANDGKLKYCYNLGGIKHFYAESADPLPAGAHQVRMEFAYAGGGLGKGGEVTLYVDGQQVGEGHVEATLAIVFSADDGCDVGMDSGSPVSPDYARGVTRSTGGSRACSSRSPRPPLLRAIWSTRSTRSASRWRANRAAQSNGEGTAMEKSRCHAVAHGGGCAGSAKSHKSGGRCGQGRGAGDSHGTRGAGRRYRAASAPHPLAVGAHLRDELAKRSADPRLTDELNDLAGHTLDDL

>16|CORE_REP|Org20_Gene3642#

MVLRVHDVTGASELTLGNTVDWEFAASVGERLARPAPPSTEYTRRQVIDELTVAAEKAEPPVRDVTGLIADGVVPPARVVDRPAWIRSAAESMRAMTHGSAKPRGFLTGRITGAQTGAVLAFVASGILGQYDPFGAAGEGCLLLVYPNVIAVERQLRVEPSDFRLWVCLHEVTHRVQFTANPWLSGYMSQALNLLTFEPVDDIGRVVSRLADFIRSRGHGTDDSEVNPSGILGLVRAVQSEPQRKALDQLLVLGTLLEGHAEHVMDAVGPMVVPSVATIRRRFDDRRHHKQPPLQRLVRALLGFDAKLSQYTRGKAFVDHVVDRAGMKLFNTIWYATTCRCLPRSKTRSDGSTECCSAAACGCGAVRSGPSRRLRPLERGALRRPGLVGAHRIAARLWPTTALIVDHGLQPGSATVAETARIQAISLGCVDARVLCVQVGAAGGREAAARSARYSALEEHRDGPVLLAHTLDDQAETVLLGLGRGSGARSIAGMRPYDPPWCRPLLGVRRSVTHAACRELGLTAWQDPHNTDRRFTRTRLRTEVLPLLEDVLGGGVAEALARTATALREDTDLIDTIAAQALPGAAVAGSRGQELSTSALTALPDAVRRRVIRGWLLAGGATGLTDRQIRGVDRLVTAWRGQGGVAVGSSCAARWSPGGATAYLCCGASPFDARVGRWSRGVARCARDPELLGDHPRADGGALSGGHQVGAAHGRADSGPHRRARRADRQRLPRAVRYHRPGSAADHRAEGRGALRHRPGASDSRADPVRVHGGEFVWVIDILVGRGADPQGPRPRHPRPRRADRRGRRRLRPYAFVVVAEPDEPESAVIAGVHAAAQARCGARQRRNRVRGFRHSQRLRRGLRPGLRRTLP

>18|CORE_REP|Org116_Gene3997#

MSFVLAMPEVLGSAATDLAALGSVLGAADAAAAATTTGIVAAAQDEVSAAIAALFSAHGRAYQVASAQAAAVHAQFVEALSAGAGAYASAEAAGAAVLANPAQSVQQDLLAAVNAQSVALTGRPLIGNGANGAPGTGANGAPGGWLLGNGGAGGSAAAGSGLPGGAGGAAGLFGTGGAGGAGGSSTVGDGGAGGAGGSGGWLLGTGGVGGVGGLGAGAGGAGGVGGAGGLLGAGGHGGAGGLGAVTGGVGGAGGAGGLLAGLVGAGGGNGGAGGIGAGGVGGAGGAGGNAGLLAGPGGAGGTGGRGFLNDGGVGGAGGNAGLLFGAGGTGGSGGAGLGGDGGAGGAGGNAGVLFGNAGSGGTGGFGDTDGGAGGAGGDAGWLGSGGVGGAGGFGETGDGGVGGAGGKAGLLIGNGGAGGAGGQGAVTGGTGGAGGDGVLIGNGGNAGIGGTGPTAGDTGAGGISGLLLGADGFNAPASASPLHTLKQQALAAINAPTQTLTGRPLIGNGTPGAVGSGATGAPGGWLLGDGGAGGSGAAGSGAPGGAGGAAGLWGTGGAGGAGGSSAGGGGAGGAGGAGGWLLGDGGAGGIGGASTVLGGTGGGGGVGGLWGAGGAGGAGGTGLVGGDGGAGGAGGTGGLLAGLIGAGGGHGGTGGLSTNGDGGVGGAGGNAGMLAGPGGAGGAGGDGENLDTGGDGGAGGSAGLLFGSGGAGGAGGFGFLGGDGGAGGNAGLLLSSGGAGGFGGFGTAGGVGGAGGNAGWLGFGGAGGIGGIGGNANGGAGGNGGTGGQLWGSGGAGGEGGAALSVGDTGGAGGVGGSAGLIGTGGNGGNGGTGANAGSPGTGGAGGLLLGQNGLNGLP

>19|CORE_REP|Org118_Gene1392#

MTLTPEASKSVAQPPTQAPLTQEEAIASLGRYGYGWADSDVAGANAQRGLSEAVVRDISAKKNEPDWMLQSRLKALRIFDRKPIPKWGSNLDGIDFDNIKYFVRSTEKQAASWDDLPEDIRNTYDRLGIPEAEKQRLVAGVAAQYESEVVYHQIREDLEAQGVIFLDTDTGLREHPDIFKEYFGTVIPAGDNKFSALNTAVWSGGSFIYVPPGVHVDIPLQAYFRINTENMGQFERTLIIADEGSYVHYVEGCLPPGGELITTADGDLRPIESIRVGDFVTGHDGRPHRVTAVQVRDLDGELFTFTPMSPANAFSVTAEHPLLAIPRDEVRVMRKERNGWKAEVNSTKLRSAEPRWIAAKDVAEGDFLIYPKPKPIPHRTVLPLEFARLAGYYLAEGHACLTNGCESLIFSFHSDEFEYVEDVRQACKSLYEKSGSVLIEEHKHSARVTVYTKAGYAAMRDNVGIGSSNKKLSDLLMRQDETFLRELVDAYVNGDGNVTRRNGAVWKRVHTTSRLWAFQLQSILARLGHYATVELRRPGGPGVIMGRNVVRKDIYQVQWTEGGRGPKQARDCGDYFAVPIKKRAVREAHEPVYNLDVENPDSYLAYGFAVHNCTAPIYKSDSLHSAVVEIIVKPHARVRYTTIQNWSNNVYNLVTKRARAEAGATMEWIDGNIGSKVTMKYPAVWMTGEHAKGEVLSVAFAGEDQHQDTGAKMLHLAPNTSSNIVSKSVARGGGRTSYRGLVQVNKGAHGSRSSVKCDALLVDTVSRSDTYPYVDIREDDVTMGHEATVSKVSENQLFYLMSRGLTEDEAMAMVVRGFVEPIAKELPMEYALELNRLIELQMEGAVG

>20|CORE_REP|Org12_Gene3362#

MTKPAADASAVLTAEDTLVLASTATPVEMELIMGWLGQQRARHPDSKFDILKLPPRNAPPAALTALVEQLEPGFASSPQSGEDRSIVPVRVIWLPPADRSRAGKVAALLPGRDPYHPSQRQQRRILRTDPRRARVVAGESAKVSELRQQWRDTTVAEHKRDFAQFVSRRALLALARAEYRILGPQYKSPRLVKPEMLASARFRAGLDRIPGATVEDAGKMLDELSTGWSQVSVDLVSVLGRLASRGFDPEFDYDEYQVAAMRAALEAHPAVLLFSHRSYIDGVVVPVAMQDNRLPPVHMFGGINLSFGLMGPLMRRSGMIFIRRNIGNDPLYKYVLKEYVGYVVEKRFNLSWSIEGTRSRTGKMLPPKLGLMSYVADAYLDGRSDDILLQGVSICFDQLHEITEYAAYARGAEKTPEGLRWLYNFIKAQGERNFGKIYVRFPEAVSMRQYLGAPHGELTQDPAAKRLALQKMSFEVAWRILQATPVTATGLVSALLLTTRGTALTLDQLHHTLQDSLDYLERKQSPVSTSALRLRSREGVRAAADALSNGHPVTRVDSGREPVWYIAPDDEHAAAFYRNSVIHAFLETSIVELALAHAKHAEGDRVAAFWAQAMRLRDLLKFDFYFADSTAFRANIAQEMAWHQDWEDHLGVGGNEIDAMLYAKRPLMSDAMLRVFFEAYEIVADVLRDAPPDIGPEELTELALGLGRQFVAQGRVRSSEPVSTLLFATARQVAVDQELIAPAADLAERRVAFRRELRNILRDFDYVEQIARNQFVAREFKARQGRDRI

>21|CORE_REP|Org20_Gene1819#

MTDRVSVGNLRIARVLYDFVNNEALPGTDIDPDSFWAGVDKVVADLTPQNQALLNARDELQAQIDKWHRRRVIEPIDMDAYRQFLTEIGYLLPEPDDFTITTSGVDAEITTTAGPQLVVPVLNARFALNAANARWGSLYDALYGTDVIPETDGAEKGPTYNKVRGDKVIAYARKFLDDSVPLSSGSFGDATGFTVQDGQLVVALPDKSTGLANPGQFAGYTGAAESPTSVLLINHGLHIEILIDPESQVGTTDRAGVKDVILESAITTIMDFEDSVAAVDAADKVLGYRNWLGLNKGDLAAAVDKDGTAFLRVLNRDRNYTAPGGGQFTLPGRSLMFVRNVGHLMTNDAIVDTDGSEVFEGIMDALFTGLIAIHGLKASDVNGPLINSRTGSIYIVKPKMHGPAEVAFTCELFSRVEDVLGLPQNTMKIGIMDEERRTTVNLKACIKAAADRVVFINTGFLDRTGDEIHTSMEAGPMVRKGTMKSQPWILAYEDHNVDAGLAAGFSGRAQVGGHVDNAELMADMVETKIAQPRAGASTAWVPSPTAATLHALHYHQVDVAAVQQGLAGKRRATIEQLLTIPLAKELAWAPDEIREEVDNNCQSILGYVVRWVDQGVGCSKVPDIHDVALMEDRATLRISSQLLANWLRHGVITSADVRASLERMAPLVDRQNAGDVAYRPMAPNFDDSIAFLAAQELILSGAQQPNGYTEPSMPDVVGSLRPGPLRSRPHRTGPVTMRPASGPLWGHRPLAGESRPHRAEPVRSSCLTGAGLRRRGRRRRYG

>22|CORE_REP|Org51_Gene3937#

MAIAETDTEVHTPFEQDFEKDVAATQRYFDSSRFAGIIRLYTARQVVEQRGTIPVDHIVAREAAGAFYERLRELFAARKSITTFGPYSPGQAVSMKRMGIEAIYLGGWATSAKGSSTEDPGPDLASYPLSQVPDDAAVLVRALLTADRNQHYLRLQMSERQRAATPAYDFRPFIIADADTGHGGDPHVRNLIRRFVEVGVPGYHIEDQRPGTKKCGHQGGKVLVPSDEQIKRLNAARFQLDIMRVPGIIVARTDAEAANLIDSRADERDQPFLLGATKLDVPSYKSCFLAMVRRFYELGVKELNGHLLYALGDSEYAAAGGWLERQGIFGLVSDAVNAWREDGQQSIDGIFDQVESRFVAAWEDDAGLMTYGEAVADVLEFGQSEGEPIGMAPEEWRAFAARASLHAARAKAKELGADPPWDCELAKTPEGYYQIRGGIPYAIAKSLAAAPFADILWMETKTADLADARQFAEAIHAEFPEQMLAYNLSPSFNWDTTGMTDEEMRRFPEELGKMGFVFNFITYGGHQIDGVAAEEFATALRQDGMLALARLQRKMRLVESPYRTPQTLVGGPRSDAALAASSGRTATTKAMGKGSTQHQHLVQTEVPRKLLEEWLAMWSGHYQLKDKLRVQLRPQRAGSEVLELGIHGESDDKLANVIFQPIQDRRGRTILLVRDQNTFGAELRQKRLMTLIHLWLVHRFKAQAVHYVTPTDDNLYQTSKMKSHGIFTEVNQEVGEIIVAEVNHPRIAELLTPDRVALRKLITKEA

>23|CORE_REP|Org59_Gene1377#

MRWATVALLLFLAGLVAQLNGAPEAMWWTLYLACYLAGGWGSAWAGAQALRBKALDVDLLMIAAAVGAVAIGQIFDGALLIVIFATSGALDDIATRHTAESVKGLLDLAPDQAVVVQGDGSERVVAASELVVGDRVVVRPGDRIXADGAVLSGXXDVDQRSITGESMPVAKARGDEVFAGTVNGSGVLHLVVTRDPSQTVVARIVELVADASATKAKTQLFIEKIEQRYSLGMVAATLALIVIPLMFGADLRPVLLRAMTFMIVASPCAVVLATMPPLLSAIANAGRHGVLVKSAVVVXRLADTSIVALDKTGTLTRGIPRLASVAPLDPNVVDARRLLXXAXAAEQSSEHPLGRAIVAEARRRGIAIPPAKDFRAVPGCGVHALVGNDFVEIASPQSYRGAPLAXLAPLLSAGATAAIVLLDGVAIGVLGLTDQLRPDAVESVAAMAALTAAPPVLLTGDNGRAAWRVARNAGITDVRAALLPEQKVEVVRNLQAGGHQVLLVGDGVNDAPAMAAARAAVAMGAGADLTLQTADGVTIRDELHXIPTIIGLARQARRVVTVNLAIAATFIAVLVLWDLFGQLPLPLGVVGHEGSTVLVALNGMRLLTNRSWRXAXXXXXXGSXVAELTRAALGVPVTTRDLTAAYFQQTISANSNVLVYFWAPLCAPCDLFTPTYEASSRKHFDVVHGKVNIETEKDLASIAGVKLLPTLMAFKKGKLXLXXSRHRQSRDHGQSGATTPGIHLQVPGRRRYRPWNKDFILRR

>24|CORE_REP|Org59_Gene3550#

MEILVTGGAGFQGSHLTESLLANGHWVTVLDKSSRNAVRNMQGFRSHDRAAFISGSVTDGQTIDRAVRDHHVVFHLAAHVNVDQSLGDPESFLETNVMGTYRVLEAVRRYRNRLIYVSTCEVYGDGHNLKEGERLDEHAELKPNSPYGASKAAADRLCYSYFRSYGLDVTIVRPFNIFGVRQKAGRFGALIPRLVRQGINGEGLTIFGAGSATRDYLYVSDIVGAYNLVLRTPTLRGQAINFASGKDTRVRDIVEYVADKFGARIEHRDARPGEVQRFPADISLAKSIGXQPQVEIWDGIDRYINWAKDQPQYPYEQDGFSGSSVSLIXPXPPSSAGKVGRNGAELPAGRITSHSMVTVARRPVCPVTLTPGDPALASVXDLVDAWSAHDALAELVTMFGGAFPQTDHLEARLASLDKFSTAWDYRARARAARALHGEPVRCQDSGGGARWLIPRLDLPAKKRDAIVGLAQQLGXTLESTPQGTTFDHVLVIGTGRHSNLIRARWARELAKGRQVGHIVLAAASRRLLPSEDDAVAVCAPGARTEFELLAAAARDAFGLDVHPAVRYVRQRDDNPHRDSMVWRFAADTNDLGVPITLLEAPSPEPDSSRATSADTFTFTAHTLGMQDSTCLLVTGQPFVPYQNFDALRTLALPFGIQVETVGFGIDRYDGLGELDQQXPAKLLQEVRSTIRAARALLERMXGRRXHGYRSSAVMXCXXWXXGSCPIRLXTXPGGGHGRSPMLARYRTGLTCPSCVRTSKCR

>25|CORE_REP|Org45_Gene3055#

MSAEQPTIIYTLTDEAPLLATYAFLPIVRAFAEPAGIKIEASDISVAARILAEFPDYLTEEQRVPDNLAELGRLTQLPDTNIIKLPNISASVPQLVAAIKELQDKGYAVPDYPADPKTDQEKAIKERYARCLGSAVNPVLRQGNSDRRAPKAVKEYARKHPHSMGEWSMASRTHVAHMRHGDFYAGEKSMTLDRARNVRMELLAKSGKTIVLKPEVPLDDGDVIDSMFMSKKALCDFYEEQMQDAFETGVMFSLHVKATMMKVSHPIVFGHAVRIFYKDAFAKHQELFDDLGVNVNNGLSDLYSKIESLPASQRDEIIEDLHRCHEHRPELAMVDSARGISNFHSPSDVIVDASMPAMIRAGGKMYGADGKLKDTKAVNPESTFSRIYQEIINFCKTNGQFDPTTMGTVPNVGLMAQQAEEYGSHDKTFEIPEDGVANIVDVATGEVLLTENVEAGDIWRMCIVKDAPIRDWVKLAVTRARISGMPVLFWLDPYRPHENELIKKVKTYLKDHDTEGLDIQIMSQVRSMRYTCERLVRGLDTIAATGNILRDYLTDLFPILELGTSAKMLSVVPLMAGGGMYETGAGGSAPKHVKQLVEENHLRWDSLGEFLALGAGFEDIGIKTGNERAKLLGKTLDAAIGKLLDNDKSPSRKTGELDNRGSQFYLAMYWAQELAAQTDDQQLAEHFASLADVLTKNEDVIVRELTEVQGEPVDIGGYYAPDSDMTTAVMRPSKTFNAALEAVQG

>27|CORE_REP|Org67_Gene624#

MPDNTIQWDKDADGIVTLTMDDPSGSTNVMNEAYIESMGKAVDRLVAEKDSITGVVVASAKKTFFAGGDVKTMIQARPEDAGDVFNTVETIKRQLRTLETLGKPVVAAINGAALGGGLEIALACHHRIAADVKGSQLGLPEVTLGLLPGGGGVTRTVRMFGIQNAFVSVLAQGTRFKPAKAKEIGLVDELVATVEELVPAAKAWIKEELKANPDGAGVQPWDKKGYKMPGGTPSSPGLAAILPSFPSNLRKQLKGAPMPAPRAILAAAVEGAQVDFDTASRIESRYFASLVTGQVAKNMMQAFFFDLQAINAGGSRPEGIGKTPIKRIGVLGAGMMGAGIAYVSAKAGYEVVLKDVSLEAAAKGKGYSEKLEAKALERGRTTQERSDALLARITPTADAADFKGVDFVIEAVFENQELKHKVFGEIEDIVEPNAILGSNTSTLPITGLATGVKRQEDFIGIHFFSPVDKMPLVEIIKGEKTSDEALARVFDYTLAIGKTPIVVNDSRGFFTSRVIGTFVNEALAMLGEGVEPASIEQAGSQAGYPAPPLQLSDELNLELMHKIAVATRKGVEDAGGTYQPHPAEAVVEKMIELGRSGRLKGAGFYEYADGKRSGLWPGLRETFKSGSSQPPLQDMIDRMLFAEALETQKCLDEGVLTSTADANIGSIMGIGFPPWTGGSAQFIVGYSGPAGTGKAAFVARARELAAAYGDRFLPPESLLS

>28|CORE_REP|Org2_Gene7#

MGKNEARRSALAPDHGTVVCDPLRRLNRMHATPEESIRIVAAQKKKAQDEYGAASITILEGLEAVRKRPGMYIGSTGERGLHHLIWEVVDNAVDEAMAGYATTVNVVLLEDGGVEVADDGRGIPVATHASGIPTVDVVMTQLHAGGKFDSDAYAISGGLHGVGVSVVNALSTRLEVEIKRDGYEWSQVYEKSEPLGLKQGAPTKKTGSTVRFWADPAVFETTEYDFETVARRLQEMAFLNKGLTINLTDERVTQDEVVDEVVSDVAEAPKSASERAAESTAPHKVKSRTFHYPGGLVDFVKHINRTKNAIHSSIVDFSGKGTGHEVEIAMQWNAGYSESVHTFANTINTHEGGTHEEGFRSALTSVVNKYAKDRKLLKDKDPNLTGDDIREGLAAVISVKVSEPQFEGQTKTKLGNTEVKSFVQKVCNEQLTHWFEANPTDAKVVVNKAVSSAQARIAARKARELVRRKSATDIGGLPGKLADCRSTDPRKSELYVVEGDSAGGSAKSGRDSMFQAILPLRGKIINVEKARIDRVLKNTEVQAIITALGTGIHDEFDIGKLRYHKIVLMADADVDGQHISTLLLTLLFRFMRPLIENGHVFLAQPPLYKLKWQRSDPEFAYSDRERDGLLEAGLKAGKKINKEDGIQRYKGLGEMDAKELWETTMDPSVRVLRQVTLDDAAAADELFSILMGEDVDARRSFITRNAKDVRFLDV

>32|CORE_REP|Org131_Gene298#

MADSSAIYLAAPESQTGKSTIALGLLHRLTAMVAKVGVFRPITRLSAERDYILELLLAHTSAGLPYERCVGVTYQQLHADRDDAIAEIVDSYHAMADECDAVVVVGSDYTDVTSPTELSVNARIAVNLGAPVLLTVRAKDRTPDQVASVVEVCLAELDTQRAHTAAVVANRCELSAIPAVTDALRRFTPPSYVVPEEPLLSAPTVAELTQAVNGAVVSGDVALREREVMGVLAAGMTADHVLERLTDGMAVITPGDRSDVVLAVASAHAAEGFPSLSCIVLNGGFQLHPAIAALVSGLRLRLPVIATALGTYDTASAAASARGLVTATSQRKIDTALELMDRHVDVAGLLAQLTIPIPTVTTPQMFTYRLLQQARSDLMRIVLPEGDDDRILKSAGRLLQRGIVDLTILGDEAKVRLRAAELGVDLDGATVIEPCASELHDQFADQYAQLRKAKGITVEHAREIMNDATYFGTMLVHNCHADGMVSGAAHTTAHTVRPALEIIKTVPGISTVSSIFLMCLPDRVLAYGDCAIIPNPTVEQLADIAICSARTAAQFGIEPRVAMLSYSTGDSGKGADVDKVRAATELVRAREPQLPVEGPIQYDAAVEPSVAATKLRDSPVAGRATVLIFPDLNTGNNTYKAVQRSAGAIAIGPVLQGLRKPVNDLSRGALVDDIVNTVAITAIQAQGVHE

>33|CORE_REP|Org2_Gene4458#

MGPLLPSASLKLNVLPVGVLPRRPVVGRVGRPVFPYEPMVRVSLWLSVTAVAVLFGWGSWQRRWIADDGLIVLRTVRNLLAGNGPVFNQGERVEANTSTAWTYLLYVGGWVGGPMRLEYVALALAMVLSLLGMVLLMLGTGRLYAPSLRGRRAIMLPAGALVYIAVPPARDFATSGLESGLVLAYLGLLWWMMVCWSQPLRARPDSQMFLGALAFVAGCSVLVRPEFALIGGLALIMMLIAARTWRRRVLIVLAGGFLPVAYQIFRMGYYGLLVPSTALAKDAAGDKWSQGMIYVSNFNRPYALWVPLVLSVPLGLLLMTARRRPSFLRPVLAPDYGRVARAVQSPPAVVAFIVGSGVLQALYWIRQGGDFMHGRVLLAPLFCLLAPVGVIPILLPDGKDFSRETGRWLVGALSGLWLGIAGWSLWAANSPGMGGDATRVTYSGIVDERRFYAQATGHAHPLTAADYLDYPRMAAVLTALNNTPEGALLLPSGNYNQWDLVPMIRPSSGTAPGGKPAPKPQHAVFFTNMGMLGMNVGLDVRVIDQIGLVNPLAAHTERLKHARIGHDKNLFPDWVIADGPWVKWYPGIPGYIDQQWVTQAEAALQCPATRAVLNSVRAPITLHRFLSNVLHSYEFTRYRIDRVPRYELVRCGLDVPDGPGPPPRE

>34|CORE_REP|Org20_Gene2239#

MLWILGPHTGPLLFDAVASLDTSPLAAARYHGDQDVAPGVLDFAVNVRHDRPPEWLVRQLAALLPELARYPSTDDVHRAQDAVAERHGRTRDEVLPLVGAAEGFALLHNLSPVRAAIVVPAFTEPAIALSAAGITAHHVVLKPPFVLDTAHVPDDADLVVVGNPTNPTSVLHLREQLLELRRPGRILVVDEAFADWVPGEPQSLADDSLPDVLVLRSLTKTWSLAGLRVGYALGSPDVLARLTVQRAHWPLGTLQLTAIAACCAPRAVAAAAADAVRLTALRAEMVAGLRSVGAEVVDGAAPFVLFNIADADGLRNYLQSKGIAVRRGDTFVGLDARYLRAAVRPEWCWWRRLPMGKAWRTHDAVRLADVIDVLDQAYPPRLAQSWDSVGLVCGDPDDVVDSVTVAVDATPAVVDQVPQAGLLLVHHPLLLRGVDTVAANTPKGVLVHRLIRTGRSLFTAHTNADSASPGVSDALAHAVGLTVDAVLDPVPGAADLDKWVIYVPRENSEAVRAAVFEAGAGHIGDYSHCSWSVAGTGQFLAHDGASPAIGSVGTVERVAEDRVEVVAPARARAEVLAAMRAAHPYEEPAFDIFALVPPPVGSGLGRIGRLPKPEPLRTFVARLEAALPPTATGVRAAGNPDLLVSRVAVCGGAGD

>36|CORE_REP|Org23_Gene3671#

MTAAQQDQAPMATPGCREGETYDVVVLGAGPVGQNVADRARAGGLRVAVVERELVGGECSYWACVPSKALLRPVIAISDARRVDGAREAVDGSINTAGVFGRRNRYVAHWDDTGQADWVSGIGATLIRGDGRLDGPRRVVVTKSSGESVALTARHAVVICTGSRPALPDLPGITEARPWTNRQATDNSTVPDRLAIVGAGGVGVEMATAWQGLGASVTLLARGSGLLPRMEPFVGELIGRGLADAGVDVRVGVSVRALGRPNPTGPVVLELDDGTELRVDEVLFATGRAPRTDDIGLETIGLTPGSWLDVDDTCRVRAVDDGWLYAAGDVNHRALLTHQGKYQARIAGTAIGARAAGRPLDTTSWGMHATTADHHAVPQAFFTDPEAAAVGLTADQAAQAGHRIKAIQQRLPRGGRLPSERELIDRSGLSRVTVRAAVGMLQRQGWLVRRQGLGTFVADPVEQELSCGVRTITEVLLSCGVTPQVDVLSHQTGPAPQRISETLGLVEVLCIRRRIRTGDQPLALVTAYLPPGVGPAVEPLLSGSADTETTYAMWERRLGVRIAQATHEIHAAGASPDVADALGLAVGSPVLVVDRTSYTNDGKPLEVVVFHHRPERYQFSVTLPRTLPGSGAGIIEKRDFA

>38|CORE_REP|Org2_Gene1795#

MSPQLCPKVSIVSTTHNQAGYARQAFDSFLDQQTDFPVEIIVADDASTDATPAIIREYAERYPHVFRPIFRTENLGLNGNLTGALSAARGEYVALCEADDYWIDPLKLSKQVAFLDRHPKTTVCFHPVRVIWEDGHAKDSKFPPVRVRGNLSLDALILMNFIQTNSAVYRRLERYDDIPADVMPLDWYLHVRHAVHGDIAMLPDTMAVYRRHAQGMWHNQVVDPPKFWLTQGPGHAATFDAMLDLFPGDPAREELIAVMADWILRQIANVPGPEGAPRCRKPSRAIPGSPCWRCSTAGDTRAAAQDPVAQARRRDAEPQGARGCVALPAPTRLSSLTMSTNPGPAEGANQVMAQEHSAGAVQFTAHNVRLDDGTLTIPESSRTLDESSWFISARGILETVFPGDKSHLRLADVGCLEGGYAVGFARMGFQVLGIEVRELNMAACNYIKSKTNLPNLRFVHDNALNIANHGLFDTVFCCGLFYHLENPKQYLETLSSVTNKLLILQTHFSIINRSDKWLRLPTTARQLTDRLLRRPAPVKFMLSAPTEHEGLPGRWFTEFSDDRSFGQRDTAKWASWDNRRSFWIQREHLLQAIKDVGVDLVMEEYDNLEPSIAESLLGGSYAANLRGTFIGIKTR

>40|CORE_REP|Org59_Gene3667#

MPEAVQEADLLTAAAVALNRHAALLRELGSVFAAAGHELYLVGGSVRDALLGRLSPDLDFTTDARPERVQEIVRPWADAVWDTGIEFGTVGVGKSDHRMEITTFRADSYDRVSRHPEVRFGDCLEGDLVRRDFTTNAMAVRVTATGPGEFLDPLGGLAALRAKVLDTPAAPSGSFGDDPLRMLRAARFVSQLGFAVAPRVRAAIEEMAPQLARISAERVAAELDKLLVGEDPAAGIDLMVQSGMGAVVLPEIGGMRMAIDEHHQHKDVYQHSLTVLRQAIALEDDGPDLVLRWAALLHDIGKPATRRHEPDGGVSFHHHEVVGAKMVRKRMRALKYSKQMIDDISQLVYLHLRFHGYGDGKWTDSAVRRYVTDAGALLPRLHKLVRADCTTRNKRRAARLQASYDRLEERIAELAAQEXLDRVRPDLDGNQIMAVLDIPAGPQVGEAWRYLKELRLERGPLSTEEATTELLSWWKSRGNXXLGSRVRTVVEYCIAGDDGSAGIWNRPFDVDLDGDGRLDAIGLDLDGDGLRDDALADFDGDDVADHAVFDVDNDGTPESYFIDDGSGTWAVAVDRGGQLRWYGLDGVEHTGGPLVDFDGFGGLDDRLLDTDGDGLADRXLCAGDGSV

>42|CORE_REP|Org59_Gene3058#

MAWSSVRSFEDGIFHMSAPSLGHGGDDGAIDILLVGLDSRTDAHGNPLSAEELATLHAGDEEATNTDTIILIRVPNNGKSATAISXPRDSYVAAPGLGKTKINGVYGQXRETKRAGLVQAXASPTEAAAAGTEAXREALIKTVADLTGVTVDHYAEIGLLGFALIADALGGVDVCLKEPVYEPLSGADFPAGRQKLNGPQALSFVRQRHDLPRGDLDRVVRQQAVMAALAHRVISGQTLSSPATLXRLEQAVQRSVVLSSGWDIMDFVRQLQKLAGGNVAFATIPVLDGAGWSDDGMQSVVRVDPRQVQDWVVGLLHEQDQGKTDELAYTPAKTTANVVNDTDINGLAAAVSKVLSSKGFTTGSVGNNDGDHVPGSQVRAAKADDLGAQQVAKELGGLPVVADASIAPGSVRVVLANDYSGPGSGLGGSDPNGVVSXARAJXPRVRRRHGXPRRRQSLPPAPTRRSASTDHTDHPERGDPGSDAARRPGRPAPITYYDDATGERIELSAVTLANWAAKTGNLLRDELAAGPASRVAILLPAHWQTAAVLFGVWWXGAQAILDDSPADVALCTADRLAEADAVVNSAAVAGEVAVLSLDPLPVDRXPXCRSASPTMRPRCGYTATR

>43|CORE_REP|Org1_Gene1647#

MASRQTPAELARCDLAKTAEREHTPTATATTPSVAGNVMPMSVRSLPAALRACARLQPHDPAFTFMDYEQDWDGVAITLTWSQLYRRTLNVAQELSRCGSTGDRVVISAPQGLEYVVAFLGALQAGRIAVPLSVPQGGVTDERSDSVLSDSSPVAILTTSSAVDDVVQHVARRPGESPPSIIEVDLLDLDAPNGYTFKEDEYPSTAYLQYTSGSTRTPAGVVMSHQNVRVNFEQLMSGYFADTDGIPPPNSALVSWLPFYHDMGLVIGICAPILGGYPAVLTSPVSFLQRPARWMHLMASDFHAFSAAPNFAFELAARRTTDDDMAGRDLGNILTILSGSERVQAATIKRFADRFARFNLQERVIRPSYGLAEATVYVATSKPGQPPETVDFDTESLSAGHAKPCAGGGATSLISYMLPRSPIVRIVDSDTCIECPDGTVGEIWVHGDNVANGYWQKPDESERTFGGKIVTPSPGAPEGPWLRTGDSGFVTDGKMFIIGRIKDLLIVYGRNHSPDDIEATIQEITRGRCAAISVPGDRSTEKLVAIIELKKRGDSDQDAMARLGAIKREVTSALSSSHGLSVADLVLVAPGSIPITTSGKVRRGACVEQYRQDQFARLDA

>45|CORE_REP|Org148_Gene3863#

MTTTIRNGRGDLITAIGGPCDVQALPESQLPELAVQMRRRLIETVTATGGHLGAGLGMVELTIALHRVFTSPHDIVVFDTGHQTYPHKLLTGRGKDFATLRQADGLSGYPNRHESPHDWVENSHASVSLAWVDGIAKALALQGQCDRRVIAVIGDGALTGGVAWEGLNNLGAATRPVIVVLNDNGRSYDPTAGALAAHLEELRVGTPRGPNLFENMGFTYIGPVDGHNIPDTCAVLRKAAAAARPVVVHAVTSKGRGYPPAEADERDHMHACGVVDIATGLASTPSQRSWTDVFEDEIARIADDRSDVVGLTAAMRLPTGLGALSRRYPHRVFDSGIAEQHLLASAAGLAAAGTHPVVAVYSTFLHRAFDQLLFDIGLHRLPVTLVLDRAGVTGPDGPSHHGLWDLALLACVPGFQIACPRDAPRLRQQLRTAIATAAPTAVRFPKGAPGEPITAEHTIGGLDVLHTPPPHWRPDVLLVAVGAMSRPCMDAARCLSEEQIGVTVVDPQWVWPISPALTELAGRHRITVCVEDAIADVGIGAHLSHHIGRTHPRTRTYTLGLPPAYIPHASRDHILSSHGLTGPAIRIRCKSLLNALHEVPGPEDHPDSGDSY

>46|CORE_REP|Org137_Gene2624#

MTDTDLITAGESTDGKPSDAAATDPPDLNADEPAGSLATMVLPELRALANRAGVKGTSGMRKNELIAAIEEIRRQANGAPAVDRSAQEHDKGDRPPSSEAPATQGEQTPTEQIDSQSQQVRPERRSATREAGPSGSGERAGTAADDTDNRQGGQQDAKTEERGTDAGGDQGGDQQASGGQQARGDEDGEARQGRRGRRFRDRRRRGERSGDGAEAELREDDVVQPVAGILDVLDNYAFVRTSGYLPGPHDVYVSMNMVRKNGMRRGDAVTGAVRVPKEGEQPNQRQKFNPLVRLDSINGGSVEDAKKRPEFGKLTPLYPNQRLRLETSTERLTTRVIDLIMPIGKGQRALIVSPPKAGKTTILQDIANAITRNNPECHLMVVLVDERPEEVTDMQRSVKGEVIASTFDRPPSDHTSVAELAIERAKRLVEQGKDVVVLLDSITRLGRAYNNASPASGRILSGGVDSTALYPPKRFLGAARNIEEGGSLTIIATAMVETGSTGDTVIFEEFKGTGNAELKLDRKIAERRVFPAVDVNPSGTRKDELLLSPDEFAIVHKLRRVLSGLDSHQAIDLLMSQLRKTKNNYEFLVQVSKTTPGSMDSD

>50|CORE_REP|Org101_Gene3729#

MAARHHTLSWSIASLHGDEQAVGAPLTTTELTALARTRLFGATGTVLMAIGALGAGARPVVQDPTFGVRLLNLPSRIQTVSLTMTTTGAVMMALAWLMLGRFTLGRRRMSRGKLDRTLLLWMLPLLIAPPMYSKDVYSYLAQSEIGRDGLDPYRVGPASGLGLGHVFTLSVPSLWRETPAPYGPLFLWIGRGISSLTGENIVAAVLCHRLVVLIGVTLIVWATPRLAQRCGVAEVSALWLGAANPLLIMHLVAGIHNEALMLGLMLTGVEFALRGLDMANTPRPSPETWRLGPATIRASRRPELGASPRAGASRAVKPRPEWGPLAMLLAGSILITLSSQVKLPSLLAMGFVTTVLAYRWGGNLRALLLAAAVMASLTLAIMAILGWASGLGFGWINTLGTANVVRSWMSPPTLLALGTGHVGILLGLGDHTTAVLSLTRAIGVLIITVMVCWLLLAVLRGRLHPIGGLGVALAVTVLLFPVVQPWYLLWAIIPLAAWATRPGFRVAAILATLIVGIFGPTANGDRFALFQIVDATAASAIIVILLIALTYTRLPWRPLAAEQVVTAAESASKTPATRRPTAAPDAYADST

>51|CORE_REP|Org73_Gene3981#

MSFVIVAPEALMSVASEVAGIGSALNAANAAAAAPTTGVLAAAADEVSAAMAALFGAHAQEYQRLSAQAAGFHAQFVQALNAGVNSYASAEAANASPLQAVEQQVLGLINGPAQTLLGRPLIGNGADGAPGTGQPGGPGGLLWGNGGNGGSGVAGVGGPGGSGGAAGLFGHGGNGGAGGSNAAGAGGVGGAGGAGWLVGNGGAGGFGGVGTTVSGNGGAGGAAGAFGNGGVGGAGGAAVIGGLPGNGGAGGNAGLIGAGGDGGVGGVGAPGTNGMNPPPNQTSQAANGSPGANNGAGSGGAGLPGNPGAVPGRAGGAGGLGGSGSDTSEGPVTGGNGGNGGDGGPGAPGGNGAPGGIGVNTGTGWAYGGNGGNGGDGGAGARGGDGGNGGNGLALNGGNGIGGNGGAGGRGGTGAAGGNGGIGGGATGTLTFFGSGGDGGPGGAGANTAGTGGVGGVGGAGGQGGLLFGDGGNGGAGGAGGIGGTGASGGAGGKGGSGLVGGDGGNGGAGGAGGNGGKGGAGGAGGGAGMFSQPGVHGAGGTGGQGGAGGAGGAGGAAGAGTVVAGNPGDPGGFGAAGADGLPG

>52|CORE_REP|Org59_Gene823#

MRLILNVIWLVFGGLWLALGYLLASLVCFLLIITIPFGFAALRIASYALWPFGRTIVEKPTAGTGALIGNVIWVLLFGIWLALGHLVSAAAMAGHDHRHSASTGQLETDPGVAGAAXQGHRRGQLTGAHMTLXALGMPALRSRTNGIADPRVVPTTGPLVDTFGRVANDLRVSLTDRCNLRCSYCMPERGLRWLPGEQLLRPDELARLIHIAVTRLGVTSVRFTGGEPLLAHHLDEVVAATARLRPRPEISLTTNGVGLARRAGALAEAGLDRVNVSLDSIDRAHFAAITRRDRLAHVLAGLAAAKAAGLTPVKVNAVLDPTTGREDVVDLLRFCLERGYQLRVIEQXPLDAGHSWRRNIALSADDVLAALRPHFRLRPDPAPRGSAPAELWLVDAGPNTPRGRFGVIASVSHAILXBXXXXXXDRRWPDXLAXLFSTEETDLRRLLRGGADDDAIEAAWRAAMWSKPAGHGINAPDFIQPDRPMSAIGXXPVTQVSDESAGIQVTVRYFAAARAAAGAGSEKVTLRSGATVAELIDGLSVRDVRLATVLSRCSYLRDGIVVRDDAVALSAGDTIDVLPPFAGG

>53|CORE_REP|Org75_Gene2858#

MSAADEQGEERATRKSAPDLRLPGSVAEILASPAGPKVGAFFDLDGTLVAGFTAVILTQERLRRRDMGVGELLGMVQAGLNHTLGRIEFEDLIGKAAAALAGRLLTDLEEIGERLFAQRIESRIYPEMRELVRAHVARGHTVVLSSSALTIQVGPVARFLGINNMLTNKFETNEDGILTGGVLKPILWCPGKATAVQRFAAEHDIDLKDSYFYADGDEDVALMYLVGNPRPTNPEGKMAAVAKRRGWPILKFNSRGGVGIRRQLRTLAGLSTIVPVAAGAVGIGVLTGSRRRGVNFFTSTFSQLLLATSGVHLNVIGKENLTAQRPAVFIFNHRNQVDPVIAGALVRDNWVGVGKKELASDPIMGTLGKLLDGVFIDRDDPVAAVETLHTVEERARNGLSIVIAPEGTRLDTTEVGSFKKGPFRIAMAAKIPIVPIVIRNAEIVASRNSTTINPGTVDVAVFPPIPVDDWTLDALPDRIAEVRQLYLDTLADWPVDGLPAVDLYAEQKAARKARAQVAKATAKRVPAKKAPAKSAANKGAAATKAATKKASPKAKPSESKIAGKDGEASASPSSSAKGRS

>55|CORE_REP|Org24_Gene2374#

MALYRKYRPASFAEVVGQEHVTAPLSVALDAGRINHAYLFSGPRGCGKTSSARILARSLNCAQGPTANPCGVCESCVSLAPNAPGSIDVVELDAASHGGVDDTRELRDRAFYAPVQSRYRVFIVDEAHMVTTAGFNALLKIVEEPPEHLIFIFATTEPEKVLPTIRSRTHHYPFRLLPPRTMRALLARICEQEGVVVDDAVYPLVIRAGGGSPRDTLSVLDQLLAGAADTHVTYTRALGLLGVTDVALIDDAVDALAACDAAALFGAIESVIDGGHDPRRFATDLLERFRDLIVLQSVPDAASRGVVDAPEDALDRMREQAARIGRATLTRYAEVVQAGLGEMRGATAPRLLLEVVCARLLLPSASDAESALLQRVERIETRLDMSIPAPQAVPRPSAAAAEPKHQPAREPRPVLAPTPASSEPTVAAVRSMWPTVRDKVRLRSRTTEVMLAGATVRALEDNTLVLTHESAPLARRLSEQRNADVLAEALKDALGVNWRVRCETGEPAAAASPVGGGANVATAKAVNPAPTANSTQRDEEEHMLAEAGRGDPSPRRDPEEVALELLQNELGARRIDNA

>56|CORE_REP|Org88_Gene3088#

MTTGGLVDENDGAAMRPLRHTLSQLRLHELLVEVQDRVEQIVEGRDRLDGLVEAMLVVTAGLDLEATLRAIVHSATSLVDARYGAMEVHDRQHRVLHFVYEGIDEETVRRIGHLPKGLGVIGLLIEDPKPLRLDDVSAHPASIGFPPYHPPMRTFLGVPVRVRDESFGTLYLTDKTNGQPFSDDDEVLVQALAAAAGIAVANARLYQQAKARQSWIEATRDIATELLSGTEPATVFRLVAAEALKLTAADAALVAVPVDEDMPAADVGELLVIETVGSAVASIVGRTIPVAGAVLREVFVNGIPRRVDRVDLEGLDELADAGPALLLPLRARGTVAGVVVVLSQGGPGAFTDEQLEMMAAFADQAALAWQLATSQRRMRELDVLTDRDRIARDLHDHVIQRLFAIGLALQGAVPHERNPEVQQRLSDVVDDLQDVIQEIRTTIYDLHGASQGITRLRQRIDAAVAQFADSGLRTSVQFVGPLSVVDSALADQAEAVVREAVSNAVRHAKASTLTVRVKVDDDLCIEVTDNGRGLPDEFTGSGLTNLRQRAEQAGGEFTLASVPGASGTVLRWSAPLSQ

>57|CORE_REP|Org59_Gene611#

MKLTDVDFAVEASGMVRAFNQAGVLDVSDVHVAQRLCALAGESDERVALAVAVAVRALRAGSVCVDLLSIARVAGHDDLPWPDPADWLAAVRASPLLADPPVLHLYDDRLLYLDRYWREEEQVCADLLALLTSRRPAGVPDLRRLFPTGFDEQRRAAEIALSQGVTVLTGGPGTGKTTTVARLLALVAEQAELAGEPRPRIALAAPTGKAAARLAEAVRREMAKLDATDRARLGDLHAVTLHRLLGAKPGAXXRLXXRQNRLPHNVIVVDETSMVSLTLMARLAEAVRPGARLILVGDADQLASVEAGAVLADLVDGFSVRDDALVAQLRTSHRFGKVIGTLAEAIRAGDGDAVLGLLRSGEERIEFVDDEDPAPRLRAVLVPHALRLREAALLGASDVALATLDEHRLLCAHRDGPTGVLHWNRRVQAWLAEETGQPPWTPWYAGRPLLVTANDYGLRVYNGDTGVVLAGPTGLRAVISGASGPLDVATGRLGDVETMHAMTIHKSQGSQVDEVTVLMPQEDSRLLTRELLYTAVTRAKRKVRVVGSEASVRAAIARRAVRASGLRMRLQSTGCG

>58|CORE_REP|Org70_Gene4023#

MPAKKTMAQRLGQALETMTRQCGQLPETPAYGSWLLGRVSESPSRRWVRIKRIVTVYIMTANLTGIVVALLVVTFAFPVPSIYTDAPWWVTFGVAPAYATLALAIGTYWITTRIVRASIRWAIEERAPSQADGRNTLLLPFRVAAVHLILWDIGGALLATLYGLANRVFVTIILFSVTICGVLVATNCYLFTEFALRPVAAKALEAGRPPRRFAPGIMGRTMTVWSLGSGVPVTGIATTALYVLLVHNLTETQLASAVLILSITTLIFGFLVMWILAWLTAAPVRVVRAALKRVEQGDLRGDLVVFDGTELGELQRGFNAMVNGLRERERVRDLFGRHVGREVAAAAERERPKLGGEERHVAVVFVDIVGSTQLVTSRPAAEVVMLLNRFFTVIVDEVNHHRGLVNKFQGDASLAVFGAPNRLSHPEDAALATARAIADRLASEMPECQAGIGVAAGQVVAGNVGAHERFEYTVIGEPVNEAARLCELAKSYPSRLLASSQTLRGASENECARWSLGETVTLRGHDQPIRLASPVQQLQMPAQSADIVGGALGDHQTHTIYRGAHPTD

>61|CORE_REP|Org120_Gene2512#

MALTCTDMSDAVAGSDAEGLTADAIVVGAGLAGLVAACELADRGLRVLILDQENRANVGGQAFWSFGGLFLVNSPEQRRLGIRDSHELALQDWLGTAAFDRPEDYWPEQWAHAYVDFAAGEKRSWLRARGLKIFPLVGWAERGGYDAQGHGNSVPRFHITWGTGPALVDIFVRQLRDRPTVRFAHRHQVDKLIVEGNAVTGVRGTVLEPSDEPRGAPSSRKSVGKFEFRASAVIVASGGIGGNHELVRKNWPRRMGRIPKQLLSGVPAHVDGRMIGIAQKAGAAVINPDRMWHYTEGITNYDPIWPRHGIRIIPGPSSLWLDAAGKRLPVPLFPGFDTLGTLEYITKSGHDYTWFVLNAKIIEKEFALSGQEQNPDLTGRRLGQLLRSRAHAGPPGPVQAFIDRGVDFVHANSLRELVAAMNELPDVVPLDYETVAAAVTARDREVVNKYSKDGQITAIRAARRYRGDRFGRVVAPHRLTDPKAGPLIAVKLHILTRKTLGGIETDLDARVLKADGTPLAGLYAAGEVAGFGGGGVHGYRALEGTFLGGCIFSGRAAGRGAAEDIR

>64|CORE_REP|Org2_Gene4222#

MPVVKINAIEVPAGAGPELEKRFAHRAHAVENSPGFLGFQLLRPVKGEERYFVVTHWESDEAFQAWANGPAIAAHAGHRANPWRPVLRCWNSRSCLTSVGPARLHNRRAGRRMLALSAAAALIVALASGCSSAPTPSANAANHGHRIDTRTPPGLRAQQTMDMLNSDWPIGEIGVGTLAAPGQVDTVKTTMEALWWDRPFALAGVDIGASVAALHLISSYGAQQDIRIHTDDDGWVDRFDVETQAPSIASWRDVDAVLSKTGARYSFQVAKVDNGRCDPVAGTNTGESLPLASIFKLYVLHALAGAVQHNTVSWDDLLTVTAKSKAVGSSGLELPVGARVSVRTAAEKMIATSDNMATDLLIERLGTRAIEEALASAGHHDPASMTPFPTMYELFSVGWGKPDLRDQWKHATQQVRAQILRQTNSTPYQPDPTRAHTPASNYGAEWYGSAEDICRVHAALRADAVGPASPVRQIMSAVPGIQLDRSVWPYIGAKAGGLPGDLTFSWYAVDKTGQPWVVSFQLNWPRDHGPTVTGWMLQVARQVFALIAPQ

>67|CORE_REP|Org23_Gene3965#

MSFVSVAPEIVVAAATDLAGIGSAISAANAAAAAPTTAVLAAGADEVSAAIAALFSGHAQAYQALSAQAAAFHQQFVQTLAGGAGAYAAAEAQVEQQLLAAINAPTQALLGRPLIGNGADGAPGTGQAGGAGGILYGNGGNGGSGAAGQAGGAGGPAGLIGHGGSGGAGGSGAAGGAGGHGGWLWGNGGVGGSGGAGVGAGVAGGHGGAGGAAGLWGAGGGGGNGGNGADANIVSGGDGGLGGAGGGGGWLYGDGGAGGHGGQGAGGGAGGAGGDGGQGGAGRGLWGTGGAGGHGGQGGGTGGPPLPGQAGMGAAGGAGGLIGNGGAGGDGGVGASGGVAGVXGAGGNAMLIGHGGAGGAGGDSSFANGAAGGAGGAGGHLFGNGGSGGHGGAVTAGNTGIGGAGGVGGDARLIGHGGAGGAGGDRAGALVGRDGGPGGNGGAGGQLYGNGGDGGPGGQGGQAFGANNIGGTGGAGGNGGPAILSGNGGNGGAGGAGGAGGAGGGAGGVGGAGGAPGTGGTLQAAVSGLVTALFGAPGQPGDTGQPG

>70|CORE_REP|Org20_Gene3484#

MDFGALPPEINSARMYAGPGSASLVAAAKMWDSVASDLFSAASAFQSVVWGLTVGSWIGSSAGLMAAAASPYVAWMSVTAGQAQLTAAQVRVAAAAYETAYRLTVPPPVIAENRTELMTLTATNLLGQNTPAIEANQAAYSQMWGQDAEAMYGYAATAATATEALLPFEDAPLITNPGGLLEQAVAVEEAIDTAAANQLMNNVPQALQQLAQPAQGVVPSSKLGGLWTAVSPHLSPLSNVSSIANNHMSMMGTGVSMTNTLHSMLKGLAPAAAQAVETAAENGVWAMSSLGSQLGSSLGSSGLGAGVAANLGRAASVGSLSVPPAWAAANQAVTPAARALPLTSLTSAAQTAPGHMLGGLPLGHPRRQRYPAHWRRHGPTRYPAHRPPDSTTGLRGCVGVVPRRGWRALAIWSKGPDPTGRTPRHRGAVDGIRKAVTGNGIDAGTTIRDDHDGRRQPRDLDGRCYAGDQPARAGLTMAQAGGNLPDQQPTHQRVSRFPAALRSTNRPPRHGRRRRHLVGNKCGRHQRGFACVIPSHRV

>71|CORE_REP|Org23_Gene3889#

MILPAPHVEYFLLAPMLIVFSVAVAGVLAEAFLPRRWRYGAQVTLALGGSAVALIAVIVVARSIHGSGHXAVLGAIAVDRATLFLQGTVLLVTIMAVVFMAERSARVSPQRQNTLAVARLPGLDSFTPQLSAVPGSDAERQAERAGATQTELFPLAMLSVGGMMVFPASNDLLTMFVALEVLSLPLYLMCGLARNRRLLSQEAAMKYFLLGAFSSAFFLYGVALLYGATGTLTLPGIRDALAARTDDSMALAGVALLAVGLLFKVGAVPFHSWIPDVYQGAPTPITGFMAAATKVAAFGALLRVVYVALPPLHDQWRPVLWAIAILTMTVGTVTAVNQTNVKRMLAYSSVAHVGFILTGVIADNPAGLSATLFYLVAYSFSTMGAFAIVGLVRGADGSAGSEDADLSHWAGLGQRSPIVGVMLSMFLLAFAGIPLTSGFVSKFAVFRAAASAGAVPLVIVGVISSGVAAYFYVRVIVSMFFTEESGDTPHVAAPGVLSKAAIAVCTVVTVVLGIAPQPVLDLADQAAQLLR

>73|CORE_REP|Org87_Gene1778#

MAATKASTATDEPVKRTATKSPAASASGAKTGAKRTAAKSASGSPPAKRATKPAARSVKPASAPQDTTTSTIPKRKTRAAAKSAAAKAPSARGHATKPRAPKDAQHEAATDPEDALDSVEELDAEPDLDVEPGEDLDLDAADLNLDDLEDDVAPDADDDLDSGDDEDHEDLEAEAAVAPGQTADDDEEIAEPTEKDKASGDFVWDEDESEALRQARKDAELTASADSVRAYLKQIGKVALLNAEEEVELAKRIEAGLYATQLMTELSERGEKLPAAQRRDMMWICRDGDRAKNHLLEANLRLVVSLAKRYTGRGMAFLDLIQEGNLGLIRAVEKFDYTKGYKFSTYATWWIRQAITRAMADQARTIRIPVHMVEVINKLGRIQRELLQDLGREPTPEELAKEMDITPEKVLEIQQYAREPISLDQTIGDEGDSQLGDFIEDSEAVVAVDAVSFTLLQDQLQSVLDTLSEREAGVVRLRFGLTDGQPRTLDEIGQVYGVTRERIRQIESKTMSKLRHPSRSQVLRDYLD

>75|CORE_REP|Org9_Gene1083#

MHADLAATTSREDFRLLAAEHRVVPVTRKVLADSETPLSAYRKLAANRPGTFLLESAENGRSWSRWSFIGAGAPTALTVREGQAVWLGAVPKDAPTGGDPLRALQVTLELLATADRQSEPGLPPLSGGMVGFFAYDMVRRLERLPERAVDDLCLPDMLLLLATDVAAVDHHEGTITLIANAVNWNGTDERVDWAYDDAVARLDVMTAALGQPLPSTVATFSRPEPRHRAQRTVEEYGAIVEYLVDQIAAGEAFQVVPSQRFEMDTDVDPIDVYRILRVTNPSPYMYLLQVPNSDGAVDFSIVGSSPEALVTVHEGWATTHPIAGTRWRGRTDDEDVLLEKELLADDKERAEHLMLVDLGRNDLGRVCTPGTVRVEDYSHIERYSHVMHLVSTVTGKLGEGRTALDAVTACFPAGTLSGAPKVRAMELIEEVEKTRRGLYGGVVGYLDFAGNADFAIAIRTALMRNGTAYVQAGGGVVADSNGSYEYNEARNKARAVLNAIAAAETLAAPGANRSGC

>76|CORE_REP|Org90_Gene2789#

MTTPSHAPAVDLATAKDAVVQHLSRLFEFTTGPQGGPARLGFAGAVLITAGGLGAGSVRQHDPLLESIHMSWLRFGHGLVLSSILLWTGVGVMLLAWLGLGRRVLAGEATEFTMRATTVIWLAPLLLSVPVFSRDTYSYLAQGALLRDGLDPYAVGPVGNPNALLDDVSPIWTITTAPYGPAFILVAKFVTVIVGNNVVAGTMLLRLCMLPGLALLVWATPRLASHLGTHGPTALWICVLNPLVLIHLMGGVHNEMLMVGLMTAGIALTVQGRNVAGIILITVAIAVKATAGIALPFLVWVWLRHLRERRGYRPVQAFLAAAAISLLIFVAVFAVLSAVAGVGLGWLTALAGSVKIINWLTVPTGAANVIHALGRGLFTVDFYTLLRITRLIGIVIIAVSLPLLWWRFRRDDRAALTGVAWSMLIVVLFVPAALPWYYSWPLAVAAPLAQARRAIAAIAGLSTWVMVIFKPDGSHGMYSWLHFWIATACALTAWYVLYRSPDRRGVQAATPVVNTP

>80|CORE_REP|Org2_Gene1185#

MTFPGDTAVLVLAAGPGTRMRSDTPKVLHTLAGRSMLSHVLHAIAKLAPQRLIVVLGHDHQRIAPLVGELADTLGRTIDVALQDRPLGTGHAVLCGLSALPDDYAGNVVVTSGDTPLLDADTLADLIATHRAVSAAVTVLTTTLDDPFGYGRILRTQDHEVMAIVEQTDATPSQREIREVNAGVYAFDIAALRSALSRLSSNNAQQELYLTDVIAILRSDGQTVHASHVDDSALVAGVNNRVQLAELASELNRRVVAAHQLAGVTVVDPATTWIDVDVTIGRDTVIHPGTQLLGRTQIGGRCVVGPDTTLTDVAVGDGASVVRTHGSSSSIGDGAAVGPFTYLRPGTALGADGKLGAFVEVKNSTIGTGTKVPHLTYVGDADIGEYSNIGASSVFVNYDGTSKRRTTVGSHVRTGSDTMFVAPVTIGDGAYTGAGTVVREDVPPGRWQCRRVRNATSRTGCSANAPAAQRLRPQKEPQKWPANSPHNHPTLIRHREVASRLPGDKSGRCATYHG

>82|CORE_REP|Org67_Gene4027#

MRIGPVELSAVKDWDPAPGVLVSWHPTPASCAKAFAAPVSAVPPSYVQARQIRSFSEQAARGLDHSRLLIASVEVFGHCDLRAMTYVINAHVRRHDTYRSWFELRDTDHIVRHSIADPADIEFVPTTHGEMTSADLRQHIVATPDSLHWDCFSFGVIQRADSFTFYASIDHLHADGQFVGVGLMEFQSMYTALIMGEPPIGLSEAGSYVDFCVRQHEYTSALTVDSPEVRAWIDFAEINNGTFPEFPLPLGDPSVRCGGDLLSMMLMDEQQTQRFESACMAANARFIGGMLACIAIAIHELTGADTYFGITPKDIRTPADLMTQGWFTGQIPVTVPVAGLSFNEIARIAQTSFDTGADLAKVPFERVVELSPSLRRPQPLFSLVNFFDAQVGPLSAVTKLFEGLNVGTYSDGRVTYPLSTMVGRFDETAASVLFPDNPVARESVTAYLRAIRSVCMRIANGGTAERVGNVVALSPGRRNNIERMTWRSCRAGDFIDICNLKVANVTVDREA

>83|CORE_REP|Org1_Gene1761#

MADAARARRLAKRIAAIVASAIEYEIKDPGLAGVTITDAKVTADLHDATVYYTVMGRTLHDEPNCAGAAAALERAKGVLRTKVGAGTGVRFTPTLTFTLDTISDSVHRMDELLARARAADADLARVRVGAKPAGEADPYRDNGSVAQSPAPGGLGIRTSDGPEAVEAPLTCGETPVTTIDPRNELVDGRRRAGARVDAVGAAALLSAAARVGVVCHVHPDADTIGAGLALALVLDGCGKRVEVSFAAPATLPESLRSLPGCHLLVRPEVMRRDVDLVVTVDIPSVDRLGALGDLTDSGRELLVIDHHASNDLFGTANFIDPSADSTTTMVAEILDAWGKPIDPRVAHCIYAGLATDTGSFRWASVRGYRLAARLVEIGVDNATVSRTLMDSHPFTWLPLLSRVLGSAQLVSEAVGGRGLVYVVVDNREWVAARSEEVESIVDIVRTTQQAEVAAVFKEVEPHRWSVSMRAKTVNLAAVASGFGGGGHRLAAGYTTTGSIDDAVASLRAALG

>85|CORE_REP|Org118_Gene3348#

MPDKRTALDDAVAQLRSGMTIGIAGWGSRRKPMAFVRAILRSDVTDLTVVTYGGPDLGLLCSAGKVKRVYYGFVSLDSPPFYDPWFAHARTSGAIEAREMDEGMLRCGLQAAAQRLPFLPIRAGLGSSVPQFWAGELQTVTSPYPAPGGGYETLIAMPALRLDAAFAHLNLGDSHGNAAYTGIDPYFDDLFLMAAEAALSVGGAHRRHRGTGQIGAAAGAVGQPDDGRRHRGSTRRRPLHHRRTGLRARRAVPAALRRSGVDTGGLAAVRAHLPIRHRSGLPGRGAQLWSITVSTRAEVCAVACAELFRDAGEIMISPMTNMASVGARLARLTFAPDILLTDGEAQLLADTPALGKTGAPNRIEGWMPFGRVFETLAWGRRHVVMGANQVDRYGNQNISAFGPLQRPTRQMFGVRGSPGNTINHATSYWVGNHCKRVFVEAVDVVSGIGYDKVDPDNPAFRFVNVYRVVSNLGVFDFGGPDHSMRAVSLHPGVTPGDVRDATSFRGA

>86|CORE_REP|Org118_Gene1678#

MAEESRGQRGSGYGLGLSTRTQVTGYQFLARRTAMALTRWRVRMEIEPGRRQTLAVVASVSAALVICLGALLWSFISPSGQLNESPIIADRDSGALYVRVGDRLYPALNLASARLITGRPDNPHLVRSSQIATMPRGPLVGIPGAPSSFSPKSPPASSWLVLGDTVATSSSIGSLQGVTVTVIDGTPDLTGHRQILSGSDAVVLRYGGDAWVIREGRRSRIEPTNRAVLLPLGLTPEQVSQARPMSRALFDALPVGPELLVPEVPNAGGPATFPGAPGPIGTVIVTPQISGPQQYSLVLGDGVQTLPPLVAQILQNAGSAGNTKPLTVEPSTLAKMPVVNRLDLSAYPDNPLEVVDIREHPSTCWWWERTAGENRARVRVVSGPTIPVAATEMNKVVSLVKADTSGRQADQVYFGPDHANFVAVTGNNPGAQTSESLWWVTDAGARFGVEDSKEARDALGLTLTPSLAPWVALRLLPQGPTLSRADALVEHDTLPMDMTPAELVVPK

>87|CORE_REP|Org1_Gene1027#

MKPAQGPDFEGSQGAVKRTPRVITRPSAQRLTLRAAAHGARDPTPGRRVIPIATTFSYKHLVGQPYQPKREGVLVADTDDTATLRYPGGEIDLQIVHATEGADGIALGPLLAKTGHTTFDVGFANTAAAKSSITYIDGDAGILRYRGYPIDQLAEKSTFIEVCYLLIYGELPDTDQLAQFTGRIQRHTMLHEDLKRFFDGFPRNAHPMPVLSSVVNALSAYYQDALDPMDNGQVELSTIRLLAKLPTIAAYAYKKSVGQPFLYPDNSLTLVENFLRLTFGFPAEPYQADPEVVRALDMLFILHADHEQNCSTSTVRLVGSSRANLFTSISGGINALWGPLHGGANQAVLEMLEGIRDSGDDVSEFVRKVKNREAGVKLMGFGHRVYKNYDPRARIVKEQADKILAKLGGDDSLLGIAKELEEAALTDDYFIERKLYPNVDFYTGLIYRALGFPTRMFTVLFALGRLPGWIAHWREMHDEGDSKIGRPRQIYTGYTERDYVTIDAR

>89|CORE_REP|Org29_Gene3386#

MSHLVTAPDMLATAAAHVDEIASTLRAANAAAAGPTCNLLAAAGDEVSAATAALFSAYGREYQAVVKQAAAFHSEFTRTLEAAGNAYAHAEAANAARVSHALDTINAPIRTLLGRAPLSPNGSSGAGGLPAIAQLAAESPITALIMGGTNNPLPDPEYVTDINKAFIQTLFPGAVSQGLFTPEQFWPVTPDLGNLTFNQSVTEGVALLNTAVNNQLALDNKVVAFGYSQSATIINNYINSLMAMGSPNPDDISFVMIGSGNNPVGGLLARFPGFYIPFLDVPFNGATPANSPYPTHIYTAQYDGIAHAPQFPLRILSDINAFMGYFYVHNTYPELMATQVDNAVPLPTSPGYTGNTQYYMFLTQDLPLLQPIRDIPYAGPPIADLFQPQLRVLVDLGYADYGPGGNYADIPTPAGLFSIPNPFAVTYYLIKGSLQAPYGAIVEIGVEAGLIGPEWFPDSYPWVPSINPGLNFYFGQPQVTLLSLMSGGLGNILHLIPPPVFT

>92|CORE_REP|Org1_Gene2915#

MSFVVTIPEALAAVATDLAGIGSTIGTANAAAAVPTTTVLAAAADEVSAAMAALFSGHAQAYQALSAQAALFHEQFVRALTAGAGSYAAAEAASAAPLEGVLDVINAPALALLGRPLIGNGANGAPGTGANGGDGGILIGNGGAGGSGAAGMPGGNGGAAGLFGNGGPAAPGERSVRHRRVRRGRRAGGLLYGAGGAGGAGGRAGGGVGGIGGAGGAGGNGGLLFGAGGPAASADSRPTPVTAGRRRRRVVLRRGRCRRAGGTGTNVTGGAGGAGGNGGLLFGAGGVGGVGGDGVAFLGTAPAGPVVPAGPVGCSASVGPAAPAESDWSGTAVPGVRRVRPALGRRRCRRRGWVGSTTGGAGGAGGNAGLLVGAGGAGGAGALGGGATGVGGAGGNGGTAGLLFGAGGAGGAGGFGFGGAGGAGGLGGKAGLIGDGGDGGAGGNGTGAKGGDGGAGGGAILVGNGGNGGNAGSGTPNGSAGTGGAGGLLGKNGMNGLP

>93|CORE_REP|Org85_Gene4018#

MSQTARRLGPQDMFFLYSESSTTMMHVGALMPFTPPSGAPPDLLRQLVDESKASEVVEPWSLRLSHPELLYHPTQSWVVDDNFDLDYHVRRSALASPGDERELGIPVSRLHSHALDLRRPPWEVHFIEGLEGGRFAIYIKMHHSLIDGYTGQKMLARSLSTDPHDTTHPLFFNIPTPGRSPADTQDSVGGGLIAGAGNVLDGLGDVVRGLGGLVSGVGSVLGSVAGAGRSTFELTKALVNAQLRSDHEYRNLVGSVQAPHCILNTRISRNRRFATQQYPLDRLKAIGAQYDATINDVALAIIGGGLRRFLDELGELPNKSLIVVLPVNVRPKDDEGGGNAVATILATLGTDVADPVQRLAAVTASTRAAKAQLRSMDKDAILAYSAALMAPYGVQLASTLSGVKPPWPYTFNLCVSNVPGPEDVLYXRGSRMEASYPVSLVAHSQALNVTLQSYAGTLNFGFIGCRDTLPHLQRLAVYTGEALDQLAAADGAAGLGS

>94|CORE_REP|Org59_Gene728#

MARHLRGRLPLRVRLVAATLILVATGLVASGIAVTSMLQHRLTSRIDRVLLEEAQIWAQITLPLAPDPYPGHNPDRPPSRFYVRVISPDGQSYTALNDNTAIPAVPANNDVGRHPTTLPSIGGSKTLWRAVSVRASDGYLTTVAIDLADVRSTVRSLVLLQVGIGSAVLVVLGVAGYAVVRRSLRPLAEFEQTAAAIGAGQLDRRVPQWHPRTEVGRLSLALNGMLAQIQRAVASAESSAEKARDSEDRMRQFITDASHELRTPLTTIRGFAELYRQGAARDVGMLLSRIESEASRMGLLVDDLLLLARLDAXRPLELCRVDLLALASDAAHDARAMDPKRRITLEVLDGPGTPEVLGDESRLRQVLRNLVANAIQHTPESADVTVRVGTEGDDAILEVADDGPGMSQEDALRVFERFYRADSSRARASGGTGLGLSIVDSLVAAHGGAVTVTTALGEGCCFRVSLPRVSDRGPAEPHASCARAALILACAIVQRSR

>98|CORE_REP|Org54_Gene2940#

MVTRIVILGGGPAGYEAALVAATSHPETTQVTVIDCDGIGGAAVLDDCVPSKTFIASTGLRTELRRAPHLGFHIDFDDAKISLPQIHARVKTLAAAQSADITAQLLSMGVQVIAGRGELIDSTPGLARHRIKATAADGSTSEHEADVVLVATGASPRILPSAQPDGERILTWRQLYDLDALPDHLIVVGSGVTGAEFVDAYTELGVPVTVVASQDHVLPYEDADAALVLEESFAERGVRLFKNARAASVTRTGAGVLVTMTDGRTVEGSHALMTIGSVPNTSGLGLERVGIQLGRGNYLTVDRVSRTLATGIYAAGDCTGLLPLASVAAMQGRIAMYHALGEGVSPIRLRTVAATVFTRPEIAAVGVPQSVIDAGSVAARTIMLPLRTNARAKMSEMRHGFVKIFCRRSTGVVIGGVVVAPIASELILPIAVAVQNRITVNELAQTLAVYPSLSGSITEAARRLMAHDDLDCTAAQDAAEQLALVPHHLPTSN

>102|CORE_REP|Org85_Gene2669#

MSAASQRVGAFGEEAGYHKGLKPRQLQMIGIGGAIGTGLFLGAGGRLAKAGPGLFLVYGVCGVFVFLILRALGELVLHRPSSGSFVSYAREFFGEKAAYAVGWMYFLHWAMTSIVDTTAIATYLQRWTIFTVVPQWILALIALTVVLSMNLISVEWFGELEFWAALIKVLALMAFLVVGTVFLAGRYPVDGHSTGLSLWNNHGGLFPTSWLPLLIVTSGVVFAYSAVELVGTAAGETAEPEKIMPRAINSVVARIAIFYVGSVALLALLLPYTAYKAGESPFVTFFSKIGFHGAGDLMNIVVLTAALSSLNAGLYSTGRVMHSIAMSGSAPRFTARMSKSGVPYGGIVLTAVITLFGVALNAFKPGEAFEIVLNMSALGIIAGWATIVLCQLRLHKLANAGIMQRPRFRMPFSPYSGYLTLLFLLVVLVTMASDKPIGTWTVATLIIVIPALTAGWYLVRKRVMAVARERLGHTGPFPAVANPPVRSRD

>103|CORE_REP|Org103_Gene469#

MRAEIGPDFRPHYTFGDAYPASERAHVNWELSAPVWHTAQMGSTTHREVAKLDRVPLPVEAARVAATGWQVTRTAVRFIGRLPRKGPWQQKVIKELPQTFADLGPTYVKFGQIIASSPGAFGESLSREFRGLLDRVPPAKTDEVHKLFVEELGDEPARLFASFEEEPFASASIAQVHYATLRSGEEVVVKIQRPGIRRRVAADLQILKRFAQTVELAKLGRRLSAQDVVADFADNLAEELDFRLEAQSMEAWVSHLHASPLGKNIRVPQVHWDFTTERVLTMERVHGIRIDNAAAIRKAGFDGVELVKALLFSVFEGGLRHGLFHGDLHAGNLYVDEAGRIVFFDFGIMGRIDPRTRWLLRELVYALLVKKDHAAAGKIVVLMGAVGTMKPETQAAKDLERFATPLTMQSLGDMSYADIGRQLSALADAYDVKLPRELVLIGKQFLYVERYMKLLAPRWQMMSDPQLTGYFANFMVEVSREHQSDIEV

>105|CORE_REP|Org62_Gene3304#

MSETFCLTDHSEPMTARFLSVVLRRIRGMRSDTREEISAALDAYHASLSRVLDLKCDALTTPELLACLQRLEVERRRQGAAEHALINQLAGQACEEELGGTLRTALANRLHITPGEASRRIAEAEDLGERRALTGEPLPAQLTATAAAQREGKIGREHIKEIQAFFKELSAAVDLGIREAAEAQLAELATSRRPDHLHGLATQLMDWLHPDGNFSDQERARKRGITMGKQEFDGMSRISGLLTPELRATIEAVLAKLAAPGACNPDDQTPLVDDTPDADAVRRDTRSQAQRNHDAFLAALRGLLASGELGQHKGLPVTIVVSTTLKELEAATGKGVTGGGSRVPMSDLIRMASHANHYLALFDGAKPLALYHTKRLASPAQRIMLYAKDRGCSRPGCDAPAYHSEVHHVTPWTTTHRTDINDLTLACGPDNRLVEKGWKTRKNAHGDTEWLPPPHLDHGQPRINRYHHPAKILCEQDDDEPH

>106|CORE_REP|Org59_Gene468#

MAGVRHDDGSGLIAQRRPVRGEGATRSRGPSGPSNRNVSAADDPRRVALLAVHTSPLAQPGTGDAGGMNVYMLQSALHLARRGIEVEIFTRATAXXRSTGGAGGTRGAGAZRGGGXXRGXGQVRPAHPALSRXAAGVLRAEAVHEPGYYDIVHSHYWLSGQVGWLARDRWAVPLVHTAHTLAAVKNAALADGDGPEPPLRTVGEQQVVDEADRLIVNTDDEARQVISLHGADPARIDVVHPGVDLDXFRPGDRRAARAALGLPVDERVVAFVGRIQPLKAPDIVLRAAAKLPGVRIIVAGGPSGSGLASPDGLVRLADELGISARVTFLPPQSHTDLATLFRAADLVAVPSYSESFGLVAVEAQACGTPVVAAAVGGLPVAVRDGITGTLVSGHEVGQWADAIDHLLRLCAGPRGRVMSRAAARHAATFSWENTTDALLASYRRAIGEYNAERQRRGGEVISDLVAVGKPRHWTPRRGVGA

>107|CORE_REP|Org13_Gene2210#

MDVTVVGSGPNGLATAVICARAGLNVQVVEAQATFGGGARSAADFEFPEVLHDVCSAVHPLALASPFFAEFDLPARGVTLTVPDIAYANPLPGRPAAIAYHDLAHTCAKLDDGASWRRLLGPLVAHSETVVEFMLSDKRSLPTALGSVLRLGLRMLAQGTPAWRSLAGEDARALFTGVAAHAISPLPSLVSAGAGLMLATLAHSVGWPIPVGGTQAIADALIADLRAHGGRLAAGVEITEPQRSVVVFDTAPTALLRVYRDKLPHRYAKALRRYRFRAGIAKVDFVLSDEIPWSDPRLRRAATLHLGGTRDQMARAEADVAAGRHADWPMVLAACPHVADPGRIDETGRRPFWTYAHVPSGSTLDATETVTSVLERFAPGFRDIVVAARAVPAARMADHNANYVGGDITVGANSTWRAIAGPTPRLNPWRTPIPKVYLCSAATPPGAGVHGMCGWYAARTLLRTEFGITRMPPLGHELRP

>109|CORE_REP|Org1_Gene3828#

MISAMTDADSAVPPRLDEDAISKLELTEVADLIRTRQLTSAEVTESTLRRIERLDPQLKSYAFVMPETALAAARAADADIACGHYEGVLHGVPIGVKDLCYTVDAPTAAGTTIFRDFRPAYDATVVARLRAAGAVIIGKLAMTEGAYLGYHPSLPTPVNPWDPTAWAGVSSSGCGVATAAGLCFGSIGSDTGGSIRFPTSMCGVTGIKPTWGRVSRHGVVELAASYDHVGPITRSAHDAAVLLSVIAGSDIHDPSCSAEPVPDYAADLALTRIPRVGVDWSQTTSFDEDTTAMLADVVKTLDDIGWPVIDVKLPALAPMVAAFGKMRAVETAIAHADTYPARADEYGPIMRAMIDAGHRLAAVEYQTLTERRLEFTRSLRRVFHDVDILLMPSAGIASPTLETMRGLGQDPELTARLAMPTAPFNVSGNPAICLPAGTTARGTPLGVQFIGREFDEHLLVRAGHAFQQVTGYHRRRPPV

>110|CORE_REP|Org67_Gene510#

MTAAVRHSDVLVVGAGSAGSVVAERLSMDSSCVVTVLEAGPGLADPGLLAQTANGLQLPIGAGSPLVERYRTRLTDRPVRHLPIVRGATVGGSGAINGGYFCRGLPSDFDRASIPGWAWSDVLEHFRAIETDLDFETPVHGRSGPIPVRRTHEMTGITESFMAAAEDAGFAWIADLNDVGPEMPSGVGAVPLNIVNGVRTSSAVGYLMPALGRPNLTLLARTRAVRLRFSATTAVGVDAIGPGGPVSLSADRIVLCAGAIQSAHLLMLSGVGEEEVLRSAGVKVLMALPVGMGCSDHPEWVMPTNWAVAVDRPVLEVLLSTHDGIEIRPYTGGFVAMTGDGTAGHRDWPHIGVALMQPRARGRITLVSSDPQIPVRIEHRYDSEPADVAALRQGSALAHELCGAATRIGPAVWATSQHLCGSAPMGTDDDPRAVVDPRCRVRGIENLWVIDGSVLPSITSRGPHATIVMLGHRAAEFVQ

>114|CORE_REP|Org28_Gene2952#

MALQTGEPRTLAEKIWDDHIVVSGGGCAPDLIYIDLHLVHEVTSPQAFDGLRLAGRRVRRPELTLATEDHNVPTVDIDQPIADPVSRTQVETLRRNCAEFGIRLHSMGDIEQGIVHVVGPQLGLTQPGMTIVCGDSHTSTHGAFGALAMGIGTSEVEHVLATQTLPLRPFKTMAVNVDGRLPDGVSAKDIILALIAKIGTGGGQGHVIEYRGSAIESLSMEGRMTICNMSIEAGARAGMVAPDETTYAFLRGRPHAPTGAQWDTALVYWQRLRTDVGAVFDTEVYLDAASLSPFVTWGTNPGQGVPLAAAVPDPQLMTDDAERQAAEKALAYMDLRPGTAMRDIAVDAVFVGSCTNGRIEDLRVVAEVLRGRKVADGVRMLIVPGSMRVRAQAEAEGLGEIFTDAGAQWRQAGCSMCLGMNPDQLASGERCAATSNRNFEGRQGAGGRTHLVSPAVAAATAVRGTLSSPADLN

>115|CORE_REP|Org18_Gene1009#

MRRNRRGSPARPAARFVRPAIPSALSVALLVCTPGLATADPQTDTIAALIADVAKANQRLQDLSDEVQAEQESVNKAMVDVETARDNAAAAEDDLEVSQRAVKDANAAIAAAQHRFDTFAAATYMNGPSVSYLSASSPDEIIATVTAAKTLSASSQAVMANLQRARTERVNTESAARLAKQKADKAAADAKASQDAAVAALTETRRKFDEQREEVQRLAAERDAAQARLQAARLVAWSSEGGQGAPPFRMWDPGSGPAGGRAWDGLWDPTLPMIPSANIPGDPIAVVNQVLGISATSAQVTANMGRKFLEQLGILQPTDTGITNAPAGSAQGRIPRVYGRQASEYVIRRGMSQIGVPYSWGGGNAAGPSKGIDSGAGTVGFDCSGLVLYSFAGVGIKLPHYSGSQYNLGRKIPSSQMRRGDVIFYGPNGSQHVTIYLGNGQMLEAPDVGLKVRVAPVRTAGMTPYVVRYIEY

>116|CORE_REP|Org17_Gene504#

MTATRLPDGFAVQVDRRVRVLGDGSALLGGSPTRLLRLAPAARGLLCDGRLKVRDEVSAELARILLDATVAHPRPPSGPSHRDVTVVIPVRNNASGLRRLVTSLRGLRVIVVDDGSACPVESDDFVGAHCDIEVLHHPHSKGPAAARNTGLAACTTDFVAFLDSDVTPRRGWLESLLGHFCDPTVALVAPRIVSLVEGENPVARYEALHSSLDLGQREAPVLPHSTVSYVPSAAIVCRSSAIRDVGGFDETMHSGEDVDLCWRLIEAGARLRYEPIALVAHDHRTQLRDWIARKAFYGGSAAPLAVRHPDKTAPLVISGGALMAWILMSIGTGLGRLASLVIAVLTGRRIARAMRCAETSFLDVLAVATRGLWAAALQLASAICRHYWPLALLAAILSRRCRRVVLIAAVVDGVVDWLRRREGADDDAEPIGPLTYLVLKRVDDLAYGAGLWYGVVRERNIGALKPQIRT

>117|CORE_REP|Org68_Gene2390#

MQGQLSRTRVYAVPVPGSAQSAYACGVERLLASYRSIPATASIRLAKPTSNLFRARVKHDARGLDASGLTGVIGIDPEARTADVAGMCTYEDLIAATLHYGLSPLVVPQLRTITLGGAVTGLGIESASFRNGLPHESVLEMDILTGAGELLTVSPGQHSDLYRAFPNSYGTLGYSTRLRIQLEPVRPFVALRHIRFSSLTAMVAAMERIIDTGGLDGESVDYLDGVVFSADESYLCIGMQTSVPGPVSDYTGQDIYYRSIQHEAGIKEDRLTIHDYFWRWDTDWFWCSRSFGAQNPRLRRWWPRRYRRSSVYWRLMALDQRFGIADRFENSRGRPARERVVQDIEVPIERTCEFLEWFGENVPISPIWLCPLRLRDHAGWPLYPIRPDRSYVNIGFWSSVPVGATEGATNRKIENKVSALDGHKSLYSDSFYTREEFDELYGGETYNTVKKAYDPDSRLLDLYAKAVQRR

>118|CORE_REP|Org128_Gene1845#

MAVGDDEEKVRAERARAIGLFRYQLIWEAADAAHSTKQRGKMVRELASREHTDPFGRRVRISRQTIDRWIRGWRAGGFDALVPNPRQCTPRTPAEVLELAVALRRENPQRTAAAIRRILRTQLGWAPDERTLQRNFHRLGLTGATTGSAPAVFGRFEAEHPNALWTGDVLHGIRIDLRKTYLFAFLDDHSRLVPGYRWGHAEDTVRLAAALRPALASRGVPNAVYVDNGSPYVDAWLLRACAKLGVRLVHSTPGRPQGRGKIERFFRTVREQFLVEITGEPDVVGRHYVADLAELNRLFTAWVETVYHRSVHSETGQTPLARWSAGGPIPLPAPETLTEAFLWEEHRRVTKTATVSLHGNRYEIDPALVGRKVELVFDPFDLTRIEVRLAGAPMGRAIPYHIGRHSHPKAKPETPTAPPKPSGIDYAQLIETAHAAELARGVNYTALTGAADQIPGQLDLLTGQEAQPK

>119|CORE_REP|Org59_Gene321#

MASTLTTGLPPGPRLPRYLQSVLYLRFREWFLPAMHRKYGDVFSLRVPPYADNLVVYTRPEHIKEIFAADPRSLHAGEGNHILGFVMGEHSVLMTDEAEHARMRSLLMPAFTRAALRGYRDMIASVAREHITRWRPHATINSLDHMNALTLDIILRVVFGVTDPKVKAELTSRLQQIINIHPAILAGVPYPSLKRMNPWKRFFHNQTKIDXXXXRXXXXRRIDSDLTARTDVLSRLLQTKDTPTKPLTDAELRDQLITLLLAGHETTAAALSWTLWELAHAPEIQSQVVWAAVGGDDGFLEAVLKEGMRRHTVIASTARKVTAPAEIGGWRLPAGTVVNTSILLAHASEVSHPKPTEFRPSRFLDGSVAPNTWLPFGGGVRRCLGFGFALTEGAVILQEIFXRFTITAAGPSKGETPLVRNITTVPKHGAHLRLIPQRRLGGXWXQXPXJAXGAAHLPRNRVPPRVPNR

>121|CORE_REP|Org1_Gene2216#

MRARRLVMLRHGQTDYNVGSRMQGQLDTELSELGRTQAVAAAEVLGKRQPLLIVSSDLRRAYDTAVKLGERTGLVVRVDTRLRETHLGDWQGLTHAQIDADAPGARLAWREDATWAPHGGESRVDVAARSRPLVAELVASEPEWGGADEPDRPVVLVAHGGLIAALSAALLKLPVANWPALGGMGNASWTQLSGHWARAPTSRASGGALMCGMLRRRSPAMSSRRGRRPALLVFADSLAYYGPTGGLPADDPRIWPNIVASQLDWDLELIGRIGWTCRDVWWAATQDPRAWAALPRAGAVIFATGGMDSLPSVLPTALRELIRYVRPSWLRRWVRDGYAWVQPRLSPVARAALPPHLTAEYLEKTRGAIDFNRPGIPIIASLPSVHIAETYGKAHHGRAGTVAAITEWAQHHDIPLVDLKAAVAEQILSGYGNRDGIHWNFEAHQAVAELMLKALAEAGVPNEKSRG

>123|CORE_REP|Org94_Gene1437#

MNWTVDIPIDQLPSLPPLPTDLRTRLDAALAKPAAQQPTWPADQALAMRTVLESVPPVTVPSEIVRLQEQLAQVAKGEAFLLQGGDCAETFMDNTEPHIRGNVRALLQMAVVLTYGASMPVVKVARIAGQYAKPRSADIDALGLRSYRGDMINGFAPDAAAREHDPSRLVRAYANASAAMNLVRALTSSGLASLHLVHDWNREFVRTSPAGARYEALATEIDRGLRFMSACGVADRNLQTAEIYASHEALVLDYERAMLRLSDGEDGEPQLFDLSAHTVWIGERTRQIDGAHIAFAQVIANPVGVKLGPNMTPELAVEYVERLDPHNKPGRLTLVSRMGNHKVRDLLPPIVEKVQATGHQVIWQCDPMHGNTHESSTGFKTRHFDRIVDEVQGFFEVHRALGTHPGGIHVEITGENVTECLGGAQDISETDLAGRYETACDPRLNTQQSLELAFLVAEMLRD

>125|CORE_REP|Org87_Gene220#

MIRAAFACLAATVVVAGWWTPPAWAIGPPVVDAAAQPPSGDPGPVAPMEQRGACSVSGVIPGTDPGVPTPSQTMLNLPAAWQFSRGEGQLVAIIDTGVQPGPRLPNVDAGGDFVESTDGLTDCDGHGTLVAGIVAGQPGNDGFSGVAPAARLLSIRAMSTKFSPRTSGGDPQLAQATLDVAVLAGAIVHAADLGAKVINVSTITCLPADRMVDQAALGAAIRYAAVDKDAVIVAAAGNTGASGSVSASCDSNPLTDLSRPDDPRNWAGVTSVSIPSWWQPYVLSVASLTSAGQPSKFSMPGPWVGIAAPGENIASVSNSGDGALANGLPDAHQKLVALSGTSYAAGYVSGVAALVRSRYPGLNATEVVRRLTATAHRGARESSNIVGAGNLDAVAALTWQLPAEPGGGAAPAKPVADPPVPAPKDTTPRNVAFAGAAALSVLVGLTAATVAIARRRREPTE

>126|CORE_REP|Org67_Gene3996#

MSIRPAENSTLDIRHVIGIGTPKAVDLWLDVVTELPDRARELGSLSKAELGKLGPLLDGTNAVELFESIDDKLAAEALHAMDPSLAATFLEALDSDHAANILREFKEPKREALLTLLPLERAMVLRGLLSWPEDCAAAHMVPETLTVRPNMTVSQAVASVRERASGLRSDARTTAYVYVTDADSHLLGVIAFRALVLANPEQRVRELMGDDLIVVSPLTDKELAAQTIMGHNLMAVPVVDADNRLLGIIAEDEAIDIAEEEATEDAERQGGSAPLEVPYLRASPWLLWRKRVVWLLVLFAAEAYTGSVLRAFSDEMQAVIALAFFIPLLIGTGGNTGTQIATTLVRAMATGQVRFRDVPAVLAKELSTGVLVGLTMAAAAVVRAWTLGVGPQVTLTVALTVAAIVVWSSLVAAVLPPLLKKLRIDPAIVSGPMIATIVDGTGLLIYFLVAHLTLTELHGL

>134|CORE_REP|Org99_Gene1764#

MTPRSYCVVGGGISGLTSAYRLRQAVGDDATITLFEPADRLGGVLRTEHIGGQPMDLGAEAFVLRRPEMPALLAELGLSDRQLASTGARPLIYSQQRLHPLPPQTVVGIPSSAGSMAGLVDDATLARIDAEAARPFTWQVGSDPAVADLVADRFGDQVVARSVDPLLSGVYAGSAATIGLRAAAPSVAAALDRGATSVTDAVRQALPPGSGGPVFGALDGGYQVLLDGLVRRSRVHWVRARVVQLERGWVLRDETGGRWQADAVILAVPAPRLARLVDGIAPRTHAAARQIVSASSAVVALAVPGGTAFPHCSGVLVAGDESPHAKAITLSSRKWGQRGDVALLRLSFGRFGDEPALTASDDQLLAWAADDLVTVFGVAVDPVDVRVRRWIEAMPQYGPGHADVVAELRAGLPPTLAVAGSYLDGIGVPACVGAAGRAVTSVIEALDAQVAR

>138|CORE_REP|Org116_Gene928#

MSTFIGQLFGFAVIVYLVWRFIVPLVGRLMSARQDTVRQQLADAAAAADRLAEASQAHTKALEDAKSEAHRVVEEARTDAERIAEQLEAQADVEAERIKMQGARQVDLIRAQLTRQLRLELGHESVRQARELVRNHVADQAQQSATVDRFLDQLDAMAPATADVDYPLLAKMRSASRRALTSLVDWFGTMAQDLDHQGLTTLAGELVSVARLLDREAVVTRYLTVPAEDATPRIRLIERLVSGKVGAPTLEVLRTAVSKRWSANSDLIDAIEHVSRQALLELAERAGQVDEVEDQLFRFSRILDVQPRLAILLGDCAVPAEGRVRLLRKVLERADSTVNPVVVALLSHTVELLRGQAVEEAVLFLAEVAVARRGEIVAQVGAAAELSDAQRTRLTEVLSRIYGHPVTVQLHIDAALLGGLSIAVGDEVIDGTLSSRLAAAEARLPD

>143|CORE_REP|Org1_Gene2272#

MMRRPITLAEQLDAEDAKLVVLARAAMARAEAGAGAAVRDVDGRTYAAAPVALSALELTGLQAAVAAAVSSGATGLQAAVLVAGSVDDPGIAAVRELARPLRSSSPIGQVTRYDAERAFTAEKSGKSLPVFTFGEETGMTEFHSGFVCLVGRPNTGKSTLTNALVGAKVAITSTRPQTTRHAIRGIVHSDDFQIILVDTPGLHRPRTLLGKRLNDLVRETYAAVDVIGLCIPADEAIGPGDRWIVEQLRSTGPANTTLVVIVTKIDKVPKEKVVAQLVAVSELVTNAAEIVPVSAMTGDRVDLLIDVLAAALPAGPAYYPDGELTDEPEEVLMAELIREAALQGVRDELPHSLAVVIDEVSPREGRDDLIDVHAALYVERDSQKGIVIGKGGARLREVGTAARSQIENLLGTKVYLDLRVKVAKNWQRDPKQLGRLGF

>144|CORE_REP|Org40_Gene2846#

MVLPKPTPRGRELIRQAAKVALHPTPEWLDELDRATLAAHPSIAADPALATVVSRANRSHLIHFATANLRKPGQPVPANLGPDPLRMARDLVRRGLDASALDVYRVGQNVAWQRWTEIAFGLTTDPQELHELLTLPFRSASEFIDATLAGLAAQMQLEYDELTRDVHAEHRRIVELILDGAPISRQSAEAKLGYPLDRSHTAAIIWYDDPDDNQNHLDHTARAFGRALGCPQPLIAVASAATRWVWVSDAATLDTDRIHQVLDHAPHARIAVGTTARGIDGFRRSHRDALATQRMLARLRSQQRLAFFADIHMIAVLTENPDSAADFITSTLGDLESASPQLLTTVLTYINEQCNASRAAHVLHTHRNTLLRRLETAQRLLPRPLDHTIIQVAVAISALQWRGSQTSDPVETPVEGITSPPPESLGRRRSRLAQLER

>145|CORE_REP|Org118_Gene2593#

MYAGAGAAPLMAAGATWNGLAVELSTTASSVESVIMQLTTEQWLGPASMSMVVAAQPYLAWLTYTAESAAHAAAQAMASAAAFEAAFAMTVPPAEVAANRALLAALVATNVLGQNTPAIMATEAHYGEMWAQDALAMYGYAASSAAAGRLNPLITPSQTANMAGLAGQAAAVSHAAAASTVQQVGLGSLISNLPNAVMGFASPLTSAADAAGLGGIIQDIEELLGITFVQNAINGAVNTTAWFVMATIPNAVFLGHAFAALNPATVTAAADAVPAAAAAAGLAHTVTPVGVGGASLTASLGEASSVGGLSVPAGWSTAAPAMTFWYHGTGGLGLGGPRGSRASRRNCRVWRGFLGRPKEPVPMPGLGTGSSPSSCPNRSSSDWPVAHRPGRPAMSRTATRQAWPVRQPTVGPQPTPPGPSIKPPEPWRRSRPRRAR

>146|CORE_REP|Org118_Gene2595#

MDFGALPPEVNSARMYGGAGAADLLAAAAAWNGIAVEVSTAASSVGSVITRLSTEHWMGPASLSMAAAVQPYLVWLTCTAESSALAAAQAMASAAAFETAFALTVPPAEVVANRALLAELTATNILGQNVSAIAATEARYGEMWAQDASAMYGYAAASAVAARLNPLTRPSHITNPAGLAHQAAAVGQAGASAFARQVGLSHLISDVADAVLSFASPVMSAADTGLEAVRQFLNLDVPAVRRIRVSRPGWRGRLCHGRHWQYDASCRCYGNRWRSRSRWRRGSRGGTRGCPSGRRRNSADRRFGQCVRGWSPVGAGKLVYCSAGDGSRRGLGWHRLGSSRGGRPDRSDAACPWNGRSRQQCWCRLRTTVRSQADCYAQARPLLIWRHRDKRTRPTSAPPRPHCANPPAARVSFRCCGERSGATLKRIVLRLPVPA

>148|CORE_REP|Org7_Gene3716#

MRKDKALARRLPAAVAAAVIAVELGGCGSADSWVEAAPAQGWPAQYGDAANSSYTTTNGATNLTLRWTRSVKGSLAAGPALSARGYLALNGQTPAGCSLMEWQNDNNGRQRWCVRLVQGGGFAGPLFDGFDNLYVGQPGAIISFPPTQWTRWRQPVIGMPSTPRFLGHGRLLVSTHLGQLLVFDTRRGMVVGSPVDLVDGIDPTDATRGLADCAPARPGCPVAAAPAFSSVNGTVVVSVWQPGEPAAKLVGLKYHAEQLVREWTSDAVSAGVLASPVLSADGSTVYVNGRDHRLWALNAADGKAKWSAPLGFLAQTPPALTPHGLIVSGGGPDTALAAFRDAGDHAEGAWRRDDVTALSTASLAGTGVGYTVISGPNHDGTPGLSLLVFDPANGHTVNSYPLPGATGYPVGVSVGNDRRVVTATSDGQVYSFAP

>149|CORE_REP|Org118_Gene2287#

MAPRVCVVGSVNMDLTFVVDALPRPGETVLAASLTRTPGGKGANQAVAAARAGAQVQFSGAFGDDPAAAQLRAHLRANAVGLDRTVTVPGPSGTAIIVVDASAENTVLVAPGANAHLTPVPSAVANCDVLLTQLEIPVATALAAARAAQSADAVVMVNASPAGQDRSSLQDLAAIADVVIANEHEANDWPSPPTHFVITLGVRGARYVGADGVFEVPAPTVTPVDTAGAGDVFAGVLAANWPRKPRFAGRATARIAAGLRCGCAGNFGVRCRRLRTGRRRDRCGPASQPPQRFMTTATHRRRPADANDHRGRLSAGTLRGFQCGRIRLTQLAAAPTHRIHRLSRRQRPGRFAGVDYRDVLCHRPDRDGGRCGAAADQRCPTAEYSAHGLDSGCRHNPGDGRDRGHGLRPACDGRFVADRGGRAGEHHSGAHRPV

>150|CORE_REP|Org36_Gene786#

MTDTRTYVLDTSVLLSDPWACSRFAEHDVVVPLVVISELEAKRHHHELGWFARQALRLFDDLRLEHGRLDQPIPVGTQGGTLHVELNHTDPAVLPAGFRTDSNDSRILSCAANLAAEGKRVTLVSKDIPLRVKAAAVGLAADEYHAQDVVVSGWSGMHELETASADIDALFADGEIDLVEARDLPCHTGIRLLGGGSHALGRVNAHKRVQLVRGDREAFGLRGRSAEQRVALDLLLDESVGIVSLGGKAGTGKSALALCAGLEAVLERRTHRKVVVFRPLYAVGGQELGYLPGSESEKMGPWAQAVFDTLEGLASPAVLEEVLSRGMLEVLPLTHIRGRSLHDSFVIVDEAQSLERNVLLTVLSRLGTGSRVVLTHDIAQRDNLRVGRHDGVAAVIEKLKGHPLFAHITLLRSERSPIAALVTEMLEEITGPR

>151|CORE_REP|Org12_Gene3891#

MSSWPPRAGSTGSTIAASTSTAATSRRSNSRLPTTLNARDQPPAEVSDQRVSGLTGAVHYAGAGSGPLFMAAAAWEGLAADLRASASSFDAVIAGLAAGPWSGPASVAMAGAAAPYVGWLSAAAGQAELSAGQATAAATAFEAALAATVHPAAVTANRVLLGALVATNILGQNTPAIAATEFDYVEMWAQDVGAMVGYHAGAAAVAETLTPFSVPPLDLAGLASQAGAQLTGMATSVSAALSPIAEGAVEGVPAVVAAAQSVAAGLPVDAALQVGQAAAYPASMLIGPMMQLAQMGTTANTAGLAGAEAAGLAAADVPTFAGDIASGTGLGGAGGLGAGMSAELGKARLVGAMSVPPTWEGSVPARMASSAMAGLGAMPAEVPAAGGPMGMMPMPMGMGGAGAGMPAGMMGRGGANPHVVQARPSVVPRVGIG

>155|CORE_REP|Org40_Gene1460#

MSRADDDAVGVPPTCGGRSDEEERRIVPGPNPQDGAKDGAKATAVPREPDEAALAAMSNQELLALGGKLDGVRIAYKEPRWPVEGTKAEKRAERSVAVWLLLGGVFGLALLLIFLFWPWEFKAADGESDFIYSLTTPLYGLTFGLSILSIAIGAVLYQKRFIPEEISIQERHDGASREIDRKTVVANLTDAFEGSTIRRRKLIGLSFGVGMGAFGLGTLVAFAGGLIKNPWKPVVPTAEGKKAVLWTSGWTPRYQGETIYLARATGTEDGPPFIKMRPEDMDAGGMETVFPWRESDGDGTTVESHHKLQEIAMGIRNPVMLIRIKPSDLGRVVKRKGQESFNFGEFFAFTKVCSHLGCPSSLYEQQSYRILCPCHQSQFDALHFAKPIFGPAARALAQLPITIDTDGYLVANGDFVEPVGPAFWERTTT

>157|CORE_REP|Org78_Gene1074#

MSAELSQSPSSSPLFSLSGADIDRAAKRIAPVVTPTPLQPSDRLSAITGATVYLKREDLQTVRSYKLRGAYNLLVQLSDEELAAGVVCSSAGNHAQGFAYACRCLGVHGRVYVPAKTPKQKRDRIRYHGGEFIDLIVGGSTYDLAAAAALEDVERTGATLVPPFDDLRTIAGQGTIAVEVLGQLEDEPDLVVVPVGGGGCIAGITTYLAERTTNTAVLGVEPAGAAAMMAALAAGEPVTLDHVDQFVDGAAVNRAGTLTYAALAAAGDMVSLTTVDEGAVCTAMLDLYQNEGIIAEPAGALSVAGLLEADIEPGSTVVCLISGGNNDVSRYGEVLERSLVHLGLKHYFLVDFPQEPGALRRFLDDVLGPNDDITLFEYVKRNNRETGEALVGIELGSAADLDGLLARMRATDIHVEALEPGSPAYRYLL

>159|CORE_REP|Org59_Gene1576#

MDFGALPPEVNSGRMYCGPGSAPMVAAASAWNGLAAELSVAAVGYERVITTLQTEEWLGPASTLMVEAVAPYVAWMRATAIQAEQAASQARAAAAAYETAFAAIVPPPLIAANRARLTSLVTHNVFGQNTASIAATEAQYAEMWAQDAMAMYGYAGSSATATKVTPFAPPPNTTSPSAAATQLSAVAKAAGTSAGAAQSAIAELIAHLPNTLLGLTSPLSSALTAAATPGWLEWFINWYLPISQLFYNTVGLPYFAIGIGNSLITSWRALGXXGPXAAEAAAAAPAAVGAAVGGTGPVSAGLGNAATIGKLSLPPNWAGASPSLAPTVGSASAPLVSDIVEQPEAGAAGKPVGRHAASRFGHRYGGCGSPLRVPGYGDVPAAVCRITRGLPYPRTRGRHICRTIXRLPXRQTPSRTCVGTXPXAIFS

>161|CORE_REP|Org119_Gene1671#

MDFGLLPPEINSGRMYTGPGPGPMLAAATAWDGLAVELHATAAGYASELSALTGAWSGPSSTSMASAAAPYVAWMSATAVHAELAGAQARLAIAAYEAAFAATVPPPVIAANRAQLMVLIATNIFGQNTPAIMMTEAQYMEMWAQDAAAMYGYAGSSATASRMTAFTEPPQTTNHGQLGAQSSAVAQTAATAAGGNLQSAFPQLLSAVPRALQGLALPTASQSASATPQWVTDLGNLSTFLGGAVTGPYTFPGVLPPSGVPYLLGIRMARVLGTQNGQGVSALLGKIGGKPITGALAPLAEFALHTPILGSEGLGGGSVSAGIGRAGLVGKLSVPQGWTVAAPEIPSPAAALQATRLAAAPIAATDGAGALLGGMALSGLAGRAAAGSTGHPIGSAAAPAVGAAAAAVEDLATEANIFVIPAMDD

>164|CORE_REP|Org59_Gene1167#

MAEIVLDHVNKSYPDGHTAVRDLNLTIADGEFLILVGPSGCGKTTTLNMIAGLEDISSGELRIAGERVNEKAPKDRDIAMVFQSYALYPHMTVRQNIAFPLTLAKMRKADIAQKVSETAKILDLTNLLDRKPSQLSGGQRQRVAMGRAIVRHPKAFLXXEPLSNLDAKLRVQMRGEIAQLQRRLGTTTVYVTHDQTEAMTLGDRVVVMYGGIAQQIGTPEELYERPANLFVAGFIGSPAMNFFPARLTAIGLXLPFGEVTLAPEVQGVIAAHPKPENVIVGVRPEHIQDAALIDAYQRIRALTFQVKVNLVESLGADKYLYFTTESPAVHSVQLDELAEVEGESALHENQFVARVPRRVQGSHRAVGRVGFRYRQTCRLRRRLRCEPDHSAPRLMAASXHISPXHAXGFGAFCVCSPTRS

>165|CORE_REP|Org59_Gene1373#

MTASVNSLDLAAIRADFPILKRIMRGGNPLAYLDSGATSQRPLQVLDAEREFLTASNGAVHRGAHXLMEEATDAYEQGRADIVGGVIRRQXTRTSWCSPKMPPRRSTWCHMCWGTAVSSVTXGPGDVIVTTELEHHANLIPWQELARRTGATLRWXGVTDDGRIDLDSLYLDDRVKVVAFTHHSNVTGVLTPVSELVSRAHQSGALTVLXXCQSVPHQPVDLHELGVDFAAFSGHKMLGPNGIGVLYGRRELLAQMPPFLTGGSMIETVTXEGATYAPAPQRFEAGTPMTSQVVGLAAAARYLGAIGMAAVEAHERELVAAAIEGLSGIDGVRILGPTSMRDRGSPVAFVVEGVHAHDVGQVLDDGGVAVRVGHHCALPLHRRFGLAATARASFAVYNTADEVDRLVAGVRRSRHFFGRA

>167|CORE_REP|Org7_Gene3851#

MASGSGLCKTTSNFIWGQLLLLGEGIPDPGDIFNTGSSLFKQISDKMGLAIPGTNWIGQAAEAYLNQNIAQQLRAQVMGDLDKLTGNMISNQAKYVSDTRDVLRAMKKMIDGVYKVCKGLEKIPLLGHLWSWELAIPMSGIAMAVVGGALLYLTIMTLMNATNLRGILGRLIEMLTTLPKFPGLPGLPSLPDIIDGLWPPKLPDIPIPGLPDIPGLPDFKWPPTPGSPLFPDLPSFPGFPGFPEFPAIPGFPALPGLPSIPNLFPGLPGLGDLLPGVGDLGKLPTWTELAALPDFLGGFAGLPSLGFGNLLSFASLPTVGQVTATMGQLQQLVAAGGGPSQLASMGSQQAQLISSQAQQGGQQHATLVSDKKEDEEGVAEAERAPIDAGTAASQRGQEGTVLRSDTESPAGLCHSESKP

>171|CORE_REP|Org98_Gene1547#

MSHVTAAPNVLAASAGELAAIGSTMRAANAAAAAPTAGVLAAGGDDVSAGIAALFGARAQAYQAISAQAALFHDRFVQILQEGAAAYAMAEAANALPLQKAQGVVSELAQDRTGGTGTGQSRGAGGFGGVGQAGGKGWDGGPIGNGQVGEQHGAGQLGSTDGNPGVAGAAHGSGVSASHGSGATGAAGVADPGGSGAGVGSAAGNGTGAGSADAVGGAGTGRDIVGSVRGDGGVGMASGDGGLSTGAAGASAEGGLMPGFGGAPWVGGHWGLGGEGHSGAIGGVGEQVAPAVATAPAVSPATTSAVAAESGSTPATKAQAMHATTNPGNAAHQGNPADPGNSARRADGGRDEQLLLLPLTSLRGLRHTLKKLSGLRARNGLLTASGDNASGSGRPWDRDQLLRALGLRPPGHE

>173|CORE_REP|Org19_Gene3437#

MSMPAKVSVLITVTGMDQPGVTSALFEVLAQHGVELLNVEQVVIRGRLTLGVLVSCPLDVADGTALRDDVAAAIHGVGLDVAIERSDDLPIIRQPSTHTIFVLGRPITAGAFSAVAREVAALGVNIDFIRGISDYPVTGLELRVSVPPGCVGPLQIALTKVAAEEHVDVAVEDYGLAWRTKRLIVFDVDSTLVQGEVIEMLAARAGAQGQVAAITEAAMRGELDFAESLQRRVATLAGLPATVIDDVAEQLELMPGARTTIRTLRRLGFRCGVVSGGFRRIIEPLARELMLDFVASNELEIVDGILTGRVVGPIVDRPGKAKALRDFASQYGVPMEQTVAVGDGANDIDMLGAAGLGIAFNAKPALREVADASLSHPYLDTVLFLLGVTRGEIEAADAGDCGVRRVEIPAD

>175|CORE_REP|Org59_Gene662#

MNAHVTSREGVNEFDDGIVIVGGGLAAARTAEQLRRAGYSGRLTIVSDEVHLPYDRPPLSKEVLRSEVDDVALKPREFYDEKDIALRLGSAAVSLDTGEQTVTLADVTVLGYDELVIATGLVPRRIPSLPDLDGIRVLRSFDESMALRKHASAARHAVVVGAGFIGCEVAASLRGLGVDVVLVEPQPAPLASVLGEQIGQLVTRLHRDEGVDVRTGVTVAEVRGKGHVDAVVLTDGTELPADLVVVGIGSTPATEWLEGSGVEVDNGVICDKAGRTSAPNVWALGDVASWRDPMGHQARVEHWSNVADQARVVVPAMLGTDVPTGVVVPYFWSDQYDVKIQCLGXPXVHRRCASGRGRRAQVPCLLRARWRAGWRGRWRDGRQGHEGARXDRRGRAHRRSVRPNSGLELT

>178|CORE_REP|Org2_Gene843#

MRPLWRHPDAADRASLSKSRNLSPVVNFTKELSPLFEERPCMTYTGSIRCEGDTWDLASSVGATATMVAAARAMATRAANPLINDQFAEPLVRAVGVDVLTRLASGELTASDIDDPERPNASMVRMAEHHAVRTKFFDEFFMDATRAGIRQVVILASGLDSRAYRLAWPAQTVVYEIDQPQVMEFKTRTLAELGATPTADRRVVTADLRADWPTALGAAGFDPTQPTAWSAEGLLRYLPPEAQDRLLDNVTALSVPDSRFATESIRNFKPHHEERMRERMTILANRWRAYGFDLDMNELVYFGDRNEPASYLSDNGWLLTEIKSQDLLTANGFQPFEDEEVPLPDFFYVSARLQRKHRQYPAHRKPAPSWRHTACPVNELSKSAAYTMTRSDAHQASTTAPPPPGLTG

>180|CORE_REP|Org145_Gene1996#

MVKPERRTKTDIAAAATIAVVVAVAASLIWWTSDARATISRPAAVAVPTPAPAREVPTSLKQLWTAASPATRVPVVVGGTVATGDGRQVDGRDPATGESLWSYARDTDLCGVTWVYHYAVAVYRYDRGCGQVSTIDGSTGRRGAARSGYADPRVRLFSDGTTVLSAGDTRLELWRSDMVRMLAYGEIDARVKPSNRGLQSGCTLESAAASSAAVSVLEACTNQADLRLVLLRPGKEDDEPIQRIVPEPGVRPGSGARVLVVSQNNTAVYLPARSGAQPRVDVIDETGATVSSTLLAKPPSTSAVASRTGNLVTWWTGDALLVFDAGNLTQRYTIAAGETTAPVGPGVMMAGQLLVPVTGGIGVYDPVSGANNRYIPVTRPPSTSAVIPAVSGSRVIEQRGDTLVALG

>184|CORE_REP|Org19_Gene1581#

MMFVTGIVLFALAILISVALHECGHMWVARRTGMKVRRYFVGFGPTLWSTRRGETEYGVKAVPLGGFCDIAGMTPVEELDPDERDRAMYKQATWKRVAVLFAGPGMNLAICLVLIYAIALVWGLPNLHPPTRAVIGETGCVAQEVSQGKLEQCTGPGPAALAGIRSGDVVVKVGDTPVSSFDEMAAAVRKSHGSVPIVVERDGTAIVTYVDIESTQRWIPNGQGGELQPATVGAIGVGAARVGPVRYGVFSAMPATFAFTGDLTVEVGKALAALPTKVGALVRAIGGGQRDPQTPISVVGASIIGGDTVDHGLWVAFWFFLAQLNLILAAINLLPLLPFDGGHIAVAVFERIRNMVRSARGKVAAAPVNYLKLLPATYVVLVLVVGYMLLTVTADLVNPIRLFQ

>185|CORE_REP|Org68_Gene851#

MREVPHVLGIVLAGGEGKRLYPLTADRAKPAVPFGGAYRLIDFVLSNLVNARYLRICVLTQYKSHSLDRHISQNWRLSGLAGEYITPVPAQQRLGPRWYTGSADAIYQSLNLIYDEDPDYIVVFGADHVYRMDPEQMVRFHIDSGAGATVAGIRVPRENATAFGCIDADDSGRIRSFVEKPLEPPGTPDDPDTTFVSMGNYIFTTKVLIDAIRADADDDHSDHDMGGDIVPRLVADGMAAVYDFSDNEVPGATDRDRAYWRDVGTLDAFYDAHMDLVSVHPVFNLYNKRWPIRGESENLAPAKFVNGGSAQESVVGAGSIISAASVRNSVLSSNVVVDDGAIVEGSVIMPGTRVGRGAVVRHAILDKNVVVGPGEMVGVDLEKDRERFAISAGGVVAVGKGVWI

>186|CORE_REP|Org118_Gene1238#

MQYGLEVSSDVAGVAGGLLALSYRGAGVPLRELALVGLTAAIITYFATGPVRMLASRLGAVAYPRERDVHVTPTPRMGGLAMFLGIVGAVFLASQLPALTRGFVYSTGMPAVLVAGAVIMGIGLIDDRWGLDALTKFAGQITAASVLVTMGVAWSVLYIPVGGVGTIVLDQASSILLTLALTVSIVNAMNFVDGLDGLAAGLGLITALAICMFSVGLLRDHGGDVLYYPPAVISVVLAGACLGFLPHNFHRAKIFMGDSGSMLIGLMLAAASTTAAGPISQNAYGARDVFALLSPFLLVVAVMFVPMLDLLLAIVRRTRAGRSAFSPDKMHLHHRLLQIGHSHRRVVLIIYLWVGIVAFGAASSIFFNPRDTAAVMLGAIVVAGVATLIPLLRRGDDYYDPDLD

>189|CORE_REP|Org60_Gene2893#

MTSTSIPTFPFDRPVPTEPSPMLSELRNSCPVAPIELPSGHTAWLVTRFDDVKGVLSDKRFSCRAAAHPSSPPFVPFVQLCPSLLSIDGPQHTAARRLLAQGLNPGFIARMRPVVQQIVDNALDDLAAAEPPVDFQEIVSVPIGEQLMAKLLGVEPETVHELAAHVDAAMSVCEIGDEEVSRRWSALCTMVIDILHRKLAEPGDDLLSTIAQANRQQSTMTDEQVVGMLLTVVIGGVDTPIAVITNGLASLLHHRDQYERLVEDPGRVARAVEEIVRFNPATEIEHLRVVTEDVVIAGTALSAGSPAFTSITSANRDSDQFLDPDEFDVERNPNEHIAFGYGPHACPASAYSRMCLTTFFTSLTQRFPQLQLARPFEDLERRGKGLHSVGIKELLVTWPT

>190|CORE_REP|Org69_Gene2725#

MVEAGTRDPLESALLDSRYLVQAKIASGGTSTVYRGLDVRLDRPVALKVMDSRYAGDEQFLTRFRLEARAVARLNNRALVAVYDQGKDGRHPFLVMELIEGGTLRELLIERGPMPPHAVVAVLRPVLGGLAAAHRAGLVHRDVKPENILISDDGDVKLADFGLVRAVAAASITSTGVILGTAAYLSPEQVRDGNADPRSDVYSVGVLVYELLTGHTPFTGDSALSIAYQRLDADVPRASAVIDGVPPQFDELVACATARNPADRYADAIAMGADLEAIAEELALPEFRVPAPRNSAQHRSAALYRSRITQQGQLGAKPVHHPTRQLTRQPGDCSEPASGSEPEHEPITGQFAGIAIEEFIWARQHARRMVLVWVSVVLAITGLVASAAWTIGSNLSGLL

>193|CORE_REP|Org59_Gene1577#

MTLDVPVNQGHVPPGSVACCLVGVTAVADGIAGHSLSNFGALPPEINSGRMYSGPGSGPLMAAAAAWDGLAAELSSAATGYGAAISELTNMRWWSGPASDSMVAAVLPFVGWLSTTATLAEQAAMQARAAAAAFEAAFAMTVPPPAIAANRTLLMTLVDTNWFGQNTPAIATTESQYAEMWAQDAAAMYGYASAAAPATVLTPFAPPPQTTNATGLVGHATAVAALRGQHSWAAAIPWSDIQKYWMMFLGALATAEGFIYDSGGLTLNALQFVGGMLWXXRXXKKPVRPMAXXXAGGAAGWSAWSQLGAGPVAASATLAAKIGPMSVPPGWSAPPATPQAQTVARSIPGIRSAAEAAETSVLLRGAPTPGRSRAAHMGRRYGRRLTVMADRPNVG

>196|CORE_REP|Org93_Gene4068#

MPAPDPMRGDPPHPAPPRLRSPLXPTSGDPLHPAPPRLRSPLDPTSGDPLHPAPPRLRSPLDPTSGDPLHPAPPRLRSPLVLLDGASMWFRSFFGVPSSITAPDGRPVNAVRGFIDSMAVVITQQRPNRLAVCLDLDWRPQFRVDLIPSYKAHRVAEPEPNGQPDVEEVPDELTPQVDMIMELLDAFGIAMAGAPGFEADDVLGTLATRERRDPVIVVSGDRDLLQVVADDPVPVRVLYLGRGLAKATLFGPAEVAERYGLPAHRAGAAYAELALLRGDPSDGLPGVPGVGEKTAATLLARHGSLDQIMAAADDRKTTMAKGLRTKLLAASAYIKAADRVVRVATDAPVTLSTPTDRLPLVAADPERTAELATRFGVESSIARLQKALDTLPG

>199|CORE_REP|Org62_Gene1151#

MRMSALLSRNTSRPGLIGIARVDRNIDRLLRRVCPGDIVVLDVLDLDRITADALVEAEIAAVVNASSSVSGRYPNLGPEVLVTNGVTLIDETGPEIFKKVKDGAKVRLYEGGVYAGDRRLIRGTERTDHDIADLMREAKSGLVAHLEAFAGNTIEFIRSESPLLIDGIGIPDVDVDLRRRHVVIVADEPSGPDDLKSLKPFIKEYQPVLVGVGTGADVLRKAGYRPQLIVGDPDQISTEVLKCGAQVVLPADADGHAPGLERIQDLGVGAMTFPAAGSATDLALLLADHHGAALLVTAGHAANIETFFDRTRVQSNPSTFLTRLRVGEKLVDAKAVATLYRNHISGGAIALLALTMLIAIIVALWVSRTDGVVLHWIIDYWNRFSLWVQHLVS

>200|CORE_REP|Org59_Gene1665#

MDQQSTRTDITVNVDGFWMLQALLDIRHVAPELRCRPYVSTDSNDWLNEHPGMAVMREQGIVVNDAVNEQVAARMKVLAAPDLEVVALLSRGKLLYGVIDDENQPPGSRDIPDNEFRVVLARRGQHWVSAVRVGNDITVDDVTVSDSASIAALVMDGLESIXXAXPAAINAVNVPMEEMLEATKSWQESGFNVFSGGDLRRMGISAATVAALGQALSDPAAEVAVYARQYRDDAKXPSASXLSLKDGSGGRIALYQQARTAGXRRGXAGYLPXYPAVGASRSEDRFGYTALRRVENTQQSMTPGRETRSTTTNLSIRYNPDTYRANCSRIDCNTARQGQPQRFGREARXXKSELXEPQLPVGYRASVPTPTELPAPLKPRCNTFAMAGGTGR

>203|CORE_REP|Org59_Gene827#

MELCGXESGXARTPDARQAAVEAAGQARDELAGEAPSLAVLLGSRAHTDRAADVLSAVLQMIDPPALVGCIAQAIVAGRHEIEDEPAVVVWLASGLAAETFQLDFVRTGSGALITGYRFDRTARDLHLLLPXXXTFPSNLLIEHPNTFDLPGTAVVGGVVSGGRRRGDTRLFRDHDVLTSGVVGVRLPGMRGVPVVSQGCRPIGYPYIVTGADGILITELGGRPPLQRLREIVEGLSPDERALVSHGLQIGIVVDEHLAAPGQGDFVIRGLLGADPSTGSIEIDEVVQVGATMQFQVRDAAGADKDLRLTVERAAARLPGRAAGALLFTCNGRGRRMFGVADHDASTIEELLGGIPLAGFFAAGEIGPIAGRNALHGFTASMALFVDDME

>207|CORE_REP|Org140_Gene3432#

MSRVLLVTNDFPPRRGGIQSYLGQFVGRLVGSRAHAMTVYAPQWKGADAFDDAARAAGYRVVRHPSTVMLPGPTVDVRMRRLIAEHDIETVWFGAAAPLALLAPRARLAGASRVLASTHGHEVGWSMLPVARSVLRRIGDGTDVVTFVSSYTRSRFASAFGPAASLEYLPPGVDTDRFRPDPAARAELRKRYRLGERPTVVCLSRLVPRKGQDTLVTALPSIRRRVDGAALVIVGGGPYLETLRKLAHDCGVADHVTFTGGVATDELPAHHALADVFAMPCRTRGAGMDVEGLGIVFLEASAAGVPVIAGNSGGAPETVQHNKTGLVVDGRSVDRVADAVAELLIDRDRAVAMGAAGREWVTAQWRWDMLAAKLADFLRGDDAAR

>208|CORE_REP|Org71_Gene3018#

MYVRHLGLRDFRSWACVDLELHPGRTVFVGPNGYGKTNLIEALWYSTTLGSHRVSADLPLIRVGTDRAVISTIVVNDGRECAVDLEIATGRVNKARLNRSSVRSTRDVVGVLRAVLFAPEDLGLVRGDPADRRRYLDDLAIVRRPAIAAVRAEYERVLRQRTALLKSVPGARYRGDRGVFDTLEVWDSRLAEHGAELVAARIDLVNQLAPEVKKAYQLLAPESRSASIGYRASMDVTGPSEQSDTDRQLLAARLLAALAARRDAELERGVCLVGPHRDDLILRLGDQPAKGFASHGEAWSLAVALRLAAYQLLRVDGGEPVLLLDDVFAELDVMRRRALATAAESAEQVLVTAAVLEDIPAGWDARRVHIDVRADDTGSMSVVLP

>209|CORE_REP|Org59_Gene2935#

MDFALLPPEVNSARMYTGPGAGSLLAAAGGWDSLAAELATTAEAYGSVLSGLAALHWRGPAAESMAVTAAPYIGWLYTTAEKTQQTAIQARAAALAFEQAYAMXLPPPVVAANRIQLLALIATNFFGQNTAAIAATEAQYAEMWAQDAAAMYGYATASXAXALLTPGXXXPRQTTNPAGLTAQAAAVSQATDPLSLLIETVTQALQALTIPSFIPEDFTFLDAIFAGYATVGVTQDVESFVAGTIGAESNLGLLNVGDENPAEVTPGDFGIGELVSATSPGGGVSASGAGGAASVGNTVLASVGRANSIGQLSVPPSWAAPSTRPVSALSPAGLTTLPGTDVAEHGMPGVPGVPVAXGRASGVLPRYGVRLTVMAHPPAAGXPGA

>211|CORE_REP|Org59_Gene1179#

MTLPKERAAQGGLERIAHVDRVASLTGIRAVAALLVVGTHAAYTTGKYTHGYWGLMSSRMEIGVPIFFVLSGFLLFRPWVKXXXXRRPPAVVEPLCVAPGPADHARLHRHRSVGLPRLSLPHGGGXXPXXTXXGXFRNLTLTQIYTDGYLGAFLHQGLTQMWSLAVEVAFYLALPALAYLLLVLVCRRRWQPRLLLATMAGXTMISPAWLILVHNTHWMPDGARLWLPTYLAWFVGGMMLAVLAAMGVRCYAFVAIPLAVICYFIVSTPIAGAPTTSPTALAEALVKTAFYAVIAVLAVAPLALGDQGWYAQLLASRPMVFLGEISYEIFLIHLVTMEIAMVDVLGYRVYTSSMVNLCLVTLVLTIPLAWLLHRFTRVQGDRPS

>212|CORE_REP|Org59_Gene3118#

MTVLGADAVVIDGRICRPGWVHTADGRILSGGAGAPPMPADAEFPDAIVVPGFVDMHVHGGGGASFADGNAADIARAAEFHLRHGTTTTLASLVTAGPAELLSAVGALAEATRDGVVAGIHLEGPWLSPARCGAHDHTRMRAPDPAEIESVLAAADGAVRMVTLAPELPGSDAAIRRFRDAEVVVAVGHTDATYTQTRHAIDLGADSRXPXVXXAXPPLDHRAPGPVLALLCDPRVTVEIIADGVHVHPAVVHAVIEAVGPDRVAVVTDAIAAAGCGDGAFRLGTMPIEVESSVARVAGASTLAGSTTTMDQLFRTVAGLGSKSDSAGDVALAAAVQVTSATPARALGLTGVGRLAAGYAANLVVLDRDLRVTAVMVNDDWRVG

>214|CORE_REP|Org118_Gene1514#

MYADRDLPGAGGLAVRVIPCLDVDDGRVVKGVNFENLRDAGDPVELAAVYDAEGADELTFLDVTASSSGRATMLEVVRRTAEQVFIPLTVGGGVRTVADVDSLLRAGADKVAVNTAAIACPDLLADMARQFGSQCIVLSVDARTVPVGSAPTPSGWEVTTHGGRRGTGMDAVQWAARGADLGVGEILLNSMDADGTKAGFDLALLRAVRAAVTVPVIASGGAGAVEHFAPAVAAGADAVLAASVFHFRELTIGQVKAALAAEGITVPMTLDPKIAARLKRNADGLVTAVVQERGSGDVLMVAWMNDEALARTLQTREATYYSRSRAEQWVKGATSGHTQHVHSVRLDCDGDAVLLTVDQVGGACHIGDHSCFDAAVLLEPDD

>216|CORE_REP|Org60_Gene4003#

MTRSGHPVTLDDLPLRADLRGKAPYGAPQLAVPVRLNTNENPHPPTRALVDDVVRSVREAAIDLHRYPDRDAVALRADLAGYLTAQTGIQLGVENIWAANGSNEILQQLLQAFGGPGRSAIGFVPSYSMHPIISDGTHTEWIEASRANDFGLDVDVAVAAVVDRKPDVVFIASPNNPSGQSVSLPDLCKLLEVAPGIAIVDEAYGEFSSQPSAVSLVEEYPSKLVVTRTMSKAFAFAGGRLGYLIATPAVIDAMLLVRLPYHLSSVTQAAARAALRHSDDTLSSVAALIAERERVTTSLNDMGFRVIPSDANFVLFGEFADAPAAWRRYLEAGILIRDVGIPGYLRATTGLAEENDAFLRASARIATDLVPVTRSPVGAP

>217|CORE_REP|Org28_Gene1462#

MCQQGRPLGWDAVSDVPELIHGPLEDRHRELGASFAEFGGWLMPVSYAGTVSEHNATRTAVGLFDVSHLGKALVRGPGAAQFVNSALTNDLGRIGPGKAQYTLCCTESGGVIDDLIAYYVSDDEIFLVPNAANTAAVVGALQAAAPGGLSITNLHRSYAVLAVQGPCSTDVLTALGLPTEMDYMGYADASYSGVPVRVCRTGYTGEHGYELLPPWESAGVVFDALLAAVSAAGGEPAGLGARDTLRTEMGYPLHGHELSLDISPLQARCGWAVGWRKDAFFGRAALLAEKAAGPRRLLRGLRMVGRGVLRPGLAVLVGDETVGVTTSGTFSPTLQVGIGLALIDSDAGIEDGQQINVDVRGRAVECQVVCPPFVAVKTR

>218|CORE_REP|Org59_Gene1712#

MSALAFTILAVLLAGPTPALLARATWPLRAPRAAMVLWQAIALAAVLSSFSAGIAIASRLLMPGPDGRPTTSFVGAAGRLGWPLWAAYITVFALTVLVGARLAVAVVRVATATRRRRAHHRMVVDLVGVGHNGALAQPCARARDLRVLDVAQPLAYCLPGVRSRVVVSEGTLTALADAEVAAILTHERAHLRARHDLVLEAFTAVHAAFPRLVRSANALGAVQLLVELLADDAAVRAAGRTPLARALVACASGRAPSGALAVGGPSTVLRVRRLSGRGNSAVLSAAAYLAAAAVLVVPTVALAVPWLTQLQRLVHRLAKPGPTERQDMSSSESPAGIAQIGCHWPGRDGFQHRPKLRPARLHRGSAQSVGRQDRRAA

>223|CORE_REP|Org2_Gene4088#

MSSYYARRPLQSSGCSNSDSCWDGAPIEITESGPSVAGRLAALASRMTIKPLMTVGSYLSPLPLPLGFVDFACRVWRPGQGTVRTTINLPNATAQLVRAPGVRAADGAGRVVLYLHGGAFVMCGPNSHSRIVNALSGFAESPVLIVDYRLIPKHSLGMALDDCHDAYQWLRARGYRPEQIVLAGDSAGGYLALALAQRLQCDDEKPAAIVAISPLLQLAKGPKQDHPNIGTDAMFPARAFDALAAWVRAAAAKNMVDGRPEDLYEPLDHIESSLPPTLIHVSGSEVLLHDAQLGAGKLAAAGVCAEVRVWPGQAHLFQLATPLVPEATCSLRQIGQFIRDATADSSLSPVHRSRYVAGSPRAASRGAFGQSPI

>225|CORE_REP|Org100_Gene2093#

MSAHVATLHPEPPFALCGPRGTLIARGVRTRYCDVRAAQAALRSGTAPILLGALPFDVSRPAALMVPDGVLRARKLPDWPTGPLPKVRVAAALPPPADYLTRIGRARDLLAAFDGPLHKVVLARAVQLTADAPLDARVLLRRLVVADPTAYGYLVDLTSAGNDDTGAALVGASPELLVARSGNRVMCKPFAGSAPRAADPKLDAANAAALASSAKNRHEHQLVVDTMRVALEPLCEDLTIPAQPQLNRTAAVWHLCTAITGRLRNISTTAIDLALALHPTPAVGGVPTKAATELIAELEGDRGFYAGAVGWCDGRGDGHWVVSIRCAQLSADRRAALAHAGGGIVAESDPDDELEETTTKFATILTALGVEQ

>226|CORE_REP|Org59_Gene452#

MQLTPHFGNVQAHYDLSDDFFRLFLDPTQTYSCAYFERDDMTLQEAQIAKIDLALGKLNLEPGMTLLDIGCGWGATMRRAIEKYDVNVVGLTLSENQAGHVQKMFDQMDTPRSRRVLLEGWEKFDEPVDRIVSIGAFEHFGHQRYHHFFEVTHRTLPADGKMLLHTIVRPTFKEGREKGLTLTHELVHFTKFILAEIFPGGWLPSIPTVHEYAEKVGFRVTAVQSLQLHYARTLDMWATALEANKDQAIAIZSQTVYBSLHEVPDRLREAVPPGLHRRRPVHTGKVTGQSALAXXRPVPGRXATPGVSSATPGTXSGGDGLXGQCELSHVADALAEEVLTSGQIVHVFVVNLLGLKSNGAVLVSLQIRRPDV

>231|CORE_REP|Org5_Gene3476#

MSEHQSLPAPEASTEVRVAIVGVGNCASSLVQGVEYYYNADDTSTVPGLMHVRFGPYHVRDVKFVAAFDVDAKKVGFDLSDAIFASENNTIKIADVAPTNVIVQRGPTLDGIGKYYADTIELSDAEPVDVVQALKEAKVDVLVSYLPVGSEEADKFYAQCAIDAGVAFVNALPVFIASDPVWAKKFTDARVPIVGDDIKSQVGATITHRVLAKLFEDRGVQLDRTMQLNVGGNMDFLNMLERERLESKKISKTQAVTSNLKREFKTKDVHIGPSDHVGWLDDRKWAYVRLEGRAFGDVPLNLEYKLEVWDSPNSAGVIIDAVRAAKIAKDRGIGGPVIPASAYLMKSPPEQLPDDIARAQLEEFIIG

>234|CORE_REP|Org59_Gene2359#

MTDIGAPVTVQVAVDPPYPVVIGTGLLDELEDLLADRHKVAVVHQPGLAETAEEIRKRLAGKGVDAHRIEIPDAEAGKDLPVVGFIWEVLGRIGIGRKDALVSLGGGAATDVAGFAAATWLRGVSIVHLPTTLLGXVXWRSCRRRQDRHQXRXAGXNLVGXXHQDPLAVLVDLATLQTLPRDEMICGMAEVVKAGFIADPVILDLIEADPQAALDPAGDVLPELIRRAITVKAEVVAADEKESELREILNYGHTLGHAIERRERYRWRHGAAVSVGLVFAAELARLAGRLDDATAQRHRTILSSLGLPVSYDPDALPQLLEIMAGDKKTRAGVLRFVVLDGLAKPGRMVGPDPGLLVTAYAGVCAP

>236|CORE_REP|Org2_Gene1866#

MGSARAEMTKDAGEYLVTQAATRPTNDAGQDGGNNSDILVVARQQVLQRGEGLNQDQVLAVLQLPDDRLEELLALAHEVRMRWCGPEVEVEGIISLKTGGCPEDCHFCSQSGLFASPVRSAWLDIPSLVEAAKQTAKSGATEFCIVAAVRGPDERLMAQVAAGIEAIRNEVEINIACSLGMLTAEQVDQLAARGVHRYNHNLETARSFFANVVTTHTWEERWQTLSMVRDAGMEVCCGGILGMGETLQQRAEFAAELAELGPDEVPLNFLNPRPGTPFADLEVMPVGDALKAVAAFRLALPRTMLRFAGGREITLGDLGAKRGILGGINAVIVGNYLTTLGRPAEADLELLDELQMPLKALNASL

>237|CORE_REP|Org118_Gene2028#

MCAHEADPYRRCRSGDGVHLADPGADPVVPGTQGFGHQIREDGPPSHHTKRGTPSMGGVAILAGIWAGYLGAHLAGLAFDGEGIGASGLLVLGLATALGGVGFIDDLIKIRRSRNLGLNKTAKTVGQITSAVLFGVLVLQFRNAAGLTPGSADLSYVREIATVTLAPVLFVLFCVVIVSAWSNAVNFTDGLDGLAAGTMAMVTAAYVLITFWQYRNACVTAPGLGCYNVRDPLDLALIAAATAGACIGFLWWNAAPAKIFMGDTGSLALGGVIAGLSVTSRTEILAVVLGALFVAEITSVVLQILTFRTTGRRMFRMAPFHHHFELVGWAETTVIIRFWLLTAITCGLGVALFYGEWLAAVGA

>238|CORE_REP|Org3_Gene1855#

MANVQYSAVTQRYPGADAPTVDNLDLDIADGEFLVLVGPSGCGKSTTLRVLAGLEPIESGRISIGDVDVTHLPPRARDVAMVFQNYALYPNMTVAANMGFALRNAGMSRADTRRRVLEVADMLELTDLLDRKPAKLSGGQRQRVAMGRAIVRRPRVFCMDEPLSNLDAKLRVSTRSQISGLQRRLGTTTVYVTHDQVEAMTMGDRVAVLKDGVLQQVDTPRALYDDPVNTFVATFIGAPAMNLIDAAVAHGVVRAPDLAIPVPDPAAERVLVGVRPESWDVASIGTPGSLTVHVELVEELGFESFVYATPVDQRGWSSRAPRIVFRTDRRTAVRVGESLAIVPHSQEVRLFNSRTETRLR

>240|CORE_REP|Org1_Gene3523#

MIKPDDHTVNVVDIQVLKNAVLLACRAPSVHNSQPWRWVAESGSEHTTVHLFVNRHRTVPATDHSGRQAIISCGAVLDHLRIAMTAAHWQANITRFPQPNQPDQLATVEFSPIDHVTAGQRNRAQAILQRRTDRLPFDSPMYWHLFEPALRDAVDKDVAMLDVVSDDQRTRLVVASQLSEVLRRDDPYYHAELEWWTSPFVLAHGVPPDTLASDAERLRVDLGRDFPVRSYQNRRAELADDRSKVLVLSTPSDTRADALRCGEVLSTILLECTMAGMATCTLTHLIESSDSRDIVRGLTRQRGEPQALIRVGIAPPLAAVPAPTPRRPLDSVLQIRQTPEKGRNASDRNARETGWFSPP

>243|CORE_REP|Org29_Gene1580#

MLSLTLSEASCIASASRWRHIIPAGVVCALIAGIGVGCHGGPSDVVGRAGPDRAHTSITLVAYAVPEPGWSAVIPAFNASEQGRGVQVITSYGASADQSRGVADGKPADLVNFSVEPDIARLVKAGKVDKDWDADATKGIPFGSVVTFVVRAGNPKNIRDWDDLLRPGIEVITPSPLSSGSAKWNLLAPYAAKSDGGRNNQAGIDFVNTLVNEHVKLRPGSGREATDVFVQGSGDVLISYENEAIATERAGKPVQHVTPPQTFKIENPLAVVATSTHLGAATAFRNFQYTVQAQKLWAQAGFRPVDPAVAADFADLFPVPAKLWTIADLGGWGSVDPQLFDKATGSITKIYLRATG

>245|CORE_REP|Org118_Gene2226#

MTSRETRAADAAGARQADAQVRSSIDVPPDLVVGLLGSADENLRALERTLSADLHVRGNAVTLCGEPADVALAERVISELIAIVASGQSLTPEVVRHSVAMLVGTGNESPAEVLTLDILSRRGKTIRPKTLNQKRYVDAIDANTIVFGIGPAGTGKTYLAMAKAVHALQTKQVTRIILTRPAVEAGERLGFLPGTLSEKIDPYLRPLYDALYDMMDPELIPKLMSAGVIEVAPLAYMRGRTLNDAFIVLDEAQNTTAEQMKMFLTRLGFGSKVVVTGDVTQIDLPGGARSGLRAAVDILEDIDDIHIAELTSVDVVRHRLVSEIVGRLCAVRGARVGAESGGSAGVRRPRSPMMGA

>249|CORE_REP|Org2_Gene3633#

MLFVSVAPESVGVAAATLVGPPLIGNGADRPRHRTSRRDLVGQRPFSPNHRSGVLNATTAGAVQFNVLGPLELNLRGTKLPLGTPKQRAVLAMLLLSRNQVVAADALVQAIWEKSPPARARRTVHTYICNLRRTLSDAGVDSRNILVSEPPGYRLLIGDRQQCDLDRFVAAKESGLRASAKGYFSEAIRYLDSALQNWRGPVLGDLRSFMFVQMFSRALTEDELLVHTKLAEAAIACGRADVVIPKLERLVAMHPYRESLWKQLMLGYYVNEYQSAAIDAYHRLKSTLAEELGVEPAPTIRALYHKILRQLPMDDLVGRVTRGRVDLRGGNGAKVEELTESDKDLLPIGLA

>250|CORE_REP|Org59_Gene3530#

MAAARAAETESDNPLINDPFARIFVDAAGDGIWSMYTNRTLLAGATDLDPDLRAPIQQMIDFMAARTAFFDEYFLATADAGVRQVVILASGLDSRAWXLPWPDGTVVYELDQPKVLEFKSATLRQHGAQPASQLVNVPIDLRQDWPKALQKAGFDPSKPCAWLAEGLVRXLPARAQDLLFERIDALSRPGSWLASNVPGAGFLDPERMRRQRADMRRMRXAAAKXVETEISDVDDLWYAEQRTAVEPSGCVNVAGXCRRQRCPSCWLGMAAASLTXXKTQSRQTFSYPRSGPRADGDRGLFREHGRHVVLDHRYRVNRSEVLALFVLSELRPPGAVGPAQLVPYLAGFGQ

>253|CORE_REP|Org33_Gene2262#

MLISQRPTLSEDVLTDNRSQFVIEPLEPGFGYTLGNSLRRTLLSSIPGAAVTSIRIDGVLHEFTTVPGVKEDVTEIILNLKSLVVSSEEDEPVTMYLRKQGPGEVTAGDIVPPAGVTVHNPGMHIATLNDKGKLEVELVVERGRGYVPAVQNRASGAEIGRIPVDSIYSPVLKVTYKVDATRVEQRTDFDKLILDVETKNSISPRDALASAGKTLVELFGLARELNVEAEGIEIGPSPAEADHIASFALPIDDLDLTVRSYNCLKREGVHTVGELVARTESDLLDIRNFGQKSIDEVKIKLHQLGLSLKDSPPSFDPSEVAGYDVATGTWSTEGAYDEQDYAETEQL

>254|CORE_REP|Org61_Gene43#

MSAPWGPVAAGPSALVRSGQASTIEPFQREMTPPTPTPEAAHNPTMNVSRETSTEFDTPIGAAAERAMRVLHTTHEPLQRPGRRRVLTIANQKGGVGKTTTAVNIAAALAVQGLKTLVIDLDPQGNASTALGITDRQSGTPSSYEMLIGEVSLHTALRRSPHSERLFCIPATIDLAGAEIELVSMVARENRLRTALAALDNFDFDYVFVDCPPSLGLLTINALVAAPEVMIPIQCEYYALEGVSQLMRNIEMVKAHLNPQLEVTTVILTMYDGRTKLADQVADEVRQYFGSKVLRTVIPRSVKVSEAPGYSMTIIDYDPGSRGAMSYLDASRELAERDRPPSAKGRP

>258|CORE_REP|Org108_Gene1099#

MVLQELWFGVIAALFLGFFILEGFDFGVGMLMAPFAHVGMGDPETHRRTALNTIGPVWDGNEVWLITAGAAIFAAFPGWYATVFSALYLPLLAILFGMILRAVAIEWRGKIDDPKWRTGADFGIAAGSWLPALLWGVAFAILVRGLPVDANGHVALSIPDVLNAYTLLGGLATAGLFSLYGAVFIALKTSGPIRDDAYRFAVWLSLPVAGLVAGFGLWTQLAYGKDWTWLVLAVAGCAQAAATVLVWRRVSDGWAFMCTLIVVAAVVVLLFGALYPNLVPSTLNPQWSLTIHNASSTPYTLKIMTWVTAFFAPLTVAYQTWTYWVFRQRISAERIPPPTGLARRAP

>260|CORE_REP|Org119_Gene1356#

MSPSLRVQRVIAAIVILTQGGIAVTGAIVRVTASGLGCPTWPQCFPGSFTPVVVAEVPRVHQAVEFGNRMVTFAVVIAAALAVLVVTRARRRTEVLAYAWLMPVSTVVQAMIGGITVRTGLLWWTVAIHLLASMTMVWLAVLLYVKIGQPDDGVVHELVVSPLRALTALSALNLAAVLVTGTLVTAAGPHAGDRSPSRTVPRLKVEITTLVHMHSSLLVAYLALLIGLGFGLLAVGATRAILVRLAVLLALVATQAARRYHAILHRGTRRPGRHSRGRCRGGYGGHRRAMGVDGRTGPAPAAPTLTGRIARSHAGQDCGCLGPATASPATGPPIAIPATGDLGFHL

>263|CORE_REP|Org83_Gene269#

MPIATPEVYAEMLGQAKQNSYAFPAINCTSSETVNAAIKGFADAGSDGIIQFSTGGAEFGSGLGVKDMVTGAVALAEFTHVIAAKYPVNVALHTDHCPKDKLDSYVRPLLAISAQRVSKGGNPLFQSHMWDGSAVPIDENLAIAQELLKAAAAAKIILEIEIGVVGGEEDGVANEINEKLYTSPEDFEKTIEALGAGEHGKYLLAATFGNVHGVYKPGNVKLRPDILAQGQQVAAAKLGLPADAKPFDFVFHGGSGSLKSEIEEALRYGVVKMNVDTDTQYAFTRPIAGHMFTNYDGVLKVDGEVGVKKVYDPRSYLKKAEASMSQRVVQACNDLHCAGKSLTH

>264|CORE_REP|Org107_Gene3207#

MTTVLGIETSCDETGVGIARLDPDGTVTLLADEVASSVDEHVRFGGVVPEIASRAHLEALGPAMRRALAAAGLKQPDIVAATIGPGLAGALLVGVAAAKAYSAAWGVPFYAVNHLGGHLAADVYEHGPLPECVALLVSGGHTHLLHVRSLGEPIIELGSTVDDAAGEAYDKVARLLGLGYPGGKALDDLARTGDRDAIVFPRGMSGPADDRYAFSFSGLKTAVARYVESHAADPGFRTADIAAGFQEAVADVLTMKAVRAATALGVSTLLIAGGVAANSRLRELATQRCGEAGRTLRIPSPRLCTDNGAMIAAFAAQLVAAGAPPSPLDVPSDPGLPVMQGQVR

>265|CORE_REP|Org124_Gene42#

MTQPSRRKGGLGRGLAALIPTGPADGESGPPTLGPRMGSATADVVIGGPVPDTSVMGAIYREIPPSAIEANPRQPRQVFDEEALAELVHSIREFGLLQPIVVRSLAGSQTGVRYQIVMGERRWRAAQEAGLATIPAIVRETGDDNLLRDALLENIHRVQLNPLEEAAAYQQLLDEFGVTHDELAARIGRSRPLITNMIRLLKLPIPVQRRVAAGVLSAGHARALLSLEAGPEAQEELASRIVAEGLSVRATEETVTLANHEANRQAHHSDATTPAPPRRKPIQMPGLQDVAERLSTTFDTRVTVSLGKRKGKIVVEFGSVDDLARIVGLMTTDGRDKGLHRDAL

>270|CORE_REP|Org59_Gene3446#

MVATTSSGGSSVGWPSRLSGVRLHLVTGKGGTGKSTIAAALALTLAAGGRKVLLVEVEGRQGIAQLFDVPPLPYQELKIATAERGGQVNALAIDIEAAFLEYGLDMFYNLGIAGRAMRRIGAVEFATTIAPGLRDVLLTGKIKETVVRLDKNKLPVYDAIVVDAPPTGRIARFLDVTKAVSDLAKGGPVHAQSEGVVKLLHSNQTAIHLVTLLEALPVQETLEAIEELAQMELPIGSVIVNRNIPAHLEPQDLAKAAEGEVDADSVRAGLLTAGVKLPDADFAGLLTETIQHATRITARAEIAQQLDALQVPRLELPTVSDGVDLGSLYELSESLAQQGVR

>271|CORE_REP|Org14_Gene116#

MTFFEQVRRLRSAATTLPRRLAIAAMGAVLVYGLVGTFGGPATAGAFSRPGLPVEYLQVPSASMGRDIKVQFQGGGPHAVYLLDGLRAQDDYNGWDINTPAFEEYYQSGLSVIMPVGGQSSFYTDWYQPSQSNGQNYTYKWETFLTREMPAWLQANKGVSPTGNAAVGLSMSGGSALILAAYYPQQFPYAASLSGFLNPSEGWWPTLIGLAMNDSGGYNANSMWGPSSDPAWKRNDPMVQIPRLVANNTRIWVYCGNGTPSDLGGDNIPAKFLEGLTLRTNQTFRDTYAADGGRNGVFNFPPNGTHSWPYWNEQLVAMKADIQHVLNGATPPAAPAAPAA

>272|CORE_REP|Org36_Gene2044#

MELGSLIRATNLWGYTDLMRELGADPLPFLRRFDIPPGIEHQEDAFMSLAGFVRMLEASAAELDCPDFGLRLARWQGLGILGPVAVIARNAATLFGGLEAIGRYLYVHSPALTLTVSSTTARSNVRFGYEVTEPGIPYPLQGYELSMANAARMIRLLGGPQARARVFSFRHAQLGTDAAYREALGCTVRFGRTWCGFEVDHRLAGRPIDHADPETKRIATKYLESQYLPSDATLSERVVGLARRLLPTGQCSAEAIADQLDMHPRTLQRRLAAEGLRCHDLIERERRAQAARYLAQPGLYLSQIAVLLGYSEQSALNRSCRRWFGMTPRQYRAYGGVSGR

>273|CORE_REP|Org59_Gene787#

MSAKLTDLQLLHELEPVVEKYLNRHLSMHKPWNPHDYIPWSDGKNYYALGGQDWDPDQSKLSDVAQVAMVQNLVTEDNLPSYHREIAMNMGMDGAWGQWVNRWTAEENRHGIALRDYLVVTRSVDPVELEKLRLEVVNRGFSPGQNHQGHYFAESLTDSVLYVSFQELATRISHRNTGKACNDPVADQLMAKISADENLHMIFYRDVSEAAFDLVPNQAMKSLHLILSHFQMPGFQVPEFRRKAVVIAVGGVYDPRIHLDEVVMPVLKKWRIFEREDFTGEGAKLRDELALVIKDLELACDKFEVSKQRQLDREARTGKKVSAHELHKTAGKLAMSRR

>276|CORE_REP|Org142_Gene2431#

MAKNSRRKRHRILAWIAAGAMASVVALVIVAVVIMLRGAESPPSAVPPGVLPPGPTPAHPHKPRPAFQDASCPDVQMISVPGTWESSPQQNPLNPVQFPKALLLKVTGPIAQQFAPARVQTYTVAYTAQFHNPLTTDNQMSYNDSRAEGTRAMVAAMTDMNNRCPLTSYVLIGFSQGAVIAGDVASDIGNGRGPVDEDLVLGVTLIADGRRQQGVGNQVPPSPRGEGAEITLHEVPVLSGLGLTMTGPRPGGFGALDGRTNEICAQGDLICAAPAQAFSPANLPTTLNTLAGGAGQPVHAMYATPEFWNSDGEPATEWTLNWAHQLIENAPHPKHR

>278|CORE_REP|Org38_Gene412#

MRTPATVVAGVDLGDAVFAAAVRAGVARVEQLMDTELRQADEVMSDSLLHLFNAGGKRFRPLFTVLSAQIGPQPDAAAVTVAGAVIEMIHLATLYHDDVMDEAQVRRGAPSANAQWGNNVAILAGDYLLATASRLVARLGPEAVRIIADTFAQLVTGQMRETRGTSENVDSIEQYLKVVQEKTGSLIGAAGRLGGMFSGATDEQVERLSRLGGVVGTAFQIADDIIDIDSESDESGKLPGTDVREGVHTLPMLYALRESGPDCARLRALLNGPVDDDAEVREALTLLRASPGMARAKDVLAQYAAQARHELALLPDVPGRRALAALVDYTVSRHG

>279|CORE_REP|Org112_Gene394#

MTEIATTSGARSVGLLSVGAYRPERVVTNDEICQHIDSSDEWIYTRTGIKTRRFAADDESAASMATEACRRALSNAGLSAADIDGVIVTTNTHFLQTPPAAPMVAASLGAKGILGFDLSAGCAGFGYALGAAADMIRGGGAATMLVVGTEKLSPTIDMYDRGNCFIFADGAAAVVVGETPFQGIGPTVAGSDGEQADAIRQDIDWITFAQNPSGPRPFVRLEGPAVFRWAAFKMGDVGRRAMDAAGVRPDQIDVFVPHQANSRINELLVKNLQLRPDAVVANDIEHTGNTSAASIPLAMAELLTTGAAKPGDLALLIGYGAGLSYAAQVVRMPKG

>280|CORE_REP|Org59_Gene1209#

MAITINMVNPTGFIRYEDVEQEAMTSDVTVGPAPGQYQLSHLRLLEAEAIHVIREVAAEFERPVLLFSGGKDSIVMLHLALKAFRPGRLPFPVMHVDTGHNFDEVIAXRDELVAAAGVRLVVASVQDDIDAGRVVETIPSRNPIQTVTLLRAIRENQFDAAFGGARRDEEKARAKERVFSFRDEFGQWDPKAQRPELWNLYNGRHHKGEHIRVFPLSNWTEFDIWSYIGAEQVRLPSIYFAHRRKVFQRDGMLLAVHRPHAXRGAXEPVFEATVRFRTVGDVTCTGCVESSASTVAEVIAETAVARLTERGATRADDRISEAGMEDRKRQGYF

>281|CORE_REP|Org119_Gene2767#

MAGAKHAGRIVAITTAAAVILAACSSGSKGGAGSGHAGKARSAVTTTDADWKPVADALGRSGKLGDNNTAYRINLPRNDLHITSYGVDIKPGLSLGGYAAFARYDNNETLLMGDLVITEEELPKVTDALQAHGIAQTALHKHLLQQDPPVWWTHIHGMGDAARLAQGLKAALDATTIGPPTPPPARQPPVDIDVAGVDQALGRKGTQDGGLLKYSIPRKDTIIEDGHVLPAVSLNLTTVINFQPVGRGRAAINGDFILIAPEVQEVIRAMRAGNITIVELHNHGLTEEPRLFYMHYWAVDDAVTLARALRPAMECHQPAVVIIPMQPHKGWCG

>283|CORE_REP|Org59_Gene2384#

MGGLTISDLVVEYSSGGXAVRPIDGXKPRRGAGVAGDLAWAQRLREDDPLVLPRRXXCARSPAQSSLTMSTSSNLXEGAALAKYRRDKXGIVFQAFNLVSSLTALENVMVPLRAAGVSRAAARKRAEDLLIRVNLGERMKHRPGDMSGGQQQRVAVARAIALDPQLILADEPTAHLDFIQVEEVLRLIRSLAQGDRVVVVATHDSRMLPLADRVLELMPAQVSPNQPPETVHVKAGEVLFEQSTMGDLIYVVSEGEFEIVRELADGGEELVKXAAPGDYFGEIGVLFXLPRSATVRARSDATAVGYTAQAFRERLGXXRXXDLIEHRELASE

>284|CORE_REP|Org18_Gene2243#

MRLLVTGGAGFIGTNFVHSAVREHPDDAVTVLDALTYAGRRESLADVEDAIRLVQGDITDAELVSQLVAESDAVVHFAAESHVDNALDNPEPFLHTNVIGTFTILEAVRRHGVRLHHISTDEVYGDLELDDRARFTESTPYNPSSPYSATKAGADMLVRAWVRSYGVRATISNCSNNYGPYQHVEKFIPRQITNVLTGRRPKLYGAGANVRDWIHVDDHNSAVRRILDRGRIGRTYLISSEGERDNLTVLRTLLRLMDRDPDDFDHVTDRVGHDLRYAIDPSTLYDELCWAPKHTDFEEGLRTTIDWYRDNESWWRPLKDATEARYQERGQ

>285|CORE_REP|Org146_Gene224#

MNPIPSWPGRGRVTLVLLAVVPVALAYPWQSTRDYVLLGVAAAVVIGLFGFWRGLYFTTIARRGLAILRRRRRIAEPATCTRTTVLVWVGPPASDTNVLPLTLIARYLDRYGIRADTIRITSRVTASGDCRTWVGLTVVADDNLAALQARSARIPLQETAQVAARRLADHLREIGWEAGTAAPDEIPALVAADSRETWRGMRHTDSDYVAAYRVSADAELPDTLPAIRSRPAQETWIALEIAYAAGSSTRYTVAAACALRTDWRPGGTAPVAGLLPQHGNHVPALTALDPRSTRRLDGHTDAPADLLTRLHWPTPTAGAHRAPLTNAVSRT

>287|CORE_REP|Org59_Gene3520#

MPTLDCDSDCRGATGVRAWAGTHRRFHHAGRRSGGXXGXXWXXXRSXXGXHGARSDGFDAITDLNQTLTRAAATEALIVLAVPMPALPGMLAHIRKSAPGCPLTDVTSVKCAVLDEVTAXGLQARYVGGHPMTGTAHSGWTAGHGGLFNRAPWVVSVDDHVDPTVWSMVMTLALDCGAMVVPAKSDEHDAAAAAVSHLPHLLAEALAVTAAEVPLAFALAAGSFRDATRVAATAPDLVRAMCEANTGQLAPAADRIIDLLSRARDSLQSHGSIADLADAGHAARTRYDSFPRSDIVTVVIGADKWREQLAAAGRAGGVITSALPSLDSPQ

>289|CORE_REP|Org57_Gene3234#

MAGWFAHTLRPAMLAAGRSDRLGRIVERSPLTRGVVRRFVPGDTLDDVVDIVTALRDSGRYLSIDYLGENVTDADDAAAAVRAYLGLLDVLGRRGDIACDGVRPLEVSLKLSALGQALDRDGQKIALDNARAICERAERVGAWVTVDAEDHTTTDSTLSISGDLRVDFPWLGTVVQAYLRRTLADCAELAAVGARVRLCKGAYDEPASVAYRDAAQVTDSYLRCLRVLTAGRGYPMVATHDPVIIAAVPGITRESGRSQGDFEYQMLYGVRDDEQRRLTGAGNHVRVYVPFGTRWYGYFLRRLAERPANLAFFLRALTDRRRARGCAER

>290|CORE_REP|Org30_Gene261#

MPGARELTLRVERGALFRRRWAASAASSARAAIRRDPRRCALGTRPRWVSFLVIVLVIMNVVTAHPKYPNDPLALVLIELRHPRTEPPVPSAISILKEELARWTPILEQEEVRQVNLETGEHTAHSQKKLVARDRRTAITFRPDAMTLEVTDYPGWEEFRSIVHAMVTARQDVAPVDGCIRIGLRYINEIRASLAEPSGWAYWVAESLLGPGTQLADLKLTTTAQRHVIQCEGPEPGDSLTLRYAGARGAVIQSTPFLQRLKEPPAEGDFFLIDIDSAWSDPCKGIPALDAHLVDEVAERLHTPIGPLFESLITSELRTKVLQQPGQE

>292|CORE_REP|Org59_Gene1366#

MLAAQFSLELKLLLRNGEQLLLTMFIPITLLVGLTLLPMGSFGHNRAATFVPVIMALAVISTAFTGQAIAVAFDRRYGALKRLGATPLPVWGIIAGKSLAVVAVVFLQAIILGAIGFALGWRPALTALTLGAGIIALGTXGFAALGLLLGGTLRAEIVLAVANLMWFVFAGFGALTLESNVIPTAFKWVARVTPSGALTEALSHGHDRVGGLVRDRRPSGVGRAGRTGRTALVPVHLNRPPGFATLQPRTRHADASVRGCSFDTGPPVLQRVVFGALRSGGVXQPACPAGHRRNRHPHPGRHRRHRGNRPGYRLRPGVSDLAAVFSG

>294|CORE_REP|Org16_Gene407#

MIPVLPPLEALLDRLYVVALPMRVRFRGITTREVALIEGPAGWGEFGAFVEYQSAQACAWLASAIETAYCAPPPVRRDRVPINATVPAVAAAQVGEVLARFPGARTAKVKVAEPGQSLADDIERVNAVRELVPMVRVDANGGWGVAEAVAAAAALTADGPLEYLEQPCATVAELAELRRRVDVPIAADESIRKAEDPLAVVRAQAADIAVLKVAPLGGISALLDIAARIAVPVVVSSALDSAVGIAAGLTAAAALPELDHACGLGTGGLFEEDVAEPAAPVDGFLAVARTTPDPARLQALGAPPQRRQWWIDRVKACYSLLVPSFG

>299|CORE_REP|Org28_Gene921#

MTSAPATMRWGNLPLAGESGTMTLRQAIDLAAALLAEAGVDSARCDAEQLAAHLAGTDRGRLPLFEPPGDEFFGRYRDIVTARARRVPLQHLIGTVSFGPVVLHVGPGVFVPRPETEAILAWATAQSLPARPLIVDACTGSGALAVALAQHRANLGLKARIIGIDDSDCALDYARRNAAGTPVELVRADVTTPCLLPELDGQVDLMVSNPPYIPDAAVLEPEVAQHDPHHALFGGPDGMTVISAVVGLAGRWLRPGGLFAVEHDDTTSSSTVDLVSSTKLFVDVQARKDLAGRPRFVTAMRWGHLPLAGENGAIDPRQRRCRAKR

>300|CORE_REP|Org82_Gene638#

MRTEDDSWDVTTSVGSTGLLVAAARALETQKADPLAIDPYAEVFCRAAGGEWADVLDGKLPDHYLTTGDFGEHFVNFQGARTRYFDEYFSRATAAGMKQVVILAAGLDSRAFRLQWPIGTTIFELDRPQVLDFKNAVLADYHIRPRAQRRSVAVDLRDEWQIALCNNGFDANRPSAWIAEGLLVYLSAEAQQRLFIGIDTLASPGSHVAVEEATPLDPCEFAAKLERERAANAQGDPRRFFQMVYNERWARATEWFDERGWRATATPLAEYLRRVGRAVPEADTEAAPMVTAITFVSAVRTGLVADPARTSPSSTSIGFKRFEAD

>303|CORE_REP|Org88_Gene387#

MNTLHVNVGLARYSDWAFTSAVVALVVALLLLAFEFAQVRGRGLAPLAVPAGSVATDSATPGIVADQRHRPFDERVGRGGLAVAYLGIGLLLACVVLRGLATQRVPWGNMYEFINLTCLSGLIAGAVVLRRARYRPLWVFLLVPVLILLTVSGRWLYANAAPVMPALQSYWLPIHVSVVSLGSGVFLVAGVASILFLVRTSRLGEPTGEGALAGMVRRLPDAQTLDGIAYRTTIFAFPVFGFGVIFGAIWAEEAWGRYWGWDPKETVSFVAWVVYAAYLHARSTAGWRDRKAAWINVAGFVAMVFNLFFVNLVTVGLHSYAGVG

>305|CORE_REP|Org89_Gene3641#

MALVSTARVDLVCEGGGVRGIGLVGAVDALADAGYRFPRVAGSSAGAIVASLVAALQTAGEPVTRLAEMMRSIDYPKFLDRNLIGHVPLIGGGLSLLLSDGVYRGAYLEQLLGGLLADLGVHTFGDLRTGEAPEQFAWSLVVTASDLSRRRLVRIPWDLDSYGIHPDDFSVARAVHASSAIPFVFEPVRVRGATWVDGGLLSNFPVALFDRTDAEPRWPTFGIRLSARPGTPPTRPVQGPVSLGIAAIETLVSNQDNAYIDDPCTVRRTIFVPAHDVSPIDFDITAEQREALYQRGFQAGQKFLANWNYADYLADCGGPFTPSL

>306|CORE_REP|Org65_Gene2020#

MTGNAKLIDRVSAINWNRLQDEKDAEVWDRLTGNFWLPEKVPVSNDIPSWGTLTAGEKQLTMRVFTGLTMLDTIQGTVGAVSLIPDALTPHEEAVLTNIAFMESVHAKSYSQIFSTLCSTAEIDDAFRWSEENRNLQRKAEIVLQYYRGDEPLKRKVASTLLESFLFYSGFYLPMYWSSRAKLTNTADMIRLIIRDEAVHGYYIGYKFQRGLALVDDVTRAELKDYTYELLFELYDNEVEYTQDLYDEVGLTEDVKKFLRYNANKALMNLGYEALFPRDETDVNPAILSALSPNADENHDFFSGSGSSYVIGKAVVTEDDDWDF

>308|CORE_REP|Org59_Gene3345#

MALDLTAYFDRINYRGATDPTLDVLQDLVTVHSRTIPFENLDPLLGVPVDDLSPQALADKLVLRRRGGYCFEHNGLMGYVLAELGYRVRRFAARVVWKLAPDAPLPPQTHTLLGVTFPGSGGCYLVDVGFGGPNNPTSPLRLETGAVQPTTHEPYRLXDXXXGFVXQAMVRDTWQTLYEFTTQTRPQIDLKVASWYASTHPASKFVTGLTAAVITDDARWNLSGRDLAVHRAGGTEKIRLADAAAVVDTLSERFGINVADIGERGALETRIDELXAXXPGXDAPXGFFDVAAWPGKRYPNKRTPPCGVEIDSGCDRNTGVGQQL

>310|CORE_REP|Org95_Gene3181#

MPGNSRRRGAVRKSGTKKGAGVGSGGQRRRGLEGRGPTPPAHLRPHHPAAKRARAQPRRPVKRADETETVLGRNPVLECLRAGVPATALYVALGTEADERLTECVARAADSGIAIVELLRADLDRMTANHLHQGIALQVPPYNYAHPDDLLAAALDQPPALLVALDNLSDPRNLGAIVRSVAAFGGHGVLIPQRRSASVTAVAWRTSAGAAARIPVARATNLTRTLKGWADRGVRVIGLDAGGGTALDDVDGTDSLVVVVGSEGKGLSRLVRQNCDEVVSIPMAAQAESLNASVAAGVVLAEIARQRRRPREPREQTQNRMI

>311|CORE_REP|Org118_Gene1438#

MNAHTSVGPLDRAARVYIAGHRGLVGSALLRTFAGAGFTNLLVRSRAELDLTDRAATFDFVLESRPQVVIDAAARVGGILANDTYPADFLSENLQIQVNLLDAAVAARVPRLLFLGSSCIYPKLAPQPIPESALLTGPLEPTNDAYAIAKIAGILAVQAVRRQHGLPWISAMPTNLYGPGDNFSPSGSHLLPALIRRYDEAKASGAPNVTNWGTGTPRRELLHVDDLASACLYLLEHFDGPTHVNVGTGIDHTIGEIAEMVASAVGYSGETRWDPSKPDGTPRKLLDVSVLREAGWRPSIALRDGIEATVAWYREHAGTVRQ

>312|CORE_REP|Org59_Gene3549#

MAAVRLGXSWXPXATLKACGSGFGCAAWAVGRSLASLXDLHDIHVMLKLASRHSRGHWPASEAIWSTGSAATSCGCTWAGRXSSSGTAARCWGRSGSPSPPERPPSRWAACIPSCFGSELSEHLPYVTLGLIVWNLINAAILDGAEVFVANEGLIKQLPAPLSVHVYRLVWRXXIFFAXNIVIYFVIAIIFPKPWSWADLSFLPALALIFLNCVWVSLCFGILATRYRDIGPLLFSVVQLLFFMTPIIWNDETLRRQGXGRWSSIVELNPLLHYLDIVRAPLLGAHQELRHWLXVLVXXVVGWMLAAFAMRQXRARXPYWV

>315|CORE_REP|Org128_Gene2716#

MPAGLPGQASVAVRLSCDVPPDARHHEPRPGMTDHPDTGNGIGLTGRPPRAIPDPAPRSSHGPAKVIAMCNQKGGVGKTTSTINLGAALGEYGRRVLLVDMDPQGALSAGLGVPHYELDKTIHNVLVEPRVSIDDVLIHSRVKNMDLVPSNIDLSAAEIQLVNEVGREQTLARALYPVLDRYDYVLIDCQPSLGLLTVNGLACTDGVIIPTECEFFSLRGLALLTDTVDKVRDRLNPKLDISGILITRYDPRTVNSREVMARVVERFGDLVFDTVITRTVRFPETSVAGEPITTWAPKSAGALAYRALARELIDRFGM

>316|CORE_REP|Org49_Gene727#

MCCTSGCALTIRLLGRTEIRRLAKELDFRPRKSLGQNFVHDANTVRRVVAASGVSRSDLVLEVGPGLGSLTLALLDRGATVTAVEIDPLLASRLQQTVAEHSHSEVHRLTVVNRDVLALRREDLAAAPTAVVANLPYNVAVPALLHLLVEFPSIRVVTVMVQAEVAERLAAEPGSKEYGVPSVKLRFFGRVRRCGMVSPTVFWPIPRVYSGLVRIDRYETSPWPTDDAFRRRVFELVDIAFAQRRKTSRNAFVQWAGSGSESANRLLAASIDPARRGETLSIDDFVRLLRRSGGSDEATSTGRDARAPDISGHASAS

>319|CORE_REP|Org70_Gene916#

MVTQALLPSGLVASAVVAASSANLGPGFDSVGLALSLYDEIIVETTDSGLTVTVDGEGGDQVPLGPEHLVVRAVQHGLQAAGVSAAGLAVRCRNAIPHSRGLGSSAAAVVGGLAAVNGLVVQTDSSPSSDAELIQLASEFEGHPDNAAAAVLGGAVVSWTDHSGDRPNYSAVSLRLHPDIRLFTAIPEQRSSTAETRVLLPAQVSHDDARFNVSRAALLVVALTERPDLLMAATEDLLHQPQRAAAMTASAEYLRLLRRHNVAAALSGAGPSLIALSTDSELPTDAVEFGAAKGFAVTELTVGEAVRWSPTVRVPG

>325|CORE_REP|Org59_Gene286#

MDATPNAVELTVDNAWFIAETIGAGTFPWVLAITMPYSDAAQRGAFVDRQRDELTRMGLLSPQGVINPAVADWIKVVCFPDRWLDLRYVGPASADGACELLRGIVALRTGTGKTSNKTGNGVVALRNAQLVTFTAMDIDDPRALVPILGXGLAHRPPARFDEFSLPTRVGARADERLRSGVPLGEVVDYLGIPASARPVVESVFSGPRSYVEIVAGCNRSXERRXHHHRGRPKHRRHLGGPGVGESVAGIRRRVGLHLQPWDTVCDRRRDPNTDRVLARRAMVPGTAGVAGLLHPILVIRNQKVSTMSQERSR

>330|CORE_REP|Org12_Gene3383#

MGKGSMTAHATPNEPDYPPPPGGPPPPADIGRLLLRCHDRPGIIAAVSTFLARAGANIISLDQHSTAPEGGTFLQRAIFHLPGLTAAVDELQRDFGSTVADKFGIDYRFAEAAKPKRVAIMASTEDHCLLDLLWRNRRGELELSVVMVIANHPDLAAHVRPFGVPFIHIPATRDTRTEAEQRQLQLLSGNVDLVVLARYMQILSPGFLEAIGCPLINIHHSFLPAFTGAAPYQRARERGVKLIGATAHYVTEVLDEGPIIEQDVVRVDHTHTVDDLVRVGADVERAVLSRAVLWHCQDRVIVHHNQTIVF

>332|CORE_REP|Org119_Gene1433#

MADRSMPVPDGLAGMRVDTGLARLLGLSRTAAAALAEEGAVELNGVPAGKSDRLVSGALLQVRLPEAPAPLQNTPIDIEGMTILYSDDDIVAVDKPAAVAAHASVGWTGPTVLGGLAAAGYRITTSGVHERQGIVHRLDVGTSGVMVVAISGWGAYTVLKRAFKYRTVDKRYHALVQGHPDPSSGTIDAPIGRHRGHEWKFAITKNGRHSLTHYDTLEAFVAASLLDVHLETGRTHQIRVHFAALHHPCCGDLVYGADPKLAKRLGLDRQWLHARSLAFAHPADGRRVEIVSPYPADLQHALKILRGEG

>333|CORE_REP|Org142_Gene1372#

MRAAGLLKRLNPRNRRSRVNPDATMSLVDHLTELRTRLLISLAAILVTTIFGFVWYSHSIFGLDSLGEWLRHPYCALPQSARADISADGECRLLATAPFDQFMLRLKVGMAAGIVLACPVWFYQLWAFITPGLYQRERRFAVAFVIPAAVLFVAGAVLAYLVLSKALGFLLTVGSDVQVTALSGDRYFGFLLNLLVVFGVSFEFPLLIVMLNLAGLLTYERLKSWRRGLIFAMFVFAAIFTPGSDPFSMTALGAALTVLLELAIQIARVHDKRKAKREAAIPDDEASVIDPPSPVPAPSVIGSHDDVT

>336|CORE_REP|Org113_Gene2614#

MNVRGRVAPRRVTGRAMSTLLAYLALTKPRVIELLLVTAIPAMLLADRGAIHPLLMLNTLVGGMMAAAGANTLNCVADADIDKVMKRTARRPLAREAVPTRNALALGLTLTVISFFWLWCATNLLAGVLALVTVAFYVFVYTLWLKRRTSQNVVWGGAAGCMPVMIGWSAITGTIAWPALAMFAIIFFWTPPHTWALAMRYKQDYQVAGVPMLPAVATERQVTKQILIYTWLTVAATLVLALATSWLYGAVALVAGGWFLTMAHQLYAGVRAGEPVRPLRLFLQSNNYLAVVFCALAVDSVIALPTLH

>339|CORE_REP|Org30_Gene950#

MDRCCQRATAFACALRPTKLIDYEEMFRGAMQARAMVANPDQWADSDRDQVNTRHYLSTSMRVALDRGEFFLVYQPIIRLADNRIIGAEALLRWEHPTLGTLLPGRFIDRAENNGLMVPLTAFVLEQACRHVRSWRDHSTDPQPFVSVNVSASTICDPGFLVLVEGVLGETGLPAHALQLELAEDARLSRDEKAVTRLQELSALGVGIAIDDFGIGFSSLAYLPRLPVDVVKLGGKFIECLDGDIQARLANEQITRAMIDLGDKLGITVTAKLVETPSQAARLRAFGCKAAQGWHFAKALPVDFFRE

>343|CORE_REP|Org46_Gene713#

MSRPEVLTPFTAIVPAAGLGTRFLPATKTVPKELLPVVDTPGIELVAAEAAAAGAERLVIVTSEGKDGVVAHFVEDLVLEGTLEARGKIAMLAKVRRAPALIKVESVVQAEPLGLGHAIGCVEPTLSPDEDAVAVLLPDDLVLPTGVLETMSKVRASRGGTVLCAIEVAREEISAYGVFDVEPVPDGDYTDDPNVLKVRGMVEKPKAETAPSRYAAAGRYVLDRAIFDALRRIDRGAGGEVQLTDAIALLIAEGHPVHVVVHQGSRHDLGNPGGYLKAAVDFALDRDDYGPDLRRWLVARLGLTEQ

>346|CORE_REP|Org13_Gene986#

MAATLRELRGRIRSAGSIKKITKAQELIATSRIARAQARLESARPYAFEITRMLTTLAAEAALDHPLLVERPEPKRAGVLVVSSDRGLCGAYNANIFRRSEELFSLLREAGKQPVLYVVGRKAQNYYSFRNWNITESWMGFSEQPTYENAAEIASTLVDAFLLGTDNGEDQRSDSGEGVDELHIVYTEFKSMLSQSAEAHRIAPMVVEYVEEDIGPRTLYSFEPDATMLFESLLPRYLTTRVYAALLESAASELASRQRAMKSATDNADDLIKALTLMANRERQAQITQEISEIVGGANALAEAR

>349|CORE_REP|Org98_Gene3124#

MAGRSERLVITGAGGQLGSHLTAQAAREGRDMLALTSSQWDITDPAAAERIIRHGDVVINCAAYTDVDGAESNEAVAYAVNATGPQHLARACARVGARLIHVSTDYVFDGDFGGAEPRPYEPTDETAPQGVYARSKLAGEQAVLAAFPEAAVVRTAWVYTGGTGKDFVAVMRRLAAGHGRVDVVDDQTGSPTYVADLAEALLALADAGVRGRVLHAANEGVVSRFGQARAVFEECGADPQRVRPVSSAQFPRPAPRSSYSALSSRQWALAGLTPLRHWRSALATALAAPANSTSIDRRLPSTRD

>353|CORE_REP|Org119_Gene3514#

MSEDVVTQPPANLVAGVVKAIRPRQWVKNVLVLAAPLAALGGGVRYDYVEVLSKVSMAFVVFSLAASAVYLVNDVRDVEADREHPTKRFRPIAAGVVPEWLAYTVAVVLGVTSLAGAWMLTPNLALVMVVYLAMQLAYCFGLKHQAVVDICVVSSAYFVSRAIAGGVATKIPLSKWFLLIMAFGSLFMVAGKRYAELHLAERTGAAIRKSLESYTSTYLRFVWTLSATAVVLCYGLWAFERDGYSGSWFAVSMIPFTIAILRYAVDVDGGLAGEPEDIALRDRVLQLLALAWIATVGAAVAFG

>354|CORE_REP|Org6_Gene2975#

MSGNEVHPDLRRIAVVTPRQLVGPRTLPVMRALIVVAGLRMSRTPPDIEVLTLESGVGVRLYRPAGSNEPAPALLWIHAGGYVMGTAQQDDRLCLRFSSRLGITVASVDYRLAPENPYPAALGDCYSALTWLASLPAVDPARVAIGGASAGGGLAAALALLARDRGGITPAFQLLVYPMLDDRPSIAPANPHYRLWNGRANRFGWRAYLGDADARVAVPGRRDDLGGLAPAWIGVGTHDLLHDEDLAYAERLTAAGVPCQVEVVEGAFHGFDRVAPNVGVSQRFFTSQCNSLRAALALSNRT

>356|CORE_REP|Org68_Gene2612#

MMNHARGVENRSEGGGIDVVLVTGLSGAGRGTAAKVLEDLGWYVADNLPPQLITRMVDFGLAAGSRITQLAVVMDVRSRGFTGDLDSVRNELATRAITPRVVFMEASDDTLVRRYEQNRRSHPLQGEQTLAEGIAAERRMLAPVRATADLIIDTSTLSVGGLRDSIERAFGGDGGATTSVTVESFGFKYGLPMDADMVMDVRFLPNPHWVDELRPLTGQHPAVRDYVLHRPGAAEFLESYHRLLSLVVDGYRREGKRYMTIAIGCTGGKHRSVAIAEALMGLLRSDQQLSVRALHRDLGRE

>357|CORE_REP|Org149_Gene3036#

MTRMAEKPISPTKTRTRFEDIQAHYDVSDDFFALFQDPTRTYSCAYFEPPELTLEEAQYAKVDLNLDKLDLKPGMTLLDIGCGWGTTMRRAVERFDVNVIGLTLSKNQHARCEQVLASIDTNRSRQVLLQGWEDFAEPVDRIVSIEAFEHFGHENYDDFFKRCFNIMPADGRMTVQSSVSYHPYEMAARGKKLSFETARFIKFIVTEIFPGGRLPSTEMMVEHGEKAGFTVPEPLSLRPHYIKTLRIWGDTLQSNKDKAIEVTSEEVYNRYMKYLRGCEHYFTDEMLDCSLVTYLKPGAAA

>358|CORE_REP|Org4_Gene3919#

MGWRDAPALSDYQHVASGKVREIYRVDDEHLLLVASDRISAYDYVLDSTIPDKGRVLTAMSAFFFGLVDAPNHLAGPPDDPRIPDEVLGRALVVRRLEMLPVECVARGYLTGSGLLDYQATGKVCGIALPPGLVEASRFATPLFTPATKAALGDHDENISFDRVVEMVGALRANQLRDRTLQTYVQAADHALTRGIIIADTKFEFGIDRHGNLLLADEIFTPDSSRYWPADDYRAGVVQTSFDKQFVRSWLTGSESGWDRGSDRPPPPLPEHIVEATRARYINAYERISELKFDDWIGPGA

>360|CORE_REP|Org13_Gene2235#

MNVLSLGSSSGVVWGRVPITAPAGAATGVTSRADAHSQMRRYAQTGPTAKLSSAPMTTMWGAPLHRRWRGSRLRDPRQAKFLTLASLKWVLANRAYTPWYLVRYWRLLRFKLANPHIITRGMVFLGKGVEIHATPELAQLEIGRWVHIGDKNTIRAHEGSLRFGDKVVLGRDNVINTYLDIEIGDSVLMADWCYICDFDHRMDDITLPIKDQGIIKSPVRIGPDTWIGVKVSVLRGTTIGRGCVLGSHAVVRGAIPDYSIAVGAPAKVVKNRQLSWEASAAQRAELAAALADIERKKAAR

>364|CORE_REP|Org45_Gene1752#

MSSGNSSLGIIVGIDDSPAAQVAVRWAARDAELRKIPLTLVHAVSPEVATWLEVPLPPGVLRWQQDHGRHLIDDALKVVEQASLRAGPPTVHSEIVPAAAVPTLVDMSKDAVLMVVGCLGSGRWPGRLLGSVSSGLLRHAHCPVVIIHDEDSVMPHPQQAPVLVGVDGSSASELATAIAFDEASRRNVDLVALHAWSDVDVSEWPGIDWPATQSMAEQVLAERLAGWQERYPNVAITRVVVRDQPARQLVQRSEEAQLVVVGSRGRGGYAGMLVGSVGETVAQLARTPVIVARESLT

>366|CORE_REP|Org89_Gene2902#

MARNPAAQTAFGPMVLAAVEQNEPPGRRLVDDDLADLFLPRPLRWLAGATRSAVLRRLLISASEWSGRGLWANLACRKRFIGDKLDEALGDIDAVVILGAGLDTRAYRLTRRVRMPVFEVDLPVNIARKAKTVRRVLGELPLSVRLVALDFEHDDLLTALAEHGYRTEYRVFFVCEGVTQYLTERAVRRTLEGLRAAAPGSRMVFTYVRRDFIDGTNRYGTRTLYHTVRQRRQLWHFGLDPEEVAGFLADYGWRLTEQAGPEELVQRYVEPTGRNLNASQIEWSAYAEKSEPVTPR

>369|CORE_REP|Org73_Gene2215#

MNLVSEKEFLDLPLVSVAEIVRCRGPKVSVFPFDGTRRWFHLECNPQYDDYQQAALRQSIRILKMLFEHGIETVISPIFSDDLLDRGDRYIVQALEGMALLANDEEILSFYKEHEVHVLFYGDYKKRLPSTAQGAAVVKSFDDLTISTSSNTEHRLCFGVFGNDAAESVAQFSISWNETHGKPPTRREIIEGYYGEYVDKADMFIGFGRFSTFDFPLLSSGKTSLYFTVAPSYYMTETTLRRILYDHIYLRHFRPKPDYSAMSADQLNVLRNRYRAQPDRVFGVGCVHDGIWFAEG

>370|CORE_REP|Org59_Gene2146#

MSSPSRVSNTAVYAVLTIGAVITLSPFLLGLLTSFTSAHQFATGTPLQLPRPPTLANYADIADAGFRRAAVVTALMTAXILLGQLTFSVLAAYAFARLQFRGRDALFWVYVATLMVPGTVTVVPLYLMMAQLGLRNTFWALVLPFMFGSPYAIFLLREHFRLIPDDLINAARLDGANTLDVIVHVVIPSSRPVLAALAMITVVSQWNNFMWPLVITSGHKWRVLTVATADLQSRFNDQWTLVMAATTVAIVPLIALFVTFQRHIVASIVVXGLKXPXPASPRWXPPPLCWWRSCWV

>371|CORE_REP|Org119_Gene530#

MVESSTASATAVLRARYPRTAASLDRYGGGTARRLERTGTFARFTRISVVQIGWALRRYRRETLRLVAEIGMGTGAMAVVGGTVAIIGFVTLSGGSLIAIQGFASPRATSVSRRLPDSLPHWPNTRVAAPIVSGVALAATVGAGATAQLGAMRISEEIDALEVMGIKSISFLVSTRILGGLVVIMPLYALALDMAFTSGQVVTTVFYGQSNGTYEHYFRTFLRPEDVGWSVVEVVIIAVVVMITDCYYGYTASGGPVGVGQAVGRSMRFSLVSVVVVVLLAELALYGVDPNFNLTV

>374|CORE_REP|Org119_Gene587#

MAKLRPYYEESQSAYDISDDFFALFLDPTWVYTCAYFERDDMTLEEAQLAKVDLALDKLNLEPGMTLLDVGCGWGGALVRAVEKYDVNVIGLTLSRNHYERSKDRLAAIGTQRRAEARLQGWEEFEENVDRIVSFEAFDAFKKERYLTFFERSYDILPDDGRMLLHSLFTYDRRWLHEQGIALTMSDLRIPQIPAGVDLPGRRAAIGARHCRQCAGRGLHHRACPAAAAALRTDSRCMGRQPTGCPRTRHRRTVRRGLQQLHALSDRMRGALPQRPNQRRPVHHDQVARPLISVP

>376|CORE_REP|Org149_Gene1860#

MTETTDSPSERQPGPAEPELSSRDPDIAGQVFDAAPFDAAPDADSEGDSKAAKTDEPRPAKRSTLREFAVLAVIAVVLYYVMLTFVARPYLIPSESMEPTLHGCSTCVGDRIMVDKLSYRFGSPQPGDVIVFRGPPSWNVGYKSIRSHNVAVRWVQNALSFIGFVPPDENDLVKRVIAVGGQTVQCRSDTGLTVNGRPLKEPYLDPATMMADPSIYPCLGSEFGPVTVPPGRVWVMGDNRTHSADSRAHCPLLCTDDPLPGTVPVANVIGKARLIVWPPSRWGVVRSVNPQQGR

>381|CORE_REP|Org83_Gene888#

MTEFASRRTLVVRRFLRNRAAVASLAALLLLFVSAYALPPLLPYSYDDLDFNALLQPPGTKHWLGTNALGQDLLAQTLRGMQKSMLIGVCVAVISTGIAATVGAISGYFGGWRDRTLMWVVDLLLVVPSFILIAIVTPRTKNSANIMFLVLLLAGFGWMISSRMVRGMTMSLREREFIRAARYMGVSSRRIIVGHVVPNVASILIIDAALNVAAAILAETGLSFLGFGIQPPDVSLGTLIADGTASATAFPWVFLFPASILVLILVCANLTGDGLRDALDPASRSLRRGVR

>385|CORE_REP|Org72_Gene3626#

MRDAPRRRTALAYALLAPSLVGVVAFLLLPILVVVWLSLHRWDLLGPLRYVGLTNWRSVLTDSGFADSLVVTAVFVAIVVPAQTVLGLLAASLLARRLPGTGLFRTLYVLPWICAPLAIAVMWRWIVAPTDGAISTVLGHRIEWLTDPGLALPVVSAVVVWTNVGYVSLFFLAGLMAIPQDIHNAARTDGASAWQRFWRITLPMLRPTMFFVLVTGIISAAQVFDTVYALTGGGPQGSTDLVAHRIYAEAFGAAAIGRASVMAVVLFVILVGATVVQHLYFRRRISYELT

>388|CORE_REP|Org118_Gene328#

MRGIILAGGSGTRLYPITMGISKQLLPVYDKPMIYYPLTTLMMAGIRDIQLITTPHDAPGFHRLLGDGAHLGVNISYATQDQPDGLAQAFVIGANHIGADSVALVLGDNIFYGPGLGTSLKRFQSISGGAIFAYWVANPSAYGVVEFGAEGMALSLEEKPVTPKSNYAVPGLYFYDNDVIEIARGLKKSARGEYEITEVNQVYLNQGRLAVEVLARGTAWLDTGTFDSLLDAADFVRTLERRQGLKVSMPRRSGVAHGLDRRRAAGVQRARALVKSGYGNYLLELLERN

>391|CORE_REP|Org13_Gene1951#

MSDEDRTDRATEDHTIFDRGVGQRDQLQRLWTPYRMNYLAEAPVKRDPNSSASPAQPFTEIPQLSDEEGLVVARGKLVYAVLNLYPYNPGHLMVVPYRRVSELEDLTDLESAELMAFTQKAIRVIKNVSRPHGFNVGLNLGTSAGGSLAEHLHVHVVPRWGGDANFITIIGGLQGDSAAAARHPSAACHRVGSATMSKLPFLSRAAFARITTPIARGLLRVGLTPDVVTILGTTASVAGALTLFPMGKLFAGACVVWFFVLFDMLDGAMARERGGRHSLRRGAGRHL

>397|CORE_REP|Org105_Gene2694#

MGLSDWELAAARAAIARGLDEDLRYGPDVTTLATVPASATTTASLVTREAGVVAGLDVALLTLNEVLGTNGYRVLDRVEDGARVPPGEALMTLEAQTRGLLTAERTMLNLVGHLSGIATATAAWVDAVRGTKAKIRDTRKTLPGLRALQKYAVRTGGGVNHRLGLGDAALIKDNHVAAAGSVVDALRAVRNAAPDLPCEVEVDSLEQLDAVLPEKPELILLDNFAVWQTQTAVQRRDSRAPTVMLESSGGLSLQTAATYAETGVDYLAVGALTHSVRVLDIGLDM

>398|CORE_REP|Org77_Gene1327#

MVKRSRATRLSPSIWSGWESPQCRSIRARLLLPRGRSRPPNADCCWNQLAVTPDTRMPASSAAGRDAAAYDAWYDSPTGRPILATEVAALRPLIEVFAQPRLEIGVGTGRFADLLGVRFGLDPSRDALMFARRRGVLVANAVGEAVPFVSRHFGAVLMAFTLCFVTDPAAIFRETRRLLADGGGLVIGFLPRGTPWADLYALRAARGQPGYRDARFYTAAELEQLLADSGFRVIARRCTLHQPPGLARYDIEAAHDGIQAGAGFVAISAVDQAHEPKDDHPLESE

>405|CORE_REP|Org119_Gene3336#

MSPAPVQVMGVLNVTDDSFSDGGCYLDLDDAVKHGLAMAAAGAGIVDAGGGESSRPGATRVDPAVETSRVIPVVKELAAQGITVSIDTMRADVARAALQNGAQMVNDVSGGRADPAMGPLLAEADVPWVLMHWRAVSADTPHVPVRYGNVVAEVRADLLASVADAVAAGVDPARLVLDPGLGFAKTAQHNWAILHALPELVATGIPVLVGASRKRFLGALLAGPDGVMRPTDGRDTATAVISALAALHGAWGVRVHDVRASVDAIKVVEAWMGAERIERDG

>408|CORE_REP|Org19_Gene1144#

MGWADRIVHRHFIRGLALYAGLIGIAWCALFPIIWALSGSLKADGEVTEPTLFPSHPQWSNYREVFALMPFWRMFFNTVLYAGCVTAGQVFFCSLAGYAFARLQFRGRDTLFVLYLSTLMVPLTVTVIPQFILMRIVGWVDTPWAMIVPGLFGSAFGTYLMRQFFRTLPTDLEEAAILDGCSPWQIYWRILLPHSRPAVLVLGVLTWVNVWNDFLWPLLMIQRNSLATLTLGLVRLRGEYVARWPVLMAASMLMLVPLVILYAVAQRSFVRGIAVTGLGG

>410|CORE_REP|Org133_Gene2124#

MSPRVPRLRWDDPFRALDMLASLWSSTGMSLVSAGAAQAVAAPYRTLFTTLQQLLIGKEVTVRIGDHDVVLTVTELDSALEPQGLAVGQLGEVRVAARGISWDQHHLHSAVAVLRNVHIRPGVPPLVIAAPVELSSALPTEIFDDVLRQATPQLRGELSESGAARLRWARRPDWGGLEVDVDVAGTTSQTTLWLRPRTVITGQRRWTLPARTPAYRVPLPELPHGLRITDVSLAADCLQLSALLPEWRTELPLRYLESVITQLSQGALSFVWPPLRSGAD

>414|CORE_REP|Org20_Gene421#

MHESRLASARLYLCTDARRERGDLAQFAEAALAGGVDIIQLRDKGSPGELRFGPLQARDELAACEILADAAHRYGALFAVNDRADIARAAGADVLHLGQRDLPVNVARQILAPDTLIGRSTHDPDQVAAAAAGDADYFCVGPCWPTLPSGRAAPLGLVRVAAELGGDDKPWFAIGGINAQRLPAVLDAGASASWWCGRSPRLTTHVRRPSSSGRRLQQRTDPPTQRRGRLIRVARRTPEENPGLDLNGARQPLPPFVAPGQWHGRRSGPQSLPTGGESGM

>415|CORE_REP|Org119_Gene237#

MSAPTANRPAIGVFTPTRAQIPERTLRTDLWWLPPLLTNLGLLAFICYATTRAFWGSQYWVEKYHYLTPFYSPCVSASCQPGASHLGVWFGHFPGWIPLGAMVLPFLLGFRLTCYYYRKAYYRSVWQSPTSCAVPEPRAHYTGETRLPLIVQNTHRYFFYIAVVVSLINTYDAIAAFHSPSGFGFGLGNVILTINVVLLWAYTISCHSCRHATGGRLKHFSKHPVRYWIWTQVSKLNTRQHAIRVDHAGDPGAHRFLHHAGGQWQHHRSQIYWLKDRFRS

>420|CORE_REP|Org13_Gene801#

MRETSNPVFRSLPKQRGGYAQFGTGTAQQGFPADPYLAPYREAKATRPLTIDDVVTKTGLTLAMLAGTAVVSYFLVASNVALAMPLTLVGALGGLALVLVATFGRKQDNPAIVLSYAALEGLFLGAISFVLANFTVASANAGVLIGEAILGTMGVFFGMLVVYKTGAIRVTPKFTRMVVAALFGVLVLMLGNLVLAMFNVGGGEGLGLRSPGPLGIIFSLVCIGIAAFSFLIDFDAADQMIRAGAPEKAAWGVALGLTVTLVWLYIEILRLLSYLQNE

>424|CORE_REP|Org20_Gene726#

MGQKINPHGFRLGITTDWKSRWYADKQYAEYVKEDVAIRRLLSSGLERAGIADVEIERTRDRVRVDIHTARPGIVIGRRGTEADRIRADLEKLTGKQVQLNILEVKNPESQAQLVAQGVAEQLSNRVAFRRAMRKAIQSAMRQPNVKGIRVQCSGRLGGAEMSRSEFYREGRVPLHTLRADIDYGLYEAKTTFGRIGVKVWIYKGDIVGGKRELAAAAPAGADRPRRERPSGTRPRRAVLRAPRRPVPTRVGPRVAKRPRLTPQRPLKRRARRAESC

>425|CORE_REP|Org20_Gene430#

MNYLPLAPPGMTPPRVLSIAGSDSGGGAGIQADMRTMALLGVHACVAVTAVTVQNTLGVKDIHEVPNDVVAGQIEAVVTDIGVQAAKTGMLASSRIVATVAATWRRLELSVPLVVDPVCASMHGDPLLAPSALDSLRGQLFPLATLLTPNLDEARLLVDIEVVDAESQRAAAKALHALGPQWVLVKGGHLRSSDGSCDLLYDGVSCYQFDAQRLPTGDDHGGGDTLATAIAARWRTASPCPTRWTSGSDGLPNACARPIHWAAATGPFRRCFGCHEP

>427|CORE_REP|Org119_Gene1497#

MSPATVLDSILEGVRADVAAREASVSLSEIKAAAAAAPPPLDVMAALREPGIGVIAEVKRASPSAGALATIADPAKLAQAYQDGGARIVSVVTEQRRFQGSLDDLDAVRASVSIPVLRKDFVVQPYQIHEARAHGADMLLLIVAALEQSVLVSMLDRTESLGMTALVEVHTEQEADRALKAGAKVIGVNARDLMTLDVDRDCFARIAPGLPSSVIRIAESGVRGTADLLAYAGAGADAVLVGEGLVTSGDPRAAVADLVTAGTHPSCPKPARASRR

>430|CORE_REP|Org11_Gene710#

MAQKPVADALTLELEPVVEANMTRHLDTEDIWFAHDYVPFDQGENFAFLGGRDWDPSQSTLPRTITDACEILLILKDNLAGHHRELVEHFILEDWWGRWLGRWTAEEHLHAIALREYLVVTREVDPVANEDVRVQHVMKGYRAEKYTQVETLVYMAFYERCGAVFCRNLAAQIEEPILAGLIDRIARDEVRHEEFFANLVTHCLDYTRDETIAAIAARAADLDVLGADIEAYRDKLQNVADAGIFGKPQLRQLISDRITAWGLAGEPSLKQFVTG

>431|CORE_REP|Org61_Gene1948#

MCAFVPHVPRHSRGDNPPSASTASPAVLTLTGERTIPDLDIENYWFRRHQVVYQRLAPRCTARDVLEAGCGEGYGADLIACVARQVIAVDYDETAVAHVRSRYPRVEVMQANLAELPLPDASVDVVVNFQVIEHLWDQARFVRECARVLRGSGLLMVSTPNRITFSPGRDTPINPFHTRELNADELTSLLIDAGFVDVAMCGLFHGPRLRDMDARHGGSIIDAQIMRAVAGAPWPPELAADVAAVTTADFEMVAAGHDRDIDDSLDLIAIAVRP

>432|CORE_REP|Org20_Gene2745#

MPWTTDADGGPALVEFAGRACYQSWSKPNPKTATNAGYLRHIIDVGHFSVLEHASVSFYITGISRSCTHELIRHRHFSYSQLSQRYVPEKDSRVVVPPGMEDDADLRHILTEAADDRRAIYSELLSRWKPSSPTNPTRSCAASRPAKPSRAVLPNATETRIVVTGNYRAWRHFIAMRASEHADVEIRRLAIECLRQLAAVAPAVFADFEVTTLADGTEVATSPLAPKPEAACRWTNTRARGRDKAPGNLGSRDHRRIRRRSAPRNPADRDGDTV

>435|CORE_REP|Org92_Gene2680#

MIPVKVENNTSLDQVQDALNCVGYAVVEDVLDEASLAATRDRMYRVQERILTEIGKERLARAGELGVLRLMMKYDPHFFTFLEIPEVLSIVDRVLSETAILHLQNGFILPSFPPFSTPDVFQNAFHQDFPRVLSGYIASVNIMFAIDPFTRDTGATLVVPGSHQRIEKPDHTYLARNAVPVQCAAGSLFVFDSTLWHAAGRNTSGKDRLAINHQFTRSFFKQQIDYVRALGDAVVLEQPARTQQLLGWYSRVVTNLDEYYQPPDKRLYRKGQG

>436|CORE_REP|Org34_Gene841#

MRRCIPHRCIGHGTVVSVRITVLGCSGSVVGPDSPASGYLLRAPHTPPLVIDFGGGVLGALQRHADPASVHVLLSHLHADHCLDLPGLFVWRRYHPSRPSGKALLYGPSDTWSRLGAASSPYGGEIDDCSDIFDVHHWADSEPVTLGALTIVPRLVAHPTESFGLRITDPSGASLAYSGDTGICDQLVELARGVDVFLCEASWTHSPKHPPDLHLSGTEAGMVAAQAGVRELLLTHIPPWTSREDVISEAKAEFDGPVHAVVCDETFEVRRAG

>437|CORE_REP|Org32_Gene1810#

MIDRPLEGKVAFITGAARGLGRAHAVRLAADGANIIAVDICEQIASVPYPLSTADDLAATVELVEDAGGGIVARQGDVRDRASLSVALQAGLDEFGRLDIVVANAGIAMMQAGDDGWRDVIDVNLTGVFHTVQVAIPTLIEQGTGGSIVLISSAAGLVGIGSSDPGSLGYAAAKHGVVGLMRAYANHLAPQNIRVNSVHPCGVDTPMINNEFFQQWLTTADMDAPHNLGNALPVELVQPTDIANAVAWLASEEARYVTGVTLPVDAGFVNKR

>438|CORE_REP|Org28_Gene1579#

MTSLPAARYLVRSVALGYVFVLLIVPVALILWRTFEPGFGQFYAWISTPAAISALNLSLLVVAIVVPLNVIFGVTTALVLARNRFRGKGVLQAIIDLPFAVSPVIVGVSLILLWGSAGALGFVEQDLGFKIIFGLPGIVLASMFVTCPFVVREVEPVLHELGTDQEQAAATLGSGWWQTFWRITLPSIRWGLTYGIVLTVARTLGEYGAVIIVSSNLPGTSQTLTLLVSDRYHRGAEYGAYALSTLLMAVSVVVLIVQMVLDARRARAVSEG

>439|CORE_REP|Org75_Gene2351#

MLLAIDVRNTHTVVGLLSGMKEHAKVVQQWRIRTESEVTADELALTIDGLIGEDSERLTGTAALSTVPSVLHEVRIMLDQYWPSVPHVLIEPGVRTGIPLLVDNPKEVGADRIVNCLAAYDRFRKAAIVVDFGSSICVDVVSAKGEFLGGAIAPGVQVSSDAAAARSAALRRVELARPRSVVGKNTVECMQAGAVFGFAGLVDGLVGRIREDVSGFSVDHDVAIVATGHTAPLLLPELHTVDHYDQHLTLQGLRLVFERNLEVQRGRLKTAR

>440|CORE_REP|Org55_Gene992#

MRAVFGCAIAVVGIAGSVVAGPADIHLVAAKQSYGFAVASVLPTRGQVVGVAHPVVVTFSAPITNPANRHAAERAVEVKSTPAMTGKFEWLDNDVVQWVPDRFWPAHSTVELSVGSLSSDFKTGPAVVGVASISQHTFTVSIDGVEEGPPPPLPAPHHRVHFGEDGVMPASMGRPEYPTPVGSYTVLSKERSVIMDSSSVGIPVDDPDGYRLSVDYAVRITSRGLYVHSAPWALPALGLENVSHGCISLSREDAEWYYNAVDIGDPVIVQE

>442|CORE_REP|Org59_Gene2732#

MSMLARHGPRYGGSXNGHSDXSXGXAKXAAPTLYIFPHAGGTAKDYVAFSREFSADVKRIAVQYPGQHDRSGLPPLESIPTLADEIFAMMKPSARIDDPVAFFGHSMGGMLAFEVALRYQSAGHRVLAFFVSXXSAPGHIRYKQLQDLSDREMLDLFTRMTGMNPDFFTDXEFFVGALPTLRAVRAIAGYSCPPETKLSCPIYAFIGDKDWIATQDDMDPWRDRTTEEFXIRVFPGDHFYLNDNLPELVSDIEDKTLQWLXSXPXLCSGCS

>443|CORE_REP|Org5_Gene583#

MPMRKVLVGVTGAAIVVAVLIVGAVGADFGASIYAEYRLSTTVRKAANLRSDPFVAILRFPFIPQAMREHYAELEIKAFAVEHAGSGTATLEATMHSIDLSYASWLIRPDAKLPVGELESRIIIDSMHLGRYLGISDLMVAAPRQESNDATGGTTESGISGSRGLVFSGTPISANFAHRVSVLVDLSVASDDRATLVITPTAVVTGPDTADQPVPDDKRDAVLHAFASKLPNQKLPFGVVPNTVGARGSDVIIEGITRGVTISLDEFKQS

>447|CORE_REP|Org119_Gene1362#

MTILEIKDLHVSVENPAEADHEIPILRGVDLTVKSGETHALMGPNGSGKSTLSYAIAGHPKYHVTSGTITLDGADVLAMSIDERARAGLFLAMQYPVEVPGVSMSNFLRSAATAIRGEPPKLRHWVKEVKAAMAALDIDPAFAERSVNEGFSGGEKKRHEILQLELLKPKIAILDETDSGPGRRRAARGQRGGEPLRRIPARRHPADHALHPHPALHPPGIRARVRRRPHRRVRWFGARRRTRPERLRAFLPRKRAVPPPTRANRSLT

>450|CORE_REP|Org69_Gene3393#

MTDTRVLAVANQKGGVAKTTTVASLGAAMVEKGRRVLLVDLDPQGCLTFSLGQDPDKLPVSVHEVLLGEVEPNAVLVTTMEGMTLLPANIDLAGAEAMLLMRAGREYALKRALAKFSDRFDVVIIDCPPSLGVLTLNGLTAADEAIVPLQCEMLAHRGVGQFLRTVADVQQITNPNLRLLGALPTLYDSRTTHTRDVLLDVADRYDLQVLAPPIPRTVRFAEASASGSSVMAGRKNKGAVAYRELAQALLKHWKTGRPLPTFTVDL

>451|CORE_REP|Org142_Gene3020#

MTDRDRLRPPLDERSLRDQLIGAGSGWRQLDVVAQTGSTNADLLARAASGADIDGVVLIAEHQTAGRGRHGRGWAATARAQIILSVGVRVVDVPVQAWGWLSLAAGLAVLDSVAPLIAVPPAETGLKWPNDVLARGGKLAGILAEVAQPFVVLGVGLNVTQAPEEVDPDATSLLDLGVAAPDRNRIASRLLRELEARIIQWRNANPQLAADYRARSLTIGSRVRVELPGGQDVVGIARDIDDQGRLCLDVGGRTVVVSAGDVVHLR

>456|CORE_REP|Org118_Gene1241#

MTETILAAQIEVGEHHTATWLGMTVNTDTVLSTAIAGLIVIALAFYLRAKVTSTDVPGGVQLFFEAITIQMRNQVESAIGMRIAPFVLPLAVTIFVFILISNWLAVLPVQYTDKHGHTTELLKSAAADINYVLALALFVFVCYHTAGIWRRGIVGHPIKLLKGHVTLLAPINLVEEVAKPISLSLRLFGNIFAGGILVALIALFPPYIMWAPNAIWKAFDLFVGAIQAFIFALLTILYFSQAMELEGGTPLVPDAGNGYQSHQGG

>458|CORE_REP|Org104_Gene1889#

MTKTWPPRTVIRKSGGLRGMRTLESALHRGGLGPVAGVDEVGRGACAGPLVVAACVLGPGRIASLAALDDSKKLSEQAREKLFPLICRYAVAYHVVFIPSAEVDRRGVHVANIEGMRRAVAGLAVRPGYVLSDGFRVPGLPMPSLPVIGGDAAAACIAAASVLAKVSRDRVMVALDADHPGYGFAEHKGYSTPAHSRALARLGPCPQHRYSFINVRRVASGSNTAEVADGQPDPRDGTAQTGEGRWSKSSHPATMRATGRAQGT

>463|CORE_REP|Org104_Gene167#

MVHHGQMHAQPGVGLRPDTPVASGQLPSTSIRSRRSGISKAQRETWERLWPELGLLALPQSPRGTPVDTRAWFGRDAPVVLEIGSGSGTSTLAMAKAEPHVDVIAVDVYRRGLAQLLCAIDKVGSDGINIRLILGNAVDVLQHLIAPDSLCGVRVFFPDPWPKARHHKRRLLQPATMALIADRLVPSGVLHAATDHPGYAEHIAAAGDAEPRLVRVDPDTELLPISVVRPATKYERKAQLGGGAVIELLWKKHGCSERDLKIR

>466|CORE_REP|Org142_Gene1047#

MTSAPTVSVITISFNDLDGLQRTVKSVRAQRYRGRIEHIVIDGGSGDDVVAYLSGCEPGFAYWQSEPDGGRYDAMNQGIAHASGDLLWFLHSADRFSGPDVVAQAVEALSGKGPVSELWGFGMDRLVGLDRVRGPIPFSLRKFLAGKQVVPHQASFFGSSLVAKIGGYDLDFGIAADQEFILRAALVCEPVTIRCVLCEFDTTGVGSHREPSAVFGDLRRMGDLHRRYPFGGRRISHAYLRGREFYAYNSRFWENVFTRMSK

>471|CORE_REP|Org59_Gene2937#

MSHDDLMLALALADRADELTRVRFGALDLRIDTKPDLTPVTDADRAVESDVRQTLGRDRPGDGVLGEEFGGSTTFTGRQWIVDPIDGTKNFVRGVPVWASLIALLEDGVPSVGVVSAPALQRRWWAARGRGAFASVDGARPHRLSVSSVAELHSASLSFSSLSGWARXGLRERFIGLTDTVWRVRAYGDFLSYCLVAEGAVDIAAEPQVSVWDLAALDIVVREAGGRLTSLRRTXAGPHGGSAVATNGLLHDEVLTRLNAG

>477|CORE_REP|Org62_Gene2295#

MTLAEAADAINFGLAGRVVLVTGGVRGVGAGISSVFAEQGATVITCARRAVDGQPYEFHRCDIRDEDSVKRLVGEIGERHGRLDMLVNNAGGSPYALAAEATHNFHRKIVELNVLAPLLVSQHANVLMQAQPNGGSIVNICSVSGRRPTPGTAAYGAAKAGLENLTTTLAVEWAPKVRVNAVVVGMVETERSELFYGDAESIARVAATVPLGRLARPADIGWAAAFLASDAASYISGATLEVHGGGEPPPYLGASSANK

>478|CORE_REP|Org148_Gene939#

MSKREDGRLDHELRPVIITRGFTENPAGSVLIEFGHTKVLCTASVTEGVPRWRKATGLGWLTAEYAMLPSATHSRSDRESVRGRLSGRTQEISRLIGRSLRACIDLAALGENTIAIDCDVLQADGGTRTAAITGAYVALADAVTYLSAAGKLSDPRPLSCAIAAVSVGVVDGRIRVDLPYEEDSRAEVDMNVVATDTGTLVEIQGTGEGATFARSTLDKLLDMALGACDTLFAAQRDALALPYPGVLPQGPPPPKAFGT

>482|CORE_REP|Org31_Gene855#

MELLGGPRVGNTESQLCVADGDDLPTYCSANSEDLNITTITTLSPTSMSHPQQVRDDQWVEPSDQLQGTAVFDATGDKATMPSWDELVRQHADRVYRLAYRLSGNQHDAEDLTQETFIRVFRSVQNYQPGTFEGWLHRITTNLFLDMVRRRARIRMEALPEDYDRVPADEPNPEQIYHDARLGPDLQAALASLPPEFRAAVVLCDIEGLSYEEIGATLGVKLGTVRSRIHRGRQALRDYLAAHPEHGECAVHVNPVR

>483|CORE_REP|Org22_Gene721#

MTTMSGYTRSQRPRQAILGQLPRIHRADGSPIRVLLVDDEPALTNLVKMALHYEGWDVEVAHDGQEAIAKFDKVGPDVLVLDIMLPDVDGLEILRRVRESDVYTPTLFLTARDSVMDRVTGLTSGADDYMTKPFSLEELVARLRGLLRRSSHLERPADEALRVGDLTLDGASREVTRDGTPISLSSTEFELLRFLMRNPRRALSRTEILDRVWNYDFAGRTSIVDLYISYLRKKIDSDREPMIHTVRGIGYMLRPPE

>485|CORE_REP|Org102_Gene3785#

MADKSKRPPRFDLKSADGSFGRLVQIGGTTTIVVVFAVVLVFYIVTSRDDKKDGVAGPGDAVRVTSSKLVTQPGTSNPKAVVSFYEDFLCPACGIFERGFGPTVSKLVDIGAVAADYTMVAILDSASNQHYSSRAAAAAYCVADESIEAFRRFHAALFSKDIQPAELGKDFPDNARLIELAREAGVVGKVPDCINSGKYIEKVDGLAAAVNVHATPTVRVNGTEYEWSTPAALVAKIKEIVGDVPGIDSAAATATS

>486|CORE_REP|Org59_Gene956#

MAKSASNQLRVTVRTETGKGASRRARRAGKIPAVLYGHGAEPQHLELPGHDYAAVLRHSGTNAVLTLDIAGKEQLALTKALHXHPIRRTIQHADLLVVRRGEKVVVEVSVVVEGQAGPDTLVTQETNSIEIEAEALSIPEQLTVSIEGAEPGTQLTAGQIALPAGVSLISDPDLLVVNVVKAPXAEEXXGXXAXEXXKPRKPRXKPAKPRPLASPSRRLATWPSRCSWSASATLEPITPVPGTTSGSWSPICSPRD

>489|CORE_REP|Org35_Gene2559#

MMISSSDELLRDGADPAVIIDQLRVIRGKRLALQDVSVRVACGTITGLLGPSGSGKTTLIRCIVGSQIIASGSVSVLGQPAGSAELRHRVGYMPQDPTIYNDLRVIDNIRYFAELCGVDRQAADEVIEAVDLRDHRTARCANLSGGQRARVSLACALVGRPDLLVLDEPTIGLDPVLRVELWDRFTALARRGTTLLVSSHVMDEADRCGDLLLLRQGQLLAHTTPHRLRKETGCTSLEEAFLSIVRRTTTVPAAG

>492|CORE_REP|Org107_Gene2468#

MVLDAVGNPQTVLLLGGTSEIGLAICERYLHNSAARIVLACLPDDPRREDAAAAMKQAGARSVELIDFDALDTDSHPKMIEAAFSGGDVDVAIVAFGLLGDAEELWQNQRKAVQIAEINYTAAVSVGVLLAEKMRAQGFGQIIAMSSAAGERVRRANFVYGSTKAGLDGFYLGLSEALREYGVRVLVIRPGQVRTRMSAHLKEAPLTVDKEYVANLAVTASAKGKELVWAPAAFRYVMMVLRHIPRSIFRKLPI

>496|CORE_REP|Org33_Gene304#

MAESKLVIGDRSFASRLIMGTGGATNLAVLEQALIASGTELTTVAIRRVDADGGTGLLDLLNRLGITPLPNTAGCRSAAEAVLTAQLAREALNTNWVKLEVIADERTLWPDAVELVRAAEQLVDDGFVVLPYTTDDPVLARRLEDTGCAAVMPLGSPIGTGLGIANPHNIEMIVAGARVPVVLDAGIGTASDAALAMELGCDAVLLASAVTRAADPPAMAAAMAAAVTAGYLARCAGRIPKRFWAQASSPAR

>501|CORE_REP|Org20_Gene2115#

MAVRSEFSVFHSPEQAMRERSELARKGIARAKSVVALAYAGGVLFVAENPSRSLQKISELYDRVGFAAAGKFNEFDNLRRGGIQFADTRGYAYDRRDVTGRQLANVYAQTLGTIFTEQAKPYEVELCVAEVAHYGETKPPELYRITYDGSIAEEPHFVVMGGTTEPIANALKESYAENASLTDALGIAVAALRAGSADTSGGDQPTLGVASLEVAVLDANRPRRAFRRITGSALQALLVDQESPQSDGESSG

>502|CORE_REP|Org111_Gene1716#

MSGHSKWATTKHKKAVVDARRGKMFARLIKNIEVAARVGGGDPAGNPTLYDAIQKAKKSSVPNENIERARKRGAGEEAGGADWQTIMYEGYAPNGVAVLIECLTDNRNRAASEVRVAMTRNGGTMADPGSVSYLFSRKGVVTLEKNGLTEDDVLAAVLEAGAEDVNDLGDSFEVISEPAELVAVRSALQDAGIDYESAEASFQPSVSVPVDLDGARKVFKLVDALEDSDDVQNVWTNVDVSDEVLAALDDE

>503|CORE_REP|Org135_Gene3644#

MTVYFIGAGPGAADLITVRGQRLLQRCPVCLYAGSIMPDDLLAQCPPGATIVDTGPLTLEQIVRKLADADADGRDVARLHSGDPSLYSALAEQCRELDALGIGYEIVPGVPAFAAAAAALKRELTVPGVAQTVTLTRVATLSTPIPPGEDLAALARSRATLVLHLAAAQIDAIVPRLLDGGYRPETPVAVVAFASWPQQRTLRGTLADIAARMHDAKITRTAVIVVGDVLTAEGFTDSYLYSVARHGRYAQ

>509|CORE_REP|Org119_Gene2031#

MWYYLFKYIFMGPLFTLLGRPKVEGLEYIPSSGPAILASNHLAVADSFYLPLVVRRRIWFLAKSEYFTGTGLKGWINRWFYSVSGQVPIDRTNADSAQGALQTAVVLLGQGKLLGMYPEGTRSPDGRLYKGKTGLARLALHTGVPVIPVAMIGTNVVNPPGRKMLRFGRVTVRFGKPMDFSRFEGLAGNHFIERAVTDEVIYELMGLSGQEYVDIYAASVVKDGRNAGGAGANPNSTDAARIPETAAG

>518|CORE_REP|Org91_Gene1477#

MKAGVAQQRSLLELAKLDAELTRIAHRATHLPQRAAYQQVQAEHNAANDRMAALRIAAEDLDGQVSRFESEIDAVRKRGDRDRSLLTSGATDAKQLADLQHELDSLQRRQASLEDALLEVLERREELQAQQTAESRALQALRADLAAAQQALDEALAEIDQARHQHSSQRDMLTATLDPELAGLYERQRAGGGPGAGRLQGHRCGACRIEIGRGELAQISAAAEDEVVRCPECGAILLRLEGFEE

>519|CORE_REP|Org92_Gene1926#

MYRVFEALDELSAIVEEARGVPMTAGCVVPRGDVLELIDDIKDAIPGELDDAQDVLDARDSMLQDAKTHADSMVSSATTEAESILNHARTEADRILSDAKAQADRMVSEARQHSERMVADAREEAIRIATAAKREYEASVSRAQAECDRLIENGNISYEKAVQEGIKEQQRLVSQNEVVAAANAESTRLVDTAHAEADRLRGECDIYVDNKLAEFEEFLNGTLRSVGRGRHQLRTAAGTHDYAVR

>520|CORE_REP|Org75_Gene2388#

MPGRWSAETRLALVRRARRMNRALAQAFPHVYCELDFTTPLELAVATILSAQSTDKRVNLTTPALFARYRTARDYAQADRTELESLIRPTGFYRNKAASLIGLGQALVERFGGEVPATMDKLVTLPGVGRKTANVILGNAFGIPGITVDTHFGRLVRRWRWTTAEDPVKVEQAVGELIERKEWTLLSHRVIFHGRRVCHARRPACGVCVLAKDCPSFGLGPTEPLLAAPLVQGPETDHLLALAGL

>526|CORE_REP|Org47_Gene1050#

MRLARRARNILRRNGIEVSRYFAELDWERNFLRQLQSHRVSAVLDVGANSGQYARGLRGAGFAGRIVSFEPLPGPFAVLQRSASTDPLWECRRCALGDVDGTISINVAGNEGASSSVLPMLKRHQDAFPPANYVGAQRVPIHRLDSVAADVLRPNDIAFLKIDVQGFEKQVIAGGDSTVHDRCVGMQLELSFQPLYEGGMLIREALDLVDSLGFTLSGLQPGFTDPRNGRMLQADGIFFRGSD

>527|CORE_REP|Org83_Gene3136#

MPNFWALPPEINSTRIYLGPGSGPILAAAQGWNALASELEKTKVGLQSALDTLLESYRGQSSQALIQQTLPYVQWLTTTAEHAHKTAIQLTAAANAYEQARAAMVPPAMVRANRVQTTVLKAINWFGQFSTRIADKEADYEQMWFQDALVMENYWEAVQEAIQSTSHFEDPPEMADDYDEAWMLNTVFDYHNENAKEEVIHLVPDVNKERGPIELVTKVDKEGTIRLVYDGEPTFSYKEHPKF

>531|CORE_REP|Org22_Gene2685#

MNSHCSHTFITDNRSPRARRGHAMSTLHKVKAYFGMAPMEDYDDEYYDDRAPSRGYARPRFDDDYGRYDGRDYDDARSDSRGDLRGEPADYPPPGYRGGYADEPRFRPREFDRAEMTRPRFGSWLRNSTRGALAMDPRRMAMMFEDGHPLSKITTLRPKDYSEARTIGERFRDGSPVIMDLVSMDNADAKRLVDFAAGLAFALRGSFDKVATKVFLLSPADVDVSPEERRRIAETGFYAYQ

>533|CORE_REP|Org132_Gene1903#

MIRSRQPLLDALGVDLPDELLSLALTHRSYAYENGGLPTNERLEFLGDAVLGLTITDALFHRHPDRSEGDLAKLRASVVNTQALADVARRLCAEGLGVHVLLGRGEANTGGADKSSILADGMESLLGAIYLQHGMEKAREVILRLFGPLLDAAPTLGAGLDWKTSLQELTAARGLGAPSYLVTSTGPDHDKEFTAVVVVMDSEYGSGVGRSKKEAEQKAAAAAWKALEVLDNAMPGKTSA

>538|CORE_REP|Org1_Gene3733#

MDGVDRSRGWTHPYQPPFRGPSHDCYIGFNAVQVHVVDHPLAAARLTTLRDERTDNAGFRAALRELTLLLIYEATRDAPCEPVPIRTPLAETVGSRLTKPPLLVPVLRAGLGMVDEAHAALPEAHVGFVGVARDEQTHQPVPYLDSLPDDLTDVPVMVLDPMVATGGSMTHTLGLLISRGAADITVLCVVAAPEGIAALQKAAPNVRLFTAAIDEGLNEVAYIVPGLGDAGDRQFGPR

>546|CORE_REP|Org146_Gene2041#

MSPPNQDAQEGRPDSPTAEVVDVRRGMFGVSGTGDTSGYGRLVRQVVLPGSSPRPYGGYFDDIVDRLAEALRHERVEFEDAVEKVVVYRDELTLHVRRDLLPRVAQRLRDEPELRFELCLGVSGVHYPHETGRELHAVYPLQSITHNRRLRLEVSAPDSDPHIPSLFAIYPTNDWHERETYDFFGIIFDGHPALTRIEMPDDWQGHPQRKDYPLGGIPVEYKGAQIPPPDERRGYN

>547|CORE_REP|Org28_Gene2905#

MNDSNDTSVAGGAAGADSRVLSADSALTERQRTILDVIRASVTSRGYPPSIREIGDAVGLTSTSSVAHQLRTLERKGYLRRDPNRPRAVNVRGADDAALPPVTEVAGSDALPEPTFVPVLGRIAAGGPILAEEAVEDVFPLPRELVGEGTLFLLKVIGDSMVEAAICDGDWVVVRQQNVADNGDIVAAMIDGEATVKTFKRAGGQVWLMPHNPAFDPIPGNDATVLGKVVTVIRKV

>548|CORE_REP|Org137_Gene2206#

MAKRTPVRKACTVLAVLAATLLLGACGGPTQPRSITLTFIRNAQSQANADGIIDTDMPGSGLSADGKAEAQQVAHQVSRRDVDSIYSSPMAADQQTAGPLAGELGKQVEILPGLQAINAGWFNGKPESMANSTYMLAPADWLAGDVHNTIPGSISGTEFNSQFSAAVRKIYDSGHNTPVVFSQGVAIMIWTLMNARNSRDSLLTTHPLPNIGRVVITGNPVTGWRLVEWDGIRNFT

>553|CORE_REP|Org1_Gene4082#

MPNTNPVAAWKALKEGNERFVAGRPQHPSQSVDHRAGLAAGQKPTAVIFGCADSRVAAEIIFDQGLGDMFVVRTAGHVIDSAVLGSIEYAVTVLNVPLIVVLGHDSCGAVNAALAAINDGTLPGGYVRDVVERVAPSVLLGRRDGLSRVDEFEQRHVHETVAILMARSSAISERIAGAAWRSWASPINSTMGGLYCATTSATSARRSELPPDNRPPLTRRRPAKPATRRGGSSAFA

>554|CORE_REP|Org40_Gene475#

MSKTSKAYRAAAAKVDRTNLYTPLQAAKLAKETSSTKQDATVEVAIRLGVDPRKADQMVRGTVNLPHGTGKTARVAVFAVGEKADAAVAAGADVVGSDDLIERIQGGWLEFDAAIATPDQMAKVGRIARVLGPRGLMPNPKTGTVTADVAKAVADIKGGKINFRVDKQANLHFVIGKASFDEKLLAENYGAAIDEVLRLKPSSSKGRYLKKITVSTTTGPGIPVDPSITRNFAGE

>555|CORE_REP|Org59_Gene769#

MFDLRITTPRLQLQLPTEELCDQLIDTILEGVHDPDRMPFSVPWTRASREXLPFNTLSHLWQQLAGXKRDDWSLPLAVLVDGRAVGVQALSSKDFPITRQVDSGSWLGLRYQGHGYGTEMRAAVLYFAFAELEAQVATSRSFVDNPASIAXXRRNGYRDNGLDRVAREGAMAEALLFRLTRDDWQRHRTVEVRVDGFDRCRPLFGPLEPPRYXPATQKALPSGSASTTQRKLSPT

>557|CORE_REP|Org20_Gene2537#

MRGAGGRGRGGDHALVLARGVRWKNSAGIRWPKPSATSPPTSAASPTAKHRPRGSPVNRWHQLFDRPAGLGKSTILRWPRRSGSACSTPTTRSSSATGRSIADIFATDGEQEFRRIEEDVVRAALADHDGVLSLGGGAVTSPGVRAALAGHTALMKISAAEGVRRTGGNTVRPLLAGPDRAEKYRALMAKRARCTGAWTCGGHQSPQPRGGGPPYPVAAAGSQPQRGGQAESAHP

>568|CORE_REP|Org54_Gene2857#

MPSPSSADQVADSPRPRLPADHPGVNELFALLAYGEVAAFYRLTDEARMAPDLRGRISMASMAAAEMGHYELLRNALERRGVDVVSAMSKYTSALENYHRLTTPSTWLEALVKTYVADALAADLYLEIADGLPDEVADVVRAALSETGHSQFVVAEVRAAVTASGKQRSRLALWSRRLLGEAITQAQLVLADHDELVDLVVSGSGGLSQLGAFFDRLQQTHDQRMRELGLS

>569|CORE_REP|Org30_Gene1769#

MARLDYDALNATLRYLMFSVFSVSPGALGDQRDAIIDDASTFFKQQEERGVVVRGLYDVAGLRADADFMVWTHAERVEALQATYADFRRTTTLGRACTPVWSGVGLHRPAEFNKSHIPAFLAGEEPGAYICVYPFVRSYEWYLLPDEERRRMLAEHGMAARGYKDVRANTVPAFALGDYEWILAFEAPELDRIVDLMRELRATDARRHTRAETPFFTGPRVPVEQLVHSLP

>571|CORE_REP|Org96_Gene319#

MARRPRPDGPQHLLALVRSAVPPVHPAGRPFIAAGLAIAAVGHRYRWLRGTGLLAAAACAGFFRHPQRVPPTRPAAIVAPADGVICAIDSAAPPAELSMGDTPLPRVSIFLSILDAHVQRAPVSGEVIAVQHRPGRFGSADLPEASDDNERTSVRIRMPNGAEVVAVQIAGLVARRIVCDAHVGDKLAIGDTYGLIRFGSRLDTYLPAGAEPIVNVGQRAVAGETVLAECR

>575|CORE_REP|Org125_Gene1079#

MKRSMKSGSFAIGLAMMLAPMVAAPGLAAADPATRPVDYQQITDVVIARGLSQRGVPFSWAGGGISGPTRGTGTGINTVGFDASGLIQYAYAGAGLKLPRSSGQMYKVGQKVLPQQARKGDLIFYGPEGTQSVALYLGKGQMLEVGDVVQVSPVRTNGMTPYLVRVLGTQPTPVQQAPVQPAPVQQAPVQQAPVQQAPVQQAPVQQAPVQQAPVQQAPVQPPPFGTARSR

>579|CORE_REP|Org147_Gene3012#

MDTMRQRILVVDDDASLAEMLTIVLRGEGFDTAVIGDGTQALTAVRELRPDLVLLDLMLPGMNGIDVCRVLRADSGVPIVMLTAKTDTVDVVLGLESGADDYIMKPFKPKELVARVRARLRRNDDEPAEMLSIADVEIDVPAHKVTRNGEQISLTPLEFDLLVALARKPRQVFTRDVLLEQVWGYRHPADTRLVNVHVQRLRAKVEKDPENPTVVLTVRGVGYKAGPP

>580|CORE_REP|Org7_Gene631#

MPEAKRPESKRRSPASRPGKAGDSVRGGRATKPSAKPSTPAPHASRKTTRTPHEHIVEPIKRAITESVEKRSEQRLGFTARRAAILAAVVCVLTLTIARPVRTYFAQRAEMEQLAATEAMLRRQIADLEEQQVKLADPAYIAAQARERLGFVMPGDIPFQVQLPSTPLAPPQPGSDAATATNNEPWYTALWHTIADDPHLPPAAPPAPEPGRPGPLPPASPNPEQPGG

>587|CORE_REP|Org133_Gene366#

MTSVLIVEDEESLADPLAFLLRKEGFEATVVTDGPAALAEFDRAGADIVLLDLMLPGMSGTDVCKQLRARSSVPVIMVTARDSEIDKVVGLELGADDYVTKPYSARELIARIRAVLRRGGDDDSEMSDGVLESGPVRMDVERHVVSVNGDTITLPLKEFDLLEYLMRNSGRVLTRGQLIDRVWGADYVGDTKTLDVHVKRLRSKIEADPANPVHLVTVRGLGYKLEG

>591|CORE_REP|Org40_Gene740#

MTLVLVIDDEPQILRALRINLTVRGYQVITASTGAGALRAAAEHPPDVVILDLGLPDMSGIDVLGGLRGWLTAPVIVLSARTDSSDKVQALDAGADDYVTKPFGMDEFLARLRAAVRRNTAAAELEQPVIETDSFTVDLAGKKVIKDGAEVHLTPTEWGMLEMLARNRGKLVGRGELLKEVWGPAYATETHYLRVYLAQLRRKLEDDPSHPKHLLTESGMGYRFEA

>599|CORE_REP|Org19_Gene1426#

MLITGFPAGLLACNCYVLAERPGTDAVIVDPGQGAMGTLRRILDKNRLTPAAVLLTHGHIDHIWSAQKVSDTFGCPTYVHPADRFMLTDPIYGLGPRIAQLVAGAFFREPKQVVELDRDGDKIDLGGISVNIDHTPGHTRGSVVFRVLQATNNDKDIVFTGDTLFERAIGRTDLAGGSGRDLLRSIVDKLLVLDDSTVVLPGHGNSTTIGAERRFNPFLEGLSR

>600|CORE_REP|Org138_Gene92#

MSVYKHAPSRVRLRQTRSTVVKGRSGSLSWRRVRTGDLGLAVWGGREEYRAVKPGTPGIQPKGDMMTVTVVDAGPGRVSRSVEVAAPAAELFAIVADPRRHRELDGSGTVRGNIKVPAKLVVGSKFSTKMKLFGLPYRITSRVTALKPNELVEWSHPLGHRWRWEFESLSPTLTRVTETFDYHAAGAIKNGLKFYEMTGFAKSNAAGIEATLAKLSDQYARGRA

>603|CORE_REP|Org59_Gene92#

MSNANFSILVDFAAGGLVLASVLIVWRRDLSPXXVRLLAWQXELRTAXPLXPLLRGIRDNDRALIAVGIAVLALRALVLPWLLARAVGAEAAAQREATPLVNTASSLLITAGLTLTAFAITQPVVNLEPGVTINAVPAAFAVVLIALFVMTTRLHAVSQAAGFLMLDNGIAATAFLLTAGVPLIVELGASLDVLFAVIVIGVLTGRLRRIFGDADLDKLRELRD

>604|CORE_REP|Org78_Gene575#

MTARIGVVTFPGTLDDVDAARAARQVGAEVVSLWHADADLKGVDAVVVPGGFSYGDYLRAGAIARFAPVMDEVVAAADRGMPVLGICNGFQVLCEAGLLPGALTRNVGLHFICRDVWLRVASTSTAWTSRFEPDADLLVPLKSGEGRYVAPEKVLDELEGEGRVVFRYHDNVNGSLRDIAGICSANGRVVGLMPHPEHAIEALTGPSDDGLGLFYSALDAVLTG

>605|CORE_REP|Org2_Gene2896#

MVSGIGGVVERGLWLPDPAHRADLATFVDHALRLDDAAVIRIRARSTGLLSAWVATGFDVLASRVVAGKVRPDDLSVAARSLAHGLATTDASGYVDPGYSMDSAWRGGLPPESGFTYLDDVPARVMLDLAHRGARLAKEHGSSAGPPVSLLDQEVIQVSSADVVVGLPMRCVFALTAMGFLPQSAETISADELIRVRISPAWLRLDARFGSVYRHRGHAALVLR

>608|CORE_REP|Org26_Gene512#

MAAQEQKTLKIDVKTPAGKVDGAIELPAELFDVPANIALMHQVVTAQRAAARQGTHSTKTRGEVSGGGRKPYRQKGTGRARQGSTRAPQFTGGGVVHGPKPRDYSQRTPKKMIAAALRGALSDRARNGRIHAITELVEGQNPSTKSARAFLASLTERKQVLVVIGRSDEAGAKSVRNLPGVHILAPDQLNTYDVLRADDVVFSVEALNAYIAANTTTSEEVSA

>613|CORE_REP|Org33_Gene2178#

MITVNVLYFGAVREACKVAHEKISLESGTTVDGLVDQLQIDYPPLADFRKRVRMAVNESIAPASTILDDGDTVAFIPQVAGGSDVYCRLTDEPLSVDEVLNAISGPSQGGAVIFVGTVRNNNNGHEVTKLYYEAYPAMVHRTLMDIIEECERQADGVRVAVAHRTGELRIGDAAVVIGASAPHRAAAFDAARMCIERLKQDVPIWKKEFALDGVEWVANRP

>617|CORE_REP|Org59_Gene1254#

MAPDRADDDAERSDEEEWRLMTKLXVASRNRKKLAELRRVLDGAGLSGXTXLSXGDVSPLPETPETGVTFEDNALAKARDAFSATGLASVADDSGLEVAALGGMPGVLSARWSGRYGDDAANTALLLAQLCDVPDERRGAAFVSACALVSGSGEVVVRGEWPGTIAREPRGDGGFGYDPVFVPYGDDRTAAQLSPAEKDAVSHRGRALALLLPALRSLATG

>619|CORE_REP|Org146_Gene531#

MAEQPAGQAGTTDNRDARGDREGRRRDSGRGSRERDGEKSNYLERVVAINRVSKVVKGGRRFSFTALVIVGDGNGMVGVGYGKAKEVPAAIAKGVEEARKSFFRVPLIGGTITHPVQGEAAAGVVLLRPASPGTGVIAGGAARAVLECAGVHDILAKSLGSDNAINVVHATVAALKLLQRPEEVAARRGLPIEDVAPAGMLKARRKSEALAASVLPDRTI

>635|CORE_REP|Org109_Gene2034#

MVKVFLVDDHEVVRRGLVDLLGADPELDVVGEAGSVAEAMARVPAARPDVAVLDVRLPDGNGIELCRDLLSRMPDLRCLILTSYTSDEAMLDAILAGASGYVVKDIKGMELARAVKDVGAGRSLLDNRAAAALMAKLRGAAEKQDPLSGLTDQERTLLGLLSEGLTNKQIADRMFLAEKTVKNYVSRLLAKLGMERRTQAAVFATELKRSRPPGDGP

>636|CORE_REP|Org43_Gene263#

MTISFSSSNLRDDATSGNGDYRLDKLPETTPSTSVFDRADVTYRQFTELHGQARDTRREAHVVELESKTGERARCAPMHALEQLADYGFAWRDIARVVGVSVPAITKWRKGAGVTGENRLKIARLLALIDMLSDRFIGEPASWLEMPIQAGVGITRMDLLERGRYDLVLALASTHTGDGTVEYVLNETDKDWRETVVDNAFESYTAEDGVISIRPKR

>654|CORE_REP|Org34_Gene3162#

MPLFSFEGRSPRIDPTAFVAPTATLIGDVTIEAGASVWFNAVLRGDYAPVVVREGANVQDGAVLHAPPGIPVDIGPGATVAHLCVIHGVHVGSEALIANHATVLDGAVIGARCMIAAGALVVAGTQIPAGMLVTGAPAKVKGPIEGTGAEMWVNVNPQAYRDLAARHLAGLEPMQASLRVKPSAVLTSGCKSRSRRRRRRCPPPHQLGRRVR

>656|CORE_REP|Org4_Gene2108#

MSTPSATVAPVKRIPYAEASRALLRDSVLDAMRDLLLTRDWSAITLSDVARAAGISRQTIYNEFGSRQGLAQGYALRLADRLVDNVHASLDANVGNFYEAFLQGFRSFFAESAADPLVISLLTGVAKPDLLQLITTDSAPIITRASARLAPAFTDTWVATTDNDANVLSRAIVRLCLSYVSMPPEADHDVAADLARLITPFAERHGVINVP

>658|CORE_REP|Org53_Gene1592#

MGGTFDPIHYGHLVAASEVADLFDLDEVVFVPSGQPWQKGRQVSAAEHRYLMTVIATASNPRFSVSRVDIDRGGPTYTKDTLADLHALHPDSELYFTTGADALASIMSWQGWEELFELARFVGVSRPGYELRNEHITSLLGQLAKDALTLVEIPALAISSTDCRQRAEQSRPLWYLMPDGVVQYVSKCRLYCGACDAGARSTTSLAAGNGL

>659|CORE_REP|Org119_Gene1487#

MTTTQTAKASRRARIERRTRESDIVIELDLDGTGQVAVDTGVPFYDHMLTALGSHASFDLTVRATGDVEIEAHHTIEDTAIALGTALGQALGDKRGIRRFGDAFIPDGRNTGPRRRRLIRPPLLRAYRRAGSPCSTPLLPAVQCPTTPSSTRHVFESLAANARIALHVRVLYGRDPHHITEAQYKAVARALRQAVEPDPRVSGVPSTKGAL

>660|CORE_REP|Org27_Gene1940#

MVAARPAERSGDPAAVRVPVPSAWWVLIGGVIGLFASMTLTVEKVRILLDPIYVPSCNVNPIVSCGSVMTTPQASLLGFPNPLLGIAGFTVVVVTGVLAVAKVPLPRWYWIGLAVGILVGVAFVHWLIFQSLYRIGALCPYCMVVWAVIATLLVVVASIVFGPMRENRGSQERVGARLLYQWRWSLATLWFTTVFLLIMVRFWDYWSTLI

>661|CORE_REP|Org9_Gene938#

MTKPTSAGQADDALVRLARERFDLPDQVRRLARPPVPSLEPPYGLRVAQLTDAEMLAEWMNRPHLAAAWEYDWPASRWRQHLNAQLEGTYSLPLIGSWHGTDGGYLELYWAAKDLISHYYDADPYDLGLHAAIADLSKVNRGFGPLLLPRIVASVFANEPRCRRIMFDPDHRNTATRRLCEWAGCKFLGEHDTTNRRMALYALEAPTTAA

>662|CORE_REP|Org59_Gene2553#

MSRSTRYSVAVSAQPETGQIAGRARIANLANILTLLRLXXVPVFLLALFYGGGHHSAARVVAWAIFATACITDRFDGLLARNYGMATEFGAFVDPIADKTLIGSALIGLSMLGDLPWWVTVLILTRELGVTVLRLAVIRRGVIPASWGGKLKTFVQAVAIGLFVLPLSGPLHVAAXVVMAAAILLTVITGVDYVARALRDIGGIRQTAS

>663|CORE_REP|Org7_Gene1062#

MEPVLTQNRVLTVPNMLSVIRLALIPAFVYVVLSAHANGWGVAILVFSGVSDWADGKIARLLNQSSRLGALLDPAVDRLYMVTVPIVFGLSGIVPWWFVLTLLTRDALLAGTLPLLWSRGLSALPVTYVGKAATFGFMVGFPTILLGQCDPLWSHVLLACGWAFLIWGMYAYLWAFVLYAVQMTMVVRQMPKLKGRAHRPAAQNAGERG

>667|CORE_REP|Org28_Gene1370#

MLDYLRDAAEIYRRSFAVIRAEADLARFPADVARVVVRLIHTCGQVDVAEHVAYTDDVVARAGAALAAGAPVLCDSSMVAAGITTSRLPADNQIVSLVADPRATELAARRQTTRSAAGVELCAERLPGAVLAIGNAPTALFRLLELVDEGAPPPAAVLGGPVGFVGSAQAKEELIERPRGMSYLVVRGRRGGSAMAAAAVNAIASDRE

>668|CORE_REP|Org1_Gene3126#

MSVMTGPTTDADAAVPRRVLIAEDEALIRMDLAEMLREEGYEIVGEAGDGQEAVELAELHKPDLVIMDVKMPRRDGIDAASEIASKRIAPIVVLTAFSQRDLVERARDAGAMAYLVKPFSISDLIPAIELAVSRFREITALEGEVATLSERLETRKLVERAKGLLQTKHGMTEPDAFKWIQRAAMDRRTTMKRVAEVVLETLGTPKDT

>669|CORE_REP|Org2_Gene567#

MFVGFVAMMTLKVAIGPQNAFVLRQGIRREYVLVIVALCGIADGALIAAGVGGFAALIHAHPNMTLVARFGGAAFLIGYALLAARNAWRPSGLVPSESGPAALIGVVQMCLVVTFLNPHVYLDTVVLIGALANEESDLRWFFGAGAWAASVVWFAVLGFSAGRLQPFFATPAAWRILDALVAVTMIGVAVVVLVTSPSVPTANVALII

>670|CORE_REP|Org86_Gene1143#

MAAPDNSRRRPGRPAGSSDTRERILSSARELFAHNGIDRTSIRAVAAKAGVDAALVHHYFGTKQQLFAAAIHIPIDPMVIIGPIREAPVEELGYKLPSLLLPIWDSELGAGLIATLRSLISGSDVGLARSFLEEVVTVELGSRVDNPPGTGKIRTQFVASQLMGVVMARYIVRIEPFASLPAEQIVQTIAPNLQRYLTGELPDDLAP

>672|CORE_REP|Org102_Gene1803#

MPQGNPLAVPNDGLTTRARRNMPILAVHTGEGKGKSTAAFGMALRAWNAGLDIAVFQFVKSAKWKVGEEAAFRQLGRLHDQHGIGGAVEWHKMGAGWSWTRTSRKAGTDVDRAAAAADGWAEIALRLATQRHDFYLLDEFTYPLKWGWLDVDEVVDVLRARPGHQHVVITGRDAPQRLVAAADLVTEMTKVKHPMDAGRKGQKGIEW

>676|CORE_REP|Org61_Gene841#

MSGDGLVRCPWAEVRPGPDAQLYRDYHDNEWGRPLYGRVALFERMSLEAFQSGLSWLIILRKRENFRRAFSGFDIDKIARYTDTDVRRLLADDGIVRNRAKIEATIANARAAADLGSSEDLSELLWSFAPPPRPRPVDGSEIPSVSTESKAMSRELKRRGFRFVGPTTAYALMQATGMVDDHIQACWVPTERPFDQPGCPMAAR

>678|CORE_REP|Org139_Gene2426#

MFEGPVQDLIDELGKLPGIGPKSAQRIAFHLLSVEPSDIDRLTGVLAKVRDGVRFCAVCGNVSDNERCRICSDIRRDASVVCIVEEPKDIQAVERTREFRGRYHVLGGALDPLSGIGPDQLRIRELLSRIGERVDDVDVTEVIIATDPNTEGEATATYLVRMLRDIPGLTVTRIASGLPMGGDLEFADELTLGRALAGRRVLA

>680|CORE_REP|Org25_Gene1456#

MTSAVGTSGTAITSRVHSLNRPNMVSVGTIVWLSSELMFFAGLFAFYFSARAQAGGNWPPPPTELNLYQAVPVTLVLIASSFTCQMGVFAAERGDIFGLRRWYVITFLMGLFFVLGQAYEYRNLMSHGTSIPSSAYGSVFYLATGFHGLHVTGGLIAFIFLLVRTGMSKFTPAQATASIVVSYYWHFVDIVWIALFTVIYFIR

>684|CORE_REP|Org21_Gene1049#

MPDEPTGSADPLTSTEEAGGAGEPNAPAPPRRLRMLLSVAVVVLTLDIVTKVVAVQLLPPGQPVSIIGDTVTWTLVRNSGAAFSMATGYTWVLTLIATGVVVGIFWMGRRLVSPWWALGLGMILGGAMGNLVDRFFRAPGPLRGHVVDFLSVGWWPVFNVADPSVVGGAILLVILSIFGFDFDTVGRRHADGDTVGRRKADG

>686|CORE_REP|Org120_Gene3695#

MKARELDVPGAWEITPTIHVDSRGLFFEWLTDHGFRAFAGHSLDVRQVNCSVSSAGVLRGLHFAQLPPSQAKYVTCVSGSVFDVVVDIREGSPTFGRWDSVLLDDQDRRTIYVSDGLAHGFLALQDNSTVMYLCSAEYNPQREHTICATDPTLAVDWPLVDGAAPSLSDRDAAAPSFEDVRASGLLPRWEQTQRFIGEMRGT

>690|CORE_REP|Org39_Gene973#

MFTGIVEERGEVTGREALVDAARLTIRGPMVTADAGHGDSIAVNGVCLTVVDVLPDGQFTADVMAETLNRSNLGELRPGSRVNLERAAALGSRLGGHIVQGHVDATGEIVARCPSEHWEVVRIEMPASVARYVVEKGSITVDGISLTVSGLGAEQRDWFEVSLIPTTRELTTLGSAAVGTRVNLEVDVVAKYVERLMRSAG

>692|CORE_REP|Org137_Gene3434#

MSTETRVNERIRVPEVRLIGPGGEQVGIVRIEDALRVAADADLDLVEVAPNARPPVCKIMDYGKYKYEAAQKARESRRNQQQTVVKEQKLRPKIDDHDYETKKGHVVRFLEAGSKVKVTIMFRGREQSRPELGYRLLQRLGADVADYGFIETSAKQDGRNMTMVLAPHRGAKTRARARHPGEPAGGPPPKPTAGDSKAAPN

>696|CORE_REP|Org10_Gene3899#

MTECFLSDQEIRKLNRDLRILIAANGTLTRVLNIVADDEVIVQIVKQRIHDVSPKLSEFEQLGQVGVGRVLQRYIILKGRNSEHLFVAAESLIAIDRLPAAIITRLTQTNDPLGEVMAASHIETFKEEAKVWVGDLPGWLALHGYQNSRKRAVARRYRVISGGQPIMVVTEHFLRSVFRDAPHEEPDRLQFSNAITLAR

>698|CORE_REP|Org41_Gene1966#

MEAFHTHSGIGVPLRRSNVDTDQIIPAVFLKRVTRTGFEDGLFAGWRSDPAFVLNLSPFDRGSVLVAGPDFGTGSSREHAVWALMDYGFRVVISSRFGDIFRGNAGKAGLLAAEVAQDDVELLWKLIEQSPGLEITANLQDRIITAATVVLPFKIDDHSAWRLLEGLDDIALTLRKLDEIEAFEGACAYWKPRTLPAP

>703|CORE_REP|Org1_Gene1468#

MTVTDDYLANNVDYASGFKGPLPMPPSKHIAIVACMDARLDVYRMLGIKEGEAHVIRNAGCVVTDDVIRSLAISQRLLGTREIILLHHTDCGMLTFTDDDFKRAIQDETGIRPTWSPESYPDAVEDVRQSLRRIEVNPFVTKHTSLRGFVFDVATGKLNEVTPSSPSRQPRAHWRTGSPPRWGCVDSDREAWLHRWQ

>710|CORE_REP|Org1_Gene3698#

MQWGYHPRAGDEAMRRSGAYDSCRRAAPGRVIMGSDSDWPVMADAAAALAEFDIPAEVRVVSAHRTPEAMFSYARGAAERGLEVIIAGAGGAAHLPGMVAAATPLPVIGVPVPLGRLDGLDSLLSIVQMPAGVPVATVSIGGAGNAGLLAVRMLGAANPQLRARIVAFQDRLADVVAAKDAELQRLAGKLTRD

>725|CORE_REP|Org62_Gene2308#

MSAQIDPRTFRSVLGQFCTGITVITTVHDDVPVGFACQSFAALSLEPPLVLFCPTKVSRSWQAIEASGRFCVNVLTEKQKDVSARFGSKEPDKFAGIDWRPSELGSPIIEGSLAYIDCTVASVHDGGDHFVVFGAVESLSEVPAVKPRPLLFYRGDYTGIEPEKTTPAHWRDDLEAFLTTTTQDTWL

>728|CORE_REP|Org86_Gene524#

MTTAQKVQPRLKERYRSEIRDALRKQFGYGNVMQIPTVTKVVVNMGVGEAARDAKLINGAVNDLALITGQKPEVRRARKSIAQFKLREGMPVGVRVTLRGDRMWEFLDRLTSIALPRIRDFRGLSPKQFDGVGNYTFGLAEQAVFHEVDVDKIDRVRGMDINVVTSAATDDEGRALLRALGFPFKEN

>735|CORE_REP|Org113_Gene1014#

MTGPYFPQTIPFLPSYIPQDVDMTAVKAEVAALGVSAPPAATPGLLEVVQHARDEGIDLKIVLLDHNPPNDTPLRDIATVVGADYSDATVLVLSPNYVGSYSTQYPRVTLEAGEDHSKTGNPVQSAQNFVHELSTPEFPWSALTIVLLIGVLAAAVGARLMQLRGRRSATSTDAAPGAGDDLNQGV

>737|CORE_REP|Org125_Gene2739#

MPGNDWIVGGNRRTIAAERIYAAATDLITRYGLNALDIDKLAREVHCSRATIYRRAGGKAQIRDVVLTRAAARIADGVRSDVETLRGRERVVAAILLSLQRIRSDPLGKLMFGSIHGGAGELAWLTESPLLADFATELTGIAGGDPQGAKWVVRVVLSLMYWPAENDEAERRLVEKYVAPAFAEQS

>740|CORE_REP|Org29_Gene1887#

MIDEALFDAEEKMEKAVAVARDDLSTIRTGRANPGMFSRITIDYYGAATPITQLASINVPEARLVVIKPYEANQLRAIETAIRNSDLGVNPTNDGALIRVAVPQLTEERRRELVKQAKHKGEEAKVSVRNIRRKAMEELHRIRKEGEAGEDEVGRAEKDLDKTTHQYVTQIDELVKHKEGELLEV

>745|CORE_REP|Org118_Gene3031#

MSSPVSSRRLANLVKESLQGSVLGGVVSDAVLPAVSDDVKPGAGEDAYRVPVVVAAGSGAVVQVGGLEVGSAAVAGEVADTVAELFVCRPTEPDVGDFVGLAGGAGDAGQAGQQFGLGVGVRGESFGARRRSLALSTVGASGATAGLRKTHDGHHGCQARGALTQRRLYIGNPSEITDTRMVHQ

>762|CORE_REP|Org119_Gene660#

MSRIGKQPIPVPAGVDVTIEGQSISVKGPKGTLGLTVAEPIKVARNDDGAIVVTRPDDERRNRSLHGLSRTLVSNLVTGVTQGYTTKMEIFGVGYRVQLKGSNLEFALGYSHPVVIEAPEGITFAVQAPTKFTVSGIDKQKVGQIAANIRRLRRPDPSKRGKGVRYEGEQIRRKVGKTGK

>764|CORE_REP|Org98_Gene289#

MAGPDRAELAELVRRLSVVHGRVTLSSGREADYYVDLRRATLHHRASALIGRLMRELTADWDYSVVGGLTLGADPVATAIMHAPGRPIDAFVVRKSAKAHGMQRLIEGSEVTGQRVLVVEDTSTTGNSALTAVHAVQDVGGEVVGVATVVDRATGAAEAIEAEGLRYRSVLGLADLGLD

>767|CORE_REP|Org15_Gene2894#

MRKRMVIGLSTGSDDDDVEVIGGVDPRLIAVQENDSDESSLTDLVEQPAKVMRIGTMIKQLLEEVRAAPLDEASRNRLRDIHATSIRELEDGLAPELREELDRLTLPFNEDAVPSDAELRIAQAQLVGWLEGLFHGIQTALFAQQMAARAQLQQMRQGALPPGVGKSGQHGHGTGQYL

>771|CORE_REP|Org82_Gene3889#

MGTCPCESSERNEPVSRVSGTNEVSDGNETNNPAEVSDGNETNNPAEVSDGNETNNPAPVSRVSGTNEVSDGNETNNPAPVSRVSGTNEVSDGNETNNPAPVTEKPLHPHEPHIEILRGQPTDQELAALIAVLGSISGSTPPAQPEPTRWGLPVDQLRYPVFSWQRITLQEMTHMRR

>774|CORE_REP|Org96_Gene1910#

MELVVGRVVKSHGVTGEVVVEIRTDDPADRFAPGTRLRAKGPFDGGAEGSAVSYVIESVRQHGGRLLVRLAGVADRDAADALRGSLFVIDADDLPPIDEPDTYYDHQLVGLMVQTATGEGVGVVTEVVHTAAGELLAVKRDSDEVLVPFVRAIVTSVSLDDGIVEIDPPHGLLNLE

>777|CORE_REP|Org59_Gene1518#

MSAYKTVVVGTDGSDSSMRAVDRAAQIAGADAKLIIASAYLPQHEDARAADILKDESYKVTGTAPIYEILHDAKERAHNAGAKNVEERPIVGAPVDALVNLADEEKADLLVVGNVGLSTIAGRLLGSVPANVSRRAKVDVLIVAAPPSGRYQPRARHSLRLGRSAPSSVSSPWPG

>780|CORE_REP|Org36_Gene1822#

MTERPRDCRPVVRRARTSDVPAIKQLVDTYAGKILLEKNLVTLYEAVQEFWVAEHPDLYGKVVGCGALHVLWSDLGEIRTVAVDPAMTGHGIGHAIVDRLLQVARDLQLQRVFVLTFETEFFARHGFTEIEGTPVTAEVFDEMCRSYDIGVAEFLDLSYVKPNILGNSRMLLVL

>787|CORE_REP|Org13_Gene407#

MISPKPLLHILIHGRSDELPDTRGRIVLRWLRIAVLIVTGLVTLQSVLLVAGAWRNDIAIQRNMGVAQAEVLSAGPRRSTIEFVTPDRITYRPQLGVLYPSELSTGMRIYVEYNKRDPNLVRVQHRNAGLAIIPAGSIAVVAWLIAAAALVVLAVLDKRLERRENSASATG

>790|CORE_REP|Org56_Gene2653#

MGEVSAIVLAASQAAEEGGESSNFLIPNGTFFVVLAIFLVVLAVIGTFVVPPILKVLRERDAMVAKTLADNKKSDEQFAAAQADYDEAMTEARVQASSLRDNARADGRKVIEDARVRAEQQVASTLQTAHEQLKRERDAVELDLRAHVGTMSATLASRILGVDLTASAATR

>799|CORE_REP|Org143_Gene2241#

MSREGIRRRPKARAGLTGGGTATLPRVEDTLTLGSRLGEQLCAGDVVVLSGPLGAGKTVLAKGIAMAMDVEGPITSPTFVLARMHRPRRPGTPAMVHVDVYRLLDHNSADLLSELDSLDLDTDLEDAVVVVEWGEGLAERLSQRHLDVRLERVSHSDTRIATWSWGRS

>800|CORE_REP|Org12_Gene548#

METLLKTSEAAQILGVSRQHVVNMCDRGEMVCVHVGSHRRVPSSEVERVTSRRLTREEERSLWLHRALLSPLLTEPDTVVSAARENLRRWSGMHRRDGMAGWYFTKWQRVLNDGLDAVMHVLTSPSEDAREMRQNSPFAGILPEATRVAVLRSFKDHWDREHERAMTE

>804|CORE_REP|Org2_Gene4061#

MARLVGVDLPRDKRMEVALTYIFGIGRTRSNEILAATGIDRDLRTRDLTEEQLIHLRDYIEANLKVEGDLRREVQADIRRKIEIGCYQGLRHRRGMPVRGQRTKPTRGPAKAPSAPSQARRRLGNRCHQQKKGRQRRLGRARRPAGGRRRTSRTAPPTSRARSTTRS

>808|CORE_REP|Org117_Gene1268#

MAQITLRGNAINTVGELPAVGSPAPAFTLTGGDLGVISSDQFRGKSVLLNIFPSVDTPVCATSVRTFDERAAASGATVLCVSKDLPFAQKRFCGAEGTENVMPASAFRDSFGEDYGVTIADGPMAGLLARAIVVIGADGNVAYTELVPEIAQEPNYEAALAALGA

>812|CORE_REP|Org135_Gene2632#

MNSGTLAGSLIFAAVLVMLIAVLARLMMRGWRRRSERQAELLGDLPDVPEHVSSATVTTRGLYVGATLSPAWNERVTVGDLGYRSKAVLTRYPSGIMVERARAQPIWIPTESIAAIRMERGVAGKVVAGIGILAIRWRLPSGTEIDVGFRADNRDEYQEWLEEPV

>816|CORE_REP|Org140_Gene763#

MTDTQVTWLTQESHDRLKAELDQLIANRPVIAAEINDRREEGDLRENGGYHAAREEQGQQEARIRQLQDLLSNAKVGEAPKQSGVALPGSVVKVYYNGDKSDSETFLIATRQEGVSDGKLEVYSPNSPLGGALIDAKVGETRSYTVPNGSTVSVTLVSAEPYHS

>821|CORE_REP|Org25_Gene421#

MADSSFDIVSKVDRQEVDNALNQAAKELATRFDFRGTDTKIAWKGDEAVELTSSTEERVKAAVDVFKEKLIRRDISLKAFEAGEPQASGKTYKVTGALKQGISSENAKKITKLIRDAGPKNVKTQIQGDEVRVTSKKRDDLQAVIAMLKKADLDVALQFVNYR

>823|CORE_REP|Org119_Gene3352#

MQFDVTIEIPKGQRNKYEVDHETGRVRLDRYLYTPMAYPTDYGFIEDTLGDDGDPLDALVLLPQPVFPGVLVAARPVGMFRMVDEHGGDDKVLCVPAGDPRWDHVQDNPGTFRLSSWMRSSISLWHYKDLEPGKFVKAADWVDRAEAEAEVQRSVERFKAGTH

>828|CORE_REP|Org68_Gene1008#

MTLRLEQIYQDVILDHYKHPQHRGLREPFGAQVYHVNPICGDEVTLRVALSEDGTRVTDVSYDGQGCSISQAATSVLTEQVIGQRVPRALNIVDAFTEMVSSRGTVPGDEDVLGDGVAFAGVAKYPARVKCALLGWMAFKDALAQASEAFEEVTDERNQRTG

>829|CORE_REP|Org1_Gene4076#

MIFKVGDTVVYPHHGAALVEAIETRTIKGEQKEYLVLKVAQGDLTVRVPAENAEYVGVRDVVGQEGLDKVFQVLRAPHTEEPTNWSRRYKANLEKLASGDVNKVAEVVRDLWRRDQERGLSAGEKRMLAKARQILVGELALAESTDDAKAETILDEVLAAAS

>832|CORE_REP|Org89_Gene146#

MTAISCSPRPRYASRMPVLSKTVEVTADAASIMAIVADIERYPEWNEGVKGAWVLARYDDGRPSQVRLDTAVQGIEGTYIHAVYYPGENQIQTVMQQGELFAKQEQLFSVVATGAASLLTVDMDVQVTMPVPEPMVKMLLNNVLEHLAENLKQRAEQLAAS

>833|CORE_REP|Org36_Gene1960#

MTGAVCPGSFDPVTLGHVDIFERAAAQFDEVVVAILVNPAKTGMFDLDERIAMVKESTTHLPNLRVQVGHGLVVDFVRSCGMTAIVKGLRTGTDFEYELQMAQMNKHIAGVDTFFVATAPRYSFVSSSLAKEVAMLGGDVSELLPEPVNRRLRDRLNTERT

>834|CORE_REP|Org28_Gene1772#

MSGTRLAPHSVRYRERLWVPWWWWPLAFALAALIAFEVNLGVAALPDWVPFATLFTVAAGTLLWLGRVEIRVTAGSADGAGVKLWAGPAHLPVAVIARSAEIPATAKSAALGRQLDPAAYVLHRAWVGPMVLVVLDDPNDPTPYWLVSCRHPERVLSALRS

>835|CORE_REP|Org96_Gene466#

MSDEGDVADEAVADGAENADSRGSGGRTALVTKPVVRPQRPTGKRSRSRAAGADADVDVEEPSTAASEATGVAKDDSTTKAVSKAARAKKASKPKARSVNPIAFVYNYLKQVVAEMRKVIWPNRKQMLTYTSVVLAFLAFMVALVAGADLGLTKLVMLVFG

>837|CORE_REP|Org44_Gene2040#

MSKSSRGGRQIVASNRKARHNYSIIEVFEAGVALQGTEVKSLREGQASLADSFATIDDGEVWLRNAHIPEYRHGSWTNHEPRRNRKLLLHRRQIDTLVGKIREGNFALVPLSLYFAEGKVKVELALARGKQARDKRQDMARRDAQREVLRELGRRAKGMT

>839|CORE_REP|Org88_Gene2659#

MKGGAGVPDLPSLDASGVRLAIVASSWHGKICDALLDGARKVAAGCGLDDPTVVRVLGAIEIPVVAQELARNHDAVVALGVVIRGQTPHFDYVCDAVTQGLTRVSLDSSTPIANGVLTTNTEEQALDRAGLPTSAEDKGAQATVAALATALTLRELRAHS

>847|CORE_REP|Org58_Gene464#

MALSADIVGMHYRYPDHYEVEREKIREYAVAVQNDDAWYFEEDGAAELGYKGLLAPLTFICVFGYKAQAAFFKHANIATAEAQIVQVDQVLKFEKPIVAGDKLYCDVYVDSVREAHGTQIIVTKNIVTNEEGDLVQETYTTLAGRAGEDGEGFSDGAA

>852|CORE_REP|Org101_Gene1661#

MTKTTRLTPGDKAPAFTLPDADGNNVSLADYRGRRVIVYFYPAASTPGCTKQACDFRDNLGDFTTAGLNVVGISPDKPEKLATFRDAQGLTFPLLSDPDREVLTAWGAYGEKQMYGKTVQGVIRSTFVVDEDGKIVVAQYNVKATGHVAKLRRDLSV

>858|CORE_REP|Org118_Gene615#

MAPKKKVAGLIKLQIVAGQANPAPPVGPALGQHGVNIMEFCKAYNAATENQRGNVIPVEITVYEDRSFTFTLKTPPAAKLLLKAAGVAKGSAEPHKTKVAKVTWDQVREIAETKKTDLNANDVGPERPRSSPVPLGRWASPSNRALPVGGPASAR

>862|CORE_REP|Org79_Gene1778#

MSTTLAIVRLDPGLPLPSRAHDGDAGVDLYSAEDVELAPGRRALVRTGVAVAVPFGMVGLVHPRSGLATRVGLSIVNSPGTIDAGYRGEIKVALINLDPAAPIVVHRGDRIAQLLVQRVELVELVEVSSFDEAGLASTSRGDGGHGSSGGHASL

>868|CORE_REP|Org133_Gene119#

MRTFESVADLAAAAGEKVGQSDWVTITQEEVNLFADATGDHQWIHVDPERAAAGPFGTTIAHGFMTLALLPRLQHQMYTVKGVKLAINYGLNKVRFPAPVPVGSRVRATSSLVGVEDLGNGTVQATVSTTVEVEGSAKPACVAESIVRYVA

>872|CORE_REP|Org57_Gene2255#

MTETTPAPQTPAAPAGPAQSFVLERPIQTVGRRKEAVVRVRLVPGTGKFDLNGRSLEDYFPNKVHQQLIKAPLVTVDRVESFDIFAHLGGGGPSGQAGALRLGIARALILVSPEDRPALKKAGFLTRDPRATERKKYGLKKARKAPQYSKR

>875|CORE_REP|Org118_Gene2056#

MNSIQIADETYVAADAARVSAAVADRCSWRRWWPDLRLQVTEDRADKGIRWTVTGALTGTMEIWLEPSMDGVLLHYFLHAEPTGVAAWQLARMNLARMTAPPTGRGQKNGLRGQDSARTVTPHRGFSGNLTGSRLARGSTVSPREIGSSRR

>876|CORE_REP|Org111_Gene1999#

MDIAGRSLVYFSSVSENTHRFVQKLGIPATRIPLHGRIEVDEPYVLILPTYGGGRANPGLDAGGYVPKQVIAFLNNDHNRAQLRGVIAAGNTNFGAEFCYAGDVVSRKCSVPYLYRFELMGTEDDVAAVRTGLAEFWKEQTCHQPSLQSL

>883|CORE_REP|Org106_Gene334#

MSRLSSILRAGAAFLVLGIAAATFPQSAAADSTEDFPIPRRMIATTCDAEQYLAAVRDTSPVYYQRYMIDFNNHANLQQATINKAHWFFSLSPAERRDYSEHFYNGDPLTFAWVNHMKIFFNNKGVVAKGTEVCNGYPAGDMSVWNWA

>904|CORE_REP|Org77_Gene774#

MTHTPIPRPDARYGRPRLSRRARRRVAIALGVLVAAAGIVIAVIGYQRISTSAVTGSLVGYRLVDDETASVTISVTRSDPSRPVACIVRVRATNGSETGRRELLVPPSEATTVQVTTTVKSSQPPVMADVYGCGTEVPSYLRLP

>916|CORE_REP|Org1_Gene4305#

MIRSESGAAPPRQHLHLSAQVMRFVVTGGLAGIVDFGLYVVLYKVAGLQVDLSKAISFIVGTITAYLINRRWTFQAEPSTARFVAVMLLYGITFAVQVGLNHLCLALLHYRAWAIPVAFVIAQGTATVINFIVQRAVIFRIR

>923|CORE_REP|Org59_Gene380#

MSYAGDITPLQAWEMLSDNPRAVLVDVRCEAEWRFVGVPDLSSLGREVVYVEWATSDGTHNDNFLAELRDRIPADADQHEXXXXFILCRSGNRSIGAAEVATEAGITPAYNVLDGFEGHLDAEGHRGATGWRAVGLPWRQG

>932|CORE_REP|Org135_Gene2330#

MLRTMLKSKIHRATVTCADLHYVGSVTIDADLMDAADLLEGEQVTIVDIDNGARLVTYAITGERGSGVIGINGAAAHLVHPGDLVILIAYATMDDARARTYQPRIVFVDAYNKPIDMGHDPAFVPENAGELLDPRLGVG

>938|CORE_REP|Org101_Gene527#

MLIPRKVKHRKQHHPRQRGIASGGTTVNFGDYGIQALEHAYVTNRQIESARIAINRHIKRGGKVWINIFPDRPLTKKPAETRMGSGKGSPEWWVANVKPGRVLFELSYPNEGVARAALTRAIHKLPIKARIITREEQF

>962|CORE_REP|Org83_Gene3312#

MSDIPSDLHYTAEHEWIRRSGDDTVRVGITDYAQSALGDVVFVQLPVIGTAVTAGETFGEVESTKSVSDLYAPISGKVSEVNSDLDGTPQLVNSDPYGAGWLLDIQVDSSDVAALESALTKLLDAEAYRGTLTE

>964|CORE_REP|Org119_Gene3335#

MADRIELRGLTVHGRHGVYDHERVAGQRFVIDVTVWIDLAEAANSDDLADTYDSPRAGISGAAEIVAGPPRKLIETVGAEIADHVMDDQRVHAVEVAVHKPQAPIPQTFDDVAVVIRRSRRGGRGWVVPAGGAV

>973|CORE_REP|Org27_Gene529#

MTMTDPIADFLTRLRNANSAYHDEVSLPHSKLKANIAQILKNEGYISDFRTEDARVGKSLVIQLKYGPSRERSIAGLRRVSKPGLRVYAKSTNLPRVLGGLGVAIISTSSGLLTDRQAARQGVGGEVLAYVW

>999|CORE_REP|Org102_Gene1073#

MARVKRAVNAHKKRRSILKASRGYRGQRSRLYRKAKEQQLHSLNYAYRDRRARKGEFRKLWIARINAAARLNDITYNRLIQGLKAAGVEVDRKNLADIAISDPAAFTALVDVARAALPEDVNAPSGEAA

>1010|CORE_REP|Org73_Gene1288#

MKTARLQVTLRCAVDLINSSSDQCFARIEHVASDQADPRPGVWHSSGMNRIRLSTTVDAALLTSARDMRAGITDAALIDEALAALLARHRSAEVDASYAAYDKHPVDEPDEWGDLASWRRAAGDS

>1019|CORE_REP|Org134_Gene118#

MTKKPRNPADYVIGDDVEVSDVDLKQEEVYVDGERLTDERVEQMASESLRLAREREANLIPGGKSLSGGSAHSPAVQVVVSKATHAKLKELARSRKMSVSKLLRPVLDEFVQRETGRILPRR

>1022|CORE_REP|Org115_Gene528#

MAQSVSATRRISRLRRHTRLRKKLSGTAERPRLVVHRSARHIHVQLVNDLNGTTVAAASSIEADVRGVPGDKKARSVRVGQLIAERAKAAGIDTVVFDRGGYTYGGRIAALADAARENGLSF

>1024|CORE_REP|Org53_Gene521#

MIQQESRLKVADNTGAKEILCIRVLGGSSRRYAGIGDVIVATVKDAIPGGNVKRGDVVKAVVVRTVKERRRPDGSYIKFDENAAVIIKPDNDPRGTRIFGPVGRELREKRFMKIISLAPEVL

>1026|CORE_REP|Org127_Gene917#

MAELNVEIVAVDRNIWSGTAKFLFTRTTVGEIGILPRHIPLVAQLVDDAMVRVEREGEKDLRIAVDGGFLSVTEEGVSILAESAEFESEIDEAAAKQDSESDDPRIAARGRARLRAVGAID

>1032|CORE_REP|Org63_Gene939#

MTAPETPAAQHAEPAIAVERIRTALLGYRIMAWTTGLWLIALCYEIVVRYVVKVDNPPTWIGVVHGWVYFTYLLLTLNLAVKVRWPLGKTAGVLLAGTIPLLGIVVEHFQTKEIKARFGL

>1040|CORE_REP|Org12_Gene1447#

MTVQNEPSAKTHGVILTEAAAAKAKSLLDQEGRDDLALRIAVQPGGCAGLRYNLFFDDRTLDGDQTAEFGGVRLIVDRMSAPYVEGASIDFVDTIEKQGFTIDNPNATGSCACGDSFN

>1052|CORE_REP|Org135_Gene38#

MTDSEKSATIKVTDASFATDVLSSNKPVLVDFWATWCGPCKMVAPVLEEIATERATDLTVAKLDVDTNPETARNFQVVSIPTLILFKDGQPVKRIVGAKGKAALLRELSDVVPNLN

>1055|CORE_REP|Org96_Gene1012#

MSETSAPAEELLADVEEAMRDVVDPELGINVVDLGLVYGLDVQDGDEGTVALIDMTLTSAACPLTDVIEDQSRSALVGSGLVDDIRINWVWNPPWGPDKITEDGREQLRALGFTV

>1056|CORE_REP|Org96_Gene1491#

MPVTQEEIIAGIAEIIEEVTGIEPSEITPEKSFVDDLDIDSLSMVEIAVQTEDKYGVKIPDEDLAGLRTVGDVVAYIQKLEEENPEAAQALRAKIESENPDAVANVQARLEAESK

>1058|CORE_REP|Org44_Gene1330#

MTYVIGSECVDVMDKSCVQECPVDCIYEGARMLYINPDECVDCGACKPACRVEAIYWEGDLPDDQHQHLGDNAAFFHQVLPGRVAPLGSPGGAAAVGPIGVDTPLVAAIPVECP

>1060|CORE_REP|Org76_Gene1314#

MGVNVLASTVSGAIERLGLTYEEVGDIVDASPRSVARWTAGQVVPQRLNKQRLIELAYVADALAEVLPRDQANVWMFSPNRLLEHRKPADLVRDGEYQRVLALIDAMAEGVFV

>1061|CORE_REP|Org147_Gene1881#

MNRLDFVDKPSLRDDIPAFNPGDTINVHVKVIEGAKERLQVFKGVVIRRQGGGIRETFTVRKESYGVGVERTFPVHSPNIDHIEVVTRGDVRRAKLYYLRELRGKKAKIKEKR

>1064|CORE_REP|Org46_Gene2352#

MAKKVTVTLVDDFDGSGAADETVEFGLDGVTYEIDLSTKNATKLRGDLKQWVAAGRRVGGRRRGRSGSGRGRGAIDREQSAAIREWARRNGHNVSTRGRIPADVIDAYHAAT

>1067|CORE_REP|Org42_Gene976#

MSISQSDASLAAVPAVDQFDPSSGASGGYDTPLGITNPPIDELLDRVSSKYALVIYAAKRARQINDYYNQLGEGILEYVGPLVEPGLQEKPLSIALREIHADLLEHTEGE

>1070|CORE_REP|Org2_Gene2044#

MTSELSLVATGKGSNIMCGDQSDHVLQHWTVDISIDEHEGLTRAKARLRWREKELVGVGLARLNPADRNVPEIGDELSVARALSDLGKRMLKVSTHDIEAVTHQPARLLY

>1078|CORE_REP|Org118_Gene1118#

MEGDGHEVFFVTTRPDSIGETAANLHEIGVTMSAHDDGVTPLITNVESPAHDLVSIVTSMLFSMHGELYKAIARQAHVIHESFVQTLQTSKTSYWLTELANRAGTST

>1083|CORE_REP|Org104_Gene516#

MKVHKGDTVLVISGKDKGAKGKVLQAYPDRNRVLVEGVNRIKKHTAISTTQRGARSGGIVTQEAPIHVSNVMVVDSDGKPTRIGYRVDEETGKRVRISKRNGKDI

>1098|CORE_REP|Org148_Gene321#

MDSAMARAIRSGDDAEVADGLTRREHDILAFERQWWKFAGVKEEAIKELFSMSATRYYQVLNALVDRPEALAADPMLVKRLRRLRASRQKARAARRLGFEVT

>1105|CORE_REP|Org123_Gene512#

MAGQKIRIRLKAYDHEAIDASARKIVETVVRTGASVVGPVPLPTEKNVYCVIRSPHKYKDSREHFEMRTHKRLIDIIDPTPKTVDALMRIDLPASVDVNIQ

>1109|CORE_REP|Org58_Gene1232#

MRLTPHEQERLLLSYAAELARRRRARGLRLNHPEAIAVIADHILEGARDGRTVAELMASGREVLGRDDVMEGVPEMLAEVQVEATFPDGTKLVTVHQPIA

>1115|CORE_REP|Org26_Gene2228#

MAKVNIKPLEDKILVQANEAETTTASGLVIPDTAKEKPQEGTVVAVGPGRWDEDGEKRIPLDVAEGDTVIYSKYGGTEIKYNGEEYLILSARDVLAVVSK

>1130|CORE_REP|Org107_Gene221#

MSQIMYNYPAMLGHAGDMAGYAGTLQSLGAEIAVEQAALQSAWQGDTGITYQAWQAQWNQAMEDLVRAYHAMSSTHEANTMAMMARDTAEAAKWGG

>1141|CORE_REP|Org59_Gene1973#

MQQSLAVKTFEDLFAELXCDRARXRXADSTTVAALDGGVHALGKKLLEEAGEVWLXXEXESNDALAEEISQLLYWTQVLMISRGLSJDDVYRKL

>1142|CORE_REP|Org1_Gene809#

MPRSLKKGPFVDEHLLKKVDVQNEKNTKQVIKTWSRRSTIIPDFIGHTFAVHDGRKHVPVFVTESMVGHKLGEFAPTRTFKGHIKDDRKSKRR

>1154|CORE_REP|Org57_Gene1830#

MALTAEQKKEILRSYGLHETDTGSPEAQIALLTKRIADLTEHLKVHKHDHHSRRGLLLLVGRRRRLIKYISQIDVERYRSLIERLGLRR

>1163|CORE_REP|Org9_Gene1586#

MAHKKGASSSRNGRDSAAQRLGVKRYGGQVVKAGEILVRQRGTKFHPGVNVGRGGDDTLFAKTAGAVEFGIKRGRKTVSIVGSTTA

>1164|CORE_REP|Org80_Gene2849#

MANIKSQQKRNRTNERARLRNKAVKSSLRTAVRAFREAAHAGDKAKAAELLASTNRKLDKAASKGVIHKNQAANKKSALAQALNKL

>1183|CORE_REP|Org48_Gene919#

MDPTIAAGALIGGGLIMAGGAIGAGIGDGVAGNALISGVARQPEAQGRLFTPFFITVGLVEAAYFINLAFMALFVFATPVK

>1188|CORE_REP|Org132_Gene902#

MKSDIHPAYEETTVVCGCGNTFQTRSTKPGGRIVVEVCSQCHPFYTGKQKILDSGGRVARFEKRYGKRKVGADKAVSTGK

>1194|CORE_REP|Org105_Gene1998#

MTVTVYTKPACVQCSATSKALDKQGIAYQKVDISLDSEARDYVMALGYLQAPVVVAGNDHWSGFRPDRIKALAGAALTA

>1210|CORE_REP|Org2_Gene3287#

MTRKMTATEVKAKILSLLDEVAQGEEIEITKHGRTVARLVAATGPHALKGRFSGVAMAAVDDDELFTTGVSWNVS

>1214|CORE_REP|Org69_Gene2244#

MAKKDGAIEVEGRVVEPLPNAMFRIELENGHKVLAHISGKMRQHYIRILPEDRVVVELSPYDLSRGRIVYRYK

>1224|CORE_REP|Org66_Gene300#

MIVVVNEQQVEVDEQTTIAALLDSLGFGDRGIAVALNFSVLPRSDWATKICELRKPVRLEVVTAVQGG

>1228|CORE_REP|Org19_Gene440#

MSQLKITQVRSTIGARWKQRESLRTLGLRRIRHSVIREDNAATRGLIAVVRHLVEVEPAQTGGKT

>1232|CORE_REP|Org147_Gene1378#

MAQEQTKRGGGGGDDDDIAGSTAAGQERREKLTEETDDLLDEIDDVLEENAEDFVRAYVQKGGQ
