# Supplementary material for: Pangenome Analysis of Mycobacterium tuberculosis Reveals Core-Drug Targets and Screening of Promising Lead Compounds for Drug Discovery
Source: Antibiotics (Basel). 2020 Nov 17;9(11):819. doi: 10.3390/antibiotics9110819 (PMC7698547; doi:10.3390/antibiotics9110819)
Supplement: Supplementary file 1 [file antibiotics-09-00819-s001.zip › antibiotics-984987-supplementary/File S3.docx]

**File S3.** Projected virulence-associated proteins from the non-homologous essential core proteins given in FASTA format below:

>3|CORE_REP|Org58_Gene4039#

MNFSVLPPEINSALIFAGAGPEPMAAAATAWDGLAMELASAAASFGSVTSGLVGGAWQGASSSAMAAAAAPYAAWLAAAAVQAEQTAAQAAAMIAEFEAVKTAVVQPMLVAANRADLVSLVMSNLFGQNAPAIAAIEATYEQMWAADVSAMSAYHAGASAIASALSPFSKPLQNLAGLPAWLASGAPAAAMTAAAGIPALAGGPTAINLGIANVGGGNVGNANNGLANIGNANLGNYNFGSGNFGNSNIGSASLGNNNIGFGNLGSNNVGVGNLGNLNTGFANTGLGNFGFGNTGNNNIGIGLTGNNQIGIGGLNSGTGNFGLFNSGSGNVGFFNSGNGNFGIGNSGNFNTGGWNSGHGNTGFFNAGSFNTGMLDVGNANTGSLNTGSYNMGDFNPGSSNTGTFNTGNANTGFLNAGNINTGVFNIGHMNNGLFNTGDMNNGVFYRGVGQGSLQFSITTPDLTLPPLQIPGISVPAFSLPAITLPSLTIPAATTPANITVGAFSLPGLTLPSLTIPAATTPANITVGAFSLPGLTLPSLNIPAATTPANITVGAFSLPGLTLPSLNIPAATTPANITVGAFSLPGLTLPSLNIPAATTPANITVGAFSLPGLTLPSLNIPAATTPANITVGAFSLPGLTLPSLNIPAATTPANITVGAFSLPGLTLPSLNIPAATTPANITVGAFSLPGLTLPSLNIPAATTPANITVGAFSLPGLTLPSLNIPAATTPANITVGAFSLPGLTLPSLNIPAATTPANITVSGFQLPPLSIPSVAIPPVTVPPITVGAFNLPPLQIPEVTIPQLTIPAGITIGGFSLPAIHTQPITVGQIGVGQFGLPSIGWDVFLSTPRITVPAFGIPFTLQFQTNVPALQPPGGGLSTFTNGALIFGEFDLPQLVVHPYTLTGPIVIGSFFLPAFNIPGIDVPAINVDGFTLPQITTPAITTPEFAIPPIGVGGFTLPQITTQEIITPELTINSIGVGGFTLPQITTPPITTPPLTIDPINLTGFTLPQITTPPITTPPLTIDPINLTGFTLPQITTPPITTPPLTIDPINLTGFTLPQITTPPITTPPLTIDPINLTGFTLPQITTPPITTPPLTIDPINLTGFTLPQITTPPITTPPLTIEPIGVGGFTTPPLTVPGIHLPSTTIGAFAIPGGPGYFNSSTAPSSGFFNSGAGGNSGFGNNGSGLSGWFNTNPAGLLGGSGYQNFGGLSSGFSNLGSGVSGFANRGILPFSVASVVSGFANIGTNLAGFFQGTTS

>4|CORE_REP|Org117_Gene2968#

MCDVLMQPVRTPRPSTNLRSKPLRPTGDGGVFPRLGRLIVRRPWVVIAFWVALAGLLAPTVPSLDAISQRHPVAILPSDAPVLVSTRQMTAAFREAGLQSVAVVVLSDAKGLGAADERSYKELVDALRRDTRDVVMLQDFVTTPPLRELMTSKDNQAWILPVGLPGDLGSTQSKQAYARVADIVEHQVAGSTLTANLTGPAATVADLNLTGQRDRSRIEFAITILLLVILLIIYGNPITMVLPLITIGMSVVVAQRLVAIAGLAGLGIANQSIIFMSGMMVGAGTDYAVFLISRYHDYLRQGADSDQAVKKALTSIGKVIAASAATVAITFLGMVFTQLGILKTVGPMLGISVAVVFFAAVTLLPALMVLTGRRGWIAPRRDLTRRFWRSSGVHIVRRPKTHLLASALVLVILAGCAGLARYNYDDRKTLPASVESSIGYAALDKHFPSNLIIPEYLFIQSSTDLRTPKALADLEQMVQRVSQVPGVAMVRGITRPAGRSLEQARTSWQAGEVGSKLDEGSKQIAVHTGDIDKLAGGANLMASKLGDVRAQVNRAISTVGGLIDALAYLQDLLGGNRVLGELEGAEKLIGSMRALGDTIDADASFVANNTEWASPVLGALDSSPMCTADPACASARTELQRLVTARDDGTLAKISELARQLQATRAVQTLAATVSGLRGALATVIRAMGSLGMSSPGGVRSKINLVNKGVNDLADGSRQLAEGVQLLVDQVKKMGFGLGEASAFLLAMKDTATTPAMAGFYIPPELLSYATGESVKAETMPSEYRDLLGGLNVDQLKKVAAAFISPDGHSIRYLIQTDLNPFSTAAMDQIDAITAAARGAQPNTALADAKVSVVGLPVVLKDTRDYSDHDLRLIIAMTVCIVLLILIVLLRAIVAPLYLIGSVIVSYLAALGIGVIVFQFLLGQEMHWSIPGLTFVILVAVGADYNMLLISRLREEAVLGVRSGVIRTVASTGGVITAAGLIMAASMYGLVFASLGSVVQGAFVLGTGLLLDTFLVRTVTVPAIAVLVGQANWWLPSSWRPATWWPLGRRRGRAQRTKRKPLLPKEEEEQSPPDDDDLIGLWLHDGLRL

>8|CORE_REP|Org1_Gene2938#

MQRFGTGSSRSWCGRAGTATIAAVLLASGALTGLPPAYAISPPTIDPGALPPDGPPGPLAPMKQNAYCTEVGVLPGTDFQLQPKYMEMLNLNEAWQFGRGDGVKVAVIDTGVTPHPRLPRLIPGGDYVMAGGDGLSDCDAHGTLVASMIAAVPANGAVPLPSVPRRPVTIPTTETPPPPQTVTLSPVPPQTVTVIPAPPPEEGVPPGAPVPGPEPPPAPGPQPPAVDRGGGTVTVPSYSGGRKIAPIDNPRNPHPSAPSPALGPPPDAFSGIAPGVEIISIRQSSQAFGLKDPYTGDEDPQTAQKIDNVETMARAIVHAANMGASVINISDVMCMSARNVIDQRALGAAVHYAAVDKDAVIVAAAGDGSKKDCKQNPIFDPLQPDDPRAWNAVTTVVTPSWFHDYVLTVGAVDANGQPLSKMSIAGPWVSISAPGTDVVGLSPRDDGLINAIDGPDNSLLVPAGTSFSAAIVSGVAALVRAKFPELSAYQIINRLIHTARPPARGVDNQVGYGVVDPVAALTWDVPKGPAEPPKQLSAPLVVPQPPAPAIWCRYGWPPGDWPGTIDRRCGVRYRDLDAAITEAAMKAQRSFGLALSWPRVTAVFLVDVLILAVASHCPDSWQADHHVAWWVGVGVAAVVTLLSVVSYHGITVISGLATWVRDWSADPGTTLGAGCTPAIDHQRRFGRDTVGVREYNGRLVSVIEVTCGESGPSGRHWHRKSPVPMLPVVAVADGLRQFDIHLDGIDIVSVLVRGGVDAAKASASLQEWEPQGWKSEERAGDRTVADRRRTWLVLRMNPQRNVAAVACRDSLASTLVAATERLVQDLDGQSCAARPVTADELTEVDSAVLADLEPTWSRPGWRHLKHFNGYATSFWVTPSDITSETLDELCLPDSPEVGTTVVTVRLTTRVGSPALSAWVRYHSDTRLPKEVAAGLNRLTGRQLAAVRASLPAPTHRPLLVIPSRNLRDHDELVLPVGQELEHATSSFVGQ

>9|CORE_REP|Org10_Gene4001#

MSGTPDDGDIGLIIAVKRLAAAKTRLAPVFSAQTRENVVLAMLVDTLTAAAGVGSLRSITVITPDEAAAAAAAGLGADVLADPTPEDDPDPLNTAITAAERVVAEGASNIVVLQGDLPALQTQELAEAISAARHHRRSFVADRLGTGTAVLCAFGTALHPRFGPDSSARHRRSGAVELTGAWPGLRCDVDTPADLTAARQLGGRARDRASGRTSLTGTGQRRRGIQGGERQTNGERMPAECWQPHPMMSNDRKVTEIENSPVTEVRPEEHAWYPDDSALAAPPAATPAAISDQLPSDRYLNRELSWLDFNARVLALAADKSMPLLERAKFLAIFASNLDEFYMVRVAGLKRRDEMGLSVRSADGLTPREQLGRIGEQTQQLASRHARVFLDSVLPALGEEGIYIVTWADLDQAERDRLSTYFNEQVFPVLTPLAVDPAHPFPFVSGLSLNLAVTVRQPEDGTQHFARVKVPDNVDRFVELAAREASEEAAGTEGRTALRFLPMEELIAAFLPVLFPGMEIVEHHAFRITRNADFEVEEDRDEDLLQALERELARRRFGSPVRLEIADDMTESMLELLLRELDVHPGDVIEVPGLLDLSSLWQIYAVDRPTLKDRTFVPATHPAFAERETPKSIFATLREGDVLVHHPYDSFSTSVQRFIEQAAADPNVLAIKQTLYRTSGDSPIVRALIDAAEAGKQVVALVEIKARFDEQANIAWARALEQAGVHVAYGLVGLKTHCKTALVVRREGPTIRRYCHVGTGNYNSKTARLYEDVGLLTAAPDIGADLTDLFNSLTGYSRKLSYRNLLVAPHGIRAGIIDRVEREVAAHRAEGAHNGKGRIRLKMNALVDEQVIDALYRASRAGVRIEVVVRGICALRPGAQGISENIIVRSILGRFLEHSRILHFRAIDEFWIGSADMMHRNLDRRVEVMAQVKNPRLTAQLDELFESALDPCTRCWELGPDGQWTASPQEGHSVRDHQESLMERHRSP

>10|CORE_REP|Org118_Gene2197#

MVPGEVHMSDTPSGPHPIIPRTIRLAAIPILLCWLGFTVFVSVAVPPLEAIGETRAVAVAPDDAQSMRAMRRAGKVFNEFDSNSIAMVVLESDQPLGEKAHRYYDHLVDTLVLDQSHIQHIQDFWRDPLTAAGAVSADGKAAYVQLYLAGNMGEALANESVEAVRKIVANSTPPEGIRTYVTGPAALFADQIAAGDRSMKLITGLTFAVITVLLLLVYRSIATTLLILPMVFIGLGATRGTIAFLGYHGMVGLSTFVVNILTALAIAAGTDYAIFLVGRYQEARHIGQNREASFYTMYRGTANVILGSGLTSIAGATYCLSFARLTLFHTMGPPLAIGMLVSVAAALTLAPAIIAIAGRFGLLDPKRRLKTRGWRRVGTAVVRWPGPILATSVALALVGLLALPGYRPGYNDRYYLRAGTPVNRGYAAADRHFGPARMNPEMLLVESDQDMRNPAGMLVIDKIAKEVLHVSGVERVQAITRPQGVPLEHASIPFQISMMGATQTMSLPYMRERMADMLTMSDEMLVAINSMEQMLDLVQQLNDVTHEMAATTREIKATTSELRDHLADIDDFVRPLRSYFYWEHHCFDIPLCSATRSLFDTLDGVDTLTDQLRALTDDMNKMEALTPQFLALLPPMITTMKTMRTMMLTMRSTISGVQDQMADMQDHATAMGQAFDTAKSGDSFYLPPEAFDNAEFQQGMKLFLSPNGKAVRFVISHESDPASTEGIDRIEAIRAATKDAIKATPLQGAKIYIGGTAATYQDIRDGTKYDILIVGIAAVCLVFIVMLMITQSLIASLVIVGTVLLSLGTAFGLSVLIWQHFVGLQVHWTIVAMSVIVLLAVGSDYNLLLVSRFKEEGAGAGLKTGIIRAMAGTGASCHVGRSGIRVHHGVHGRQRTPRYRTGRHHHRARSTFRYPGGPIVHDAIHRSAARSLVLVAEHDPLETHRPGGAHTPGRSPNSAASAPGLICTSVP

>12|CORE_REP|Org122_Gene4011#

MRSQRLAGHLSAAARTIHALSLPIILFWVALTIVVNVVAPQLQSVARTHSVALGPHDAPSLIAMKRIGKDFQQFDSDTTAMVLLEGQEKLGDEAHRFYDVLVTKLSQDTTHVQHIENFWGDPLTAAGSQSADGKAAYVQLNLTGDQGGSQANESVAAVQRIVDSVPPPPGIKAYVTGPGPLGADRVVYGDRSLHTITGISIAVIAIMLFIAYRSLSAALIMLLTVGLELLAVRGIISTFAVNDLMGLSTFTVNVLVALTIAASTDYIIFLVGRYQEARATGQNREAAYYTMFGGTAHVVLASGLTVAGAMYCLGFTRLPYFNTLASPYFNTLASPCAIGLVTVMLASLTLAPAIIAVASRFGLFDPKRATTKRRWRRIGTVVVRWPGPVLAATLLIALIGLLALPKYQTNYNERYYIPSAAPSNIGYLASDRHFPQARMEPEVLMVEADHDLRNPTDMLILDRIAKTVFHTPGIARVQSITRPLGAPIDHSSIPFQLGMQSTMTIENLQNLKDRVADLSTLTDQLQRMIDITQRTQELTRQLTDATHDMNAHTRQMRDNANELRDRIADFDDFWRPLRSFTYWERHCFDIPICWSMRSLLNSMDNVDKLTEDLANLTDDTERMDTTQRQLLAQLDPTIATMQTVKDLAQTLTSAFSGLVTQMEDMTRNATVMGRTFDAANNDDSFYLPPEAFQNPDFQRGLKLFLSPDGTCARFVITHRGDPASAEGISHIDPIMQAADEAVKGTPLQAASIYLAGTSSTYKDIHEGTLYDVMIAVVASLCLIFIIMLGITRSVVASAVIVGTVALSLGSAFGLSVLIWQHILHMPLHWLVLPMAIIVMLAVGSDYNLLLIARFQEEIGAGLKTGMIRAMAGTGRVVTIAGLVFAFTMGSMVASDLRVVGQIGTTIMIGLLFDTLVVRSYMTPALATLLGRWFWWPRRVDRLARQPQVLGPRRTTALSAERAALLQ

>19|CORE_REP|Org118_Gene1392#

MTLTPEASKSVAQPPTQAPLTQEEAIASLGRYGYGWADSDVAGANAQRGLSEAVVRDISAKKNEPDWMLQSRLKALRIFDRKPIPKWGSNLDGIDFDNIKYFVRSTEKQAASWDDLPEDIRNTYDRLGIPEAEKQRLVAGVAAQYESEVVYHQIREDLEAQGVIFLDTDTGLREHPDIFKEYFGTVIPAGDNKFSALNTAVWSGGSFIYVPPGVHVDIPLQAYFRINTENMGQFERTLIIADEGSYVHYVEGCLPPGGELITTADGDLRPIESIRVGDFVTGHDGRPHRVTAVQVRDLDGELFTFTPMSPANAFSVTAEHPLLAIPRDEVRVMRKERNGWKAEVNSTKLRSAEPRWIAAKDVAEGDFLIYPKPKPIPHRTVLPLEFARLAGYYLAEGHACLTNGCESLIFSFHSDEFEYVEDVRQACKSLYEKSGSVLIEEHKHSARVTVYTKAGYAAMRDNVGIGSSNKKLSDLLMRQDETFLRELVDAYVNGDGNVTRRNGAVWKRVHTTSRLWAFQLQSILARLGHYATVELRRPGGPGVIMGRNVVRKDIYQVQWTEGGRGPKQARDCGDYFAVPIKKRAVREAHEPVYNLDVENPDSYLAYGFAVHNCTAPIYKSDSLHSAVVEIIVKPHARVRYTTIQNWSNNVYNLVTKRARAEAGATMEWIDGNIGSKVTMKYPAVWMTGEHAKGEVLSVAFAGEDQHQDTGAKMLHLAPNTSSNIVSKSVARGGGRTSYRGLVQVNKGAHGSRSSVKCDALLVDTVSRSDTYPYVDIREDDVTMGHEATVSKVSENQLFYLMSRGLTEDEAMAMVVRGFVEPIAKELPMEYALELNRLIELQMEGAVG

>21|CORE_REP|Org20_Gene1819#

MTDRVSVGNLRIARVLYDFVNNEALPGTDIDPDSFWAGVDKVVADLTPQNQALLNARDELQAQIDKWHRRRVIEPIDMDAYRQFLTEIGYLLPEPDDFTITTSGVDAEITTTAGPQLVVPVLNARFALNAANARWGSLYDALYGTDVIPETDGAEKGPTYNKVRGDKVIAYARKFLDDSVPLSSGSFGDATGFTVQDGQLVVALPDKSTGLANPGQFAGYTGAAESPTSVLLINHGLHIEILIDPESQVGTTDRAGVKDVILESAITTIMDFEDSVAAVDAADKVLGYRNWLGLNKGDLAAAVDKDGTAFLRVLNRDRNYTAPGGGQFTLPGRSLMFVRNVGHLMTNDAIVDTDGSEVFEGIMDALFTGLIAIHGLKASDVNGPLINSRTGSIYIVKPKMHGPAEVAFTCELFSRVEDVLGLPQNTMKIGIMDEERRTTVNLKACIKAAADRVVFINTGFLDRTGDEIHTSMEAGPMVRKGTMKSQPWILAYEDHNVDAGLAAGFSGRAQVGGHVDNAELMADMVETKIAQPRAGASTAWVPSPTAATLHALHYHQVDVAAVQQGLAGKRRATIEQLLTIPLAKELAWAPDEIREEVDNNCQSILGYVVRWVDQGVGCSKVPDIHDVALMEDRATLRISSQLLANWLRHGVITSADVRASLERMAPLVDRQNAGDVAYRPMAPNFDDSIAFLAAQELILSGAQQPNGYTEPSMPDVVGSLRPGPLRSRPHRTGPVTMRPASGPLWGHRPLAGESRPHRAEPVRSSCLTGAGLRRRGRRRRYG

>22|CORE_REP|Org51_Gene3937#

MAIAETDTEVHTPFEQDFEKDVAATQRYFDSSRFAGIIRLYTARQVVEQRGTIPVDHIVAREAAGAFYERLRELFAARKSITTFGPYSPGQAVSMKRMGIEAIYLGGWATSAKGSSTEDPGPDLASYPLSQVPDDAAVLVRALLTADRNQHYLRLQMSERQRAATPAYDFRPFIIADADTGHGGDPHVRNLIRRFVEVGVPGYHIEDQRPGTKKCGHQGGKVLVPSDEQIKRLNAARFQLDIMRVPGIIVARTDAEAANLIDSRADERDQPFLLGATKLDVPSYKSCFLAMVRRFYELGVKELNGHLLYALGDSEYAAAGGWLERQGIFGLVSDAVNAWREDGQQSIDGIFDQVESRFVAAWEDDAGLMTYGEAVADVLEFGQSEGEPIGMAPEEWRAFAARASLHAARAKAKELGADPPWDCELAKTPEGYYQIRGGIPYAIAKSLAAAPFADILWMETKTADLADARQFAEAIHAEFPEQMLAYNLSPSFNWDTTGMTDEEMRRFPEELGKMGFVFNFITYGGHQIDGVAAEEFATALRQDGMLALARLQRKMRLVESPYRTPQTLVGGPRSDAALAASSGRTATTKAMGKGSTQHQHLVQTEVPRKLLEEWLAMWSGHYQLKDKLRVQLRPQRAGSEVLELGIHGESDDKLANVIFQPIQDRRGRTILLVRDQNTFGAELRQKRLMTLIHLWLVHRFKAQAVHYVTPTDDNLYQTSKMKSHGIFTEVNQEVGEIIVAEVNHPRIAELLTPDRVALRKLITKEA

>23|CORE_REP|Org59_Gene1377#

MRWATVALLLFLAGLVAQLNGAPEAMWWTLYLACYLAGGWGSAWAGAQALRBKALDVDLLMIAAAVGAVAIGQIFDGALLIVIFATSGALDDIATRHTAESVKGLLDLAPDQAVVVQGDGSERVVAASELVVGDRVVVRPGDRIXADGAVLSGXXDVDQRSITGESMPVAKARGDEVFAGTVNGSGVLHLVVTRDPSQTVVARIVELVADASATKAKTQLFIEKIEQRYSLGMVAATLALIVIPLMFGADLRPVLLRAMTFMIVASPCAVVLATMPPLLSAIANAGRHGVLVKSAVVVXRLADTSIVALDKTGTLTRGIPRLASVAPLDPNVVDARRLLXXAXAAEQSSEHPLGRAIVAEARRRGIAIPPAKDFRAVPGCGVHALVGNDFVEIASPQSYRGAPLAXLAPLLSAGATAAIVLLDGVAIGVLGLTDQLRPDAVESVAAMAALTAAPPVLLTGDNGRAAWRVARNAGITDVRAALLPEQKVEVVRNLQAGGHQVLLVGDGVNDAPAMAAARAAVAMGAGADLTLQTADGVTIRDELHXIPTIIGLARQARRVVTVNLAIAATFIAVLVLWDLFGQLPLPLGVVGHEGSTVLVALNGMRLLTNRSWRXAXXXXXXGSXVAELTRAALGVPVTTRDLTAAYFQQTISANSNVLVYFWAPLCAPCDLFTPTYEASSRKHFDVVHGKVNIETEKDLASIAGVKLLPTLMAFKKGKLXLXXSRHRQSRDHGQSGATTPGIHLQVPGRRRYRPWNKDFILRR

>38|CORE_REP|Org2_Gene1795#

MSPQLCPKVSIVSTTHNQAGYARQAFDSFLDQQTDFPVEIIVADDASTDATPAIIREYAERYPHVFRPIFRTENLGLNGNLTGALSAARGEYVALCEADDYWIDPLKLSKQVAFLDRHPKTTVCFHPVRVIWEDGHAKDSKFPPVRVRGNLSLDALILMNFIQTNSAVYRRLERYDDIPADVMPLDWYLHVRHAVHGDIAMLPDTMAVYRRHAQGMWHNQVVDPPKFWLTQGPGHAATFDAMLDLFPGDPAREELIAVMADWILRQIANVPGPEGAPRCRKPSRAIPGSPCWRCSTAGDTRAAAQDPVAQARRRDAEPQGARGCVALPAPTRLSSLTMSTNPGPAEGANQVMAQEHSAGAVQFTAHNVRLDDGTLTIPESSRTLDESSWFISARGILETVFPGDKSHLRLADVGCLEGGYAVGFARMGFQVLGIEVRELNMAACNYIKSKTNLPNLRFVHDNALNIANHGLFDTVFCCGLFYHLENPKQYLETLSSVTNKLLILQTHFSIINRSDKWLRLPTTARQLTDRLLRRPAPVKFMLSAPTEHEGLPGRWFTEFSDDRSFGQRDTAKWASWDNRRSFWIQREHLLQAIKDVGVDLVMEEYDNLEPSIAESLLGGSYAANLRGTFIGIKTR

>43|CORE_REP|Org1_Gene1647#

MASRQTPAELARCDLAKTAEREHTPTATATTPSVAGNVMPMSVRSLPAALRACARLQPHDPAFTFMDYEQDWDGVAITLTWSQLYRRTLNVAQELSRCGSTGDRVVISAPQGLEYVVAFLGALQAGRIAVPLSVPQGGVTDERSDSVLSDSSPVAILTTSSAVDDVVQHVARRPGESPPSIIEVDLLDLDAPNGYTFKEDEYPSTAYLQYTSGSTRTPAGVVMSHQNVRVNFEQLMSGYFADTDGIPPPNSALVSWLPFYHDMGLVIGICAPILGGYPAVLTSPVSFLQRPARWMHLMASDFHAFSAAPNFAFELAARRTTDDDMAGRDLGNILTILSGSERVQAATIKRFADRFARFNLQERVIRPSYGLAEATVYVATSKPGQPPETVDFDTESLSAGHAKPCAGGGATSLISYMLPRSPIVRIVDSDTCIECPDGTVGEIWVHGDNVANGYWQKPDESERTFGGKIVTPSPGAPEGPWLRTGDSGFVTDGKMFIIGRIKDLLIVYGRNHSPDDIEATIQEITRGRCAAISVPGDRSTEKLVAIIELKKRGDSDQDAMARLGAIKREVTSALSSSHGLSVADLVLVAPGSIPITTSGKVRRGACVEQYRQDQFARLDA

>56|CORE_REP|Org88_Gene3088#

MTTGGLVDENDGAAMRPLRHTLSQLRLHELLVEVQDRVEQIVEGRDRLDGLVEAMLVVTAGLDLEATLRAIVHSATSLVDARYGAMEVHDRQHRVLHFVYEGIDEETVRRIGHLPKGLGVIGLLIEDPKPLRLDDVSAHPASIGFPPYHPPMRTFLGVPVRVRDESFGTLYLTDKTNGQPFSDDDEVLVQALAAAAGIAVANARLYQQAKARQSWIEATRDIATELLSGTEPATVFRLVAAEALKLTAADAALVAVPVDEDMPAADVGELLVIETVGSAVASIVGRTIPVAGAVLREVFVNGIPRRVDRVDLEGLDELADAGPALLLPLRARGTVAGVVVVLSQGGPGAFTDEQLEMMAAFADQAALAWQLATSQRRMRELDVLTDRDRIARDLHDHVIQRLFAIGLALQGAVPHERNPEVQQRLSDVVDDLQDVIQEIRTTIYDLHGASQGITRLRQRIDAAVAQFADSGLRTSVQFVGPLSVVDSALADQAEAVVREAVSNAVRHAKASTLTVRVKVDDDLCIEVTDNGRGLPDEFTGSGLTNLRQRAEQAGGEFTLASVPGASGTVLRWSAPLSQ

>70|CORE_REP|Org20_Gene3484#

MDFGALPPEINSARMYAGPGSASLVAAAKMWDSVASDLFSAASAFQSVVWGLTVGSWIGSSAGLMAAAASPYVAWMSVTAGQAQLTAAQVRVAAAAYETAYRLTVPPPVIAENRTELMTLTATNLLGQNTPAIEANQAAYSQMWGQDAEAMYGYAATAATATEALLPFEDAPLITNPGGLLEQAVAVEEAIDTAAANQLMNNVPQALQQLAQPAQGVVPSSKLGGLWTAVSPHLSPLSNVSSIANNHMSMMGTGVSMTNTLHSMLKGLAPAAAQAVETAAENGVWAMSSLGSQLGSSLGSSGLGAGVAANLGRAASVGSLSVPPAWAAANQAVTPAARALPLTSLTSAAQTAPGHMLGGLPLGHPRRQRYPAHWRRHGPTRYPAHRPPDSTTGLRGCVGVVPRRGWRALAIWSKGPDPTGRTPRHRGAVDGIRKAVTGNGIDAGTTIRDDHDGRRQPRDLDGRCYAGDQPARAGLTMAQAGGNLPDQQPTHQRVSRFPAALRSTNRPPRHGRRRRHLVGNKCGRHQRGFACVIPSHRV

>73|CORE_REP|Org87_Gene1778#

MAATKASTATDEPVKRTATKSPAASASGAKTGAKRTAAKSASGSPPAKRATKPAARSVKPASAPQDTTTSTIPKRKTRAAAKSAAAKAPSARGHATKPRAPKDAQHEAATDPEDALDSVEELDAEPDLDVEPGEDLDLDAADLNLDDLEDDVAPDADDDLDSGDDEDHEDLEAEAAVAPGQTADDDEEIAEPTEKDKASGDFVWDEDESEALRQARKDAELTASADSVRAYLKQIGKVALLNAEEEVELAKRIEAGLYATQLMTELSERGEKLPAAQRRDMMWICRDGDRAKNHLLEANLRLVVSLAKRYTGRGMAFLDLIQEGNLGLIRAVEKFDYTKGYKFSTYATWWIRQAITRAMADQARTIRIPVHMVEVINKLGRIQRELLQDLGREPTPEELAKEMDITPEKVLEIQQYAREPISLDQTIGDEGDSQLGDFIEDSEAVVAVDAVSFTLLQDQLQSVLDTLSEREAGVVRLRFGLTDGQPRTLDEIGQVYGVTRERIRQIESKTMSKLRHPSRSQVLRDYLD

>75|CORE_REP|Org9_Gene1083#

MHADLAATTSREDFRLLAAEHRVVPVTRKVLADSETPLSAYRKLAANRPGTFLLESAENGRSWSRWSFIGAGAPTALTVREGQAVWLGAVPKDAPTGGDPLRALQVTLELLATADRQSEPGLPPLSGGMVGFFAYDMVRRLERLPERAVDDLCLPDMLLLLATDVAAVDHHEGTITLIANAVNWNGTDERVDWAYDDAVARLDVMTAALGQPLPSTVATFSRPEPRHRAQRTVEEYGAIVEYLVDQIAAGEAFQVVPSQRFEMDTDVDPIDVYRILRVTNPSPYMYLLQVPNSDGAVDFSIVGSSPEALVTVHEGWATTHPIAGTRWRGRTDDEDVLLEKELLADDKERAEHLMLVDLGRNDLGRVCTPGTVRVEDYSHIERYSHVMHLVSTVTGKLGEGRTALDAVTACFPAGTLSGAPKVRAMELIEEVEKTRRGLYGGVVGYLDFAGNADFAIAIRTALMRNGTAYVQAGGGVVADSNGSYEYNEARNKARAVLNAIAAAETLAAPGANRSGC

>82|CORE_REP|Org67_Gene4027#

MRIGPVELSAVKDWDPAPGVLVSWHPTPASCAKAFAAPVSAVPPSYVQARQIRSFSEQAARGLDHSRLLIASVEVFGHCDLRAMTYVINAHVRRHDTYRSWFELRDTDHIVRHSIADPADIEFVPTTHGEMTSADLRQHIVATPDSLHWDCFSFGVIQRADSFTFYASIDHLHADGQFVGVGLMEFQSMYTALIMGEPPIGLSEAGSYVDFCVRQHEYTSALTVDSPEVRAWIDFAEINNGTFPEFPLPLGDPSVRCGGDLLSMMLMDEQQTQRFESACMAANARFIGGMLACIAIAIHELTGADTYFGITPKDIRTPADLMTQGWFTGQIPVTVPVAGLSFNEIARIAQTSFDTGADLAKVPFERVVELSPSLRRPQPLFSLVNFFDAQVGPLSAVTKLFEGLNVGTYSDGRVTYPLSTMVGRFDETAASVLFPDNPVARESVTAYLRAIRSVCMRIANGGTAERVGNVVALSPGRRNNIERMTWRSCRAGDFIDICNLKVANVTVDREA

>86|CORE_REP|Org118_Gene1678#

MAEESRGQRGSGYGLGLSTRTQVTGYQFLARRTAMALTRWRVRMEIEPGRRQTLAVVASVSAALVICLGALLWSFISPSGQLNESPIIADRDSGALYVRVGDRLYPALNLASARLITGRPDNPHLVRSSQIATMPRGPLVGIPGAPSSFSPKSPPASSWLVLGDTVATSSSIGSLQGVTVTVIDGTPDLTGHRQILSGSDAVVLRYGGDAWVIREGRRSRIEPTNRAVLLPLGLTPEQVSQARPMSRALFDALPVGPELLVPEVPNAGGPATFPGAPGPIGTVIVTPQISGPQQYSLVLGDGVQTLPPLVAQILQNAGSAGNTKPLTVEPSTLAKMPVVNRLDLSAYPDNPLEVVDIREHPSTCWWWERTAGENRARVRVVSGPTIPVAATEMNKVVSLVKADTSGRQADQVYFGPDHANFVAVTGNNPGAQTSESLWWVTDAGARFGVEDSKEARDALGLTLTPSLAPWVALRLLPQGPTLSRADALVEHDTLPMDMTPAELVVPK

>92|CORE_REP|Org1_Gene2915#

MSFVVTIPEALAAVATDLAGIGSTIGTANAAAAVPTTTVLAAAADEVSAAMAALFSGHAQAYQALSAQAALFHEQFVRALTAGAGSYAAAEAASAAPLEGVLDVINAPALALLGRPLIGNGANGAPGTGANGGDGGILIGNGGAGGSGAAGMPGGNGGAAGLFGNGGPAAPGERSVRHRRVRRGRRAGGLLYGAGGAGGAGGRAGGGVGGIGGAGGAGGNGGLLFGAGGPAASADSRPTPVTAGRRRRRVVLRRGRCRRAGGTGTNVTGGAGGAGGNGGLLFGAGGVGGVGGDGVAFLGTAPAGPVVPAGPVGCSASVGPAAPAESDWSGTAVPGVRRVRPALGRRRCRRRGWVGSTTGGAGGAGGNAGLLVGAGGAGGAGALGGGATGVGGAGGNGGTAGLLFGAGGAGGAGGFGFGGAGGAGGLGGKAGLIGDGGDGGAGGNGTGAKGGDGGAGGGAILVGNGGNGGNAGSGTPNGSAGTGGAGGLLGKNGMNGLP

>94|CORE_REP|Org59_Gene728#

MARHLRGRLPLRVRLVAATLILVATGLVASGIAVTSMLQHRLTSRIDRVLLEEAQIWAQITLPLAPDPYPGHNPDRPPSRFYVRVISPDGQSYTALNDNTAIPAVPANNDVGRHPTTLPSIGGSKTLWRAVSVRASDGYLTTVAIDLADVRSTVRSLVLLQVGIGSAVLVVLGVAGYAVVRRSLRPLAEFEQTAAAIGAGQLDRRVPQWHPRTEVGRLSLALNGMLAQIQRAVASAESSAEKARDSEDRMRQFITDASHELRTPLTTIRGFAELYRQGAARDVGMLLSRIESEASRMGLLVDDLLLLARLDAXRPLELCRVDLLALASDAAHDARAMDPKRRITLEVLDGPGTPEVLGDESRLRQVLRNLVANAIQHTPESADVTVRVGTEGDDAILEVADDGPGMSQEDALRVFERFYRADSSRARASGGTGLGLSIVDSLVAAHGGAVTVTTALGEGCCFRVSLPRVSDRGPAEPHASCARAALILACAIVQRSR

>123|CORE_REP|Org94_Gene1437#

MNWTVDIPIDQLPSLPPLPTDLRTRLDAALAKPAAQQPTWPADQALAMRTVLESVPPVTVPSEIVRLQEQLAQVAKGEAFLLQGGDCAETFMDNTEPHIRGNVRALLQMAVVLTYGASMPVVKVARIAGQYAKPRSADIDALGLRSYRGDMINGFAPDAAAREHDPSRLVRAYANASAAMNLVRALTSSGLASLHLVHDWNREFVRTSPAGARYEALATEIDRGLRFMSACGVADRNLQTAEIYASHEALVLDYERAMLRLSDGEDGEPQLFDLSAHTVWIGERTRQIDGAHIAFAQVIANPVGVKLGPNMTPELAVEYVERLDPHNKPGRLTLVSRMGNHKVRDLLPPIVEKVQATGHQVIWQCDPMHGNTHESSTGFKTRHFDRIVDEVQGFFEVHRALGTHPGGIHVEITGENVTECLGGAQDISETDLAGRYETACDPRLNTQQSLELAFLVAEMLRD

>125|CORE_REP|Org87_Gene220#

MIRAAFACLAATVVVAGWWTPPAWAIGPPVVDAAAQPPSGDPGPVAPMEQRGACSVSGVIPGTDPGVPTPSQTMLNLPAAWQFSRGEGQLVAIIDTGVQPGPRLPNVDAGGDFVESTDGLTDCDGHGTLVAGIVAGQPGNDGFSGVAPAARLLSIRAMSTKFSPRTSGGDPQLAQATLDVAVLAGAIVHAADLGAKVINVSTITCLPADRMVDQAALGAAIRYAAVDKDAVIVAAAGNTGASGSVSASCDSNPLTDLSRPDDPRNWAGVTSVSIPSWWQPYVLSVASLTSAGQPSKFSMPGPWVGIAAPGENIASVSNSGDGALANGLPDAHQKLVALSGTSYAAGYVSGVAALVRSRYPGLNATEVVRRLTATAHRGARESSNIVGAGNLDAVAALTWQLPAEPGGGAAPAKPVADPPVPAPKDTTPRNVAFAGAAALSVLVGLTAATVAIARRRREPTE

>145|CORE_REP|Org118_Gene2593#

MYAGAGAAPLMAAGATWNGLAVELSTTASSVESVIMQLTTEQWLGPASMSMVVAAQPYLAWLTYTAESAAHAAAQAMASAAAFEAAFAMTVPPAEVAANRALLAALVATNVLGQNTPAIMATEAHYGEMWAQDALAMYGYAASSAAAGRLNPLITPSQTANMAGLAGQAAAVSHAAAASTVQQVGLGSLISNLPNAVMGFASPLTSAADAAGLGGIIQDIEELLGITFVQNAINGAVNTTAWFVMATIPNAVFLGHAFAALNPATVTAAADAVPAAAAAAGLAHTVTPVGVGGASLTASLGEASSVGGLSVPAGWSTAAPAMTFWYHGTGGLGLGGPRGSRASRRNCRVWRGFLGRPKEPVPMPGLGTGSSPSSCPNRSSSDWPVAHRPGRPAMSRTATRQAWPVRQPTVGPQPTPPGPSIKPPEPWRRSRPRRAR

>146|CORE_REP|Org118_Gene2595#

MDFGALPPEVNSARMYGGAGAADLLAAAAAWNGIAVEVSTAASSVGSVITRLSTEHWMGPASLSMAAAVQPYLVWLTCTAESSALAAAQAMASAAAFETAFALTVPPAEVVANRALLAELTATNILGQNVSAIAATEARYGEMWAQDASAMYGYAAASAVAARLNPLTRPSHITNPAGLAHQAAAVGQAGASAFARQVGLSHLISDVADAVLSFASPVMSAADTGLEAVRQFLNLDVPAVRRIRVSRPGWRGRLCHGRHWQYDASCRCYGNRWRSRSRWRRGSRGGTRGCPSGRRRNSADRRFGQCVRGWSPVGAGKLVYCSAGDGSRRGLGWHRLGSSRGGRPDRSDAACPWNGRSRQQCWCRLRTTVRSQADCYAQARPLLIWRHRDKRTRPTSAPPRPHCANPPAARVSFRCCGERSGATLKRIVLRLPVPA

>151|CORE_REP|Org12_Gene3891#

MSSWPPRAGSTGSTIAASTSTAATSRRSNSRLPTTLNARDQPPAEVSDQRVSGLTGAVHYAGAGSGPLFMAAAAWEGLAADLRASASSFDAVIAGLAAGPWSGPASVAMAGAAAPYVGWLSAAAGQAELSAGQATAAATAFEAALAATVHPAAVTANRVLLGALVATNILGQNTPAIAATEFDYVEMWAQDVGAMVGYHAGAAAVAETLTPFSVPPLDLAGLASQAGAQLTGMATSVSAALSPIAEGAVEGVPAVVAAAQSVAAGLPVDAALQVGQAAAYPASMLIGPMMQLAQMGTTANTAGLAGAEAAGLAAADVPTFAGDIASGTGLGGAGGLGAGMSAELGKARLVGAMSVPPTWEGSVPARMASSAMAGLGAMPAEVPAAGGPMGMMPMPMGMGGAGAGMPAGMMGRGGANPHVVQARPSVVPRVGIG

>159|CORE_REP|Org59_Gene1576#

MDFGALPPEVNSGRMYCGPGSAPMVAAASAWNGLAAELSVAAVGYERVITTLQTEEWLGPASTLMVEAVAPYVAWMRATAIQAEQAASQARAAAAAYETAFAAIVPPPLIAANRARLTSLVTHNVFGQNTASIAATEAQYAEMWAQDAMAMYGYAGSSATATKVTPFAPPPNTTSPSAAATQLSAVAKAAGTSAGAAQSAIAELIAHLPNTLLGLTSPLSSALTAAATPGWLEWFINWYLPISQLFYNTVGLPYFAIGIGNSLITSWRALGXXGPXAAEAAAAAPAAVGAAVGGTGPVSAGLGNAATIGKLSLPPNWAGASPSLAPTVGSASAPLVSDIVEQPEAGAAGKPVGRHAASRFGHRYGGCGSPLRVPGYGDVPAAVCRITRGLPYPRTRGRHICRTIXRLPXRQTPSRTCVGTXPXAIFS

>161|CORE_REP|Org119_Gene1671#

MDFGLLPPEINSGRMYTGPGPGPMLAAATAWDGLAVELHATAAGYASELSALTGAWSGPSSTSMASAAAPYVAWMSATAVHAELAGAQARLAIAAYEAAFAATVPPPVIAANRAQLMVLIATNIFGQNTPAIMMTEAQYMEMWAQDAAAMYGYAGSSATASRMTAFTEPPQTTNHGQLGAQSSAVAQTAATAAGGNLQSAFPQLLSAVPRALQGLALPTASQSASATPQWVTDLGNLSTFLGGAVTGPYTFPGVLPPSGVPYLLGIRMARVLGTQNGQGVSALLGKIGGKPITGALAPLAEFALHTPILGSEGLGGGSVSAGIGRAGLVGKLSVPQGWTVAAPEIPSPAAALQATRLAAAPIAATDGAGALLGGMALSGLAGRAAAGSTGHPIGSAAAPAVGAAAAAVEDLATEANIFVIPAMDD

>164|CORE_REP|Org59_Gene1167#

MAEIVLDHVNKSYPDGHTAVRDLNLTIADGEFLILVGPSGCGKTTTLNMIAGLEDISSGELRIAGERVNEKAPKDRDIAMVFQSYALYPHMTVRQNIAFPLTLAKMRKADIAQKVSETAKILDLTNLLDRKPSQLSGGQRQRVAMGRAIVRHPKAFLXXEPLSNLDAKLRVQMRGEIAQLQRRLGTTTVYVTHDQTEAMTLGDRVVVMYGGIAQQIGTPEELYERPANLFVAGFIGSPAMNFFPARLTAIGLXLPFGEVTLAPEVQGVIAAHPKPENVIVGVRPEHIQDAALIDAYQRIRALTFQVKVNLVESLGADKYLYFTTESPAVHSVQLDELAEVEGESALHENQFVARVPRRVQGSHRAVGRVGFRYRQTCRLRRRLRCEPDHSAPRLMAASXHISPXHAXGFGAFCVCSPTRS

>165|CORE_REP|Org59_Gene1373#

MTASVNSLDLAAIRADFPILKRIMRGGNPLAYLDSGATSQRPLQVLDAEREFLTASNGAVHRGAHXLMEEATDAYEQGRADIVGGVIRRQXTRTSWCSPKMPPRRSTWCHMCWGTAVSSVTXGPGDVIVTTELEHHANLIPWQELARRTGATLRWXGVTDDGRIDLDSLYLDDRVKVVAFTHHSNVTGVLTPVSELVSRAHQSGALTVLXXCQSVPHQPVDLHELGVDFAAFSGHKMLGPNGIGVLYGRRELLAQMPPFLTGGSMIETVTXEGATYAPAPQRFEAGTPMTSQVVGLAAAARYLGAIGMAAVEAHERELVAAAIEGLSGIDGVRILGPTSMRDRGSPVAFVVEGVHAHDVGQVLDDGGVAVRVGHHCALPLHRRFGLAATARASFAVYNTADEVDRLVAGVRRSRHFFGRA

>167|CORE_REP|Org7_Gene3851#

MASGSGLCKTTSNFIWGQLLLLGEGIPDPGDIFNTGSSLFKQISDKMGLAIPGTNWIGQAAEAYLNQNIAQQLRAQVMGDLDKLTGNMISNQAKYVSDTRDVLRAMKKMIDGVYKVCKGLEKIPLLGHLWSWELAIPMSGIAMAVVGGALLYLTIMTLMNATNLRGILGRLIEMLTTLPKFPGLPGLPSLPDIIDGLWPPKLPDIPIPGLPDIPGLPDFKWPPTPGSPLFPDLPSFPGFPGFPEFPAIPGFPALPGLPSIPNLFPGLPGLGDLLPGVGDLGKLPTWTELAALPDFLGGFAGLPSLGFGNLLSFASLPTVGQVTATMGQLQQLVAAGGGPSQLASMGSQQAQLISSQAQQGGQQHATLVSDKKEDEEGVAEAERAPIDAGTAASQRGQEGTVLRSDTESPAGLCHSESKP

>186|CORE_REP|Org118_Gene1238#

MQYGLEVSSDVAGVAGGLLALSYRGAGVPLRELALVGLTAAIITYFATGPVRMLASRLGAVAYPRERDVHVTPTPRMGGLAMFLGIVGAVFLASQLPALTRGFVYSTGMPAVLVAGAVIMGIGLIDDRWGLDALTKFAGQITAASVLVTMGVAWSVLYIPVGGVGTIVLDQASSILLTLALTVSIVNAMNFVDGLDGLAAGLGLITALAICMFSVGLLRDHGGDVLYYPPAVISVVLAGACLGFLPHNFHRAKIFMGDSGSMLIGLMLAAASTTAAGPISQNAYGARDVFALLSPFLLVVAVMFVPMLDLLLAIVRRTRAGRSAFSPDKMHLHHRLLQIGHSHRRVVLIIYLWVGIVAFGAASSIFFNPRDTAAVMLGAIVVAGVATLIPLLRRGDDYYDPDLD

>190|CORE_REP|Org69_Gene2725#

MVEAGTRDPLESALLDSRYLVQAKIASGGTSTVYRGLDVRLDRPVALKVMDSRYAGDEQFLTRFRLEARAVARLNNRALVAVYDQGKDGRHPFLVMELIEGGTLRELLIERGPMPPHAVVAVLRPVLGGLAAAHRAGLVHRDVKPENILISDDGDVKLADFGLVRAVAAASITSTGVILGTAAYLSPEQVRDGNADPRSDVYSVGVLVYELLTGHTPFTGDSALSIAYQRLDADVPRASAVIDGVPPQFDELVACATARNPADRYADAIAMGADLEAIAEELALPEFRVPAPRNSAQHRSAALYRSRITQQGQLGAKPVHHPTRQLTRQPGDCSEPASGSEPEHEPITGQFAGIAIEEFIWARQHARRMVLVWVSVVLAITGLVASAAWTIGSNLSGLL

>193|CORE_REP|Org59_Gene1577#

MTLDVPVNQGHVPPGSVACCLVGVTAVADGIAGHSLSNFGALPPEINSGRMYSGPGSGPLMAAAAAWDGLAAELSSAATGYGAAISELTNMRWWSGPASDSMVAAVLPFVGWLSTTATLAEQAAMQARAAAAAFEAAFAMTVPPPAIAANRTLLMTLVDTNWFGQNTPAIATTESQYAEMWAQDAAAMYGYASAAAPATVLTPFAPPPQTTNATGLVGHATAVAALRGQHSWAAAIPWSDIQKYWMMFLGALATAEGFIYDSGGLTLNALQFVGGMLWXXRXXKKPVRPMAXXXAGGAAGWSAWSQLGAGPVAASATLAAKIGPMSVPPGWSAPPATPQAQTVARSIPGIRSAAEAAETSVLLRGAPTPGRSRAAHMGRRYGRRLTVMADRPNVG

>200|CORE_REP|Org59_Gene1665#

MDQQSTRTDITVNVDGFWMLQALLDIRHVAPELRCRPYVSTDSNDWLNEHPGMAVMREQGIVVNDAVNEQVAARMKVLAAPDLEVVALLSRGKLLYGVIDDENQPPGSRDIPDNEFRVVLARRGQHWVSAVRVGNDITVDDVTVSDSASIAALVMDGLESIXXAXPAAINAVNVPMEEMLEATKSWQESGFNVFSGGDLRRMGISAATVAALGQALSDPAAEVAVYARQYRDDAKXPSASXLSLKDGSGGRIALYQQARTAGXRRGXAGYLPXYPAVGASRSEDRFGYTALRRVENTQQSMTPGRETRSTTTNLSIRYNPDTYRANCSRIDCNTARQGQPQRFGREARXXKSELXEPQLPVGYRASVPTPTELPAPLKPRCNTFAMAGGTGR

>209|CORE_REP|Org59_Gene2935#

MDFALLPPEVNSARMYTGPGAGSLLAAAGGWDSLAAELATTAEAYGSVLSGLAALHWRGPAAESMAVTAAPYIGWLYTTAEKTQQTAIQARAAALAFEQAYAMXLPPPVVAANRIQLLALIATNFFGQNTAAIAATEAQYAEMWAQDAAAMYGYATASXAXALLTPGXXXPRQTTNPAGLTAQAAAVSQATDPLSLLIETVTQALQALTIPSFIPEDFTFLDAIFAGYATVGVTQDVESFVAGTIGAESNLGLLNVGDENPAEVTPGDFGIGELVSATSPGGGVSASGAGGAASVGNTVLASVGRANSIGQLSVPPSWAAPSTRPVSALSPAGLTTLPGTDVAEHGMPGVPGVPVAXGRASGVLPRYGVRLTVMAHPPAAGXPGA

>214|CORE_REP|Org118_Gene1514#

MYADRDLPGAGGLAVRVIPCLDVDDGRVVKGVNFENLRDAGDPVELAAVYDAEGADELTFLDVTASSSGRATMLEVVRRTAEQVFIPLTVGGGVRTVADVDSLLRAGADKVAVNTAAIACPDLLADMARQFGSQCIVLSVDARTVPVGSAPTPSGWEVTTHGGRRGTGMDAVQWAARGADLGVGEILLNSMDADGTKAGFDLALLRAVRAAVTVPVIASGGAGAVEHFAPAVAAGADAVLAASVFHFRELTIGQVKAALAAEGITVPMTLDPKIAARLKRNADGLVTAVVQERGSGDVLMVAWMNDEALARTLQTREATYYSRSRAEQWVKGATSGHTQHVHSVRLDCDGDAVLLTVDQVGGACHIGDHSCFDAAVLLEPDD

>223|CORE_REP|Org2_Gene4088#

MSSYYARRPLQSSGCSNSDSCWDGAPIEITESGPSVAGRLAALASRMTIKPLMTVGSYLSPLPLPLGFVDFACRVWRPGQGTVRTTINLPNATAQLVRAPGVRAADGAGRVVLYLHGGAFVMCGPNSHSRIVNALSGFAESPVLIVDYRLIPKHSLGMALDDCHDAYQWLRARGYRPEQIVLAGDSAGGYLALALAQRLQCDDEKPAAIVAISPLLQLAKGPKQDHPNIGTDAMFPARAFDALAAWVRAAAAKNMVDGRPEDLYEPLDHIESSLPPTLIHVSGSEVLLHDAQLGAGKLAAAGVCAEVRVWPGQAHLFQLATPLVPEATCSLRQIGQFIRDATADSSLSPVHRSRYVAGSPRAASRGAFGQSPI

>225|CORE_REP|Org100_Gene2093#

MSAHVATLHPEPPFALCGPRGTLIARGVRTRYCDVRAAQAALRSGTAPILLGALPFDVSRPAALMVPDGVLRARKLPDWPTGPLPKVRVAAALPPPADYLTRIGRARDLLAAFDGPLHKVVLARAVQLTADAPLDARVLLRRLVVADPTAYGYLVDLTSAGNDDTGAALVGASPELLVARSGNRVMCKPFAGSAPRAADPKLDAANAAALASSAKNRHEHQLVVDTMRVALEPLCEDLTIPAQPQLNRTAAVWHLCTAITGRLRNISTTAIDLALALHPTPAVGGVPTKAATELIAELEGDRGFYAGAVGWCDGRGDGHWVVSIRCAQLSADRRAALAHAGGGIVAESDPDDELEETTTKFATILTALGVEQ

>226|CORE_REP|Org59_Gene452#

MQLTPHFGNVQAHYDLSDDFFRLFLDPTQTYSCAYFERDDMTLQEAQIAKIDLALGKLNLEPGMTLLDIGCGWGATMRRAIEKYDVNVVGLTLSENQAGHVQKMFDQMDTPRSRRVLLEGWEKFDEPVDRIVSIGAFEHFGHQRYHHFFEVTHRTLPADGKMLLHTIVRPTFKEGREKGLTLTHELVHFTKFILAEIFPGGWLPSIPTVHEYAEKVGFRVTAVQSLQLHYARTLDMWATALEANKDQAIAIZSQTVYBSLHEVPDRLREAVPPGLHRRRPVHTGKVTGQSALAXXRPVPGRXATPGVSSATPGTXSGGDGLXGQCELSHVADALAEEVLTSGQIVHVFVVNLLGLKSNGAVLVSLQIRRPDV

>238|CORE_REP|Org3_Gene1855#

MANVQYSAVTQRYPGADAPTVDNLDLDIADGEFLVLVGPSGCGKSTTLRVLAGLEPIESGRISIGDVDVTHLPPRARDVAMVFQNYALYPNMTVAANMGFALRNAGMSRADTRRRVLEVADMLELTDLLDRKPAKLSGGQRQRVAMGRAIVRRPRVFCMDEPLSNLDAKLRVSTRSQISGLQRRLGTTTVYVTHDQVEAMTMGDRVAVLKDGVLQQVDTPRALYDDPVNTFVATFIGAPAMNLIDAAVAHGVVRAPDLAIPVPDPAAERVLVGVRPESWDVASIGTPGSLTVHVELVEELGFESFVYATPVDQRGWSSRAPRIVFRTDRRTAVRVGESLAIVPHSQEVRLFNSRTETRLR

>245|CORE_REP|Org118_Gene2226#

MTSRETRAADAAGARQADAQVRSSIDVPPDLVVGLLGSADENLRALERTLSADLHVRGNAVTLCGEPADVALAERVISELIAIVASGQSLTPEVVRHSVAMLVGTGNESPAEVLTLDILSRRGKTIRPKTLNQKRYVDAIDANTIVFGIGPAGTGKTYLAMAKAVHALQTKQVTRIILTRPAVEAGERLGFLPGTLSEKIDPYLRPLYDALYDMMDPELIPKLMSAGVIEVAPLAYMRGRTLNDAFIVLDEAQNTTAEQMKMFLTRLGFGSKVVVTGDVTQIDLPGGARSGLRAAVDILEDIDDIHIAELTSVDVVRHRLVSEIVGRLCAVRGARVGAESGGSAGVRRPRSPMMGA

>258|CORE_REP|Org108_Gene1099#

MVLQELWFGVIAALFLGFFILEGFDFGVGMLMAPFAHVGMGDPETHRRTALNTIGPVWDGNEVWLITAGAAIFAAFPGWYATVFSALYLPLLAILFGMILRAVAIEWRGKIDDPKWRTGADFGIAAGSWLPALLWGVAFAILVRGLPVDANGHVALSIPDVLNAYTLLGGLATAGLFSLYGAVFIALKTSGPIRDDAYRFAVWLSLPVAGLVAGFGLWTQLAYGKDWTWLVLAVAGCAQAAATVLVWRRVSDGWAFMCTLIVVAAVVVLLFGALYPNLVPSTLNPQWSLTIHNASSTPYTLKIMTWVTAFFAPLTVAYQTWTYWVFRQRISAERIPPPTGLARRAP

>271|CORE_REP|Org14_Gene116#

MTFFEQVRRLRSAATTLPRRLAIAAMGAVLVYGLVGTFGGPATAGAFSRPGLPVEYLQVPSASMGRDIKVQFQGGGPHAVYLLDGLRAQDDYNGWDINTPAFEEYYQSGLSVIMPVGGQSSFYTDWYQPSQSNGQNYTYKWETFLTREMPAWLQANKGVSPTGNAAVGLSMSGGSALILAAYYPQQFPYAASLSGFLNPSEGWWPTLIGLAMNDSGGYNANSMWGPSSDPAWKRNDPMVQIPRLVANNTRIWVYCGNGTPSDLGGDNIPAKFLEGLTLRTNQTFRDTYAADGGRNGVFNFPPNGTHSWPYWNEQLVAMKADIQHVLNGATPPAAPAAPAA

>281|CORE_REP|Org119_Gene2767#

MAGAKHAGRIVAITTAAAVILAACSSGSKGGAGSGHAGKARSAVTTTDADWKPVADALGRSGKLGDNNTAYRINLPRNDLHITSYGVDIKPGLSLGGYAAFARYDNNETLLMGDLVITEEELPKVTDALQAHGIAQTALHKHLLQQDPPVWWTHIHGMGDAARLAQGLKAALDATTIGPPTPPPARQPPVDIDVAGVDQALGRKGTQDGGLLKYSIPRKDTIIEDGHVLPAVSLNLTTVINFQPVGRGRAAINGDFILIAPEVQEVIRAMRAGNITIVELHNHGLTEEPRLFYMHYWAVDDAVTLARALRPAMECHQPAVVIIPMQPHKGWCG

>283|CORE_REP|Org59_Gene2384#

MGGLTISDLVVEYSSGGXAVRPIDGXKPRRGAGVAGDLAWAQRLREDDPLVLPRRXXCARSPAQSSLTMSTSSNLXEGAALAKYRRDKXGIVFQAFNLVSSLTALENVMVPLRAAGVSRAAARKRAEDLLIRVNLGERMKHRPGDMSGGQQQRVAVARAIALDPQLILADEPTAHLDFIQVEEVLRLIRSLAQGDRVVVVATHDSRMLPLADRVLELMPAQVSPNQPPETVHVKAGEVLFEQSTMGDLIYVVSEGEFEIVRELADGGEELVKXAAPGDYFGEIGVLFXLPRSATVRARSDATAVGYTAQAFRERLGXXRXXDLIEHRELASE

>284|CORE_REP|Org18_Gene2243#

MRLLVTGGAGFIGTNFVHSAVREHPDDAVTVLDALTYAGRRESLADVEDAIRLVQGDITDAELVSQLVAESDAVVHFAAESHVDNALDNPEPFLHTNVIGTFTILEAVRRHGVRLHHISTDEVYGDLELDDRARFTESTPYNPSSPYSATKAGADMLVRAWVRSYGVRATISNCSNNYGPYQHVEKFIPRQITNVLTGRRPKLYGAGANVRDWIHVDDHNSAVRRILDRGRIGRTYLISSEGERDNLTVLRTLLRLMDRDPDDFDHVTDRVGHDLRYAIDPSTLYDELCWAPKHTDFEEGLRTTIDWYRDNESWWRPLKDATEARYQERGQ

>285|CORE_REP|Org146_Gene224#

MNPIPSWPGRGRVTLVLLAVVPVALAYPWQSTRDYVLLGVAAAVVIGLFGFWRGLYFTTIARRGLAILRRRRRIAEPATCTRTTVLVWVGPPASDTNVLPLTLIARYLDRYGIRADTIRITSRVTASGDCRTWVGLTVVADDNLAALQARSARIPLQETAQVAARRLADHLREIGWEAGTAAPDEIPALVAADSRETWRGMRHTDSDYVAAYRVSADAELPDTLPAIRSRPAQETWIALEIAYAAGSSTRYTVAAACALRTDWRPGGTAPVAGLLPQHGNHVPALTALDPRSTRRLDGHTDAPADLLTRLHWPTPTAGAHRAPLTNAVSRT

>311|CORE_REP|Org118_Gene1438#

MNAHTSVGPLDRAARVYIAGHRGLVGSALLRTFAGAGFTNLLVRSRAELDLTDRAATFDFVLESRPQVVIDAAARVGGILANDTYPADFLSENLQIQVNLLDAAVAARVPRLLFLGSSCIYPKLAPQPIPESALLTGPLEPTNDAYAIAKIAGILAVQAVRRQHGLPWISAMPTNLYGPGDNFSPSGSHLLPALIRRYDEAKASGAPNVTNWGTGTPRRELLHVDDLASACLYLLEHFDGPTHVNVGTGIDHTIGEIAEMVASAVGYSGETRWDPSKPDGTPRKLLDVSVLREAGWRPSIALRDGIEATVAWYREHAGTVRQ

>325|CORE_REP|Org59_Gene286#

MDATPNAVELTVDNAWFIAETIGAGTFPWVLAITMPYSDAAQRGAFVDRQRDELTRMGLLSPQGVINPAVADWIKVVCFPDRWLDLRYVGPASADGACELLRGIVALRTGTGKTSNKTGNGVVALRNAQLVTFTAMDIDDPRALVPILGXGLAHRPPARFDEFSLPTRVGARADERLRSGVPLGEVVDYLGIPASARPVVESVFSGPRSYVEIVAGCNRSXERRXHHHRGRPKHRRHLGGPGVGESVAGIRRRVGLHLQPWDTVCDRRRDPNTDRVLARRAMVPGTAGVAGLLHPILVIRNQKVSTMSQERSR

>339|CORE_REP|Org30_Gene950#

MDRCCQRATAFACALRPTKLIDYEEMFRGAMQARAMVANPDQWADSDRDQVNTRHYLSTSMRVALDRGEFFLVYQPIIRLADNRIIGAEALLRWEHPTLGTLLPGRFIDRAENNGLMVPLTAFVLEQACRHVRSWRDHSTDPQPFVSVNVSASTICDPGFLVLVEGVLGETGLPAHALQLELAEDARLSRDEKAVTRLQELSALGVGIAIDDFGIGFSSLAYLPRLPVDVVKLGGKFIECLDGDIQARLANEQITRAMIDLGDKLGITVTAKLVETPSQAARLRAFGCKAAQGWHFAKALPVDFFRE

>343|CORE_REP|Org46_Gene713#

MSRPEVLTPFTAIVPAAGLGTRFLPATKTVPKELLPVVDTPGIELVAAEAAAAGAERLVIVTSEGKDGVVAHFVEDLVLEGTLEARGKIAMLAKVRRAPALIKVESVVQAEPLGLGHAIGCVEPTLSPDEDAVAVLLPDDLVLPTGVLETMSKVRASRGGTVLCAIEVAREEISAYGVFDVEPVPDGDYTDDPNVLKVRGMVEKPKAETAPSRYAAAGRYVLDRAIFDALRRIDRGAGGEVQLTDAIALLIAEGHPVHVVVHQGSRHDLGNPGGYLKAAVDFALDRDDYGPDLRRWLVARLGLTEQ

>349|CORE_REP|Org98_Gene3124#

MAGRSERLVITGAGGQLGSHLTAQAAREGRDMLALTSSQWDITDPAAAERIIRHGDVVINCAAYTDVDGAESNEAVAYAVNATGPQHLARACARVGARLIHVSTDYVFDGDFGGAEPRPYEPTDETAPQGVYARSKLAGEQAVLAAFPEAAVVRTAWVYTGGTGKDFVAVMRRLAAGHGRVDVVDDQTGSPTYVADLAEALLALADAGVRGRVLHAANEGVVSRFGQARAVFEECGADPQRVRPVSSAQFPRPAPRSSYSALSSRQWALAGLTPLRHWRSALATALAAPANSTSIDRRLPSTRD

>354|CORE_REP|Org6_Gene2975#

MSGNEVHPDLRRIAVVTPRQLVGPRTLPVMRALIVVAGLRMSRTPPDIEVLTLESGVGVRLYRPAGSNEPAPALLWIHAGGYVMGTAQQDDRLCLRFSSRLGITVASVDYRLAPENPYPAALGDCYSALTWLASLPAVDPARVAIGGASAGGGLAAALALLARDRGGITPAFQLLVYPMLDDRPSIAPANPHYRLWNGRANRFGWRAYLGDADARVAVPGRRDDLGGLAPAWIGVGTHDLLHDEDLAYAERLTAAGVPCQVEVVEGAFHGFDRVAPNVGVSQRFFTSQCNSLRAALALSNRT

>357|CORE_REP|Org149_Gene3036#

MTRMAEKPISPTKTRTRFEDIQAHYDVSDDFFALFQDPTRTYSCAYFEPPELTLEEAQYAKVDLNLDKLDLKPGMTLLDIGCGWGTTMRRAVERFDVNVIGLTLSKNQHARCEQVLASIDTNRSRQVLLQGWEDFAEPVDRIVSIEAFEHFGHENYDDFFKRCFNIMPADGRMTVQSSVSYHPYEMAARGKKLSFETARFIKFIVTEIFPGGRLPSTEMMVEHGEKAGFTVPEPLSLRPHYIKTLRIWGDTLQSNKDKAIEVTSEEVYNRYMKYLRGCEHYFTDEMLDCSLVTYLKPGAAA

>358|CORE_REP|Org4_Gene3919#

MGWRDAPALSDYQHVASGKVREIYRVDDEHLLLVASDRISAYDYVLDSTIPDKGRVLTAMSAFFFGLVDAPNHLAGPPDDPRIPDEVLGRALVVRRLEMLPVECVARGYLTGSGLLDYQATGKVCGIALPPGLVEASRFATPLFTPATKAALGDHDENISFDRVVEMVGALRANQLRDRTLQTYVQAADHALTRGIIIADTKFEFGIDRHGNLLLADEIFTPDSSRYWPADDYRAGVVQTSFDKQFVRSWLTGSESGWDRGSDRPPPPLPEHIVEATRARYINAYERISELKFDDWIGPGA

>374|CORE_REP|Org119_Gene587#

MAKLRPYYEESQSAYDISDDFFALFLDPTWVYTCAYFERDDMTLEEAQLAKVDLALDKLNLEPGMTLLDVGCGWGGALVRAVEKYDVNVIGLTLSRNHYERSKDRLAAIGTQRRAEARLQGWEEFEENVDRIVSFEAFDAFKKERYLTFFERSYDILPDDGRMLLHSLFTYDRRWLHEQGIALTMSDLRIPQIPAGVDLPGRRAAIGARHCRQCAGRGLHHRACPAAAAALRTDSRCMGRQPTGCPRTRHRRTVRRGLQQLHALSDRMRGALPQRPNQRRPVHHDQVARPLISVP

>385|CORE_REP|Org72_Gene3626#

MRDAPRRRTALAYALLAPSLVGVVAFLLLPILVVVWLSLHRWDLLGPLRYVGLTNWRSVLTDSGFADSLVVTAVFVAIVVPAQTVLGLLAASLLARRLPGTGLFRTLYVLPWICAPLAIAVMWRWIVAPTDGAISTVLGHRIEWLTDPGLALPVVSAVVVWTNVGYVSLFFLAGLMAIPQDIHNAARTDGASAWQRFWRITLPMLRPTMFFVLVTGIISAAQVFDTVYALTGGGPQGSTDLVAHRIYAEAFGAAAIGRASVMAVVLFVILVGATVVQHLYFRRRISYELT

>388|CORE_REP|Org118_Gene328#

MRGIILAGGSGTRLYPITMGISKQLLPVYDKPMIYYPLTTLMMAGIRDIQLITTPHDAPGFHRLLGDGAHLGVNISYATQDQPDGLAQAFVIGANHIGADSVALVLGDNIFYGPGLGTSLKRFQSISGGAIFAYWVANPSAYGVVEFGAEGMALSLEEKPVTPKSNYAVPGLYFYDNDVIEIARGLKKSARGEYEITEVNQVYLNQGRLAVEVLARGTAWLDTGTFDSLLDAADFVRTLERRQGLKVSMPRRSGVAHGLDRRRAAGVQRARALVKSGYGNYLLELLERN

>405|CORE_REP|Org119_Gene3336#

MSPAPVQVMGVLNVTDDSFSDGGCYLDLDDAVKHGLAMAAAGAGIVDAGGGESSRPGATRVDPAVETSRVIPVVKELAAQGITVSIDTMRADVARAALQNGAQMVNDVSGGRADPAMGPLLAEADVPWVLMHWRAVSADTPHVPVRYGNVVAEVRADLLASVADAVAAGVDPARLVLDPGLGFAKTAQHNWAILHALPELVATGIPVLVGASRKRFLGALLAGPDGVMRPTDGRDTATAVISALAALHGAWGVRVHDVRASVDAIKVVEAWMGAERIERDG

>439|CORE_REP|Org75_Gene2351#

MLLAIDVRNTHTVVGLLSGMKEHAKVVQQWRIRTESEVTADELALTIDGLIGEDSERLTGTAALSTVPSVLHEVRIMLDQYWPSVPHVLIEPGVRTGIPLLVDNPKEVGADRIVNCLAAYDRFRKAAIVVDFGSSICVDVVSAKGEFLGGAIAPGVQVSSDAAAARSAALRRVELARPRSVVGKNTVECMQAGAVFGFAGLVDGLVGRIREDVSGFSVDHDVAIVATGHTAPLLLPELHTVDHYDQHLTLQGLRLVFERNLEVQRGRLKTAR

>442|CORE_REP|Org59_Gene2732#

MSMLARHGPRYGGSXNGHSDXSXGXAKXAAPTLYIFPHAGGTAKDYVAFSREFSADVKRIAVQYPGQHDRSGLPPLESIPTLADEIFAMMKPSARIDDPVAFFGHSMGGMLAFEVALRYQSAGHRVLAFFVSXXSAPGHIRYKQLQDLSDREMLDLFTRMTGMNPDFFTDXEFFVGALPTLRAVRAIAGYSCPPETKLSCPIYAFIGDKDWIATQDDMDPWRDRTTEEFXIRVFPGDHFYLNDNLPELVSDIEDKTLQWLXSXPXLCSGCS

>447|CORE_REP|Org119_Gene1362#

MTILEIKDLHVSVENPAEADHEIPILRGVDLTVKSGETHALMGPNGSGKSTLSYAIAGHPKYHVTSGTITLDGADVLAMSIDERARAGLFLAMQYPVEVPGVSMSNFLRSAATAIRGEPPKLRHWVKEVKAAMAALDIDPAFAERSVNEGFSGGEKKRHEILQLELLKPKIAILDETDSGPGRRRAARGQRGGEPLRRIPARRHPADHALHPHPALHPPGIRARVRRRPHRRVRWFGARRRTRPERLRAFLPRKRAVPPPTRANRSLT

>450|CORE_REP|Org69_Gene3393#

MTDTRVLAVANQKGGVAKTTTVASLGAAMVEKGRRVLLVDLDPQGCLTFSLGQDPDKLPVSVHEVLLGEVEPNAVLVTTMEGMTLLPANIDLAGAEAMLLMRAGREYALKRALAKFSDRFDVVIIDCPPSLGVLTLNGLTAADEAIVPLQCEMLAHRGVGQFLRTVADVQQITNPNLRLLGALPTLYDSRTTHTRDVLLDVADRYDLQVLAPPIPRTVRFAEASASGSSVMAGRKNKGAVAYRELAQALLKHWKTGRPLPTFTVDL

>482|CORE_REP|Org31_Gene855#

MELLGGPRVGNTESQLCVADGDDLPTYCSANSEDLNITTITTLSPTSMSHPQQVRDDQWVEPSDQLQGTAVFDATGDKATMPSWDELVRQHADRVYRLAYRLSGNQHDAEDLTQETFIRVFRSVQNYQPGTFEGWLHRITTNLFLDMVRRRARIRMEALPEDYDRVPADEPNPEQIYHDARLGPDLQAALASLPPEFRAAVVLCDIEGLSYEEIGATLGVKLGTVRSRIHRGRQALRDYLAAHPEHGECAVHVNPVR

>483|CORE_REP|Org22_Gene721#

MTTMSGYTRSQRPRQAILGQLPRIHRADGSPIRVLLVDDEPALTNLVKMALHYEGWDVEVAHDGQEAIAKFDKVGPDVLVLDIMLPDVDGLEILRRVRESDVYTPTLFLTARDSVMDRVTGLTSGADDYMTKPFSLEELVARLRGLLRRSSHLERPADEALRVGDLTLDGASREVTRDGTPISLSSTEFELLRFLMRNPRRALSRTEILDRVWNYDFAGRTSIVDLYISYLRKKIDSDREPMIHTVRGIGYMLRPPE

>489|CORE_REP|Org35_Gene2559#

MMISSSDELLRDGADPAVIIDQLRVIRGKRLALQDVSVRVACGTITGLLGPSGSGKTTLIRCIVGSQIIASGSVSVLGQPAGSAELRHRVGYMPQDPTIYNDLRVIDNIRYFAELCGVDRQAADEVIEAVDLRDHRTARCANLSGGQRARVSLACALVGRPDLLVLDEPTIGLDPVLRVELWDRFTALARRGTTLLVSSHVMDEADRCGDLLLLRQGQLLAHTTPHRLRKETGCTSLEEAFLSIVRRTTTVPAAG

>502|CORE_REP|Org111_Gene1716#

MSGHSKWATTKHKKAVVDARRGKMFARLIKNIEVAARVGGGDPAGNPTLYDAIQKAKKSSVPNENIERARKRGAGEEAGGADWQTIMYEGYAPNGVAVLIECLTDNRNRAASEVRVAMTRNGGTMADPGSVSYLFSRKGVVTLEKNGLTEDDVLAAVLEAGAEDVNDLGDSFEVISEPAELVAVRSALQDAGIDYESAEASFQPSVSVPVDLDGARKVFKLVDALEDSDDVQNVWTNVDVSDEVLAALDDE

>526|CORE_REP|Org47_Gene1050#

MRLARRARNILRRNGIEVSRYFAELDWERNFLRQLQSHRVSAVLDVGANSGQYARGLRGAGFAGRIVSFEPLPGPFAVLQRSASTDPLWECRRCALGDVDGTISINVAGNEGASSSVLPMLKRHQDAFPPANYVGAQRVPIHRLDSVAADVLRPNDIAFLKIDVQGFEKQVIAGGDSTVHDRCVGMQLELSFQPLYEGGMLIREALDLVDSLGFTLSGLQPGFTDPRNGRMLQADGIFFRGSD

>527|CORE_REP|Org83_Gene3136#

MPNFWALPPEINSTRIYLGPGSGPILAAAQGWNALASELEKTKVGLQSALDTLLESYRGQSSQALIQQTLPYVQWLTTTAEHAHKTAIQLTAAANAYEQARAAMVPPAMVRANRVQTTVLKAINWFGQFSTRIADKEADYEQMWFQDALVMENYWEAVQEAIQSTSHFEDPPEMADDYDEAWMLNTVFDYHNENAKEEVIHLVPDVNKERGPIELVTKVDKEGTIRLVYDGEPTFSYKEHPKF

>579|CORE_REP|Org147_Gene3012#

MDTMRQRILVVDDDASLAEMLTIVLRGEGFDTAVIGDGTQALTAVRELRPDLVLLDLMLPGMNGIDVCRVLRADSGVPIVMLTAKTDTVDVVLGLESGADDYIMKPFKPKELVARVRARLRRNDDEPAEMLSIADVEIDVPAHKVTRNGEQISLTPLEFDLLVALARKPRQVFTRDVLLEQVWGYRHPADTRLVNVHVQRLRAKVEKDPENPTVVLTVRGVGYKAGPP

>587|CORE_REP|Org133_Gene366#

MTSVLIVEDEESLADPLAFLLRKEGFEATVVTDGPAALAEFDRAGADIVLLDLMLPGMSGTDVCKQLRARSSVPVIMVTARDSEIDKVVGLELGADDYVTKPYSARELIARIRAVLRRGGDDDSEMSDGVLESGPVRMDVERHVVSVNGDTITLPLKEFDLLEYLMRNSGRVLTRGQLIDRVWGADYVGDTKTLDVHVKRLRSKIEADPANPVHLVTVRGLGYKLEG

>591|CORE_REP|Org40_Gene740#

MTLVLVIDDEPQILRALRINLTVRGYQVITASTGAGALRAAAEHPPDVVILDLGLPDMSGIDVLGGLRGWLTAPVIVLSARTDSSDKVQALDAGADDYVTKPFGMDEFLARLRAAVRRNTAAAELEQPVIETDSFTVDLAGKKVIKDGAEVHLTPTEWGMLEMLARNRGKLVGRGELLKEVWGPAYATETHYLRVYLAQLRRKLEDDPSHPKHLLTESGMGYRFEA

>617|CORE_REP|Org59_Gene1254#

MAPDRADDDAERSDEEEWRLMTKLXVASRNRKKLAELRRVLDGAGLSGXTXLSXGDVSPLPETPETGVTFEDNALAKARDAFSATGLASVADDSGLEVAALGGMPGVLSARWSGRYGDDAANTALLLAQLCDVPDERRGAAFVSACALVSGSGEVVVRGEWPGTIAREPRGDGGFGYDPVFVPYGDDRTAAQLSPAEKDAVSHRGRALALLLPALRSLATG

>635|CORE_REP|Org109_Gene2034#

MVKVFLVDDHEVVRRGLVDLLGADPELDVVGEAGSVAEAMARVPAARPDVAVLDVRLPDGNGIELCRDLLSRMPDLRCLILTSYTSDEAMLDAILAGASGYVVKDIKGMELARAVKDVGAGRSLLDNRAAAALMAKLRGAAEKQDPLSGLTDQERTLLGLLSEGLTNKQIADRMFLAEKTVKNYVSRLLAKLGMERRTQAAVFATELKRSRPPGDGP

>636|CORE_REP|Org43_Gene263#

MTISFSSSNLRDDATSGNGDYRLDKLPETTPSTSVFDRADVTYRQFTELHGQARDTRREAHVVELESKTGERARCAPMHALEQLADYGFAWRDIARVVGVSVPAITKWRKGAGVTGENRLKIARLLALIDMLSDRFIGEPASWLEMPIQAGVGITRMDLLERGRYDLVLALASTHTGDGTVEYVLNETDKDWRETVVDNAFESYTAEDGVISIRPKR

>654|CORE_REP|Org34_Gene3162#

MPLFSFEGRSPRIDPTAFVAPTATLIGDVTIEAGASVWFNAVLRGDYAPVVVREGANVQDGAVLHAPPGIPVDIGPGATVAHLCVIHGVHVGSEALIANHATVLDGAVIGARCMIAAGALVVAGTQIPAGMLVTGAPAKVKGPIEGTGAEMWVNVNPQAYRDLAARHLAGLEPMQASLRVKPSAVLTSGCKSRSRRRRRRCPPPHQLGRRVR

>661|CORE_REP|Org9_Gene938#

MTKPTSAGQADDALVRLARERFDLPDQVRRLARPPVPSLEPPYGLRVAQLTDAEMLAEWMNRPHLAAAWEYDWPASRWRQHLNAQLEGTYSLPLIGSWHGTDGGYLELYWAAKDLISHYYDADPYDLGLHAAIADLSKVNRGFGPLLLPRIVASVFANEPRCRRIMFDPDHRNTATRRLCEWAGCKFLGEHDTTNRRMALYALEAPTTAA

>670|CORE_REP|Org86_Gene1143#

MAAPDNSRRRPGRPAGSSDTRERILSSARELFAHNGIDRTSIRAVAAKAGVDAALVHHYFGTKQQLFAAAIHIPIDPMVIIGPIREAPVEELGYKLPSLLLPIWDSELGAGLIATLRSLISGSDVGLARSFLEEVVTVELGSRVDNPPGTGKIRTQFVASQLMGVVMARYIVRIEPFASLPAEQIVQTIAPNLQRYLTGELPDDLAP

>686|CORE_REP|Org120_Gene3695#

MKARELDVPGAWEITPTIHVDSRGLFFEWLTDHGFRAFAGHSLDVRQVNCSVSSAGVLRGLHFAQLPPSQAKYVTCVSGSVFDVVVDIREGSPTFGRWDSVLLDDQDRRTIYVSDGLAHGFLALQDNSTVMYLCSAEYNPQREHTICATDPTLAVDWPLVDGAAPSLSDRDAAAPSFEDVRASGLLPRWEQTQRFIGEMRGT

>696|CORE_REP|Org10_Gene3899#

MTECFLSDQEIRKLNRDLRILIAANGTLTRVLNIVADDEVIVQIVKQRIHDVSPKLSEFEQLGQVGVGRVLQRYIILKGRNSEHLFVAAESLIAIDRLPAAIITRLTQTNDPLGEVMAASHIETFKEEAKVWVGDLPGWLALHGYQNSRKRAVARRYRVISGGQPIMVVTEHFLRSVFRDAPHEEPDRLQFSNAITLAR

>698|CORE_REP|Org41_Gene1966#

MEAFHTHSGIGVPLRRSNVDTDQIIPAVFLKRVTRTGFEDGLFAGWRSDPAFVLNLSPFDRGSVLVAGPDFGTGSSREHAVWALMDYGFRVVISSRFGDIFRGNAGKAGLLAAEVAQDDVELLWKLIEQSPGLEITANLQDRIITAATVVLPFKIDDHSAWRLLEGLDDIALTLRKLDEIEAFEGACAYWKPRTLPAP

>703|CORE_REP|Org1_Gene1468#

MTVTDDYLANNVDYASGFKGPLPMPPSKHIAIVACMDARLDVYRMLGIKEGEAHVIRNAGCVVTDDVIRSLAISQRLLGTREIILLHHTDCGMLTFTDDDFKRAIQDETGIRPTWSPESYPDAVEDVRQSLRRIEVNPFVTKHTSLRGFVFDVATGKLNEVTPSSPSRQPRAHWRTGSPPRWGCVDSDREAWLHRWQ

>762|CORE_REP|Org119_Gene660#

MSRIGKQPIPVPAGVDVTIEGQSISVKGPKGTLGLTVAEPIKVARNDDGAIVVTRPDDERRNRSLHGLSRTLVSNLVTGVTQGYTTKMEIFGVGYRVQLKGSNLEFALGYSHPVVIEAPEGITFAVQAPTKFTVSGIDKQKVGQIAANIRRLRRPDPSKRGKGVRYEGEQIRRKVGKTGK

>808|CORE_REP|Org117_Gene1268#

MAQITLRGNAINTVGELPAVGSPAPAFTLTGGDLGVISSDQFRGKSVLLNIFPSVDTPVCATSVRTFDERAAASGATVLCVSKDLPFAQKRFCGAEGTENVMPASAFRDSFGEDYGVTIADGPMAGLLARAIVVIGADGNVAYTELVPEIAQEPNYEAALAALGA

>828|CORE_REP|Org68_Gene1008#

MTLRLEQIYQDVILDHYKHPQHRGLREPFGAQVYHVNPICGDEVTLRVALSEDGTRVTDVSYDGQGCSISQAATSVLTEQVIGQRVPRALNIVDAFTEMVSSRGTVPGDEDVLGDGVAFAGVAKYPARVKCALLGWMAFKDALAQASEAFEEVTDERNQRTG

>833|CORE_REP|Org36_Gene1960#

MTGAVCPGSFDPVTLGHVDIFERAAAQFDEVVVAILVNPAKTGMFDLDERIAMVKESTTHLPNLRVQVGHGLVVDFVRSCGMTAIVKGLRTGTDFEYELQMAQMNKHIAGVDTFFVATAPRYSFVSSSLAKEVAMLGGDVSELLPEPVNRRLRDRLNTERT

>852|CORE_REP|Org101_Gene1661#

MTKTTRLTPGDKAPAFTLPDADGNNVSLADYRGRRVIVYFYPAASTPGCTKQACDFRDNLGDFTTAGLNVVGISPDKPEKLATFRDAQGLTFPLLSDPDREVLTAWGAYGEKQMYGKTVQGVIRSTFVVDEDGKIVVAQYNVKATGHVAKLRRDLSV

>872|CORE_REP|Org57_Gene2255#

MTETTPAPQTPAAPAGPAQSFVLERPIQTVGRRKEAVVRVRLVPGTGKFDLNGRSLEDYFPNKVHQQLIKAPLVTVDRVESFDIFAHLGGGGPSGQAGALRLGIARALILVSPEDRPALKKAGFLTRDPRATERKKYGLKKARKAPQYSKR

>932|CORE_REP|Org135_Gene2330#

MLRTMLKSKIHRATVTCADLHYVGSVTIDADLMDAADLLEGEQVTIVDIDNGARLVTYAITGERGSGVIGINGAAAHLVHPGDLVILIAYATMDDARARTYQPRIVFVDAYNKPIDMGHDPAFVPENAGELLDPRLGVG

>1052|CORE_REP|Org135_Gene38#

MTDSEKSATIKVTDASFATDVLSSNKPVLVDFWATWCGPCKMVAPVLEEIATERATDLTVAKLDVDTNPETARNFQVVSIPTLILFKDGQPVKRIVGAKGKAALLRELSDVVPNLN

>1109|CORE_REP|Org58_Gene1232#

MRLTPHEQERLLLSYAAELARRRRARGLRLNHPEAIAVIADHILEGARDGRTVAELMASGREVLGRDDVMEGVPEMLAEVQVEATFPDGTKLVTVHQPIA

>1130|CORE_REP|Org107_Gene221#

MSQIMYNYPAMLGHAGDMAGYAGTLQSLGAEIAVEQAALQSAWQGDTGITYQAWQAQWNQAMEDLVRAYHAMSSTHEANTMAMMARDTAEAAKWGG

>1214|CORE_REP|Org69_Gene2244#

MAKKDGAIEVEGRVVEPLPNAMFRIELENGHKVLAHISGKMRQHYIRILPEDRVVVELSPYDLSRGRIVYRYK
