# Supplementary material for: Pangenome Analysis of Mycobacterium tuberculosis Reveals Core-Drug Targets and Screening of Promising Lead Compounds for Drug Discovery
Source: Antibiotics (Basel). 2020 Nov 17;9(11):819. doi: 10.3390/antibiotics9110819 (PMC7698547; doi:10.3390/antibiotics9110819)
Supplement: Supplementary file 1 [file antibiotics-09-00819-s001.zip › antibiotics-984987-supplementary/File S4.docx]

**File S4:** Protein targets found to have appropriate physiochemical parameters provided in Fasta format below:

>8|CORE_REP|Org1_Gene2938#

MQRFGTGSSRSWCGRAGTATIAAVLLASGALTGLPPAYAISPPTIDPGALPPDGPPGPLAPMKQNAYCTEVGVLPGTDFQLQPKYMEMLNLNEAWQFGRGDGVKVAVIDTGVTPHPRLPRLIPGGDYVMAGGDGLSDCDAHGTLVASMIAAVPANGAVPLPSVPRRPVTIPTTETPPPPQTVTLSPVPPQTVTVIPAPPPEEGVPPGAPVPGPEPPPAPGPQPPAVDRGGGTVTVPSYSGGRKIAPIDNPRNPHPSAPSPALGPPPDAFSGIAPGVEIISIRQSSQAFGLKDPYTGDEDPQTAQKIDNVETMARAIVHAANMGASVINISDVMCMSARNVIDQRALGAAVHYAAVDKDAVIVAAAGDGSKKDCKQNPIFDPLQPDDPRAWNAVTTVVTPSWFHDYVLTVGAVDANGQPLSKMSIAGPWVSISAPGTDVVGLSPRDDGLINAIDGPDNSLLVPAGTSFSAAIVSGVAALVRAKFPELSAYQIINRLIHTARPPARGVDNQVGYGVVDPVAALTWDVPKGPAEPPKQLSAPLVVPQPPAPAIWCRYGWPPGDWPGTIDRRCGVRYRDLDAAITEAAMKAQRSFGLALSWPRVTAVFLVDVLILAVASHCPDSWQADHHVAWWVGVGVAAVVTLLSVVSYHGITVISGLATWVRDWSADPGTTLGAGCTPAIDHQRRFGRDTVGVREYNGRLVSVIEVTCGESGPSGRHWHRKSPVPMLPVVAVADGLRQFDIHLDGIDIVSVLVRGGVDAAKASASLQEWEPQGWKSEERAGDRTVADRRRTWLVLRMNPQRNVAAVACRDSLASTLVAATERLVQDLDGQSCAARPVTADELTEVDSAVLADLEPTWSRPGWRHLKHFNGYATSFWVTPSDITSETLDELCLPDSPEVGTTVVTVRLTTRVGSPALSAWVRYHSDTRLPKEVAAGLNRLTGRQLAAVRASLPAPTHRPLLVIPSRNLRDHDELVLPVGQELEHATSSFVGQ

>9|CORE_REP|Org10_Gene4001#

MSGTPDDGDIGLIIAVKRLAAAKTRLAPVFSAQTRENVVLAMLVDTLTAAAGVGSLRSITVITPDEAAAAAAAGLGADVLADPTPEDDPDPLNTAITAAERVVAEGASNIVVLQGDLPALQTQELAEAISAARHHRRSFVADRLGTGTAVLCAFGTALHPRFGPDSSARHRRSGAVELTGAWPGLRCDVDTPADLTAARQLGGRARDRASGRTSLTGTGQRRRGIQGGERQTNGERMPAECWQPHPMMSNDRKVTEIENSPVTEVRPEEHAWYPDDSALAAPPAATPAAISDQLPSDRYLNRELSWLDFNARVLALAADKSMPLLERAKFLAIFASNLDEFYMVRVAGLKRRDEMGLSVRSADGLTPREQLGRIGEQTQQLASRHARVFLDSVLPALGEEGIYIVTWADLDQAERDRLSTYFNEQVFPVLTPLAVDPAHPFPFVSGLSLNLAVTVRQPEDGTQHFARVKVPDNVDRFVELAAREASEEAAGTEGRTALRFLPMEELIAAFLPVLFPGMEIVEHHAFRITRNADFEVEEDRDEDLLQALERELARRRFGSPVRLEIADDMTESMLELLLRELDVHPGDVIEVPGLLDLSSLWQIYAVDRPTLKDRTFVPATHPAFAERETPKSIFATLREGDVLVHHPYDSFSTSVQRFIEQAAADPNVLAIKQTLYRTSGDSPIVRALIDAAEAGKQVVALVEIKARFDEQANIAWARALEQAGVHVAYGLVGLKTHCKTALVVRREGPTIRRYCHVGTGNYNSKTARLYEDVGLLTAAPDIGADLTDLFNSLTGYSRKLSYRNLLVAPHGIRAGIIDRVEREVAAHRAEGAHNGKGRIRLKMNALVDEQVIDALYRASRAGVRIEVVVRGICALRPGAQGISENIIVRSILGRFLEHSRILHFRAIDEFWIGSADMMHRNLDRRVEVMAQVKNPRLTAQLDELFESALDPCTRCWELGPDGQWTASPQEGHSVRDHQESLMERHRSP

>19|CORE_REP|Org118_Gene1392#

MTLTPEASKSVAQPPTQAPLTQEEAIASLGRYGYGWADSDVAGANAQRGLSEAVVRDISAKKNEPDWMLQSRLKALRIFDRKPIPKWGSNLDGIDFDNIKYFVRSTEKQAASWDDLPEDIRNTYDRLGIPEAEKQRLVAGVAAQYESEVVYHQIREDLEAQGVIFLDTDTGLREHPDIFKEYFGTVIPAGDNKFSALNTAVWSGGSFIYVPPGVHVDIPLQAYFRINTENMGQFERTLIIADEGSYVHYVEGCLPPGGELITTADGDLRPIESIRVGDFVTGHDGRPHRVTAVQVRDLDGELFTFTPMSPANAFSVTAEHPLLAIPRDEVRVMRKERNGWKAEVNSTKLRSAEPRWIAAKDVAEGDFLIYPKPKPIPHRTVLPLEFARLAGYYLAEGHACLTNGCESLIFSFHSDEFEYVEDVRQACKSLYEKSGSVLIEEHKHSARVTVYTKAGYAAMRDNVGIGSSNKKLSDLLMRQDETFLRELVDAYVNGDGNVTRRNGAVWKRVHTTSRLWAFQLQSILARLGHYATVELRRPGGPGVIMGRNVVRKDIYQVQWTEGGRGPKQARDCGDYFAVPIKKRAVREAHEPVYNLDVENPDSYLAYGFAVHNCTAPIYKSDSLHSAVVEIIVKPHARVRYTTIQNWSNNVYNLVTKRARAEAGATMEWIDGNIGSKVTMKYPAVWMTGEHAKGEVLSVAFAGEDQHQDTGAKMLHLAPNTSSNIVSKSVARGGGRTSYRGLVQVNKGAHGSRSSVKCDALLVDTVSRSDTYPYVDIREDDVTMGHEATVSKVSENQLFYLMSRGLTEDEAMAMVVRGFVEPIAKELPMEYALELNRLIELQMEGAVG

>21|CORE_REP|Org20_Gene1819#

MTDRVSVGNLRIARVLYDFVNNEALPGTDIDPDSFWAGVDKVVADLTPQNQALLNARDELQAQIDKWHRRRVIEPIDMDAYRQFLTEIGYLLPEPDDFTITTSGVDAEITTTAGPQLVVPVLNARFALNAANARWGSLYDALYGTDVIPETDGAEKGPTYNKVRGDKVIAYARKFLDDSVPLSSGSFGDATGFTVQDGQLVVALPDKSTGLANPGQFAGYTGAAESPTSVLLINHGLHIEILIDPESQVGTTDRAGVKDVILESAITTIMDFEDSVAAVDAADKVLGYRNWLGLNKGDLAAAVDKDGTAFLRVLNRDRNYTAPGGGQFTLPGRSLMFVRNVGHLMTNDAIVDTDGSEVFEGIMDALFTGLIAIHGLKASDVNGPLINSRTGSIYIVKPKMHGPAEVAFTCELFSRVEDVLGLPQNTMKIGIMDEERRTTVNLKACIKAAADRVVFINTGFLDRTGDEIHTSMEAGPMVRKGTMKSQPWILAYEDHNVDAGLAAGFSGRAQVGGHVDNAELMADMVETKIAQPRAGASTAWVPSPTAATLHALHYHQVDVAAVQQGLAGKRRATIEQLLTIPLAKELAWAPDEIREEVDNNCQSILGYVVRWVDQGVGCSKVPDIHDVALMEDRATLRISSQLLANWLRHGVITSADVRASLERMAPLVDRQNAGDVAYRPMAPNFDDSIAFLAAQELILSGAQQPNGYTEPSMPDVVGSLRPGPLRSRPHRTGPVTMRPASGPLWGHRPLAGESRPHRAEPVRSSCLTGAGLRRRGRRRRYG

>22|CORE_REP|Org51_Gene3937#

MAIAETDTEVHTPFEQDFEKDVAATQRYFDSSRFAGIIRLYTARQVVEQRGTIPVDHIVAREAAGAFYERLRELFAARKSITTFGPYSPGQAVSMKRMGIEAIYLGGWATSAKGSSTEDPGPDLASYPLSQVPDDAAVLVRALLTADRNQHYLRLQMSERQRAATPAYDFRPFIIADADTGHGGDPHVRNLIRRFVEVGVPGYHIEDQRPGTKKCGHQGGKVLVPSDEQIKRLNAARFQLDIMRVPGIIVARTDAEAANLIDSRADERDQPFLLGATKLDVPSYKSCFLAMVRRFYELGVKELNGHLLYALGDSEYAAAGGWLERQGIFGLVSDAVNAWREDGQQSIDGIFDQVESRFVAAWEDDAGLMTYGEAVADVLEFGQSEGEPIGMAPEEWRAFAARASLHAARAKAKELGADPPWDCELAKTPEGYYQIRGGIPYAIAKSLAAAPFADILWMETKTADLADARQFAEAIHAEFPEQMLAYNLSPSFNWDTTGMTDEEMRRFPEELGKMGFVFNFITYGGHQIDGVAAEEFATALRQDGMLALARLQRKMRLVESPYRTPQTLVGGPRSDAALAASSGRTATTKAMGKGSTQHQHLVQTEVPRKLLEEWLAMWSGHYQLKDKLRVQLRPQRAGSEVLELGIHGESDDKLANVIFQPIQDRRGRTILLVRDQNTFGAELRQKRLMTLIHLWLVHRFKAQAVHYVTPTDDNLYQTSKMKSHGIFTEVNQEVGEIIVAEVNHPRIAELLTPDRVALRKLITKEA

>38|CORE_REP|Org2_Gene1795#

MSPQLCPKVSIVSTTHNQAGYARQAFDSFLDQQTDFPVEIIVADDASTDATPAIIREYAERYPHVFRPIFRTENLGLNGNLTGALSAARGEYVALCEADDYWIDPLKLSKQVAFLDRHPKTTVCFHPVRVIWEDGHAKDSKFPPVRVRGNLSLDALILMNFIQTNSAVYRRLERYDDIPADVMPLDWYLHVRHAVHGDIAMLPDTMAVYRRHAQGMWHNQVVDPPKFWLTQGPGHAATFDAMLDLFPGDPAREELIAVMADWILRQIANVPGPEGAPRCRKPSRAIPGSPCWRCSTAGDTRAAAQDPVAQARRRDAEPQGARGCVALPAPTRLSSLTMSTNPGPAEGANQVMAQEHSAGAVQFTAHNVRLDDGTLTIPESSRTLDESSWFISARGILETVFPGDKSHLRLADVGCLEGGYAVGFARMGFQVLGIEVRELNMAACNYIKSKTNLPNLRFVHDNALNIANHGLFDTVFCCGLFYHLENPKQYLETLSSVTNKLLILQTHFSIINRSDKWLRLPTTARQLTDRLLRRPAPVKFMLSAPTEHEGLPGRWFTEFSDDRSFGQRDTAKWASWDNRRSFWIQREHLLQAIKDVGVDLVMEEYDNLEPSIAESLLGGSYAANLRGTFIGIKTR

>43|CORE_REP|Org1_Gene1647#

MASRQTPAELARCDLAKTAEREHTPTATATTPSVAGNVMPMSVRSLPAALRACARLQPHDPAFTFMDYEQDWDGVAITLTWSQLYRRTLNVAQELSRCGSTGDRVVISAPQGLEYVVAFLGALQAGRIAVPLSVPQGGVTDERSDSVLSDSSPVAILTTSSAVDDVVQHVARRPGESPPSIIEVDLLDLDAPNGYTFKEDEYPSTAYLQYTSGSTRTPAGVVMSHQNVRVNFEQLMSGYFADTDGIPPPNSALVSWLPFYHDMGLVIGICAPILGGYPAVLTSPVSFLQRPARWMHLMASDFHAFSAAPNFAFELAARRTTDDDMAGRDLGNILTILSGSERVQAATIKRFADRFARFNLQERVIRPSYGLAEATVYVATSKPGQPPETVDFDTESLSAGHAKPCAGGGATSLISYMLPRSPIVRIVDSDTCIECPDGTVGEIWVHGDNVANGYWQKPDESERTFGGKIVTPSPGAPEGPWLRTGDSGFVTDGKMFIIGRIKDLLIVYGRNHSPDDIEATIQEITRGRCAAISVPGDRSTEKLVAIIELKKRGDSDQDAMARLGAIKREVTSALSSSHGLSVADLVLVAPGSIPITTSGKVRRGACVEQYRQDQFARLDA

>70|CORE_REP|Org20_Gene3484#

MDFGALPPEINSARMYAGPGSASLVAAAKMWDSVASDLFSAASAFQSVVWGLTVGSWIGSSAGLMAAAASPYVAWMSVTAGQAQLTAAQVRVAAAAYETAYRLTVPPPVIAENRTELMTLTATNLLGQNTPAIEANQAAYSQMWGQDAEAMYGYAATAATATEALLPFEDAPLITNPGGLLEQAVAVEEAIDTAAANQLMNNVPQALQQLAQPAQGVVPSSKLGGLWTAVSPHLSPLSNVSSIANNHMSMMGTGVSMTNTLHSMLKGLAPAAAQAVETAAENGVWAMSSLGSQLGSSLGSSGLGAGVAANLGRAASVGSLSVPPAWAAANQAVTPAARALPLTSLTSAAQTAPGHMLGGLPLGHPRRQRYPAHWRRHGPTRYPAHRPPDSTTGLRGCVGVVPRRGWRALAIWSKGPDPTGRTPRHRGAVDGIRKAVTGNGIDAGTTIRDDHDGRRQPRDLDGRCYAGDQPARAGLTMAQAGGNLPDQQPTHQRVSRFPAALRSTNRPPRHGRRRRHLVGNKCGRHQRGFACVIPSHRV

>73|CORE_REP|Org87_Gene1778#

MAATKASTATDEPVKRTATKSPAASASGAKTGAKRTAAKSASGSPPAKRATKPAARSVKPASAPQDTTTSTIPKRKTRAAAKSAAAKAPSARGHATKPRAPKDAQHEAATDPEDALDSVEELDAEPDLDVEPGEDLDLDAADLNLDDLEDDVAPDADDDLDSGDDEDHEDLEAEAAVAPGQTADDDEEIAEPTEKDKASGDFVWDEDESEALRQARKDAELTASADSVRAYLKQIGKVALLNAEEEVELAKRIEAGLYATQLMTELSERGEKLPAAQRRDMMWICRDGDRAKNHLLEANLRLVVSLAKRYTGRGMAFLDLIQEGNLGLIRAVEKFDYTKGYKFSTYATWWIRQAITRAMADQARTIRIPVHMVEVINKLGRIQRELLQDLGREPTPEELAKEMDITPEKVLEIQQYAREPISLDQTIGDEGDSQLGDFIEDSEAVVAVDAVSFTLLQDQLQSVLDTLSEREAGVVRLRFGLTDGQPRTLDEIGQVYGVTRERIRQIESKTMSKLRHPSRSQVLRDYLD

>75|CORE_REP|Org9_Gene1083#

MHADLAATTSREDFRLLAAEHRVVPVTRKVLADSETPLSAYRKLAANRPGTFLLESAENGRSWSRWSFIGAGAPTALTVREGQAVWLGAVPKDAPTGGDPLRALQVTLELLATADRQSEPGLPPLSGGMVGFFAYDMVRRLERLPERAVDDLCLPDMLLLLATDVAAVDHHEGTITLIANAVNWNGTDERVDWAYDDAVARLDVMTAALGQPLPSTVATFSRPEPRHRAQRTVEEYGAIVEYLVDQIAAGEAFQVVPSQRFEMDTDVDPIDVYRILRVTNPSPYMYLLQVPNSDGAVDFSIVGSSPEALVTVHEGWATTHPIAGTRWRGRTDDEDVLLEKELLADDKERAEHLMLVDLGRNDLGRVCTPGTVRVEDYSHIERYSHVMHLVSTVTGKLGEGRTALDAVTACFPAGTLSGAPKVRAMELIEEVEKTRRGLYGGVVGYLDFAGNADFAIAIRTALMRNGTAYVQAGGGVVADSNGSYEYNEARNKARAVLNAIAAAETLAAPGANRSGC

>86|CORE_REP|Org118_Gene1678#

MAEESRGQRGSGYGLGLSTRTQVTGYQFLARRTAMALTRWRVRMEIEPGRRQTLAVVASVSAALVICLGALLWSFISPSGQLNESPIIADRDSGALYVRVGDRLYPALNLASARLITGRPDNPHLVRSSQIATMPRGPLVGIPGAPSSFSPKSPPASSWLVLGDTVATSSSIGSLQGVTVTVIDGTPDLTGHRQILSGSDAVVLRYGGDAWVIREGRRSRIEPTNRAVLLPLGLTPEQVSQARPMSRALFDALPVGPELLVPEVPNAGGPATFPGAPGPIGTVIVTPQISGPQQYSLVLGDGVQTLPPLVAQILQNAGSAGNTKPLTVEPSTLAKMPVVNRLDLSAYPDNPLEVVDIREHPSTCWWWERTAGENRARVRVVSGPTIPVAATEMNKVVSLVKADTSGRQADQVYFGPDHANFVAVTGNNPGAQTSESLWWVTDAGARFGVEDSKEARDALGLTLTPSLAPWVALRLLPQGPTLSRADALVEHDTLPMDMTPAELVVPK

>123|CORE_REP|Org94_Gene1437#

MNWTVDIPIDQLPSLPPLPTDLRTRLDAALAKPAAQQPTWPADQALAMRTVLESVPPVTVPSEIVRLQEQLAQVAKGEAFLLQGGDCAETFMDNTEPHIRGNVRALLQMAVVLTYGASMPVVKVARIAGQYAKPRSADIDALGLRSYRGDMINGFAPDAAAREHDPSRLVRAYANASAAMNLVRALTSSGLASLHLVHDWNREFVRTSPAGARYEALATEIDRGLRFMSACGVADRNLQTAEIYASHEALVLDYERAMLRLSDGEDGEPQLFDLSAHTVWIGERTRQIDGAHIAFAQVIANPVGVKLGPNMTPELAVEYVERLDPHNKPGRLTLVSRMGNHKVRDLLPPIVEKVQATGHQVIWQCDPMHGNTHESSTGFKTRHFDRIVDEVQGFFEVHRALGTHPGGIHVEITGENVTECLGGAQDISETDLAGRYETACDPRLNTQQSLELAFLVAEMLRD

>146|CORE_REP|Org118_Gene2595#

MDFGALPPEVNSARMYGGAGAADLLAAAAAWNGIAVEVSTAASSVGSVITRLSTEHWMGPASLSMAAAVQPYLVWLTCTAESSALAAAQAMASAAAFETAFALTVPPAEVVANRALLAELTATNILGQNVSAIAATEARYGEMWAQDASAMYGYAAASAVAARLNPLTRPSHITNPAGLAHQAAAVGQAGASAFARQVGLSHLISDVADAVLSFASPVMSAADTGLEAVRQFLNLDVPAVRRIRVSRPGWRGRLCHGRHWQYDASCRCYGNRWRSRSRWRRGSRGGTRGCPSGRRRNSADRRFGQCVRGWSPVGAGKLVYCSAGDGSRRGLGWHRLGSSRGGRPDRSDAACPWNGRSRQQCWCRLRTTVRSQADCYAQARPLLIWRHRDKRTRPTSAPPRPHCANPPAARVSFRCCGERSGATLKRIVLRLPVPA

>164|CORE_REP|Org59_Gene1167#

MAEIVLDHVNKSYPDGHTAVRDLNLTIADGEFLILVGPSGCGKTTTLNMIAGLEDISSGELRIAGERVNEKAPKDRDIAMVFQSYALYPHMTVRQNIAFPLTLAKMRKADIAQKVSETAKILDLTNLLDRKPSQLSGGQRQRVAMGRAIVRHPKAFLXXEPLSNLDAKLRVQMRGEIAQLQRRLGTTTVYVTHDQTEAMTLGDRVVVMYGGIAQQIGTPEELYERPANLFVAGFIGSPAMNFFPARLTAIGLXLPFGEVTLAPEVQGVIAAHPKPENVIVGVRPEHIQDAALIDAYQRIRALTFQVKVNLVESLGADKYLYFTTESPAVHSVQLDELAEVEGESALHENQFVARVPRRVQGSHRAVGRVGFRYRQTCRLRRRLRCEPDHSAPRLMAASXHISPXHAXGFGAFCVCSPTRS

>165|CORE_REP|Org59_Gene1373#

MTASVNSLDLAAIRADFPILKRIMRGGNPLAYLDSGATSQRPLQVLDAEREFLTASNGAVHRGAHXLMEEATDAYEQGRADIVGGVIRRQXTRTSWCSPKMPPRRSTWCHMCWGTAVSSVTXGPGDVIVTTELEHHANLIPWQELARRTGATLRWXGVTDDGRIDLDSLYLDDRVKVVAFTHHSNVTGVLTPVSELVSRAHQSGALTVLXXCQSVPHQPVDLHELGVDFAAFSGHKMLGPNGIGVLYGRRELLAQMPPFLTGGSMIETVTXEGATYAPAPQRFEAGTPMTSQVVGLAAAARYLGAIGMAAVEAHERELVAAAIEGLSGIDGVRILGPTSMRDRGSPVAFVVEGVHAHDVGQVLDDGGVAVRVGHHCALPLHRRFGLAATARASFAVYNTADEVDRLVAGVRRSRHFFGRA

>190|CORE_REP|Org69_Gene2725#

MVEAGTRDPLESALLDSRYLVQAKIASGGTSTVYRGLDVRLDRPVALKVMDSRYAGDEQFLTRFRLEARAVARLNNRALVAVYDQGKDGRHPFLVMELIEGGTLRELLIERGPMPPHAVVAVLRPVLGGLAAAHRAGLVHRDVKPENILISDDGDVKLADFGLVRAVAAASITSTGVILGTAAYLSPEQVRDGNADPRSDVYSVGVLVYELLTGHTPFTGDSALSIAYQRLDADVPRASAVIDGVPPQFDELVACATARNPADRYADAIAMGADLEAIAEELALPEFRVPAPRNSAQHRSAALYRSRITQQGQLGAKPVHHPTRQLTRQPGDCSEPASGSEPEHEPITGQFAGIAIEEFIWARQHARRMVLVWVSVVLAITGLVASAAWTIGSNLSGLL

>200|CORE_REP|Org59_Gene1665#

MDQQSTRTDITVNVDGFWMLQALLDIRHVAPELRCRPYVSTDSNDWLNEHPGMAVMREQGIVVNDAVNEQVAARMKVLAAPDLEVVALLSRGKLLYGVIDDENQPPGSRDIPDNEFRVVLARRGQHWVSAVRVGNDITVDDVTVSDSASIAALVMDGLESIXXAXPAAINAVNVPMEEMLEATKSWQESGFNVFSGGDLRRMGISAATVAALGQALSDPAAEVAVYARQYRDDAKXPSASXLSLKDGSGGRIALYQQARTAGXRRGXAGYLPXYPAVGASRSEDRFGYTALRRVENTQQSMTPGRETRSTTTNLSIRYNPDTYRANCSRIDCNTARQGQPQRFGREARXXKSELXEPQLPVGYRASVPTPTELPAPLKPRCNTFAMAGGTGR

>226|CORE_REP|Org59_Gene452#

MQLTPHFGNVQAHYDLSDDFFRLFLDPTQTYSCAYFERDDMTLQEAQIAKIDLALGKLNLEPGMTLLDIGCGWGATMRRAIEKYDVNVVGLTLSENQAGHVQKMFDQMDTPRSRRVLLEGWEKFDEPVDRIVSIGAFEHFGHQRYHHFFEVTHRTLPADGKMLLHTIVRPTFKEGREKGLTLTHELVHFTKFILAEIFPGGWLPSIPTVHEYAEKVGFRVTAVQSLQLHYARTLDMWATALEANKDQAIAIZSQTVYBSLHEVPDRLREAVPPGLHRRRPVHTGKVTGQSALAXXRPVPGRXATPGVSSATPGTXSGGDGLXGQCELSHVADALAEEVLTSGQIVHVFVVNLLGLKSNGAVLVSLQIRRPDV

>238|CORE_REP|Org3_Gene1855#

MANVQYSAVTQRYPGADAPTVDNLDLDIADGEFLVLVGPSGCGKSTTLRVLAGLEPIESGRISIGDVDVTHLPPRARDVAMVFQNYALYPNMTVAANMGFALRNAGMSRADTRRRVLEVADMLELTDLLDRKPAKLSGGQRQRVAMGRAIVRRPRVFCMDEPLSNLDAKLRVSTRSQISGLQRRLGTTTVYVTHDQVEAMTMGDRVAVLKDGVLQQVDTPRALYDDPVNTFVATFIGAPAMNLIDAAVAHGVVRAPDLAIPVPDPAAERVLVGVRPESWDVASIGTPGSLTVHVELVEELGFESFVYATPVDQRGWSSRAPRIVFRTDRRTAVRVGESLAIVPHSQEVRLFNSRTETRLR

>271|CORE_REP|Org14_Gene116#

MTFFEQVRRLRSAATTLPRRLAIAAMGAVLVYGLVGTFGGPATAGAFSRPGLPVEYLQVPSASMGRDIKVQFQGGGPHAVYLLDGLRAQDDYNGWDINTPAFEEYYQSGLSVIMPVGGQSSFYTDWYQPSQSNGQNYTYKWETFLTREMPAWLQANKGVSPTGNAAVGLSMSGGSALILAAYYPQQFPYAASLSGFLNPSEGWWPTLIGLAMNDSGGYNANSMWGPSSDPAWKRNDPMVQIPRLVANNTRIWVYCGNGTPSDLGGDNIPAKFLEGLTLRTNQTFRDTYAADGGRNGVFNFPPNGTHSWPYWNEQLVAMKADIQHVLNGATPPAAPAAPAA

>281|CORE_REP|Org119_Gene2767#

MAGAKHAGRIVAITTAAAVILAACSSGSKGGAGSGHAGKARSAVTTTDADWKPVADALGRSGKLGDNNTAYRINLPRNDLHITSYGVDIKPGLSLGGYAAFARYDNNETLLMGDLVITEEELPKVTDALQAHGIAQTALHKHLLQQDPPVWWTHIHGMGDAARLAQGLKAALDATTIGPPTPPPARQPPVDIDVAGVDQALGRKGTQDGGLLKYSIPRKDTIIEDGHVLPAVSLNLTTVINFQPVGRGRAAINGDFILIAPEVQEVIRAMRAGNITIVELHNHGLTEEPRLFYMHYWAVDDAVTLARALRPAMECHQPAVVIIPMQPHKGWCG

>283|CORE_REP|Org59_Gene2384#

MGGLTISDLVVEYSSGGXAVRPIDGXKPRRGAGVAGDLAWAQRLREDDPLVLPRRXXCARSPAQSSLTMSTSSNLXEGAALAKYRRDKXGIVFQAFNLVSSLTALENVMVPLRAAGVSRAAARKRAEDLLIRVNLGERMKHRPGDMSGGQQQRVAVARAIALDPQLILADEPTAHLDFIQVEEVLRLIRSLAQGDRVVVVATHDSRMLPLADRVLELMPAQVSPNQPPETVHVKAGEVLFEQSTMGDLIYVVSEGEFEIVRELADGGEELVKXAAPGDYFGEIGVLFXLPRSATVRARSDATAVGYTAQAFRERLGXXRXXDLIEHRELASE

>284|CORE_REP|Org18_Gene2243#

MRLLVTGGAGFIGTNFVHSAVREHPDDAVTVLDALTYAGRRESLADVEDAIRLVQGDITDAELVSQLVAESDAVVHFAAESHVDNALDNPEPFLHTNVIGTFTILEAVRRHGVRLHHISTDEVYGDLELDDRARFTESTPYNPSSPYSATKAGADMLVRAWVRSYGVRATISNCSNNYGPYQHVEKFIPRQITNVLTGRRPKLYGAGANVRDWIHVDDHNSAVRRILDRGRIGRTYLISSEGERDNLTVLRTLLRLMDRDPDDFDHVTDRVGHDLRYAIDPSTLYDELCWAPKHTDFEEGLRTTIDWYRDNESWWRPLKDATEARYQERGQ

>285|CORE_REP|Org146_Gene224#

MNPIPSWPGRGRVTLVLLAVVPVALAYPWQSTRDYVLLGVAAAVVIGLFGFWRGLYFTTIARRGLAILRRRRRIAEPATCTRTTVLVWVGPPASDTNVLPLTLIARYLDRYGIRADTIRITSRVTASGDCRTWVGLTVVADDNLAALQARSARIPLQETAQVAARRLADHLREIGWEAGTAAPDEIPALVAADSRETWRGMRHTDSDYVAAYRVSADAELPDTLPAIRSRPAQETWIALEIAYAAGSSTRYTVAAACALRTDWRPGGTAPVAGLLPQHGNHVPALTALDPRSTRRLDGHTDAPADLLTRLHWPTPTAGAHRAPLTNAVSRT

>311|CORE_REP|Org118_Gene1438#

MNAHTSVGPLDRAARVYIAGHRGLVGSALLRTFAGAGFTNLLVRSRAELDLTDRAATFDFVLESRPQVVIDAAARVGGILANDTYPADFLSENLQIQVNLLDAAVAARVPRLLFLGSSCIYPKLAPQPIPESALLTGPLEPTNDAYAIAKIAGILAVQAVRRQHGLPWISAMPTNLYGPGDNFSPSGSHLLPALIRRYDEAKASGAPNVTNWGTGTPRRELLHVDDLASACLYLLEHFDGPTHVNVGTGIDHTIGEIAEMVASAVGYSGETRWDPSKPDGTPRKLLDVSVLREAGWRPSIALRDGIEATVAWYREHAGTVRQ

>325|CORE_REP|Org59_Gene286#

MDATPNAVELTVDNAWFIAETIGAGTFPWVLAITMPYSDAAQRGAFVDRQRDELTRMGLLSPQGVINPAVADWIKVVCFPDRWLDLRYVGPASADGACELLRGIVALRTGTGKTSNKTGNGVVALRNAQLVTFTAMDIDDPRALVPILGXGLAHRPPARFDEFSLPTRVGARADERLRSGVPLGEVVDYLGIPASARPVVESVFSGPRSYVEIVAGCNRSXERRXHHHRGRPKHRRHLGGPGVGESVAGIRRRVGLHLQPWDTVCDRRRDPNTDRVLARRAMVPGTAGVAGLLHPILVIRNQKVSTMSQERSR

>339|CORE_REP|Org30_Gene950#

MDRCCQRATAFACALRPTKLIDYEEMFRGAMQARAMVANPDQWADSDRDQVNTRHYLSTSMRVALDRGEFFLVYQPIIRLADNRIIGAEALLRWEHPTLGTLLPGRFIDRAENNGLMVPLTAFVLEQACRHVRSWRDHSTDPQPFVSVNVSASTICDPGFLVLVEGVLGETGLPAHALQLELAEDARLSRDEKAVTRLQELSALGVGIAIDDFGIGFSSLAYLPRLPVDVVKLGGKFIECLDGDIQARLANEQITRAMIDLGDKLGITVTAKLVETPSQAARLRAFGCKAAQGWHFAKALPVDFFRE

>349|CORE_REP|Org98_Gene3124#

MAGRSERLVITGAGGQLGSHLTAQAAREGRDMLALTSSQWDITDPAAAERIIRHGDVVINCAAYTDVDGAESNEAVAYAVNATGPQHLARACARVGARLIHVSTDYVFDGDFGGAEPRPYEPTDETAPQGVYARSKLAGEQAVLAAFPEAAVVRTAWVYTGGTGKDFVAVMRRLAAGHGRVDVVDDQTGSPTYVADLAEALLALADAGVRGRVLHAANEGVVSRFGQARAVFEECGADPQRVRPVSSAQFPRPAPRSSYSALSSRQWALAGLTPLRHWRSALATALAAPANSTSIDRRLPSTRD

>357|CORE_REP|Org149_Gene3036#

MTRMAEKPISPTKTRTRFEDIQAHYDVSDDFFALFQDPTRTYSCAYFEPPELTLEEAQYAKVDLNLDKLDLKPGMTLLDIGCGWGTTMRRAVERFDVNVIGLTLSKNQHARCEQVLASIDTNRSRQVLLQGWEDFAEPVDRIVSIEAFEHFGHENYDDFFKRCFNIMPADGRMTVQSSVSYHPYEMAARGKKLSFETARFIKFIVTEIFPGGRLPSTEMMVEHGEKAGFTVPEPLSLRPHYIKTLRIWGDTLQSNKDKAIEVTSEEVYNRYMKYLRGCEHYFTDEMLDCSLVTYLKPGAAA

>358|CORE_REP|Org4_Gene3919#

MGWRDAPALSDYQHVASGKVREIYRVDDEHLLLVASDRISAYDYVLDSTIPDKGRVLTAMSAFFFGLVDAPNHLAGPPDDPRIPDEVLGRALVVRRLEMLPVECVARGYLTGSGLLDYQATGKVCGIALPPGLVEASRFATPLFTPATKAALGDHDENISFDRVVEMVGALRANQLRDRTLQTYVQAADHALTRGIIIADTKFEFGIDRHGNLLLADEIFTPDSSRYWPADDYRAGVVQTSFDKQFVRSWLTGSESGWDRGSDRPPPPLPEHIVEATRARYINAYERISELKFDDWIGPGA

>374|CORE_REP|Org119_Gene587#

MAKLRPYYEESQSAYDISDDFFALFLDPTWVYTCAYFERDDMTLEEAQLAKVDLALDKLNLEPGMTLLDVGCGWGGALVRAVEKYDVNVIGLTLSRNHYERSKDRLAAIGTQRRAEARLQGWEEFEENVDRIVSFEAFDAFKKERYLTFFERSYDILPDDGRMLLHSLFTYDRRWLHEQGIALTMSDLRIPQIPAGVDLPGRRAAIGARHCRQCAGRGLHHRACPAAAAALRTDSRCMGRQPTGCPRTRHRRTVRRGLQQLHALSDRMRGALPQRPNQRRPVHHDQVARPLISVP

>388|CORE_REP|Org118_Gene328#

MRGIILAGGSGTRLYPITMGISKQLLPVYDKPMIYYPLTTLMMAGIRDIQLITTPHDAPGFHRLLGDGAHLGVNISYATQDQPDGLAQAFVIGANHIGADSVALVLGDNIFYGPGLGTSLKRFQSISGGAIFAYWVANPSAYGVVEFGAEGMALSLEEKPVTPKSNYAVPGLYFYDNDVIEIARGLKKSARGEYEITEVNQVYLNQGRLAVEVLARGTAWLDTGTFDSLLDAADFVRTLERRQGLKVSMPRRSGVAHGLDRRRAAGVQRARALVKSGYGNYLLELLERN

>442|CORE_REP|Org59_Gene2732#

MSMLARHGPRYGGSXNGHSDXSXGXAKXAAPTLYIFPHAGGTAKDYVAFSREFSADVKRIAVQYPGQHDRSGLPPLESIPTLADEIFAMMKPSARIDDPVAFFGHSMGGMLAFEVALRYQSAGHRVLAFFVSXXSAPGHIRYKQLQDLSDREMLDLFTRMTGMNPDFFTDXEFFVGALPTLRAVRAIAGYSCPPETKLSCPIYAFIGDKDWIATQDDMDPWRDRTTEEFXIRVFPGDHFYLNDNLPELVSDIEDKTLQWLXSXPXLCSGCS

>447|CORE_REP|Org119_Gene1362#

MTILEIKDLHVSVENPAEADHEIPILRGVDLTVKSGETHALMGPNGSGKSTLSYAIAGHPKYHVTSGTITLDGADVLAMSIDERARAGLFLAMQYPVEVPGVSMSNFLRSAATAIRGEPPKLRHWVKEVKAAMAALDIDPAFAERSVNEGFSGGEKKRHEILQLELLKPKIAILDETDSGPGRRRAARGQRGGEPLRRIPARRHPADHALHPHPALHPPGIRARVRRRPHRRVRWFGARRRTRPERLRAFLPRKRAVPPPTRANRSLT

>482|CORE_REP|Org31_Gene855#

MELLGGPRVGNTESQLCVADGDDLPTYCSANSEDLNITTITTLSPTSMSHPQQVRDDQWVEPSDQLQGTAVFDATGDKATMPSWDELVRQHADRVYRLAYRLSGNQHDAEDLTQETFIRVFRSVQNYQPGTFEGWLHRITTNLFLDMVRRRARIRMEALPEDYDRVPADEPNPEQIYHDARLGPDLQAALASLPPEFRAAVVLCDIEGLSYEEIGATLGVKLGTVRSRIHRGRQALRDYLAAHPEHGECAVHVNPVR

>483|CORE_REP|Org22_Gene721#

MTTMSGYTRSQRPRQAILGQLPRIHRADGSPIRVLLVDDEPALTNLVKMALHYEGWDVEVAHDGQEAIAKFDKVGPDVLVLDIMLPDVDGLEILRRVRESDVYTPTLFLTARDSVMDRVTGLTSGADDYMTKPFSLEELVARLRGLLRRSSHLERPADEALRVGDLTLDGASREVTRDGTPISLSSTEFELLRFLMRNPRRALSRTEILDRVWNYDFAGRTSIVDLYISYLRKKIDSDREPMIHTVRGIGYMLRPPE

>502|CORE_REP|Org111_Gene1716#

MSGHSKWATTKHKKAVVDARRGKMFARLIKNIEVAARVGGGDPAGNPTLYDAIQKAKKSSVPNENIERARKRGAGEEAGGADWQTIMYEGYAPNGVAVLIECLTDNRNRAASEVRVAMTRNGGTMADPGSVSYLFSRKGVVTLEKNGLTEDDVLAAVLEAGAEDVNDLGDSFEVISEPAELVAVRSALQDAGIDYESAEASFQPSVSVPVDLDGARKVFKLVDALEDSDDVQNVWTNVDVSDEVLAALDDE

>526|CORE_REP|Org47_Gene1050#

MRLARRARNILRRNGIEVSRYFAELDWERNFLRQLQSHRVSAVLDVGANSGQYARGLRGAGFAGRIVSFEPLPGPFAVLQRSASTDPLWECRRCALGDVDGTISINVAGNEGASSSVLPMLKRHQDAFPPANYVGAQRVPIHRLDSVAADVLRPNDIAFLKIDVQGFEKQVIAGGDSTVHDRCVGMQLELSFQPLYEGGMLIREALDLVDSLGFTLSGLQPGFTDPRNGRMLQADGIFFRGSD

>527|CORE_REP|Org83_Gene3136#

MPNFWALPPEINSTRIYLGPGSGPILAAAQGWNALASELEKTKVGLQSALDTLLESYRGQSSQALIQQTLPYVQWLTTTAEHAHKTAIQLTAAANAYEQARAAMVPPAMVRANRVQTTVLKAINWFGQFSTRIADKEADYEQMWFQDALVMENYWEAVQEAIQSTSHFEDPPEMADDYDEAWMLNTVFDYHNENAKEEVIHLVPDVNKERGPIELVTKVDKEGTIRLVYDGEPTFSYKEHPKF

>579|CORE_REP|Org147_Gene3012#

MDTMRQRILVVDDDASLAEMLTIVLRGEGFDTAVIGDGTQALTAVRELRPDLVLLDLMLPGMNGIDVCRVLRADSGVPIVMLTAKTDTVDVVLGLESGADDYIMKPFKPKELVARVRARLRRNDDEPAEMLSIADVEIDVPAHKVTRNGEQISLTPLEFDLLVALARKPRQVFTRDVLLEQVWGYRHPADTRLVNVHVQRLRAKVEKDPENPTVVLTVRGVGYKAGPP

>587|CORE_REP|Org133_Gene366#

MTSVLIVEDEESLADPLAFLLRKEGFEATVVTDGPAALAEFDRAGADIVLLDLMLPGMSGTDVCKQLRARSSVPVIMVTARDSEIDKVVGLELGADDYVTKPYSARELIARIRAVLRRGGDDDSEMSDGVLESGPVRMDVERHVVSVNGDTITLPLKEFDLLEYLMRNSGRVLTRGQLIDRVWGADYVGDTKTLDVHVKRLRSKIEADPANPVHLVTVRGLGYKLEG

>591|CORE_REP|Org40_Gene740#

MTLVLVIDDEPQILRALRINLTVRGYQVITASTGAGALRAAAEHPPDVVILDLGLPDMSGIDVLGGLRGWLTAPVIVLSARTDSSDKVQALDAGADDYVTKPFGMDEFLARLRAAVRRNTAAAELEQPVIETDSFTVDLAGKKVIKDGAEVHLTPTEWGMLEMLARNRGKLVGRGELLKEVWGPAYATETHYLRVYLAQLRRKLEDDPSHPKHLLTESGMGYRFEA

>617|CORE_REP|Org59_Gene1254#

MAPDRADDDAERSDEEEWRLMTKLXVASRNRKKLAELRRVLDGAGLSGXTXLSXGDVSPLPETPETGVTFEDNALAKARDAFSATGLASVADDSGLEVAALGGMPGVLSARWSGRYGDDAANTALLLAQLCDVPDERRGAAFVSACALVSGSGEVVVRGEWPGTIAREPRGDGGFGYDPVFVPYGDDRTAAQLSPAEKDAVSHRGRALALLLPALRSLATG

>635|CORE_REP|Org109_Gene2034#

MVKVFLVDDHEVVRRGLVDLLGADPELDVVGEAGSVAEAMARVPAARPDVAVLDVRLPDGNGIELCRDLLSRMPDLRCLILTSYTSDEAMLDAILAGASGYVVKDIKGMELARAVKDVGAGRSLLDNRAAAALMAKLRGAAEKQDPLSGLTDQERTLLGLLSEGLTNKQIADRMFLAEKTVKNYVSRLLAKLGMERRTQAAVFATELKRSRPPGDGP

>636|CORE_REP|Org43_Gene263#

MTISFSSSNLRDDATSGNGDYRLDKLPETTPSTSVFDRADVTYRQFTELHGQARDTRREAHVVELESKTGERARCAPMHALEQLADYGFAWRDIARVVGVSVPAITKWRKGAGVTGENRLKIARLLALIDMLSDRFIGEPASWLEMPIQAGVGITRMDLLERGRYDLVLALASTHTGDGTVEYVLNETDKDWRETVVDNAFESYTAEDGVISIRPKR

>661|CORE_REP|Org9_Gene938#

MTKPTSAGQADDALVRLARERFDLPDQVRRLARPPVPSLEPPYGLRVAQLTDAEMLAEWMNRPHLAAAWEYDWPASRWRQHLNAQLEGTYSLPLIGSWHGTDGGYLELYWAAKDLISHYYDADPYDLGLHAAIADLSKVNRGFGPLLLPRIVASVFANEPRCRRIMFDPDHRNTATRRLCEWAGCKFLGEHDTTNRRMALYALEAPTTAA

>686|CORE_REP|Org120_Gene3695#

MKARELDVPGAWEITPTIHVDSRGLFFEWLTDHGFRAFAGHSLDVRQVNCSVSSAGVLRGLHFAQLPPSQAKYVTCVSGSVFDVVVDIREGSPTFGRWDSVLLDDQDRRTIYVSDGLAHGFLALQDNSTVMYLCSAEYNPQREHTICATDPTLAVDWPLVDGAAPSLSDRDAAAPSFEDVRASGLLPRWEQTQRFIGEMRGT

>696|CORE_REP|Org10_Gene3899#

MTECFLSDQEIRKLNRDLRILIAANGTLTRVLNIVADDEVIVQIVKQRIHDVSPKLSEFEQLGQVGVGRVLQRYIILKGRNSEHLFVAAESLIAIDRLPAAIITRLTQTNDPLGEVMAASHIETFKEEAKVWVGDLPGWLALHGYQNSRKRAVARRYRVISGGQPIMVVTEHFLRSVFRDAPHEEPDRLQFSNAITLAR

>703|CORE_REP|Org1_Gene1468#

MTVTDDYLANNVDYASGFKGPLPMPPSKHIAIVACMDARLDVYRMLGIKEGEAHVIRNAGCVVTDDVIRSLAISQRLLGTREIILLHHTDCGMLTFTDDDFKRAIQDETGIRPTWSPESYPDAVEDVRQSLRRIEVNPFVTKHTSLRGFVFDVATGKLNEVTPSSPSRQPRAHWRTGSPPRWGCVDSDREAWLHRWQ

>762|CORE_REP|Org119_Gene660#

MSRIGKQPIPVPAGVDVTIEGQSISVKGPKGTLGLTVAEPIKVARNDDGAIVVTRPDDERRNRSLHGLSRTLVSNLVTGVTQGYTTKMEIFGVGYRVQLKGSNLEFALGYSHPVVIEAPEGITFAVQAPTKFTVSGIDKQKVGQIAANIRRLRRPDPSKRGKGVRYEGEQIRRKVGKTGK

>828|CORE_REP|Org68_Gene1008#

MTLRLEQIYQDVILDHYKHPQHRGLREPFGAQVYHVNPICGDEVTLRVALSEDGTRVTDVSYDGQGCSISQAATSVLTEQVIGQRVPRALNIVDAFTEMVSSRGTVPGDEDVLGDGVAFAGVAKYPARVKCALLGWMAFKDALAQASEAFEEVTDERNQRTG

>852|CORE_REP|Org101_Gene1661#

MTKTTRLTPGDKAPAFTLPDADGNNVSLADYRGRRVIVYFYPAASTPGCTKQACDFRDNLGDFTTAGLNVVGISPDKPEKLATFRDAQGLTFPLLSDPDREVLTAWGAYGEKQMYGKTVQGVIRSTFVVDEDGKIVVAQYNVKATGHVAKLRRDLSV

>872|CORE_REP|Org57_Gene2255#

MTETTPAPQTPAAPAGPAQSFVLERPIQTVGRRKEAVVRVRLVPGTGKFDLNGRSLEDYFPNKVHQQLIKAPLVTVDRVESFDIFAHLGGGGPSGQAGALRLGIARALILVSPEDRPALKKAGFLTRDPRATERKKYGLKKARKAPQYSKR

>1109|CORE_REP|Org58_Gene1232#

MRLTPHEQERLLLSYAAELARRRRARGLRLNHPEAIAVIADHILEGARDGRTVAELMASGREVLGRDDVMEGVPEMLAEVQVEATFPDGTKLVTVHQPIA

>1214|CORE_REP|Org69_Gene2244#

MAKKDGAIEVEGRVVEPLPNAMFRIELENGHKVLAHISGKMRQHYIRILPEDRVVVELSPYDLSRGRIVYRYK
